# Regio- and Enantioselective Alkoxycarbonylation of Unactivated Terminal Alkenes under Palladium-Bromide-Monophosphine Catalysis

**DOI:** 10.1021/jacs.6c10237

**Published:** 2026-06-18

**Authors:** Michel Sigrist, Kuhali Das, Gracjan Kurpik, Wei Tian, Jianxun Huang, Ala Covas, Andrew D. Bond, Wenjun Tang, Paweł Dydio

**Affiliations:** † Yusuf Hamied Department of Chemistry, 2152 University of Cambridge, Cambridge CB2 1EW, U.K.; ‡ 27083 University of Strasbourg, CNRS, ISIS UMR 7006, 67000 Strasbourg, France; § State Key Laboratory of Bio-Organic and Natural Products Chemistry, Center for Excellence in Molecular Synthesis, Shanghai Institute of Organic Chemistry, 58309 University of Chinese Academy of Sciences, Shanghai 200032, China; ∥ School of Chemistry and Material Science, Hangzhou Institute for Advanced Study, University of Chinese Academy of Sciences, Hangzhou 310024, China

## Abstract

Regio- and stereoselective hydrocarbonylation of unactivated alkenes, including abundant light olefins produced on a large scale and a diverse range of other commercial and synthetic materials, remains a formidable challenge in fine-chemical synthesis. Here, we report a highly regio- and enantioselective alkoxycarbonylation of a range of unactivated terminal olefins with a variety of alcohols, including biologically relevant motifs, to afford valuable chiral esters in typically >95:5 enantiomeric ratios, >95:5 regioisomeric ratios, and >80% yields. Chiral alcohols bearing adjacent stereogenic centers can be transformed into either diastereomer of the products with >92:8 diastereomeric ratios and >80% yields. Central to this method is a palladium-bromide catalyst featuring a newly developed valley-shaped monophosphorus ligand, ValleyPhos. The crystal structure of the precatalyst (ValleyPhos)­PdBr_2_ reveals that the chiral ligand forms a deep asymmetric pocket around the metal center, enabling precise enantiocontrol. The bromide ligand, in turn, is essential for achieving branched regioselectivity and suppressing deleterious chain-walking processes.

## Graphical abstract

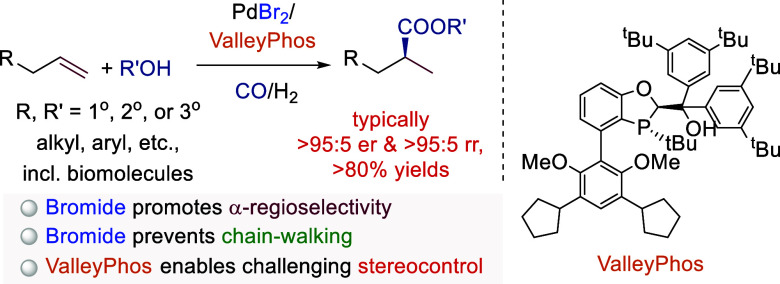

## Introduction

Chiral carboxylic esters and their derivatives are some of the most common structural motifs of various utility chemicals, including pharmaceuticals, agrochemicals, fragrances, and flavors (Figure 1a).^1^ Pd-catalyzed alkoxycarbonylation that installs carboxylic ester groups on abundant alkenes is attractive for the synthesis of such fine chemicals.^2^ Because these reactions can form different regio- and stereoisomers,^3,4^ the precise control of both regioselectivity and stereoselectivity remains crucial (Figure 1b). Significant progress in that respect was achieved with a series of simultaneously branch-selective and enantioselective methods for *activated* alkenes containing electronically biased double bonds, such as vinyl arenes, or those bearing specific directing groups (Figure 1b, bottom).^5^ In sharp contrast, advances in branch-selective transformations of unbiased *unactivated* terminal alkenes, such as α-olefins, are rare, with the prominent *racemic methods* reported by Beller^6^ and Bredenkamp.^7^ Most importantly, however, the *regio- and enantioselective* methods for these transformations remain unprecedented,^5a–5c^ constituting the goal of the current study (Figure 1c).

**Figure 1:**
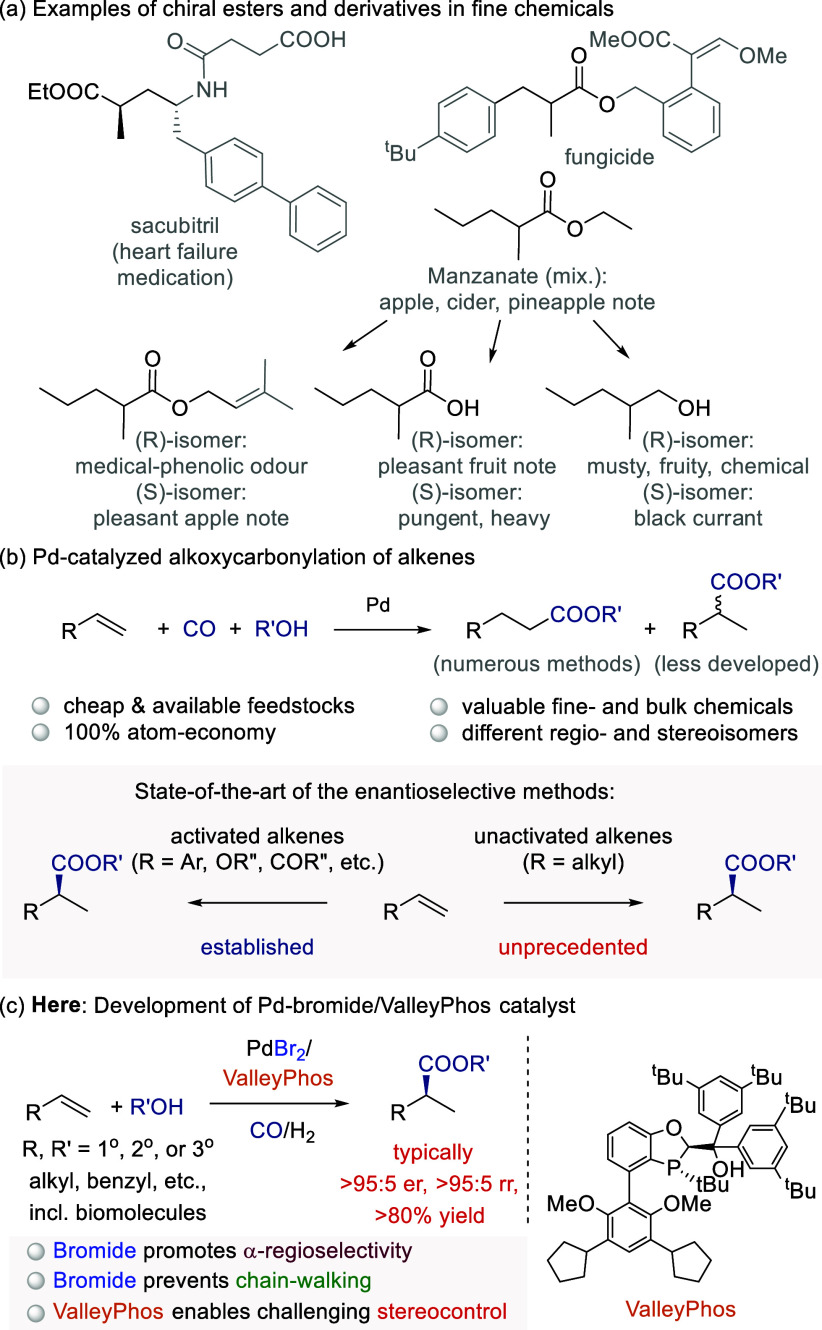
Context and summary of the current study.

The analysis of the mechanism of Pd-catalyzed alkoxycarbonylation reveals well-founded reasons for the simultaneous control of regio- and enantioselectivity for unactivated terminal alkenes to constitute a formidable challenge (Scheme 1).

- First, the target transformation requires the formation of a branched Pd alkyl intermediate, **iso-Pd** ^**alk**^, by the regioselective alkene insertion into the Pd–H bond of the palladium hydride intermediate, **PdH**. This requirement is difficult to meet,^6a^ given a minimal electronic bias of a double bond in an unactivated alkene, while the steric effects intrinsically favor the alternative regioisomeric intermediate (linear Pd alkyl, **n-Pd** ^**alk**^) due to the diminished steric requirements of the *n*-alkyl group versus those of the iso-alkyl group.
- Second, **iso-Pd** ^**alk**^ is intrinsically prone to undergoing β-hydride elimination on either side of its alkyl chain, which can be followed by subsequent reinsertion to an isomerized double bond,^6a,8^ leading to the detrimental chain-walking and eventually producing a mixture of site-isomer products.
- Most importantly, to be enantioselective, the catalyst must differentiate between both prochiral faces of the alkene double bond by distinguishing a small steric bias of a C–H bond and a flexible alkyl group.^9^ Without any additional attractions imposed by other functional groups, the effective enantioinduction for unactivated alkenes has proven persistently difficult in many catalytic processes.^9,10^

- In addition, the chiral ligand creating the chiral microenvironment around the Pd center remains remote from the reacting alkene, increasing the difficulty of achieving effective enantio-differentiation. At the same time, increasing the steric bulk of the ligand that could assist with the enantiocontrol inherently favors the wrong regioisomer, **n-Pd** ^**alk**^, over the target **iso-Pd** ^**alk**^.

**Scheme 1:**
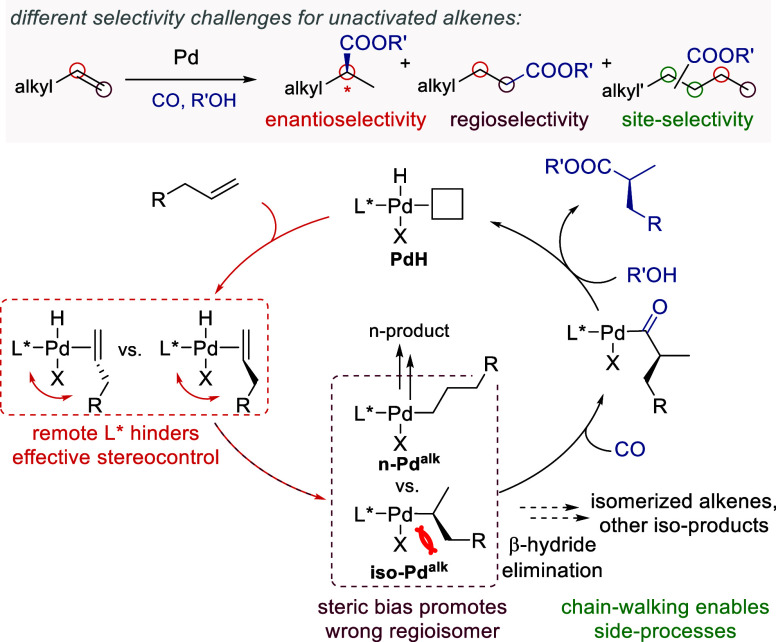
Mechanism and Different Challenges of Regio- and Enantioselective Alkoxycarbonylation of Unactivated Alkenes^a^

a L*, chiral monodentate ligand; X, halide; CO, solvent.

## Results and Discussion

We hypothesized that palladium-halide complexes bearing chiral valley-shaped monodentate ligands would be the catalysts of choice for enantioselective alkoxycarbonylation of unactivated terminal alkenes, simultaneously addressing all the above-listed challenges. Our reasoning was as follows. (1) The presence of a halide in the precursor promotes the formation of neutral Pd-hydride active species, which favor branched selective alkene insertion for unactivated olefins, as supported by previous empirical observations and recent mechanistic studies.^5j,6,11^ (2) Halide ligands bound to the metal center can modulate both alkene insertion and β-hydride elimination, thus aiding in regulating these critical steps toward promoting the productive reaction pathway and preventing the deleterious chain-walking processes. (3) The chiral valley-shaped ligands can create a deep pocket around the catalytic metal center, confining the reacting alkene molecule and thus promoting effective stereocontrol. Additionally, palladium–halide precatalysts can be activated with dihydrogen,^11a,12^ eliminating the need for cocatalysts, such as Bro̷nsted acids, which otherwise trigger Pd-independent alkene isomerization and other side reactions.^13^

To investigate our design, we focused on the alkoxycarbonylation of 1-octene (**1a**) and ethanol (**2a**) as model starting materials, which can react to form a mixture of several isomeric products **3aa**: target α-branched esters, linear regioisomer, and chain-walking-derived β- and γ-branched isomers (Figure 2). In the initial tests, we used a series of chiral monophosphorus ligands from different classes,^14^ including TADDOL-derived **L1** and **L2**,^15^ BINOL-derived **L3**,^16^ MOP **L4,** ^17^ and bidime **L5**,^18^ which previously proved effective in Pd-catalyzed enantioselective reactions^19^ or regio- and enantioselective functionalization of unactivated alkenes.^9,20^ We used PdI_2_ as a palladium precursor, the phosphorus complexes of which can be activated under a H_2_/CO atmosphere^11a,12^ and have been shown to be catalytically active in the alkoxycarbonylation of styrene.^12^

**Figure 2:**
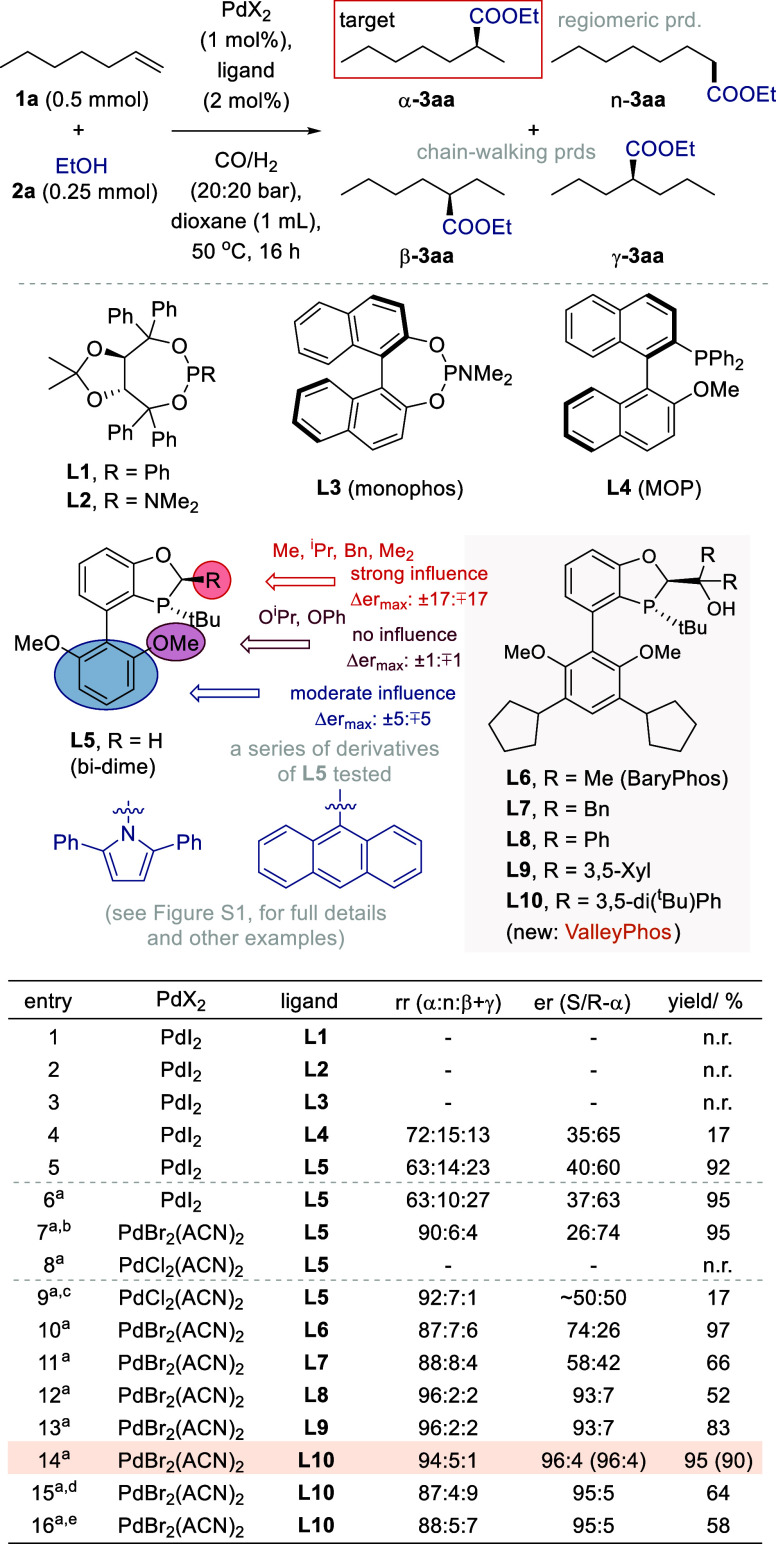
Reaction development—the effect of the chiral monophosphorus ligand and palladium halide precursor. Regioisomeric ratio (rr), enantiomeric ratio (er), and yield were determined by GC and NMR analyses; er and yield of the isolated material (major regioisomer) are reported in parentheses; the absolute configuration is assigned upon converting α-**3aa** and (*S*)-α-**3au** to the same major enantiomer of 2-methyloctan-1-ol. ^a^ Reaction in mtbe at 40 °C. ^b^ Similar selectivities for reactions with PdBr_2_(PhCN)_2_; sluggish reactions with PdBr_2_ due to limited solubility. ^c^ Reaction in mtbe at 100 °C. ^d^ **1a** (1 equiv), **2a** (1.2 equiv). ^e^ **1a** (1 equiv), **2a** (1 equiv).

We observed that PdI_2_ and bidime **L5** in 1,4-dioxane catalyzed the model reaction of 1-octene (**1a**) and ethanol (**2a**) to form **3aa** in 92% yield with high branched/linear regioselectivity (86:14 all b/l) and a modest level of enantioenrichment for the main α-regioisomer (60:40 enantiomeric ratio, er, entry 5, Figure 2). However, chain-walking processes produced β- and γ-esters in addition to the α-branched ester, resulting in a mixture of α-, n-, and β+γ-esters in a 63:14:23 regioisomeric ratio (rr). The evaluation of reaction conditions showed that lowering the reaction temperature and using mtbe as a solvent slightly improved both the branched/linear regioselectivity (90:10 all b/l) and enantioselectivity (63:37 er), although a substantial portion of chain-walking products was still formed (entry 6). Favorably, the exchange of palladium­(II) iodide for palladium­(II) bromide effectively suppressed the formation of the chain-walking products (entries 6 vs 7). Under such conditions, a mixture of PdBr_2_(ACN)_2_ and **L5** formulated a catalyst that formed **3aa** in 95% yield, producing the α-isomer as the main product with a 90:6:4 rr and an encouraging 74:26 er (entry 7).

To develop a more enantioselective catalyst than PdBr_2_(ACN)_2_/**L5**, we evaluated a library of commercial derivatives of **L5** bearing modifications to different parts of the framework. As summarized in Figure 2 (for details, see Figure S1), we observed that while changes to the lower aryl ring of **L5** had no or a moderate influence on the enantioselectivity (up to ± 5:∓5 er changes), introducing substituents onto the oxaphosphole ring modulated the enantioselectivity of the corresponding catalyst significantly (up to ± 17:∓17 er changes). Consequently, we focused on BaryPhos **L6** and its derivatives.^21^ Through the iterative changes to the structure of its isopropanol motif (entries 10–14 and Figure S1), we found that the installation of bulky 3,5-di-*tert*-butylphenyl rings constructed **L10**, coined ValleyPhos, which formed a catalyst with a superior selectivity profile. Precisely, the combination of PdBr_2_(ACN)_2_ and **L10** formulated the catalyst, in the presence of which the model reaction produced ester α-**3aa** in 96:4 er and 90% isolated yield (94:5:1 rr and 95% combined NMR yield of all isomers of **3a** **a**; entry 14). If needed, the reaction can also be performed with either an alkene as a limiting reagent or even with stoichiometric amounts of both starting materials, forming the esters with similar regio- and enantioselectivities in 58–64% yields (entries 15–16).

We next examined the scope and limitations of the method with other alcohols and alkenes (Figure 3). We found that the protocol is applicable to a broad range of achiral and chiral alcohols bearing different functional groups. A series of achiral primary, secondary, and tertiary aliphatic, homobenzylic, and (hetero)­benzylic alcohols of diverse sizes, substitution patterns, and varied electronic properties are competent starting materials for the reactions with **1a**, as indicated by the selective formation of **3ab–** **3am** with >96:4 er’s, > 93:7 rr’s, and typically >80% yields. Lower unoptimized yields were obtained for *tert*-butyl ester **3ag** or pentafluorobenzyl ester **3an**, but the regio- and enantioselectivities remained equally high. Strongly coordinating and polar functional groups, such as thiophene, thioether, lactam, and phthalimide-protected amine, are also compatible with the method, as indicated by forming **3ao–3ar** with 87:13–96:4 er’s, 91:9–95:5 rr’s, and 49–95% yields. Notably, the reaction forming **3ar** was similarly selective when conducted on either a 0.25 or a 2.5 mmol scale, in the latter case forming 0.45 g of the product. Lastly, the reaction of alkene **1a** with phenol **2s** formed trace amounts of ester **3as**, indicating the current limitation.

**Figure 3:**
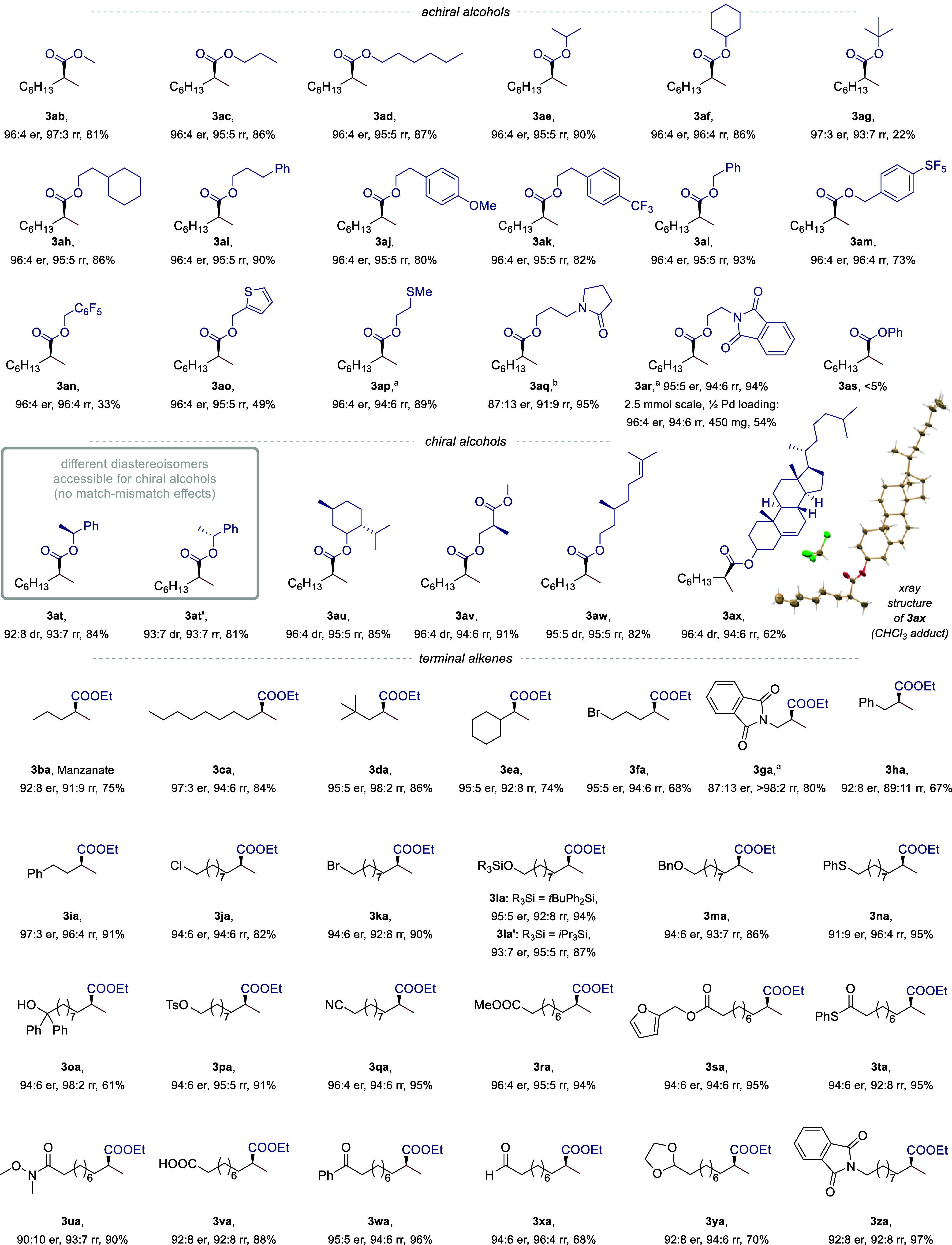
Scope of enantio- and regioselective alkoxycarbonylation of unactivated terminal alkenes. Conditions: 0.25 mmol alkene, 0.50 mmol alcohol, 1–2 mol % PdBr_2_(ACN)_2_, 2–4 mol % **L10**, 20/20 bar CO/H_2_, mtbe or 1,4-dioxane, 40–45 °C, 16 h, er’s and yields of isolated materials; dr’s and rr’s for the reaction mixture; rr’s reflect ratios of α-regioisomer versus the sum of all other regioisomers; some er’s determined upon converting the ester to the alcohol; see the SI for all experimental details. ^a^ 48 h. ^b^ 120 h.

Attractively, in the case of reactions with chiral alcohols, either diastereomer of the product can be prepared with high stereoselectivity, as demonstrated with **3at** and **3at’**. Each diastereomer was formed with >92:8 diastereomeric ratios (dr’s), 93:7 rr’s, and 81–84% yields, indicating the lack of common match–mismatch effects. Further, a series of other synthetically or biologically relevant chiral alcohols, such as menthol **2u**, Roche ester **2v**, citronellol **2w**, and cholesterol **2x,** reacted with **1a** to form **3au–** **3ax** with >93:7 dr’s, > 93:7 rr’s, and 62–91% yields of the α-regioisomers.

We also found that both linear and branched terminal unactivated alkenes of different sizes, bearing substituents in the α-, β-, γ-, and more remote sites of the alkyl chain, are competent starting materials, reacting to form esters **3ba–** **3za** with high enantio- and regioselectivities and moderate to high yields (87:13–97:3 er’s, 89:11–98:2 rr’s, and 61–97% yields). These examples demonstrate the excellent functional-group tolerance, including alkyl halide, silyl and benzyl ether, thioether, bulky tertiary alcohol, tosylate, nitrile, ester, thioester, the Weinreb amide, carboxylic acid, ketone, aldehyde, acetal, and phthalimide. Notably, the method is effective for allylamine derivatives and (homo)­allylbenzenes, which tend to isomerize easily^22^ but here formed α-esters **3ga–3ia** selectively, with high enantioselectivities, regioselectivities, and yields.

While the method development was focused on *unactivated terminal* alkenes, such as 1-octene **1a**, we also explored the applicability of the protocol to other classes of olefins, including 1,2- and 1,1-disubstituted unactivated and activated alkenes (Figure 4). The results demonstrate the distinct reactivity profiles of the different classes of alkenes.

**Figure 4:**
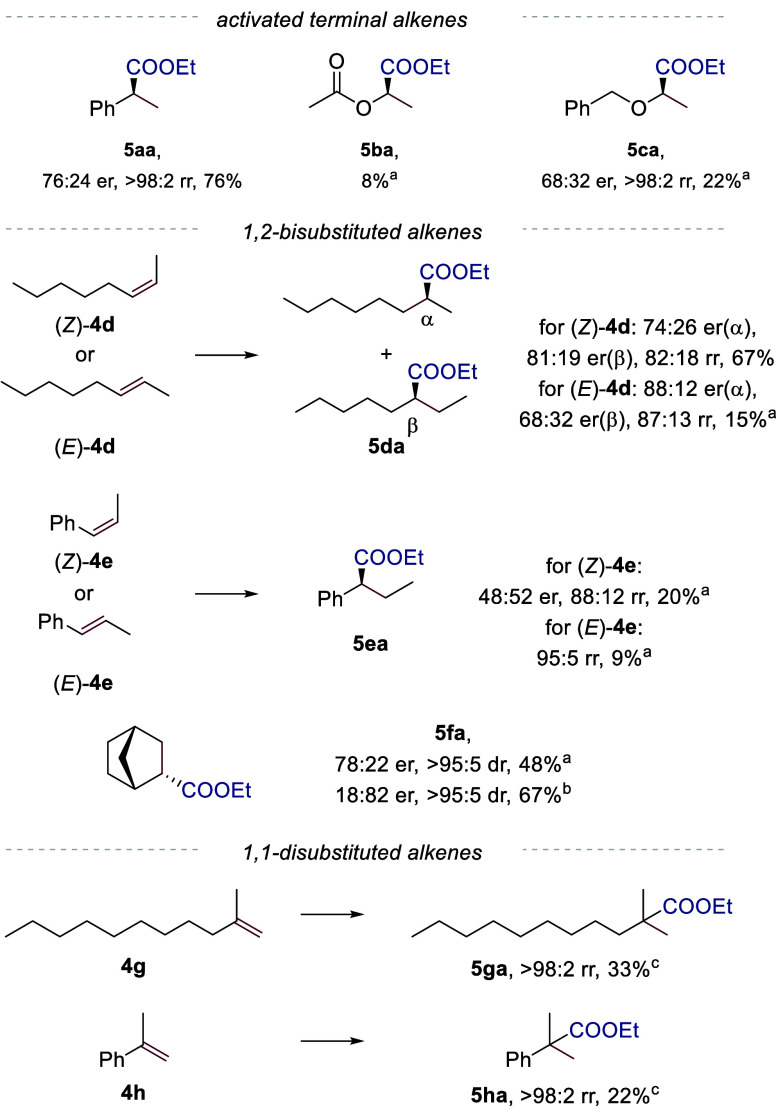
Reactivity of alkenes other than unactivated terminal olefins. Conditions as in Figure 3, 16–48 h; see the SI for all experimental details. ^a^ NMR yield. ^b^ **L5**. ^c^ 4 mol % Pd, **L5**.

Under standard conditions, both cyclic and acyclic unactivated internal alkenes reacted to form the esters, with (*Z*)-olefins reacting with higher conversions than (*E*)-isomers, consistent with established reactivity trends.^23^ Both stereoisomers of 2-octene **4d** converged to the same major regio- and enantiomer (up to 88:12 er, 87:13 rr). Studies with **L5** and **L6**, precursor ligands of ValleyPhos **L10**, established that the bulky 3,5-di-*tert*-butylphenyl and cyclopentyl groups are essential for achieving high enantio- and regioselectivities (Figure S2). Notably, such simultaneous regio- and enantiocontrol in reactions of unsymmetrical internal alkenes—particularly linear, unfunctionalized ones—with transition-metal hydride catalysts has remained a long-standing challenge.^24^

For activated alkenes, styrene **4a** furnished **5aa** with moderate enantioselectivity and yield but excellent regioselectivity (76:24 er, >98:2 rr, 76% yield). In contrast, β-methylstyrene **4e** yielded the nearly racemic ester **5ea**, yet still with high regioselectivity (>88:12 rr), while vinyl acetate **4b** and benzyl vinyl ether **4c** displayed low reactivity, showcasing different reactivity trends between activated and unactivated alkenes.

Unactivated and activated 1,1-disubstituted alkenes (**4g–4h**) furnished esters **5ga–5ha** bearing quaternary carbons. Although yields were modest, these reactions displayed an unusual regioselectivity that violates Keulemans’ rule.^25^ Collectively, these findings establish substrate-dependent reactivity trends and demonstrate the potential of this system to address other long-standing challenges in selective alkene alkoxycarbonylation.

Finally, a series of experiments provided insight into the factors governing the activity, regioselectivity, and stereoselectivity (Figure 5). First, in agreement with the precatalyst activation with H_2_, the model reaction performed in the absence of H_2_ furnished **3aa** in a substantially lower yield than the same reaction in the presence of H_2_ (93:7 er, 94:5:1 rr, and 36% yield versus 96:4 er, 94:5:1 rr, and 95% yield; Figure 5a). However, the comparable regio- and enantioselectivity observed in both experiments indicated that the reactions most likely involved the same catalytically active Pd-hydride species, which, in the absence of H_2_, were formed more slowly, for instance, by β-hydride elimination from an alkoxide.^8c^ Notably, the presence of H_2_ does not promote the potential competitive hydroformylation reaction, presumably due to the mild reaction conditions.^11,12^

**Figure 5:**
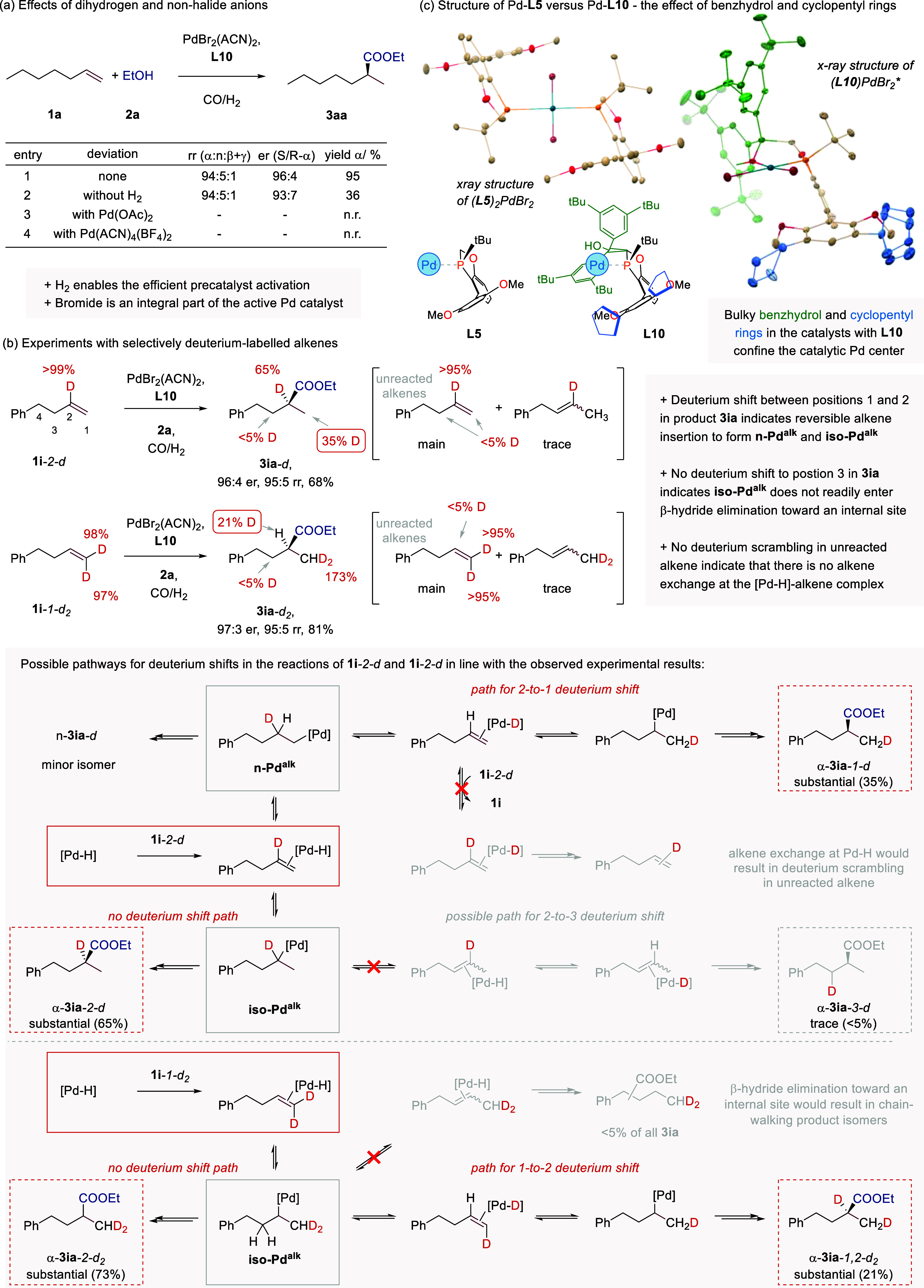
Control and mechanistic experiments. See the SI for experimental details. * The complex co-crystallized with mtbe molecules, which are omitted for clarity.

Second, the reactions with Pd­(OAc)_2_ or Pd­(ACN)_4_BF_4_ in place of PdBr_2_(ACN)_2_, under otherwise standard conditions, did not form detectable amounts of **3aa**, which is consistent with the halide ligand constituting an integral part of the active catalyst.

Third, experiments with the selectively deuterium-labeled alkenes **1i**-*2-d* and **1i**-*1-d*_2_ formed esters **3ia** containing deuterium labeling scrambled between the terminal and subterminal positions of the initial alkene chain (between positions 1 and 2) but without noticeable deuterium incorporation into more internal positions (positions 3 and 4; Figure 5b). In addition, deuterium labeling in the unreacted alkenes **1i**-*2-d* and **1i**-*1-d*_2_ was unchanged, and only trace amounts of isomerized alkenes were formed. Deuterium scrambling between terminal and subterminal positions in the product **3ia** indicates that both catalytic intermediates **n-Pd** ^**alk**^ and **iso-Pd** ^**alk**^ are formed in substantial amounts and that their formation is reversible (through reversible alkene insertion and β-hydride elimination steps; possible pathways forming **3ia**-*1-d* and **3ia**-*2-d* through **n-Pd** ^**alk**^ and **iso-Pd** ^**alk**^ are shown in Figure 5b). The data suggest that the regioselectivity of the reaction is not determined during alkene insertion but later in the cycle.^23^ In turn, no deuterium shift to position 3 in **3ia** indicates that, while **iso-Pd** ^**alk**^ does undergo β-hydride elimination toward the terminal position, it does not readily undergo the reaction toward the internal position, hence suppressing the chain-walking processes. In this case, the selective β-hydride elimination toward the terminal position over the internal position is likely imposed by steric effects promoting the reaction involving the stronger but more accessible primary C­(sp^3^)–H bond over that occurring at the weaker but less accessible secondary C­(sp^3^)–H bond. Besides, given the reversible insertion of an alkene into a Pd–hydride bond, no deuterium scrambling in the remaining alkenes **1i**-*2-d* and **1i**-*1-d*_2_ indicates that free alkenes do not exchange readily with an alkene bound to the Pd-hydride complex. These data, in turn, suggest that alkene coordination, during which one of the prochiral faces of the double bond binds to the metal center, is irreversible and, hence, enantiodetermining.^26^

Lastly, the crystal structures of the precatalysts (**L10**)­PdBr_2_ and (**L5**)_2_PdBr_2_ help visualize both the position of the Pd with respect to the main ligand framework and how the bulky benzhydrol motif (green) and cyclopentyl rings (blue) in **L10** help create a deep chiral pocket around the catalytic Pd center, enabling the challenging enantio-differentiation of the prochiral faces of aliphatic alkenes (Figure 5c). Although further studies are required to elucidate the detailed mechanism of the reaction, these experiments illustrate the importance of the monophosphorus and bromide ligands and suggest the features controlling the regio- and enantioselectivities.

## Conclusions

To summarize, the method established here enables, for the first time, highly regio- and enantioselective alkoxycarbonylation of unactivated terminal alkenes, providing easy access to valuable enantioenriched chiral aliphatic esters from various readily available starting materials. Furthermore, the catalyst showed unique selectivity profiles with 1,1- and 1,2-disubstituted alkenes, creating an entry point to previously inaccessible alkoxycarbonylative reactions. The successful development of the method rests on the formulation of a palladium-bromide complex with a new chiral valley-shaped monodentate ligand. The control experiments and preliminary mechanistic studies illustrated the importance of both chiral phosphorus and bromide ligands on the activity, regioselectivity, and enantioselectivity of the reaction and provided an insight into the alkene hydrometalation step, which is central to other Pd-catalyzed transformations of alkenes.^3a–3f^ Therefore, besides providing a new, valuable method for the synthesis of fine chemicals containing chiral ester motifs, this work is expected to support further research on the selective functionalization of unactivated alkenes, including the extension of our methodology to the synthesis of other highly valuable chiral carboxylic acid derivatives of widespread use in fine chemistry.

## Supplementary Material



## Notes

Details on experimental procedures, including detailed catalytic protocols, optimization and control experiments, analytical data, and copies of the NMR spectra and GC traces (PDF)
