# Supplementary material for: Regio- and Enantioselective Alkoxycarbonylation of Unactivated Terminal Alkenes under Palladium-Bromide-Monophosphine Catalysis
Source: J Am Chem Soc. 2026 Jun 18;148(25):26960–9. doi: 10.1021/jacs.6c10237 (PMC13339162; doi:10.1021/jacs.6c10237)
Supplement: Supplementary file 1 [file ja6c10237_si_001.pdf]

Supporting Information for

Regio- and Enantioselective Alkoxycarbonylation of Unactivated Terminal Alkenes under Palladium-Bromide-Monophosphine Catalysis

Michel Sigrist,[‡,1,2] Kuhali Das,[‡,1,2] Gracjan Kurpik,[‡,1] Wei Tian,[3,4] Jianxun Huang,[3] Ala Covas,[2] Andrew D. Bond,[1] Wenjun Tang,[3,4*] and Paweł Dydio[1,2*]

[1] Yusuf Hamied Department of Chemistry, University of Cambridge, Cambridge CB2 1EW, United Kingdom

[2] University of Strasbourg, CNRS, ISIS UMR 7006, 67000 Strasbourg, France

[3] State Key Laboratory of Bio-Organic and Natural Products Chemistry, Center for Excellence in Molecular Synthesis, Shanghai Institute of Organic Chemistry, University of Chinese Academy of Sciences, Shanghai 200032, China

[4] School of Chemistry and Material Science, Hangzhou Institute for Advanced Study, University of Chinese Academy of Sciences, Hangzhou 310024, China

‡These authors contributed equally.

*E-mails: pd552@cam.ac.uk, tangwenjun@sioc.ac.cn

Table of content

1. Supplementary methods S2

2. Synthesis of $PdBr_2(ACN)_2$ S3

3. Synthesis of ligands S4

4. Evaluation of chiral monophosphorus ligands and palladium halide precursors S34

5. General procedure for the palladium-catalysed α-branched regioselective and enantioselective carbonylation of unactivated alkenes S35

6. Characterisation of products S39

7. Synthesis and characterisation of starting materials S104

8. Mechanistic studies S109

   a. Synthesis of deuterium-containing alkenes S109

   b. Deuterium-labelled experiments S111

9. Copies of NMR spectra S118

10. Single-crystal X-ray diffraction S187

11. References S190

1. Supplementary methods

Unless otherwise stated, all experiments were conducted on the laboratory bench or in a well-ventilated fume hood in air with reagent-grade solvents. Reactions under an inert gas atmosphere were carried out in oven-dried glassware in a nitrogen-filled glovebox or by standard Schlenk techniques under nitrogen. Unless otherwise noted, all reagents and solvents were purchased from commercial suppliers and used without further purification. For experiments conducted under an inert gas atmosphere, dried and degassed solvents were either purchased from commercial suppliers and used without further purification or dried and degassed via standard distillation procedures under an inert gas atmosphere. Column chromatography was performed with the aid of a CombiFlash EZ Prep Chromatography System equipped with an integrated ELSD, using RediSep Rf (Gold) Silica Gel Disposable Flash columns, or manually using Merck Kieselgel 60 (230-400 mesh). NMR spectra were recorded on the Bruker 400 MHz, 500 MHz or 700 MHz Brucker spectrometers at the facilities of the Yusuf Hamied Department of Chemistry, University of Cambridge, or the Institute of Science and Supramolecular Engineering, the University of Strasbourg and CNRS. NMR spectra were processed using the MestReNova x64 software. Chemical shifts are reported in parts per million (ppm) and referenced to residual solvent peaks. Coupling constants (*J*) are reported in hertz (Hz). GC-FID analysis was performed using a Shimadzu GC-2010 Plus instrument equipped with an SH-I-5MS column (30 m x 0.25 mm ID x 0.25 µm df) and connected to an FID detector. GC-MS analysis was obtained on a Shimadzu QP2020 (EI) instrument equipped with an SH-I-5MS column (30 m x 0.25 mm ID x 0.25 µm df). NMR yields were calculated using mesitylene as the internal standard. GC-FID chiral analyses were obtained on a Shimadzu GC-2010 Plus instrument equipped with a Cyclosil-B column (30 m x 0.25 mm ID x 0.25 um film) or CP-Chirasil-Dex CB column (25 m x 0.25 mm ID x 0.25 um film) for chiral analysis, and an FID detector. SFC analysis was performed on an Agilent SFC 1260 Infinity II instrument coupled to an Agilent MSD XT mass spectrometer. Chiral SFC separations were carried out using DAICEL CHIRALPAK columns (100 mm × 3 mm ID, 3 µm) with supercritical CO_2 and appropriate organic modifiers, with conditions specified for each individual separation. Electrospray-ionisation quadrupole-time-of-flight high-resolution mass spectrometric (ESI-QTOFHRMS) experiments were performed with a Synapt G3-S HDMS, Waters Co., Milford, MA, USA, at the Yusuf Hamied Department of Chemistry, University of Cambridge. Optical rotations were measured in spectrophotometric grade $CHCl_3$ on a Perkin Elmer 343 Polarimeter or an Anton Paar MCP 100 Polarimeter equipped with an Anton Paar stainless steel cuvette (100 mm, CL. 0.01, ø 5 mm), using a sodium lamp (λ = 589 nm, D-line). $[\alpha]_D$ values are reported at a specified temperature in degrees mL g^{-1} dm^{-1} with concentration (c) in cg mL^{-1}.

2. Synthesis of $PdBr_2(ACN)_2$

$$PdBr_2 \xrightarrow[\text{2 hours}]{\text{MeCN, 80 °C,}} PdBr_2(MeCN)_2$$

The material was prepared following a literature procedure: [1] To a 250 mL round-bottom flask equipped with a magnetic stirring bar, palladium bromide (1.06 g, 4 mmol) and acetonitrile (80 mL) were added. The mixture was heated at 80 °C until a clear red-orange mixture was obtained (~2 h). Then, the reaction mixture was cooled to room temperature. The mixture was concentrated under reduced pressure to a volume of approximately 5 mL and triturated with diethyl ether (15 mL). The solid material was filtered, washed with diethyl ether (2 x 15 mL), and dried under vacuum, affording the title compound as a red-orange solid (1.33 g, 95%).

3. Synthesis of ligands

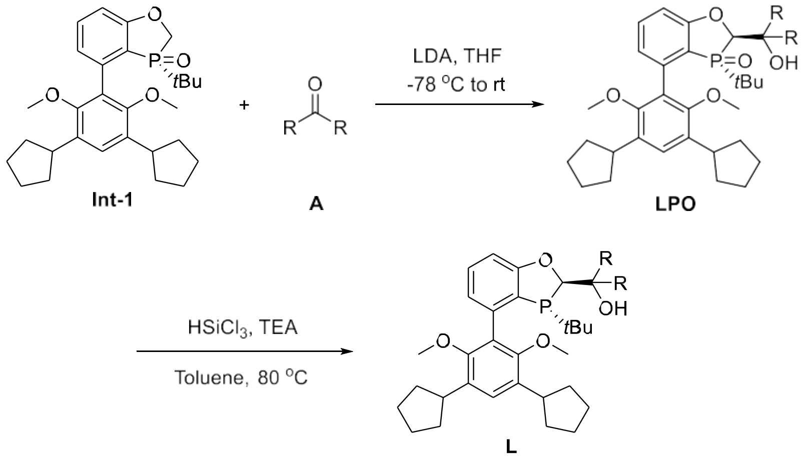

A 25 mL flame-dried Schlenk tube was charged with **Int-1** (0.5 mmol, 1.0 equiv), dissolved with 2 mL anhydrous THF, then a solution of LDA in hexane (2.0 M, 2 mmol, 4 equiv) was added dropwise at -78 °C under nitrogen. The reaction mixture was stirred for 1 h at -78 °C before the addition of **A** (4 mmol, 8 equiv) at the same temperature. The reaction system was warmed to rt within 2 h and stirred for 8 h at rt. Upon completion, the reaction was quenched by the addition of H_2O (6 mL) and the resulting mixture was extracted with EA (10 mL×3). The organic phases were combined, dried over Na_2SO_4, filtered and concentrated under reduced pressure. The crude product was purified by column chromatography (eluent: PE/EA = 5/1) to give the product **LPO**.

At 0 °C, to a solution of **LPO** (0.4 mmol, 1 equiv) in anhydrous toluene (2.0 mL) was added triethylamine (6.4 mmol, 16 equiv), followed by $HSiCl_3$ (3.2 mmol, 8 equiv). The mixture was warmed to 80 °C and stirred for 12 h under nitrogen. Upon completion, the reaction system was cooled to 0 °C and a solution of degassed 30% NaOH (aq) was added dropwise under nitrogen until the reaction system stabilized. The resulting mixture was allowed to warm to 70 °C and stirred until the two phases separated clearly (around 0.5 h). The organic layer was separated and the aqueous phase was extracted with EA (10 mL×3). The organic phases were combined, dried over Na_2SO_4, filtered and concentrated under reduced pressure. The crude product was purified by flash column chromatography (eluent: hexane, then PE/EA = 10:1) to afford the final product **L**.

Int-1 was synthesized according to the reported literature.[2]

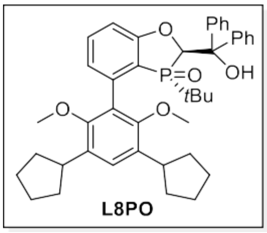

L8PO (colourless oil, 58% yield), $[\alpha]_D^{25}$ = -304.5 (c = 0.20, $CHCl_3$).

^{1}H NMR (600 MHz, $CDCl_3$) δ 7.64 (d, J = 7.2 Hz, 2H), 7.55 (d, J = 7.2 Hz, 2H), 7.42 (t, J = 7.9 Hz, 1H), 7.31 (t, *J* = 7.8 Hz, 2H), 7.22-7.17 (m, 3H), 7.11-7.09 (m, 2H), 6.93 (dd, J = 8.4, 3.3 Hz, 1H), 6.88 (dd, J = 7.4, 3.6 Hz, 1H), 6.01 (s, 1H), 5.33 (d, *J* = 2.2 Hz, 1H), 3.50 (s, 3H), 3.27 (tt, *J* = 9.8, 7.5 Hz, 1H), 3.19 (tt, *J* = 9.9, 7.5 Hz, 1H), 3.13 (s, 3H), 2.05-1.99 (m, 2H), 1.90-1.76 (m, 4H), 1.69-1.56 (m, 8H), 1.49-1.44 (m, 1H), 1.33-1.30 (m, 1H), 0.93 (d, *J* = 16.5 Hz, 9H).

^{13}C NMR (151 MHz, $CDCl_3$) δ 164.8 (d, J = 18.7 Hz), 154.5, 153.7, 145.7 (d, J = 4.8 Hz), 143.0 (d, J = 3.5 Hz), 139.5 (d, J = 5.7 Hz), 135.5, 134.2, 133.6, 128.1, 127.5, 127.2, 127.2, 127.1, 126.6, 126.4, 125.5, 125.1 (d, J = 8.6 Hz), 114.7, 114.1, 112.9 (d, J = 5.4 Hz), 80.3, 62.0, 61.3, 38.7, 38.3, 35.4, 34.9, 34.5, 34.4, 34.2, 34.0, 25.8, 25.7, 25.6 (d, J = 2.9 Hz), 23.7.

^{31}P NMR (162 MHz, $CDCl_3$) δ 69.1.

ESI-MS: m/z 665.50 $[M+H]^+$, 687.40 $[M+Na]^+$. HRMS (ESI) calculated for $[M+H, C_{42}H_{50}O_5P]^+$: 665.3390; found: 665.3390; $[M+Na, C_{42}H_{49}NaO_5P]^+$: 687.3210; found: 687.3212.

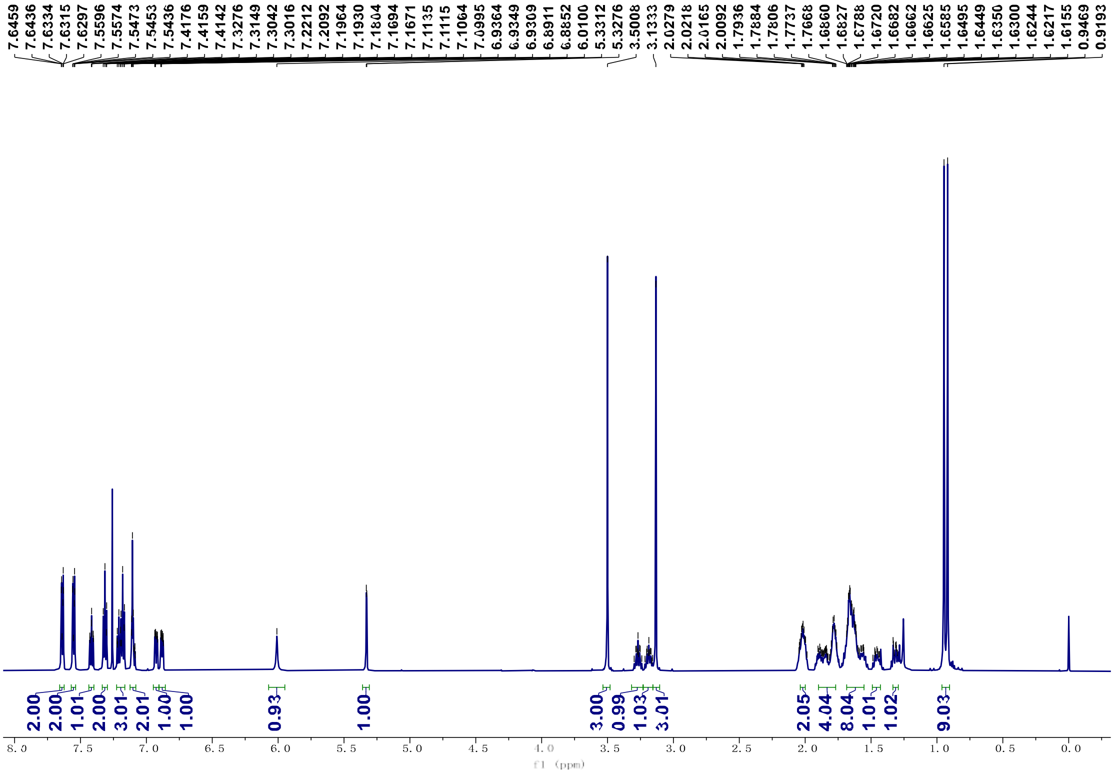

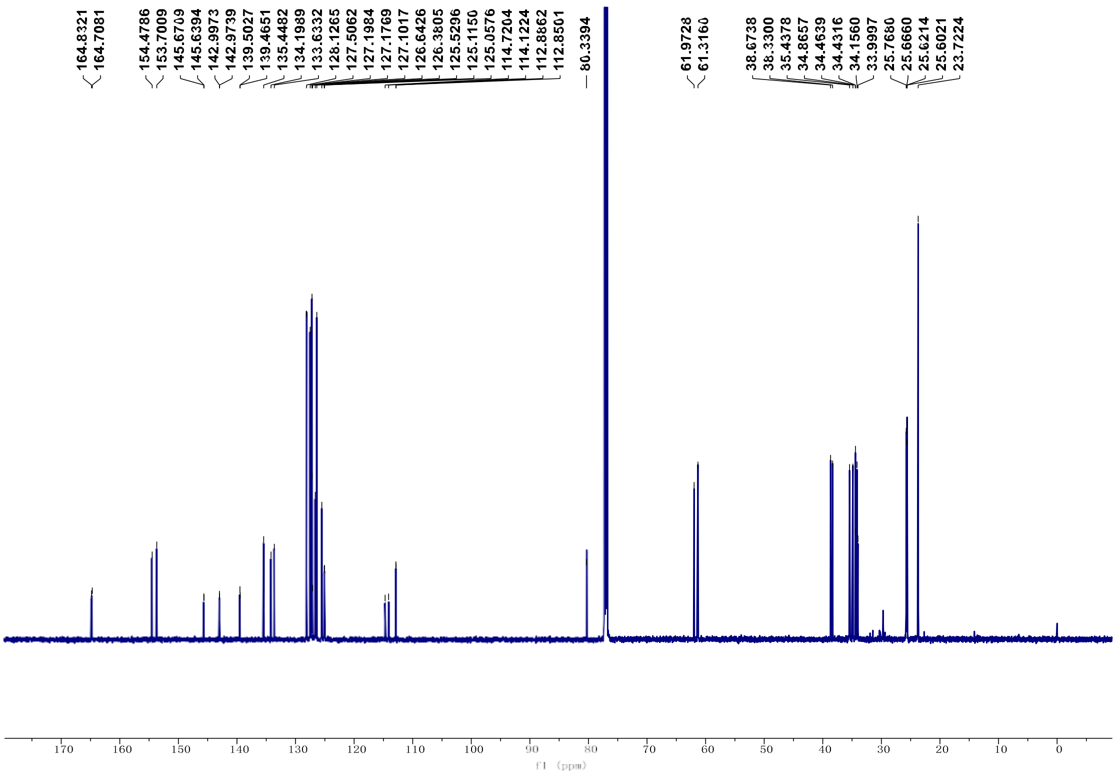
164.8321
164.7081
154.4786
153.7009
145.6709
145.6394
142.9973
142.9739
139.5027
139.4651
135.4482
134.1989
133.6332
128.1265
127.5062
127.1984
127.1769
127.1017
126.6426
126.3805
125.5296
125.1150
125.0576
114.7204
114.1224
112.8862
112.8501
80.3394
61.9728
61.3160
38.6738
38.3300
35.4378
34.8657
34.4639
34.4316
34.1560
33.9997
25.7680
25.6660
25.6214
25.6021
23.7224
f1 (ppm)

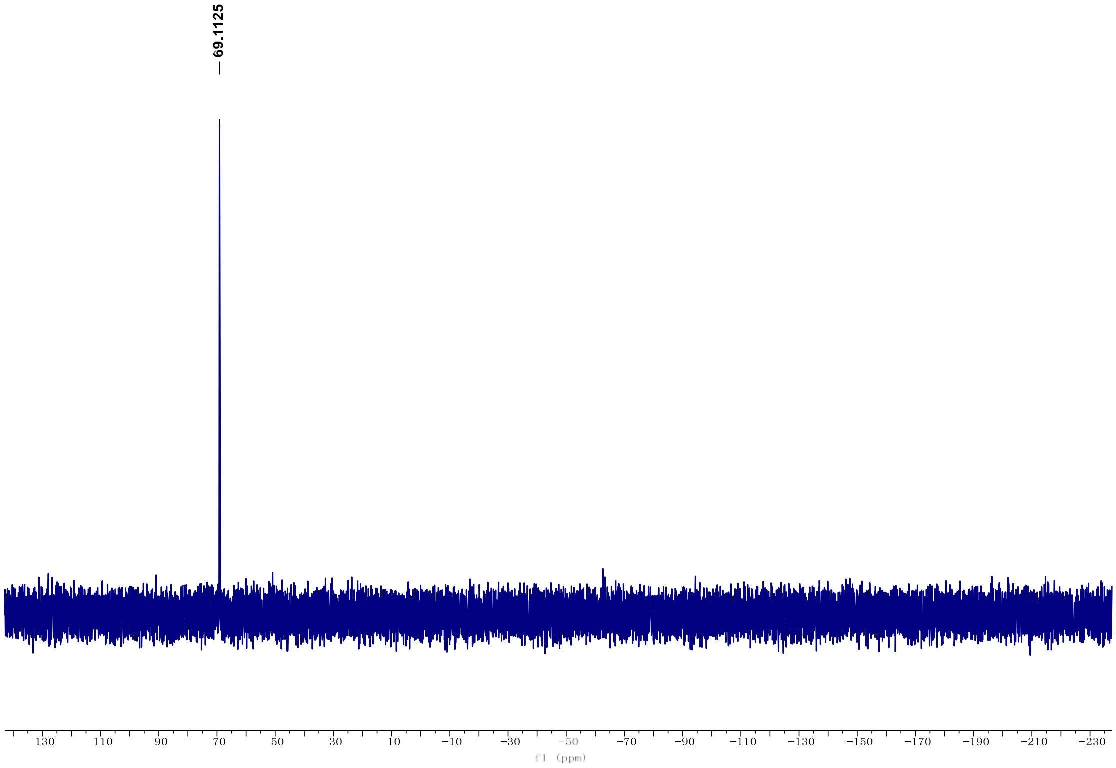
69.1125
f1 (ppm)

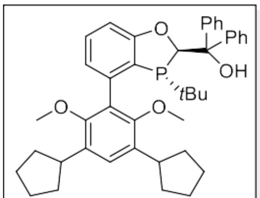
Ph
Ph
OH
O
P
tBu
O
O

L8

L8 (white solid, 80% yield), $[\alpha]_D^{25}$ = -34.1 (c = 0.50, $CHCl_3$).

^{1}H NMR (600 MHz, $CDCl_3$) δ 7.63 (d, J = 6.7 Hz, 2H), 7.44 (d, J = 6.9 Hz, 2H), 7.32 (t, *J* = 7.7 Hz, 2H), 7.29-7.25 (m, 3H), 7.22 (t, *J* = 7.4 Hz, 1H), 7.17 (t, *J* = 7.4 Hz, 1H), 7.09 (s, 1H), 6.96 (dd, *J* = 7.4, 3.4 Hz, 1H), 6.84 (d, *J* = 8.1 Hz, 1H), 5.70 (s, 1H), 3.73 (s, 3H), 3.30-3.21 (m, 5H), 3.14 (d, *J* = 3.7 Hz, 1H), 2.13-1.66 (m, 14H), 1.50-1.45 (m, 1H), 1.34-1.30 (m, 1H), 0.63 (d, *J* = 12.3 Hz, 9H).

^{13}C NMR (151 MHz, $CDCl_3$) δ 165.2, 154.2 (d, J = 25.6 Hz), 144.6 (d, J = 3.3 Hz), 144.2 (d, J = 3.3 Hz), 139.4 (d, J = 18.0 Hz), 135.3, 134.6, 130.3, 129.3, 127.9 (d, J = 10.4 Hz), 127.2, 126.8 (d, J = 5.8 Hz), 126.6 (d, J = 3.0 Hz), 124.8 (d, J = 15.1 Hz), 124.7, 122.6 (d, J = 3.9 Hz), 109.4, 89.4 (d, J = 29.7 Hz), 82.1 (d, J = 17.4 Hz), 62.9, 61.0, 39.6, 38.6, 35.5, 34.8, 34.2, 33.3, 30.7 (d, J = 20.1 Hz), 26.8 (d, J = 15.0 Hz), 25.7 (d, J = 5.6 Hz), 25.6 (d, J = 6.2 Hz).

^{31}P NMR (162 MHz, $CDCl_3$) δ -5.2.

ESI-MS: m/z 671.55 $[M+Na]^+$; HRMS (ESI) calculated for $[M+H, C_{42}H_{50}O_4P]^+$: 649.3441; found: 649.3447; $[M+Na, C_{42}H_{49}NaO_5P]^+$: 671.3261; found: 671.3256.

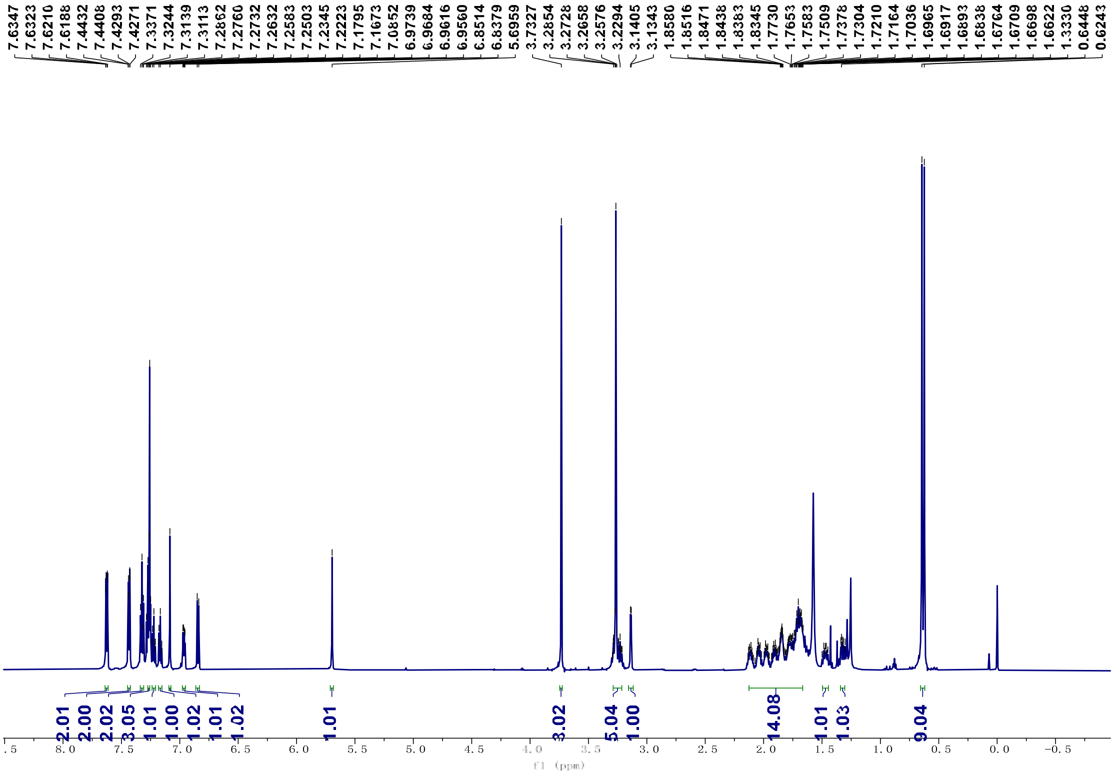

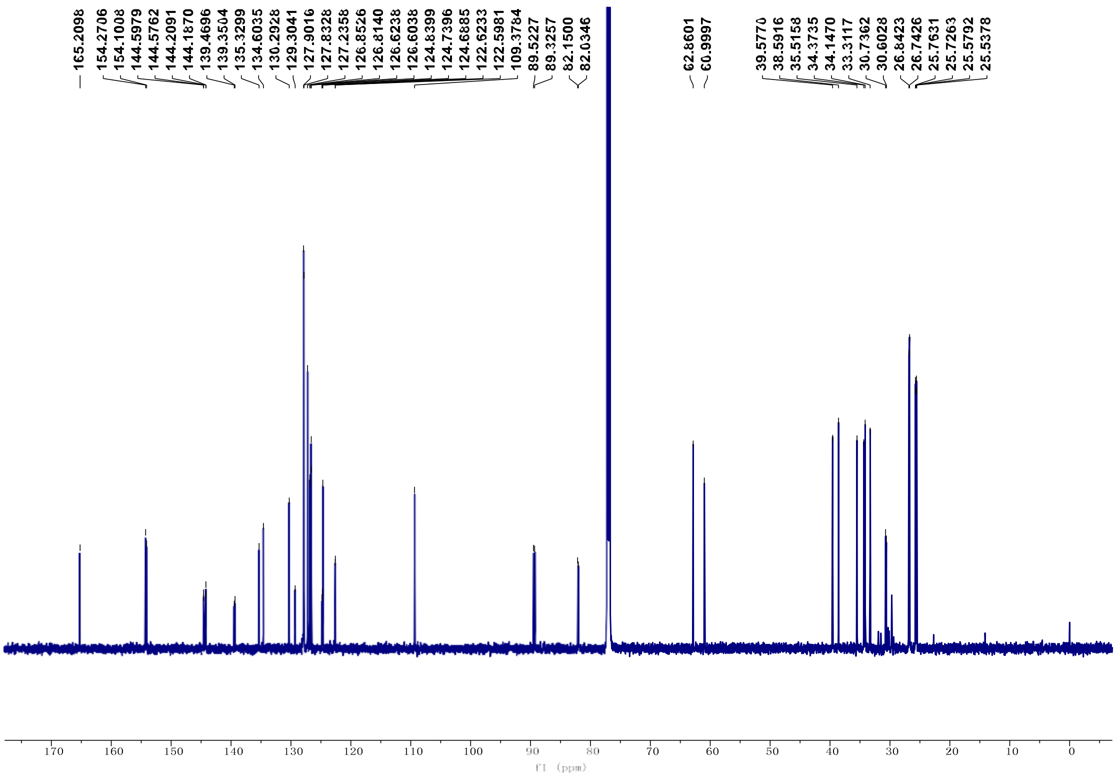
165.2098
154.2706
154.1008
144.5979
144.5762
144.2091
144.1870
139.4696
139.3504
135.3299
134.6035
130.2928
129.3041
127.9016
127.8328
127.2358
126.8526
126.8140
126.6238
126.6038
124.8399
124.7396
124.6885
122.6233
122.5981
109.3784
89.5227
89.3257
82.1500
82.0346
62.8601
60.9997
39.5770
38.5916
35.5158
34.3735
34.1470
33.3117
30.7362
30.6028
26.8423
26.7426
25.7631
25.7263
25.5792
25.5378
f1 (ppm)

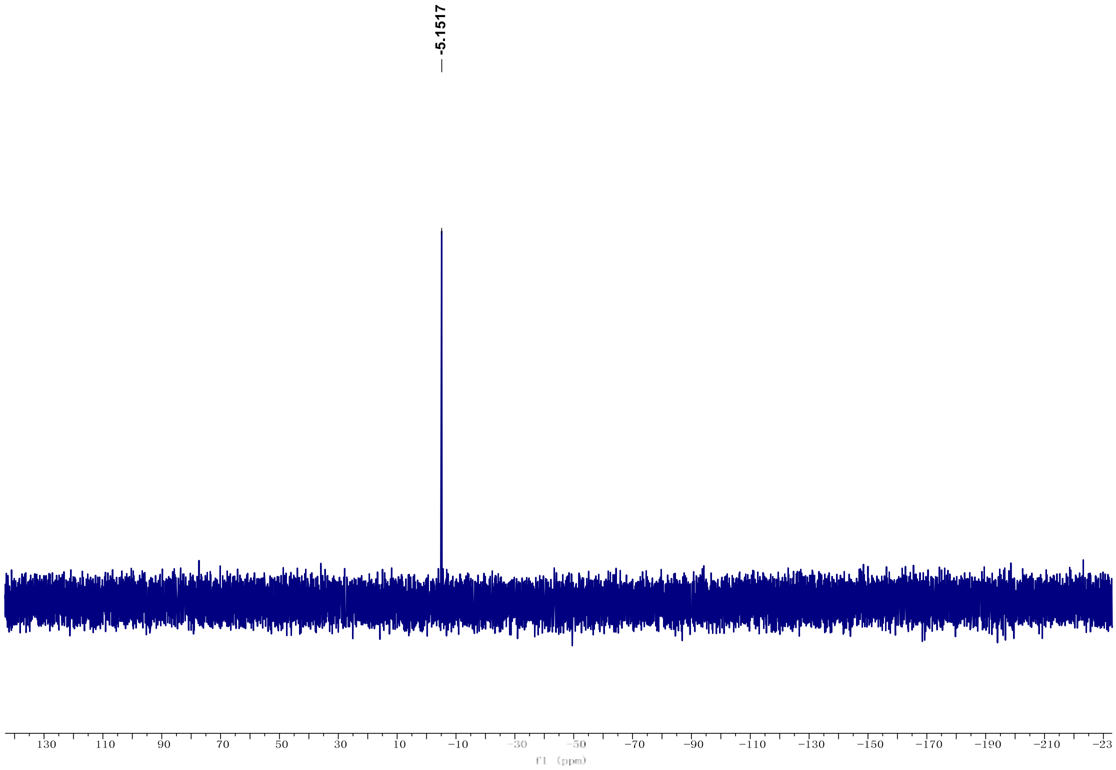
-5.1517
f1 (ppm)

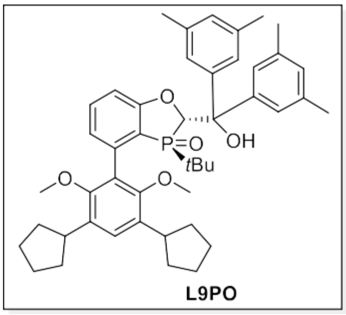

L9PO (white solid, 65% yield), $[\alpha]_D^{25}$ = 269.8 (c = 0.23, $CHCl_3$).

^{1}H NMR (600 MHz, $CDCl_3$) δ 7.43 (t, J = 7.9 Hz, 1H), 7.24 (s, 2H), 7.14 (s 2H), 7.09 (s, 1H), 6.97 (dd, J = 8.4, 3.3 Hz, 1H), 6.87-6.85 (m, 2H), 6.70 (s, 1H), 5.74 (s, 1H), 5.31 (d, *J* = 2.2 Hz, 1H), 3.47 (s, 3H), 3.27-3.16 (m, 2H), 3.04 (s, 3H), 2.30 (s, 6H), 2.18 (s, 6H), 2.04-1.99 (m, 3H), 1.91-1.75 (m, 5H), 1.65-1.55 (m, 6H), 1.47-1.42 (m, 1H), 1.33-1.29 (m, 1H), 1.00 (d, *J* = 16.5 Hz, 9H).

^{13}C NMR (151 MHz, $CDCl_3$) δ 165.0 (d, J = 19.3 Hz), 154.4, 153.6, 145.9 (d, J = 5.5 Hz), 142.4 (d, J = 3.1 Hz), 139.6 (d, J = 5.6 Hz), 137.4, 136.5, 135.4, 134.1, 133.4, 128.9, 128.2, 127.1, 125.4, 125.1 (d, J = 8.6 Hz), 125.0, 124.0, 114.9, 114.3, 112.8 (d, J = 5.4 Hz), 79.7, 77.7, 61.8, 61.1, 38.7, 38.3, 35.4, 34.9, 34.6, 34.5, 34.1, 34.0, 25.7 (d, J = 19.5 Hz), 25.6, 23.8, 21.6 (d, J = 22.9 Hz).

^{31}P NMR (162 MHz, $CDCl_3$) δ 68.9.

ESI-MS: m/z 743.45 $[M+Na]^+$; HRMS (ESI) calculated for $[M+H, C_{46}H_{58}O_5P]^+$: 721.4016; found: 721.4018; $[M+Na, C_{46}H_{57}NaO_5P]^+$: 743.3836; found: 743.3838.

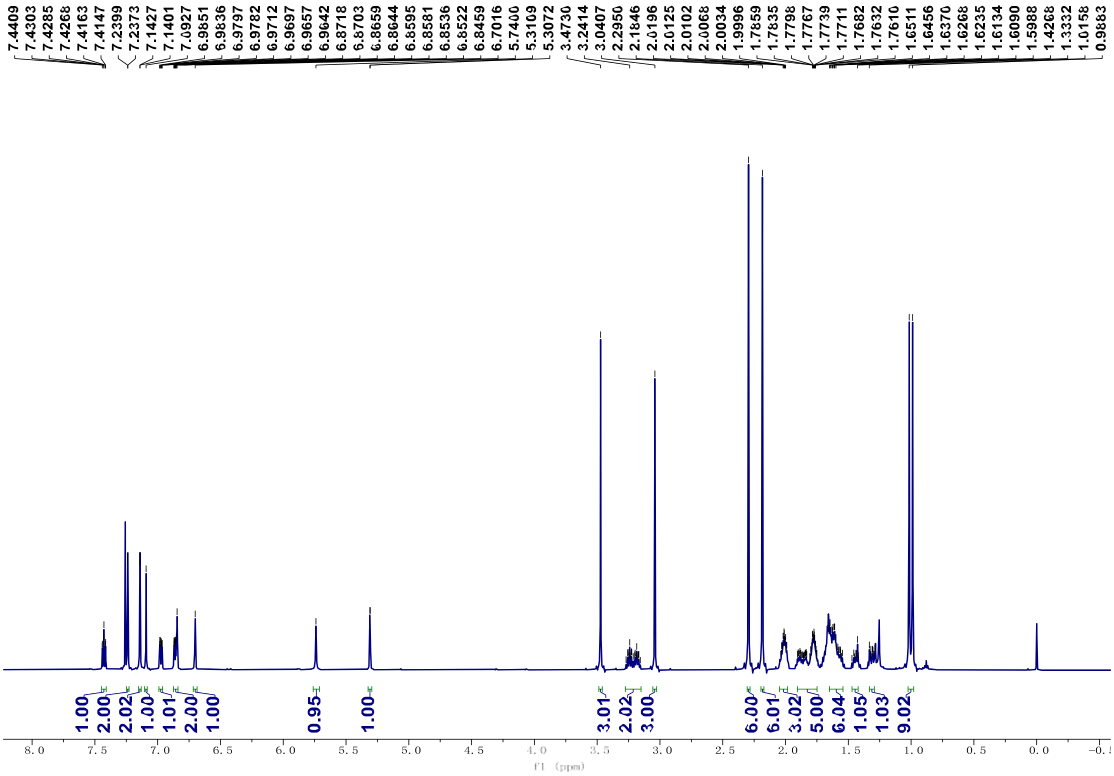

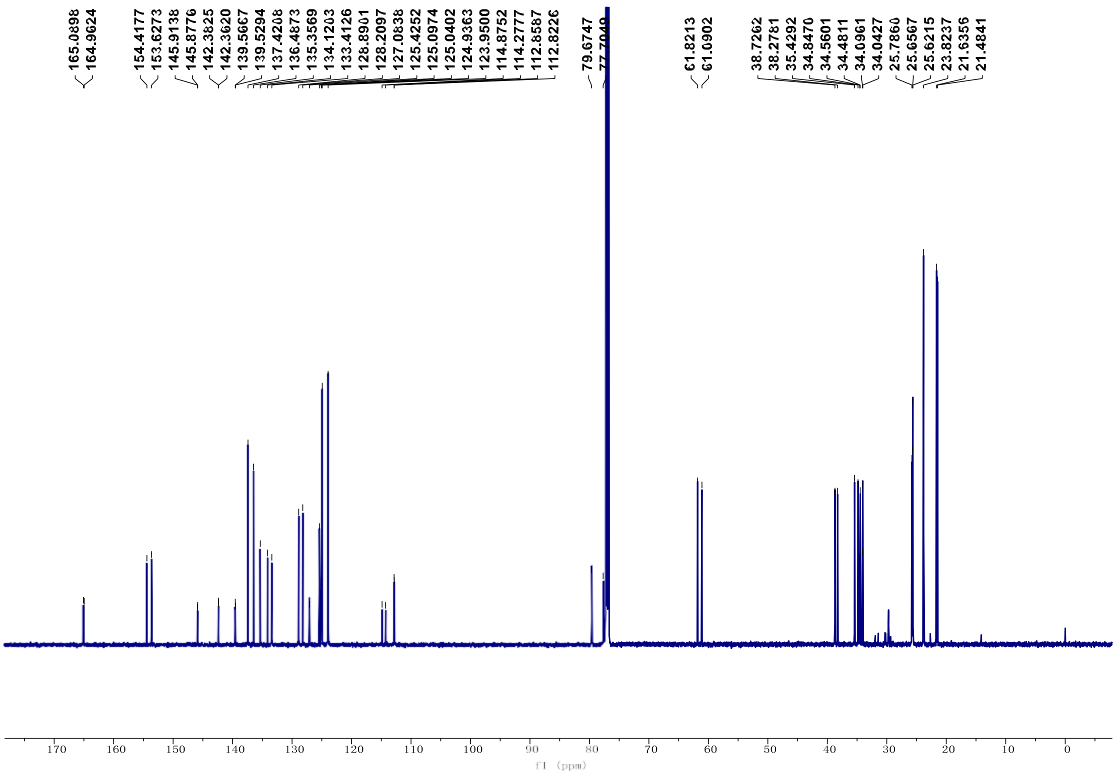

165.0898
164.9624
154.4177
153.6273
145.9138
145.8776
142.3825
142.3620
139.5667
139.5294
137.4208
136.4873
135.3569
134.1203
133.4126
128.8901
128.2097
127.0838
125.4252
125.0974
125.0402
124.9363
123.9500
114.8752
114.2777
112.8587
112.8226
79.6747
77.7049
61.8213
61.0902
38.7262
38.2781
35.4292
34.8470
34.5601
34.4811
34.0961
34.0427
25.7860
25.6567
25.6215
23.8237
21.6356
21.4841
170
160
150
140
130
120
110
100
90
80
70
60
50
40
30
20
10
0
f1 (ppm)

Luo

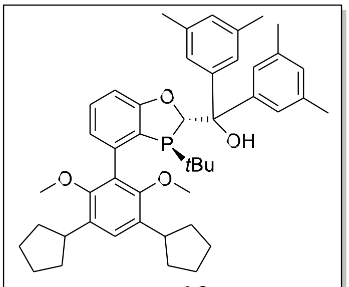

L9

L9 (white solid, 48% yield) $[\alpha]_D^{25}$ = 65.7 (c = 0.17, $CHCl_3$).

^{1}H NMR (600 MHz, $CDCl_3$) δ 7.26 (t, *J* = 7.8 Hz, 1H), 7.22 (d, *J* = 1.6 Hz, 2H), 7.08 (s, 1H), 7.04 (s, 2H), 6.94 (dd, *J* = 7.4, 3.4 Hz, 1H), 6.86-6.84 (m, 2H), 6.79 (s, 1H), 5.68 (s, 1H), 3.74 (s, 3H), 3.26-3.21 (m, 5H), 2.98 (d, *J* = 3.8 Hz, 1H), 2.31 (s, 6H), 2.26 (s, 6H), 2.09-2.03 (m, 2H), 1.99-1.74 (m, 8H), 1.70-1.66 (m, 4H), 1.48-1.44 (m, 1H), 1.33-1.30 (m, 1H), 0.66 (d, *J* = 12.2 Hz, 9H).

^{13}C NMR (151 MHz, $CDCl_3$) δ 165.4, 154.2 (d, *J* = 16.8 Hz), 144.8 (d, *J* = 3.6 Hz), 144.0 (d, *J* = 3.3 Hz), 139.3 (d, *J* = 18.0 Hz), 137.1 (d, *J* = 23.0 Hz), 135.1, 134.5, 130.1, 129.4, 128.4 (d, *J* = 19.5 Hz), 124.9, 124.8, 124.5, 124.1 (d, *J* = 2.7 Hz), 122.5 (d, *J* = 3.6 Hz), 109.4, 89.6 (d, *J* = 29.4 Hz), 81.8 (d, *J* = 17.9 Hz), 62.9, 60.9, 39.6, 38.6, 35.5, 34.2 (d, *J* = 16.6 Hz), 33.2, 30.9, 30.8, 27.3 (d, *J* = 15.0 Hz), 26.9 (d, *J* = 14.6 Hz), 25.7, 25.6, 25.6 (d, *J* = 3.0 Hz), 21.6 (d, *J* = 18.0 Hz), 21.44.

^{31}P NMR (162 MHz, $CDCl_3$) δ -4.8.

ESI-MS: m/z 705.50 $[M+H]^+$, 727.40 $[M+Na]^+$; HRMS (ESI) calculated for [M+H, $C_{46}H_{58}O_4P]^+$: 705.4067; found: 705.4070; [M+Na, $C_{46}H_{57}NaO_4P]^+$: 727.3887; found: 727.3890.

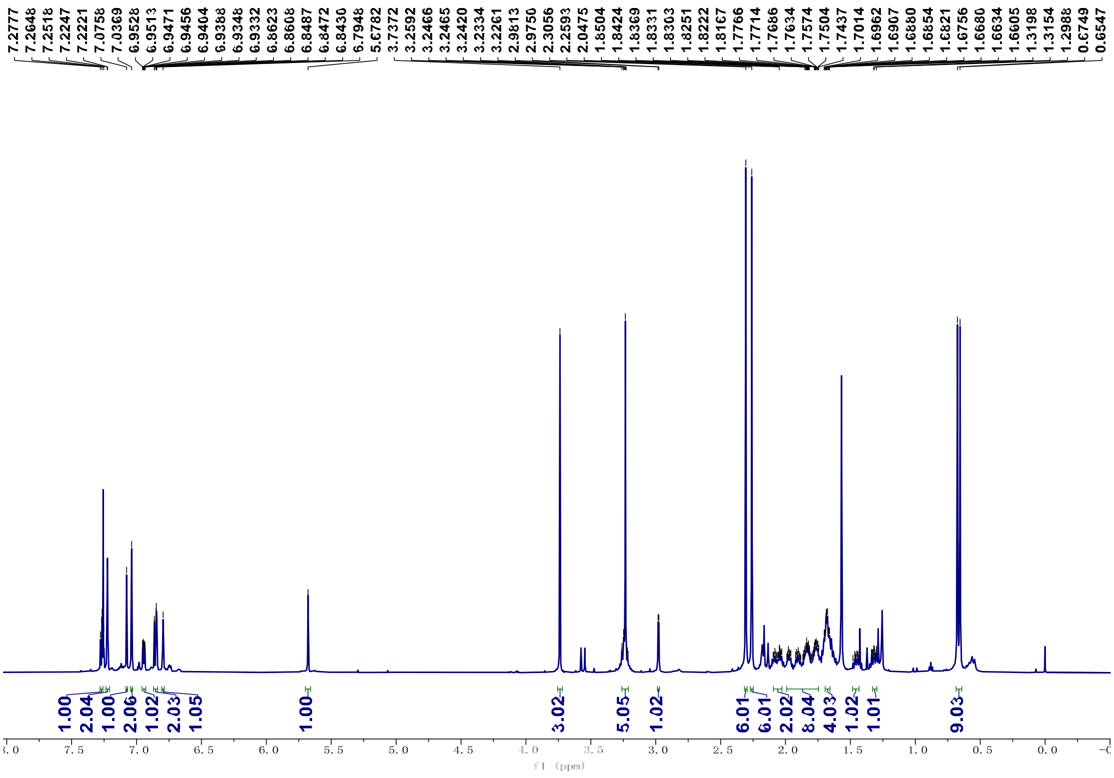

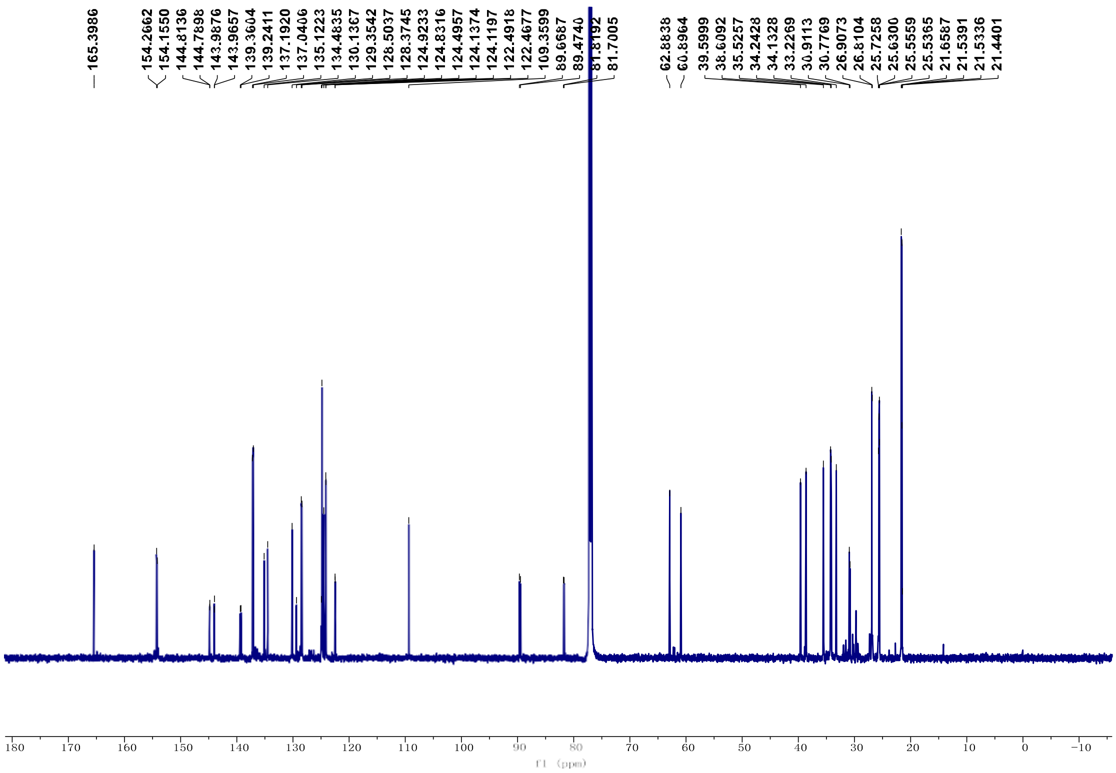
165.3986
154.2662
154.1550
144.8136
144.7898
143.9876
143.9657
139.3604
139.2411
137.1920
137.0406
135.1223
134.4835
130.1367
129.3542
128.5037
128.3745
124.9233
124.8316
124.4957
124.1374
124.1197
122.4918
122.4677
109.3599
89.6687
89.4740
81.8192
81.7005
62.8838
60.8964
39.5999
38.6092
35.5257
34.2428
34.1328
33.2269
30.9113
30.7769
26.9073
26.8104
25.7258
25.6300
25.5559
25.5365
21.6587
21.5391
21.5336
21.4401
f1 (ppm)

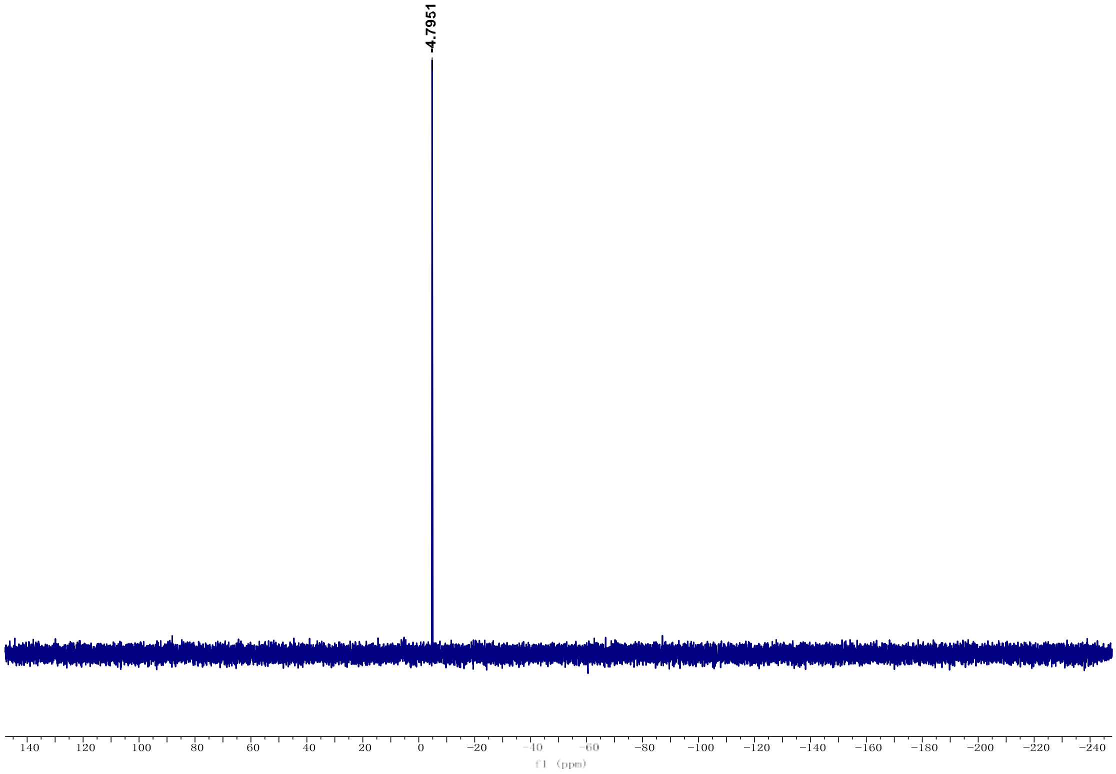
-4.7951
f1 (ppm)

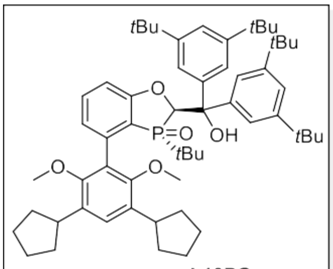

L10PO

L10PO (white solid, 75% yield), $[\alpha]_D^{25}$ = -87.7 (c = 0.17, $CHCl_3$).

^{1}H NMR (600 MHz, $CDCl_3$) δ 7.58 (d, *J* = 1.8 Hz, 2H), 7.48-7.45 (m, 3H), 7.29 (t, *J* = 1.8 Hz, 1H), 7.17 (t, *J* = 1.8 Hz, 1H), 7.07 (s, 1H), 7.04 (dd, *J* = 8.4, 3.3 Hz, 1H), 6.87 (dd, *J* = 7.4, 3.5 Hz 1H), 5.67 (s, 1H), 5.15 (d, *J* = 1.8 Hz, 1H), 3.51 (s, 3H), 3.20-3.13 (m, 2H), 2.83 (s, 3H), 2.01-1.94 (m, 3H), 1.91-1.59 (m, 10H), 1.46-1.40 (m, 1H), 1.30 (s, 18H), 1.29-1.25 (m, 2H), 1.20 (s, 18H), 0.99 (d, *J* = 16.3 Hz, 9H).

^{13}C NMR (151 MHz, $CDCl_3$) δ 164.6 (d, *J* = 19.5 Hz), 154.7, 153.5, 149.8, 149.2, 145.0 (d, *J* = 6.1 Hz), 141.9, 140.0 (d, *J* = 5.6 Hz), 135.3, 134.0, 133.4, 127.1, 125.5, 125.0 (d, *J* = 8.2 Hz), 121.2, 121.0, 120.7, 120.6, 114.9, 114.3, 113.0 (d, *J* = 5.5 Hz), 79.1 (d, *J* = 36.0 Hz), 78.6, 62.0, 61.1, 38.7, 38.3, 35.5, 35.0, 34.9, 34.8, 34.7, 34.4 (d, *J* = 2.9 Hz), 34.3, 31.5 (d, *J* = 6.3 Hz), 25.7, 25.6, 25.6 (d, *J* = 3.5 Hz), 23.8.

^{31}P NMR (162 MHz, $CDCl_3$) δ 67.3.

ESI-MS: m/z 911.60 $[M+Na]^+$; HRMS (ESI) calculated for $[M+H, C_{58}H_{82}O_5P]^+$: 889.5894; found: 889.5893; $[M+Na, C_{58}H_{81}NaO_5P]^+$: 911.5714; found: 911.5714.

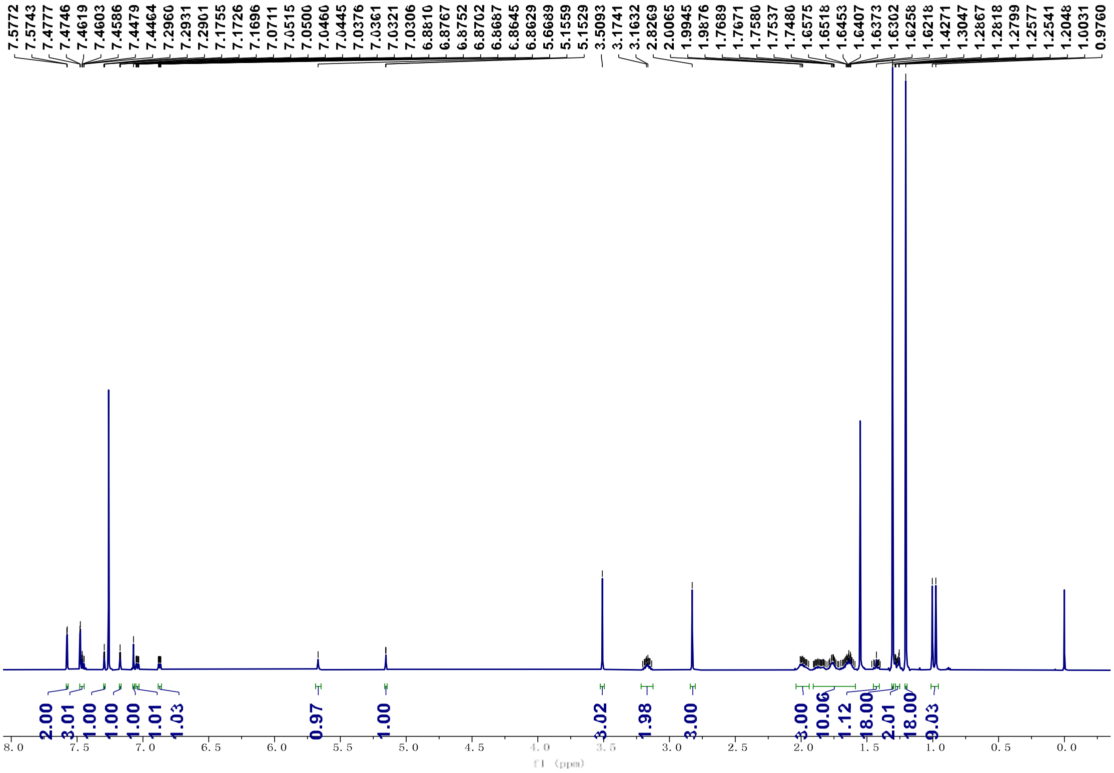

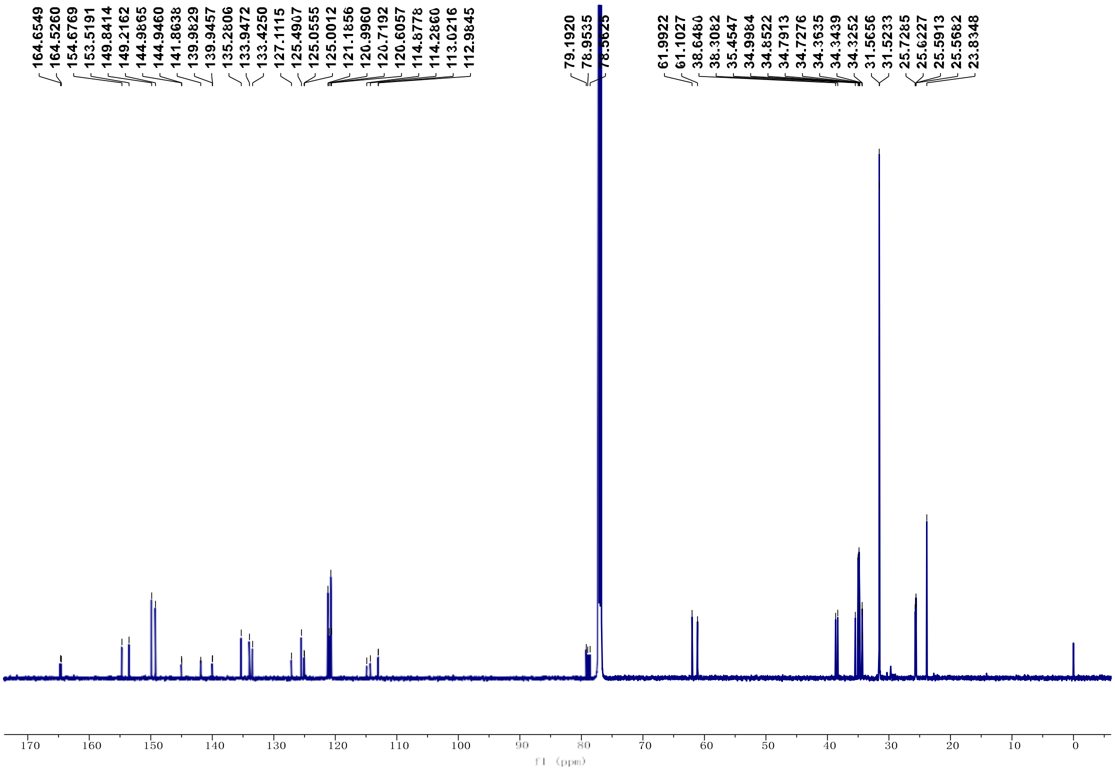
164.6549
164.5260
154.6769
153.5191
149.8414
149.2162
144.9865
144.9460
141.8638
139.9829
139.9457
135.2806
133.9472
133.4250
127.1115
125.4907
125.0555
125.0012
121.1856
120.9960
120.7192
120.6057
114.8778
114.2860
113.0216
112.9845
79.1920
78.9535
78.5625
61.9922
61.1027
38.6480
38.3082
35.4547
34.9984
34.8522
34.7913
34.7276
34.3635
34.3439
34.3252
31.5656
31.5233
25.7285
25.6227
25.5913
25.5682
23.8348
f1 (ppm)

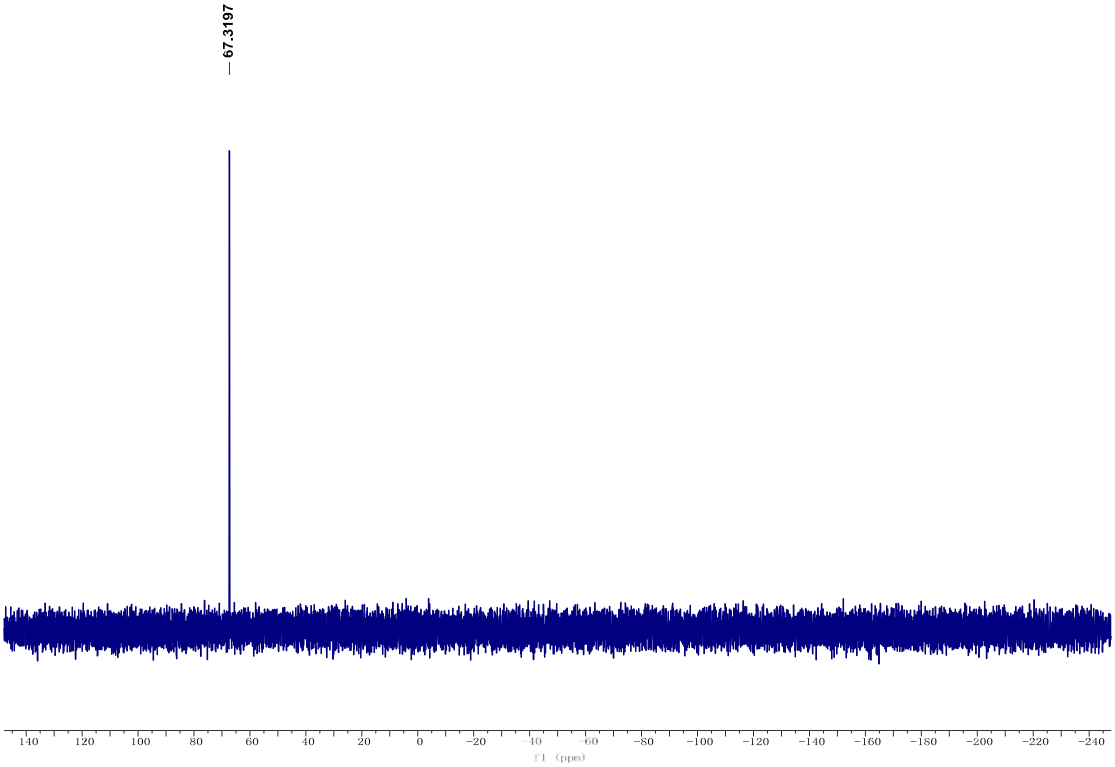
67.3197
f1 (ppm)

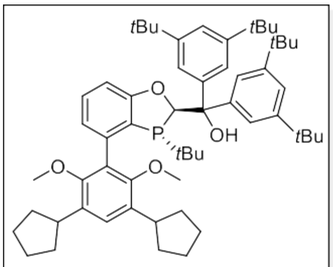

L10

L10 (white solid, 61% yield) $[\alpha]_D^{25}$ = -25.2 (c = 0.77, $CHCl_3$).

^{1}H NMR (600 MHz, $CDCl_3$) δ 7.46 (d, *J* = 1.8 Hz, 2H), 7.33 (d, *J* = 1.8 Hz, 2H), 7.31-7.29 (m, 2H), 7.25 (t, *J* = 1.8 Hz, 1H), 7.08 (s, 1H), 6.98 (dd, *J* = 5.5, 3.4 Hz, 1H), 6.92 (d, *J* = 8.1 Hz, 1H), 5.54 (s 1H), 3.69 (s, 3H), 3.31-3.21 (m, 5H), 2.94 (d, *J* = 4.2 Hz, 1H), 2.06-1.57 (m, 14H), 1.50-1.44 (m, 1H), 1.38-1.34 (m, 1H), 1.29 (d, *J* = 14.0 Hz, 36H), 0.59 (d, *J* = 12.1 Hz, 9H).

^{13}C NMR (151 MHz, $CDCl_3$) δ 165.4, 154.2, 154.0, 149.5, 149.2, 142.5 (*dd*, J = 9.2 Hz, 2.6 Hz), 139.3 (d, *J* = 17.7 Hz), 135.1, 134.4, 129.8, 129.0, 125.1 (d, *J* = 17.3 Hz), 124.4, 122.8 (d, *J* = 3.7 Hz), 122.2, 121.4 (d, *J* = 3.0 Hz), 120.8 (d, *J* = 13.4 Hz), 109.3, 90.7 (d, *J* = 30.3 Hz), 82.6 (d, *J* = 17.3 Hz), 62.6, 61.0, 38.8 (d, *J* = 54.4 Hz), 35.4, 34.9 (d, *J* = 9.7 Hz), 34.3, 34.1 (d, *J* = 11.7 Hz), 31.5 (d, *J* = 15.2 Hz), 30.7 (d, *J* = 20.9 Hz), 29.7, 27.0 (d, *J* = 14.9 Hz), 25.7, 25.5 (d, *J* = 2.8 Hz), 25.4.

^{31}P NMR (162 MHz, $CDCl_3$) δ -2.7.

HRMS (ESI) calculated for [M+H, $C_{58}H_{82}O_4P$]$^+$: 873.5945; found: 873.5946; [M+Na, $C_{58}H_{81}NaO_4P$]$^+$: 895.5765; found: 895.5760.

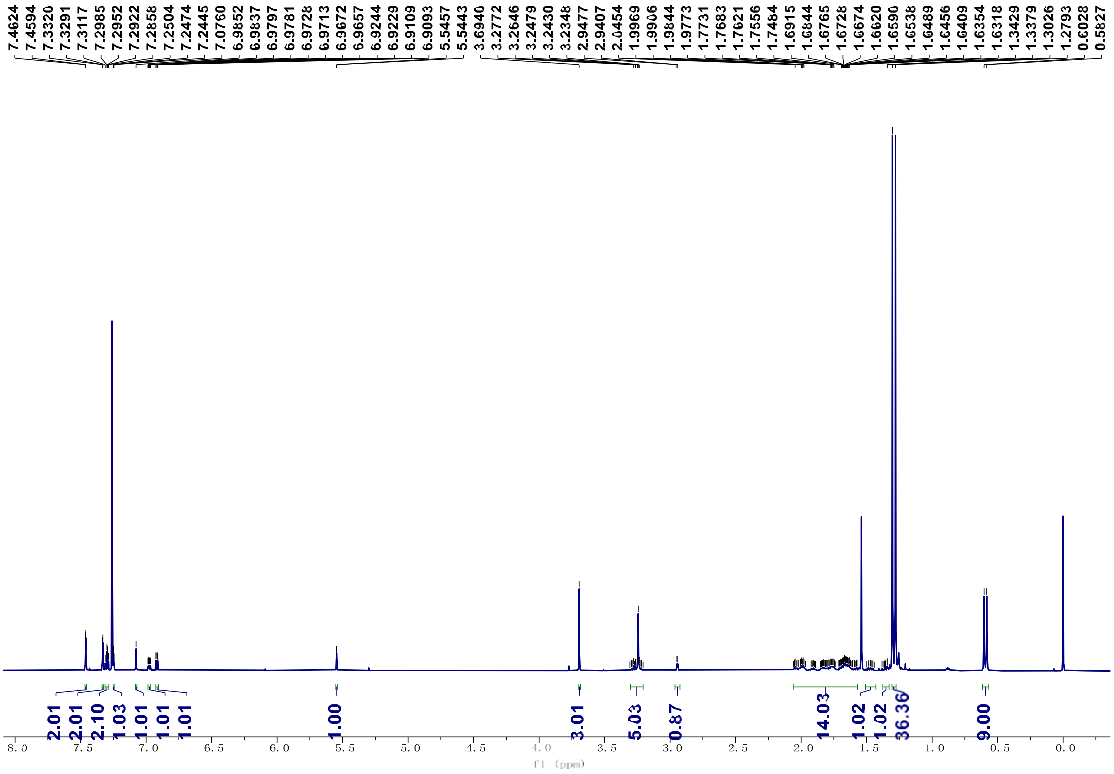

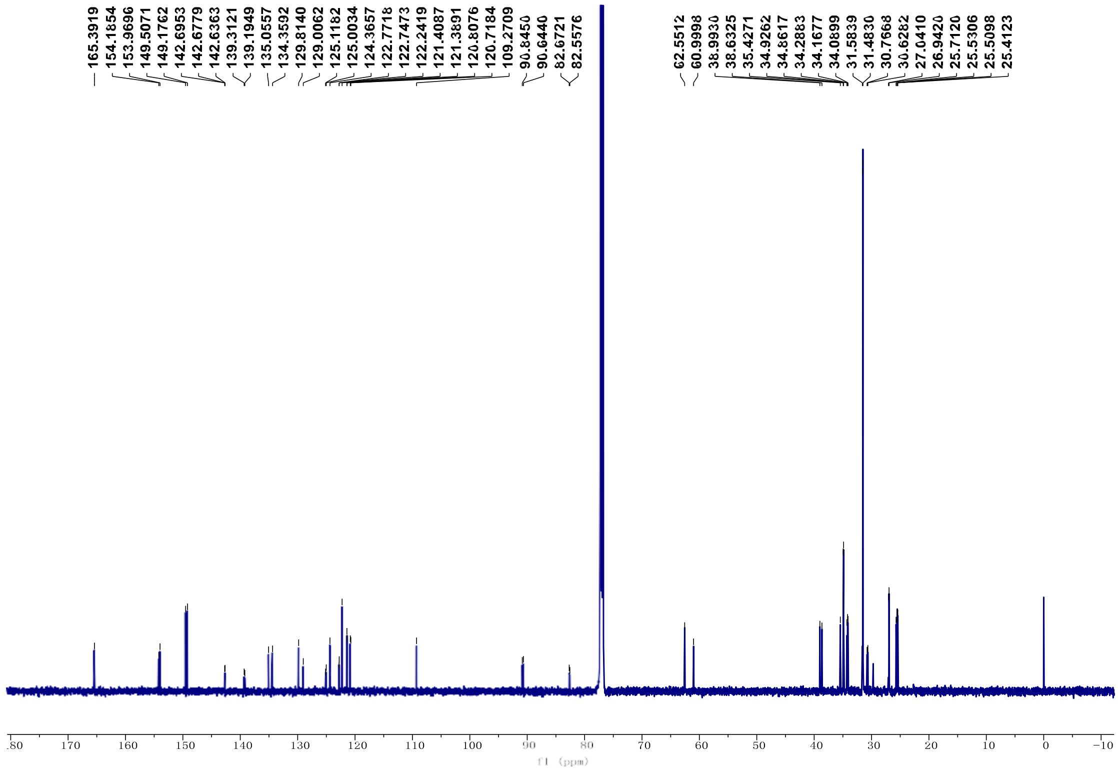
165.3919
154.1854
153.9696
149.5071
149.1762
142.6953
142.6779
142.6363
139.3121
139.1949
135.0557
134.3592
129.8140
129.0062
125.1182
125.0034
124.3657
122.7718
122.7473
122.2419
121.4087
121.3891
120.8076
120.7184
109.2709
90.8450
90.6440
82.6721
82.5576
62.5512
60.9998
38.9930
38.6325
35.4271
34.9262
34.8617
34.2883
34.1677
34.0899
31.5839
31.4830
30.7668
30.6282
27.0410
26.9420
25.7120
25.5306
25.5098
25.4123
f1 (ppm)

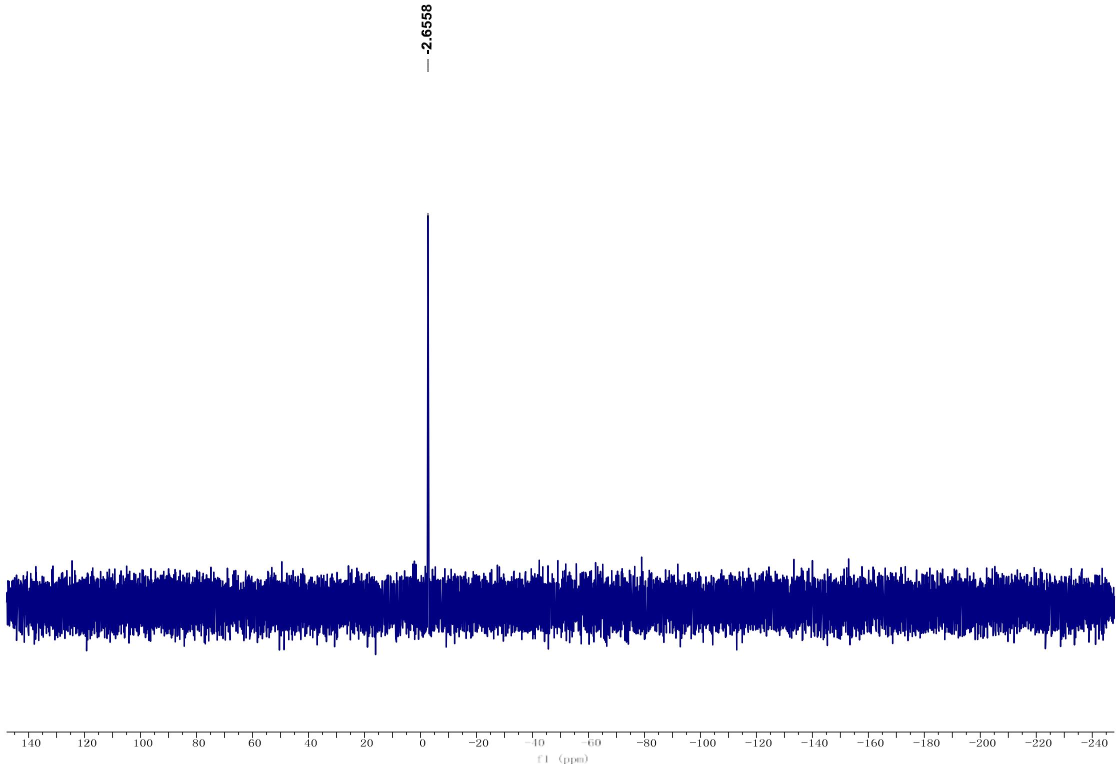
-2.6558
f1 (ppm)

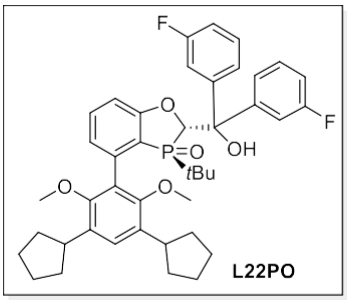

L22PO (white solid, 73% yield), $[\alpha]_D^{25}$ = 285.8 (c = 0.40, $CHCl_3$).

^{1}H NMR (600 MHz, $CDCl_3$) δ 7.45-7.43 (m, 2H), 7.38 (dt, *J* = 10.5, 2.2 Hz, 1H), 7.32-7.26 (m, 3H), 7.15-7.12 (m, 2H), 6.95-6.90 (m, 3H), 6.80 (td, *J* = 8.3, 2.6 Hz, 1H), 6.20 (s, 1H), 5.24-5.23 (m, 1H), 3.52 (d, *J* = 1.2 Hz, 3H), 3.31-3.15 (m, 1H), 3.22-3.16 (m, 1H), 3.14 (d, *J* = 1.2 Hz, 3H), 2.06-1.99 (m, 3H), 1.92-1.84 (m, 2H), 1.80-1.76 (m, 3H), 1.70-1.55 (m, 6H), 1.50-1.45 (m, 1H), 1.34-1.30 (m, 1H), 0.95 (d, *J* = 16.7 Hz, 9H).

^{13}C NMR (151 MHz, $CDCl_3$) δ 164.5 (d, *J* = 18.7 Hz), 163.5, 163.1, 161.9, 161.5, 154.5, 153.7, 148.2 (t, *J* = 6.0 Hz), 145.0 (dd, *J* = 6.9, 2.9 Hz), 139.6 (d, *J* = 5.7 Hz), 135.5, 134.3, 133.9, 129.7 (d, *J* = 8.2 Hz), 128.9 (d, *J* = 8.1 Hz), 126.9, 125.7, 125.3 (d, *J* = 8.7 Hz), 122.9 (d, *J* = 2.8 Hz), 121.8 (d, *J* = 2.8 Hz), 114.5 (d, *J* = 22.4 Hz), 113.8 (d, *J* = 4.8 Hz), 113.7, 113.5, 112.8 (d, *J* = 5.4 Hz), 79.6, 76.5, 62.1, 61.1, 38.7, 38.4, 35.5, 34.8, 34.5, 34.4, 34.2, 34.1, 25.7 (d, *J* = 15.5 Hz), 25.6, 23.6.

^{31}P NMR (162 MHz, $CDCl_3$) δ 69.2.

^{19}F NMR (376 MHz, $CDCl_3$) δ -112.5, -113.6.

ESI-MS: m/z 723.45 $[M+Na]^+$; HRMS (ESI) calculated for $[M+H, C_{42}H_{48}F_2O_5P]^+$: 701.3202; found: 701.3202; $[M+Na, C_{42}H_{47}F_2NaO_5P]^+$: 723.3021; found: 723.3022.

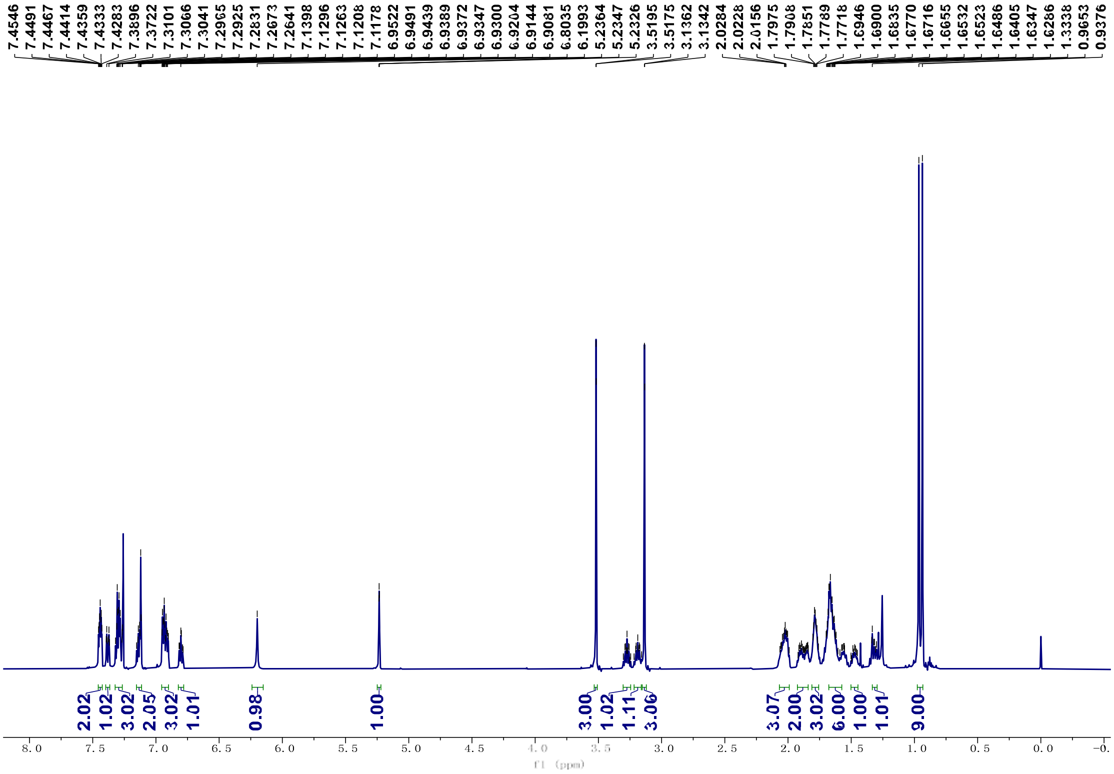

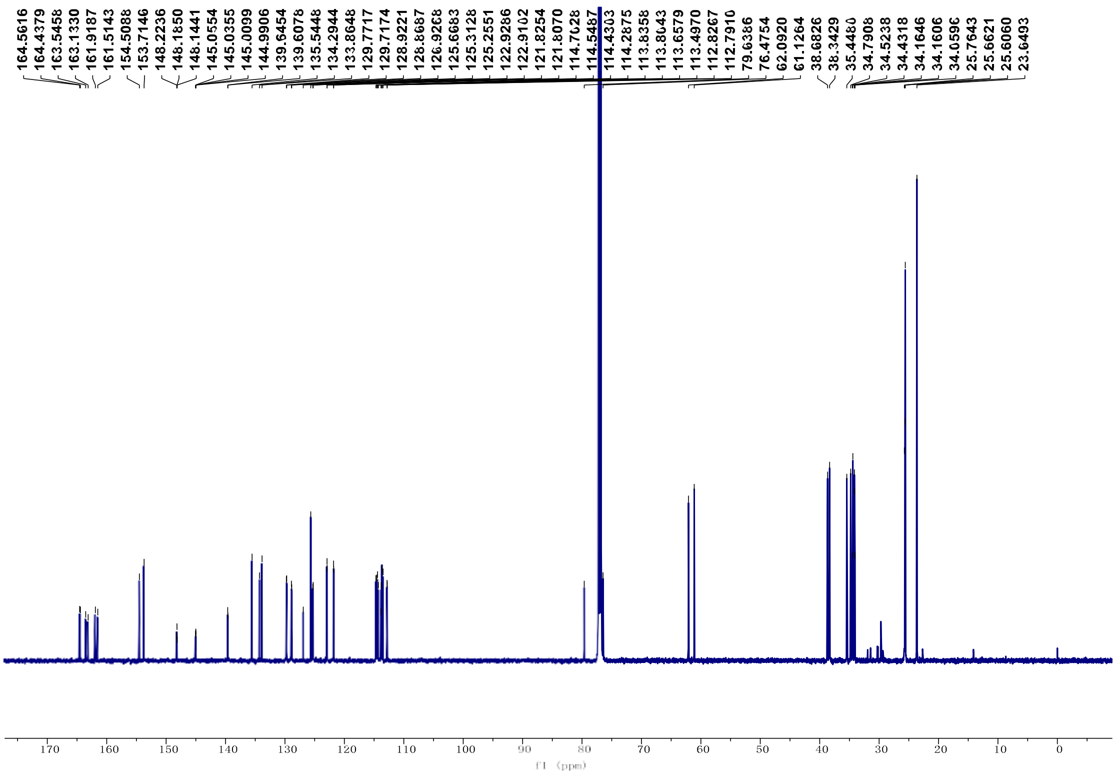

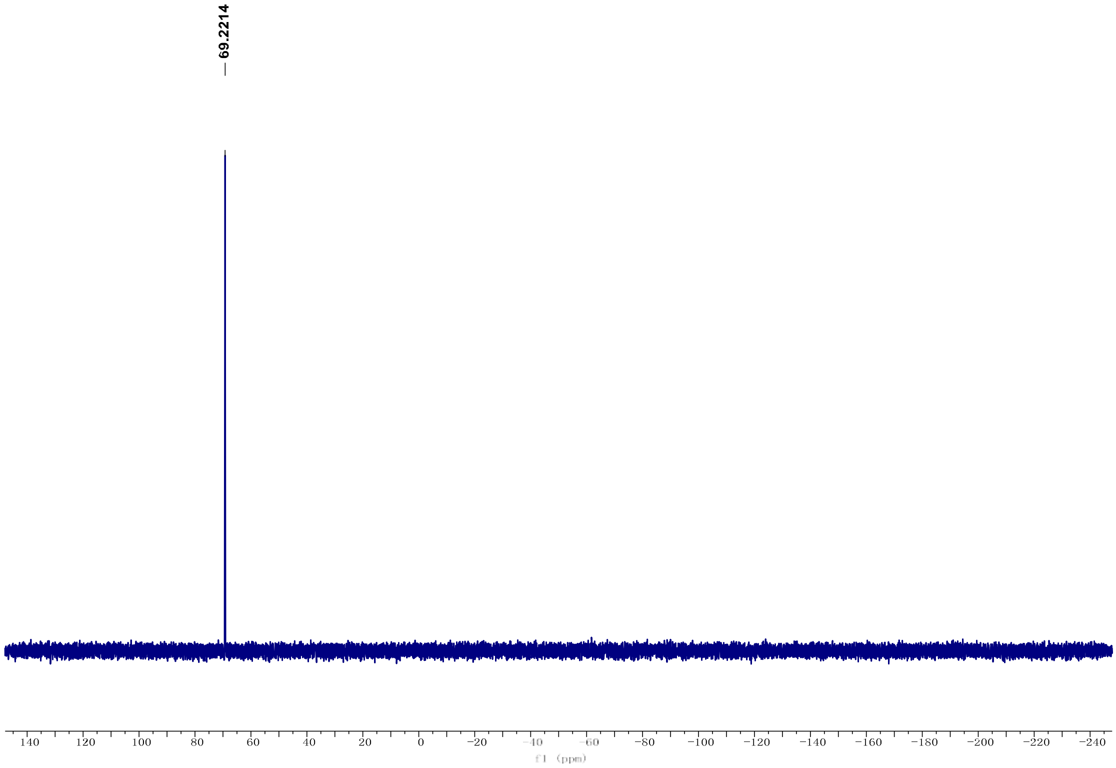
69.2214
f1 (ppm)

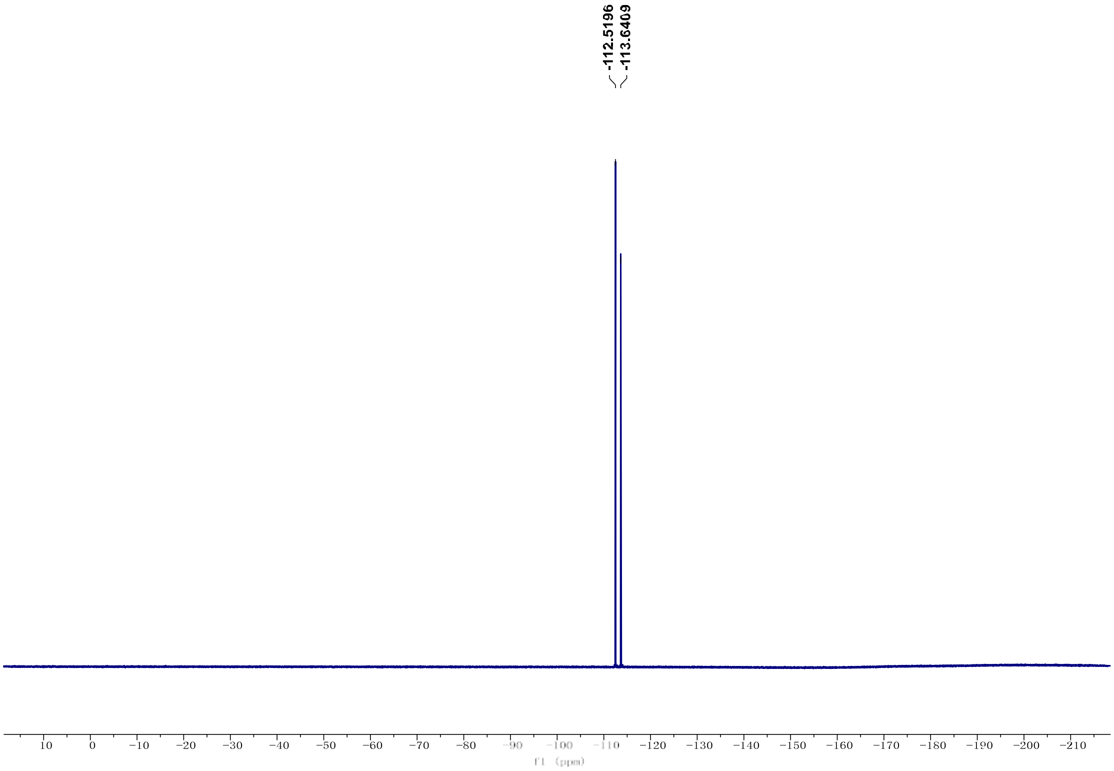

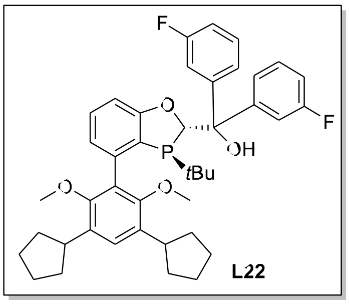

L22 (white solid, 74% yield), $[\alpha]_D^{25}$ = 27.2 (c = 0.65, $CHCl_3$).

^{1}H NMR (600 MHz, $CDCl_3$) δ 7.38 (dt, *J* = 7.9, 1.3 Hz, 1H), 7.35-7.33 (m, 1H), 7.31-7.28 (m, 2H), 7.24-7.22 (m, 2H), 7.15 (dt, *J* = 8.0, 1.3 Hz, 1H), 7.10 (s, 1H), 6.99 (dd, *J* = 7.5, 3.5 Hz, 1H), 6.93 (td, *J* = 8.3, 2.6 Hz, 1H), 6.90-6.87 (m, 1H), 6.85 (dd, *J* = 8.1 Hz, 1H), 5.61 (s, 1H), 3.75 (s, 3H), 3.30-3.21 (m, 6H), 2.13-2.03 (m, 2H), 1.99-1.84 (m, 4H), 1.78-1.65 (m, 8H), 1.45-1.42 (m, 1H), 1.35-1.31 (m, 1H), 0.66 (d, *J* = 12.4 Hz, 9H).

^{13}C NMR (151 MHz, $CDCl_3$) δ 165.1, 163.6 (d, J = 23.3 Hz), 161.9 (d, J = 22.3 Hz), 154.2, 154.0, 147.1 (dd, J = 6.5, 3.3 Hz), 146.2 (dd, J = 7.0, 3.3 Hz), 139.5 (d, J = 18.0 Hz), 135.5, 134.8, 130.6, 129.4, 129.3, 129.2, 124.8, 124.5 (d, J = 13.9 Hz), 122.6 (dd, J = 8.7, 3.4 Hz), 122.1 (d, J = 3.0 Hz), 114.5 (d, J = 22.9 Hz), 114.0 (d, J = 7.1 Hz), 113.9 (d, J = 7.3 Hz), 113.8 (d, J = 3.4 Hz), 113.6 (d, J = 3.3 Hz), 109.4, 89.0 (d, J = 29.9 Hz), 81.5 (d, J = 17.8 Hz), 63.0, 61.0, 39.5, 38.6, 35.5, 34.4, 34.2, 33.3, 30.8 (d, J = 20.1 Hz), 26.7 (d, J = 14.6 Hz), 25.7, 25.5 (d, J = 6.3 Hz).

^{31}P NMR (162 MHz, $CDCl_3$) δ -5.4.

^{19}F NMR (376 MHz, $CDCl_3$) δ -82.6 (dt, *J* = 14.6, 7.0 Hz), -83.1 (td, *J* = 9.2, 5.8 Hz).

ESI-MS: m/z 707.35 $[M+Na]^+$; HRMS (ESI) calculated for $[M+H, C_{42}H_{48}F_2O_5P]^+$: 685.3253; found: 685.3251; $[M+Na, C_{42}H_{47}F_2NaO_4P]^+$: 707.3072; found: 707.3070.

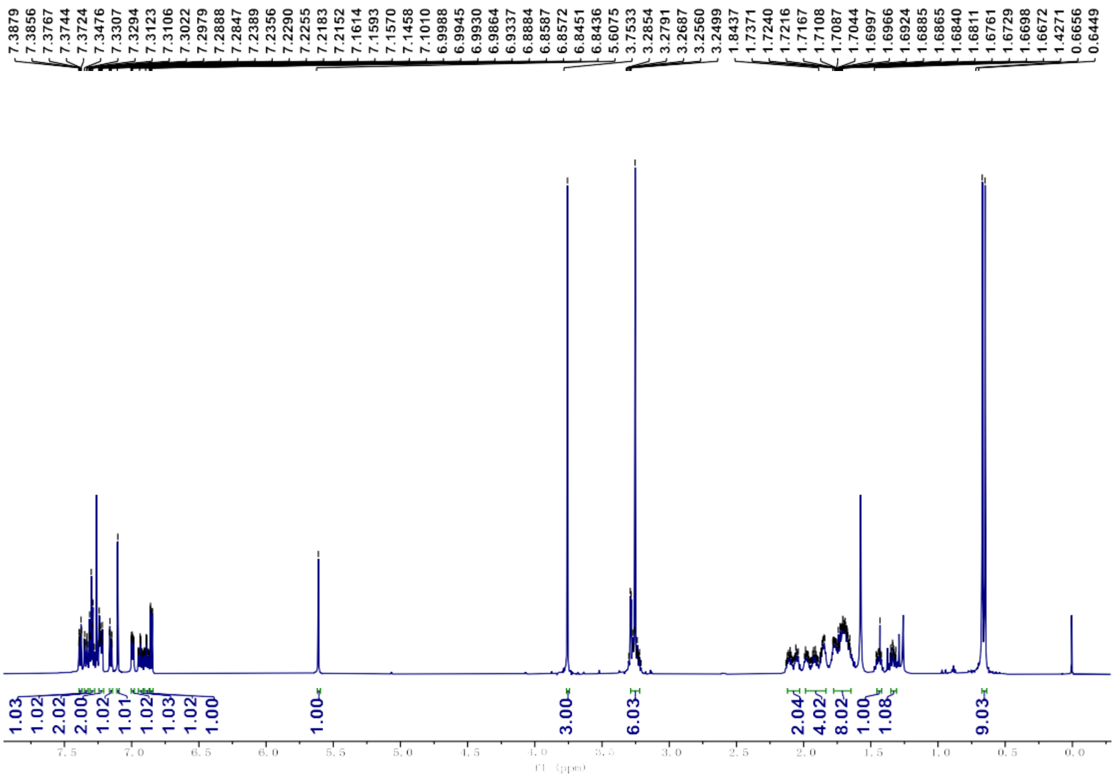

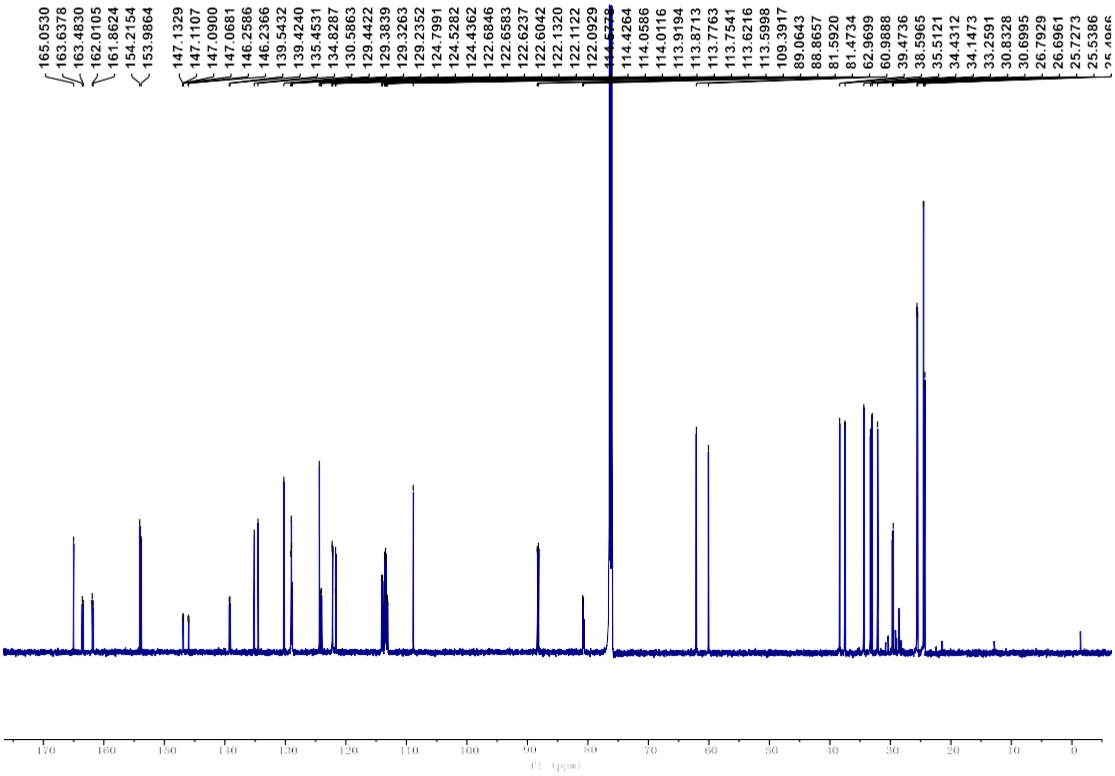

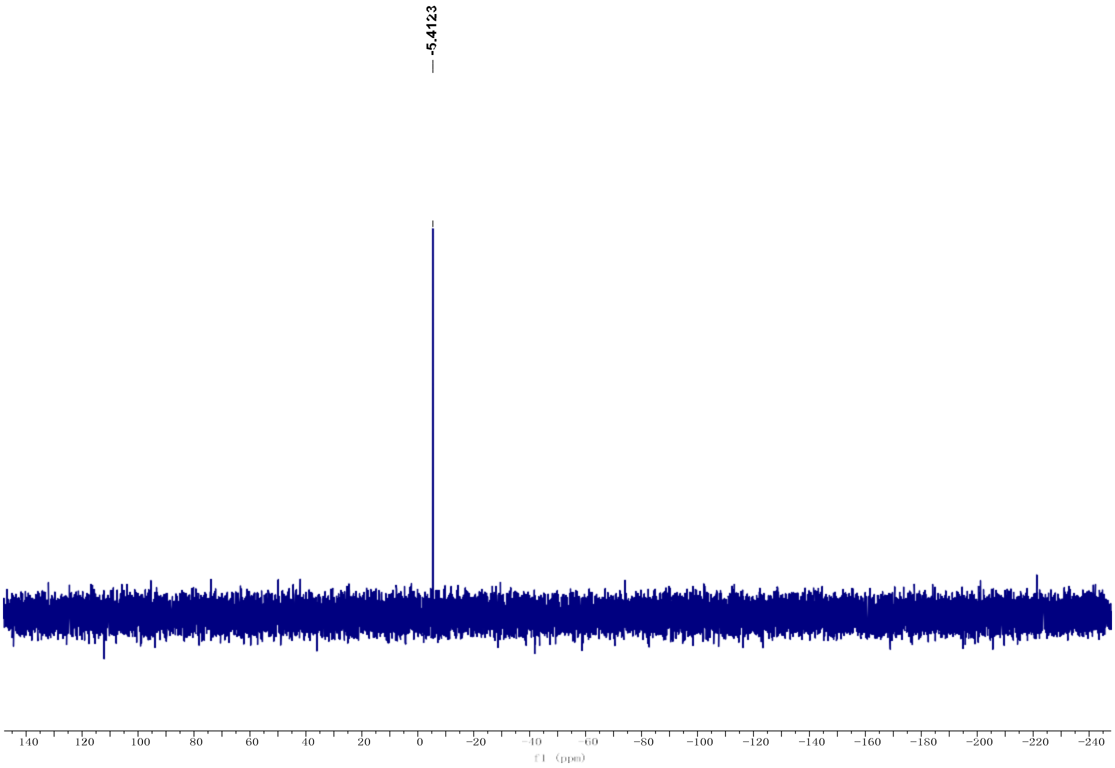
-5.4123
140 120 100 80 60 40 20 0 −20 −40 −60 −80 −100 −120 −140 −160 −180 −200 −220 −240
f1 (ppm)

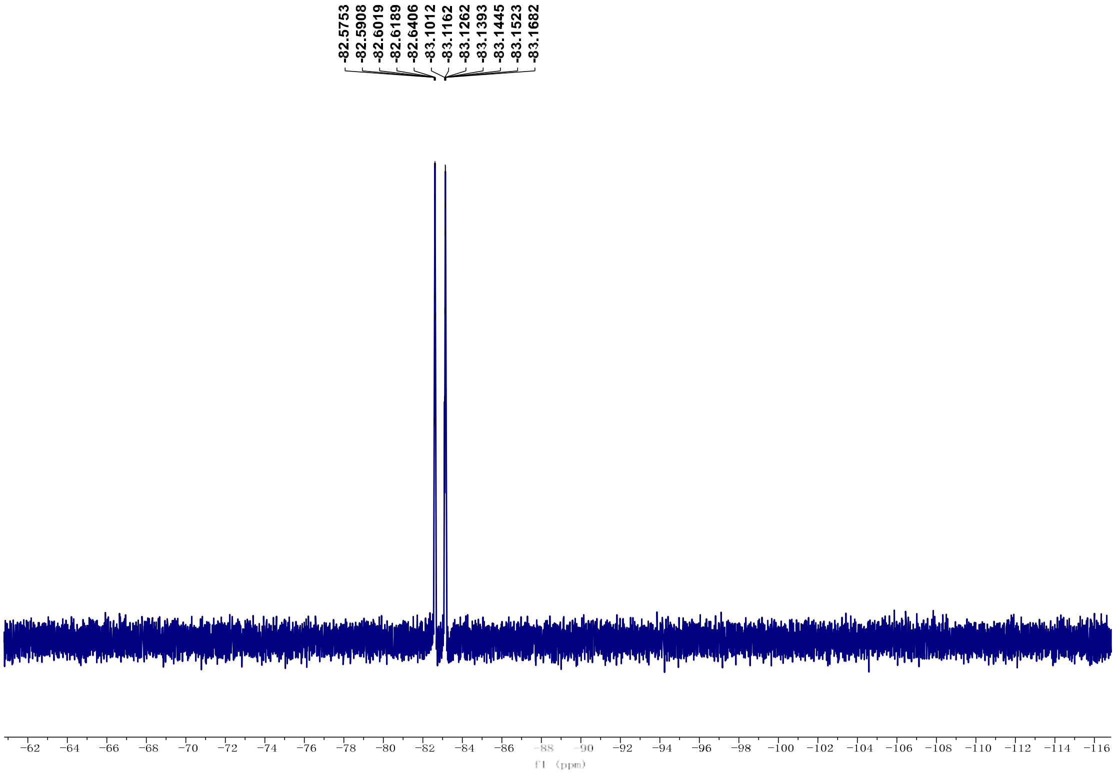
-82.5753
-82.5908
-82.6019
-82.6189
-82.6406
-83.1012
-83.1162
-83.1262
-83.1393
-83.1445
-83.1523
-83.1682
−62 −64 −66 −68 −70 −72 −74 −76 −78 −80 −82 −84 −86 −88 −90 −92 −94 −96 −98 −100 −102 −104 −106 −108 −110 −112 −114 −116
f1 (ppm)

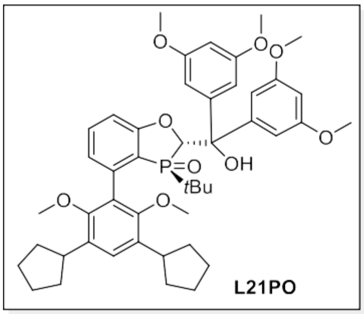

L21PO (white solid, 77% yield), $[\alpha]_D^{25}$ = 203.8 (c = 0.38, $CHCl_3$).

^{1}H NMR (600 MHz, $CDCl_3$) δ 7.43-7.40 (m, 1H), 7.10 (s, 1H), 6.95 (dd, *J* = 8.3, 3.3 Hz, 1H), 6.88 (dd, *J* = 7.4, 3.6 Hz, 1H), 6.85 (d, *J* = 2.3 Hz, 2H), 6.72 (d, *J* = 2.3 Hz, 2H), 6.34 (t, *J* = 2.2 Hz, 1H), 6.19 (t, *J* = 2.3 Hz, 1H), 5.23 (d, *J* = 1.8 Hz, 1H), 3.77 (s, 6H), 3.70 (s, 6H), 3.52 (s, 3H), 3.28-3.16 (m, 2H), 3.09 (s, 3H), 2.05-1.99 (m, 3H), 1.91-1.75 (m, 4H), 1.70-1.56 (m, 7H), 1.50-1.45 (m, 1H), 1.34-1.30 (m, 1H), 0.98 (d, *J* = 16.4 Hz, 9H).

^{13}C NMR (151 MHz, $CDCl_3$) δ 164.6 (d, *J* = 18.8 Hz), 160.5, 159.7, 154.7, 153.7, 148.5 (d, *J* = 6.1 Hz), 144.5 (d, *J* = 2.7 Hz), 139.6 (d, *J* = 5.7 Hz), 135.3, 134.1, 133.5, 127.0, 125.5, 125.0 (d, *J* = 8.6 Hz), 114.5, 113.9, 112.8 (d, *J* = 5.6 Hz), 105.6, 104.8, 99.4, 98.8, 79.8, 62.1, 61.0, 55.3 (d, *J* = 19.3 Hz), 38.8, 38.3, 35.5, 34.6 (d, *J* = 7.9 Hz), 34.4, 34.1 (d, *J* = 13.9 Hz), 31.6, 25.8, 25.6 (d, *J* = 2.4 Hz), 25.6, 23.7.

^{31}P NMR (162 MHz, $CDCl_3$) δ 68.8.

ESI-MS: m/z 807.50 $[M+Na]^+$; HRMS (ESI) calculated for $[M+H, C_{46}H_{58}O_9P]^+$: 785.3813; found: 785.3813; $[M+Na, C_{46}H_{57}NaO_9P]^+$: 807.3632; found: 807.3634.

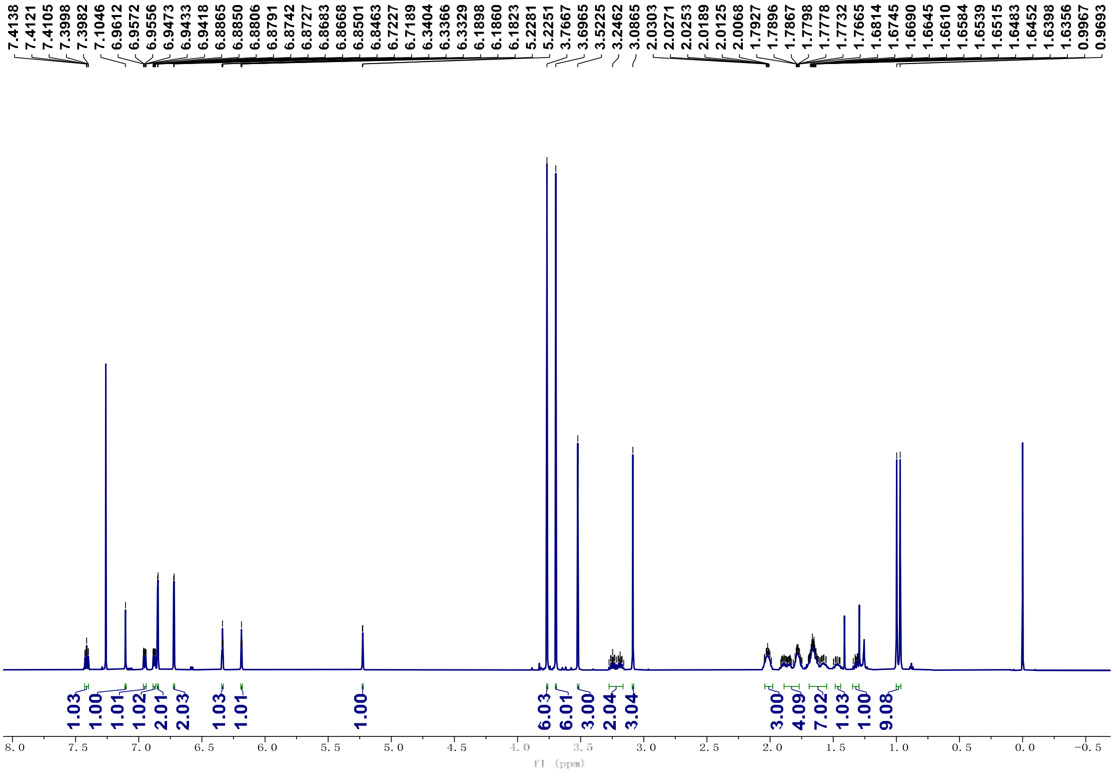

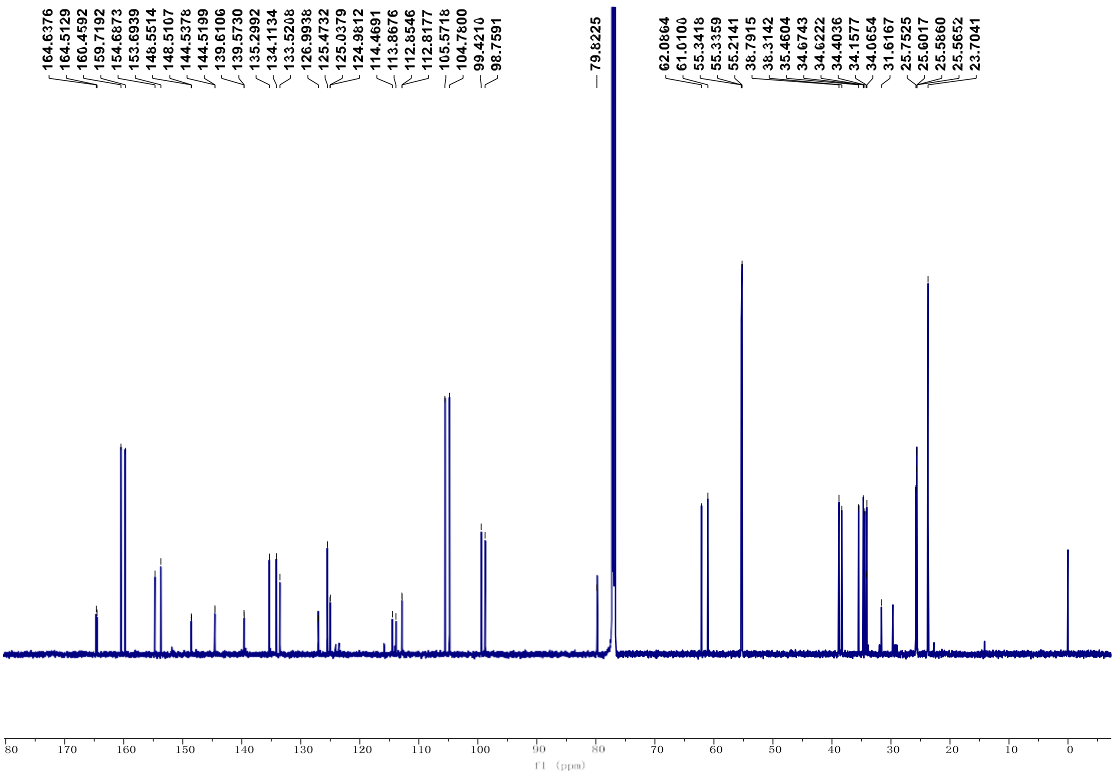
164.6376
164.5129
160.4592
159.7192
154.6873
153.6939
148.5514
148.5107
144.5378
144.5199
139.6106
139.5730
135.2992
134.1134
133.5208
126.9938
125.4732
125.0379
124.9812
114.4691
113.8676
112.8546
112.8177
105.5718
104.7800
99.4210
98.7591
79.8225
62.0864
61.0100
55.3418
55.3359
55.2141
38.7915
38.3142
35.4604
34.6743
34.6222
34.4036
34.1577
34.0654
31.6167
25.7525
25.6017
25.5860
25.5652
23.7041
f1 (ppm)

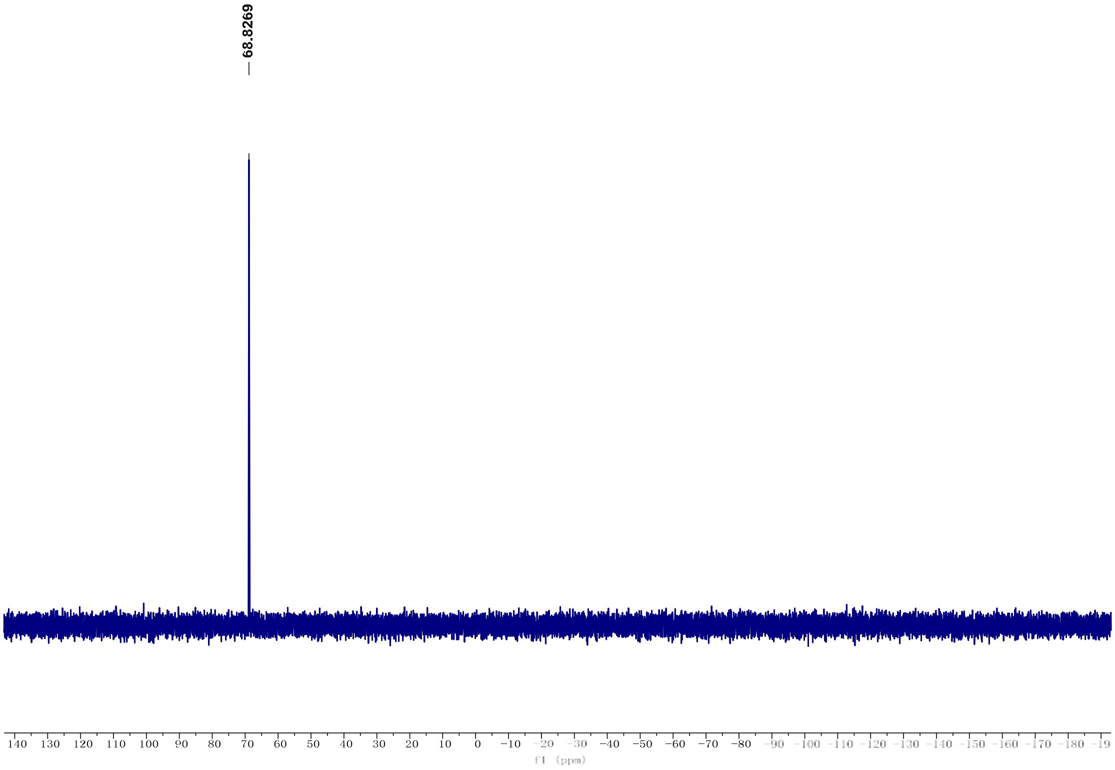
68.8269
f1 (ppm)

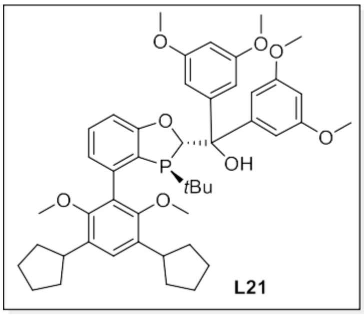

L21 (white solid, 51% yield), $[\alpha]_D^{25}$ = 89.6 (c = 0.22, $CHCl_3$).

^{1}H NMR (600 MHz, $CDCl_3$) δ 7.26 (dd, *J* = 8.1, 7.4 Hz, 1H), 7.08 (s, 1H), 6.95 (dd, *J* = 7.4, 3.4 Hz, 1H), 6.86 (d, *J* = 8.1 Hz, 1H), 6.80 (d, *J* = 2.3 Hz, 2H), 6.65 (d, *J* = 2.3 Hz, 2H), 6.33 (t, *J* = 2.3 Hz, 1H), 6.29 (t, *J* = 2.2 Hz, 1H), 5.61 (s, 1H), 3.76 (d, *J* = 13.0 Hz, 12H), 3.72 (s, 3H), 3.28-3.21 (m, 5H), 2.98 (d, *J* = 3.7 Hz, 1H), 2.08-2.02 (m, 2H), 1.99-1.61 (m, 12H), 1.48-1.43 (m, 1H), 1.36-1.31 (m, 1H), 0.68 (d, *J* = 12.2 Hz, 9H).

^{13}C NMR (151 MHz, $CDCl_3$) δ 165.2, 160.4, 160.2, 154.2, 154.0, 146.9 (d, *J* = 3.3 Hz), 146.3 (d, *J* = 3.4 Hz), 139.4, 139.3, 135.2, 134.6, 130.2, 129.2, 124.9, 124.8, 124.5, 122.7 (d, *J* = 3.9 Hz), 109.4, 105.7, 104.9 (d, *J* = 2.7 Hz), 98.7 (d, *J* = 2.5 Hz), 89.3, 89.1, 81.8 (d, *J* = 17.5 Hz), 62.8, 61.0, 55.3 (d, *J* = 7.1 Hz), 39.3, 38.6, 35.5, 34.3, 34.1, 33.6, 30.9, 30.7, 26.9 (d, *J* = 14.9 Hz), 25.7, 25.6, 25.5 (d, *J* = 6.9 Hz).

^{31}P NMR (162 MHz, $CDCl_3$) δ -4.1.

HRMS (ESI) calculated for $[M+H, C_{46}H_{58}O_8P]^+$: 769.3864; found: 769.3870; $[M+Na, C_{46}H_{57}NaO_8P]^+$: 791.3683; found: 791.3689.

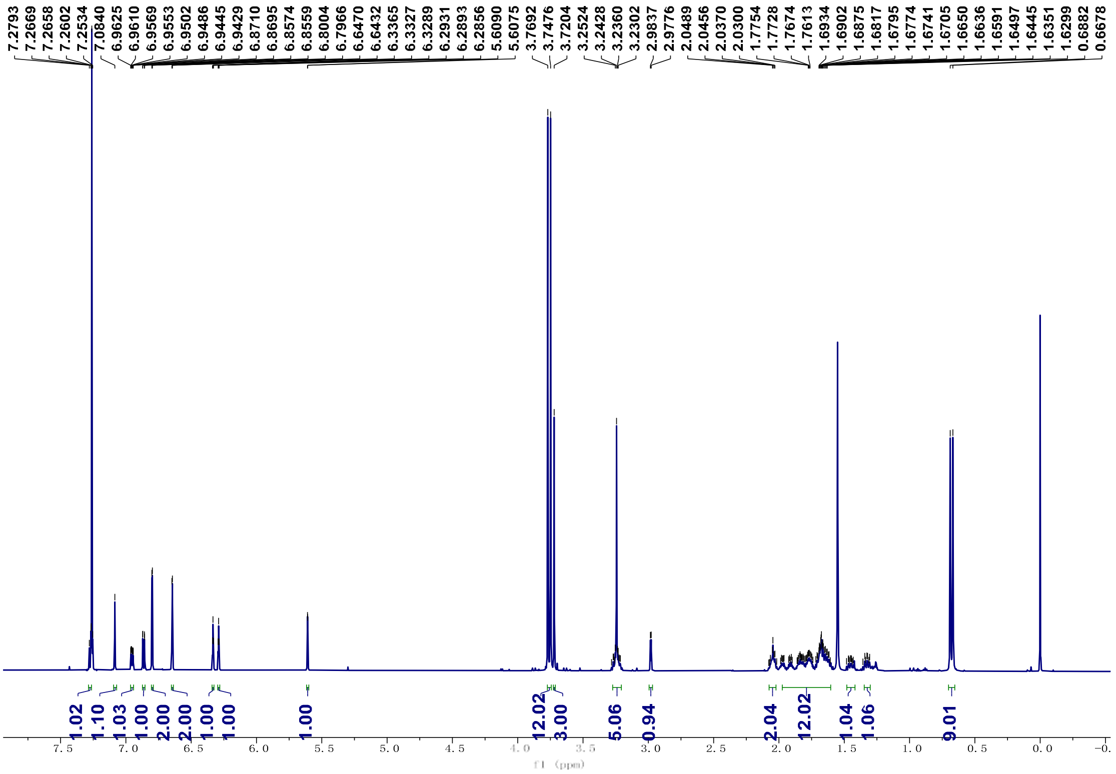

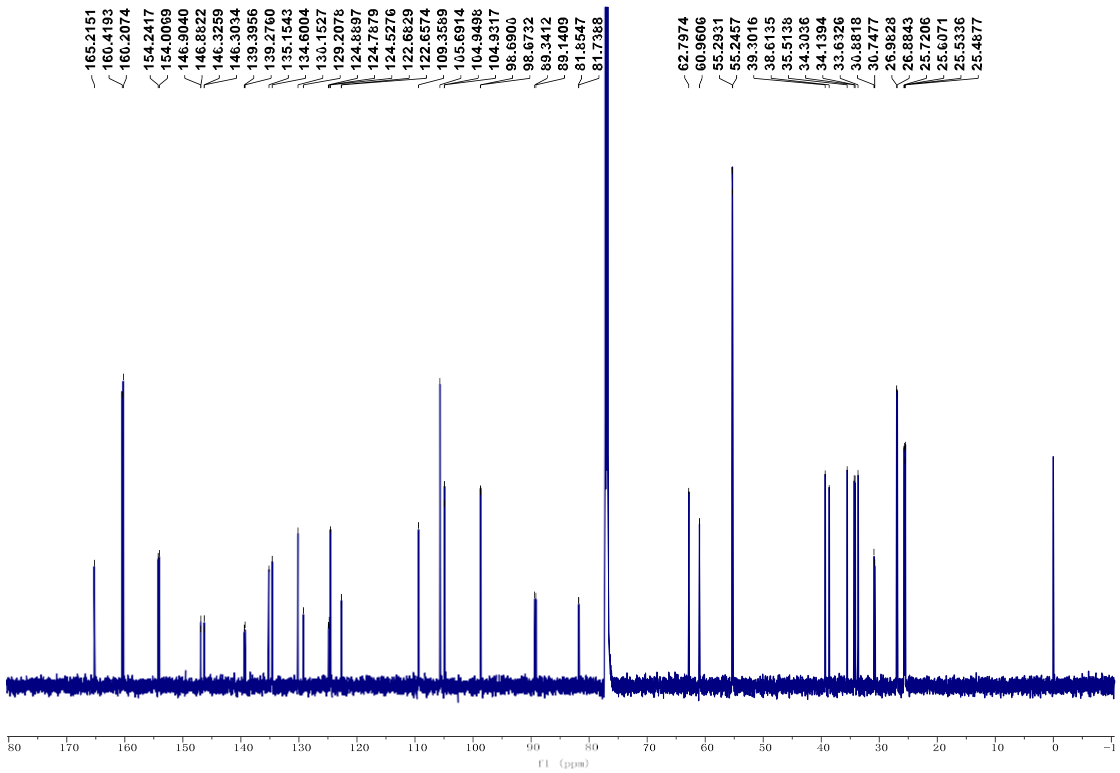
165.2151
160.4193
160.2074
154.2417
154.0069
146.9040
146.8822
146.3259
146.3034
139.3956
139.2760
135.1543
134.6004
130.1527
129.2078
124.8897
124.7879
124.5276
122.6829
122.6574
109.3589
105.6914
104.9498
104.9317
98.6900
98.6732
89.3412
89.1409
81.8547
81.7388
62.7974
60.9606
55.2931
55.2457
39.3016
38.6135
35.5138
34.3036
34.1394
33.6326
30.8818
30.7477
26.9828
26.8843
25.7206
25.6071
25.5336
25.4877
f1 (ppm)

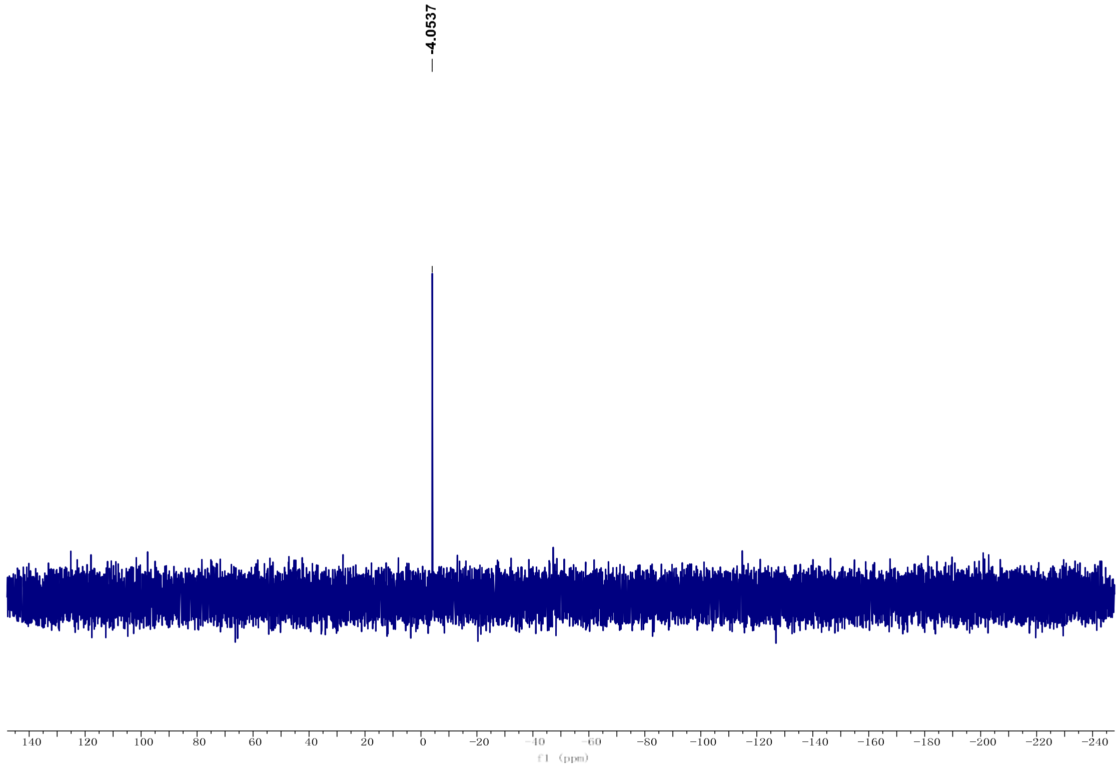
-4.0537
f1 (ppm)

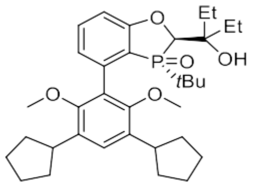

L20PO

L20PO (colourless oil, 84% yield), $[\alpha]_D^{25}$ = -62.8 (c = 1.00, $CHCl_3$).

^{1}H NMR (500 MHz, $CDCl_3$) δ 7.48-7.45 (m, 1H), 7.15 (s, 1H), 6.95 (ddd, *J* = 11.6, 7.8, 3.3 Hz, 2H), 4.41 (d, *J* = 2.6 Hz, 1H), 3.96 (s, 1H), 3.58 (s, 3H), 3.44 (s, 3H), 3.33 (tt, *J* = 9.7, 7.5 Hz, 1H), 3.22 (tt, *J* = 9.4, 7.4 Hz, 1H), 2.09-2.01 (m, 3H), 1.96-1.66 (m, 14H), 1.59-1.49 (m, 2H), 1.37-1.33 (m, 1H), 1.00-0.96 (m, 12H), 0.88 (t, *J* = 7.5 Hz, 3H).

^{13}C NMR (151 MHz, $CDCl_3$) δ 165.2, 165.0, 154.6, 153.9, 139.6 (d, *J* = 5.6 Hz), 135.5, 134.3, 133.5, 127.2, 125.5, 124.7 (d, *J* = 8.5 Hz), 114.9, 114.3, 112.4 (d, *J* = 5.5 Hz), 77.7 (d, *J* = 2.6 Hz), 76.0, 75.6, 62.2, 61.3, 38.5, 38.5, 35.5, 34.9, 34.5, 34.4, 34.3, 33.8, 28.2 (d, *J* = 4.1 Hz), 26.6 (d, *J* = 3.4 Hz), 25.8, 25.7, 25.6, 23.7, 7.6, 7.2.

^{31}P NMR (162 MHz, $CDCl_3$) δ 67.0.

HRMS (ESI) calculated for [M+H, $C_{46}H_{58}O_8P]^+$: 569.3390; found: 569.3395; [M+Na, $C_{46}H_{57}NaO_8P]^+$: 591.3210; found: 591.3216.

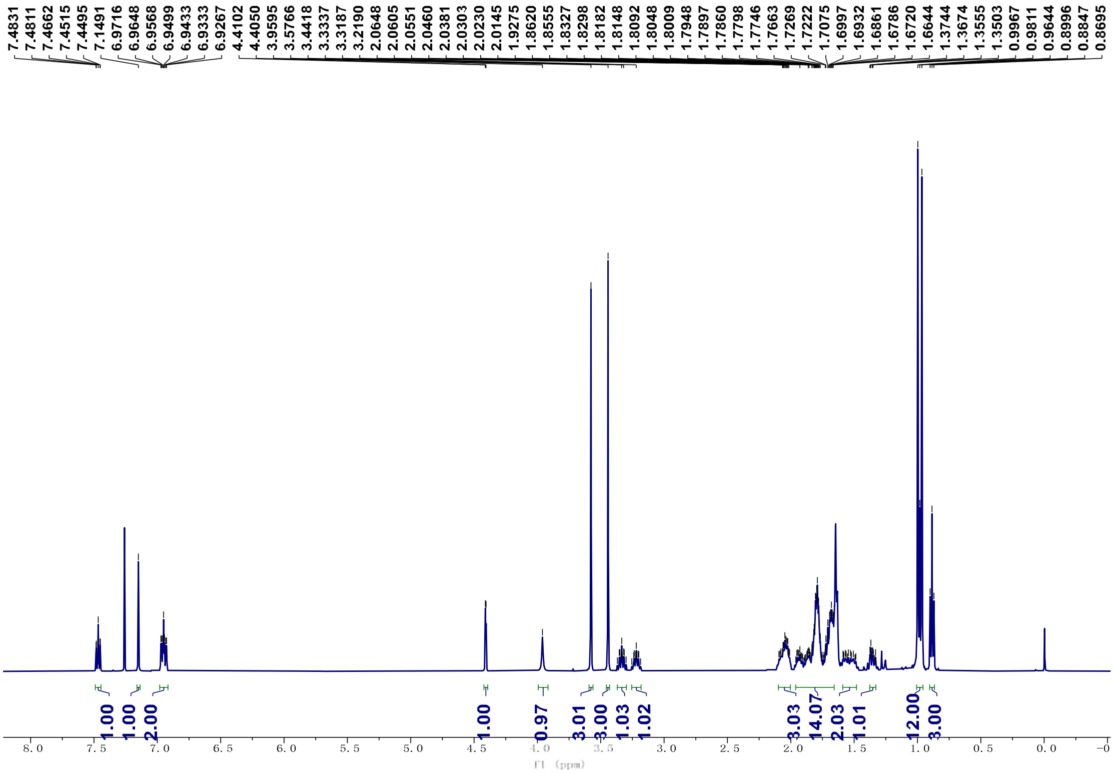

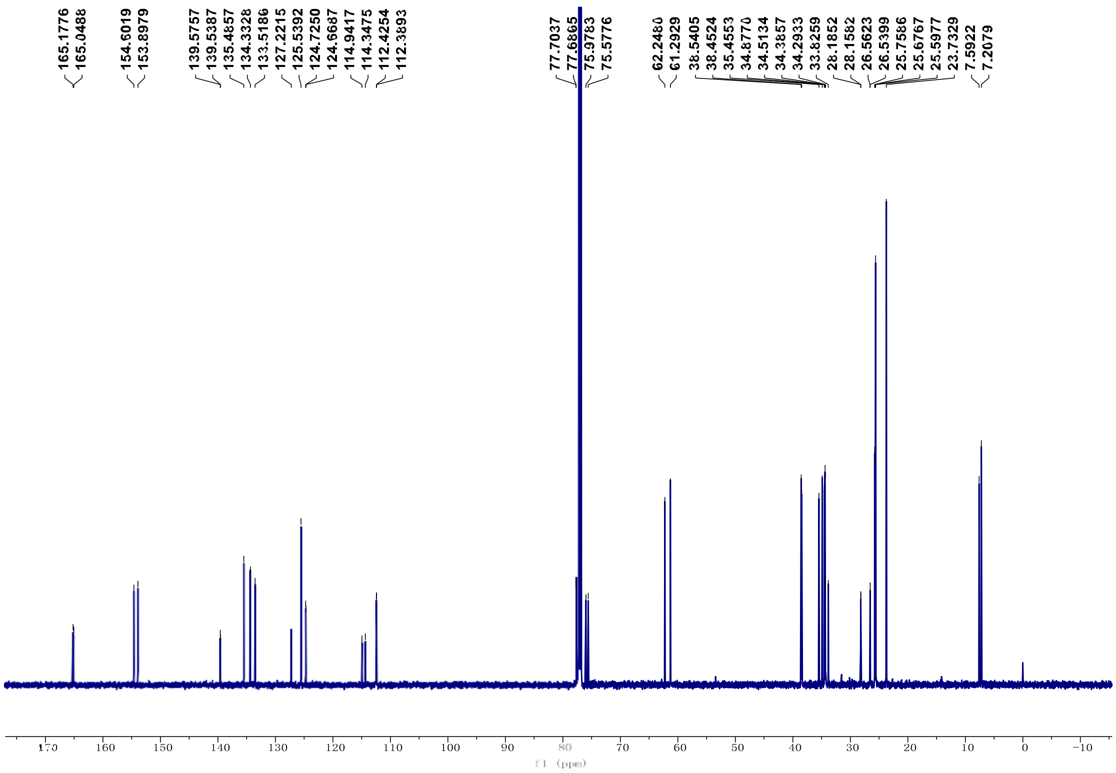
165.1776
165.0488
154.6019
153.8979
139.5757
139.5387
135.4857
134.3328
133.5186
127.2215
125.5392
124.7250
124.6687
114.9417
114.3475
112.4254
112.3893
77.7037
77.6865
75.9783
75.5776
62.2480
61.2929
38.5405
38.4524
35.4553
34.8770
34.5134
34.3857
34.2933
33.8259
28.1852
28.1582
26.5623
26.5399
25.7586
25.6767
25.5977
23.7329
7.5922
7.2079
f1 (ppm)

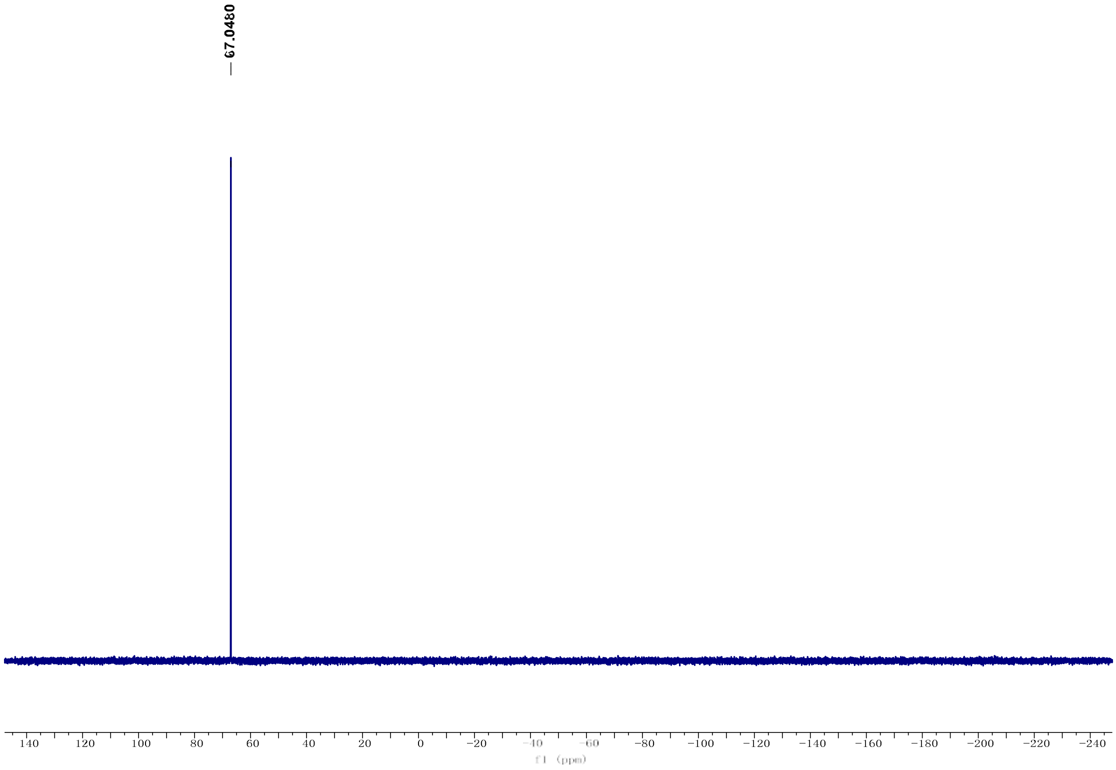
67.0480
f1 (ppm)

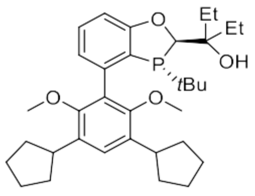

L20

L20 (yellow white oil, 84% yield), $[\alpha]_D^{25}$ = -19.1 (c = 1.00, $CHCl_3$).

^{1}H NMR (600 MHz, $CDCl_3$) δ 7.29 (t, *J* = 7.8 Hz, 1H), 7.12 (s, 1H), 6.97 (dd, *J* = 7.4, 3.3 Hz, 1H), 6.89 (d, *J* = 8.1 Hz, 1H), 4.82 (d, *J* = 1.1 Hz, 1H), 3.70 (s, 3H), 3.33 (tt, *J* = 9.7, 7.5 Hz, 1H), 3.26 (tt, *J* = 9.5, 7.4 Hz, 1H), 3.18 (s, 3H), 2.11-2.04 (m, 2H), 2.01-1.76 (m, 7H), 1.71-1.61 (m, 9H), 1.56-1.52 (m, 1H), 1.43-1.36 (m, 1H), 0.93 (dt, *J* = 12.6, 7.5 Hz, 6H), 0.73 (d, *J* = 12.3 Hz, 9H).

^{13}C NMR (151 MHz, $CDCl_3$) δ 164.9, 154.4, 153.7, 139.5, 139.4, 135.3, 134.6, 130.1, 129.1, 124.6, 124.5, 124.4, 122.9 (d, *J* = 4.1 Hz), 109.2, 89.1, 88.9, 77.1, 62.6, 60.8, 38.9, 38.7, 35.5, 34.3, 34.2 (d, *J* = 5.0 Hz), 30.8, 30.7, 27.6 (d, *J* = 9.0 Hz), 27.0, 26.9, 26.6 (d, *J* = 6.1 Hz), 25.8, 25.7, 25.6, 25.5, 7.9, 7.4.

^{31}P NMR (162 MHz, $CDCl_3$) δ -4.2.

HRMS (ESI) calculated for [M+H, $C_{46}H_{58}O_8P$]$^+$: 553.3441; found: 553.3441; [M+Na, $C_{46}H_{57}NaO_8P$]$^+$: 575.3261; found: 575.3261.

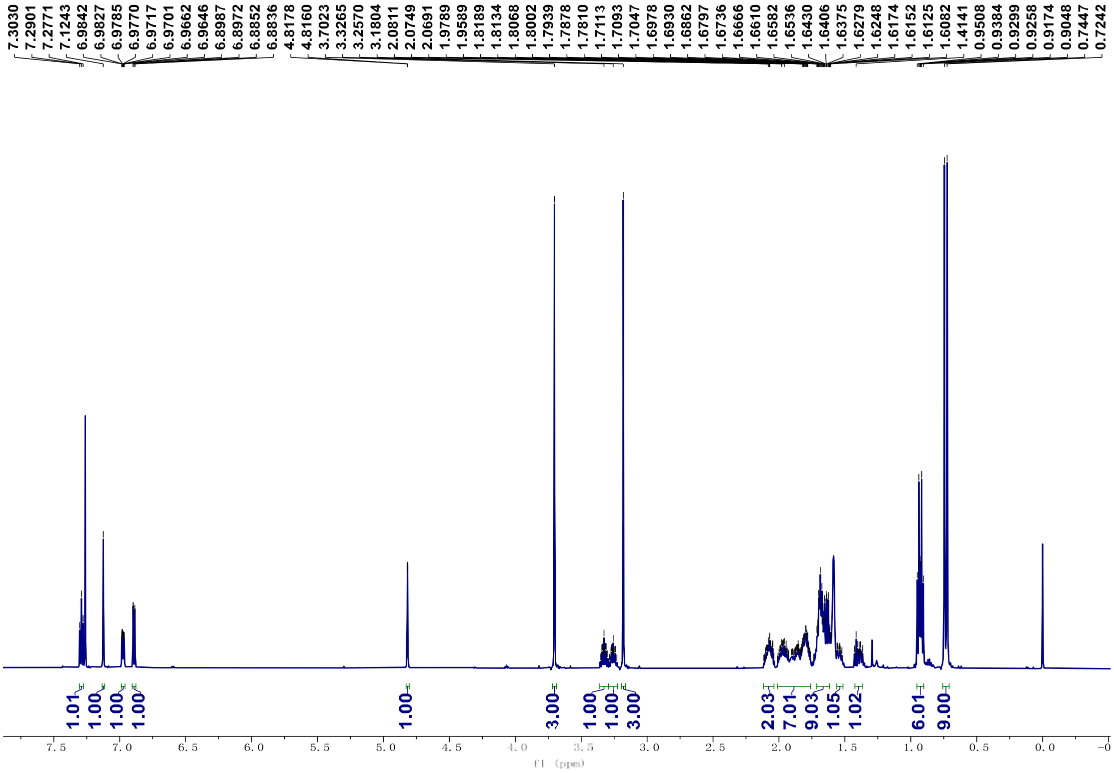

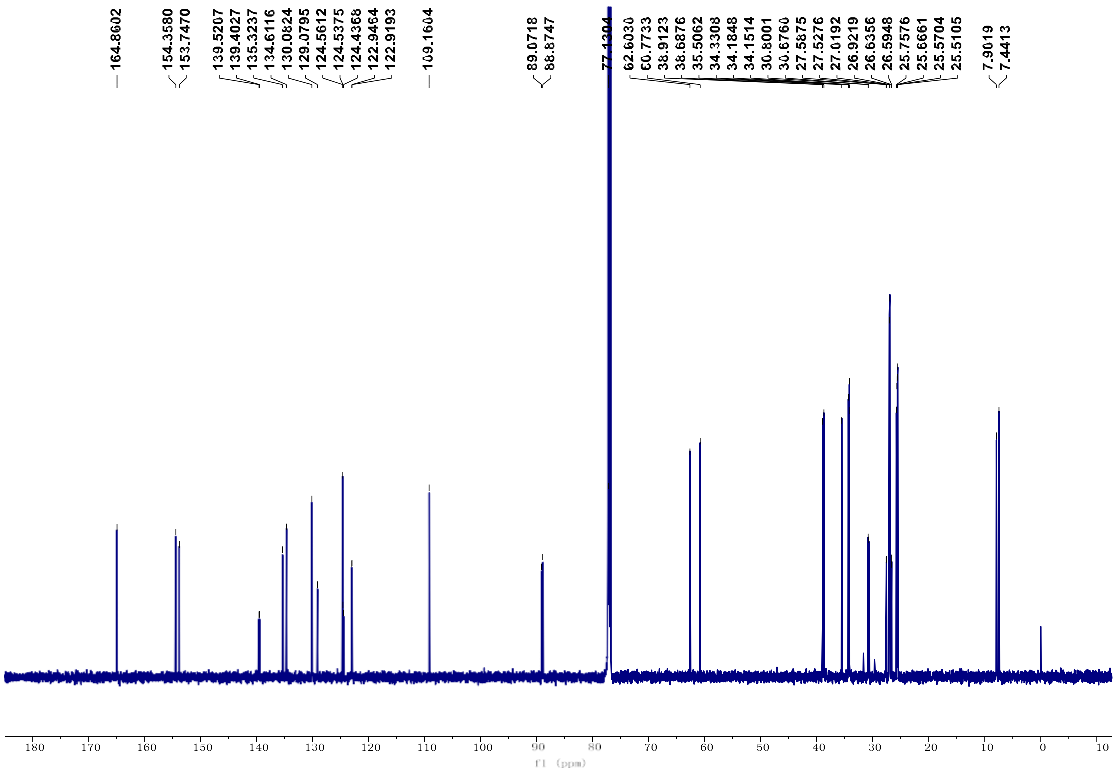
164.8602
154.3580
153.7470
139.5207
139.4027
135.3237
134.6116
130.0824
129.0795
124.5612
124.5375
124.4368
122.9464
122.9193
109.1604
89.0718
88.8747
77.1304
62.6030
60.7733
38.9123
38.6876
35.5062
34.3308
34.1848
34.1514
30.8001
30.6760
27.5875
27.5276
27.0192
26.9219
26.6356
26.5948
25.7576
25.6661
25.5704
25.5105
7.9019
7.4413
f1 (ppm)

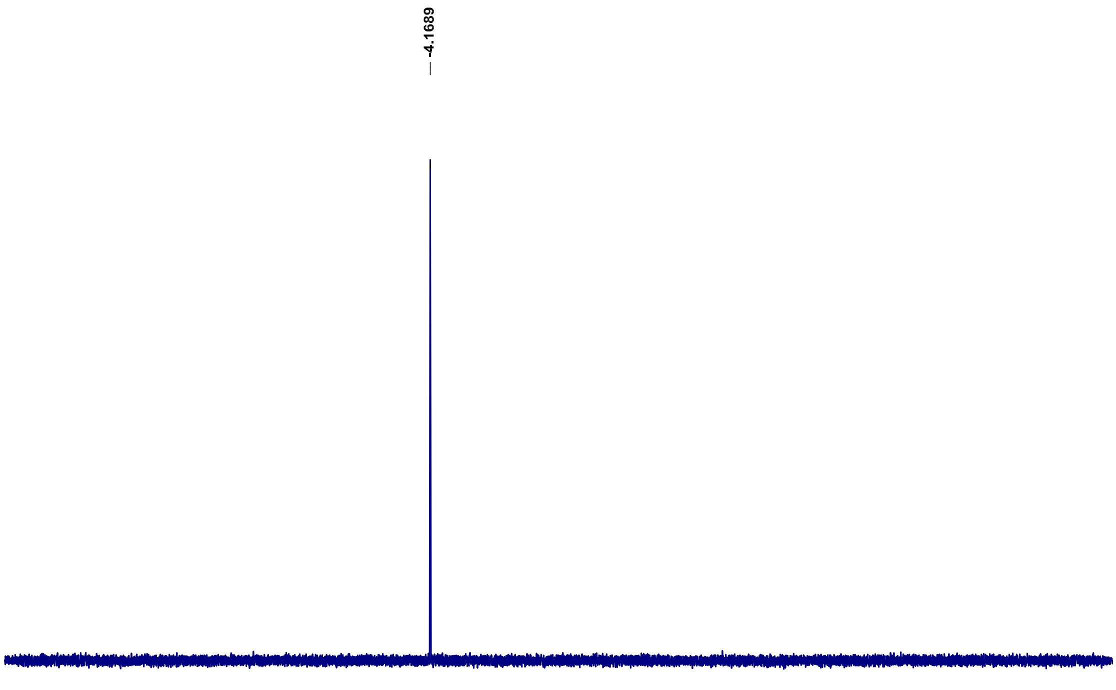
-4.1689
f1 (ppm)

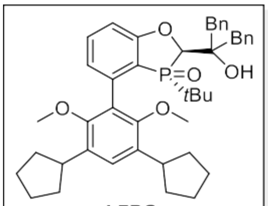

L7PO

L7PO (yellow solid, 96% yield), $[\alpha]_D^{25}$ = -199.05 (c = 0.28, $CHCl_3$).

^{1}H NMR (600 MHz, $CDCl_3$) δ 7.53 (dd, *J* = 8.3, 7.3 Hz, 1H), 7.32-7.26 (m, 7H), 7.25-7.18 (m, 3H), 7.15 (s, 1H), 7.07 (dd, *J* = 8.4, 3.4 Hz, 1H), 7.00 (dd, *J* = 7.4, 3.4 Hz, 1H), 4.31 (d, *J* = 2.5 Hz, 1H), 4.29 (s, 1H), 3.55 (s, 3H), 3.47 (d, *J* = 14.1 Hz, 1H), 3.45 (s, 3H), 3.31 (tt, *J* = 9.7, 7.5 Hz, 1H), 3.20 (tt, *J* = 9.7, 7.5 Hz, 1H), 2.90 (d, *J* = 13.8 Hz, 2H), 2.78 (dd, *J* = 13.9, 3.0 Hz, 1H), 2.13-2.09 (m, 1H), 2.05-2.00 (m, 2H), 1.94-1.85 (m, 2H), 1.81-1.76 (m, 3H), 1.70-1.60 (m, 7H), 1.53-1.50 (m, 1H), 0.77 (d, *J* = 16.3 Hz, 9H).

^{13}C NMR (151 MHz, $CDCl_3$) δ 165.0 (d, *J* = 19.7 Hz), 154.6, 153.8, 139.9 (d, *J* = 5.5 Hz), 137.3, 137.0, 135.6, 134.4, 133.8, 131.2, 131.1, 127.9, 127.8, 127.1, 126.3, 126.2, 125.7, 125.1 (d, *J* = 8.0 Hz), 115.3, 114.7, 112.6 (d, *J* = 5.4 Hz), 72.7, 72.3, 62.1, 61.2, 42.7 (d, *J* = 6.8 Hz), 42.0, 38.7, 38.4, 35.4, 34.8, 34.5, 34.4, 34.1, 25.8, 25.7, 25.7, 25.6, 23.3.

^{31}P NMR (162 MHz, $CDCl_3$) δ 67.1.

ESI-MS: m/z 693.50 $[M+H]^+$; 715.45 $[M+Na]^+$; HRMS (ESI) calculated for $[M+H, C_{44}H_{54}O_5P]^+$: 693.3703; found: 693.3704; $[M+Na, C_{44}H_{53}NaO_5P]^+$: 715.3523; found: 715.3525.

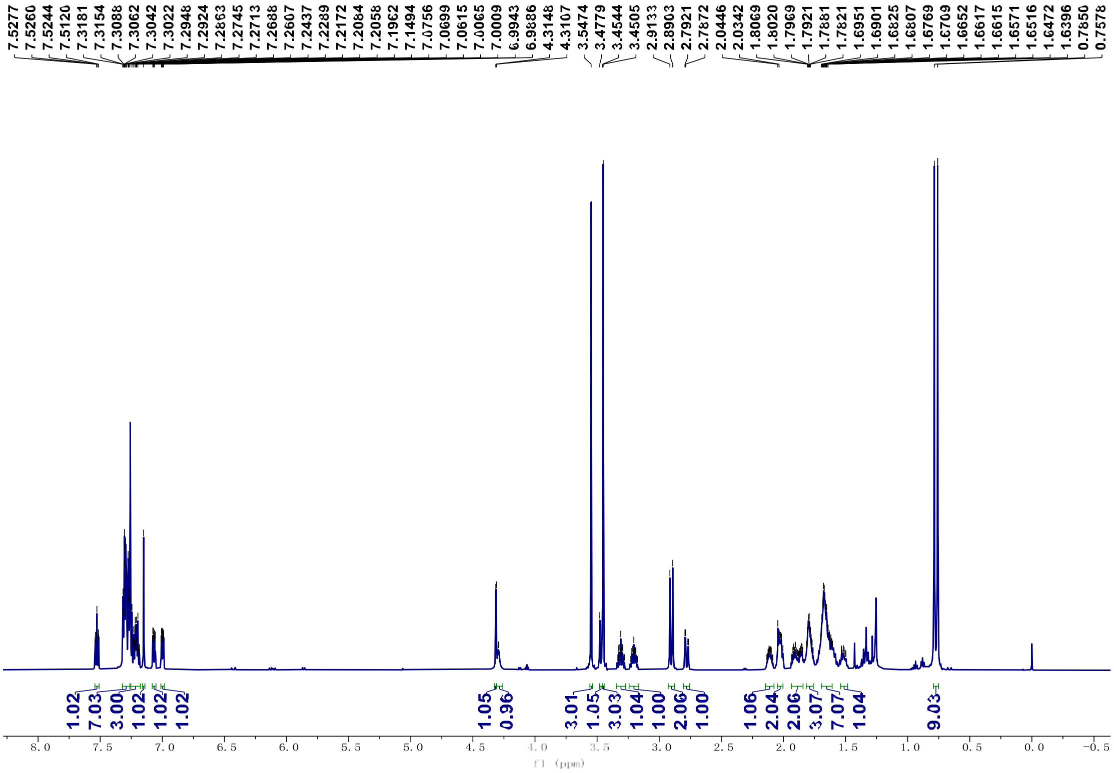

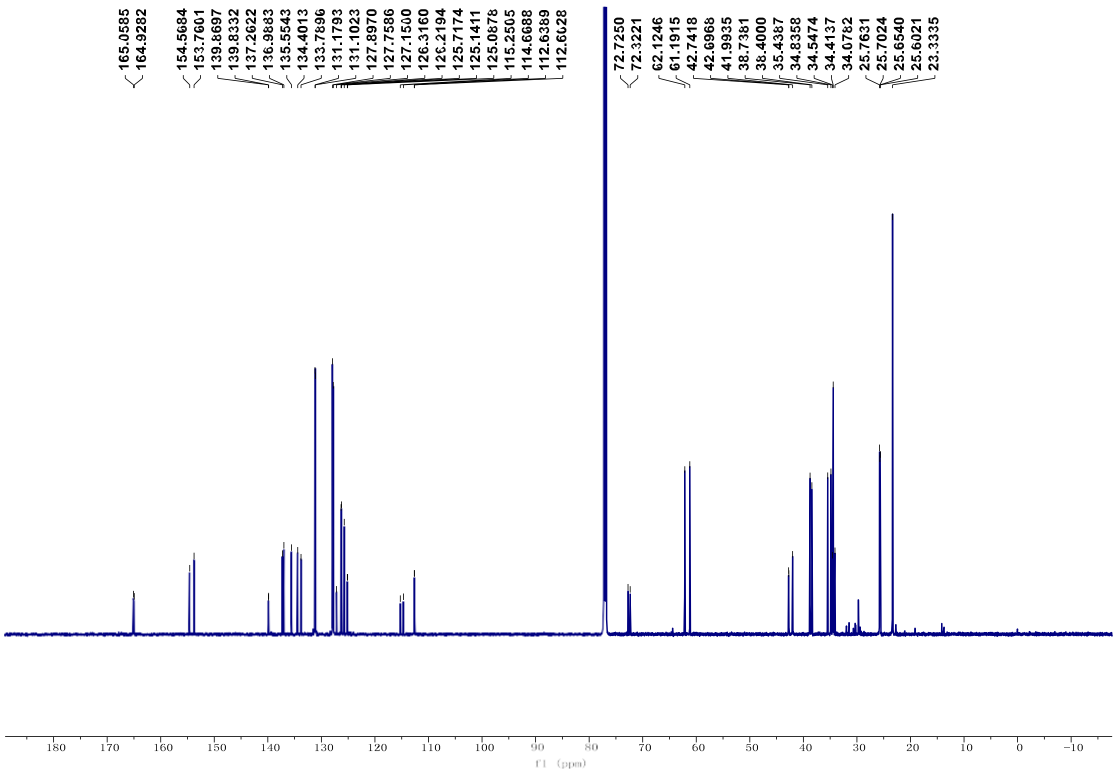

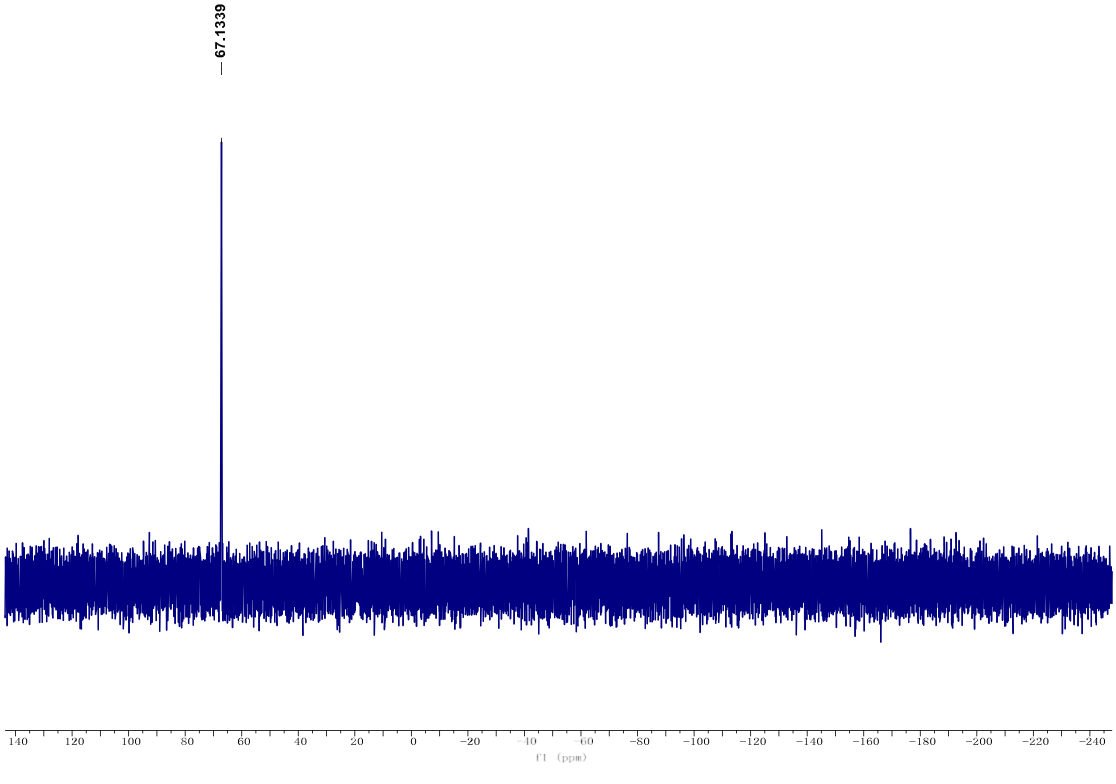

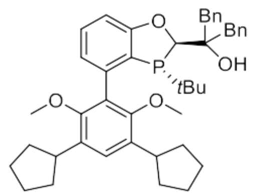

L7

L7 (white solid, 76% yield), $[\alpha]_D^{25}$ = -33.2 (c = 0.50, $CHCl_3$).

^{1}H NMR (600 MHz, $CDCl_3$) δ 7.35-7.27 (m, 9H), 7.23-7.19 (m, 2H), 7.13 (s, 1H), 7.03-6.99 (m, 2H), 4.67 (d, *J* = 1.0 Hz, 1H), 3.70 (s, 3H), 3.35 (tt, *J* = 9.4, 7.6 Hz, 1H), 3.25 (tt, *J* = 9.5, 7.4 Hz, 1H), 3.12 (s, 3H), 3.01 (t, *J* = 14.4 Hz, 2H), 2.75 (dd, *J* = 13.8, 3.1 Hz, 1H), 2.69 (d, *J* = 14.0 Hz, 1H), 2.18-2.13 (m, 1H), 2.10-2.05 (m, 2H), 2.00-1.92 (m, 2H), 1.88-1.77 (m, 4H), 1.74-1.64 (m, 6H), 1.40-1.36 (m, 1H), 0.55 (d, *J* = 12.4 Hz, 9H).

^{13}C NMR (151 MHz, $CDCl_3$) δ 164.7, 154.4, 153.8, 139.7 (d, *J* = 15.6 Hz), 137.2 (d, J = 15.8 Hz), 135.5, 134.6, 131.1 (d, *J* = 6.6 Hz), 130.2, 129.0, 128.0 (d, J = 11.6 Hz), 126.2 (d, *J* = 3.9 Hz), 124.8 (d, *J* = 3.8 Hz), 124.7, 123.3 (d, *J* = 4.1 Hz), 109.4, 87.2 (d, *J* = 28.9 Hz), 76.2 (d, *J* = 15.3 Hz), 62.6, 60.6, 41.8 (d, *J* = 8.6 Hz), 41.4 (d, *J* = 4.2 Hz), 39.1, 38.7, 35.5, 34.4, 34.2 (d, J = 9.2 Hz), 31.0, 30.9, 26.8, 26.7, 25.8, 25.6 (d, *J* = 9.0 Hz).

^{31}P NMR (162 MHz, $CDCl_3$) δ -5.0.

HRMS (ESI) calculated for [M+H, $C_{36}H_{54}O_5P$]$^+$: 677.3754; found: 677.3754; [M+Na, $C_{36}H_{53}NaO_5P$]$^+$: 699.3574; found: 699.3573.

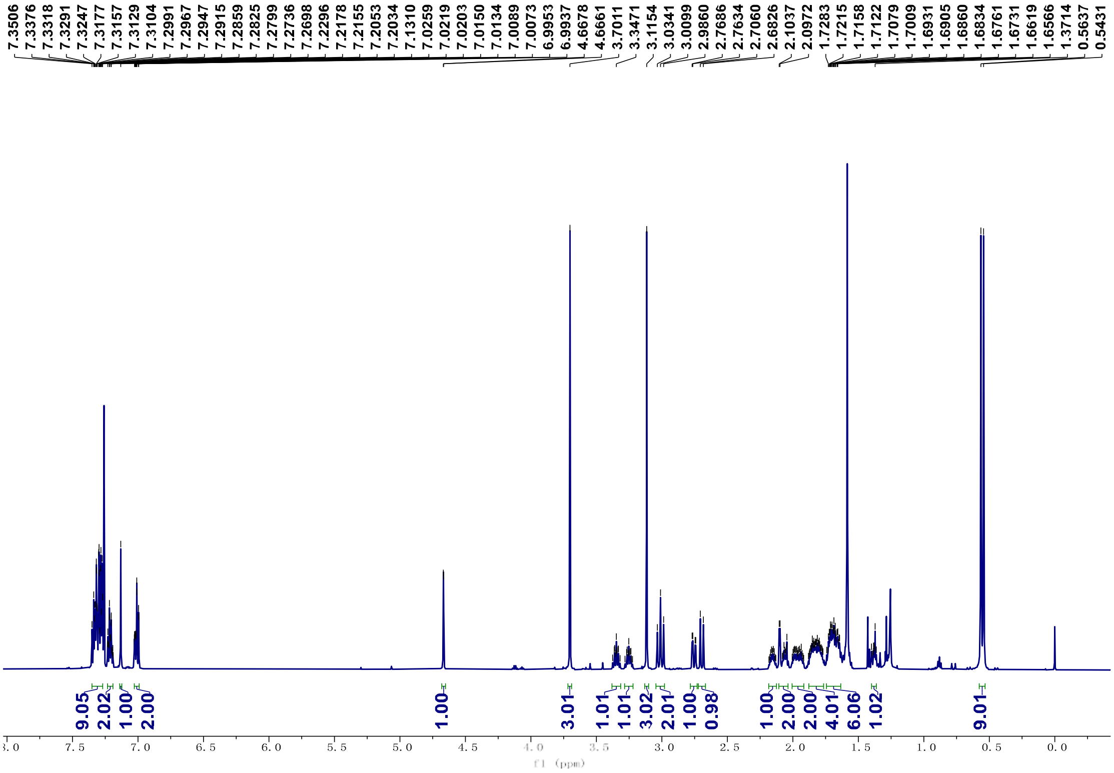

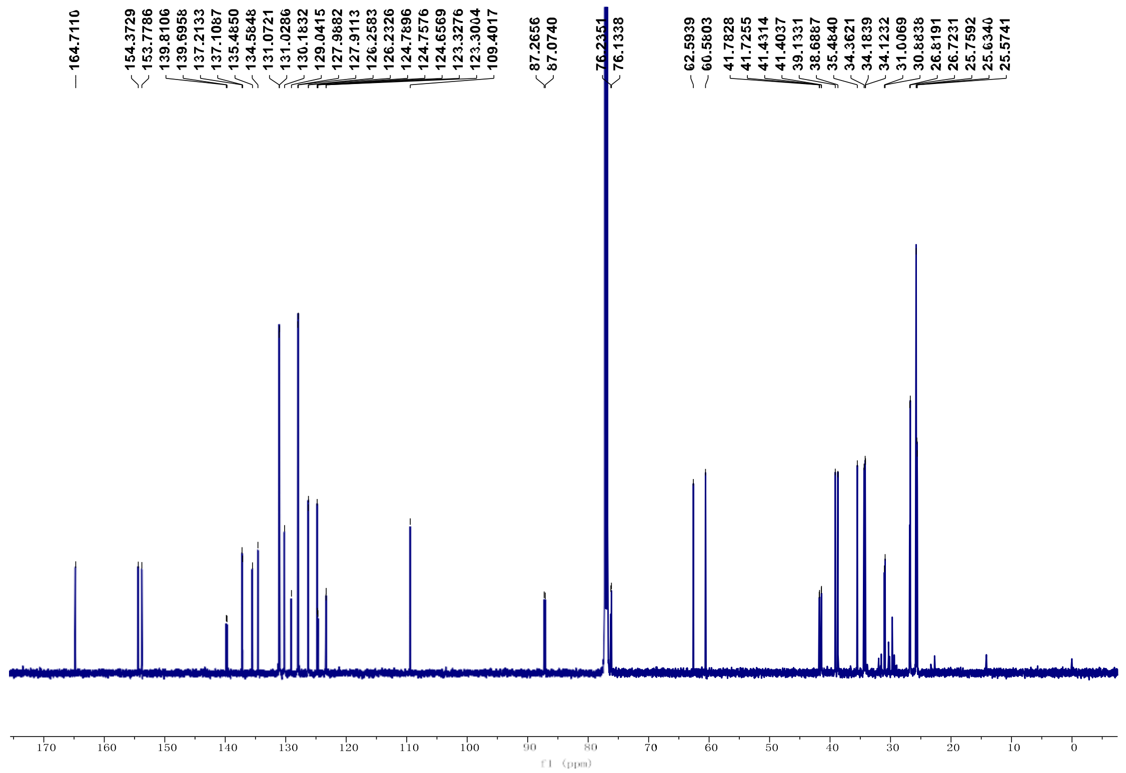
164.7110
154.3729
153.7786
139.8106
139.6958
137.2133
137.1087
135.4850
134.5848
131.0721
131.0286
130.1832
129.0415
127.9882
127.9113
126.2583
126.2326
124.7896
124.7576
124.6569
123.3276
123.3004
109.4017
87.2656
87.0740
76.1338
62.5939
60.5803
41.7828
41.7255
41.4314
41.4037
39.1331
38.6887
35.4840
34.3621
34.1839
34.1232
31.0069
30.8838
26.8191
26.7231
25.7592
25.5741
f1 (ppm)

4. Evaluation of chiral monophosphorus ligands and palladium halide precursors

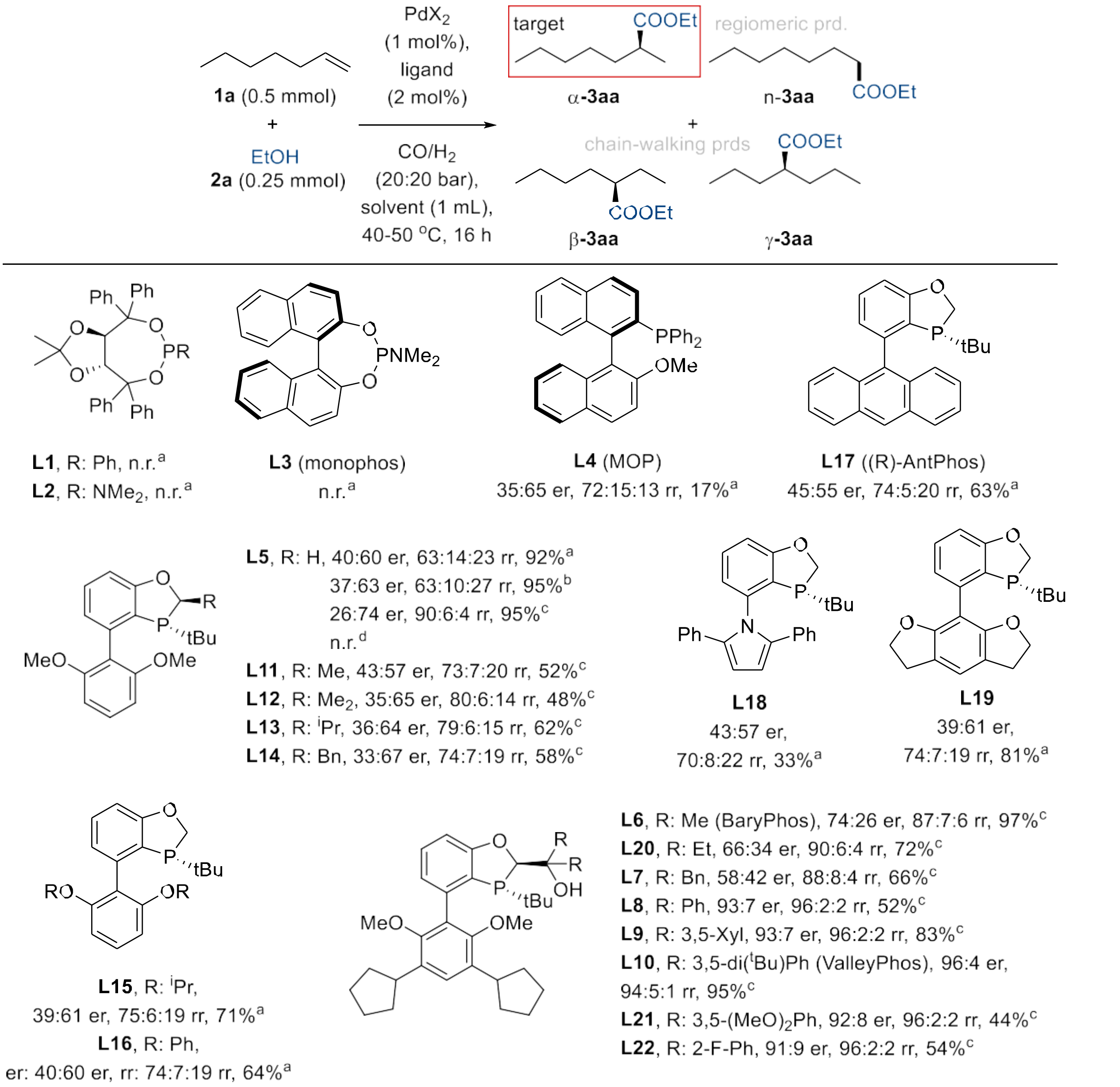

Fig. S1. Evaluation of chiral monophosphorus ligands in the palladium-catalysed alkoxycarbonylation of 1-octene with ethanol.

Regioisomeric ratio (rr: α:n:β+γ), enantiomeric ratio (er: S/R-α), and yield determined by the GC-FID and NMR analysis of the reaction mixture.

Conditions:

[a] **1a** (0.50 mmol), **2a** (0.25 mmol), PdI_2 (1 mol%), ligand (2 mol%), CO/H_2 (20/20 bar), 1,4-dioxane (1 mL), 50 °C, 16 h.

[b] **1a** (0.50 mmol), **2a** (0.25 mmol), PdI_2 (1 mol%), ligand (2 mol%), CO/H_2 (20/20 bar), MTBE (1 mL), 40 °C, 16 h.

[c] **1a** (0.50 mmol), **2a** (0.25 mmol), $PdBr_2(ACN)_2$ (1 mol%), ligand (2 mol%), CO/H_2 (20/20 bar), MTBE (1 mL), 40 °C, 16 h.

[d] **1a** (0.50 mmol), **2a** (0.25 mmol), $PdCl_2(ACN)_2$ (1 mol%), ligand (2 mol%), CO/H_2 (20/20 bar), MTBE (1 mL), 40 °C, 16 h.

5. General procedure for the palladium-catalysed α-branched regioselective and enantioselective carbonylation of unactivated alkenes

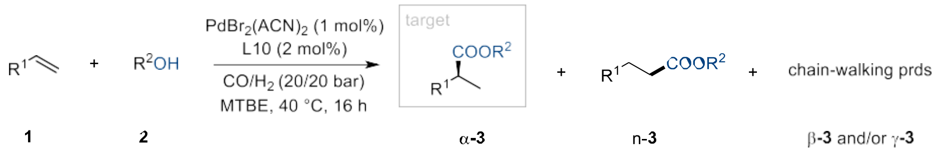

CAUTION: Syngas and CO are highly TOXIC and flammable. Therefore, all manipulations with syngas and CO should be performed with great care in a well-ventilated fume hood. The use of a personal detector of CO is highly advised.

Inside a nitrogen-filled glovebox, a 4 mL vial was charged with a Teflon-coated magnetic stirring bar, $PdBr_2(ACN)_2$ (0.9 mg, 0.0025 mmol), **L10** (4.4 mg, 0.0050 mmol), and MTBE (1 mL). The vial was closed with a cap and allowed to stir overnight, or until a clear solution was obtained (a stock solution of the $PdBr_2(ACN)_2$ and **L10** in MTBE could be prepared in advance and stored in a glovebox for at least 5 days with no noticeable change in the performance of the complex in the catalytic experiments). Then, an alkene (0.50 mmol) and an alcohol (0.25 mmol) were added to the vial. The vial was closed with a cap containing a Teflon-coated septum, which was then punctured with a thick-short needle (the tip of the needle should not be in contact with the reaction mixture). The charged vial was placed into a 300-mL stainless steel Parr autoclave equipped with a heating jacket and an internal thermocouple. The autoclave was sealed before being transferred out of the glovebox. The autoclave was subsequently purged three times with carbon monoxide (~20 bar) and then pressurised with carbon monoxide (20 bar) and dihydrogen (20 bar). The reaction mixture was allowed to stir at 40 °C for 16 h. Upon cooling to room temperature, the pressure was released, the autoclave was opened, and a solution of mesitylene in MTBE as an internal standard was added to the reaction mixture. The spectroscopic yield of the reaction was determined by the NMR spectroscopy. The regioselectivity was determined based on the GC-FID analysis on an achiral stationary phase. The enantioselectivity was determined by the chiral GC-FID analysis on a chiral stationary phase. The reaction mixture was concentrated under reduced pressure. The residue was subjected to column chromatography on silica gel, typically using a mixture of petroleum ether/dichloromethane (8/2) as an eluent, to afford the target ester product. The enantioenrichment of the isolated material was re-determined by the chiral GC-FID analysis on a chiral stationary phase, indicating no noticeable change in the enantioselectivity during the product isolation.

Supplementary Notes

Note 1. The order of gas addition is critical for reproducibility. Sequential addition of CO and H_2 is effective; however, when available, premixed syngas (CO/H_2 1:1) is preferable and can be used directly, simplifying the setup.

Note 2. Preparing a stock solution of $PdBr_2(ACN)_2$ and **L10** in MTBE and stirring it overnight improves reproducibility. Reactions are also effective after shorter stirring times (~1 h), but conversion may be lower.

Note 3. In some cases, longer reaction times (up to 48 h) or increased catalyst loading (or both) are required to achieve near-complete conversion. Further extension beyond 48 h generally has negligible effect.

Note 4. The reaction is sensitive to temperature. Catalytic activity is low below 40 °C, while enantioselectivity decreases above 50 °C. Accurate internal temperature control of the reaction mixture inside of the autoclave is therefore essential (rather than relying on external oil baths or heating mantles).

If low conversion is observed at 40 ^{o}C, raising the temperature to 45–50 ^{o}C may be beneficial. Note that some autoclave heating mantles overshoot during initial heating; proper calibration should be verified if results are not reproducible.
Note 5. If any starting material is insoluble in MTBE at the reaction temperature, 1,4-dioxane can be used as an alternative solvent.

For the development of the GC-FID methods to determine the enantioenrichment of the products, the racemic products were prepared in analogous experiments using rac-BIDIME (0.9 mg, 0.00275 mmol) in place of enantiopure **L10** (4.4 mg, 0.0050 mol).

Supplementary procedure for the reduction of chiral esters to chiral alcohols for GC analysis

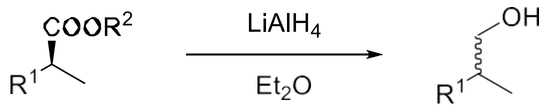

In a 25 mL vial equipped with a stirring bar, $LiAlH_4$ (0.1 mmol, 3.7 mg) was added to dry diethyl ether (3 mL). Subsequently, an ester solution (~5 mg in 1 mL of diethyl ether) was added to the suspension at room temperature. The mixture was allowed to stir for 1 hour at room temperature. The mixture was then sequentially treated with an aqueous solution of NaOH (1 M, 1 mL) and water (1 mL) and then allowed to stir for 5 min. Diethyl ether (3 mL) was added. The organic layer was separated, dried over $MgSO_4$, and filtered. The volatiles from the filtrate were removed under reduced pressure, affording the corresponding alcohol, as confirmed by the GC-MS analysis. The material was subjected to the GC-FID analysis to determine enantioselectivity without further purification.

Reduction of enantioenriched ethyl (S)-2-methyloctanoate – the GC traces of the starting material and the product

The reduction of enantioenriched ethyl *(S)*-2-methyloctanoate **3aa** (97:3 er) resulted in enantioenriched *(S)*-2-methyloctan-1-ol (96:4), indicating the retention of the enantioenrichment. Corresponding GC chromatograms for racemic and enantioenriched esters and alcohols are shown below:

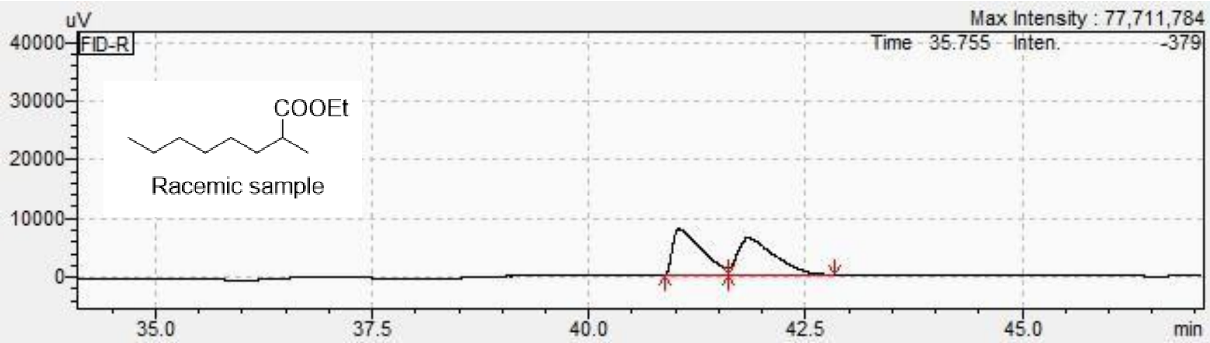

Peak#	Ret. Time	Area	Conc.	Area%
1	41.035	182305	49.375	49.375
2	41.825	186921	50.625	50.625
Total		369226	100.000	100.000

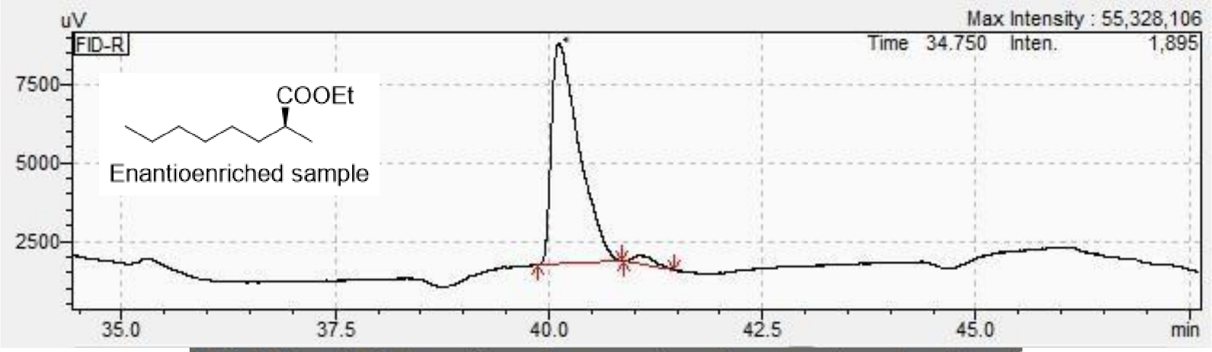

Peak#	Ret. Time	Area	Conc.	Area%
1	40.120	158843	96.859	96.859
2	41.059	5151	3.141	3.141
Total		163995	100.000	100.000

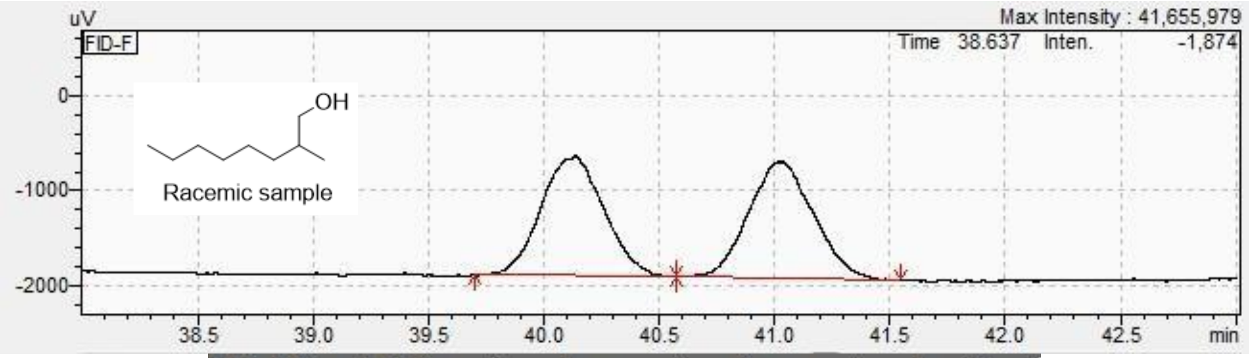

Peak#	Ret. Time	Area	Conc.	Area%
1	40.138	23848	49.958	49.958
2	41.034	23888	50.042	50.042
Total		47736	100.000	100.000

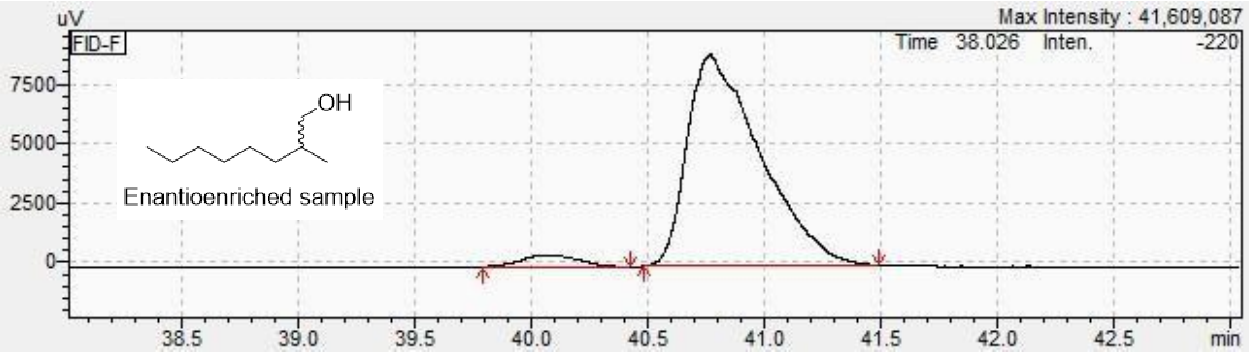

Peak#	Ret. Time	Area	Conc.	Area%
1	40.071	8438	4.171	4.171
2	40.772	193869	95.829	95.829
Total		202307	100.000	100.000

6. Characterisation of products

Ethyl (S)-2-methyloctanoate (3aa):

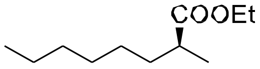

The compound was prepared according to the general procedure by reaction of 1-octene (79 µL, 0.50 mmol) and ethanol (15 µL, 0.25 mmol), and isolated by column chromatography (silica gel, petroleum ether/dichloromethane = 9/1), yielding the title product as a colourless oil (42 mg, 90%). The NMR data match those reported in the literature for a racemic product.[3]

^{1}H NMR (500 MHz, $CDCl_3$) δ 4.12 (q, *J* = 7.1 Hz, 2H), 2.40 (h, *J* = 7.0 Hz, 1H), 1.71 – 1.59 (m, 1H), 1.45 – 1.35 (m, 1H), 1.31 – 1.22 (m, 11H), 1.13 (d, *J* = 7.0 Hz, 3H), 0.88 (t, *J* = 6.7 Hz, 3H).

^{13}C-{^{1}H} NMR (126 MHz, $CDCl_3$) δ 177.1, 60.2, 39.7, 34.0, 31.9, 29.3, 27.3, 22.7, 17.2, 14.4, 14.2.

96:4 er, $[\alpha]_D^{25}$ = +3.7 (c = 2.0, $CHCl_3$).

GC analysis of the ester: (CP-Chirasil-Dex CB Column 25 m x 0.25 mm x 0.25 µm, flow: 2.84 mL/min, 90 °C): t_1 = 40.12 min (major), t_2 = 41.06 min (minor). For GC traces of racemic and enantioenriched samples, see below.

For the absolute configuration determination, the isolated ester was reduced to the alcohol according to the procedure in section 5. GC analysis (CycloSil-B Column 25 m x 0.25 mm x 0.25 µm, flow: 3.16 mL/min, 90 °C): t_1 = 40.07 min (minor), t_2 = 40.77 min (major). The same major isomer of the alcohol was formed upon the reduction of (*S*)-α-**3ax**, for which the absolute configuration was assigned unambiguously by the X-ray crystallography, enabling the assignment of the absolute configuration of **3aa** as (*S*)-enantiomer.

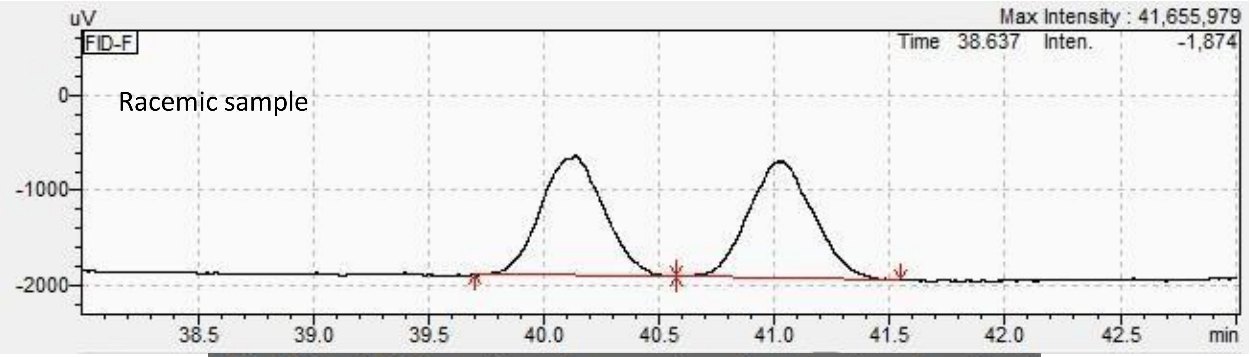

Peak#	Ret. Time	Area	Conc.	Area%
1	40.138	23848	49.958	49.958
2	41.034	23888	50.042	50.042
Total		47736	100.000	100.000

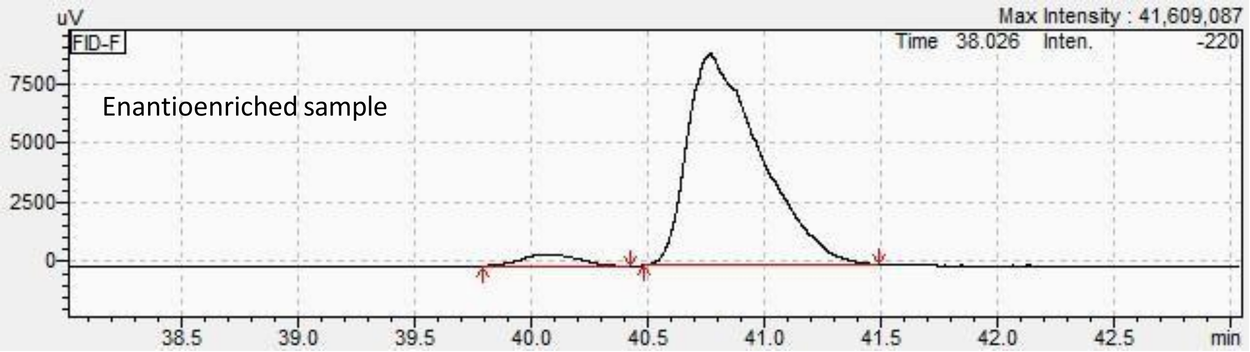

Peak#	Ret. Time	Area	Conc.	Area%
1	40.071	8438	4.171	4.171
2	40.772	193869	95.829	95.829
Total		202307	100.000	100.000

Methyl (S)-2-methyloctanoate (3ab):

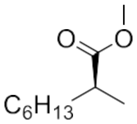

The compound was prepared according to the general procedure by reaction of 1-octene (79 µL, 0.50 mmol) and methanol (10 µL, 0.25 mmol), and was isolated by column chromatography (silica gel, petroleum ether/dichloromethane = 9/1), yielding the title product as a colourless oil (35 mg, 81%). The NMR data match those reported in the literature for the racemic product.[3]

^{1}H NMR (500 MHz, $CDCl_3$) δ 3.67 (s, 3H), 2.43 (h, 1H), 1.71 – 1.60 (m, 1H), 1.43 – 1.35 (m, 1H), 1.28 – 1.24 (m, 8H), 1.14 (d, *J* = 7.0 Hz, 3H), 0.88 (t, *J* = 6.9 Hz, 3H).

^{13}C-{^{1}H} NMR (126 MHz, $CDCl_3$) δ 177.6, 51.6, 39.6, 34.0, 31.8, 29.3, 27.4, 22.7, 17.2, 14.2.

96:4 er, $[\alpha]_D^{25}$ = +2.0 (c = 1.1, $CHCl_3$).

To determine the enantiomeric ratio, the isolated ester was reduced to the alcohol according to the procedure in section 5. GC analysis (CycloSil-B Column 25 m x 0.25 mm x 0.25 µm, flow: 3.16 mL/min, 90 °C): t_1 = 40.11 min (minor), t_2 = 40.90 min (major). The same major isomer of the alcohol was formed upon the reduction of (*S*)-α-**3ax**, for which the absolute configuration was assigned unambiguously by the X-ray crystallography, enabling the assignment of the absolute configuration of **3ab** as (*S*)-enantiomer.

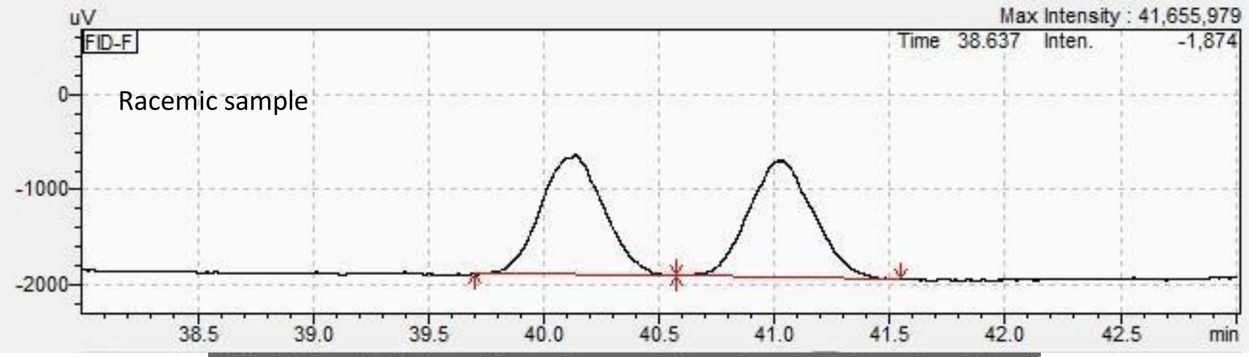

Peak#	Ret. Time	Area	Conc.	Area%
1	40.138	23848	49.958	49.958
2	41.034	23888	50.042	50.042
Total		47736	100.000	100.000

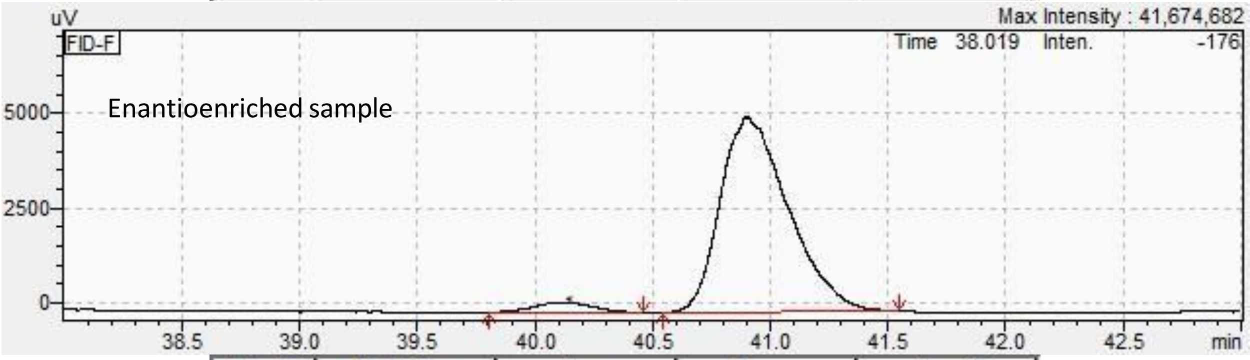

Peak#	Ret. Time	Area	Conc.	Area%
1	40.112	4614	4.271	4.271
2	40.904	103435	95.729	95.729
Total		108049	100.000	100.000

Propyl (S)-2-methyloctanoate (3ac):

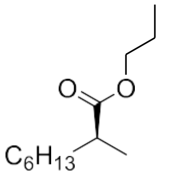

The compound was prepared according to the general procedure by reaction of 1-octene (79 µL, 0.50 mmol) and n-propanol (19 µL, 0.25 mmol), and was isolated by column chromatography (silica gel, petroleum ether/dichloromethane = 9/1), yielding the title product as a colourless oil (43 mg, 86%). The NMR data match those reported in the literature for the racemic product.[3]

^{1}H NMR (500 MHz, $CDCl_3$) δ 4.03 (td, *J* = 6.7, 1.6 Hz, 2H), 2.42 (h, *J* = 7.0 Hz, 1H), 1.65 (h, *J* = 7.0 Hz, 3H), 1.45 – 1.34 (m, 1H), 1.33 – 1.21 (m, 8H), 1.14 (d, *J* = 7.0 Hz, 3H), 0.94 (t, *J* = 7.4 Hz, 3H), 0.87 (t, *J* = 6.8 Hz, 3H).

^{13}C-{^{1}H} NMR (126 MHz, $CDCl_3$) δ 177.2, 65.9, 39.8, 34.0, 31.9, 29.3, 27.4, 22.7, 22.2, 17.3, 14.2, 10.6.

96:4 er, $[\alpha]_D^{25}$ = +3.1 (c = 1.1, $CHCl_3$).

To determine the enantiomeric ratio, the isolated ester was reduced to the alcohol according to the procedure in section 5. GC analysis (CycloSil-B Column 25 m x 0.25 mm x 0.25 µm, flow: 3.16 mL/min, 90 °C): t_1 = 40.01 min (minor), t_2 = 40.77 min (major). The same major isomer of the alcohol was formed upon the reduction of (*S*)-α-**3ax**, for which the absolute configuration was assigned unambiguously by the X-ray crystallography, enabling the assignment of the absolute configuration of **3ac** as (*S*)-enantiomer.

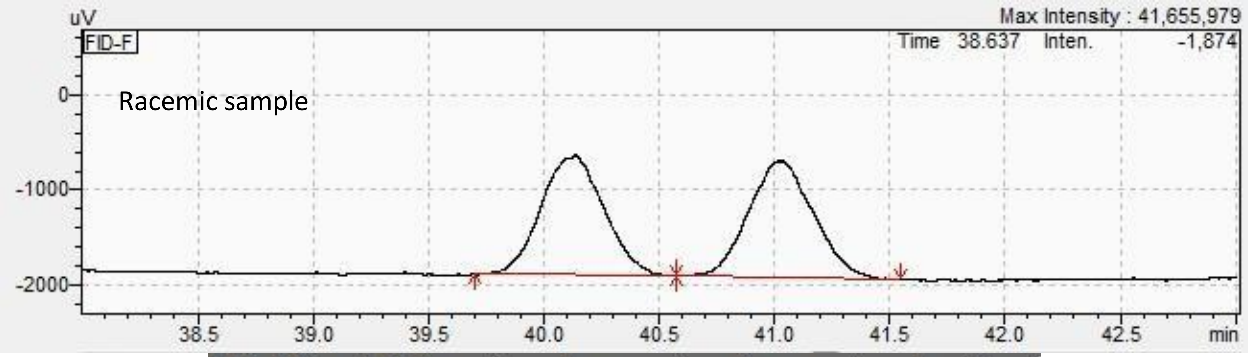

Peak#	Ret. Time	Area	Conc.	Area%
1	40.138	23848	49.958	49.958
2	41.034	23888	50.042	50.042
Total		47736	100.000	100.000

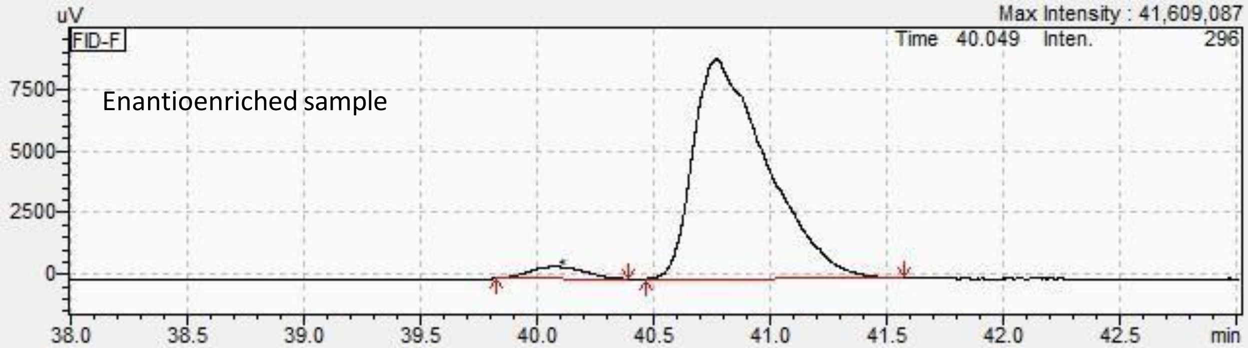

Peak#	Ret. Time	Area	Conc.	Area%
1	40.071	7942	3.911	3.911
2	40.772	195109	96.089	96.089
Total		203051	100.000	100.000

Hexyl (S)-2-methyloctanoate (3ad):

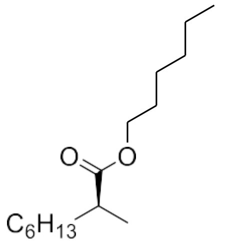

The compound was prepared according to the general procedure by reaction of 1-octene (79 µL, 0.50 mmol) and n-hexanol (31 µL, 0.25 mmol), and was isolated by column chromatography (silica gel, petroleum ether/dichloromethane = 9/1), yielding the title product as a colourless oil (53 mg, 87%). The NMR data match those reported in the literature for the racemic product.[3]

^{1}H NMR (500 MHz, $CDCl_3$) δ 4.06 (td, *J* = 6.7, 2.6 Hz, 2H), 2.41 (h, *J* = 7.0 Hz, 1H), 1.69 – 1.57 (m, 3H), 1.46 – 1.23 (m, 15H), 1.13 (d, *J* = 7.0 Hz, 3H), 0.91 – 0.85 (m, 6H).

^{13}C-{^{1}H} NMR (126 MHz, $CDCl_3$) δ 177.2, 64.4, 39.8, 34.0, 31.9, 31.6, 29.4, 28.8, 27.4, 25.8, 22.8, 22.7, 17.3, 14.2, 14.1.

96:4 er, $[\alpha]_D^{25}$ = +2.7 (c = 1.2, $CHCl_3$).

To determine the enantiomeric ratio, the isolated ester was reduced to the alcohol according to the procedure in section 5. GC analysis (CycloSil-B Column 25 m x 0.25 mm x 0.25 µm, flow: 3.16 mL/min, 90 °C): t_1 = 40.14 min (minor), t_2 = 40.90 min (major). The same major isomer of the alcohol was formed upon the reduction of (*S*)-α-**3ax**, for which the absolute configuration was assigned unambiguously by the X-ray crystallography, enabling the assignment of the absolute configuration of **3ad** as (*S*)-enantiomer.

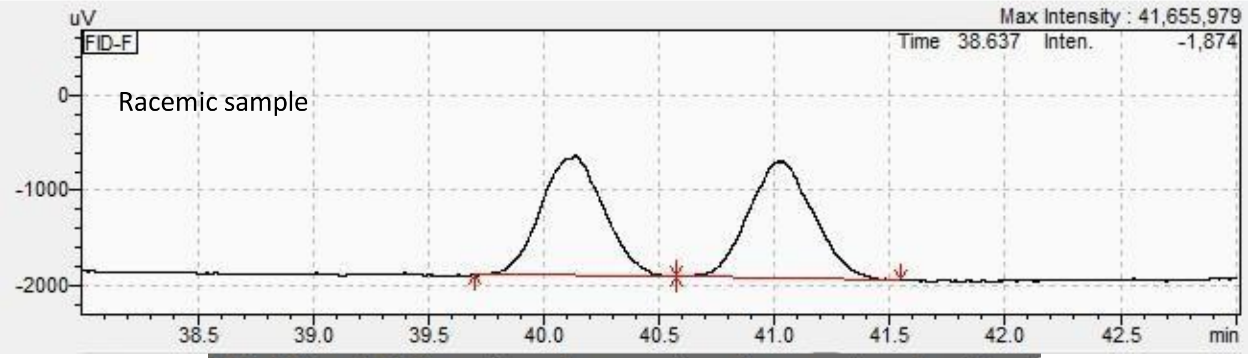

Peak#	Ret. Time	Area	Conc.	Area%
1	40.138	23848	49.958	49.958
2	41.034	23888	50.042	50.042
Total		47736	100.000	100.000

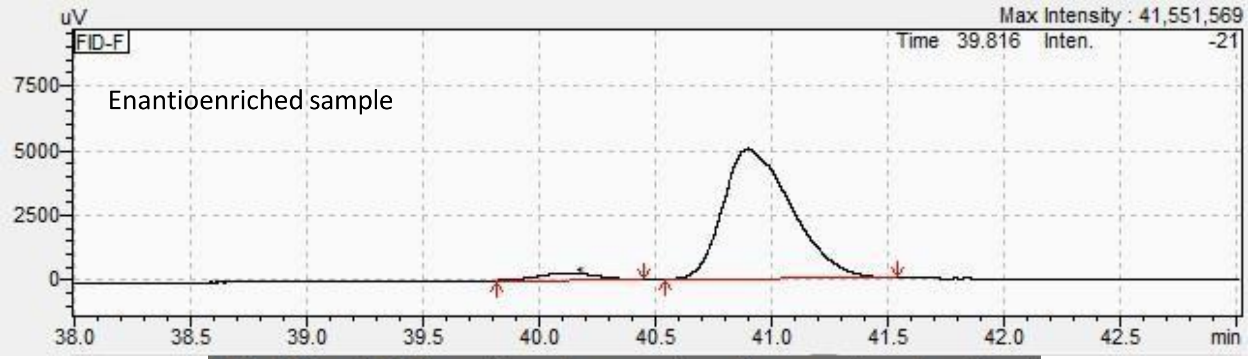

Peak#	Ret. Time	Area	Conc.	Area%
1	40.138	4687	4.341	4.341
2	40.900	103288	95.659	95.659
Total		107975	100.000	100.000

Isopropyl (S)-2-methyloctanoate (3ae):

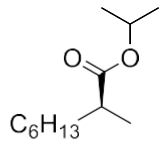

The compound was prepared according to the general procedure by reaction of 1-octene (79 µL, 0.50 mmol) and isopropanol (19 µL, 0.25 mmol), and was isolated by column chromatography (silica gel, petroleum ether/dichloromethane = 9/1), yielding the title product as a colourless oil (45 mg, 90%). The NMR data match those reported in the literature for the racemic product.[3]

^{1}H NMR (500 MHz, CDCl$_3$) δ 5.00 (hept, J = 6.2 Hz, 1H), 2.37 (h, J = 7.0 Hz, 1H), 1.66 – 1.59 (m, 1H), 1.41 – 1.35 (m, 1H), 1.29 – 1.24 (m, 8H), 1.22 (d, J = 6.3 Hz, 6H), 1.12 (d, J = 7.0 Hz, 3H), 0.87 (t, J = 7.1 Hz, 3H).

^{13}C-{^{1}H} NMR (126 MHz, CDCl$_3$) δ 176.7, 67.3, 39.9, 34.0, 31.9, 29.3, 27.3, 22.7, 22.00, 21.96, 17.2, 14.2.

96:4 er, $[\alpha]_D^{25}$ = +1.9 (c = 1.2, CHCl$_3$).

To determine the enantiomeric ratio, the isolated ester was reduced to the alcohol according to the procedure in section 5. GC analysis (CycloSil-B Column 25 m x 0.25 mm x 0.25 µm, flow: 3.16 mL/min, 90 °C): t_1 = 40.12 min (minor), t_2 = 40.90 min (major). The same major isomer of the alcohol was formed upon the reduction of (*S*)-α-**3ax**, for which the absolute configuration was assigned unambiguously by the X-ray crystallography, enabling the assignment of the absolute configuration of **3ae** as (*S*)-enantiomer.

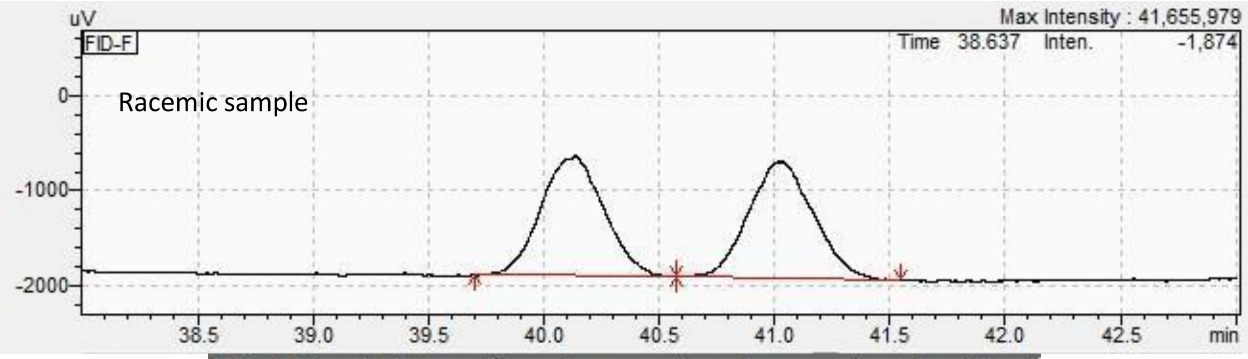

Peak#	Ret. Time	Area	Conc.	Area%
1	40.138	23848	49.958	49.958
2	41.034	23888	50.042	50.042
Total		47736	100.000	100.000

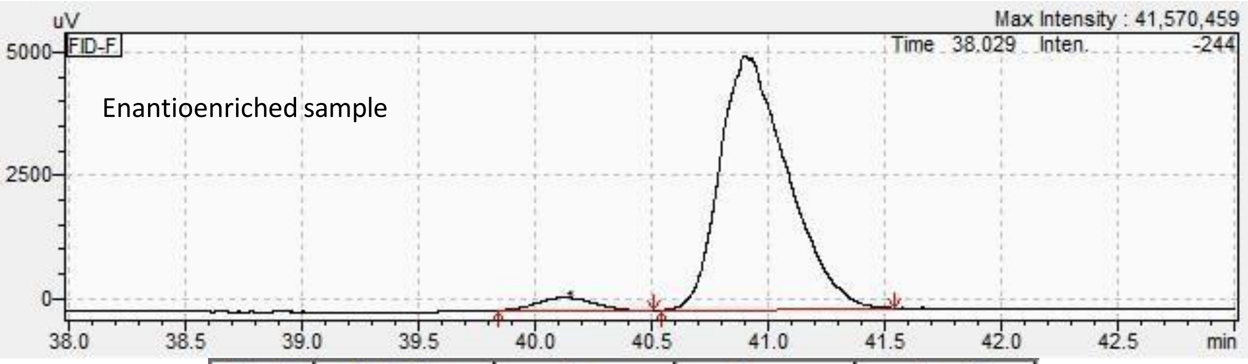

Peak#	Ret. Time	Area	Conc.	Area%
1	40.116	4534	4.210	4.210
2	40.899	103156	95.790	95.790
Total		107690	100.000	100.000

Cyclohexyl (S)-2-methyloctanoate (3af):

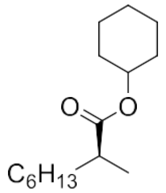

The compound was prepared according to the general procedure by reaction of 1-octene (79 µL, 0.50 mmol) and cyclohexanol (26 µL, 0.25 mmol), and was isolated by column chromatography (silica gel, petroleum ether/dichloromethane = 9/1), yielding the title product as a colourless oil (52 mg, 86%). The NMR data match those reported in the literature for the racemic product.[3]

^{1}H NMR (500 MHz, CDCl$_3$) δ 4.80 – 4.73 (m, 1H), 2.39 (h, *J* = 6.9 Hz, 1H), 1.86 – 1.78 (m, 2H), 1.75 – 1.68 (m, 2H), 1.67 – 1.59 (m, 1H), 1.57 – 1.33 (m, 7H), 1.29 – 1.25 (m, 8H), 1.13 (d, *J* = 7.0 Hz, 3H), 0.88 (t, *J* = 6.9 Hz, 3H).

^{13}C-{^{1}H} NMR (126 MHz, CDCl$_3$) δ 176.6, 72.1, 40.0, 34.1, 31.9, 31.8, 31.7, 29.4, 27.3, 25.6, 23.8, 22.7, 17.3, 14.2.

96:4 er, $[\alpha]_D^{25}$ = +5.2 (c = 1.3, CHCl$_3$).

To determine the enantiomeric ratio, the isolated ester was reduced to the alcohol according to the procedure in section 5. GC analysis (CycloSil-B Column 25 m x 0.25 mm x 0.25 µm, flow: 3.16 mL/min, 90 °C): t_1 = 40.08 min (minor), t_2 = 40.77 min (major). The same major isomer of the alcohol was formed upon the reduction of (*S*)-α-**3ax**, for which the absolute configuration was assigned unambiguously by the X-ray crystallography, enabling the assignment of the absolute configuration of **3af** as (*S*)-enantiomer.

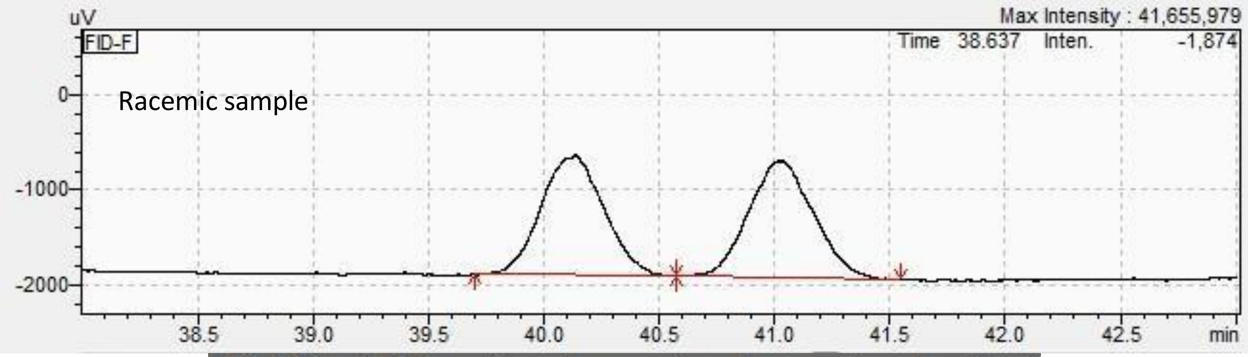

Peak#	Ret. Time	Area	Conc.	Area%
1	40.138	23848	49.958	49.958
2	41.034	23888	50.042	50.042
Total		47736	100.000	100.000

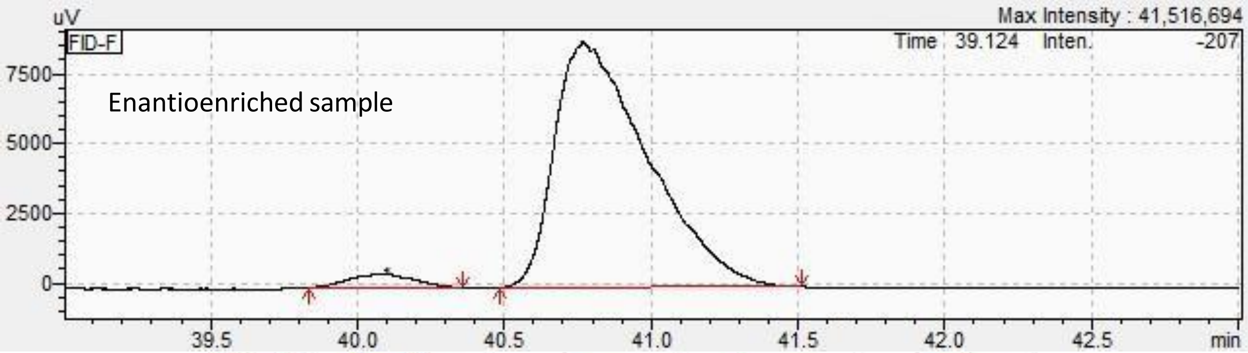

Peak#	Ret. Time	Area	Conc.	Area%
1	40.076	7749	3.841	3.841
2	40.769	193969	96.159	96.159
Total		201717	100.000	100.000

***tert*-Butyl (S)-2-methyloctanoate (3ag):**

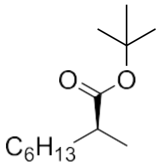

The compound was prepared according to the general procedure by reaction of 1-octene (79 µL, 0.50 mmol) and tert-butanol (23 µL, 0.25 mmol), and was isolated by column chromatography (silica gel, petroleum ether/dichloromethane = 9/1), yielding the title product as a colourless oil (12 mg, 22%). The NMR data match those reported in the literature for the racemic product.[3]

^{1}H NMR (500 MHz, $CDCl_3$) δ 2.30 (h, *J* = 7.0 Hz, 1H), 1.63 – 1.56 (m, 1H), 1.44 (s, 9H), 1.40 – 1.33 (m, 1H), 1.29 – 1.25 (m, 8H), 1.09 (d, *J* = 7.0 Hz, 3H), 0.87 (t, *J* = 7.1 Hz, 3H).

^{13}C-{^{1}H} NMR (126 MHz, $CDCl_3$) δ 176.6, 79.8, 40.6, 34.1, 31.9, 29.4, 28.3, 27.3, 22.8, 17.3, 14.2.

97:3 er, $[\alpha]_D^{25}$ = +2.7 (c = 1.0, $CHCl_3$).

To determine the enantiomeric ratio, the isolated ester was reduced to the alcohol according to the procedure in section 5. GC analysis (CycloSil-B Column 25 m x 0.25 mm x 0.25 µm, flow: 3.16 mL/min, 90 °C): t_1 = 40.10 min (minor), t_2 = 40.94 min (major). The same major isomer of the alcohol was formed upon the reduction of (*S*)-α-**3ax**, for which the absolute configuration was assigned unambiguously by the X-ray crystallography, enabling the assignment of the absolute configuration of **3ag** as (*S*)-enantiomer.

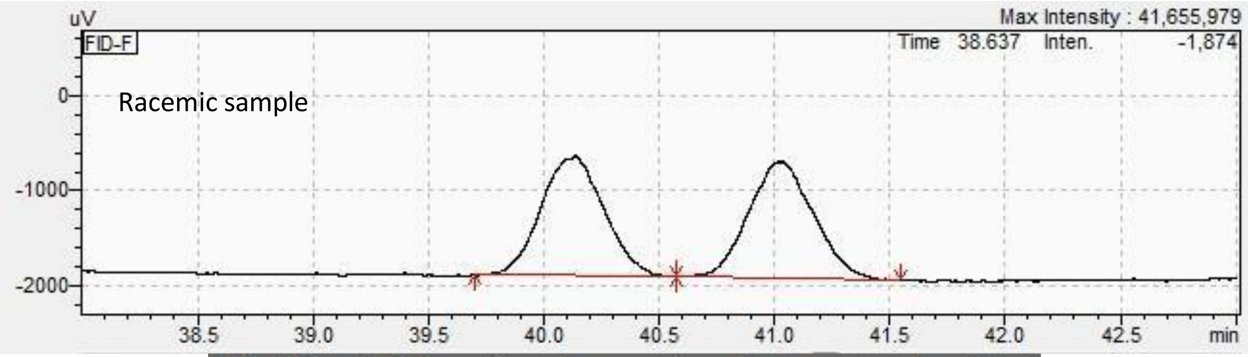

Peak#	Ret. Time	Area	Conc.	Area%
1	40.138	23848	49.958	49.958
2	41.034	23888	50.042	50.042
Total		47736	100.000	100.000

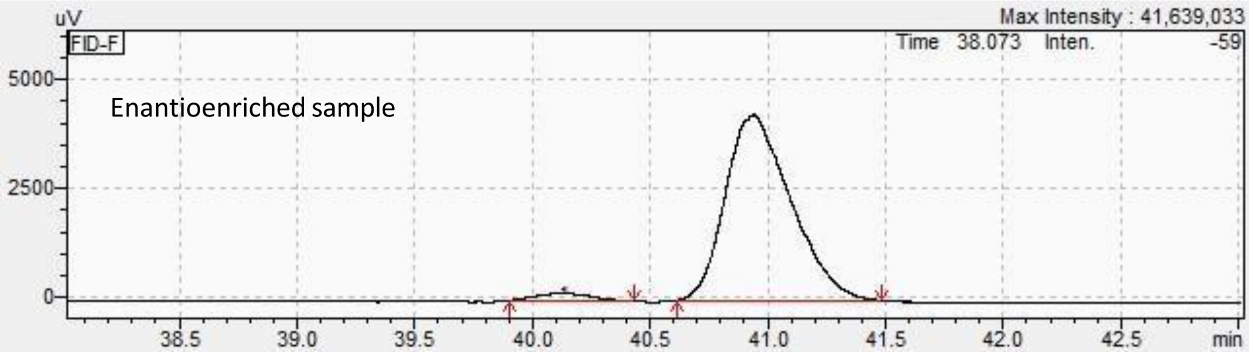

Peak#	Ret. Time	Area	Conc.	Area%
1	40.104	2913	3.413	3.413
2	40.941	82446	96.587	96.587
Total		85359	100.000	100.000

2-Cyclohexylethyl (S)-2-methyloctanoate (3ah):

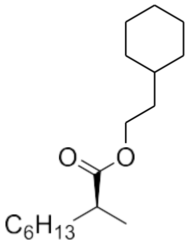

The compound was prepared according to the general procedure by reaction of 1-octene (79 µL, 0.50 mmol) and 2-cyclohexylethanol (36 µL, 0.25 mmol), and was isolated by column chromatography (silica gel, petroleum ether/dichloromethane = 8/2), yielding the title product as a colourless oil (58 mg, 86%).

^{1}H NMR (500 MHz, CDCl$_3$) δ 4.14 – 4.05 (m, 2H), 2.41 (h, *J* = 7.0 Hz, 1H), 1.76 – 1.59 (m, 6H), 1.51 (q, *J* = 6.8 Hz, 2H), 1.43 – 1.32 (m, 2H), 1.30 – 1.16 (m, 11H), 1.13 (d, *J* = 7.0 Hz, 3H), 0.97 – 0.90 (m, 2H), 0.88 (t, *J* = 6.9 Hz, 3H).

^{13}C-{^{1}H} NMR (126 MHz, CDCl$_3$) δ 177.2, 62.5, 39.8, 36.2, 34.7, 34.0, 33.3, 33.3, 31.9, 29.4, 27.4, 26.6, 26.4, 22.8, 17.3, 14.2.

HRMS (APPI) m/z calcd. For $C_{17}H_{33}O_2$ ([M+H]$^+$): 269.2481; found: 269.2481.

96:4 er, $[\alpha]_D^{25}$ = +1.9 (c = 1.4, CHCl$_3$).

To determine the enantiomeric ratio, the isolated ester was reduced to the alcohol according to the procedure in section 5. GC analysis (CycloSil-B Column 25 m x 0.25 mm x 0.25 µm, flow: 3.16 mL/min, 90 °C): t_1 = 40.15 min (minor), t_2 = 40.92 min (major). The same major isomer of the alcohol was formed upon the reduction of (*S*)-α-**3ax**, for which the absolute configuration was assigned unambiguously by the X-ray crystallography, enabling the assignment of the absolute configuration of **3ah** as (*S*)-enantiomer.

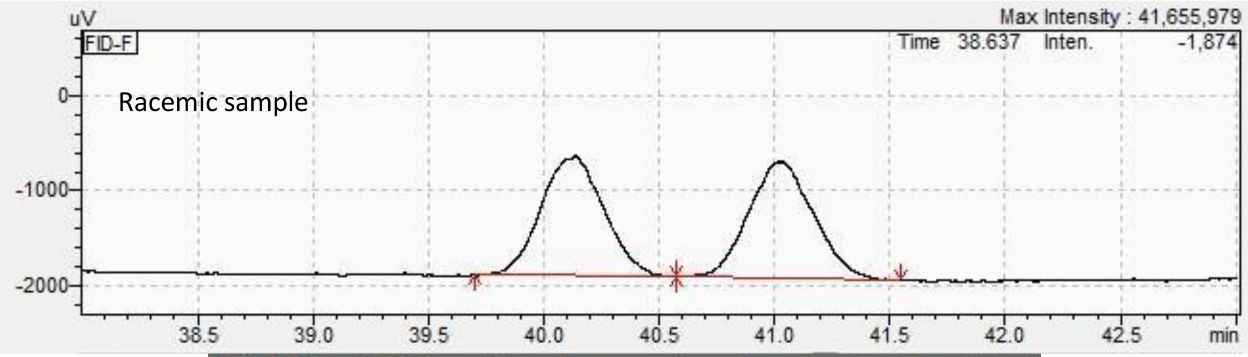

Peak#	Ret. Time	Area	Conc.	Area%
1	40.138	23848	49.958	49.958
2	41.034	23888	50.042	50.042
Total		47736	100.000	100.000

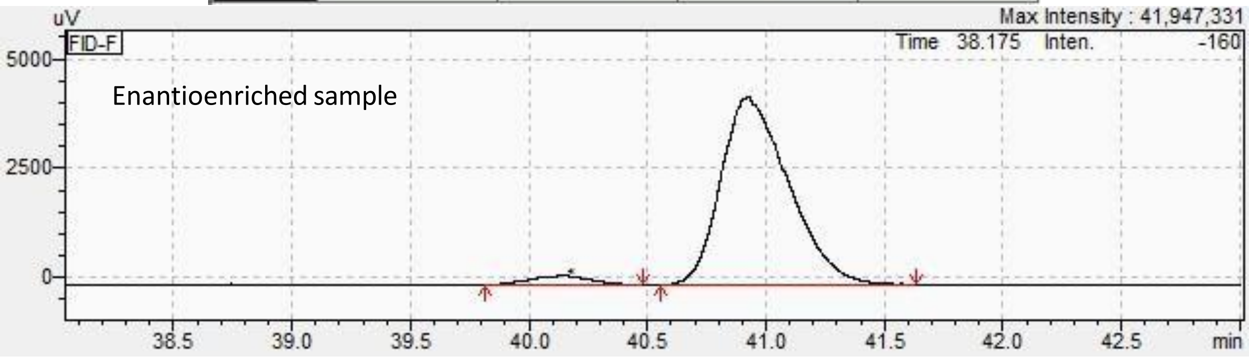

Peak#	Ret. Time	Area	Conc.	Area%
1	40.145	3618	4.071	4.071
2	40.924	85257	95.929	95.929
Total		88875	100.000	100.000

3-Phenylpropyl (S)-2-methyloctanoate (3ai):

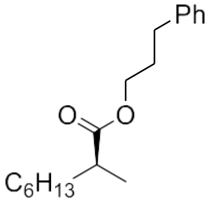

The compound was prepared according to the general procedure by reaction of 1-octene (79 µL, 0.50 mmol) and 3-phenylpropan-1-ol (34 µL, 0.25 mmol), and was isolated by column chromatography (silica gel, petroleum ether/dichloromethane = 8/2), yielding the title product as a colourless oil (62 mg, 90%).

^{1}H NMR (500 MHz, CDCl$_3$) δ 7.31 – 7.26 (m, 2H), 7.22 – 7.15 (m, 3H), 4.09 (td, *J* = 6.5, 1.6 Hz, 2H), 2.72 – 2.66 (m, 2H), 2.44 (h, *J* = 7.0 Hz, 1H), 2.00 – 1.91 (m, 2H), 1.69 – 1.62 (m, 1H), 1.45 – 1.38 (m, 1H), 1.30 – 1.25 (m, 8H), 1.15 (d, *J* = 7.0 Hz, 3H), 0.88 (t, *J* = 7.1 Hz, 3H).

^{13}C-{^{1}H} NMR (126 MHz, CDCl$_3$) δ 177.1, 141.4, 128.6, 128.6, 126.1, 63.6, 39.8, 34.0, 32.3, 31.9, 30.5, 29.4, 27.4, 22.8, 17.3, 14.2.

HRMS (APPI) m/z calcd. For $C_{18}H_{29}O_2$ ([M+H]$^+$): 277.2168; found: 277.2175.

96:4 er, $[\alpha]_D^{25}$ = +2.2 (c = 1.3, CHCl$_3$).

To determine the enantiomeric ratio, the isolated ester was reduced to the alcohol according to the procedure in section 5. GC analysis (CycloSil-B Column 25 m x 0.25 mm x 0.25 µm, flow: 3.16 mL/min, 90 °C): t_1 = 40.07 min (minor), t_2 = 40.75 min (major). The same major isomer of the alcohol was formed upon the reduction of (*S*)-α-**3ax**, for which the absolute configuration was assigned unambiguously by the X-ray crystallography, enabling the assignment of the absolute configuration of **3ai** as (*S*)-enantiomer.

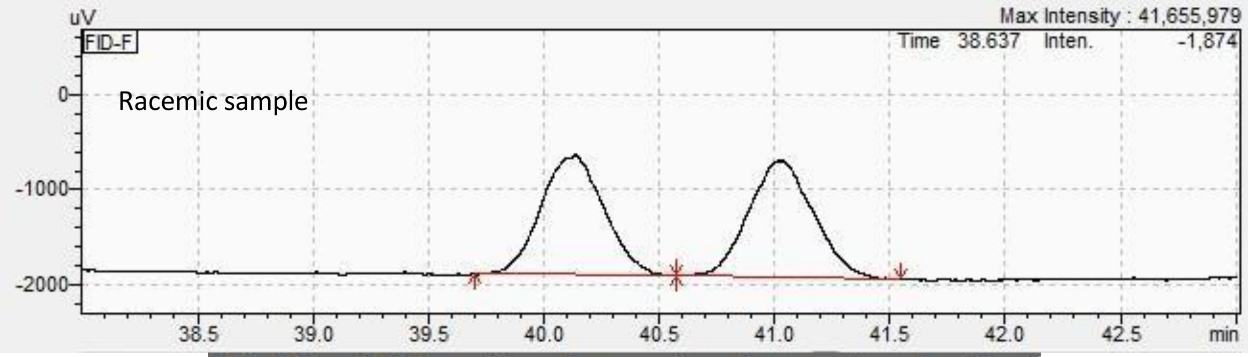

Peak#	Ret. Time	Area	Conc.	Area%
1	40.138	23848	49.958	49.958
2	41.034	23888	50.042	50.042
Total		47736	100.000	100.000

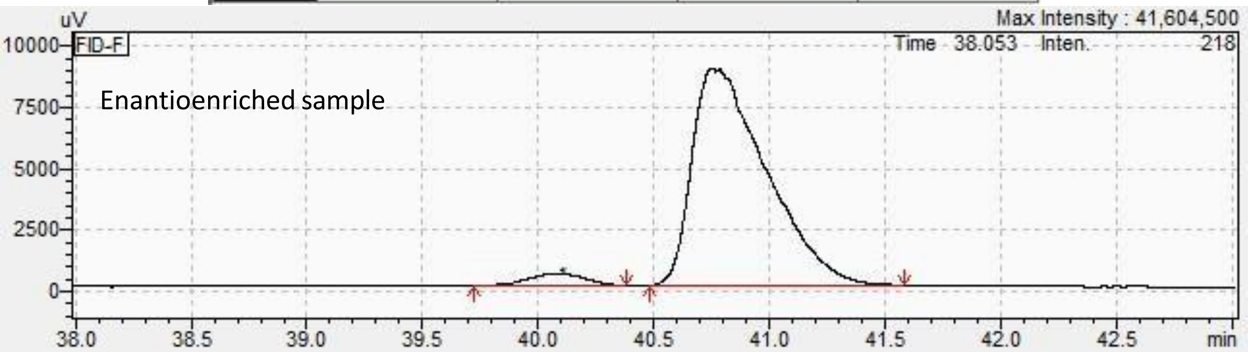

Peak#	Ret. Time	Area	Conc.	Area%
1	40.073	8454	4.119	4.119
2	40.746	196788	95.881	95.881
Total		205242	100.000	100.000

4-Methoxyphenethyl (S)-2-methyloctanoate (3aj):

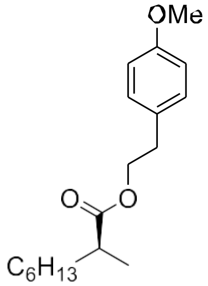

The compound was prepared according to the general procedure by reaction of 1-octene (79 µL, 0.50 mmol) and methyl 3-hydroxypropanoate (26 mg, 0.25 mmol), was isolated by column chromatography (silica gel, petroleum ether/dichloromethane = 8/2), yielding the title product as a colourless oil (58 mg, 80%).

^{1}H NMR (500 MHz, $CDCl_3$) δ 7.14 (d, *J* = 8.6 Hz, 2H), 6.84 (d, *J* = 8.6 Hz, 2H), 4.25 (td, *J* = 7.1, 2.2 Hz, 2H), 3.79 (s, 3H), 2.88 (t, *J* = 7.0 Hz, 2H), 2.40 (h, *J* = 7.0 Hz, 1H), 1.65 – 1.57 (m, 1H), 1.41 – 1.33 (m, 1H), 1.28 – 1.20 (m, 8H), 1.11 (d, *J* = 7.0 Hz, 3H), 0.88 (t, *J* = 7.0 Hz, 3H).

^{13}C-{^{1}H} NMR (126 MHz, $CDCl_3$) δ 177.0, 158.4, 130.1, 130.0, 114.0, 65.0, 55.4, 39.7, 34.4, 33.9, 31.8, 29.3, 27.3, 22.8, 17.2, 14.2.

HRMS (APPI) m/z calcd. For $C_{18}H_{29}O_3$ ($[M+H]^+$): 293.2097; found:293.2100.

96:4 er, $[\alpha]_D^{25}$ = +1.4 (c = 1.1, $CHCl_3$).

To determine the enantiomeric ratio, the isolated ester was reduced to the alcohol according to the procedure in section 5. GC analysis (CycloSil-B Column 25 m x 0.25 mm x 0.25 µm, flow: 3.16 mL/min, 90 °C): t_1 = 40.15 min (minor), t_2 = 40.92 min (major). The same major isomer of the alcohol was formed upon the reduction of (*S*)-α-**3ax**, for which the absolute configuration was assigned unambiguously by the X-ray crystallography, enabling the assignment of the absolute configuration of **3aj** as (*S*)-enantiomer.

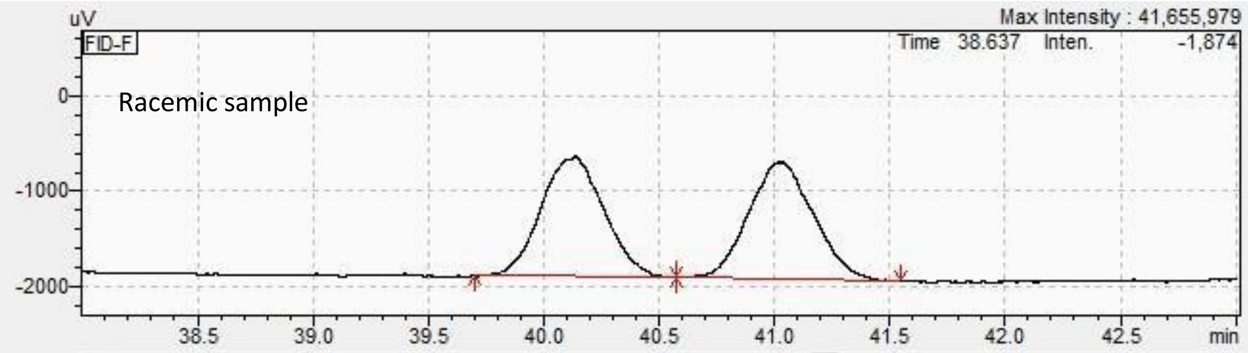

Peak#	Ret. Time	Area	Conc.	Area%
1	40.138	23848	49.958	49.958
2	41.034	23888	50.042	50.042
Total		47736	100.000	100.000

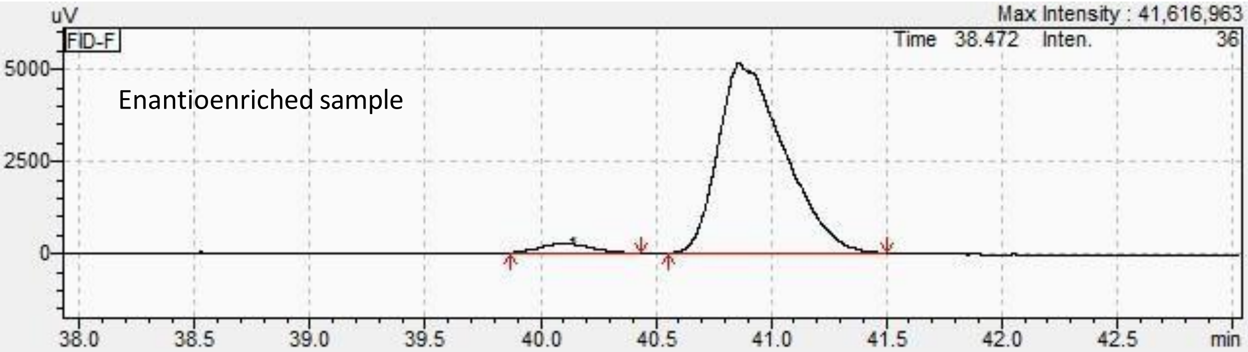

Peak#	Ret. Time	Area	Conc.	Area%
1	40.107	4199	3.910	3.910
2	40.864	103190	96.090	96.090
Total		107389	100.000	100.000

4-(Trifluoromethyl)phenethyl (S)-2-methyloctanoate (3ak):

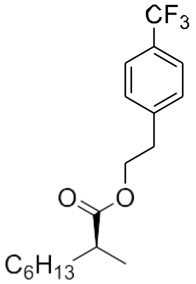

The compound was prepared according to the general procedure by reaction of 1-octene (79 µL, 0.50 mmol) and 2-[4-(trifluoromethyl)phenyl]ethanol (48 mg, 0.25 mmol), and was isolated by column chromatography (silica gel, petroleum ether/dichloromethane = 8/2), yielding the title product as a colourless oil (68 mg, 82%).

^{1}H NMR (500 MHz, CDCl$_3$) δ 7.56 (d, *J* = 8.1 Hz, 2H), 7.34 (d, *J* = 8.0 Hz, 2H), 4.31 (td, *J* = 6.8, 2.5 Hz, 2H), 3.00 (t, *J* = 6.7 Hz, 2H), 2.39 (h, *J* = 7.0 Hz, 1H), 1.62 – 1.56 (m, 1H), 1.39 – 1.32 (m, 1H), 1.29 – 1.17 (m, 8H), 1.10 (d, *J* = 7.0 Hz, 3H), 0.87 (t, *J* = 7.1 Hz, 3H).

^{13}C-{^{1}H} NMR (126 MHz, CDCl$_3$) δ 176.9, 142.3 – 142.3 (m), 129.4, 129.2 – 128.9 (m), 125.6 – 125.5 (m), 124.2 – 123.3 (m), 64.1, 39.7, 35.1, 33.9, 31.8, 29.3, 27.3, 22.7, 17.2, 14.2.

^{19}F-{^{1}H} NMR (376 MHz, CDCl$_3$) δ -62.46.

HRMS (APPI) m/z calcd. For $C_{18}H_{26}F_3O_2$ ($[M+H]^+$): 331.1885; found: 331.1887.

96:4 er, $[\alpha]_D^{25}$ = +1.9 (c = 1.5, CHCl$_3$).

To determine the enantiomeric ratio, the isolated ester was reduced to the alcohol according to the procedure in section 5. GC analysis (CycloSil-B Column 25 m x 0.25 mm x 0.25 µm, flow: 3.16 mL/min, 90 °C): t_1 = 40.04 min (minor), t_2 = 40.77 min (major). The same major isomer of the alcohol was formed upon the reduction of (*S*)-α-**3ax**, for which the absolute configuration was assigned unambiguously by the X-ray crystallography, enabling the assignment of the absolute configuration of **3ak** as (*S*)-enantiomer.

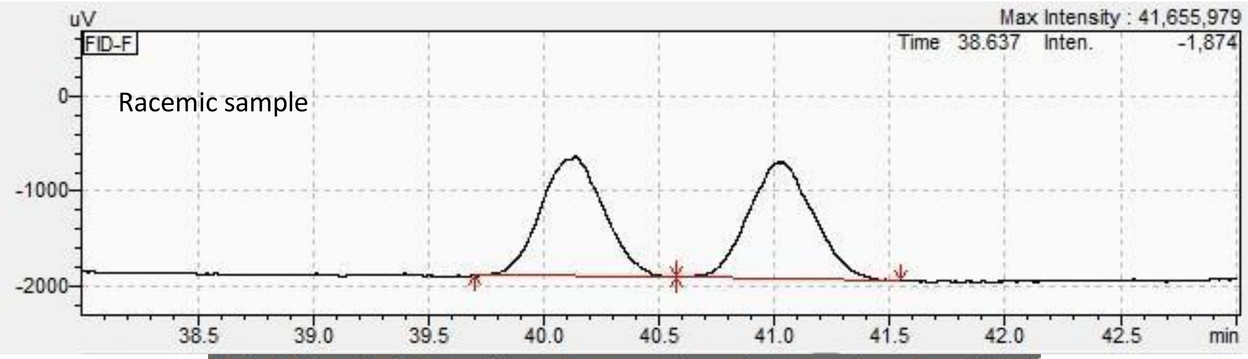

Peak#	Ret. Time	Area	Conc.	Area%
1	40.138	23848	49.958	49.958
2	41.034	23888	50.042	50.042
Total		47736	100.000	100.000

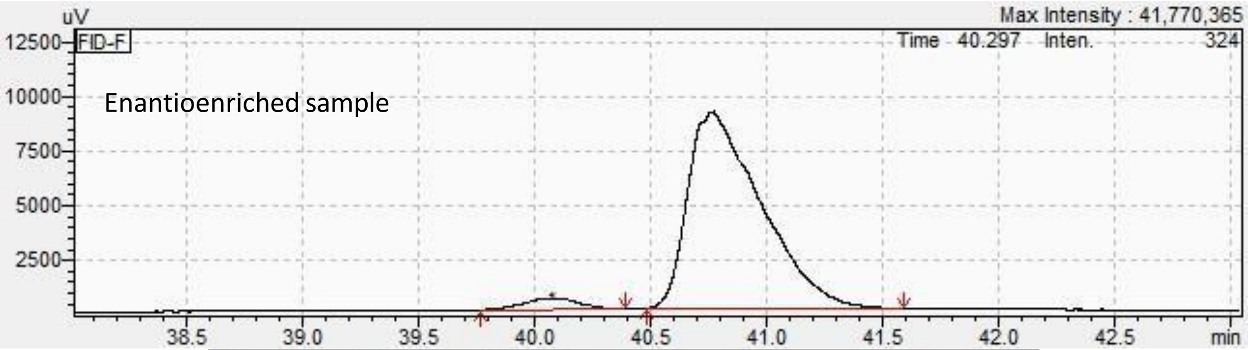

Peak#	Ret. Time	Area	Conc.	Area%
1	40.042	8573	4.173	4.173
2	40.769	196881	95.827	95.827
Total		205454	100.000	100.000

Benzyl (S)-2-methyloctanoate (3al):

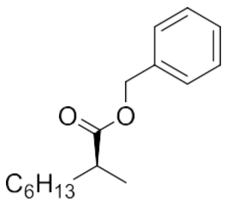

The compound was prepared according to the general procedure by reaction of 1-octene (79 µL, 0.50 mmol) and benzyl alcohol (27 µL, 0.25 mmol), and was isolated by column chromatography (silica gel, petroleum ether/dichloromethane = 8/2), yielding the title product as a colourless oil (58 mg, 93%). The NMR data match those reported in the literature for the racemic product.[3]

^{1}H NMR (500 MHz, CDCl$_3$) δ 7.40 – 7.29 (m, 5H), 5.12 (s, 2H), 2.49 (h, *J* = 7.0 Hz, 1H), 1.72 – 1.63 (m, 1H), 1.46 – 1.39 (m, 1H), 1.32 – 1.23 (m, 8H), 1.16 (d, *J* = 7.0 Hz, 3H), 0.87 (t, *J* = 6.9 Hz, 3H).

^{13}C-{^{1}H} NMR (126 MHz, CDCl$_3$) δ 176.9, 136.5, 128.7, 128.2, 128.2, 66.1, 39.7, 34.0, 31.8, 29.3, 27.3, 22.7, 17.2, 14.2.

96:4 er, $[\alpha]_D^{25}$ = +1.2 (c = 1.3, CHCl$_3$).

To determine the enantiomeric ratio, the isolated ester was reduced to the alcohol according to the procedure in section 5. GC analysis (CycloSil-B Column 25 m x 0.25 mm x 0.25 µm, flow: 3.16 mL/min, 90 °C): t_1 = 40.10 min (minor), t_2 = 40.88 min (major). The same major isomer of the alcohol was formed upon the reduction of (*S*)-α-**3ax**, for which the absolute configuration was assigned unambiguously by the X-ray crystallography, enabling the assignment of the absolute configuration of **3al** as (*S*)-enantiomer.

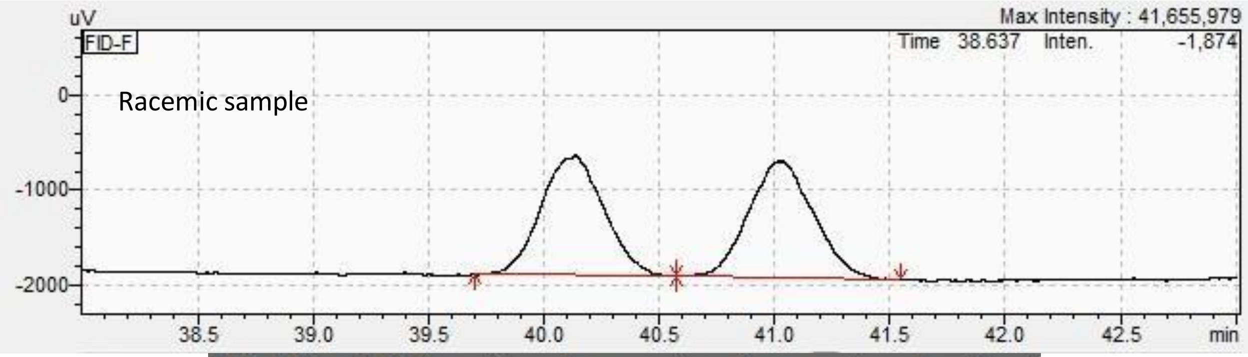

Peak#	Ret. Time	Area	Conc.	Area%
1	40.138	23848	49.958	49.958
2	41.034	23888	50.042	50.042
Total		47736	100.000	100.000

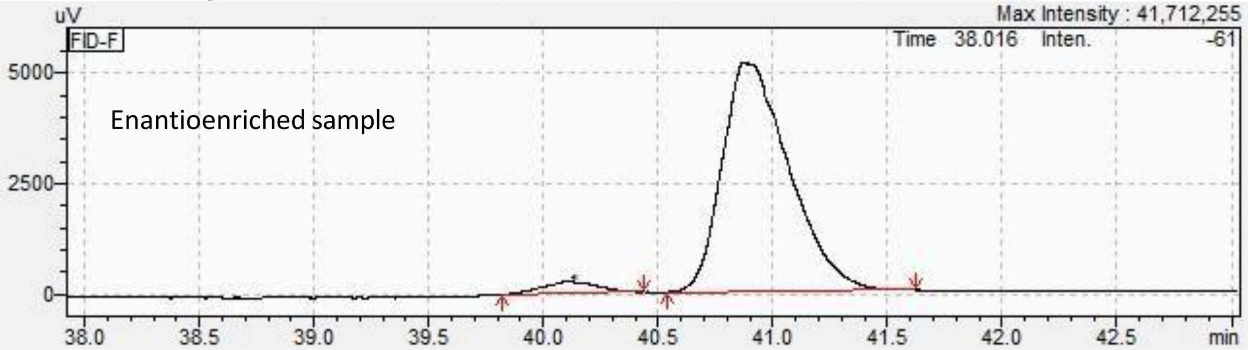

Peak#	Ret. Time	Area	Conc.	Area%
1	40.101	4451	4.057	4.057
2	40.878	105271	95.943	95.943
Total		109722	100.000	100.000

4-(Pentafluoro-λ^6-sulfaneyl)benzyl (S)-2-methyloctanoate (3am):

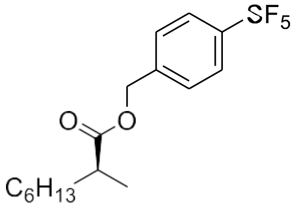

The compound was prepared according to the general procedure by reaction of 1-octene (79 µL, 0.50 mmol) and 4-(pentafluorosulfur)benzyl alcohol (59 mg, 0.25 mmol), was isolated by column chromatography (silica gel, petroleum ether/dichloromethane = 8/2), yielding the title product as a colourless oil (68 mg, 73%).

^{1}H NMR (500 MHz, CDCl$_3$) δ 7.75 (d, *J* = 8.7 Hz, 2H), 7.44 (d, *J* = 8.4 Hz, 2H), 5.15 (s, 2H), 2.51 (h, *J* = 7.0 Hz, 1H), 1.75 – 1.63 (m, 1H), 1.48 – 1.37 (m, 1H), 1.32 – 1.22 (m, 8H), 1.18 (d, *J* = 7.0 Hz, 3H), 0.87 (t, *J* = 7.0 Hz, 3H).

^{13}C-{^{1}H} NMR (126 MHz, CDCl$_3$) δ 176.5, 153.6 – 153.3 (m), 140.2, 126.3 – 126.22 (m), 126.18, 64.5, 39.5, 33.8, 31.7, 29.1, 27.2, 22.6, 17.0, 14.0.

^{19}F-{^{1}H} NMR (377 MHz, CDCl$_3$) δ 87.11 – 81.27 (m), 62.84 (d, *J* = 149.4 Hz).

HRMS (APPI) m/z calcd. For $C_{18}H_{22}F_5O_2S$ ([M+H]$^+$): 373.1261; found: 373.1273.

96:4 er, $[\alpha]_D^{25}$ = -0.9 (c = 1.7, CHCl$_3$).

To determine the enantiomeric ratio, the isolated ester was reduced to the alcohol according to the procedure in section 5. GC analysis (CycloSil-B Column 25 m x 0.25 mm x 0.25 µm, flow: 3.16 mL/min, 90 °C): t_1 = 39.96 min (minor), t_2 = 40.71 min (major). The same major isomer of the alcohol was formed upon the reduction of (*S*)-α-**3ax**, for which the absolute configuration was assigned unambiguously by the X-ray crystallography, enabling the assignment of the absolute configuration of **3am** as (*S*)-enantiomer.

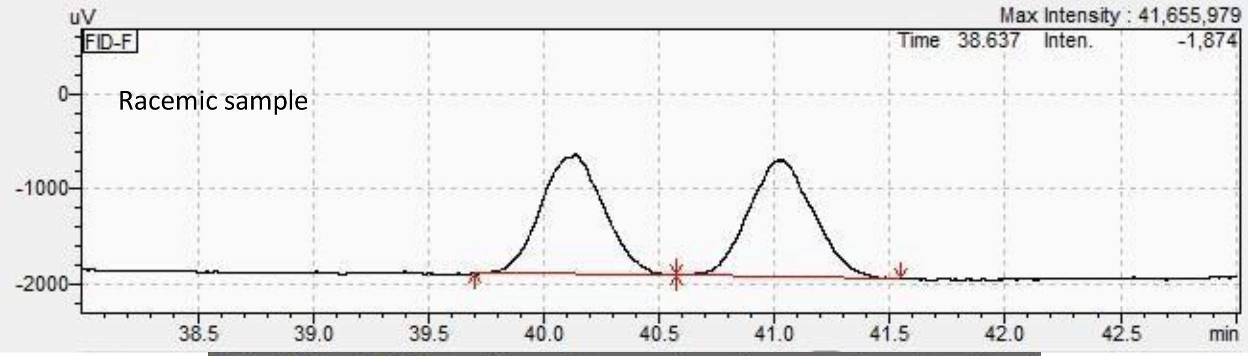

Peak#	Ret. Time	Area	Conc.	Area%
1	40.138	23848	49.958	49.958
2	41.034	23888	50.042	50.042
Total		47736	100.000	100.000

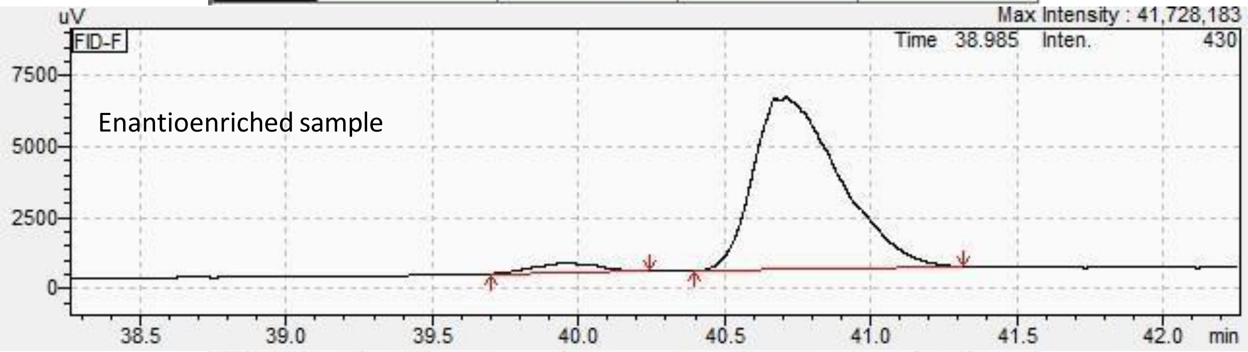

Peak#	Ret. Time	Area	Conc.	Area%
1	39.956	5072	3.919	3.919
2	40.710	124341	96.081	96.081
Total		129413	100.000	100.000

(Perfluorophenyl)methyl (S)-2-methyloctanoate (3an):

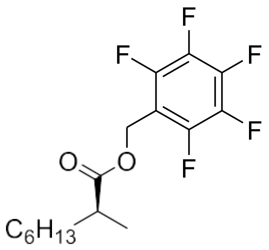

The compound was prepared according to the general procedure by reaction of 1-octene (79 µL, 0.50 mmol) and 2,3,4,5,6-Pentafluorobenzyl alcohol (50 mg, 0.25 mmol), and was isolated by column chromatography (silica gel, petroleum ether/dichloromethane = 8/2), yielding the title product as a colourless oil (28 mg, 33%). The NMR data match those reported in the literature for the racemic product.[3]

^{1}H NMR (500 MHz, CDCl$_3$) δ 5.19 (d, *J* = 1.5 Hz, 2H), 2.45 (h, *J* = 7.0 Hz, 1H), 1.67 – 1.58 (m, 1H), 1.46 – 1.36 (m, 1H), 1.31 – 1.21 (m, 8H), 1.14 (d, *J* = 7.0 Hz, 3H), 0.87 (t, *J* = 6.9 Hz, 3H).

^{13}C-{^{1}H} NMR (126 MHz, CDCl$_3$) δ 176.3, 146.7 – 144.9 (m), 142.7 – 140.9 (m), 138.5 – 136.8 (m), 109.9 (td, *J* = 17.2, 3.9 Hz), 53.3, 39.5, 33.9, 31.8, 29.2, 27.2, 22.7, 17.0, 14.2.

^{19}F-{^{1}H} NMR (377 MHz, CDCl3) δ -141.92 – -142.13 (m), -152.62 – -152.99 (m), -161.53 – -161.84 (m).

96:4 er, $[\alpha]_D^{25}$ = -1.9 (c = 1.4, CHCl$_3$).

To determine the enantiomeric ratio, the isolated ester was reduced to the alcohol according to the procedure in section 5. GC analysis (CycloSil-B Column 25 m x 0.25 mm x 0.25 µm, flow: 3.16 mL/min, 90 °C): t_1 = 40.14 min (minor), t_2 = 40.93 min (major). The same major isomer of the alcohol was formed upon the reduction of (*S*)-α-**3ax**, for which the absolute configuration was assigned unambiguously by the X-ray crystallography, enabling the assignment of the absolute configuration of **3an** as (*S*)-enantiomer.

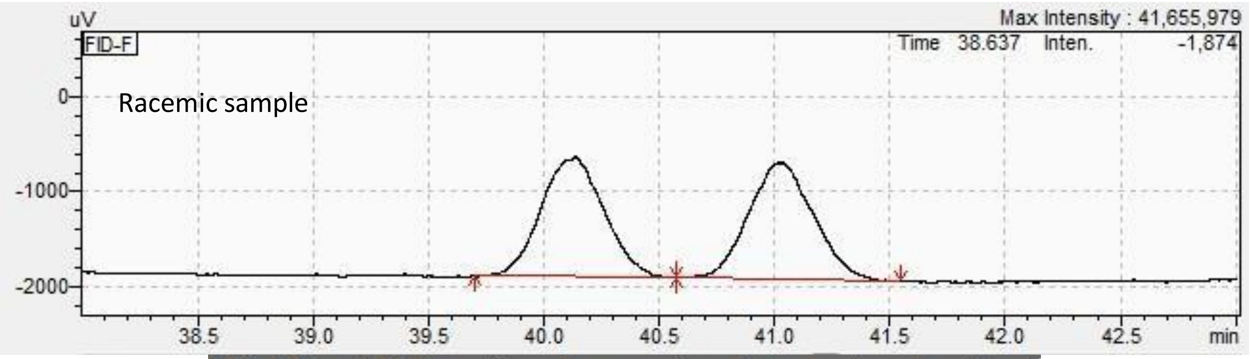

Peak#	Ret. Time	Area	Conc.	Area%
1	40.138	23848	49.958	49.958
2	41.034	23888	50.042	50.042
Total		47736	100.000	100.000

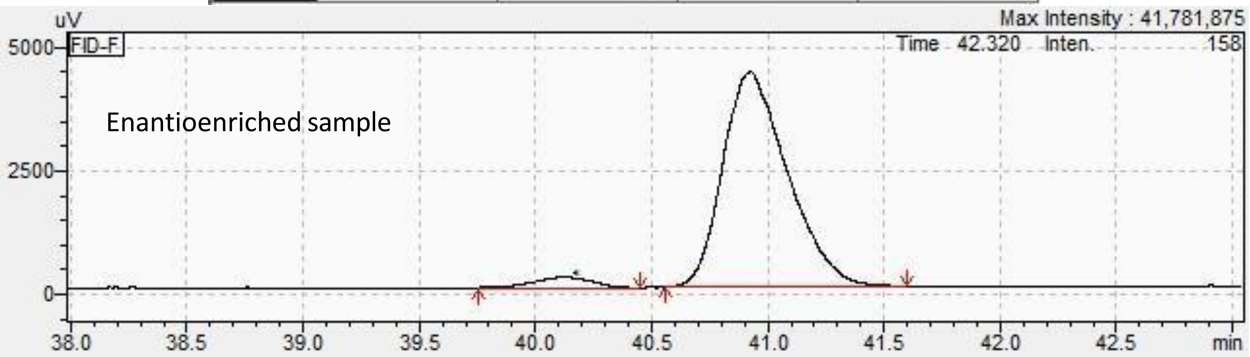

Peak#	Ret. Time	Area	Conc.	Area%
1	40.137	3664	4.147	4.147
2	40.927	84694	95.853	95.853
Total		88358	100.000	100.000

Thiophen-2-ylmethyl (S)-2-methyloctanoate (3ao):

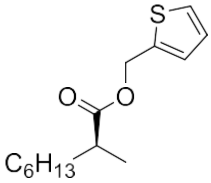

The compound was prepared according to the general procedure by reaction of 1-octene (79 µL, 0.50 mmol) and thiophen-2-ylmethanol (24 µL, 0.25 mmol), and was isolated by column chromatography (silica gel, petroleum ether/dichloromethane = 8/2), yielding the title product as a colourless oil (29 mg, 49%). The NMR data match those reported in the literature for the racemic product.[3]

^{1}H NMR (500 MHz, CDCl$_3$) δ 7.31 (dd, *J* = 5.1, 1.2 Hz, 1H), 7.09 – 7.06 (m, 1H), 6.98 (dd, *J* = 5.1, 3.5 Hz, 1H), 5.27 (s, 2H), 2.46 (h, *J* = 7.0 Hz, 1H), 1.70 – 1.60 (m, 1H), 1.45 – 1.37 (m, 1H), 1.28 – 1.22 (m, 8H), 1.14 (d, *J* = 7.0 Hz, 3H), 0.86 (t, *J* = 7.0 Hz, 3H).

^{13}C-{^{1}H} NMR (126 MHz, CDCl$_3$) δ 176.7, 138.5, 128.0, 126.9, 126.8, 60.4, 39.6, 33.9, 31.8, 29.3, 27.2, 22.7, 17.1, 14.2.

96:4 er, $[\alpha]_D^{25}$ = +1.2 (c = 1.5, CHCl$_3$).

To determine the enantiomeric ratio, the isolated ester was reduced to the alcohol according to the procedure in section 5. GC analysis (CycloSil-B Column 25 m x 0.25 mm x 0.25 µm, flow: 3.16 mL/min, 90 °C): t_1 = 40.14 min (minor), t_2 = 40.92 min (major). The same major isomer of the alcohol was formed upon the reduction of (*S*)-α-**3ax**, for which the absolute configuration was assigned unambiguously by the X-ray crystallography, enabling the assignment of the absolute configuration of **3ao** as (*S*)-enantiomer.

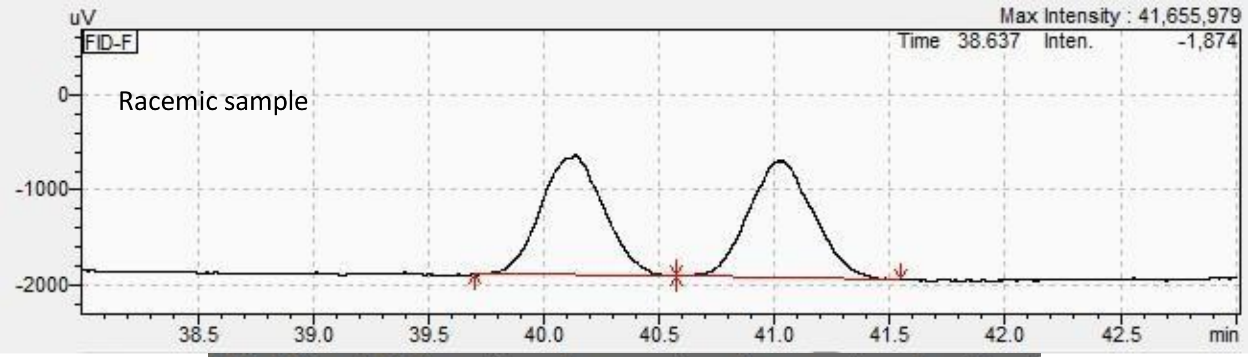

Peak#	Ret. Time	Area	Conc.	Area%
1	40.138	23848	49.958	49.958
2	41.034	23888	50.042	50.042
Total		47736	100.000	100.000

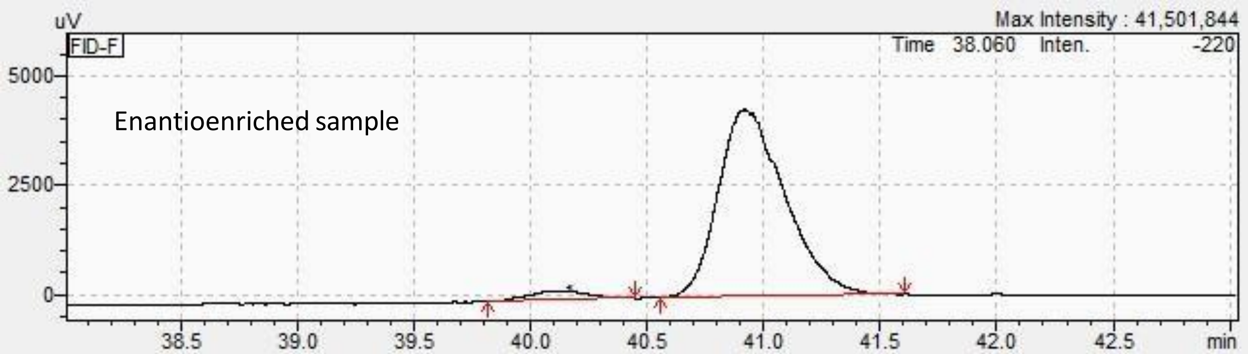

Peak#	Ret. Time	Area	Conc.	Area%
1	40.136	3572	4.028	4.028
2	40.920	85089	95.972	95.972
Total		88661	100.000	100.000

2-(Methylthio)ethyl (S)-2-methyloctanoate (3ap):

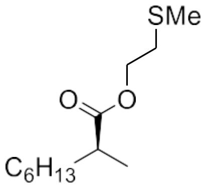

The compound was prepared according to the general procedure by reaction of 1-octene (79 µL, 0.50 mmol) and 2-(Methylthio)ethanol (21.8 µL, 0.25 mmol), $PdBr_2(ACN)_2$ (1.8 mg, 0.005 mmol), **L10** (8.8 mg, 0.01 mmol), at 45 °C for 48 h, and was isolated by column chromatography (silica gel, petroleum ether/dichloromethane = 8/2), yielding the title product as a colourless oil (52 mg, 89%).

^{1}H NMR (500 MHz, CDCl$_3$) δ 4.24 (t, *J* = 6.8 Hz, 2H), 2.74 – 2.68 (m, 2H), 2.44 (h, *J* = 7.0 Hz, 1H), 2.15 (s, 3H), 1.69 – 1.60 (m, 1H), 1.45 – 1.37 (m, 1H), 1.29 – 1.25 (m, 8H), 1.15 (d, *J* = 6.9 Hz, 3H), 0.87 (t, *J* = 7.0 Hz, 3H).

^{13}C NMR (126 MHz, CDCl$_3$) δ 176.9, 62.9, 39.7, 33.9, 32.8, 31.8, 29.3, 27.3, 22.7, 17.2, 15.9, 14.2.

HRMS (ESI) m/z calcd. for $C_{12}H_{23}O_2S$ ([M+H]$^+$): 231.1413; found: 231.1410.

95:5 er, $[\alpha]_D^{24.3}$ = −8.5 (c = 0.3, CHCl$_3$).

To determine the enantiomeric ratio, the isolated ester was reduced to the alcohol according to the procedure in section 5. GC analysis (CycloSil-B Column 25 m x 0.25 mm x 0.25 µm, flow: 4.0 mL/min, 75 °C): t_1 = 58.5 min (minor), t_2 = 59.9 min (major). The same major isomer of the alcohol was formed upon the reduction of (*S*)-α-**3ax**, for which the absolute configuration was assigned unambiguously by the X-ray crystallography, enabling the assignment of the absolute configuration of **3ap** as (*S*)-enantiomer.

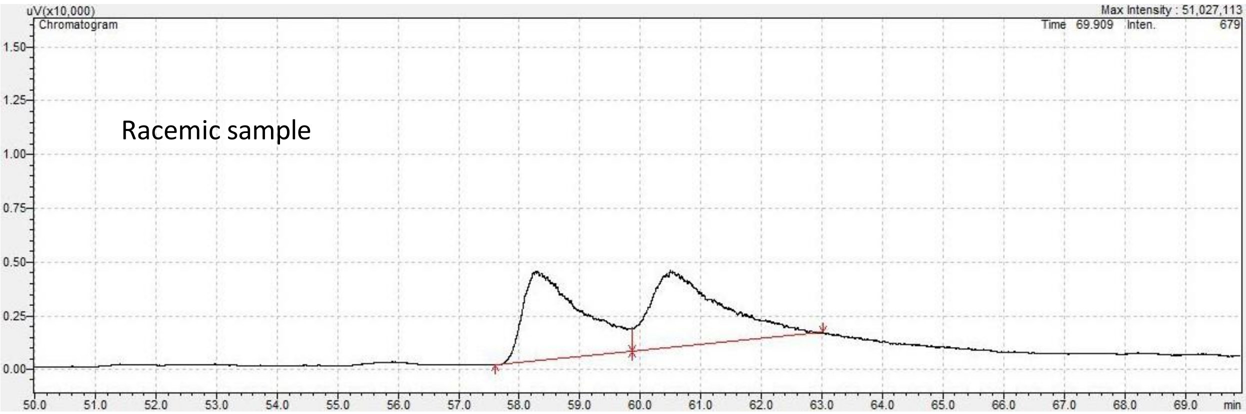

Peak#	Ret.Time	Area	Area%
1	58.274	279668.3	48.9658
2	60.496	291482.1	51.0342

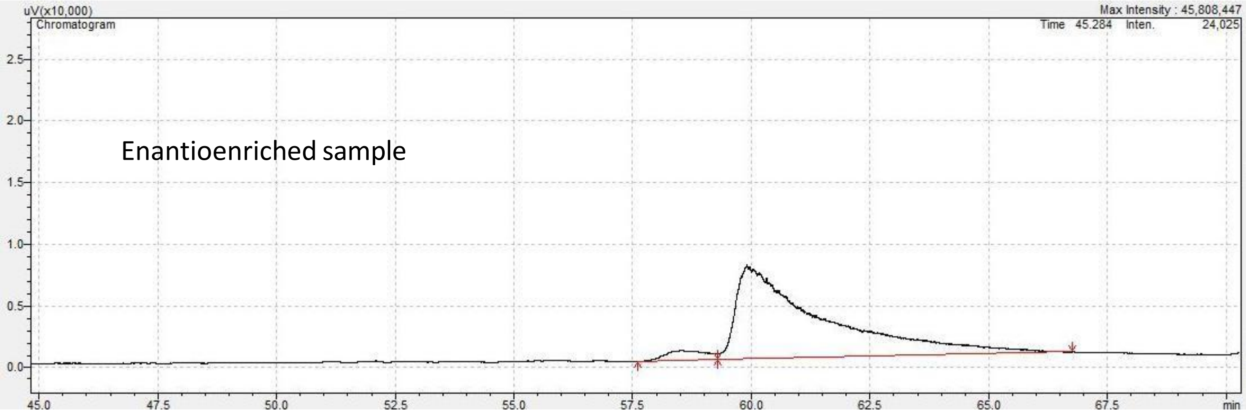

Peak#	Ret.Time	Area	Area%
1	58.534	48039.2	4.8239
2	59.904	947829.7	95.1761

3-(2-Oxopyrrolidin-1-yl)propyl (S)-2-methyloctanoate (3aq):

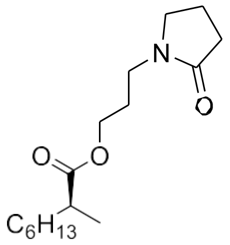

The compound was prepared according to the general procedure by reaction of 1-octene (79 µL, 0.50 mmol) and 1-(3-Hydroxypropyl)-2-pyrrolidone (32 µL, 0.25 mmol), $PdBr_2(ACN)_2$ (1.8 mg, 0.005 mmol), **L10** (8.8 mg, 0.01 mmol), in 1,4-dioxane, at 45 °C for 5 days, and was isolated by column chromatography (silica gel, petroleum ether/dichloromethane = 2/8), yielding the title product as a colourless oil (63 mg, 89%).

^{1}H NMR (500 MHz, CDCl$_3$) δ 4.08 (t, *J* = 6.5 Hz, 2H), 3.42 – 3.31 (m, 4H), 2.47 – 2.32 (m, 3H), 2.07 – 1.98 (m, 2H), 1.91 – 1.83 (m, 2H), 1.64 – 1.58 (m, 1H), 1.44 – 1.35 (m, 1H), 1.30 – 1.23 (m, 8H), 1.14 (d, *J* = 6.9 Hz, 3H), 0.90 – 0.85 (m, 3H).

^{13}C NMR (126 MHz, CDCl$_3$) δ 177.0, 175.2, 61.8, 47.5, 39.7, 39.6, 33.9, 31.8, 31.1, 29.3, 27.3, 26.8, 22.7, 18.1, 17.2, 14.2.

HRMS (ESI) m/z calcd. for $C_{16}H_{30}O_3N$ ([M+H]$^+$): 284.2220; found: 284.2217.

87:13 er, $[\alpha]_D^{24.7}$ = +4.9 (c = 0.4, $CHCl_3$).

GC analysis (CP-Chirasil Dex CB Column 25 m x 0.25 mm x 0.25 µm, flow: 1.65 mL/min, 130 °C): t_1 = 23.4 min (major), t_2 = 24.7 min (minor). The absolute configuration of **3aq** was assigned as (*S*)-enantiomer by analogy to *(S)*-**3aa**.

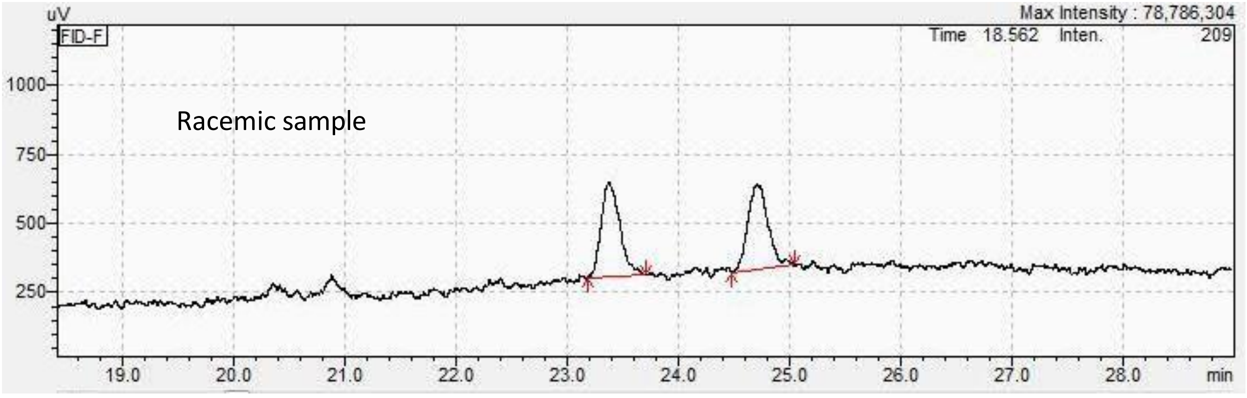

Peak#	Ret. Time	Area	Conc.	Area%
1	23.369	3966	50.030	50.030
2	24.707	3962	49.970	49.970
Total		7928	100.000	100.000

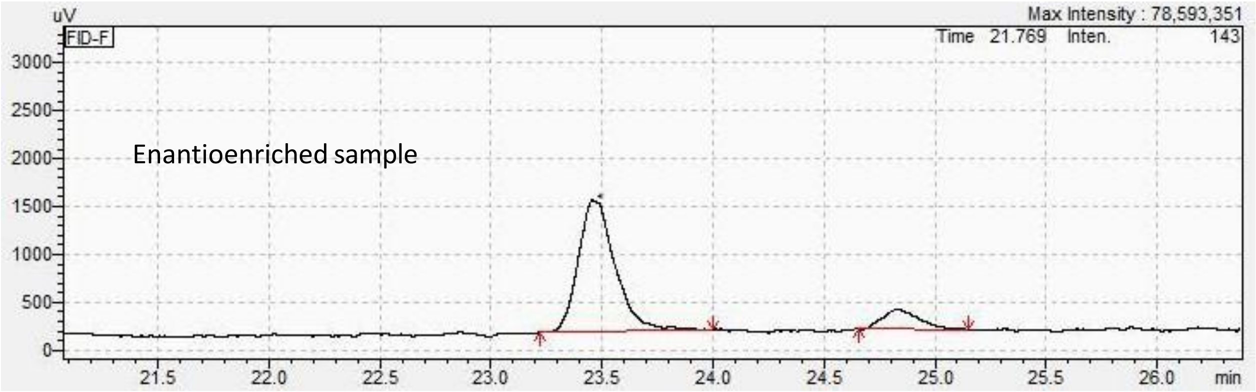

Peak#	Ret. Time	Area	Conc.	Area%
1	23.458	15713	87.100	87.100
2	24.827	2327	12.900	12.900
Total		18041	100.000	100.000

2-(1,3-Dioxoisoindolin-2-yl)ethyl (S)-2-methyloctanoate (3ar):

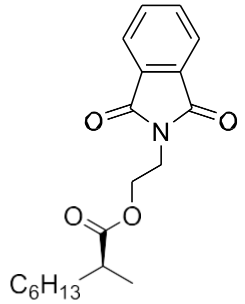

0.25 mmol-scale: The compound was prepared according to the general procedure by reaction of 1-octene (79 µL, 0.50 mmol) and 1-(3-Hydroxypropyl)-2-pyrrolidone (47.8 mg, 0.25 mmol), $PdBr_2(ACN)_2$ (1.8 mg, 0.005 mmol), **L10** (8.8 mg, 0.01 mmol), in 1,4-dioxane, at 45 °C for 48 h, and was isolated by column chromatography (silica gel, petroleum ether/ethyl acetate = 8/2), yielding the title product as a yellow oil (78 mg, 94%).

2.5 mmol-scale with ½ Pd lodaing: The reaction was performed in a 20 mL vial with 1-octene (785 µL, 5.0 mmol) and 1-(3-Hydroxypropyl)-2-pyrrolidone (478 mg, 2.5 mmol), $PdBr_2(ACN)_2$ (8.7 mg, 0.025 mmol), **L10** (43.7 mg, 0.05 mmol), in 1,4-dioxane, at 45 °C for 48 h, yielding the title product as a yellow oil (450 mg, 54%).

^{1}H NMR (500 MHz, $CDCl_3$) δ 7.88 – 7.82 (m, 2H), 7.75 – 7.69 (m, 2H), 4.37 – 4.27 (m, 2H), 4.01 – 3.91 (m, 2H), 2.37 (h, J = 7.0 Hz, 1H), 1.61 – 1.52 (m, 1H), 1.36 – 1.29 (m, 1H), 1.25 – 1.14 (m, 8H), 1.08 (d, J = 7.0 Hz, 3H), 0.84 (t, J = 7.2 Hz, 3H).

^{13}C NMR (126 MHz, $CDCl_3$) δ 176.8, 168.2, 134.2, 132.1, 123.5, 61.3, 39.5, 37.2, 33.7, 31.8, 29.3, 27.2, 22.7, 17.0, 14.2.

HRMS (ESI) m/z calcd. For $C_{19}H_{26}O_4N$ ($[M+H]^+$): 332.1856; found: 332.1846.

95:5 er, $[\alpha]_D^{23.4}$ = −1.2 (c = 0.3, $CHCl_3$).

To determine the enantiomeric ratio, the isolated ester was reduced to the alcohol according to the procedure in section 5. GC analysis (CycloSil-B Column 25 m x 0.25 mm x 0.25 µm, flow: 4.0 mL/min, 75 °C): t_1 = 58.5 min (minor), t_2 = 59.9 min (major). The same major isomer of the alcohol was formed upon the reduction of (*S*)-α-**3ax**, for which the absolute configuration was assigned unambiguously by the X-ray crystallography, enabling the assignment of the absolute configuration of **3ar** as (*S*)-enantiomer.

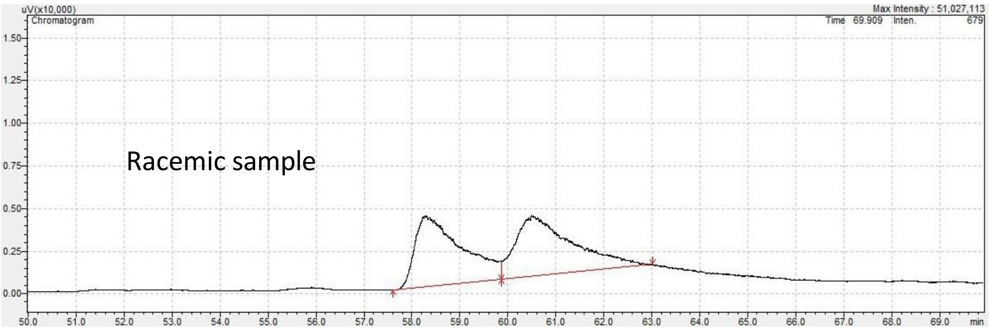

Peak#	Ret.Time	Area	Area%
1	58.274	279668.3	48.9658
2	60.496	291482.1	51.0342

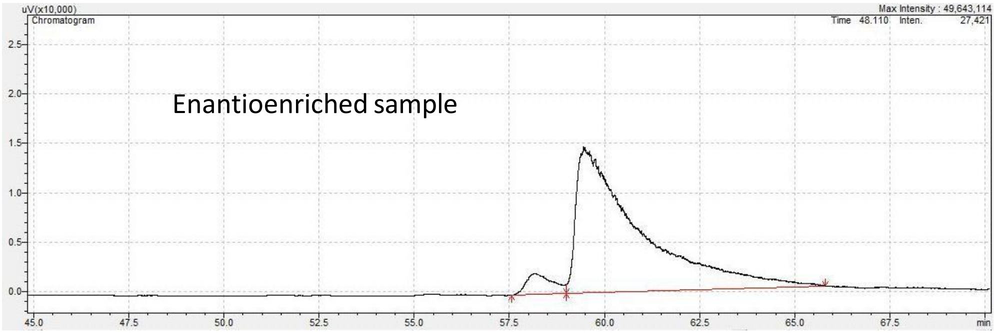

Peak#	Ret.Time	Area	Area%
1	58.178	110204.6	6.0420
2	59.452	1713771.8	93.9580

(S)-1-Phenylethyl (S)-2-methyloctanoate (3at):

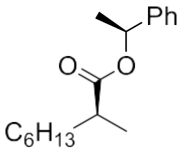

The compound was prepared according to the general procedure by reaction of 1-octene (79 µL, 0.50 mmol) and (S)-phenylethanol (31 µL, 0.25 mmol), and was isolated by column chromatography (silica gel, petroleum ether/dichloromethane = 8/2), yielding the title product as a colourless oil (55 mg, 84%). The NMR data match those reported in the literature for the reaction with the racemic phenylethanol.[3]

^{1}H NMR (500 MHz, $CDCl_3$) δ 7.36 – 7.27 (m, 5H), 5.89 (q, *J* = 6.6 Hz, 1H), 2.45 (h, *J* = 7.0 Hz, 1H), 1.70 – 1.61 (m, 1H), 1.53 (d, *J* = 6.6 Hz, 3H), 1.45 – 1.38 (m, 1H), 1.30 – 1.24 (m, 8H), 1.13 (d, *J* = 7.0 Hz, 3H), 0.88 (t, *J* = 7.0 Hz, 3H).

^{13}C-{^{1}H} NMR (126 MHz, $CDCl_3$) δ 176.2, 142.1, 128.6, 127.9, 126.2, 72.0, 39.8, 34.0, 31.9, 29.3, 27.3, 22.7, 22.4, 17.2, 14.2.

92:8 dr, $[\alpha]_D^{25}$ = +26.5 (c = 1.3, $CHCl_3$).

To determine the diastereomeric ratio, the isolated ester was reduced to the alcohol according to the procedure in section 5. GC analysis (CycloSil-B Column 25 m x 0.25 mm x 0.25 µm, flow: 3.16 mL/min, 90 °C): t_1 = 40.08 min (minor), t_2 = 40.89 min (major). The same major isomer of the alcohol was formed upon the reduction of (*S*)-α-**3ax**, for which the absolute configuration was assigned unambiguously by the X-ray crystallography, enabling the assignment of the absolute configuration of **3aq** as (*S*)-enantiomer.

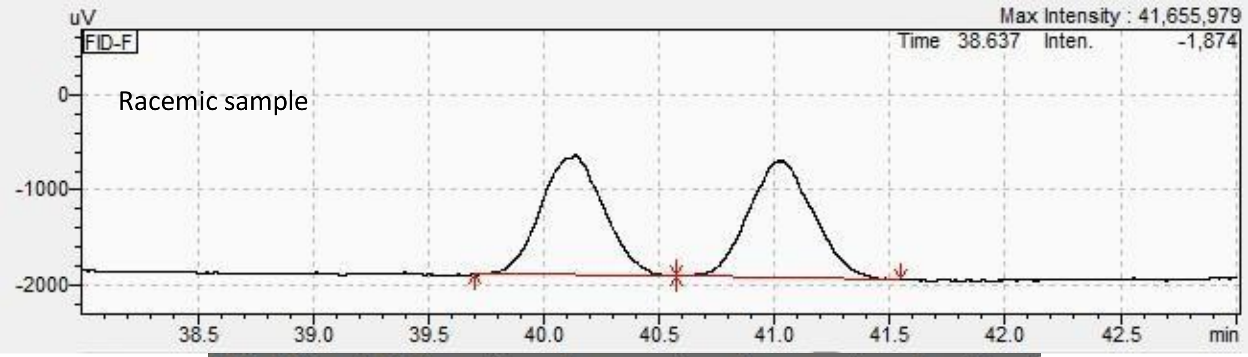

Peak#	Ret. Time	Area	Conc.	Area%
1	40.138	23848	49.958	49.958
2	41.034	23888	50.042	50.042
Total		47736	100.000	100.000

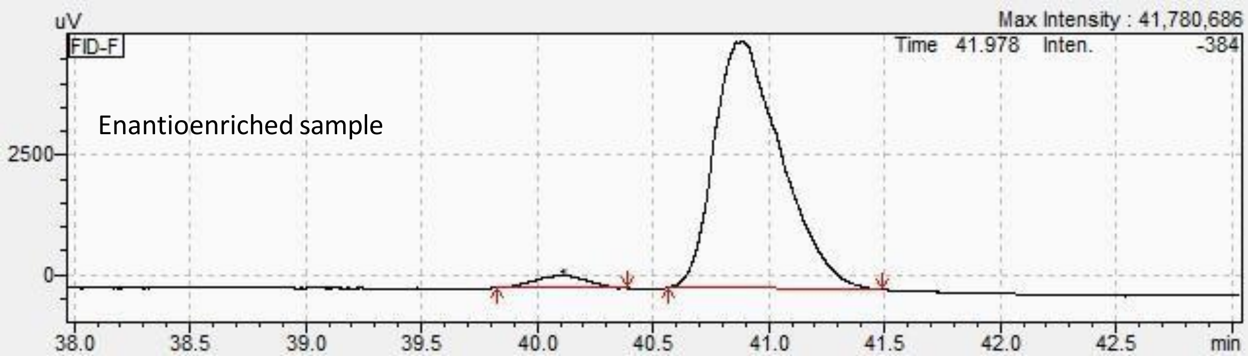

Peak#	Ret. Time	Area	Conc.	Area%
1	40.082	4105	3.795	3.795
2	40.887	104044	96.205	96.205
Total		108149	100.000	100.000

(R)-1-Phenylethyl (S)-2-methyloctanoate (3at'):

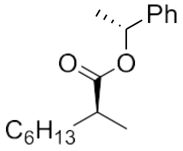

The compound was prepared according to the general procedure by reaction of 1-octene (79 µL, 0.50 mmol) and (R)-phenylethanol (31 µL, 0.25 mmol), and was isolated by column chromatography (silica gel, petroleum ether/dichloromethane = 8/2), yielding the title product as a colourless oil (53 mg, 81%). The NMR data match those reported in the literature for the reaction with the racemic phenylethanol.[3]

^{1}H NMR (500 MHz, $CDCl_3$) δ 7.37 – 7.26 (m, 5H), 5.89 (q, *J* = 6.6 Hz, 1H), 2.45 (h, *J* = 7.0 Hz, 1H), 1.67 – 1.60 (m, 1H), 1.53 (d, *J* = 6.6 Hz, 3H), 1.41 – 1.36 (m, 1H), 1.26 – 1.18 (m, 8H), 1.15 (d, *J* = 7.0 Hz, 3H), 0.86 (t, *J* = 7.0 Hz, 3H).

^{13}C-{^{1}H} NMR (126 MHz, $CDCl_3$) δ 176.3, 142.1, 128.6, 127.9, 126.2, 72.0, 39.8, 33.9, 31.8, 29.3, 27.2, 22.7, 22.4, 17.2, 14.2.

93:7 dr, $[\alpha]_D^{25}$ = -15.8 (c = 1.3, $CHCl_3$).

To determine the diastereomeric ratio, the isolated ester was reduced to the alcohol according to the procedure in section 5. GC analysis (CycloSil-B Column 25 m x 0.25 mm x 0.25 µm, flow: 3.16 mL/min, 90 °C): t_1 = 40.06 min (minor), t_2 = 40.73 min (major). The same major isomer of the alcohol was formed upon the reduction of (*S*)-α-**3ax**, for which the absolute configuration was assigned unambiguously by the X-ray crystallography, enabling the assignment of the absolute configuration of **3aq'** as (*S*)-enantiomer.

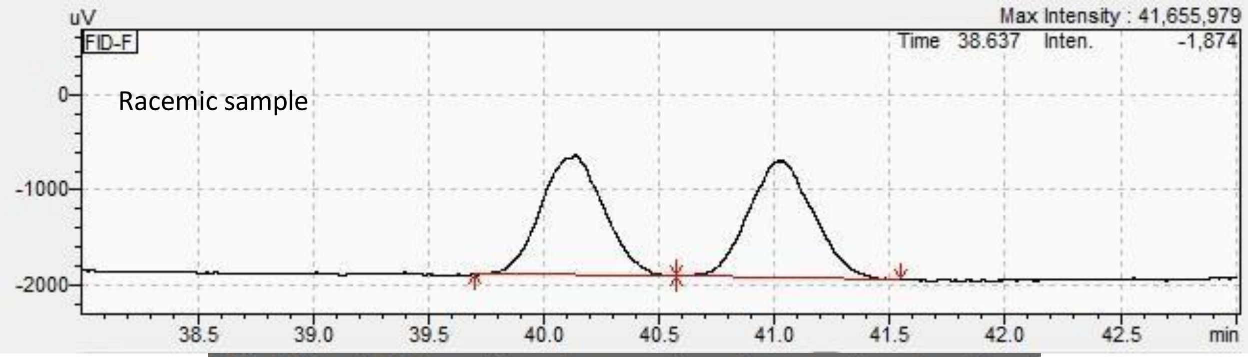

Peak#	Ret. Time	Area	Conc.	Area%
1	40.138	23848	49.958	49.958
2	41.034	23888	50.042	50.042
Total		47736	100.000	100.000

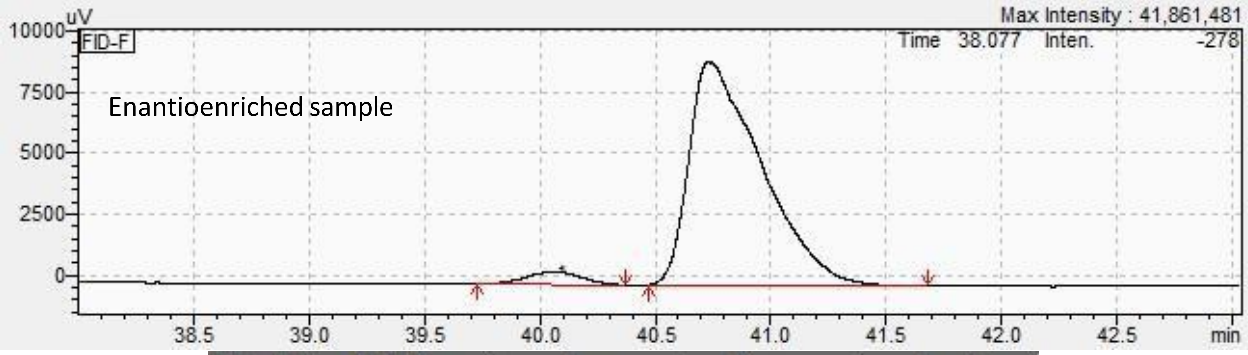

Peak#	Ret. Time	Area	Conc.	Area%
1	40.062	9015	4.316	4.316
2	40.726	199834	95.684	95.684
Total		208849	100.000	100.000

(2R,5S)-2-Isopropyl-5-methylcyclohexyl (2S)-2-methyloctanoate (3au):

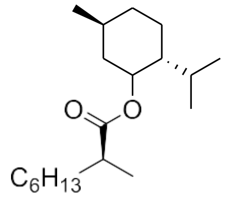

The compound was prepared according to the general procedure by reaction of 1-octene (79 µL, 0.50 mmol) and D-menthol (39 mg, 0.25 mmol), and was isolated by column chromatography (silica gel, petroleum ether/dichloromethane = 8/2), yielding the title product as a colourless oil (63 mg, 85%). The NMR data match those reported in the literature.[3]

^{1}H NMR (500 MHz, $CDCl_3$) δ 4.66 (td, *J* = 10.9, 4.4 Hz, 1H), 2.44 – 2.36 (m, 1H), 2.02 – 1.95 (m, 1H), 1.90 (dtd, *J* = 13.9, 7.0, 2.8 Hz, 1H), 1.72 – 1.62 (m, 2H), 1.53 – 1.34 (m, 2H), 1.30 – 1.23 (m, 8H), 1.13 (d, *J* = 7.0 Hz, 3H), 1.05 (qd, *J* = 13.5, 3.7 Hz, 2H), 0.98 – 0.82 (m, 12H), 0.75 (d, *J* = 7.0 Hz, 3H).

^{13}C-{^{1}H} NMR (126 MHz, $CDCl_3$) δ 176.6, 73.8, 47.2, 41.0, 40.1, 34.5, 33.9, 31.9, 31.5, 29.3, 27.3, 26.2, 23.4, 22.7, 22.2, 21.0, 17.4, 16.2, 14.2.

96:4 dr, $[\alpha]_D^{25}$ = +9.7 (c = 1.5, $CHCl_3$).

To determine the diastereomeric ratio, the isolated ester was reduced to the alcohol according to the procedure in section 5. GC analysis (CycloSil-B Column 25 m x 0.25 mm x 0.25 µm, flow: 3.16 mL/min, 90 °C): t_1 = 40.11 min (minor), t_2 = 40.89 min (major). The same major isomer of the alcohol was formed upon the reduction of (*S*)-α-**3ax**, for which the absolute configuration was assigned unambiguously by the X-ray crystallography, enabling the assignment of the absolute configuration of **3ar** as (*S*)-enantiomer.

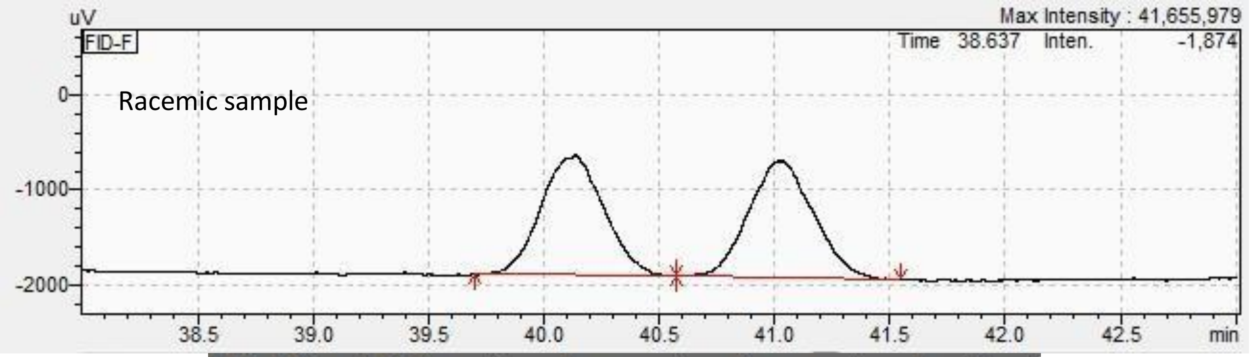

Peak#	Ret. Time	Area	Conc.	Area%
1	40.138	23848	49.958	49.958
2	41.034	23888	50.042	50.042
Total		47736	100.000	100.000

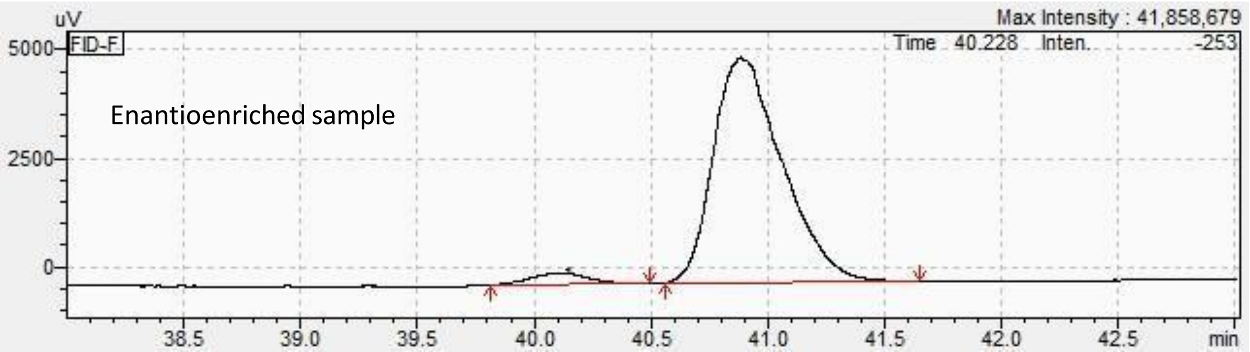

Peak#	Ret. Time	Area	Conc.	Area%
1	40.109	4741	4.285	4.285
2	40.887	105886	95.715	95.715
Total		110627	100.000	100.000

(S)-3-Methoxy-2-methyl-3-oxopropyl (S)-2-methyloctanoate (3av):

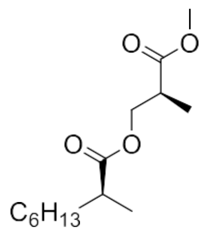

The compound was prepared according to the general procedure by reaction of 1-octene (79 µL, 0.50 mmol) and (S)-Roche ester (30 mg 0.25 mmol), and was isolated by column chromatography (silica gel, petroleum ether/dichloromethane = 8/2), yielding the title product as a colourless oil (59 mg, 91%).

^{1}H NMR (500 MHz, CDCl$_3$) δ 4.25 – 4.14 (m, 2H), 3.70 (s, 3H), 2.84 – 2.77 (m, 1H), 2.45 – 2.38 (m, 1H), 1.65 – 1.60 (m, 1H), 1.41 – 1.35 (m, 1H), 1.29 – 1.24 (m, 8H), 1.20 (d, *J* = 7.1 Hz, 3H), 1.12 (d, *J* = 7.0 Hz, 3H), 0.88 (t, *J* = 6.9 Hz, 3H).

^{13}C-{^{1}H} NMR (126 MHz, CDCl$_3$) δ 176.7, 174.5, 65.5, 52.0, 39.6, 39.3, 33.9, 31.8, 29.3, 27.3, 22.8, 17.2, 14.2, 13.9.

HRMS (APPI) m/z calcd. For $C_{14}H_{27}O_4$ ([M+H]$^+$): 259.1909; found: 259.1912.

96:4 dr, $[\alpha]_D^{25}$ = +1.5 (c = 1.5, CHCl$_3$).

To determine the diastereomeric ratio, the isolated ester was reduced to the alcohol according to the procedure in section 5. GC analysis (CycloSil-B Column 25 m x 0.25 mm x 0.25 µm, flow: 3.16 mL/min, 90 °C): t_1 = 40.10 min (minor), t_2 = 40.86 min (major). The same major isomer of the alcohol was formed upon the reduction of (*S*)-α-**3ax**, for which the absolute configuration was assigned unambiguously by the X-ray crystallography, enabling the assignment of the absolute configuration of **3as** as (*S*)-enantiomer.

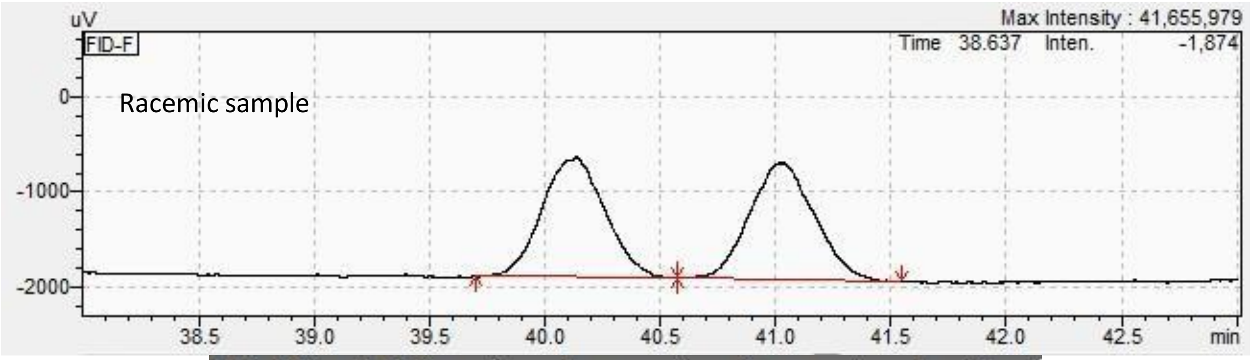

Peak#	Ret. Time	Area	Conc.	Area%
1	40.138	23848	49.958	49.958
2	41.034	23888	50.042	50.042
Total		47736	100.000	100.000

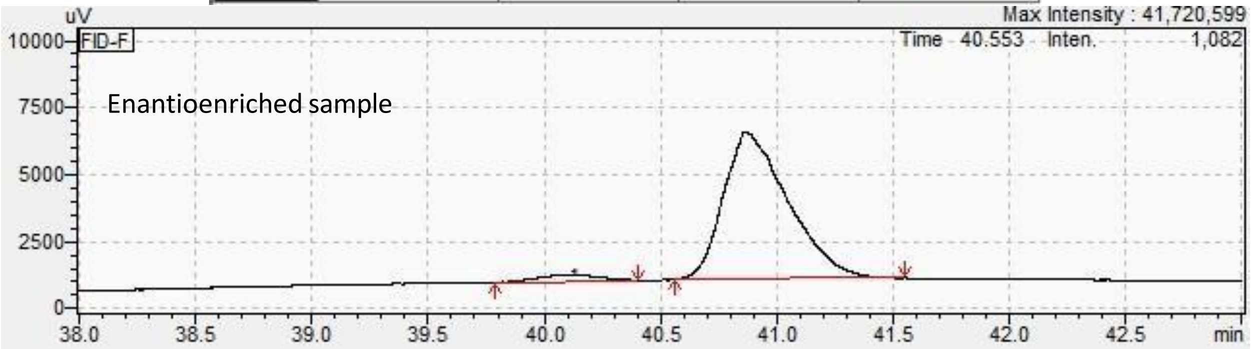

Peak#	Ret. Time	Area	Conc.	Area%
1	40.095	4405	3.998	3.998
2	40.861	105780	96.002	96.002
Total		110185	100.000	100.000

(S)-3,7-Dimethyloct-6-en-1-yl (S)-2-methyloctanoate (3aw):

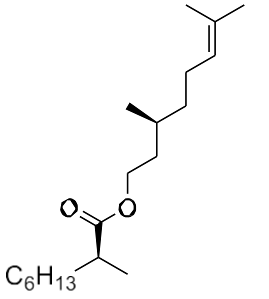

The compound was prepared according to the general procedure by reaction of 1-octene (79 µL, 0.50 mmol) and (-)-β-citronellol (39 mg, 0.25 mmol), and was isolated by column chromatography (silica gel, petroleum ether/dichloromethane = 8/2), yielding the title product as a colourless oil (61 mg, 82%).

^{1}H NMR (500 MHz, CDCl$_3$) δ 5.09 (ddt, *J* = 7.1, 4.3, 1.4 Hz, 1H), 4.15 – 4.04 (m, 2H), 2.41 (h, *J* = 7.0 Hz, 1H), 2.04 – 1.92 (m, 2H), 1.71 – 1.62 (m, 5H), 1.60 (s, 3H), 1.47 – 1.37 (m, 2H), 1.33 – 1.18 (m, 11H), 1.13 (d, *J* = 7.0 Hz, 3H), 0.91 (d, *J* = 6.6 Hz, 3H), 0.88 (t, *J* = 7.1 Hz, 3H).

^{13}C-{^{1}H} NMR (126 MHz, CDCl$_3$) δ 177.2, 131.5, 124.7, 62.8, 39.8, 37.1, 35.7, 34.0, 31.9, 29.6, 29.3, 27.4, 25.9, 25.6, 22.8, 19.5, 17.8, 17.3, 14.2.

HRMS (APPI) m/z calcd. For $C_{19}H_{37}O_2$ ([M+H]$^+$): 297.2794; found: 297.2792.

95:5 dr, $[\alpha]_D^{25}$ = +1.7 (c = 1.5, CHCl$_3$).

To determine the diastereomeric ratio, the isolated ester was reduced to the alcohol according to the procedure in section 5. GC analysis (CycloSil-B Column 25 m x 0.25 mm x 0.25 µm, flow: 3.16 mL/min, 90 °C): t_1 = 40.09 min (minor), t_2 = 40.90 min (major). The same major isomer of the alcohol was formed upon the reduction of (*S*)-α-**3ax**, for which the absolute configuration was assigned unambiguously by the X-ray crystallography, enabling the assignment of the absolute configuration of **3at** as (*S*)-enantiomer.

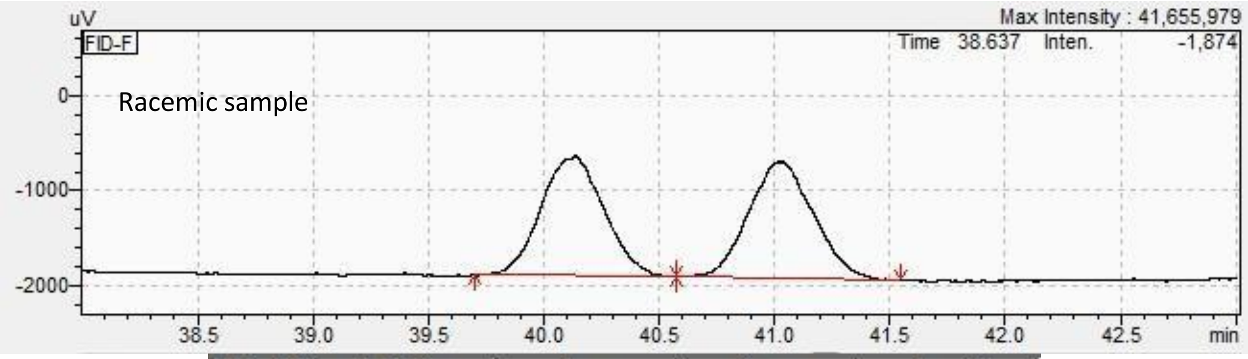

Peak#	Ret. Time	Area	Conc.	Area%
1	40.138	23848	49.958	49.958
2	41.034	23888	50.042	50.042
Total		47736	100.000	100.000

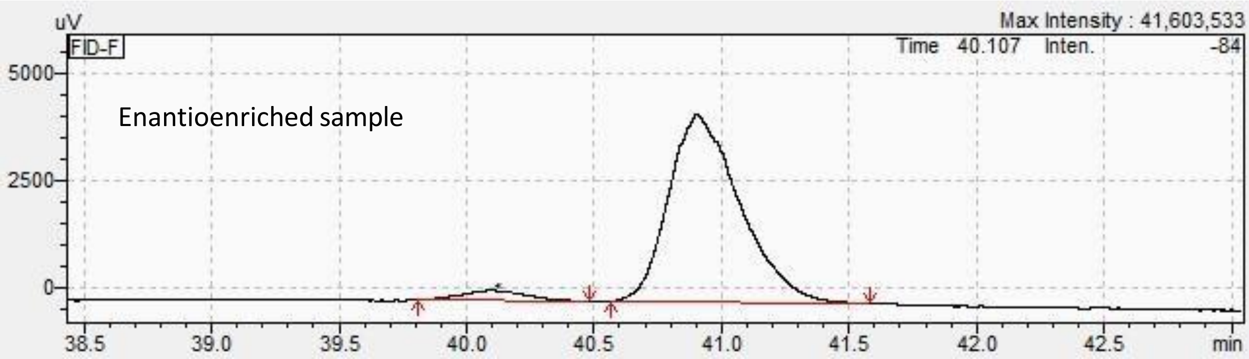

Peak#	Ret. Time	Area	Conc.	Area%
1	40.091	4244	4.767	4.767
2	40.903	84770	95.233	95.233
Total		89014	100.000	100.000

(8S,9S,10R,13R,14S,17R)-10,13-Dimethyl-17-((R)-6-methylheptan-2-yl)-2,3,4,7,8,9,10,11,12,13,14,15,16,17-tetradecahydro-1H-cyclopenta[a]phenanthren-3-yl (2S)-2-methyloctanoate (3ax):

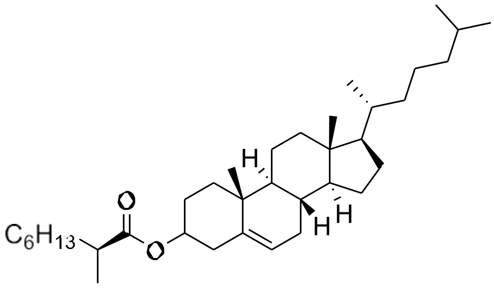

The compound was prepared according to the general procedure by reaction of 1-octene (79 µL, 0.50 mmol) and cholesterol (97 mg, 0.25 mmol), and was isolated by column chromatography (silica gel, petroleum ether/dichloromethane = 8/2), yielding the title product as a white solid (82 mg, 62%). Slow evaporation of the solution of 3au in $CHCl_3$ led to the formation of crystals suitable for X-ray crystallography.

^{1}H NMR (500 MHz, $CDCl_3$) δ 5.40 (d, *J* = 4.3 Hz, 1H), 4.67 – 4.59 (m, 1H), 2.40 (q, *J* = 7.0 Hz, 1H), 2.33 (d, *J* = 7.8 Hz, 2H), 2.05 – 1.97 (m, 1H), 1.89 – 1.84 (m, 1H), 1.69 – 1.25 (m, 20H), 1.22 – 0.95 (m, 17H), 0.94 (d, *J* = 6.4 Hz, 3H), 0.92 – 0.86 (m, 12H), 0.70 (s, 3H).

^{13}C-{^{1}H} NMR (176 MHz, MeOD) δ 178.2, 141.0, 123.7, 75.2, 58.1, 57.6, 51.6, 43.5, 41.1, 41.0, 40.7, 39.3, 38.2, 37.8, 37.4, 37.1, 35.0, 33.2, 33.0, 32.9, 30.2, 29.3, 29.2, 28.8, 28.3, 25.3, 24.9, 23.6, 23.2, 22.9, 22.1, 19.7, 19.2, 17.6, 14.4, 12.3.

HRMS (APPI) m/z calcd. for $C_{36}H_{63}O_2$ ($[M+H]^+$): 527.4828; found: 527.4818.

96:4 dr, $[\alpha]_D^{25}$ = +1.9 (c = 1.3, $CHCl_3$).

To determine the diastereomeric ratio, the isolated ester was reduced to the alcohol according to the procedure in section 5. GC analysis (CycloSil-B Column 25 m x 0.25 mm x 0.25 µm, flow: 3.16 mL/min, 90 °C): t_1 = 40.14 min (minor), t_2 = 40.92 min (major). The absolute configuration was assigned unambiguously by the X-ray crystallography, enabling to assign the absolute configuration of **3ax** as (*S*)-enantiomer.

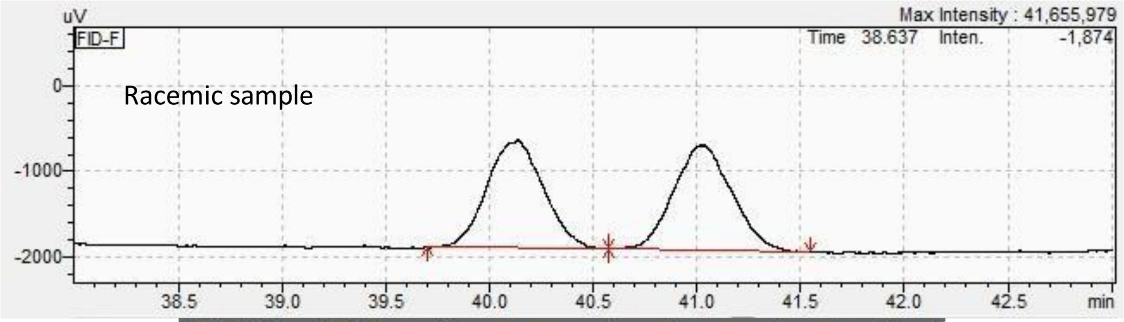

Peak#	Ret. Time	Area	Conc.	Area%
1	40.138	23848	49.958	49.958
2	41.034	23888	50.042	50.042
Total		47736	100.000	100.000

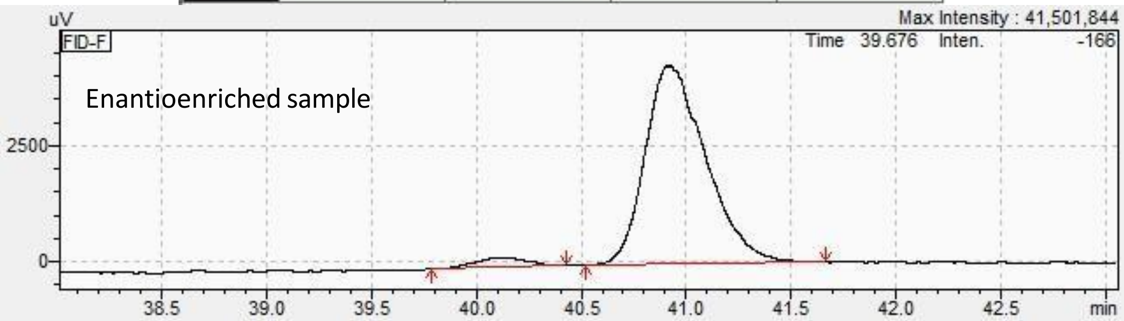

Peak#	Ret. Time	Area	Conc.	Area%
1	40.136	3553	3.964	3.964
2	40.920	86068	96.036	96.036
Total		89621	100.000	100.000

Ethyl (S)-2-methylpentanoate (3ba):

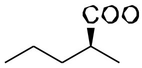

The compound was prepared according to the general procedure by reaction of 1-pentene (55 µL, 0.50 mmol) and ethanol (15 µL, 0.25 mmol), and was isolated by column chromatography (silica gel, petroleum ether/dichloromethane = 9/1), yielding the title product as a colourless oil (27 mg, 75%). The NMR data match those reported in the literature for a racemic product.[3]

^{1}H NMR (500 MHz, CDCl$_3$) δ 4.12 (td, *J* = 7.1, 0.8 Hz, 1H), 2.43 (h, *J* = 7.0 Hz, 1H), 1.66 – 1.60 (m, 1H), 1.36 – 1.32 (m, 1H), 1.29 – 1.22 (m, 6H), 1.13 (d, *J* = 7.0 Hz, 3H), 0.90 (t, *J* = 7.2 Hz, 3H).

^{13}C-{^{1}H} NMR (126 MHz, CDCl$_3$) δ 177.1, 60.2, 39.5, 36.1, 20.6, 17.2, 14.4, 14.1.

92:8 er, $[\alpha]_D^{25}$ = -2.8 (c = 0.9, CHCl$_3$).

To determine the enantiomeric ratio, the isolated ester was reduced to the alcohol according to the procedure in section 5. GC analysis (CycloSil-B Column 25 m x 0.25 mm x 0.25 µm, flow: 3.16 mL/min, 70 °C): t_1 = 10.02 min (minor), t_2 = 10.34 min (major). The absolute configuration of **3ba** was assigned as (*S*)-enantiomer by analogy to *(S)*-**3aa**.

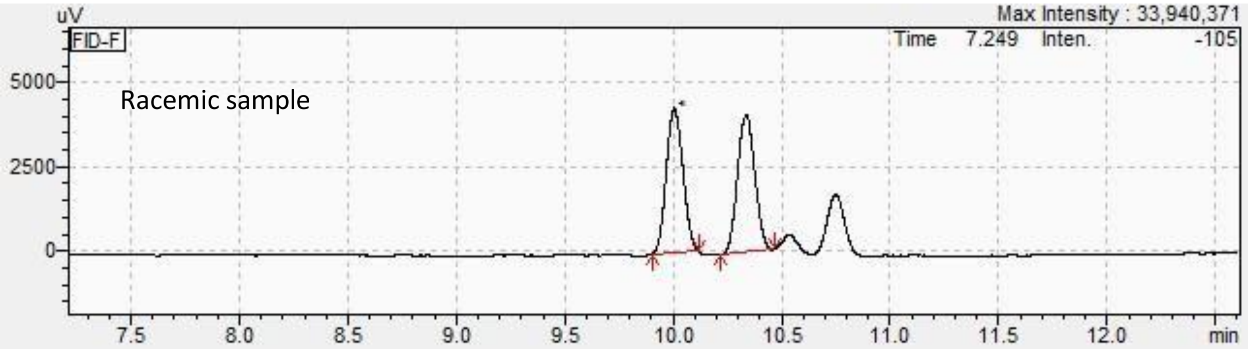

Peak#	Ret. Time	Area	Conc.	Area%
1	10.004	23016	50.446	50.446
2	10.338	22609	49.554	49.554
Total		45626	100.000	100.000

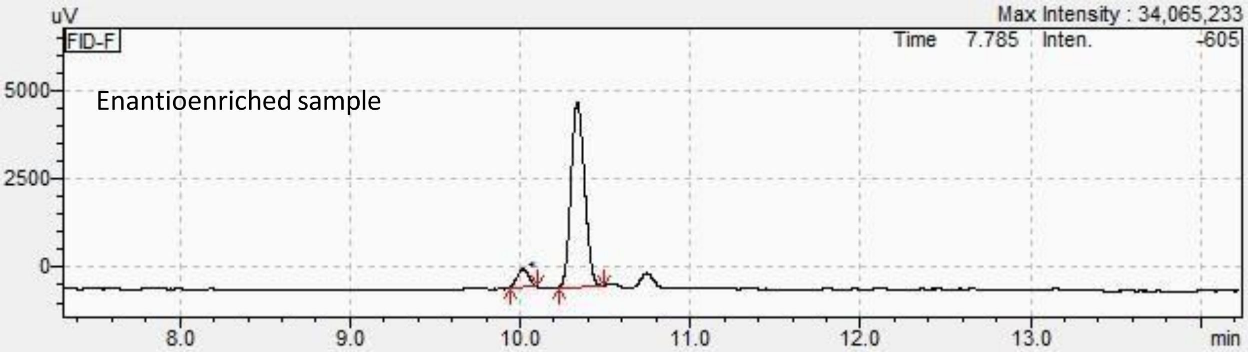

Peak#	Ret. Time	Area	Conc.	Area%
1	10.017	2411	7.700	7.700
2	10.335	28901	92.300	92.300
Total		31312	100.000	100.000

Ethyl (S)-2-methyldecanoate (3ca):

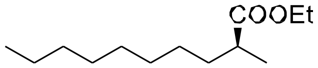

The compound was prepared according to the general procedure by reaction of 1-decene (95 µL, 0.50 mmol) and ethanol (15 µL, 0.25 mmol), and was isolated by column chromatography (silica gel, petroleum ether/dichloromethane = 9/1), yielding the title product as a colourless oil (45 mg, 84%). The NMR data match those reported in the literature for a racemic product.[3]

^{1}H NMR (500 MHz, $CDCl_3$) δ 4.12 (qd, *J* = 7.1, 0.8 Hz, 2H), 2.40 (h, *J* = 7.0 Hz, 1H), 1.67 – 1.61 (m, 1H), 1.42 – 1.34 (m, 1H), 1.29 – 1.22 (m, 15H), 1.13 (d, *J* = 7.0 Hz, 3H), 0.88 (t, *J* = 6.9 Hz, 3H).

^{13}C-{^{1}H} NMR (126 MHz, $CDCl_3$) δ 177.2, 60.2, 39.7, 34.0, 32.0, 29.7, 29.6, 29.4, 27.4, 22.8, 17.2, 14.4, 14.3.

97:3 er, $[\alpha]_D^{25}$ = +4.2 (c = 1.9, $CHCl_3$).

GC analysis (CP-Chirasil-Dex CB Column 25 m x 0.25 mm x 0.25 µm, flow: 2.84 mL/min, 90 °C): t_1 = 44.07 min (major), t_2 = 45.11 min (minor). The absolute configuration of **3ca** was assigned as (*S*)-enantiomer by analogy to *(S)*-**3aa**.

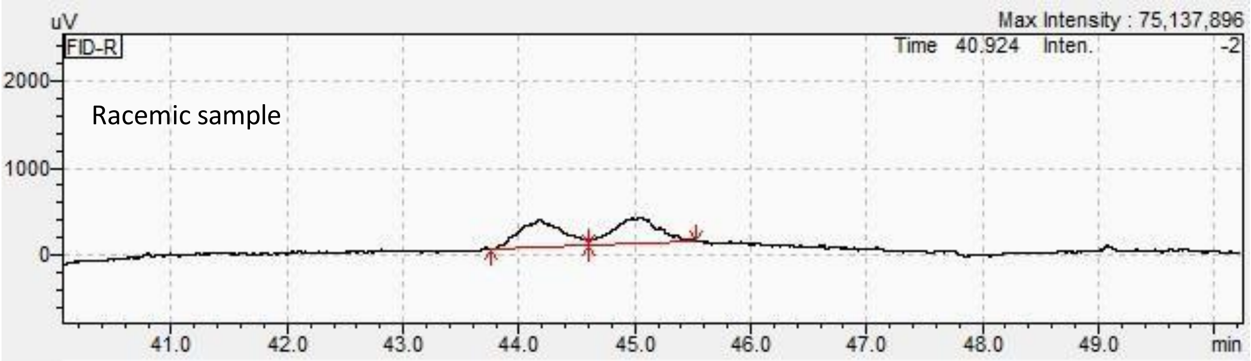

Peak#	Ret. Time	Area	Conc.	Area%
1	44.174	8430	50.039	50.039
2	45.046	8416	49.961	49.961
Total		16846	100.000	100.000

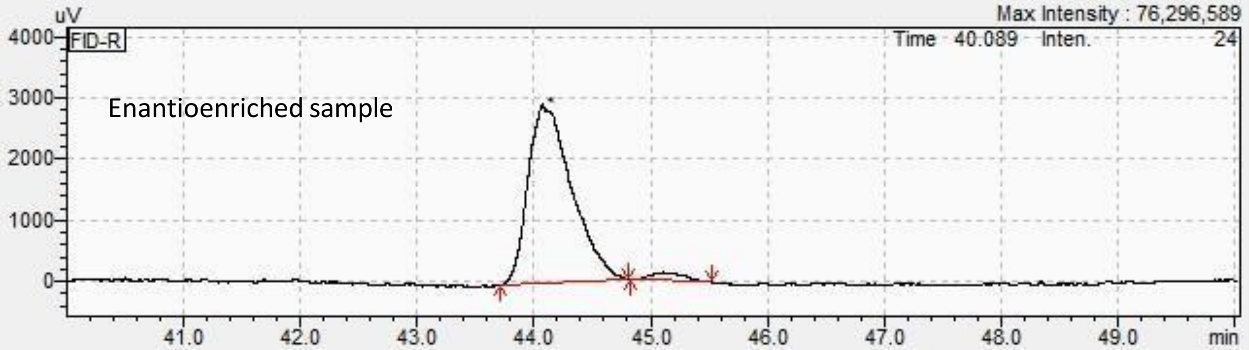

Peak#	Ret. Time	Area	Conc.	Area%
1	44.074	75500	96.720	96.720
2	45.107	2560	3.280	3.280
Total		78061	100.000	100.000

Ethyl (S)-2,4,4-trimethylpentanoate (3da):

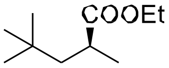

The compound was prepared according to the general procedure by reaction of 4,4-dimethylpent-1-ene (72 µL, 0.50 mmol) and ethanol (15 µL, 0.25 mmol), and was isolated by column chromatography (silica gel, petroleum ether/dichloromethane = 9/1), yielding the title product as a colourless oil (37 mg, 86%). The NMR data match those reported in the literature for the racemic product.[3]

^{1}H NMR (500 MHz, CDCl$_3$) δ 4.11 (qd, *J* = 7.1, 1.3 Hz, 2H), 2.53 – 2.44 (m, 1H), 1.85 (dd, *J* = 14.1, 9.2 Hz, 1H), 1.28 – 1.23 (m, 4H), 1.15 (d, *J* = 7.1 Hz, 3H), 0.88 (s, 9H).

^{13}C-{^{1}H} NMR (126 MHz, CDCl$_3$) δ 178.1, 60.3, 47.9, 36.4, 30.9, 29.6, 20.6, 14.3.

95:5 er, $[\alpha]_D^{25}$ = +0.9 (c = 1.1, CHCl$_3$).

GC analysis (CP-Chirasil-Dex CB Column 25 m x 0.25 mm x 0.25 µm, flow: 2.84 mL/min, 75 °C): t_1 = 6.60 min (minor), t_2 = 6.87 min (major). The absolute configuration of **3da** was assigned as (*S*)-enantiomer by analogy to *(S)*-**3aa**.

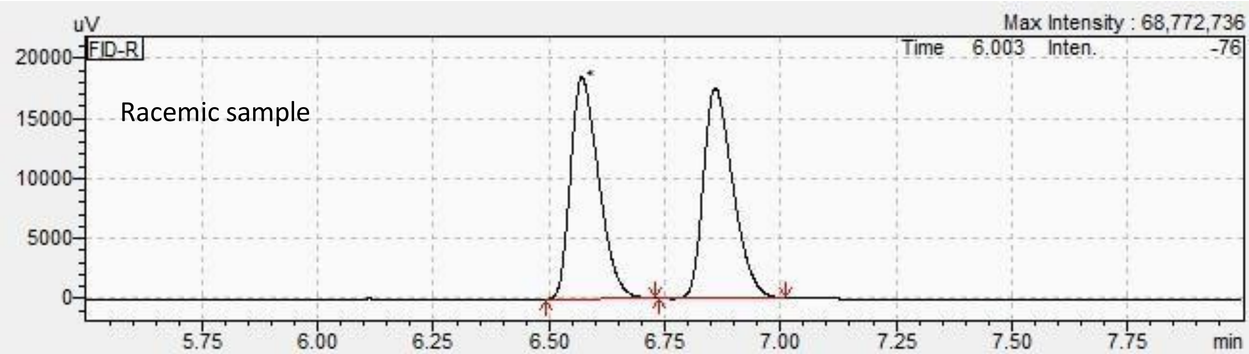

Peak#	Ret. Time	Area	Conc.	Area%
1	6.571	78284	50.173	50.173
2	6.859	77744	49.827	49.827
Total		156028	100.000	100.000

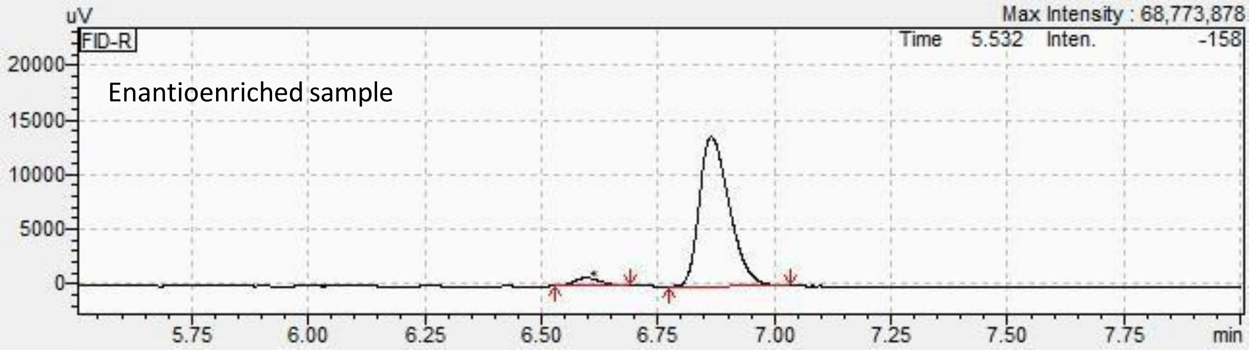

Peak#	Ret. Time	Area	Conc.	Area%
1	6.597	2994	4.677	4.677
2	6.865	61025	95.323	95.323
Total		64019	100.000	100.000

Ethyl (S)-2-cyclohexylpropanoate (3ea):

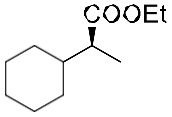

The compound was prepared according to the general procedure by reaction of vinyl cyclohexane (64 µL, 0.50 mmol) and ethanol (15 µL, 0.25 mmol), and was isolated by column chromatography (silica gel, petroleum ether/dichloromethane = 9/1), yielding the title product as a colourless oil (34 mg, 74%). The NMR data match those reported in the literature for the racemic product.[3]

^{1}H NMR (500 MHz, $CDCl_3$) δ 4.13 (p, *J* = 6.9 Hz, 2H), 2.22 (p, *J* = 7.1 Hz, 1H), 1.76 – 1.69 (m, 3H), 1.67 – 1.59 (m, 2H), 1.53 – 1.50 (m, 1H), 1.28 – 1.25 (m, 3H), 1.24 – 1.19 (m, 2H), 1.16 – 1.12 (m, 1H), 1.10 (d, *J* = 7.0 Hz, 3H), 1.06 – 0.87 (m, 2H).

^{13}C-{^{1}H} NMR (126 MHz, $CDCl_3$) δ 176.7, 60.1, 45.7, 40.9, 31.3, 29.8, 26.5, 26.5, 26.4, 14.5, 14.2.

95:5 er, $[\alpha]_D^{25}$ = +5.2 (c = 1.2, $CHCl_3$).

GC analysis (CP-Chirasil-Dex CB Column 25 m x 0.25 mm x 0.25 µm, flow: 2.84 mL/min, 80 °C): t_1 = 32.35 min (major), t_2 = 33.53 min (minor). The absolute configuration of **3ea** was assigned as (*S*)-enantiomer by analogy to *(S)*-**3aa**.

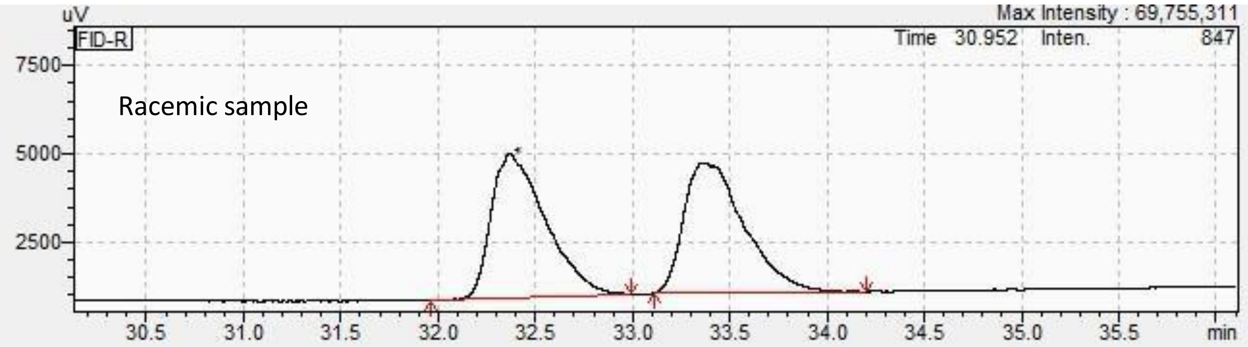

Peak#	Ret. Time	Area	Conc.	Area%
1	32.367	78801	49.950	49.950
2	33.380	78958	50.050	50.050
Total		157759	100.000	100.000

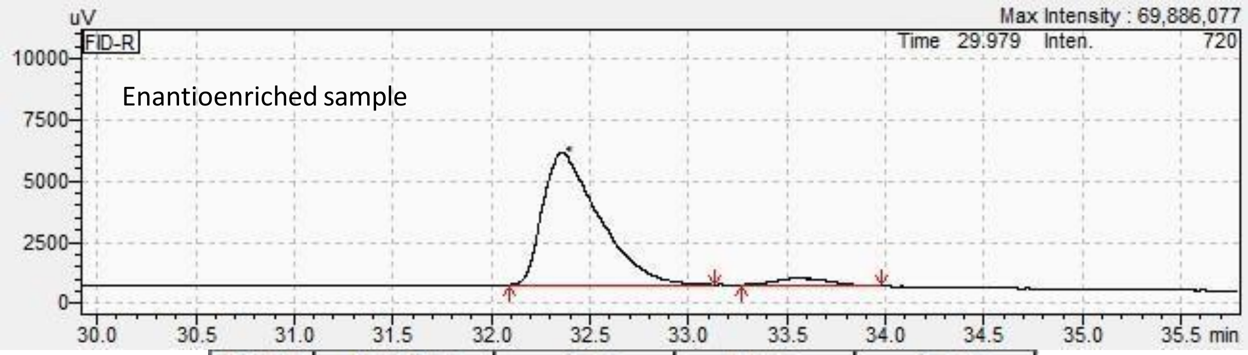

Peak#	Ret. Time	Area	Conc.	Area%
1	32.353	106878	95.084	95.084
2	33.525	5525	4.916	4.916
Total		112404	100.000	100.000

Ethyl (S)-5-bromo-2-methylpentanoate (3fa):

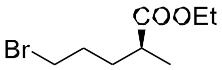

The compound was prepared according to the general procedure by reaction of 5-bromopent-1-ene (59 µL, 0.50 mmol) and ethanol (15 µL, 0.25 mmol), and was isolated by column chromatography (silica gel, petroleum ether/dichloromethane = 7/3), yielding the title product as a red oil (38 mg, 68%). The NMR data match those reported in the literature for a racemic product.[3]

^{1}H NMR (500 MHz, $CDCl_3$) δ 4.14 (q, *J* = 7.1 Hz, 2H), 3.40 (td, *J* = 6.7, 2.3 Hz, 2H), 2.49 – 2.41 (m, 1H), 1.90 – 1.83 (m, 2H), 1.80 – 1.72 (m, 1H), 1.63 – 1.57 (m, 1H), 1.26 (t, *J* = 7.1 Hz, 3H), 1.17 (d, *J* = 7.0 Hz, 3H).

^{13}C-{^{1}H} NMR (126 MHz, $CDCl_3$) δ 176.4, 60.5, 39.0, 33.5, 32.4, 30.6, 17.3, 14.4.

95:5 er, $[\alpha]_D^{25}$ = +3.4 (c = 1.9, $CHCl_3$).

GC analysis (CP-Chirasil-Dex CB Column 25 m x 0.25 mm x 0.25 µm, flow: 2.84 mL/min, 78 °C): t_1 = 41.38 min (minor), t_2 = 41.96 min (major). The absolute configuration of **3fa** was assigned as (*S*)-enantiomer by analogy to *(S)*-**3aa**.

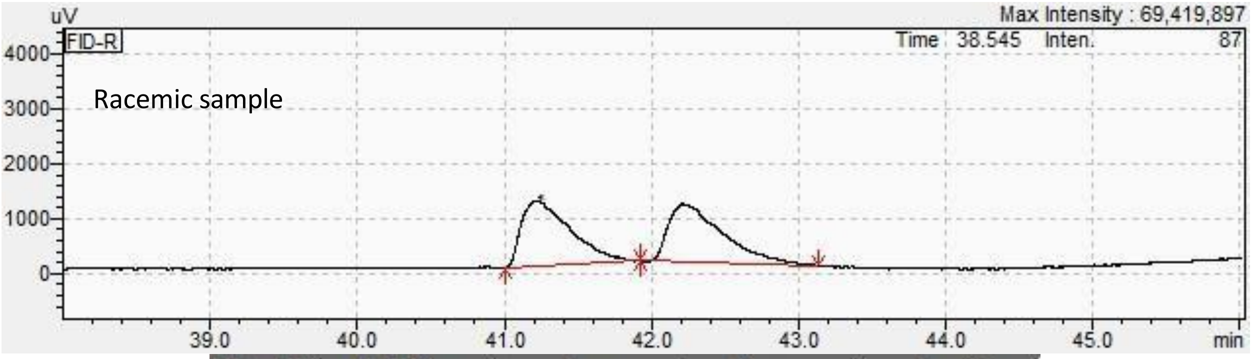

Peak#	Ret. Time	Area	Conc.	Area%
1	41.198	27445	50.240	50.240
2	42.210	27183	49.760	49.760
Total		54627	100.000	100.000

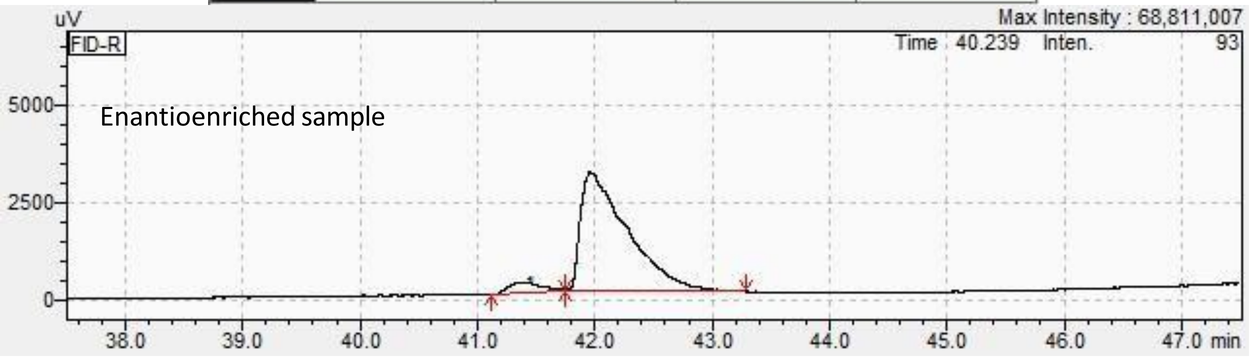

Peak#	Ret. Time	Area	Conc.	Area%
1	41.375	4430	5.049	5.049
2	41.958	83315	94.951	94.951
Total		87746	100.000	100.000

Ethyl (S)-3-(1,3-dioxoisoindolin-2-yl)-2-methylpropanoate (3ga):

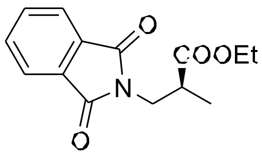

The compound was prepared according to the general procedure by reaction of N-allyl pthalimide (93.6 mg, 0.50 mmol) and ethanol (14.6 µL, 0.25 mmol), $PdBr_2(ACN)_2$ (1.8 mg, 0.005 mmol), **L10** (8.8 mg, 0.01 mmol), in 1,4-dioxane (1 mL), at 45 °C for 48 h, and was isolated by column chromatography (silica gel, petroleum ether/ethyl acetate = 8/2), yielding the title product as a colourless oil (48 mg, 74%).

^{1}H NMR (500 MHz, $CDCl_3$) δ 7.90 – 7.79 (m, 2H), 7.76 – 7.66 (m, 2H), 4.16 – 4.03 (m, 2H), 4.01 – 3.93 (m, 1H), 3.80 – 3.71 (m, 1H), 3.04 – 2.89 (m, 1H), 1.23 – 1.12 (m, 6H).

^{13}C NMR (126 MHz, $CDCl_3$) δ 174.0, 168.3, 134.2, 132.1, 123.5, 61.0, 40.7, 38.6, 14.8, 14.2.

HRMS (APPI) m/z calcd. for $C_{14}H_{16}O_4N$ ([M+H]+): 262.1074; found:262.1068.

87:13 er, $[\alpha]_D^{24.7}$ = +12.3 (c = 0.3, $CHCl_3$).

DAICEL CHIRALPAK IK (CO_2:MeOH 95:5, 2.50 mL min^{-1}, 40 °C) t_1 = 4.21 min (minor), t_2 = 4.63 min (major). The absolute configuration of **3ga** was assigned as (*S*)-enantiomer by analogy to *(S)*-**3aa**.

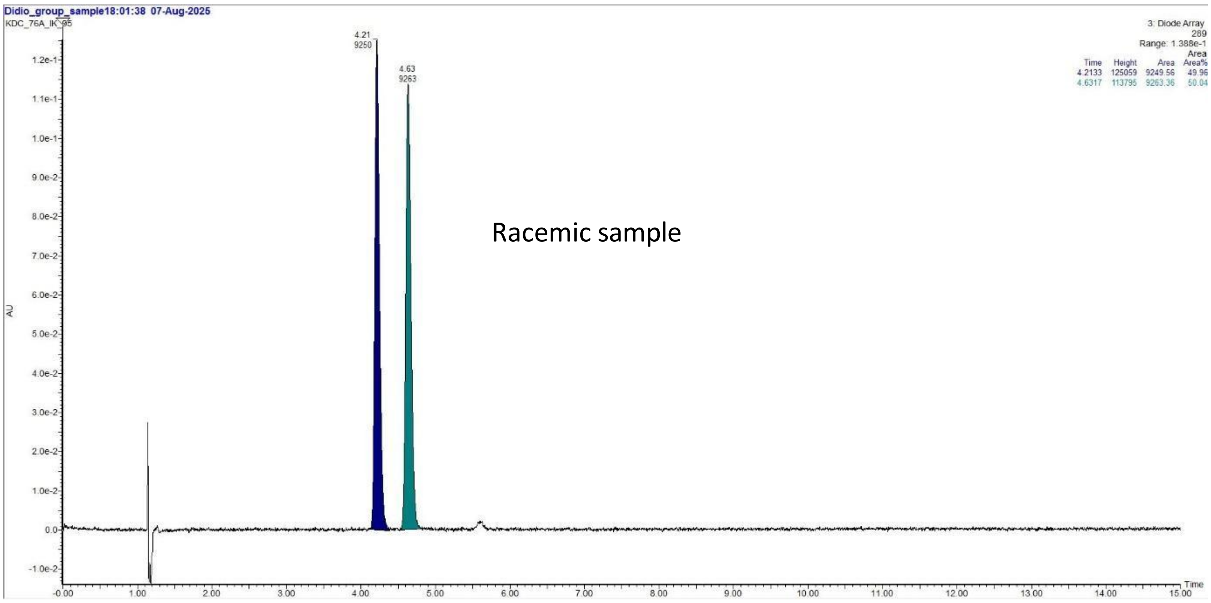

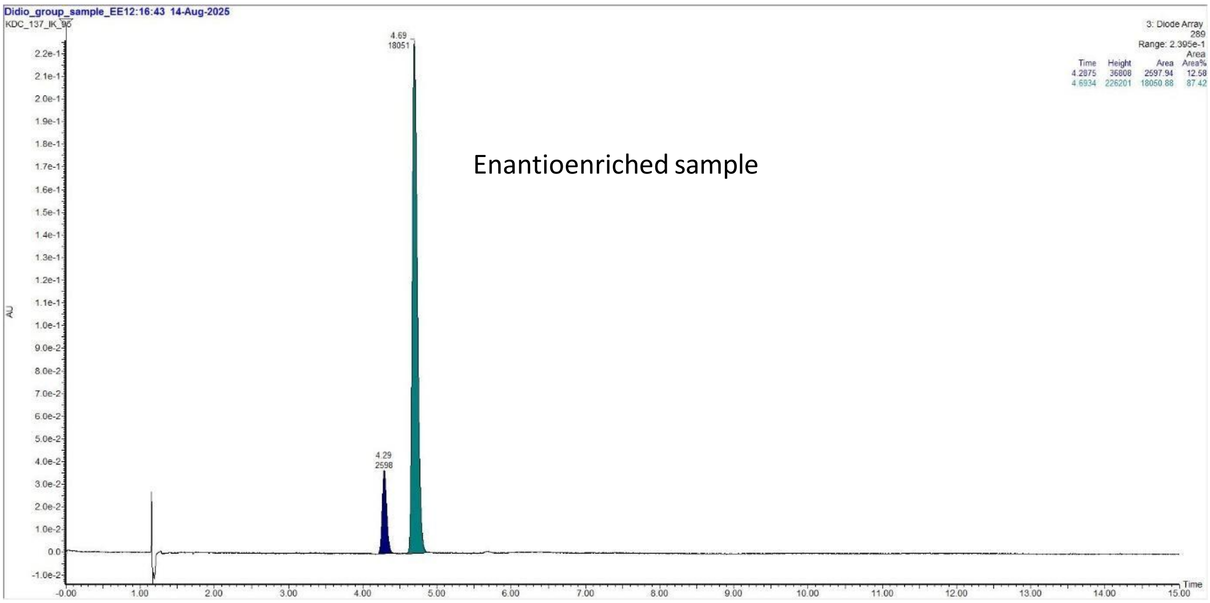

Ethyl (S)-2-methyl-3-phenylpropanoate (3ha):

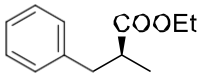

The compound was prepared according to the general procedure by reaction of allyl benzene (66 µL, 0.50 mmol) and ethanol (15 µL, 0.25 mmol), and was isolated by column chromatography (silica gel, petroleum ether/dichloromethane = 8/2), yielding the title product as a colourless oil (32 mg, 67%). The NMR data match those reported in the literature for the racemic product.

^{1}H NMR (500 MHz, CDCl$_3$) δ 7.30 – 7.26 (m, 2H), 7.21 – 7.15 (m, 3H), 4.09 (q, *J* = 7.1 Hz, 2H), 3.02 (dd, *J* = 13.0, 6.6 Hz, 1H), 2.75 – 2.63 (m, 2H), 1.19 (t, *J* = 7.1 Hz, 3H), 1.15 (d, *J* = 6.7 Hz, 3H).[3]

^{13}C-{^{1}H} NMR (126 MHz, CDCl$_3$) δ 176.27, 139.59, 129.14, 128.46, 126.41, 60.42, 41.65, 39.90, 16.94, 14.31.

92:8 er, $[\alpha]_D^{25}$ = +2.9 (c = 1.6, CHCl$_3$).

GC analysis (CP-Chirasil-Dex CB Column 25 m x 0.25 mm x 0.25 µm, flow: 2.84 mL/min, 90 °C): t_1 = 38.51 min (minor), t_2 = 39.63 min (major). The absolute configuration of **3ha** was assigned as (*S*)-enantiomer by analogy to *(S)*-**3aa**.

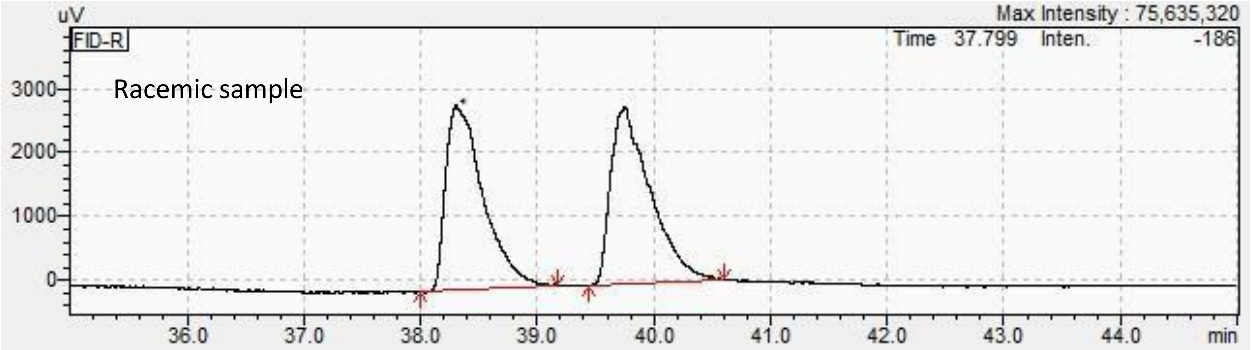

Peak#	Ret. Time	Area	Conc.	Area%
1	38.301	64454	49.689	49.689
2	39.753	65261	50.311	50.311
Total		129715	100.000	100.000

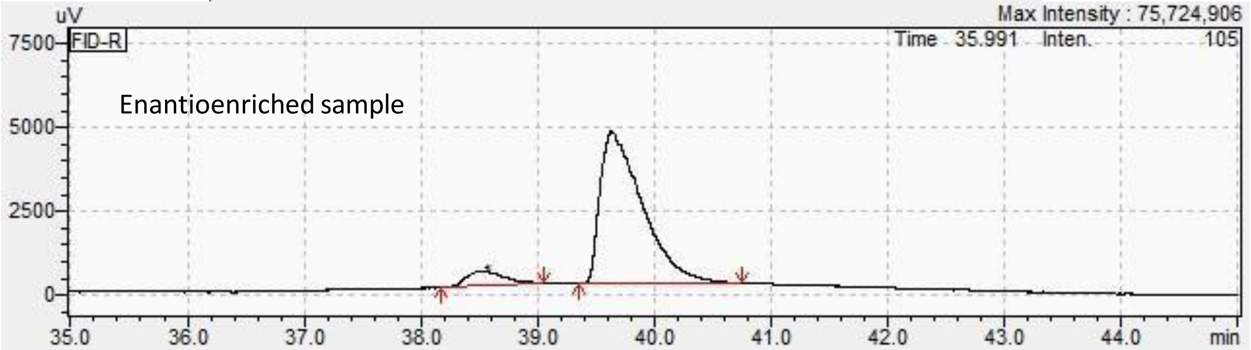

Peak#	Ret. Time	Area	Conc.	Area%
1	38.512	9677	7.884	7.884
2	39.625	113063	92.116	92.116
Total		122740	100.000	100.000

Ethyl (S)-2-methyl-4-phenylbutanoate (3ia):

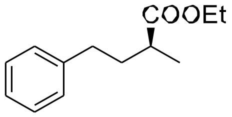

The compound was prepared according to the general procedure by reaction of 4-phenyl-1-butene (75 µL, 0.50 mmol) and ethanol (15 µL, 0.25 mmol), and was isolated by column chromatography (silica gel, petroleum ether/dichloromethane = 8/2), yielding the title product as a colourless oil (47 mg, 91%). The NMR data match those reported in the literature for a racemic product.[3]

^{1}H NMR (500 MHz, $CDCl_3$) δ 7.32 – 7.26 (m, 2H), 7.21 – 7.16 (m, 3H), 4.14 (q, *J* = 7.1 Hz, 2H), 2.62 (t, *J* = 8.0 Hz, 2H), 2.46 (dt, *J* = 14.0, 7.0 Hz, 1H), 2.06 – 1.96 (m, 1H), 1.77 – 1.67 (m, 1H), 1.28 (t, *J* = 7.1 Hz, 3H), 1.19 (d, *J* = 7.0 Hz, 3H).

^{13}C-{^{1}H} NMR (126 MHz, $CDCl_3$) δ 176.7, 141.9, 128.6, 128.5, 126.0, 60.4, 39.2, 35.6, 33.6, 17.3, 14.4.

97:3 er, $[\alpha]_D^{25}$ = +4.0 (c = 1.1, $CHCl_3$).

To determine the enantiomeric ratio, the isolated ester was reduced to the alcohol according to the procedure in section 5. GC analysis (CycloSil-B Column 25 m x 0.25 mm x 0.25 µm, flow: 3.16 mL/min, 95 °C): t_1 = 39.98 min (minor), t_2 = 40.77 min (major). The absolute configuration of **3ia** was assigned as (*S*)-enantiomer by analogy to *(S)*-**3aa**.

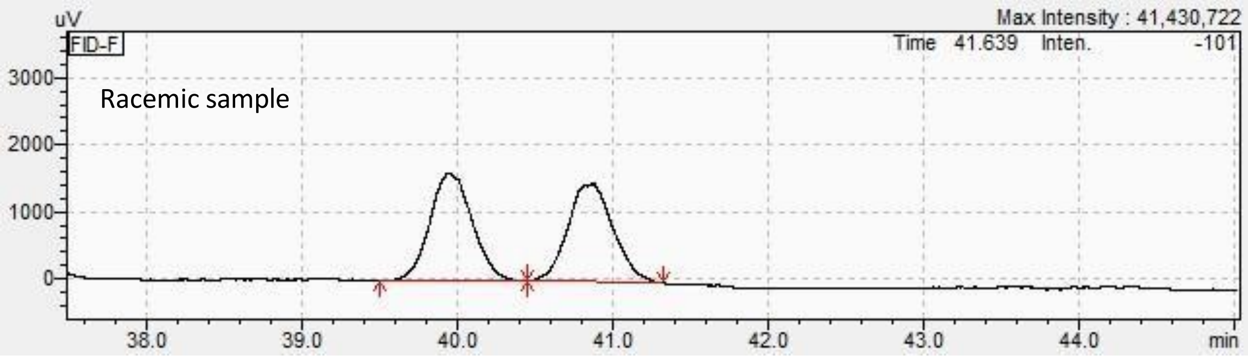

Peak#	Ret. Time	Area	Conc.	Area%
1	39.946	30328	50.440	50.440
2	40.880	29799	49.560	49.560
Total		60127	100.000	100.000

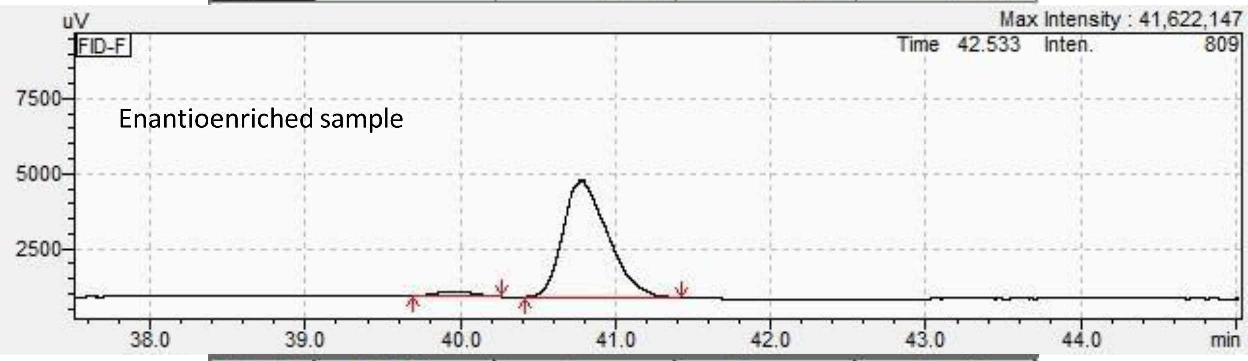

Peak#	Ret. Time	Area	Conc.	Area%
1	39.977	2770	3.432	3.432
2	40.769	77954	96.568	96.568
Total		80724	100.000	100.000

Ethyl (S)-10-chloro-2-methyldecanoate (3ja):

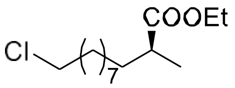

The compound was prepared according to the general procedure by reaction of 11-chloroundec-1-ene (95 mg, 0.50 mmol) and ethanol (15 µL, 0.25 mmol), $PdBr_2(ACN)_2$ (1.8 mg, 0.005 mmol), **L10** (8.8 mg, 0.01 mmol), at 45 °C for 16 h, and was isolated by column chromatography (silica gel, 0-3% EtOAc in *n*-hexane), yielding the title product as a colourless oil (54 mg, 82%).

^{1}H NMR (500 MHz, $CDCl_3$) δ = 4.12 (q, *J* = 7.4 Hz, 2H), 3.53 (t, *J* = 6.8 Hz, 2H), 2.40 (h, *J* = 6.9 Hz, 1H), 1.76 (p, *J* = 7.2 Hz, 2H), 1.68 – 1.59 (m, 1H), 1.45 – 1.22 (m, 16H), 1.13 (d, *J* = 7.0 Hz, 3H).

^{13}C-{^{1}H} NMR (126 MHz, $CDCl_3$) δ = 177.1, 60.2, 45.3, 39.7, 33.9, 32.8, 29.6, 29.5, 29.5, 29.0, 27.3, 27.0, 17.3, 14.4.

HRMS (ESI) m/z calcd. for $C_{14}H_{28}ClO_2$ ($[M+H]^+$): 263.1772; found: 263.1761.

94:6 er, $[\alpha]_D^{24.9}$ = +8.8 (c = 0.4, $CHCl_3$).

GC analysis (CP-Chirasil-Dex CB Column 25 m x 0.25 mm x 0.25 µm, flow: 2.37 mL/min, 120 °C): t_1 = 115.97 min (major), t_2 = 118.37 min (minor). The absolute configuration of **3ja** was assigned as (*S*)-enantiomer by analogy to (*S*)-**3aa**.

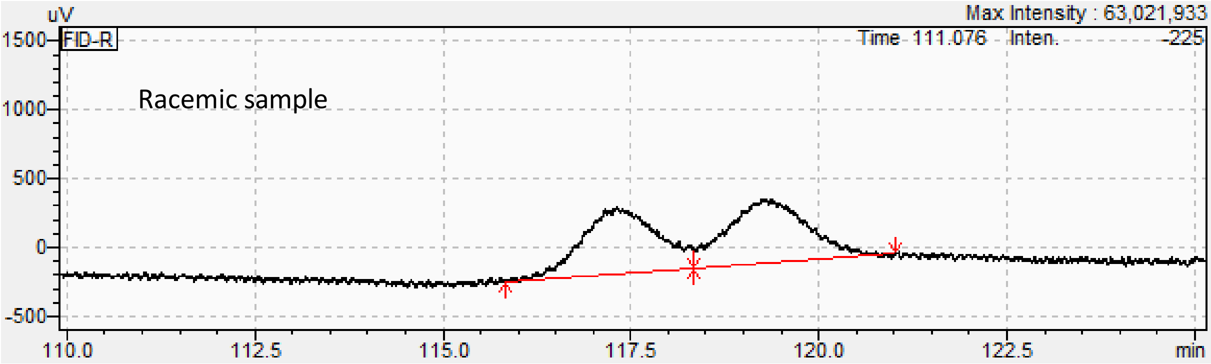

Peak#	Ret. Time	Area	Conc.	Area%
1	117.309	34406	50.126	50.126
2	119.237	34233	49.874	49.874
Total		68638	100.000	100.000

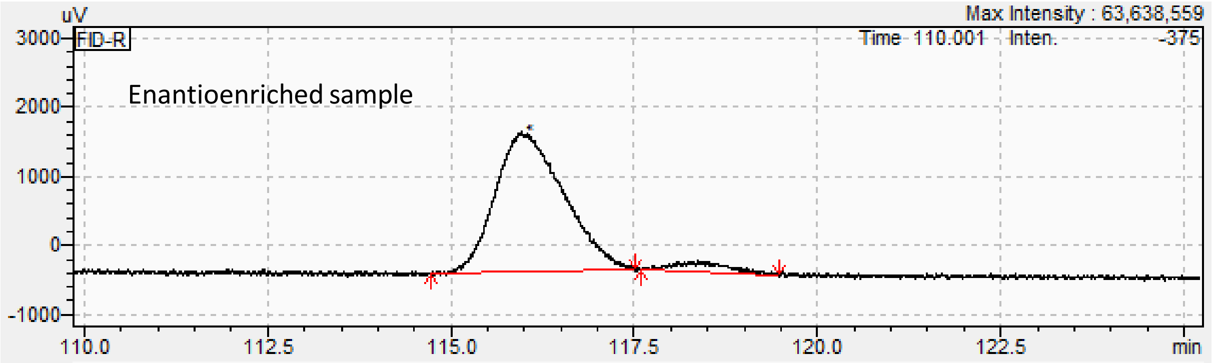

Peak#	Ret. Time	Area	Conc.	Area%
1	115.965	131090	94.221	94.221
2	118.373	8041	5.779	5.779
Total		139131	100.000	100.000

Ethyl (S)-10-bromo-2-methyldecanoate (3ka):

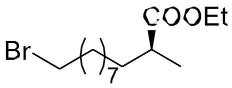

The compound was prepared according to the general procedure by reaction of 11-bromoundec-1-ene (117 mg, 0.50 mmol) and ethanol (15 µL, 0.25 mmol), $PdBr_2(ACN)_2$ (1.8 mg, 0.005 mmol), **L10** (8.8 mg, 0.01 mmol), at 45 °C for 16 h, and was isolated by column chromatography (silica gel, 0-3% EtOAc in *n*-hexane), yielding the title product as a colourless oil (69 mg, 90%).

^{1}H NMR (500 MHz, $CDCl_3$) δ = 4.12 (q, *J* = 7.2 Hz, 2H), 3.40 (t, *J* = 6.9 Hz, 2H), 2.40 (h, *J* = 7.0 Hz, 1H), 1.85 (p, *J* = 6.9 Hz, 2H), 1.68 – 1.59 (m, 1H), 1.45 – 1.22 (m, 16H), 1.13 (d, *J* = 7.0 Hz, 3H).

^{13}C-{^{1}H} NMR (126 MHz, $CDCl_3$) δ = 177.1, 60.2, 39.7, 34.2, 33.9, 33.0, 29.6, 29.5, 29.5, 28.9, 28.3, 27.3, 17.3, 14.4.

HRMS (ESI) m/z calcd. for $C_{14}H_{28}BrO_2$ ($[M+H]^+$): 307.1267; found: 307.1266.

94:6 er, $[\alpha]_D^{25.2}$ = +8.8 (c = 0.4, $CHCl_3$).

GC analysis (CP-Chirasil-Dex CB Column 25 m x 0.25 mm x 0.25 µm, flow: 2.35 mL/min, 125 °C): t_1 = 138.56 min (major), t_2 = 140.69 min (minor). The absolute configuration of **3ka** was assigned as (*S*)-enantiomer by analogy to (*S*)-**3aa**.

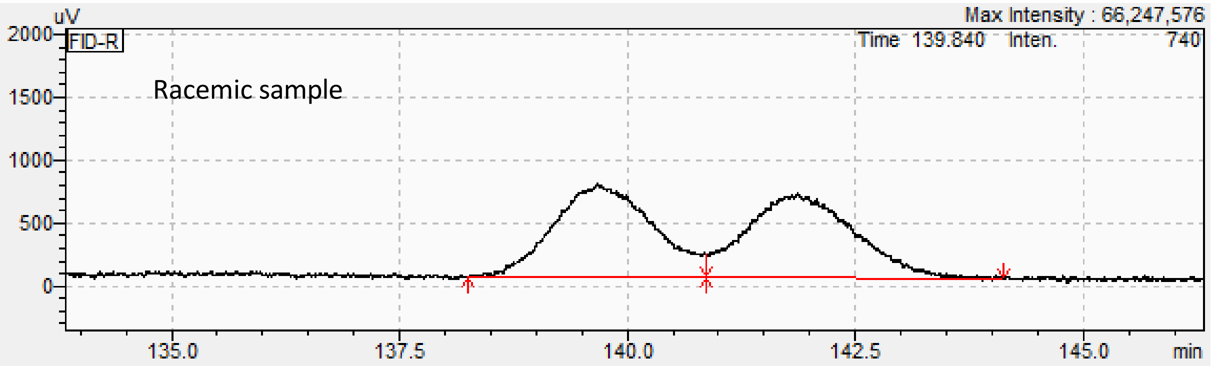

Peak#	Ret. Time	Area	Conc.	Area%
1	139.666	56103	50.234	50.234
2	141.864	55581	49.766	49.766
Total		111684	100.000	100.000

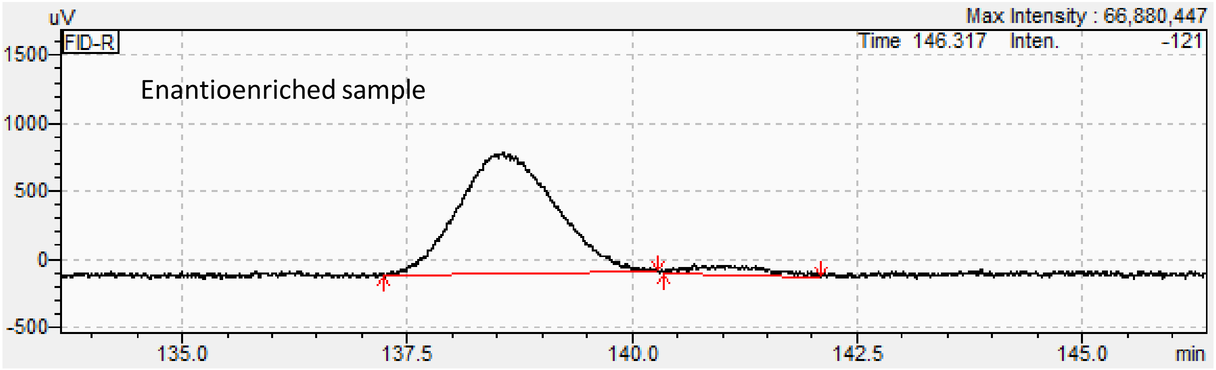

Peak#	Ret. Time	Area	Conc.	Area%
1	138.564	65749	94.154	94.154
2	140.690	4083	5.846	5.846
Total		69832	100.000	100.000

Ethyl (S)-11-((tert-butyldiphenylsilyl)oxy)-2-methylundecanoate (3la):

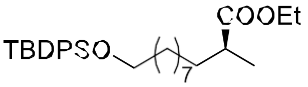

The compound was prepared according to the general procedure by reaction of *tert*-butyldiphenyl(undec-10-en-1-yloxy)silane (204 mg, 0.50 mmol) and ethanol (15 µL, 0.25 mmol), $PdBr_2(ACN)_2$ (1.8 mg, 0.005 mmol), **L10** (8.8 mg, 0.01 mmol), at 45 °C for 16 h, and was isolated by column chromatography (silica gel, 0-3% EtOAc in *n*-hexane), yielding the title product as a colourless oil (113 mg, 94%).

^{1}H NMR (500 MHz, $CDCl_3$) δ = 7.68 – 7.65 (m, 4H), 7.44 – 7.35 (m, 6H), 4.13 (q, *J* = 7.2 Hz, 2H), 3.65 (t, *J* = 6.5 Hz, 2H), 2.41 (h, *J* = 7.0 Hz, 1H), 1.68 – 1.60 (m, 1H), 1.58 – 1.51 (m, 3H), 1.43 – 1.21 (m, 15H), 1.14 (d, *J* = 7.0 Hz, 3H), 1.05 (s, 9H).

^{13}C-{^{1}H} NMR (126 MHz, $CDCl_3$) δ = 177.1, 135.7, 134.3, 129.6, 127.7, 64.2, 60.2, 39.7, 34.0, 32.7, 29.7, 29.7, 29.6, 29.5, 27.4, 27.0, 25.9, 19.4, 17.2, 14.4.

HRMS (ESI) m/z calcd. for $C_{30}H_{47}O_3Si$ ($[M+H]^+$): 483.3289; found: 483.3289.

95:5 er, $[\alpha]_D^{24.7}$ = +5.3 (c = 0.4, $CHCl_3$).

To determine the enantiomeric ratio, the silyl group of the isolated product was deprotected by treatment with TBAF (~10 equiv), affording the corresponding alcohol, as confirmed by GC–MS analysis. GC analysis (CP-Chirasil-Dex CB Column 25 m x 0.25 mm x 0.25 µm, flow: 2.35 mL/min, 125 °C): t_1 = 170.91 min (major), t_2 = 173.50 min (minor). The absolute configuration of **3la** was assigned as (*S*)-enantiomer by analogy to (*S*)-**3aa**.

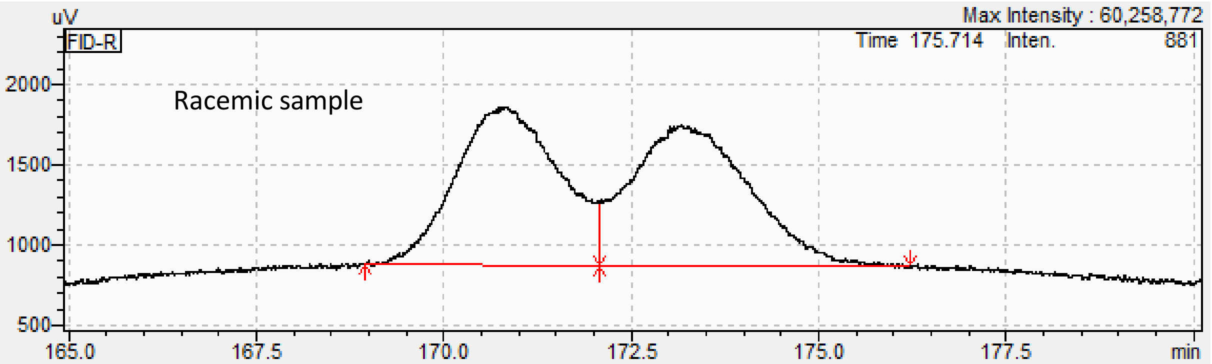

Peak#	Ret. Time	Area	Conc.	Area%
1	170.825	94842	49.617	49.617
2	173.178	96305	50.383	50.383
Total		191147	100.000	100.000

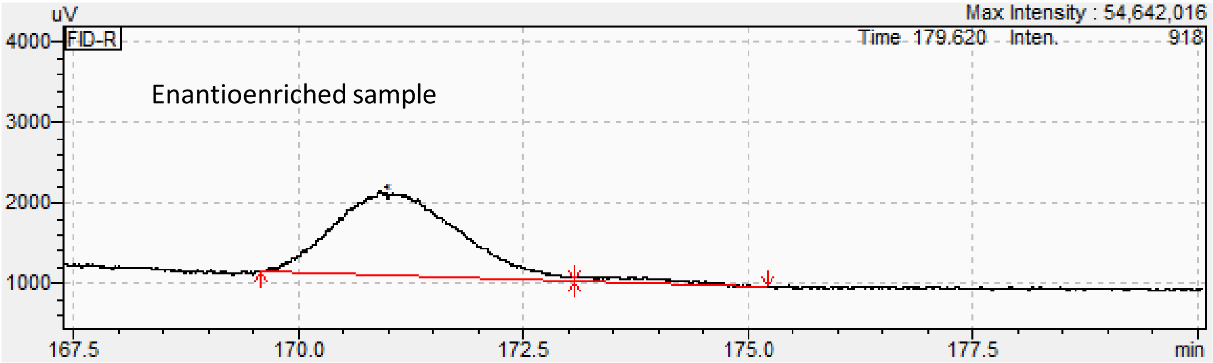

Peak#	Ret. Time	Area	Conc.	Area%
1	170.910	100255	94.850	94.850
2	173.496	5444	5.150	5.150
Total		105699	100.000	100.000

Ethyl (S)-2-methyl-11-((triisopropylsilyl)oxy)undecanoate (3la'):

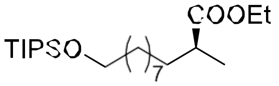

The compound was prepared according to the general procedure by reaction of triisopropyl(undec-10-en-1-yloxy)silane (164 mg, 0.50 mmol) and ethanol (15 µL, 0.25 mmol), $PdBr_2(ACN)_2$ (1.8 mg, 0.005 mmol), **L10** (8.8 mg, 0.01 mmol), at 45 °C for 16 h, and was isolated by column chromatography (silica gel, 0-3% EtOAc in *n*-hexane), yielding the title product as a colourless oil (87 mg, 87%).

^{1}H NMR (500 MHz, $CDCl_3$) δ = 4.12 (q, *J* = 7.1 Hz, 2H), 3.66 (t, *J* = 6.7 Hz, 2H), 2.40 (h, *J* = 7.0 Hz, 1H), 1.67 – 1.50 (m, 4H), 1.41 – 1.23 (m, 15H), 1.13 (d, *J* = 7.0 Hz, 3H), 1.11 – 1.02 (m, 21H).

^{13}C-{^{1}H} NMR (126 MHz, $CDCl_3$) δ = 177.1, 63.7, 60.2, 39.7, 34.0, 33.2, 29.7, 29.7, 29.6, 29.6, 27.4, 26.0, 18.2, 17.2, 14.4, 12.2.

HRMS (ESI) m/z calcd. for $C_{23}H_{49}O_3Si$ ($[M+H]^+$): 401.3445; found: 401.3437.

93:7 er, $[\alpha]_D^{23.7}$ = +5.4 (c = 0.3, $CHCl_3$).

To determine the enantiomeric ratio, the silyl group of the isolated product was deprotected by treatment with TBAF (~10 equiv), affording the corresponding alcohol, as confirmed by GC–MS analysis. GC analysis (CP-Chirasil-Dex CB Column 25 m x 0.25 mm x 0.25 µm, flow: 2.35 mL/min, 125 °C): t_1 = 170.83 min (major), t_2 = 173.16 min (minor). The absolute configuration of **3la'** was assigned as (*S*)-enantiomer by analogy to (*S*)-**3aa**.

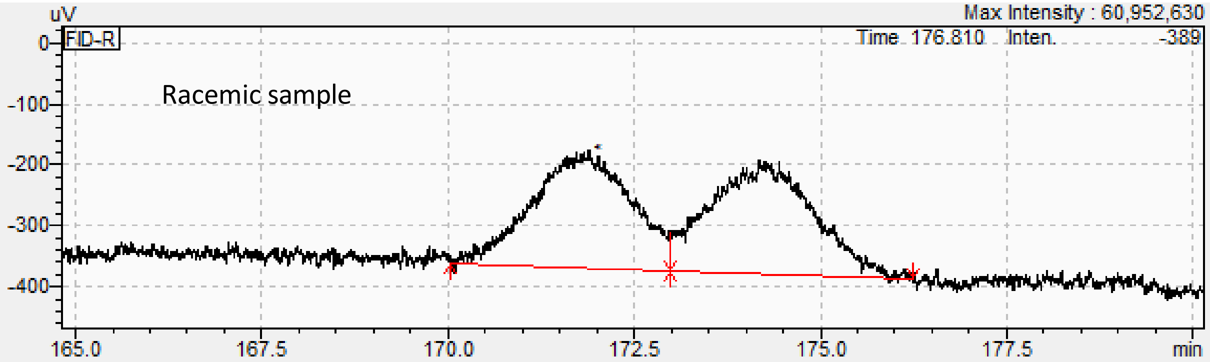

Peak#	Ret. Time	Area	Conc.	Area%
1	171.895	17547	49.683	49.683
2	174.175	17771	50.317	50.317
Total		35318	100.000	100.000

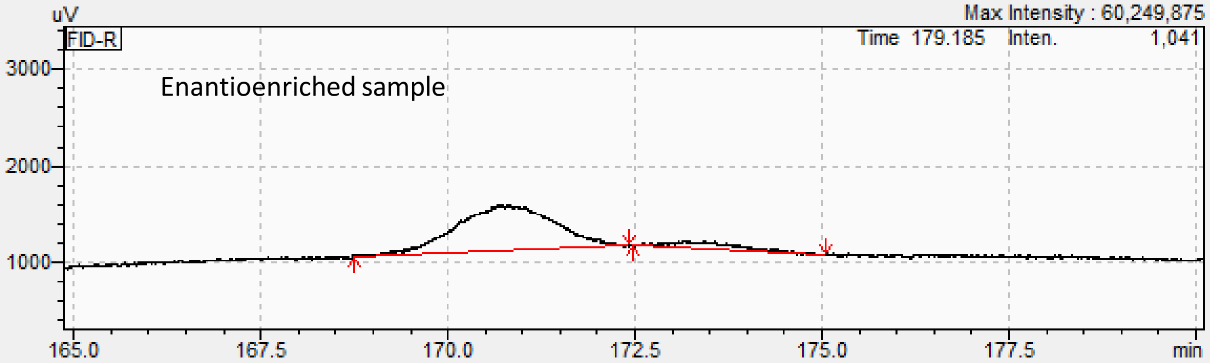

Peak#	Ret. Time	Area	Conc.	Area%
1	170.834	42805	92.750	92.750
2	173.157	3346	7.250	7.250
Total		46150	100.000	100.000

Ethyl (S)-11-(benzyloxy)-2-methylundecanoate (3ma):

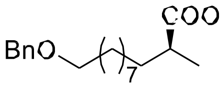

The compound was prepared according to the general procedure by reaction of ((undec-10-en-1-yloxy)methyl)benzene (130 mg, 0.50 mmol) and ethanol (15 µL, 0.25 mmol), $PdBr_2(ACN)_2$ (1.8 mg, 0.005 mmol), **L10** (8.8 mg, 0.01 mmol), at 45 °C for 16 h, and was isolated by column chromatography (silica gel, 0-5% EtOAc in *n*-hexane), yielding the title product as a colourless oil (72 mg, 86%).

^{1}H NMR (500 MHz, $CDCl_3$) δ = 7.35 – 7.32 (m, 4H), 7.30 – 7.26 (m, 1H), 4.50 (s, 2H), 4.12 (q, *J* = 7.1 Hz, 2H), 3.46 (t, *J* = 6.7 Hz, 2H), 2.40 (h, *J* = 7.0 Hz, 1H), 1.67 – 1.58 (m, 3H), 1.42 – 1.23 (m, 16H), 1.13 (d, *J* = 7.0 Hz, 3H).

^{13}C-{^{1}H} NMR (126 MHz, $CDCl_3$) δ = 177.1, 138.9, 128.5, 127.8, 127.6, 73.0, 70.7, 60.2, 39.7, 34.0, 29.9, 29.7, 29.7, 29.6, 29.6, 27.4, 26.3, 17.2, 14.4.

HRMS (ESI) m/z calcd. for $C_{21}H_{35}O_3$ ([M+H]$^+$): 335.2581; found: 335.2566.

94:6 er, $[\alpha]_D^{23.9}$ = +7.3 (c = 0.3, $CHCl_3$).

SFC analysis (CHIRALPAK ID-3, 3 mmø x 100 mmL, CO_2:CH_3CN 95:5, 1.2 mL min^{-1}, 40 °C): t_1 = 4.83 min (minor), t_2 = 6.56 min (major). The absolute configuration of **3ma** was assigned as (*S*)-enantiomer by analogy to (*S*)-**3aa**.

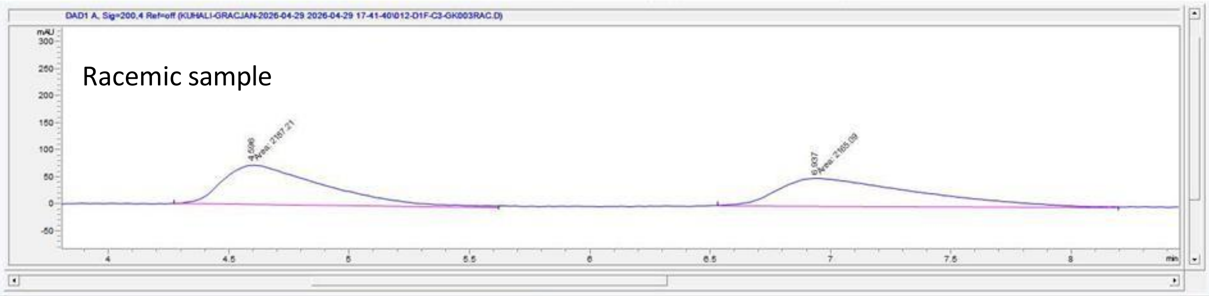

File Information	
LC-File	012-D1F-C3-GK003RAC.D
File Path	C:\Users\Public\Documents\ChemStation\1\Data\KUHALI-GRACJAN
Date	29-Apr-26, 20:40:04
Sample	GK003RAC
Sample Info	
Barcode	
Operator	SYSTEM
Method	Col6_5%ACN_8min_40%ACN_4min_5%ACN_3min.M
Reference	
Analysis Time	14.993 min
Sampling Rate	0.0067 min (0.402 sec), 2250 datapoints

#	Time	Type	Area	Height	Width	Area%	Symmetry
1	4.596	MM	2187.2	73.1	0.4989	50.254	0.404
2	6.937	MM	2165.1	52.6	0.686	49.746	0.373

Activate Windows

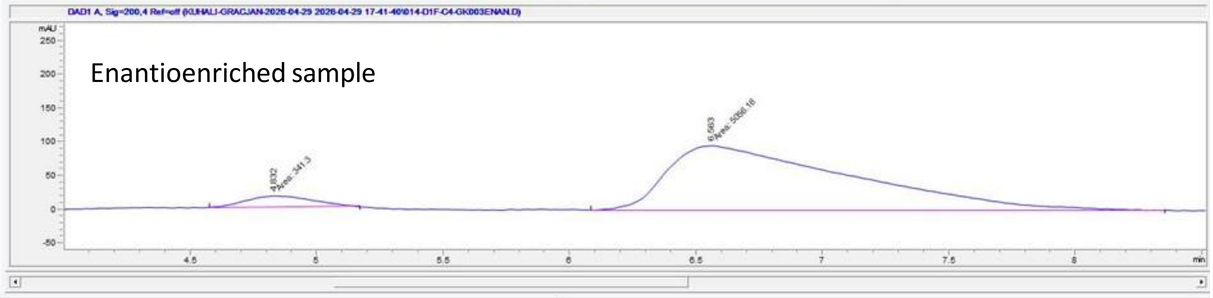

File Information	
LC-File	014-D1F-C4-GK003ENAN.D
File Path	C:\Users\Public\Documents\ChemStation\1\Data\KUHALI-GRACJAN
Date	29-Apr-26, 21:12:35
Sample	GK003ENAN
Sample Info	
Barcode	
Operator	SYSTEM
Method	Col6_5%ACN_8min_40%ACN_4min_5%ACN_3min.M
Reference	
Analysis Time	14.993 min
Sampling Rate	0.0067 min (0.402 sec), 2250 datapoints

#	Time	Type	Area	Height	Width	Area%	Symmetry
1	4.832	MM	341.3	17.8	0.3191	6.323	0.79
2	6.563	MM	5056.2	97.5	0.8643	93.677	0.322

Activate Windows

Ethyl (S)-2-methyl-11-(phenylthio)undecanoate (3na):

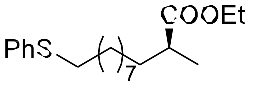

The compound was prepared according to the general procedure by reaction of phenyl(undec-10-en-1-yl)sulfane (131 mg, 0.50 mmol) and ethanol (15 µL, 0.25 mmol), $PdBr_2(ACN)_2$ (1.8 mg, 0.005 mmol), **L10** (8.8 mg, 0.01 mmol), at 45 °C for 16 h, and was isolated by column chromatography (silica gel, 0-5% EtOAc in *n*-hexane), yielding the title product as a colourless oil (80 mg, 95%).

^{1}H NMR (500 MHz, $CDCl_3$) δ = 7.33 – 7.30 (m, 2H), 7.29 – 7.25 (m, 2H), 7.18 – 7.14 (m, 1H), 4.12 (q, *J* = 7.1 Hz, 2H), 2.91 (t, *J* = 7.0 Hz, 2H), 2.40 (h, *J* = 7.0 Hz, 1H), 1.67 – 1.60 (m, 3H), 1.45 – 1.22 (m, 16H), 1.13 (d, *J* = 7.0 Hz, 3H).

^{13}C-{^{1}H} NMR (126 MHz, $CDCl_3$) δ = 177.1, 137.2, 129.0, 128.9, 125.8, 60.2, 39.7, 33.9, 33.7, 29.6, 29.6, 29.5, 29.3, 29.3, 29.0, 27.4, 17.2, 14.4.

HRMS (ESI) m/z calcd. for $C_{20}H_{33}O_2S$ ($[M+H]^+$): 337.2196; found: 337.2196.

91:9 er, $[\alpha]_D^{25.3}$ = +4.8 (c = 0.3, $CHCl_3$).

SFC analysis (CHIRALPAK ID-3, 3 mmø x 100 mmL, CO_2:THF 97.5:2.5, 1.2 mL min^{-1}, 40 °C): t_1 = 6.02 min (minor), t_2 = 6.53 min (major). The absolute configuration of **3na** was assigned as (*S*)-enantiomer by analogy to (*S*)-**3aa**.

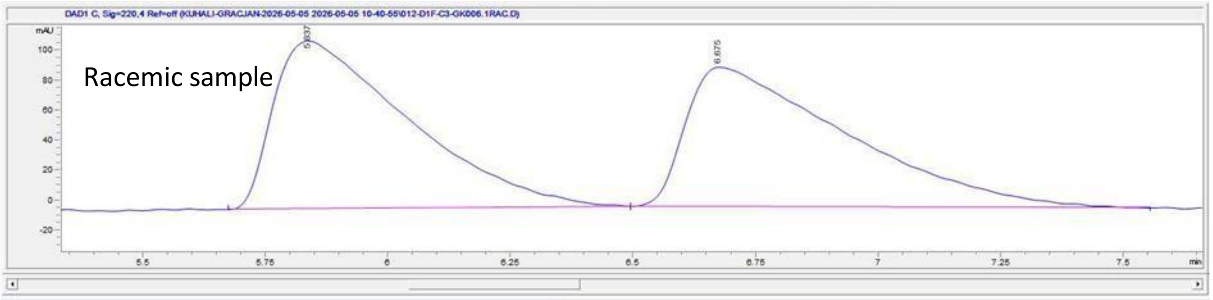

File Information

LC-File	012-D1F-C3-GK006.1RAC.D
File Path	C:\Users\Public\Documents\ChemStation\1\Data\KUHALI-GRACJAN
Date	05-May-26, 13:45:39
Sample	GK006.1RAC
Sample Info	
Barcode	
Operator	SYSTEM
Method	Col6_2.5%THF_12min_40%THF_4min_2.5%THF_3min.M
Reference	
Analysis Time	15.993 min
Sampling Rate	0.0067 min (0.402 sec), 2400 datapoints

#	Time	Type	Area	Height	Width	Area%	Symmetry
1	5.837	BB	2145.8	112.1	0.2613	50.299	0.325
2	6.675	BB	2120.3	93.2	0.3095	49.701	0.252

Activate Windows

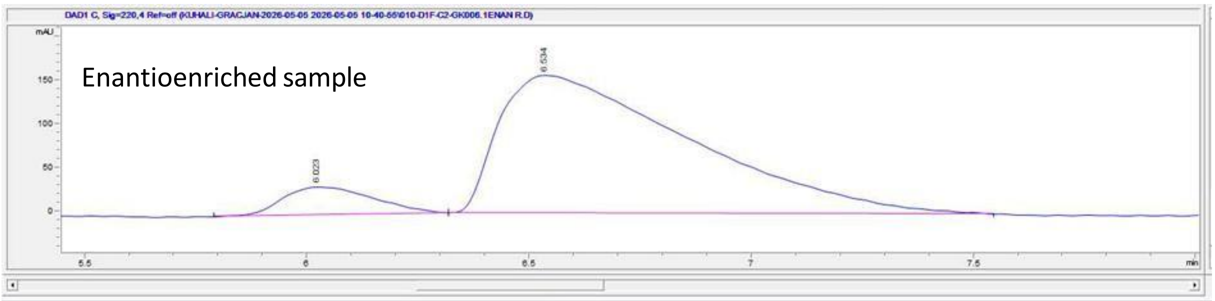

File Information

LC-File	010-D1F-C2-GK006.1ENAN R.D
File Path	C:\Users\Public\Documents\ChemStation\1\Data\KUHALI-GRACJAN
Date	05-May-26, 13:11:33
Sample	GK006.1ENAN R
Sample Info	
Barcode	
Operator	SYSTEM
Method	Col6_2.5%THF_12min_40%THF_4min_2.5%THF_3min.M
Reference	
Analysis Time	15.993 min
Sampling Rate	0.0067 min (0.402 sec), 2400 datapoints

#	Time	Type	Area	Height	Width	Area%	Symmetry
1	6.023	BB	451.1	31.9	0.197	8.742	0.623
2	6.534	BB	4708.7	157.2	0.4075	91.258	0.291

Activate Windows

Ethyl (S)-11-hydroxy-2-methyl-11,11-diphenylundecanoate (3oa):

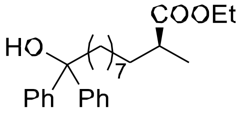

The compound was prepared according to the general procedure by reaction of 1,1-diphenylundec-10-en-1-ol (161 mg, 0.50 mmol) and ethanol (15 µL, 0.25 mmol), $PdBr_2(ACN)_2$ (1.8 mg, 0.005 mmol), **L10** (8.8 mg, 0.01 mmol), at 45 °C for 16 h, and was isolated by column chromatography (silica gel, 0-10% EtOAc in *n*-hexane), yielding the title product as a colourless oil (60 mg, 61%).

^{1}H NMR (500 MHz, C_6D_6) δ = 7.45 – 7.42 (m, 4H), 7.16 – 7.13 (m, 4H), 7.04 (tt, *J* = 6.8, 1.1 Hz, 2H), 3.99 (qd, *J* = 7.1, 1.0 Hz, 2H), 2.38 (h, *J* = 13.9 Hz, 1H), 2.15 – 2.11 (m, 2H), 1.76 – 1.69 (m, 1H), 1.60 (s, 1H), 1.37 – 1.14 (m, 13H), 1.12 (d, *J* = 7.0 Hz, 3H), 0.98 (t, *J* = 7.1 Hz, 3H).

^{13}C-{^{1}H} NMR (126 MHz, C_6D_6) δ = 176.1, 148.1, 128.3, 126.9, 126.6, 78.1, 60.0, 42.5, 39.9, 34.3, 30.5, 29.9, 29.8, 29.8, 27.7, 24.0, 17.4, 14.4.

HRMS (ESI) m/z calcd. for $C_{26}H_{36}NaO_3$ ($[M+Na]^+$): 419.2562; found: 419.2582.

94:6 er, $[\alpha]_D^{24.4}$ = +4.0 (c = 0.3, $CHCl_3$).

SFC analysis (CHIRALPAK ID-3, 3 mmø x 100 mmL, CO_2:CH_3CN 85:15, 1.2 mL min^{-1}, 40 °C): t_1 = 6.84 min (minor), 7.90 min (major). The absolute configuration of **3oa** was assigned as (*S*)-enantiomer by analogy to (*S*)-**3aa**.

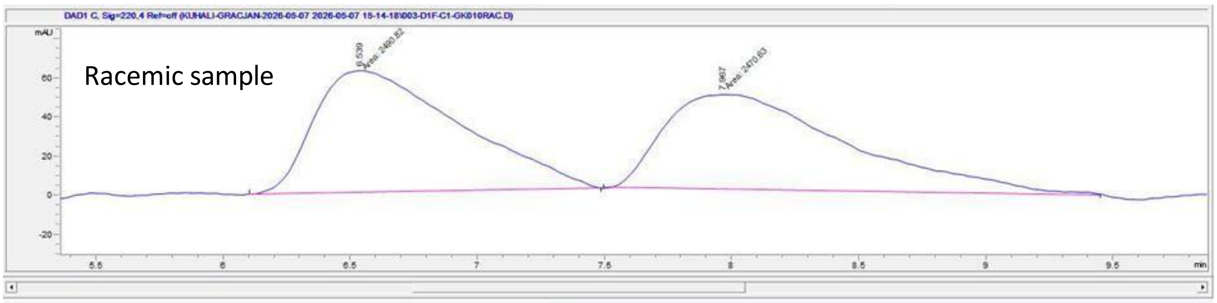

File Information

LC-File	003-D1F-C1-GK010RAC.D
File Path	C:\Users\Public\Documents\ChemStation\1\Data\KUHALI-GRACJAN
Date	07-May-26, 15:49:11
Sample	GK010RAC
Sample Info	
Barcode	
Operator	SYSTEM
Method	Col6_15%ACN_12min_40%ACN_4min_15%ACN_3min.M
Reference	
Analysis Time	15.993 min
Sampling Rate	0.0067 min (0.402 sec), 2400 datapoints

#	Time	Type	Area	Height	Width	Area%	Symmetry
1	6.539	MM	2490.8	62.5	0.6638	50.203	0.456
2	7.967	MM	2470.6	48.7	0.8456	49.797	0.457

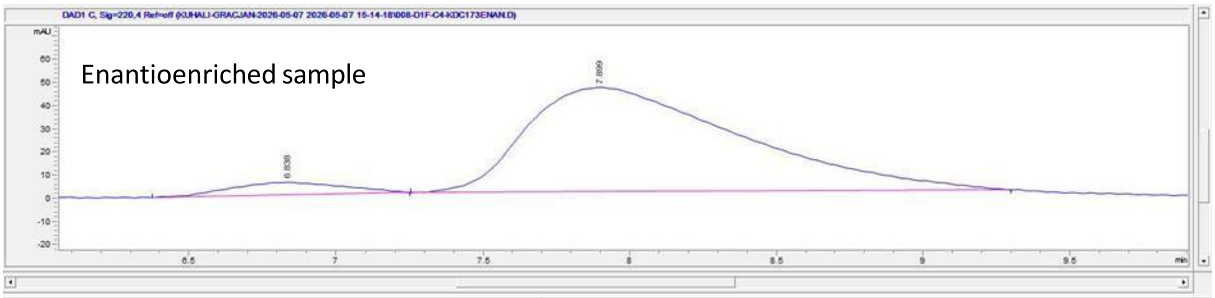

File Information

LC-File	008-D1F-C4-KDC173ENAN.D
File Path	C:\Users\Public\Documents\ChemStation\1\Data\KUHALI-GRACJAN
Date	07-May-26, 17:14:33
Sample	KDC173ENAN
Sample Info	
Barcode	
Operator	SYSTEM
Method	Col6_15%ACN_12min_40%ACN_4min_15%ACN_3min.M
Reference	
Analysis Time	15.993 min
Sampling Rate	0.0067 min (0.402 sec), 2400 datapoints

#	Time	Type	Area	Height	Width	Area%	Symmetry
1	6.838	BB	146	5.5	0.3226	5.980	1.051
2	7.899	BB	2295.5	45	0.6075	94.020	0.504

Ethyl (S)-2-methyl-11-(tosyloxy)undecanoate (3pa):

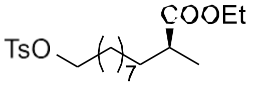

The compound was prepared according to the general procedure by reaction of undec-10-en-1-yl 4-methylbenzenesulfonate (162 mg, 0.50 mmol) and ethanol (15 µL, 0.25 mmol), $PdBr_2(ACN)_2$ (1.8 mg, 0.005 mmol), **L10** (8.8 mg, 0.01 mmol), at 45 ^oC for 16 h, and was isolated by column chromatography (silica gel, 0-10% EtOAc in *n*-hexane), yielding the title product as a colourless oil (91 mg, 91%).

^{1}H NMR (500 MHz, $CDCl_3$) δ = 7.80 – 7.75 (m, 2H), 7.36 – 7.31 (m, 2H), 4.12 (q, *J* = 7.2 Hz, 2H), 4.01 (t, *J* = 6.6 Hz, 2H), 2.44 (s, 3H), 2.43 – 2.35 (m, 1H), 1.66 – 1.58 (m, 3H), 1.41 – 1.33 (m, 1H), 1.29 – 1.17 (m, 15H), 1.12 (d, *J* = 6.9 Hz, 3H).

^{13}C-{^{1}H} NMR (126 MHz, $CDCl_3$) δ = 177.1, 144.7, 133.4, 129.9, 128.0, 70.8, 60.2, 39.7, 33.9, 29.6, 29.4, 29.4, 29.0, 28.9, 27.3, 25.4, 21.8, 17.2, 14.4.

HRMS (ESI) m/z calcd. for $C_{21}H_{35}O_5S$ ($[M+H]^+$): 399.2200; found: 399.2211.

94:6 er, $[\alpha]_D^{23.7}$ = +5.0 (c = 0.4, $CHCl_3$).

SFC analysis (CHIRALPAK ID-3, 3 mmø x 100 mmL, CO_2:CH_3CN 92.5:7.5, 1.2 mL min^{-1}, 40 ^oC): t_1 = 5.87 min (minor), t_2 = 6.63 min (major). The absolute configuration of **3pa** was assigned as (*S*)-enantiomer by analogy to (*S*)-**3aa**.

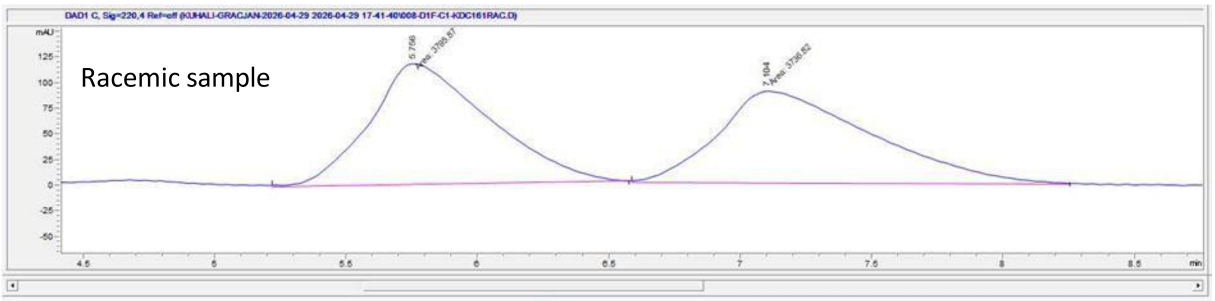

	File Information
LC-File	008-D1F-C1-KDC161RAC.D
File Path	C:\Users\Public\Documents\ChemStation\1\Data\KUHALI-GRACJAN
Date	29-Apr-26, 19:35:55
Sample	KDC161RAC
Sample Info	
Barcode	
Operator	SYSTEM
Method	Col6_7.5%ACN_8min_40%ACN_4min_7.5%ACN_3min.M
Reference	
Analysis Time	14.993 min
Sampling Rate	0.0067 min (0.402 sec), 2250 datapoints

#	Time	Type	Area	Height	Width	Area%	Symmetry
1	5.756	MM	3795.9	119	0.5314	50.392	0.586
2	7.104	MM	3736.8	90.6	0.6874	49.608	0.48

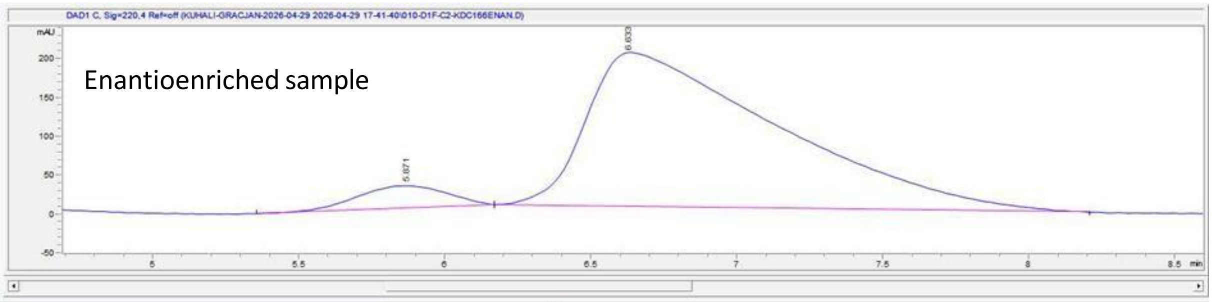

	File Information
LC-File	010-D1F-C2-KDC166ENAN.D
File Path	C:\Users\Public\Documents\ChemStation\1\Data\KUHALI-GRACJAN
Date	29-Apr-26, 20:07:56
Sample	KDC166ENAN
Sample Info	
Barcode	
Operator	SYSTEM
Method	Col6_7.5%ACN_8min_40%ACN_4min_7.5%ACN_3min.M
Reference	
Analysis Time	14.993 min
Sampling Rate	0.0067 min (0.402 sec), 2250 datapoints

#	Time	Type	Area	Height	Width	Area%	Symmetry
1	5.871	BB	616.5	28.5	0.266	6.365	1.319
2	6.633	BB	9068.8	197.9	0.6047	93.635	0.285

Ethyl (S)-10-cyano-2-methyldecanoate (3qa):

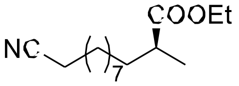

The compound was prepared according to the general procedure by reaction of dodec-11-enenitrile (90 mg, 0.50 mmol) and ethanol (15 µL, 0.25 mmol), $PdBr_2(ACN)_2$ (1.8 mg, 0.005 mmol), **L10** (8.8 mg, 0.01 mmol), at 45 °C for 16 h, and was isolated by column chromatography (silica gel, 0-3% EtOAc in *n*-hexane), yielding the title product as a pale yellow oil (60 mg, 95%).

^{1}H NMR (500 MHz, $CDCl_3$) δ = 4.12 (q, *J* = 7.2 Hz, 2H), 2.40 (h, *J* = 7.0 Hz, 1H), 2.33 (t, *J* = 7.1 Hz, 2H), 1.68 – 1.61 (m, 3H), 1.47 – 1.22 (m, 16H), 1.13 (d, *J* = 7.0 Hz, 3H).

^{13}C-{^{1}H} NMR (126 MHz, $CDCl_3$) δ = 177.1, 120.0, 60.2, 39.7, 33.9, 29.6, 29.5, 29.4, 28.9, 28.8, 27.3, 25.5, 17.3, 17.3, 14.4.

HRMS (ESI) m/z calcd. for $C_{15}H_{28}NO_2$ ($[M+H]^+$): 254.2115; found: 254.2106.

96:4 er, $[\alpha]_D^{25.4}$ = +8.8 (c = 0.3, $CHCl_3$).

GC analysis (CP-Chirasil-Dex CB Column 25 m x 0.25 mm x 0.25 µm, flow: 2.34 mL/min, 130 °C): t_1 = 141.06 min (major), t_2 = 143.62 min (minor). The absolute configuration of **3qa** was assigned as (*S*)-enantiomer by analogy to (*S*)-**3aa**.

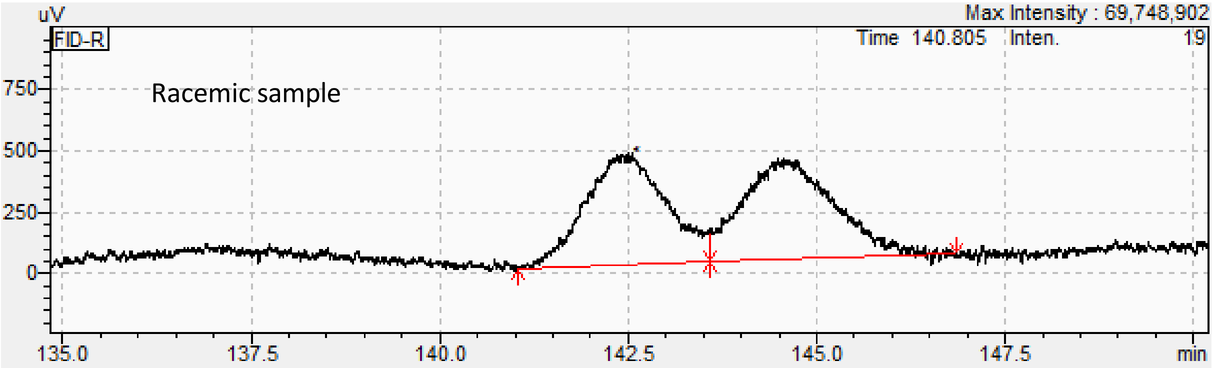

Peak#	Ret. Time	Area	Conc.	Area%
1	142.496	34097	50.026	50.026
2	144.488	34061	49.974	49.974
Total		68158	100.000	100.000

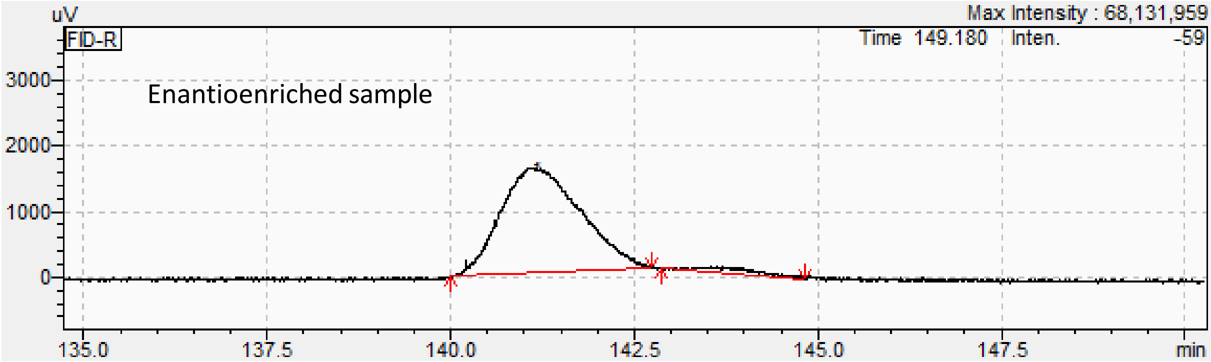

Peak#	Ret. Time	Area	Conc.	Area%
1	141.064	121328	96.032	96.032
2	143.624	5013	3.968	3.968
Total		126340	100.000	100.000

1-Ethyl 11-methyl (S)-2-methylundecanedioate (3ra):

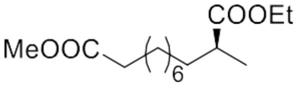

The compound was prepared according to the general procedure by reaction of methyl undec-10-enoate (99 mg, 0.50 mmol) and ethanol (15 µL, 0.25 mmol), $PdBr_2(ACN)_2$ (1.8 mg, 0.005 mmol), **L10** (8.8 mg, 0.01 mmol), at 45 °C for 16 h, and was isolated by column chromatography (silica gel, 0-10% EtOAc in *n*-hexane), yielding the title product as a colourless oil (64 mg, 94%).

^{1}H NMR (500 MHz, $CDCl_3$) δ = 4.12 (q, *J* = 7.1 Hz, 2H), 3.66 (s, 3H), 2.40 (h, *J* = 7.0 Hz, 1H), 2.29 (t, *J* = 7.6 Hz, 2H), 1.68 – 1.56 (m, 3H), 1.43 – 1.22 (m, 14H), 1.13 (d, *J* = 7.0 Hz, 3H).

^{13}C-{^{1}H} NMR (126 MHz, $CDCl_3$) δ = 177.1, 174.5, 60.2, 51.6, 39.7, 34.3, 33.9, 29.6, 29.4, 29.3, 29.3, 27.3, 25.1, 17.2, 14.4.

HRMS (ESI) m/z calcd. for $C_{15}H_{29}O_4$ ($[M+H]^+$): 273.2060; found: 273.2057.

96:4 er, $[\alpha]_D^{24.9}$ = +10.0 (c = 0.3, $CHCl_3$).

GC analysis (CP-Chirasil-Dex CB Column 25 m x 0.25 mm x 0.25 µm, flow: 2.37 mL/min, 120 °C): t_1 = 151.12 min (major), t_2 = 154.57 min (minor). The absolute configuration of **3ra** was assigned as (*S*)-enantiomer by analogy to (*S*)-**3aa**.

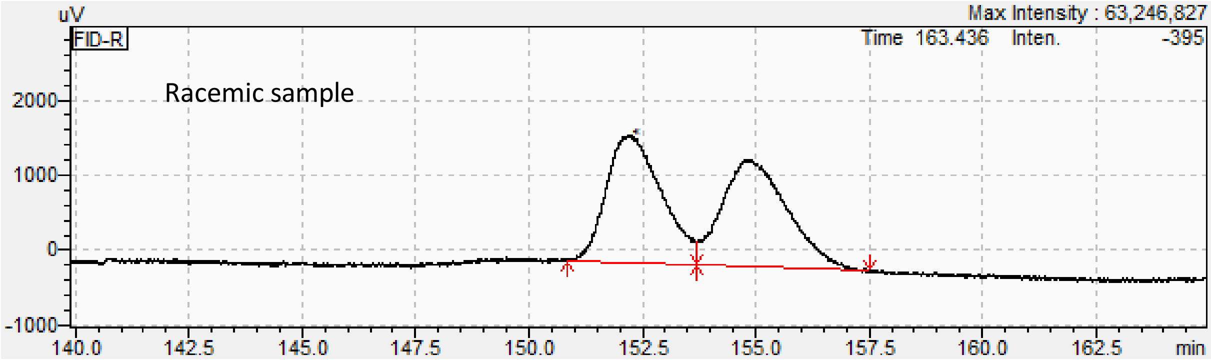

Peak#	Ret. Time	Area	Conc.	Area%
1	152.177	142451	49.995	49.995
2	154.795	142477	50.005	50.005
Total		284928	100.000	100.000

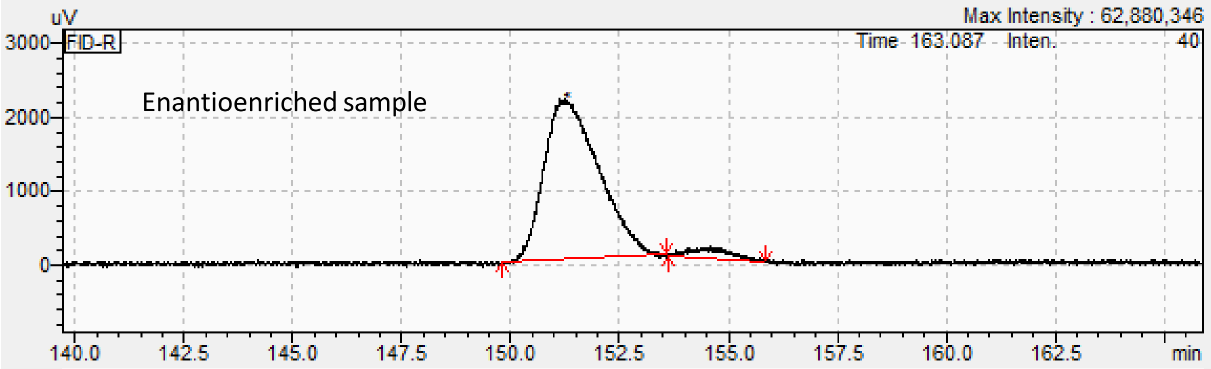

Peak#	Ret. Time	Area	Conc.	Area%
1	151.124	188136	95.607	95.607
2	154.565	8645	4.393	4.393
Total		196781	100.000	100.000

1-Ethyl 11-(furan-2-ylmethyl) (S)-2-methylundecanedioate (3sa):

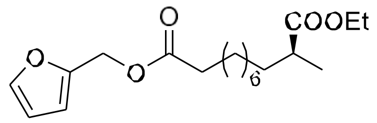

The compound was prepared according to the general procedure by reaction of furan-2-ylmethyl undec-10-enoate (132 mg, 0.50 mmol) and ethanol (15 µL, 0.25 mmol), $PdBr_2(ACN)_2$ (1.8 mg, 0.005 mmol), **L10** (8.8 mg, 0.01 mmol), at 45 °C for 16 h, and was isolated by column chromatography (silica gel, 0-10% EtOAc in *n*-hexane), yielding the title product as a colourless oil (80 mg, 95%).

^{1}H NMR (500 MHz, $CDCl_3$) δ = 7.40 (dd, *J* = 1.8, 0.9 Hz, 1H), 6.42 – 6.36 (m, 1H), 6.34 (dd, *J* = 3.3, 1.8 Hz, 1H), 5.04 (s, 2H), 4.11 (q, *J* = 7.2 Hz, 2H), 2.45 – 2.34 (m, 1H), 2.30 (t, *J* = 7.6 Hz, 2H), 1.67 – 1.54 (m, 3H), 1.41 – 1.33 (m, 1H), 1.31 – 1.22 (m, 12H), 1.12 (d, *J* = 7.0 Hz, 3H).

^{13}C-{^{1}H} NMR (126 MHz, $CDCl_3$) δ = 177.0, 173.5, 149.8, 143.3, 110.6, 110.6, 60.2, 58.0, 57.9, 39.7, 34.2, 33.9, 29.5, 29.4, 29.2, 29.1, 27.3, 24.9, 17.2, 14.4.

HRMS (ESI) m/z calcd. for $C_{19}H_{30}NaO_5$ ($[M+Na]^+$): 361.1985; found: 361.1987.

94:6 er, $[\alpha]_D^{24.9}$ = +9.4 (c = 0.3, $CHCl_3$).

SFC analysis (CHIRALPAK ID-3, 3 mmø x 100 mmL, CO_2:THF 97.5:2.5, 1.2 mL min^{-1}, 40 °C): t_1 = 6.21 min (minor), 6.74 min (major). The absolute configuration of **3sa** was assigned as (*S*)-enantiomer by analogy to (*S*)-**3aa**.

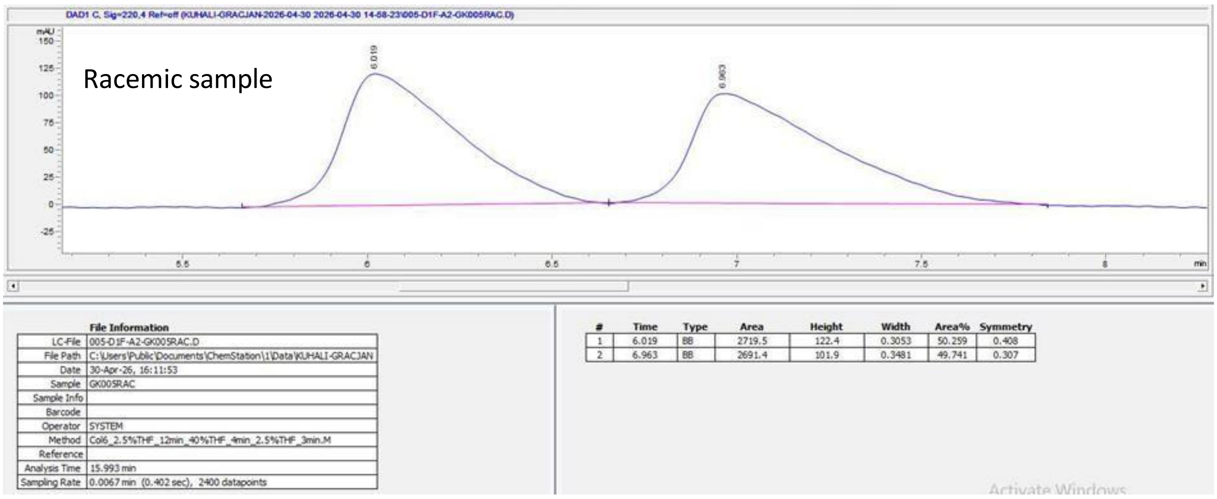

#	Time	Type	Area	Height	Width	Area%	Symmetry
1	6.019	BB	2719.5	122.4	0.3053	50.259	0.408
2	6.963	BB	2691.4	101.9	0.3481	49.741	0.307

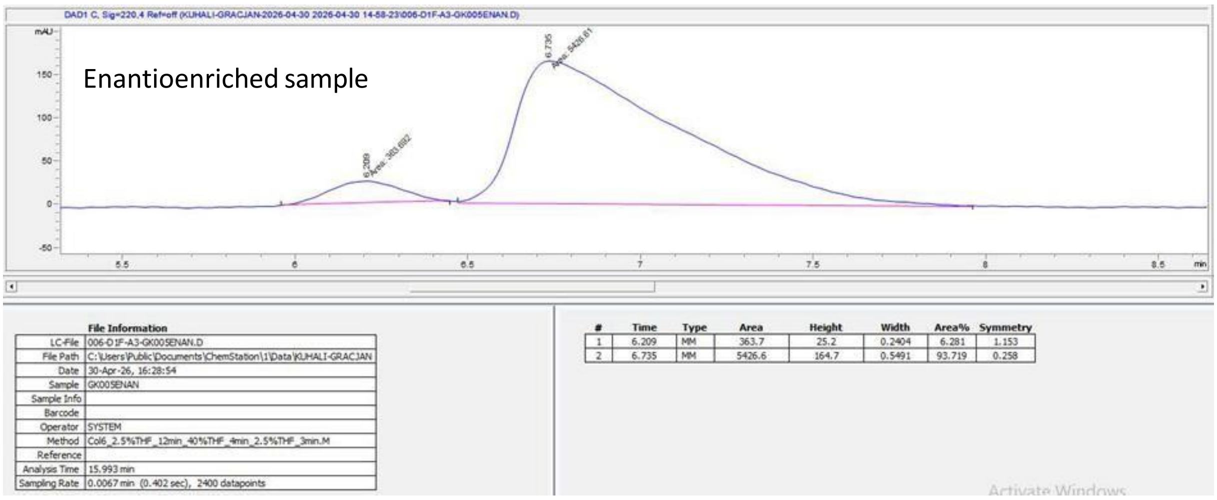

#	Time	Type	Area	Height	Width	Area%	Symmetry
1	6.209	MM	363.7	25.2	0.2404	6.281	1.153
2	6.735	MM	5426.6	164.7	0.5491	93.719	0.258

Ethyl (S)-2-methyl-11-oxo-11-(phenylthio)undecanoate (3ta):

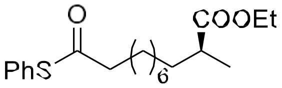

The compound was prepared according to the general procedure by reaction of *S*-phenyl undec-10-enethioate (138 mg, 0.50 mmol) and ethanol (15 µL, 0.25 mmol), $PdBr_2(ACN)_2$ (1.8 mg, 0.005 mmol), **L10** (8.8 mg, 0.01 mmol), at 45 °C for 16 h, and was isolated by column chromatography (silica gel, 0-5% EtOAc in *n*-hexane), yielding the title product as a yellow oil (80 mg, 95%).

^{1}H NMR (500 MHz, $CDCl_3$) δ = 7.40 (m, 5H), 4.13 (q, *J* = 7.1 Hz, 2H), 2.65 (t, *J* = 7.5 Hz, 2H), 2.40 (h, *J* = 7.2 Hz, 1H), 1.74 – 1.61 (m, 3H), 1.42 – 1.22 (m, 14H), 1.13 (d, *J* = 7.0 Hz, 3H).

^{13}C-{^{1}H} NMR (126 MHz, $CDCl_3$) δ = 197.7, 177.1, 134.6, 129.4, 129.3, 128.1, 60.2, 43.9, 39.7, 33.9, 29.6, 29.4, 29.3, 29.1, 27.3, 25.7, 17.3, 14.4.

HRMS (ESI) m/z calcd. for $C_{20}H_{31}O_3S$ ($[M+H]^+$): 351.1988; found: 351.1996.

94:6 er, $[\alpha]_D^{25.5}$ = +10.6 (c = 0.4, $CHCl_3$).

SFC analysis (CHIRALPAK ID-3, 3 mmø x 100 mmL, CO_2:THF 95:5, 1.2 mL min^{-1}, 40 °C): t_1 = 3.63 min (minor), 3.89 min (major). The absolute configuration of **3ta** was assigned as (*S*)-enantiomer by analogy to (*S*)-**3aa**.

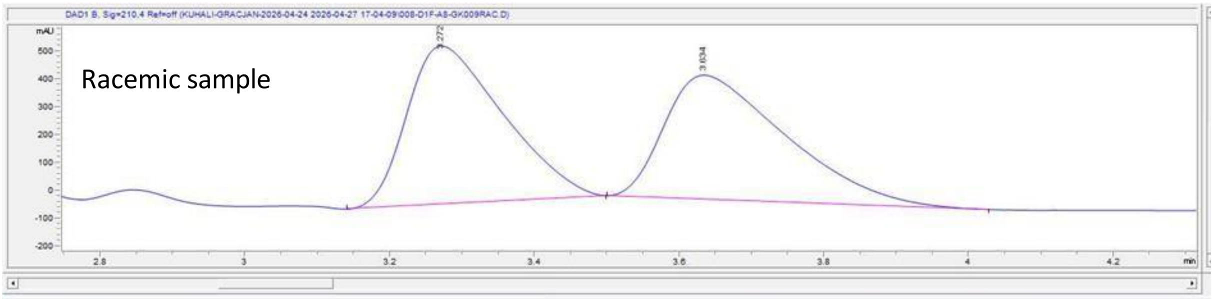

File Information	
LC-File	008-D1F-A8-GK009RAC.D
File Path	C:\Users\Public\Documents\ChemStation\1\Data\KUHALI-GRACJAN
Date	27-Apr-26, 19:05:39
Sample	GK009RAC
Sample Info	
Barcode	
Operator	SYSTEM
Method	Col6_5%THF_12min_40%THF_4min_5%THF_3min.M
Reference	
Analysis Time	15.993 min
Sampling Rate	0.0067 min (0.402 sec), 2400 datapoints

#	Time	Type	Area	Height	Width	Area%	Symmetry
1	3.272	BB	5240.8	568.4	0.1396	50.227	0.538
2	3.634	BB	5193.5	445.4	0.176	49.773	0.454

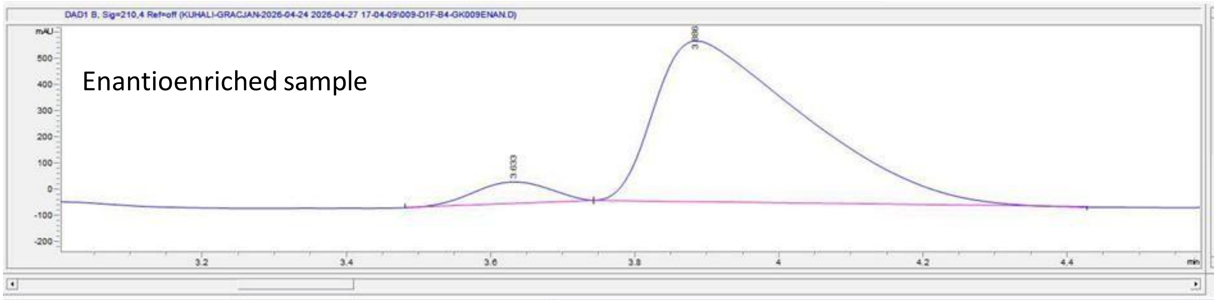

File Information	
LC-File	009-D1F-B4-GK009ENAN.D
File Path	C:\Users\Public\Documents\ChemStation\1\Data\KUHALI-GRACJAN
Date	27-Apr-26, 19:22:40
Sample	GK009ENAN
Sample Info	
Barcode	
Operator	SYSTEM
Method	Col6_5%THF_12min_40%THF_4min_5%THF_3min.M
Reference	
Analysis Time	15.993 min
Sampling Rate	0.0067 min (0.402 sec), 2400 datapoints

#	Time	Type	Area	Height	Width	Area%	Symmetry
1	3.633	BB	602.2	82.1	0.1176	6.330	1.151
2	3.886	BB	8910.6	614.4	0.2172	93.670	0.355

Ethyl (S)-11-(methoxy(methyl)amino)-2-methyl-11-oxoundecanoate (3ua):

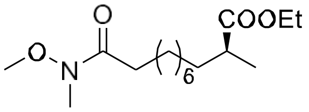

The compound was prepared according to the general procedure by reaction of *N*-methoxy-*N*-methylundec-10-enamide (114 mg, 0.50 mmol) and ethanol (15 μL, 0.25 mmol), $PdBr_2(ACN)_2$ (1.8 mg, 0.005 mmol), **L10** (8.8 mg, 0.01 mmol), at 45 °C for 16 h, and was isolated by column chromatography (silica gel, 0-10% EtOAc in *n*-hexane), yielding the title product as a pale yellow oil (68 mg, 90%).

^{1}H NMR (500 MHz, $CDCl_3$) δ = 4.12 (q, *J* = 7.1 Hz, 2H), 3.68 (s, 3H), 3.17 (s, 3H), 2.43 – 2.36 (m, 3H), 1.66 – 1.59 (m, 3H), 1.42 – 1.23 (m, 14H), 1.13 (d, *J* = 7.0 Hz, 3H).

^{13}C-{^{1}H} NMR (126 MHz, $CDCl_3$) δ = 177.1, 174.9 (bs), 61.3, 60.2, 39.7, 33.9, 32.3 (bs), 32.0 (bs), 29.6, 29.6, 29.5, 29.5, 27.3, 24.8, 17.2, 14.4.

HRMS (ESI) m/z calcd. for $C_{16}H_{32}NO_4$ ($[M+H]^+$): 302.2326; found: 302.2331.

90:10 er, $[\alpha]_D^{23.9}$ = +6.8 (c = 0.3, $CHCl_3$).

SFC analysis (CHIRALPAK IG-3, 3 mmø x 100 mmL, CO_2:MTBE 80:20 to 60:40, 20 min, 1.2 mL min^{-1}, 40 °C): t_1 = 11.02 min (minor), t_2 = 11.21 min (major). The absolute configuration of **3ua** was assigned as (*S*)-enantiomer by analogy to (*S*)-**3aa**.

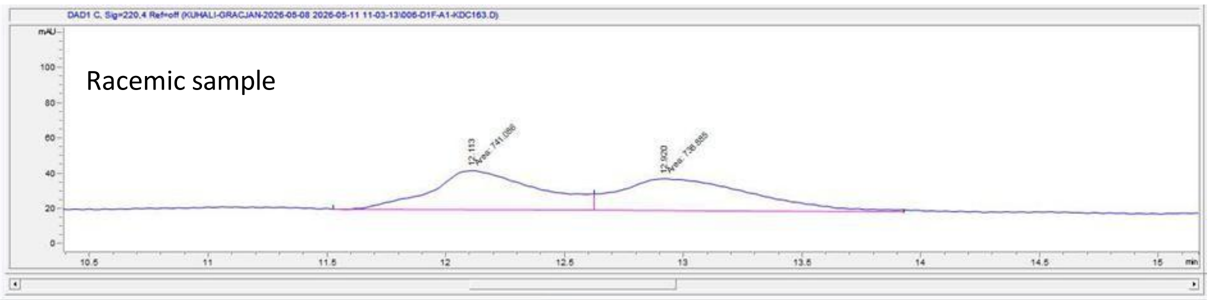

File Information

LC-File	006-D1F-A1-KDC163.D
File Path	C:\Users\Public\Documents\ChemStation\1\Data\KUHALI-GRACJAN
Date	11-May-26, 13:48:31
Sample	KDC163
Sample Info	
Barcode	
Operator	SYSTEM
Method	Col7_20-40%MTBE_30min_40%MTBE_4min_25%MTBE_3min.M
Reference	
Analysis Time	26.993 min
Sampling Rate	0.0067 min (0.402 sec), 4050 datapoints

#	Time	Type	Area	Height	Width	Area%	Symmetry
1	12.113	MF	741.1	22.8	0.5418	50.074	0
2	12.92	FM	738.9	18.6	0.6623	49.926	0.495

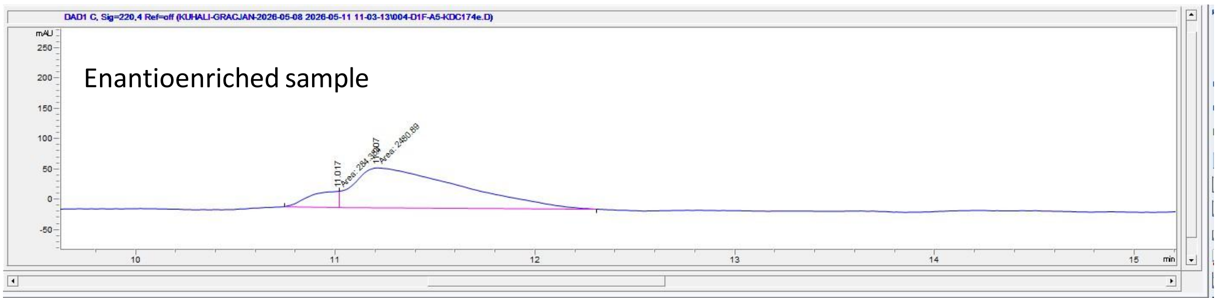

File Information

LC-File	004-D1F-A5-KDC174e.D
File Path	C:\Users\Public\Documents\ChemStation\1\Data\KUHALI-GRACJAN
Date	11-May-26, 12:40:59
Sample	KDC174e
Sample Info	
Barcode	
Operator	SYSTEM
Method	Col7_20-40%MTBE_20min_40%MTBE_4min_25%MTBE_3min.M
Reference	
Analysis Time	26.993 min
Sampling Rate	0.0067 min (0.402 sec), 4050 datapoints

#	Time	Type	Area	Height	Width	Area%	Symmetry
1	11.017	MF	284.4	26.8	0.1769	10.284	0
2	11.207	FM	2480.9	65.6	0.6307	89.716	0.263

(S)-11-ethoxy-10-methyl-11-oxoundecanoic acid (3va):

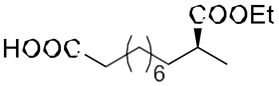

The compound was prepared according to the general procedure by reaction of undec-10-enoic acid (92 mg, 0.50 mmol) and ethanol (15 µL, 0.25 mmol), $PdBr_2(ACN)_2$ (1.8 mg, 0.005 mmol), **L10** (8.8 mg, 0.01 mmol), at 45 °C for 16 h, and was isolated by column chromatography (silica gel, 0-20% EtOAc in *n*-hexane), yielding the title product as a yellow oil (57 mg, 88%).

^{1}H NMR (500 MHz, $CDCl_3$) δ = 9.72 (bs, 1H), 4.12 (q, *J* = 7.2 Hz, 2H), 2.41 (h, *J* = 6.9 Hz, 1H), 2.34 (t, *J* = 7.5 Hz, 2H), 1.67 – 1.59 (m, 3H), 1.43 – 1.23 (m, 14H), 1.13 (d, *J* = 7.0 Hz, 3H).

^{13}C-{^{1}H} NMR (126 MHz, $CDCl_3$) δ = 179.1, 177.1, 60.2, 39.7, 34.0, 33.9, 29.6, 29.4, 29.3, 29.2, 27.3, 24.8, 17.2, 14.4.

HRMS (ESI) m/z calcd. for $C_{14}H_{27}O_4$ ($[M+H]^+$): 259.1904; found: 259.1903.

92:8 er, $[\alpha]_D^{25.3}$ = +9.8 (c = 0.3, $CHCl_3$).

SFC analysis (CHIRALPAK ID-3, 3 mmø x 100 mmL, CO_2:CH_3CN:HCOOH 92:7.2:0.8, 1.2 mL min^{-1}, 40 °C): t_1 = 5.79 min (minor), 6.63 min (major). The absolute configuration of **3va** was assigned as (*S*)-enantiomer by analogy to (*S*)-**3aa**.

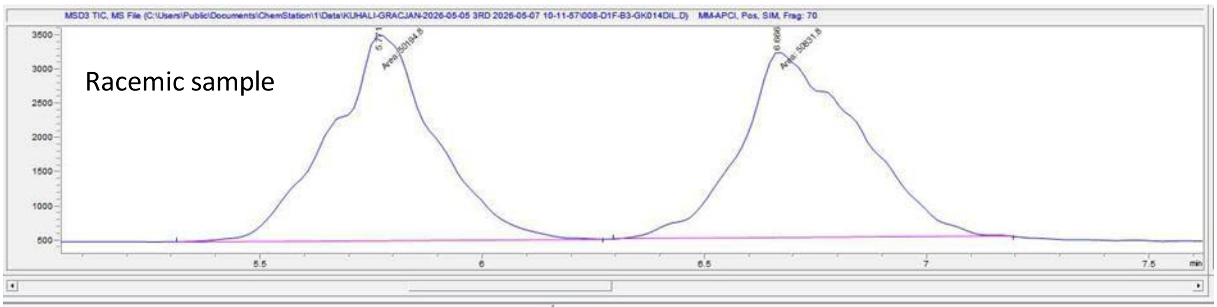

File Information	
LC-File	008-D1F-B3-GK014DIL.D
File Path	C:\Users\Public\Documents\ChemStation\1\Data\KUHALI-GRACJAN
Date	7 May 26 12:08 pm -0000
Sample	GK014DIL
Sample Info	
Barcode	
Operator	SYSTEM
Method	Col6_8%ACN_10min_40%ACN_4min_8%ACN_3min.M
Reference	
Analysis Time	14.903 min
Datapoints	1148

#	Time	Type	Area	Height	Width	Area%	Symmetry
1	5.771	MM	50194.8	3024.1	0.2766	49.685	0.923
2	6.666	MM	50831.8	2719.8	0.3115	50.315	0.506

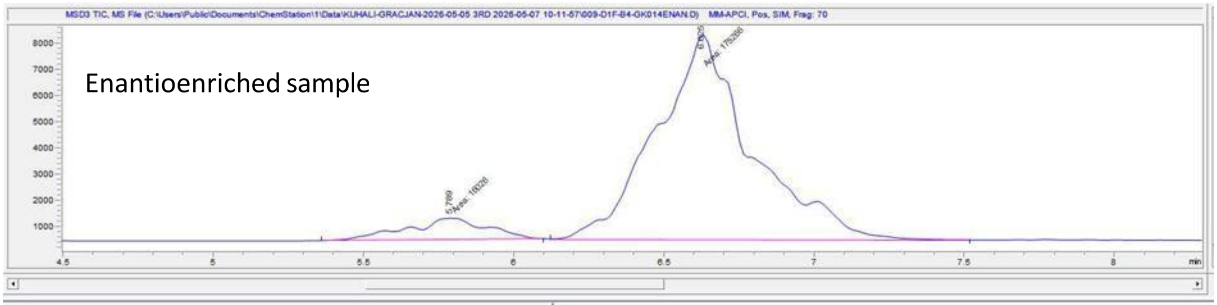

File Information	
LC-File	009-D1F-B4-GK014ENAN.D
File Path	C:\Users\Public\Documents\ChemStation\1\Data\KUHALI-GRACJAN
Date	7 May 26 12:24 pm -0000
Sample	GK014ENAN
Sample Info	
Barcode	
Operator	SYSTEM
Method	Col6_8%ACN_10min_40%ACN_4min_8%ACN_3min.M
Reference	
Analysis Time	14.916 min
Datapoints	1149

#	Time	Type	Area	Height	Width	Area%	Symmetry
1	5.789	MM	16026	838.8	0.3184	8.378	1.22
2	6.625	MM	175266.5	7895.9	0.37	91.622	0.892

Ethyl (S)-2-methyl-11-oxo-11-phenylundecanoate (3wa):

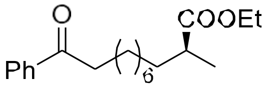

The compound was prepared according to the general procedure by reaction of 1-phenylundec-10-en-1-one (122 mg, 0.50 mmol) and ethanol (15 µL, 0.25 mmol), $PdBr_2(ACN)_2$ (1.8 mg, 0.005 mmol), **L10** (8.8 mg, 0.01 mmol), at 45 °C for 16 h, and was isolated by column chromatography (silica gel, 0-10% EtOAc in *n*-hexane), yielding the title product as a pale yellow oil (76 mg, 96%).

^{1}H NMR (500 MHz, $CDCl_3$) δ = 7.97 – 7.94 (m, 2H), 7.57 – 7.53 (m, 1H), 7.48 – 7.44 (m, 2H), 4.12 (qd, *J* = 7.1, 0.7 Hz, 2H), 2.96 (t, *J* = 7.3 Hz, 2H), 2.40 (h, *J* = 7.0 Hz, 1H), 1.73 (p, *J* = 7.4 Hz, 2H), 1.67 – 1.59 (m, 1H), 1.43 – 1.22 (m, 14H), 1.13 (d, *J* = 7.0 Hz, 3H).

^{13}C-{^{1}H} NMR (126 MHz, $CDCl_3$) δ = 200.7, 177.1, 137.2, 133.0, 128.7, 128.2, 60.2, 39.7, 38.8, 33.9, 29.6, 29.6, 29.5, 29.5, 27.3, 24.5, 17.2, 14.4.

HRMS (ESI) m/z calcd. for $C_{20}H_{31}O_3$ ($[M+H]^+$): 319.2268; found: 319.2262.

95:5 er, $[\alpha]_D^{24.6}$ = +3.9 (c = 0.3, $CHCl_3$).

SFC analysis (CHIRALPAK IG-3, 3 mmø x 100 mmL, CO_2:THF 92.5:7.5, 1.2 mL min^{-1}, 40 °C): t_1 = 4.19 min (major), 4.92 min (minor). The absolute configuration of **3wa** was assigned as (*S*)-enantiomer by analogy to (*S*)-**3aa**.

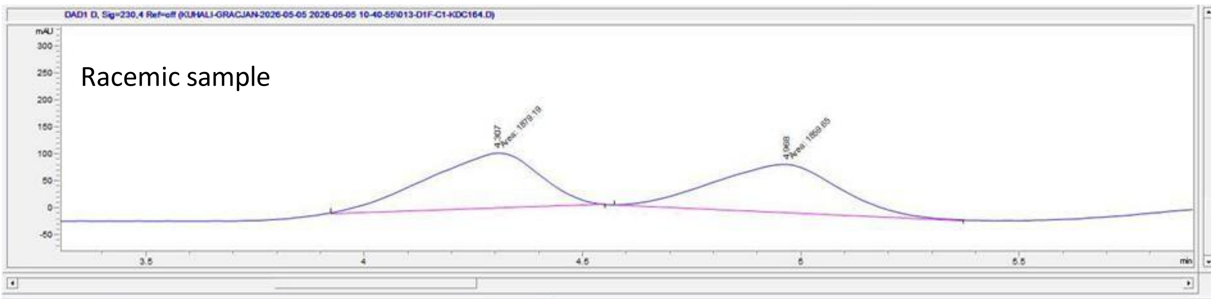

File Information	
LC-File	013-D1F-C1-KDC164.D
File Path	C:\Users\Public\Documents\ChemStation\1\Data\KUHALI-GRACJAN
Date	05-May-26, 14:03:11
Sample	KDC164
Sample Info	
Barcode	
Operator	SYSTEM
Method	Col7_7.5%THF_8min_40%THF_4min_7.5%THF_3min.M
Reference	
Analysis Time	14.993 min
Sampling Rate	0.0067 min (0.402 sec), 2250 datapoints

#	Time	Type	Area	Height	Width	Area%	Symmetry
1	4.307	MM	1879.2	103.6	0.3024	50.261	1.701
2	4.968	MM	1859.7	91.4	0.3392	49.739	1.185

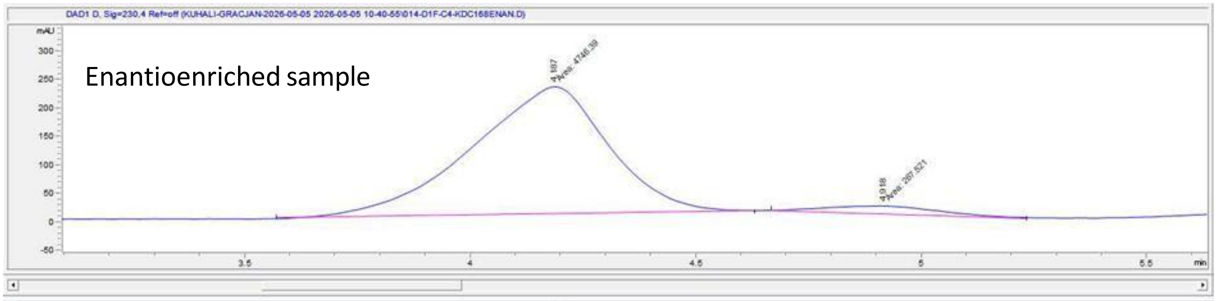

File Information	
LC-File	014-D1F-C4-KDC168ENAN.D
File Path	C:\Users\Public\Documents\ChemStation\1\Data\KUHALI-GRACJAN
Date	05-May-26, 14:19:38
Sample	KDC168ENAN
Sample Info	
Barcode	
Operator	SYSTEM
Method	Col7_7.5%THF_8min_40%THF_4min_7.5%THF_3min.M
Reference	
Analysis Time	14.993 min
Sampling Rate	0.0067 min (0.402 sec), 2250 datapoints

#	Time	Type	Area	Height	Width	Area%	Symmetry
1	4.187	MM	4746.4	224.3	0.3527	94.664	1.482
2	4.918	MM	267.5	14.9	0.2992	5.336	0.846

Ethyl (S)-2-methyl-11-oxoundecanoate (3xa):

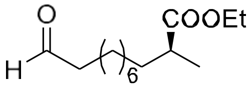

The compound was prepared according to the general procedure by reaction of undec-10-enal (84 mg, 0.50 mmol) and ethanol (15 µL, 0.25 mmol), $PdBr_2(ACN)_2$ (1.8 mg, 0.005 mmol), **L10** (8.8 mg, 0.01 mmol), at 45 °C for 16 h, and was isolated by column chromatography (silica gel, 0-10% EtOAc in *n*-hexane), yielding the title product as a colourless oil (41 mg, 68%).

^{1}H NMR (500 MHz, $CDCl_3$) δ = 9.76 (t, *J* = 1.9 Hz, 1H), 4.12 (q, *J* = 7.1 Hz, 2H), 2.44 – 2.37 (m, 3H), 1.67 – 1.59 (m, 3H), 1.42 – 1.23 (m, 14H), 1.13 (d, *J* = 7.0 Hz, 3H).

^{13}C-{^{1}H} NMR (126 MHz, $CDCl_3$) δ = 203.1, 177.1, 60.2, 44.1, 39.7, 33.9, 29.6, 29.4, 29.4, 29.3, 27.3, 22.2, 17.3, 14.4.

94:6 er, $[\alpha]_D^{24.3}$ = +10.2 (c = 0.2, $CHCl_3$).

HRMS (ESI) m/z calcd. for $C_{14}H_{27}O_3$ ($[M+H]^+$): 243.1955; found: 243.1952.

GC analysis (CP-Chirasil-Dex CB Column 25 m x 0.25 mm x 0.25 µm, flow: 2.37 mL/min, 120 °C): t_1 = 88.97 min (major), t_2 = 90.69 min (minor). The absolute configuration of **3xa** was assigned as (*S*)-enantiomer by analogy to (*S*)-**3aa**.

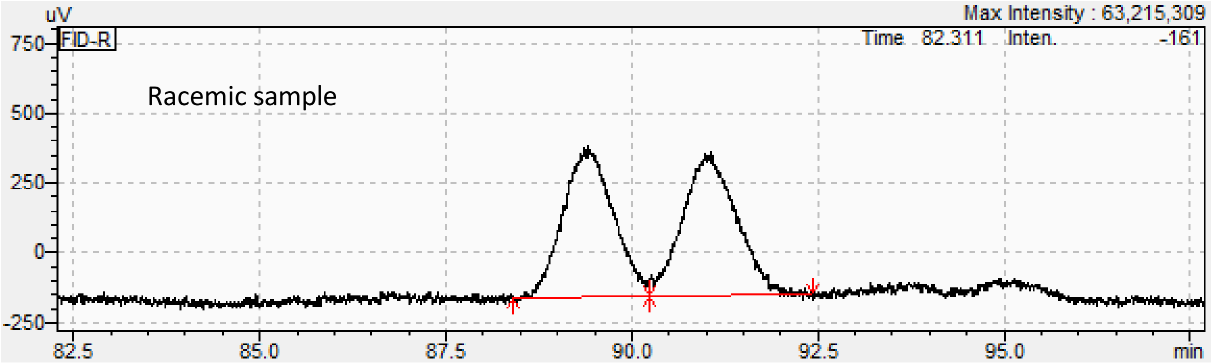

Peak#	Ret. Time	Area	Conc.	Area%
1	89.414	24834	49.671	49.671
2	91.069	25163	50.329	50.329
Total		49997	100.000	100.000

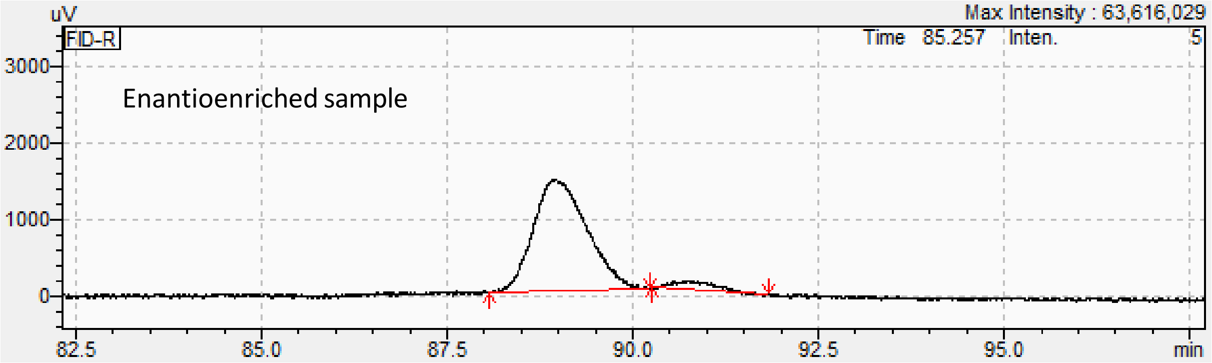

Peak#	Ret. Time	Area	Conc.	Area%
1	88.969	72397	94.308	94.308
2	90.688	4370	5.692	5.692
Total		76766	100.000	100.000

Ethyl (S)-10-(1,3-dioxolan-2-yl)-2-methyldecanoate (3ya):

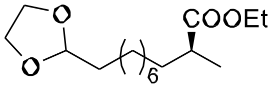

The compound was prepared according to the general procedure by reaction of 2-(dec-9-en-1-yl)-1,3-dioxolane (106 mg, 0.50 mmol) and ethanol (15 µL, 0.25 mmol), $PdBr_2(ACN)_2$ (1.8 mg, 0.005 mmol), **L10** (8.8 mg, 0.01 mmol), at 45 °C for 16 h, and was isolated by column chromatography (silica gel, 0-10% EtOAc in n-hexane), yielding the title product as a colourless oil (50 mg, 70%).

^{1}H NMR (500 MHz, $CDCl_3$) δ = 4.83 (t, *J* = 4.8 Hz, 1H), 4.11 (q, *J* = 7.1 Hz, 2H), 3.99 – 3.91 (m, 2H), 3.88 – 3.79 (m, 2H), 2.39 (h, *J* = 6.9 Hz, 1H), 1.67 – 1.58 (m, 3H), 1.43 – 1.35 (m, 3H), 1.34 – 1.23 (m, 13H), 1.12 (d, *J* = 7.0 Hz, 3H).

^{13}C-{^{1}H} NMR (126 MHz, $CDCl_3$) δ = 177.1, 104.8, 65.0, 60.2, 39.7, 34.0, 33.9, 29.6, 29.6, 29.6, 29.5, 27.3, 24.2, 17.2, 14.4.

HRMS (ESI) m/z calcd. for $C_{16}H_{31}O_4$ ($[M+H]^+$): 287.2217; found: 287.2215.

92:8 er, $[\alpha]_D^{24.0}$ = +8.0 (c = 0.3, $CHCl_3$).

SFC analysis (CHIRALPAK ID-3, 3 mmø x 100 mmL, CO_2:CH_3CN 90:10, 1.2 mL min^{-1}, 40 °C): t_1 = 4.11 min (minor), 4.94 min (major). The absolute configuration of **3ya** was assigned as (*S*)-enantiomer by analogy to (*S*)-**3aa**.

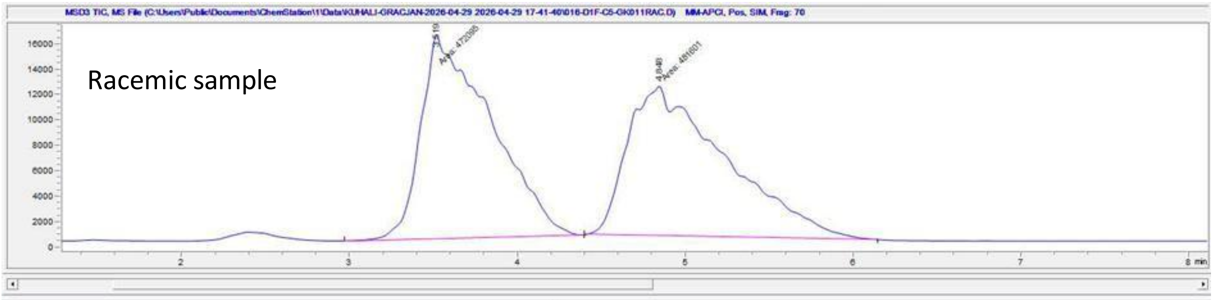

File Information

LC-File	016-D1F-C5-GK011RAC.D
File Path	C:\Users\Public\Documents\ChemStation\1\Data\KUHALI-GRACJAN
Date	29 Apr 26 9:45 pm -0000
Sample	GK011RAC
Sample Info	
Barcode	
Operator	SYSTEM
Method	Col6SIM_10%ACN_8min_40%ACN_4min_10%ACN_3min.M
Reference	
Analysis Time	14.903 min
Datapoints	1148

#	Time	Type	Area	Height	Width	Area%	Symmetry
1	3.519	MM	472094.6	16149.9	0.4872	49.502	0.301
2	4.848	MM	481600.6	11855.4	0.677	50.498	0.466

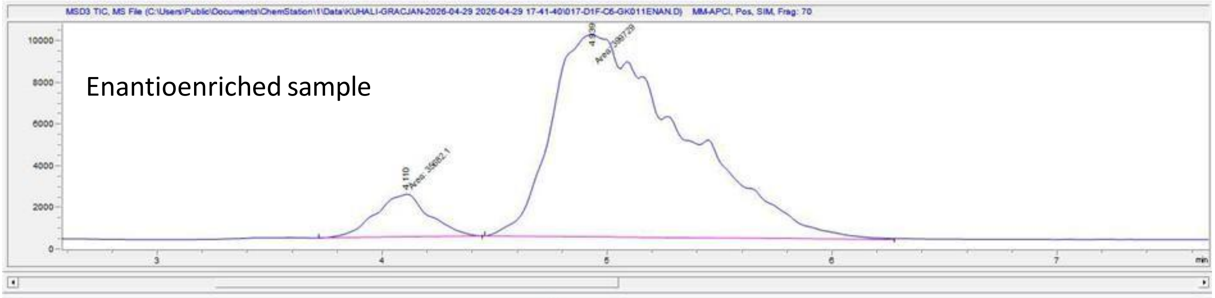

File Information

LC-File	017-D1F-C6-GK011ENAN.D
File Path	C:\Users\Public\Documents\ChemStation\1\Data\KUHALI-GRACJAN
Date	29 Apr 26 10:01 pm -0000
Sample	GK011ENAN
Sample Info	
Barcode	
Operator	SYSTEM
Method	Col6SIM_10%ACN_8min_40%ACN_4min_10%ACN_3min.M
Reference	
Analysis Time	14.916 min
Datapoints	1149

#	Time	Type	Area	Height	Width	Area%	Symmetry
1	4.11	MM	35682.1	2093.1	0.2841	8.195	1.332
2	4.939	MM	399728.6	9770.1	0.6819	91.805	0.453

Ethyl (S)-11-(1,3-dioxoisoindolin-2-yl)-2-methylundecanoate (3za):

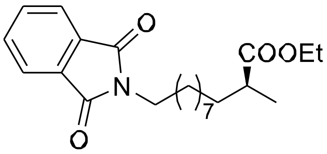

The compound was prepared according to the general procedure by reaction of 2-(undec-10-en-1-yl)isoindoline-1,3-dione (150 mg, 0.50 mmol) and ethanol (15 µL, 0.25 mmol), $PdBr_2(ACN)_2$ (1.8 mg, 0.005 mmol), **L10** (8.8 mg, 0.01 mmol), at 45 °C for 16 h, and was isolated by column chromatography (silica gel, 0-10% EtOAc in n-hexane), yielding the title product as a colourless oil (91 mg, 97%).

^{1}H NMR (500 MHz, $CDCl_3$) δ = 7.86 – 7.80 (m, 2H), 7.73 – 7.67 (m, 2H), 4.11 (q, *J* = 7.2 Hz, 2H), 3.66 (t, *J* = 7.0 Hz, 2H), 2.46 – 2.32 (m, 1H), 1.69 – 1.58 (m, 3H), 1.40 – 1.23 (m, 16H), 1.12 (d, *J* = 7.0 Hz, 3H).

^{13}C-{^{1}H} NMR (126 MHz, $CDCl_3$) δ = 177.1, 168.6, 134.0, 132.3, 123.3, 60.2, 39.7, 38.2, 33.9, 29.6, 29.5, 29.5, 29.3, 28.7, 27.3, 27.0, 17.2, 14.4.

HRMS (ESI) m/z calcd. for $C_{22}H_{32}NO_4$ ($[M+H]^+$): 374.2326; found: 374.2325.

92:8 er, $[\alpha]_D^{22.9}$ = +8.2 (c = 0.4, $CHCl_3$).

SFC analysis (CHIRALPAK ID-3, 3 mmø x 100 mmL, CO_2:MeOH, 85:15, 1.2 mL min^{-1}, 40 °C): t_1 = 5.64 min (major), t_2 = 7.14 min (minor). The absolute configuration of **3za** was assigned as (*S*)-enantiomer by analogy to (*S*)-**3aa**.

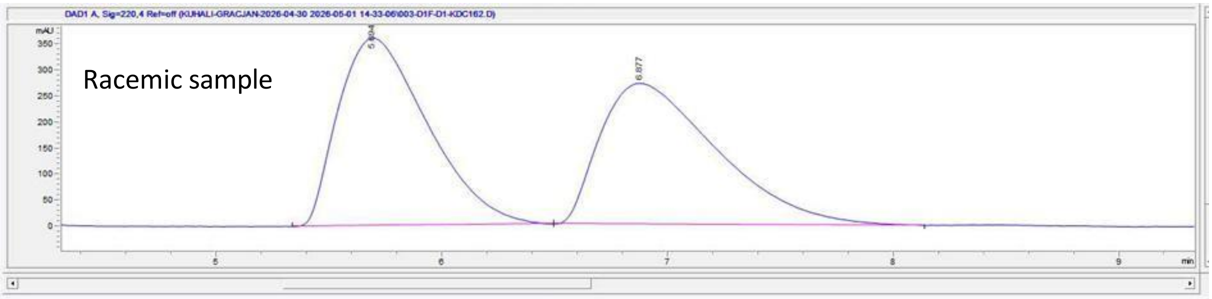

File Information	
LC-File	003-D1F-D1-KDC162.D
File Path	C:\Users\Public\Documents\ChemStation\1\Data\KUHALI-GRACJAN
Date	01-May-26, 15:13:08
Sample	KDC162
Sample Info	
Barcode	
Operator	SYSTEM
Method	Col6_15%MeOH_12min_40%MeOH_4min_2.5%MeOH_3min.M
Reference	
Analysis Time	18.993 min
Sampling Rate	0.0067 min (0.402 sec), 2850 datapoints

#	Time	Type	Area	Height	Width	Area%	Symmetry
1	5.694	BB	9851.7	358.8	0.4243	50.366	0.605
2	6.877	BB	9708.6	269.4	0.5285	49.634	0.482

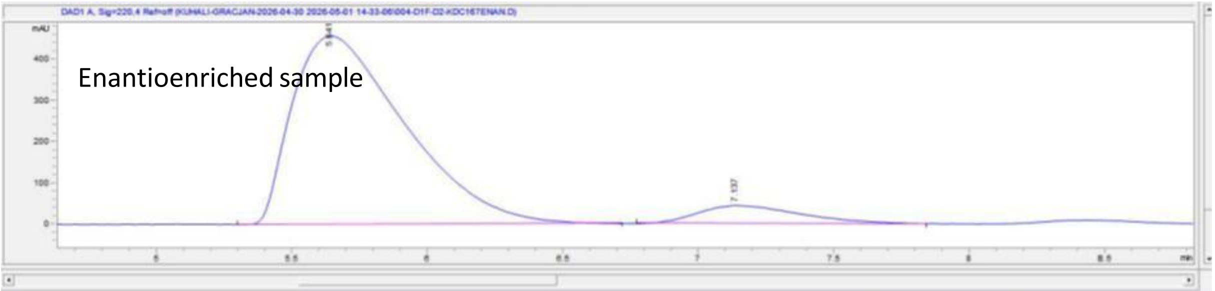

File Information	
LC-File	004-D1F-D2-KDC167ENAN.D
File Path	C:\Users\Public\Documents\ChemStation\1\Data\KUHALI-GRACJAN
Date	01-May-26, 15:33:09
Sample	KDC167ENAN
Sample Info	
Barcode	
Operator	SYSTEM
Method	Col6_15%MeOH_12min_40%MeOH_4min_2.5%MeOH_3min.M
Reference	
Analysis Time	18.993 min
Sampling Rate	0.0067 min (0.402 sec), 2850 datapoints

#	Time	Type	Area	Height	Width	Area%	Symmetry
1	5.641	BB	13197.1	456.4	0.4414	91.967	0.488
2	7.137	BB	1152.8	43.5	0.343	8.033	0.558

Ethyl (S)-2-phenylpropanoate (5aa):

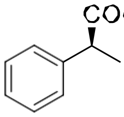

The compound was prepared according to the general procedure by reaction of styrene (58 µL, 0.5 mmol) and ethanol (15 µL, 0.25 mmol), and was isolated by column chromatography (silica gel, petroleum ether/dichloromethane = 8/2), yielding the title product as a colourless oil (34 mg, 76 %). The NMR data match those reported in literature.[4]

^{1}H NMR (500 MHz, CDCl$_3$) δ = 7.35 – 7.29 (m, 4H), 7.27 – 7.25 (m, 1H), 4.19 – 4.06 (m, 2H), 3.71 (q, *J* = 7.2 Hz, 1H), 1.50 (d, *J* = 7.2 Hz, 3H), 1.21 (t, *J* = 7.1 Hz, 3H).

^{13}C-{^{1}H} NMR (126 MHz, CDCl$_3$) δ = 174.7, 140.9, 128.7, 127.6, 127.2, 60.9, 45.7, 18.8, 14.3.

75:25 er, $[\alpha]_D^{25}$ = +1.5 (c = 1.1, CHCl$_3$).

To determine the enantiomeric ratio, the isolated ester was reduced to the alcohol according to the procedure in section 5. GC analysis (CycloSil-B Column 25m x 0.25 mm x 0.25 µm, flow: 3.16 mL/min, 120 °C): t_1 = 27.43 min (minor), t_2 = 28.65 min (major). The absolute configuration of **5aa** was assigned as (*S*)-enantiomer by analogy to (*S*)-**3aa**.

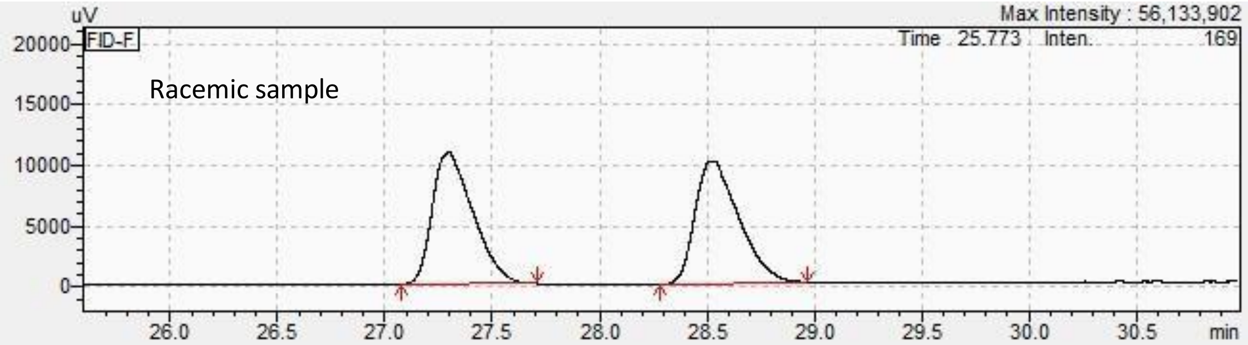

Peak#	Ret. Time	Area	Conc.	Area%
1	27.300	143793	50.124	50.124
2	28.536	143084	49.876	49.876
Total		286877	100.000	100.000

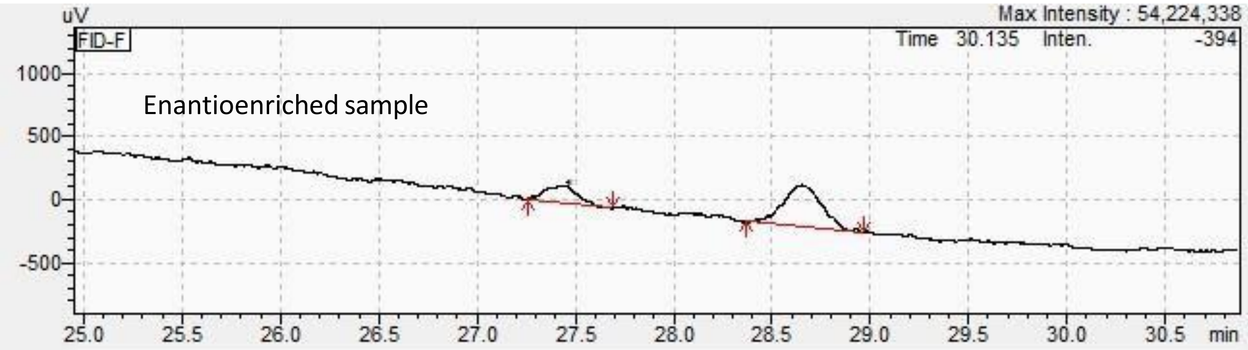

Peak#	Ret. Time	Area	Conc.	Area%
1	27.429	1437	24.481	24.481
2	28.654	4432	75.519	75.519
Total		5869	100.000	100.000

Ethyl (R)-2-(benzyloxy)propanoate (5ca):

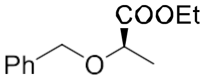

The compound was prepared according to the general procedure by reaction of ((Vinyloxy)methyl)benzene (138.32 µL, 1.0 mmol) and ethanol (15 µL, 0.25 mmol), $PdBr_2(ACN)_2$ (1.8 mg, 0.005 mmol), **L10** (5.3 mg, 0.01 mmol) and the yield was determined using ^1H NMR. Yield = 22%.

GC analysis (CP-Chirasil-Dex CB Column 25 m x 0.25 mm x 0.25 µm, flow: 1.97 mL/min, 90 °C): t_1 = 27.11 min (minor), t_2 = 28.17 min (major). The absolute configuration of **5ca** was assigned as (*R*)-enantiomer by analogy to *(S)*-**3aa**.

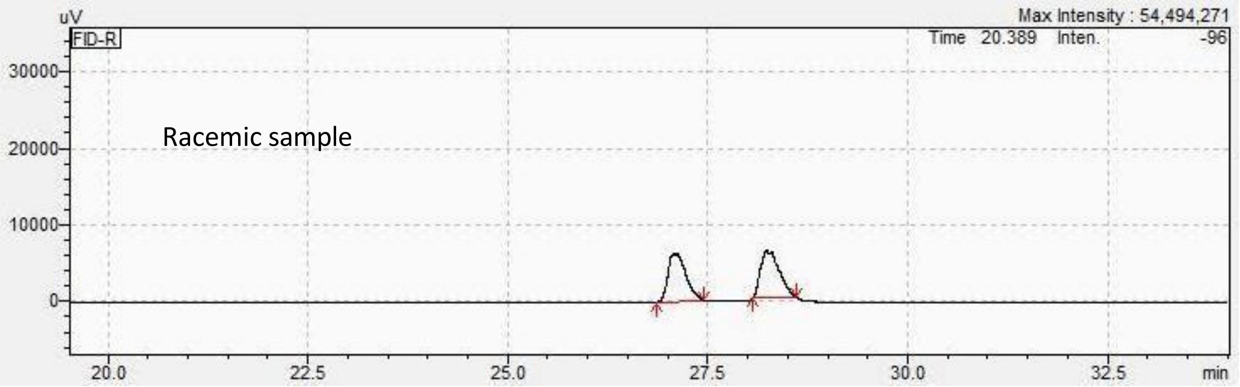

Peak#	Ret. Time	Area	Conc.	Area%
1	27.083	97623	49.216	49.216
2	28.241	100732	50.784	50.784
Total		198355	100.000	100.000

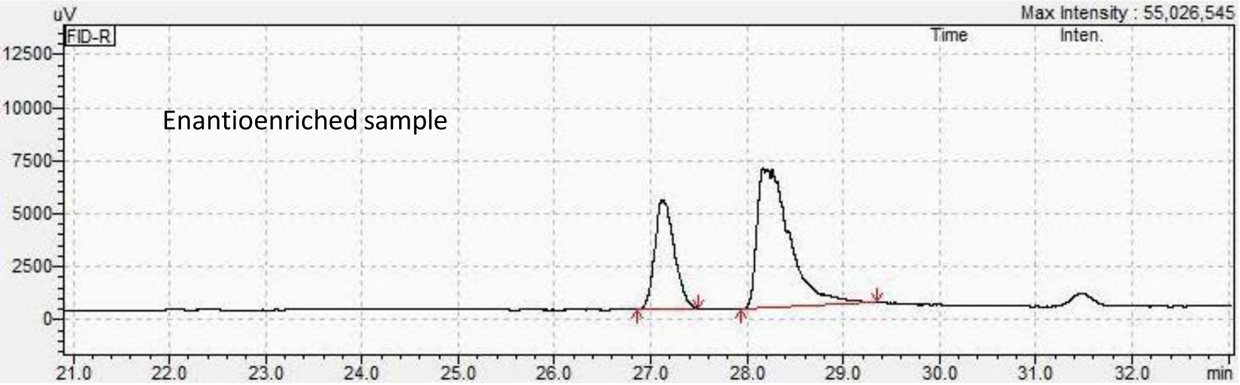

Peak#	Ret. Time	Area	Conc.	Area%
1	27.112	72337	31.798	31.798
2	28.165	155156	68.202	68.202
Total		227493	100.000	100.000

Ethyl (S)-2-methyloctanoate + ethyl 2-ethylheptanoate (α- and β-5da):

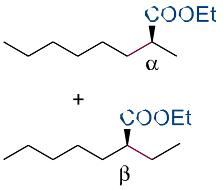

The compound was prepared according to the general procedure by reaction of cis-2-octene (79 μL, 0.50 mmol) and ethanol (15 μL, 0.25 mmol), $PdBr_2(ACN)_2$ (1.8 mg, 0.005 mmol), **L10** (8.8 mg, 0.01 mmol), at 45 °C for 48 h, and was isolated by column chromatography (silica gel, petroleum ether/dichloromethane = 9/1), yielding the title product as a colorless liquid containig a mixture **α-** and **β-5da** (31 mg, 67%, **α-5da:β-5da** = 81:19). The NMR data match those reported in the literature for the racemic product.[3]

^{1}H NMR (500 MHz, $CDCl_3$) δ = 4.19 – 4.10 (m, 2H), 2.48 – 2.37 (m, 0.81H, **α-5da**), 2.30 – 2.23 (m, 0.19H, **β-5da**), 1.69 – 1.58 (m, 1H), 1.48 – 1.37 (m, 1H), 1.32 – 1.25 (m, 11H), 1.15 (d, *J* = 7.0 Hz, 2H, **α-5da**), 0.95 – 0.85 (m, 4H).

^{13}C NMR (126 MHz, $CDCl_3$) δ 177.1 (**α-5da**), 176.6 (**β-5da**), 60.2 (**α-5da**), 60.1 (**β-5da**), 47.5 (**β-5da**), 39.7 (**α-5da**), 34.0 (**α-5da**), 32.2 (**β-5da**), 31.9 (**β-5da**), 31.8 (**c**), 29.3 (**α-5da**), 27.3 (**α-5da**), 27.2 (**β-5da**), 25.6 (**β-5da**), 22.7 (**α-5da**), 22.6 (**β-5da**), 17.2 (**α-5da**), 14.5 (**β-5da**), 14.4 (**α-5da**), 14.2 (**α-5da**), 14.1 (**β-5da**), 12.0 (**β-5da**).

GC analysis (CP-Chirasil-Dex CB Column 25 m x 0.25 mm x 0.25 μm, flow:2.11 mL/min, 75 °C): t_1 = 29.0 min (major, **β-5da**), 30.0 min (minor, **β-5da**), 39.5 min (major, **α-5da**), t_2 = 40.6 min (minor, **α-5da**).

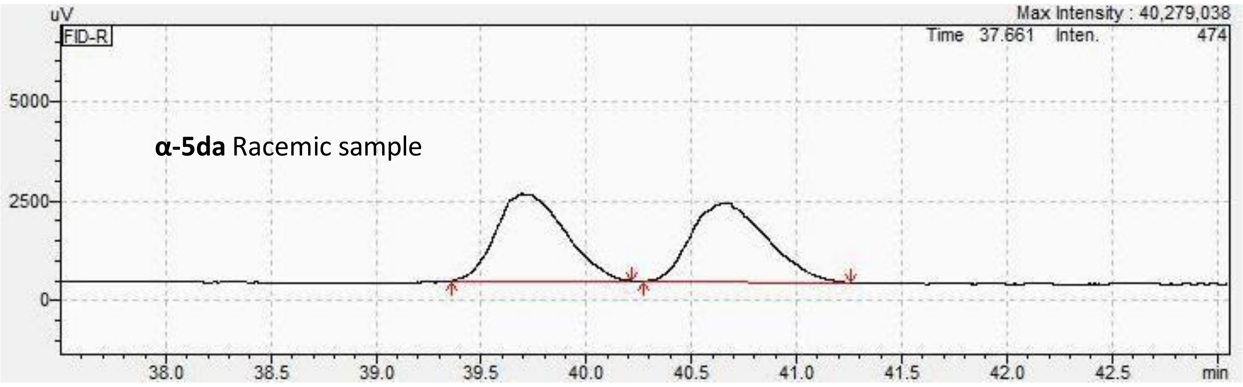

Peak#	Ret. Time	Area	Conc.	Area%
1	39.696	49613	49.971	49.971
2	40.673	49669	50.029	50.029
Total		99282	100.000	100.000

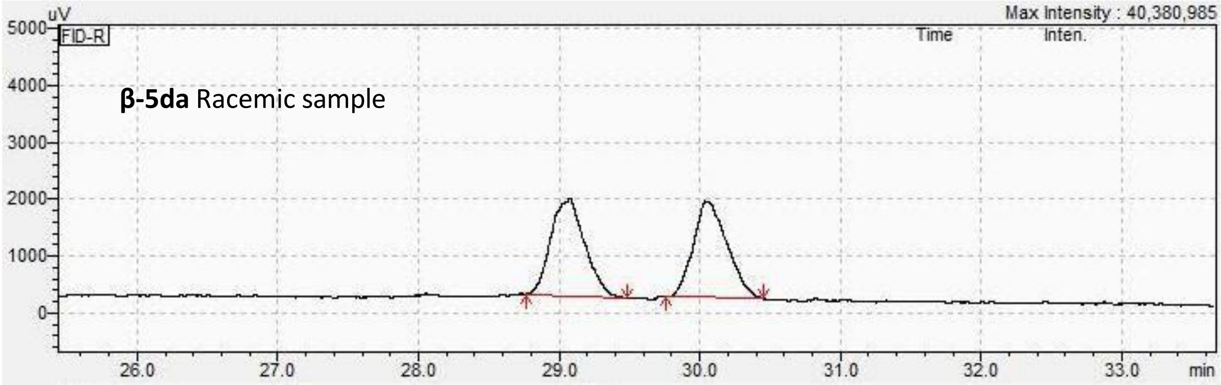

Peak#	Ret. Time	Area	Conc.	Area%
1	29.077	28365	49.967	49.967
2	30.038	28403	50.033	50.033
Total		56768	100.000	100.000

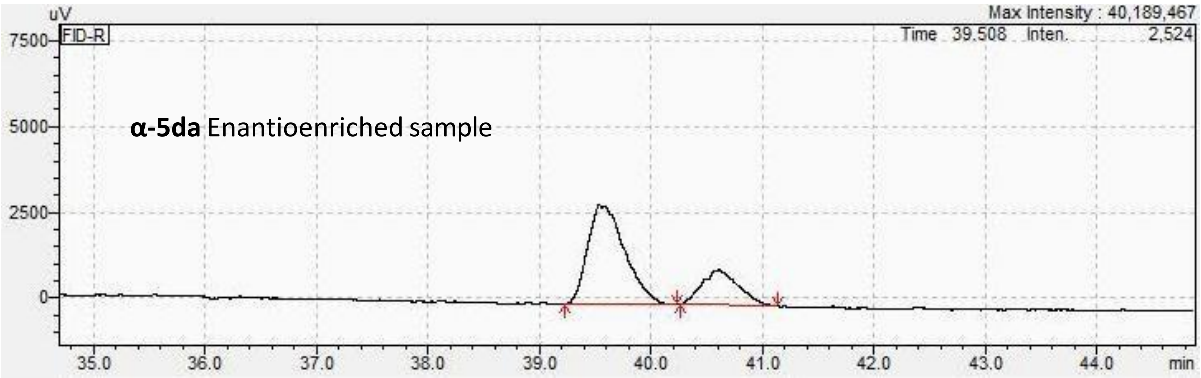

Peak#	Ret. Time	Area	Conc.	Area%
1	39.535	66525	73.827	73.827
2	40.610	23585	26.173	26.173
Total		90110	100.000	100.000

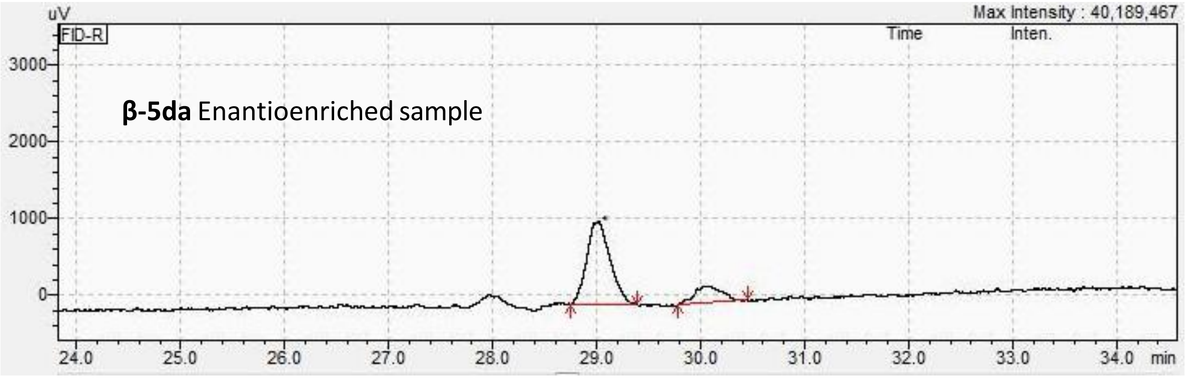

Peak#	Ret. Time	Area	Conc.	Area%
1	29.018	16752	80.699	80.699
2	30.033	4007	19.301	19.301
Total		20759	100.000	100.000

Chiral GC data for the same experiment but starting from (*Z*)-**4d** and ethanol with **L5** as ligand.

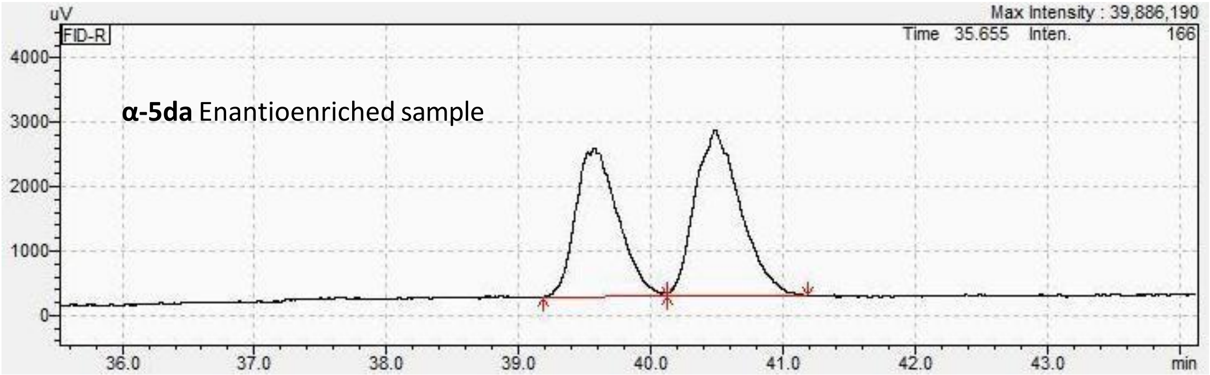

Peak#	Ret. Time	Area	Conc.	Area%
1	39.573	51139	45.189	45.189
2	40.485	62027	54.811	54.811
Total		113166	100.000	100.000

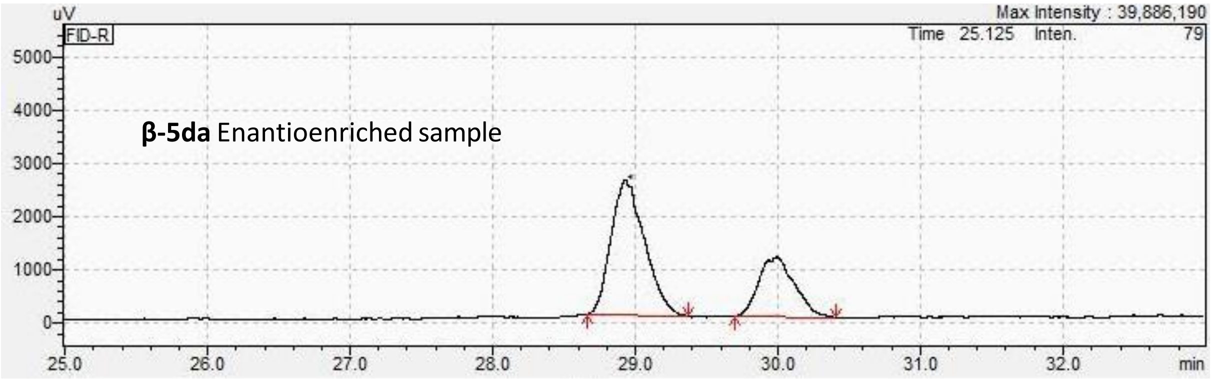

Peak#	Ret. Time	Area	Conc.	Area%
1	28.924	41598	67.793	67.793
2	29.995	19762	32.207	32.207
Total		61360	100.000	100.000

Chiral GC data for the same experiment but starting from (*Z*)-**4d** and ethanol with **L6** as ligand.

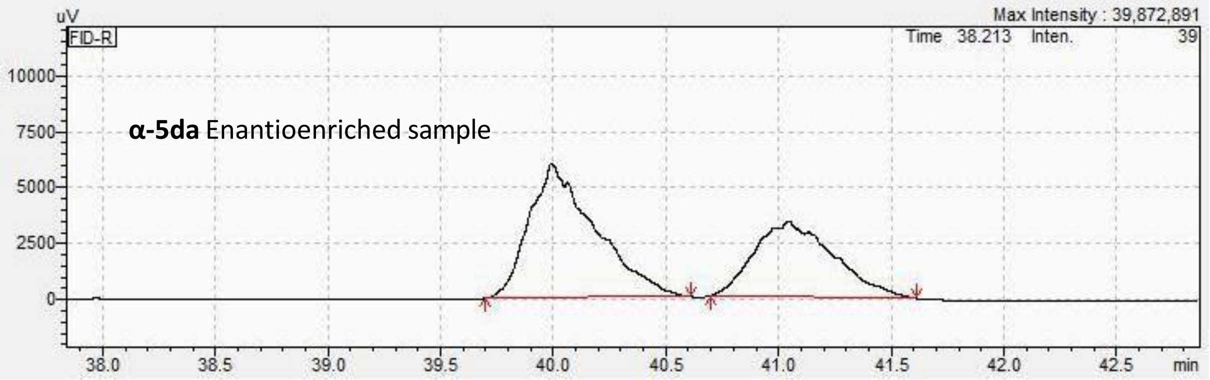

Peak#	Ret. Time	Area	Conc.	Area%
1	39.992	121796	59.865	59.865
2	41.044	81655	40.135	40.135
Total		203451	100.000	100.000

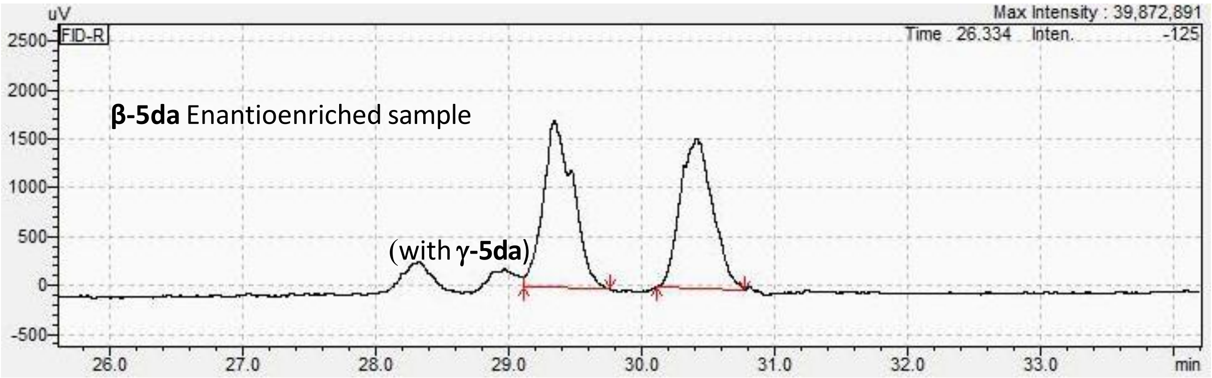

Peak#	Ret. Time	Area	Conc.	Area%
1	29.347	26796	50.663	50.663
2	30.415	26094	49.337	49.337
Total		52890	100.000	100.000

Chiral GC data for the same experiment but starting from (*E*)-**4d** and ethanol with **L10** as ligand.

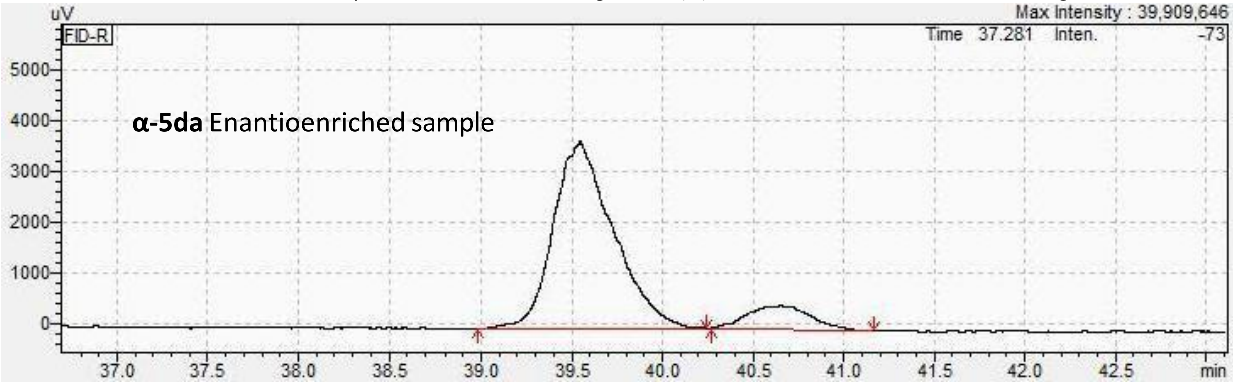

Peak#	Ret. Time	Area	Conc.	Area%
1	39.548	84379	88.025	88.025
2	40.652	11479	11.975	11.975
Total		95858	100.000	100.000

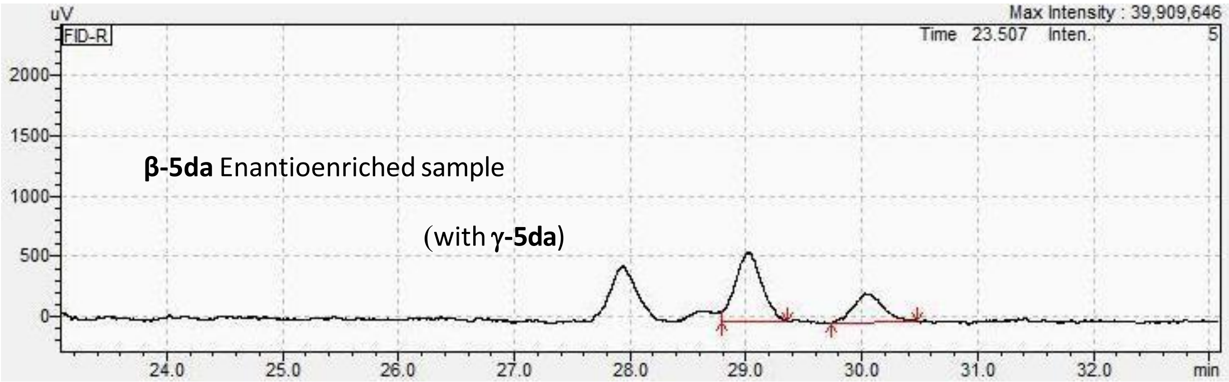

Peak#	Ret. Time	Area	Conc.	Area%
1	29.028	9208	67.965	67.965
2	30.026	4340	32.035	32.035
Total		13548	100.000	100.000

Chiral GC data for the same experiment but starting from (*E*)-**4d** and ethanol with **L5** as ligand.

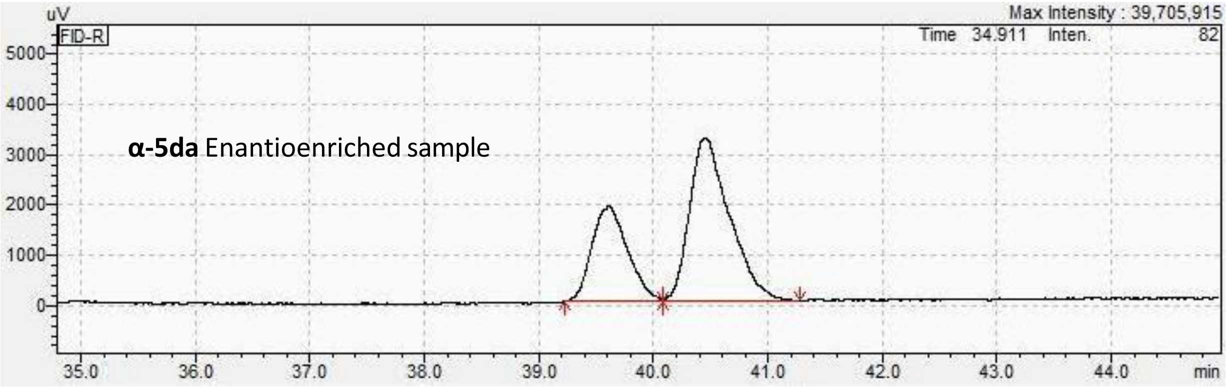

Peak#	Ret. Time	Area	Conc.	Area%
1	39.606	40978	34.190	34.190
2	40.445	78877	65.810	65.810
Total		119855	100.000	100.000

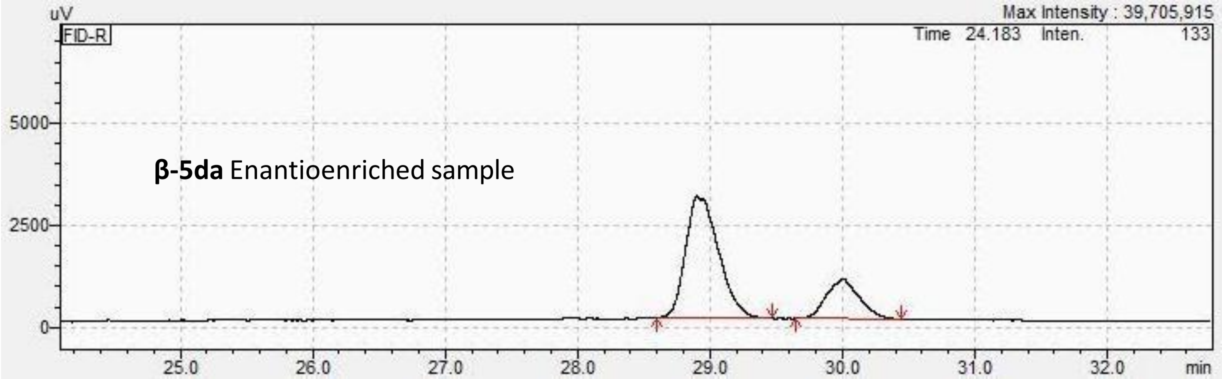

Peak#	Ret. Time	Area	Conc.	Area%
1	28.899	51232	75.606	75.606
2	30.013	16530	24.394	24.394
Total		67761	100.000	100.000

Chiral GC data for the same experiment but starting from (*E*)-**4d** and ethanol with **L6** as ligand.

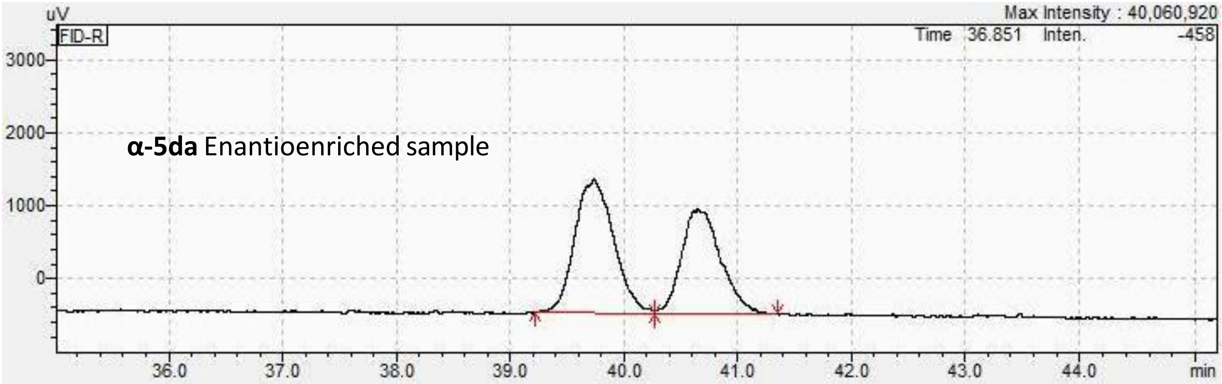

Peak#	Ret. Time	Area	Conc.	Area%
1	39.737	43773	56.092	56.092
2	40.648	34264	43.908	43.908
Total		78037	100.000	100.000

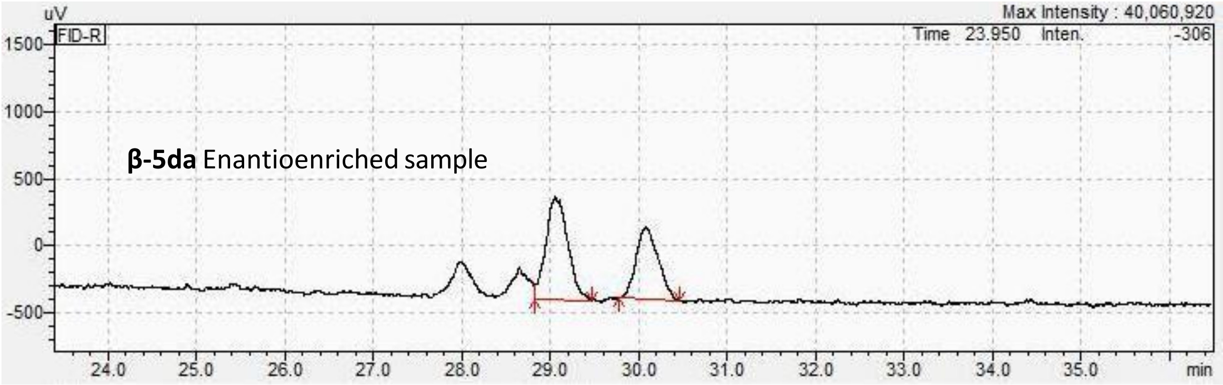

Peak#	Ret. Time	Area	Conc.	Area%
1	29.055	13302	58.967	58.967
2	30.080	9256	41.033	41.033
Total		22558	100.000	100.000

Ethyl (S)-2-phenylbutanoate (5ea):

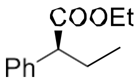

The compound was prepared according to the general procedure by reaction of cis-β-methylstyrene (66 µL, 0.50 mmol) and ethanol (15 µL, 0.25 mmol), $PdBr_2(ACN)_2$ (1.8 mg, 0.005 mmol), **L6** (5.3 mg, 0.01 mmol), at 45 °C for 48 h, and was isolated by column chromatography (silica gel, petroleum ether/dichloromethane = 8/2), yielding the title product as a colourless oil (39 mg, 80%). The NMR data match those reported in the literature for the racemic product.[5]

^{1}H NMR (500 MHz, $CDCl_3$) δ = 7.34 – 7.29 (m, 4H), 7.28 – 7.23 (m, 1H), 4.20 – 4.05 (m, 2H), 3.47 – 3.41 (m, 1H), 2.16 – 2.05 (m, 1H), 1.85 – 1.74 (m, 1H), 1.21 (t, *J* = 7.2 Hz, 3H), 0.90 (t, *J* = 7.3 Hz, 3H).

^{13}C-{^{1}H} NMR (126 MHz, $CDCl_3$) δ = 174.2, 139.4, 128.6, 128.1, 127.2, 60.7, 53.7, 26.9, 14.3, 12.3.

$[\alpha]_D^{23.4}$ = −0.2 (c = 0.4, $CHCl_3$).

GC analysis (CP-Chirasil-Dex CB Column 25 m x 0.25 mm x 0.25 µm, flow: 2.14 mL/min, 72 °C): t_1 = 110.91 min (minor), t_2 = 112.87 min (major). The absolute configuration of **5ea** was assigned as (*S*)-enantiomer by analogy to *(S)*-**3aa**.

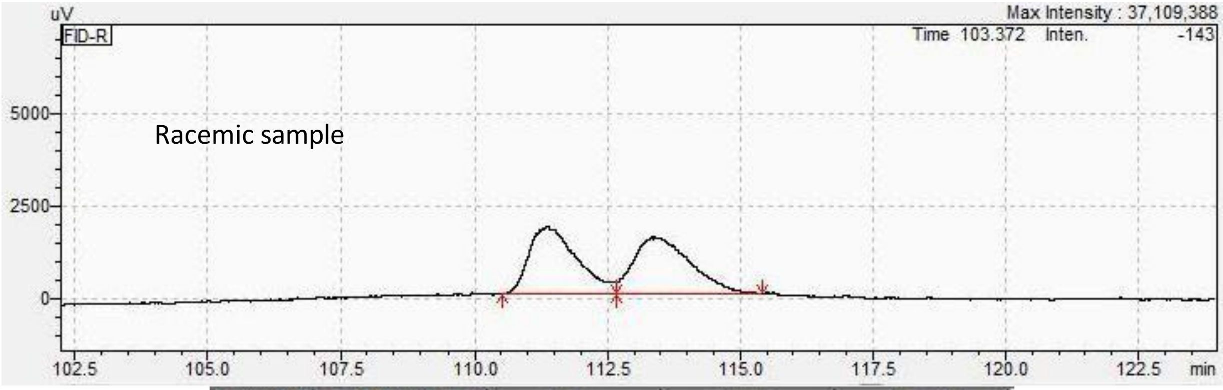

Peak#	Ret. Time	Area	Conc.	Area%
1	111.365	113705	49.937	49.937
2	113.323	113990	50.063	50.063
Total		227696	100.000	100.000

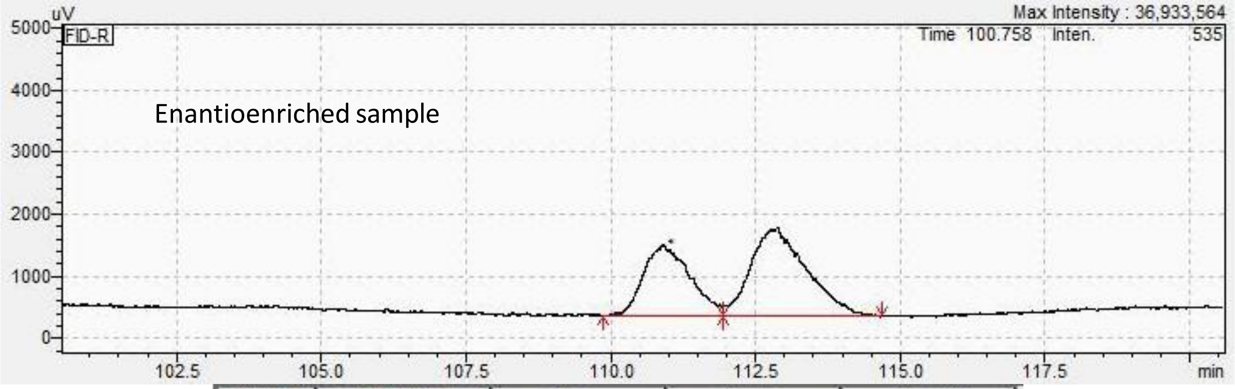

Peak#	Ret. Time	Area	Conc.	Area%
1	110.912	63464	40.206	40.206
2	112.868	94383	59.794	59.794
Total		157848	100.000	100.000

Chiral GC data for the same experiment but starting from (*Z*)-**4e** and ethanol with **L10** as ligand.

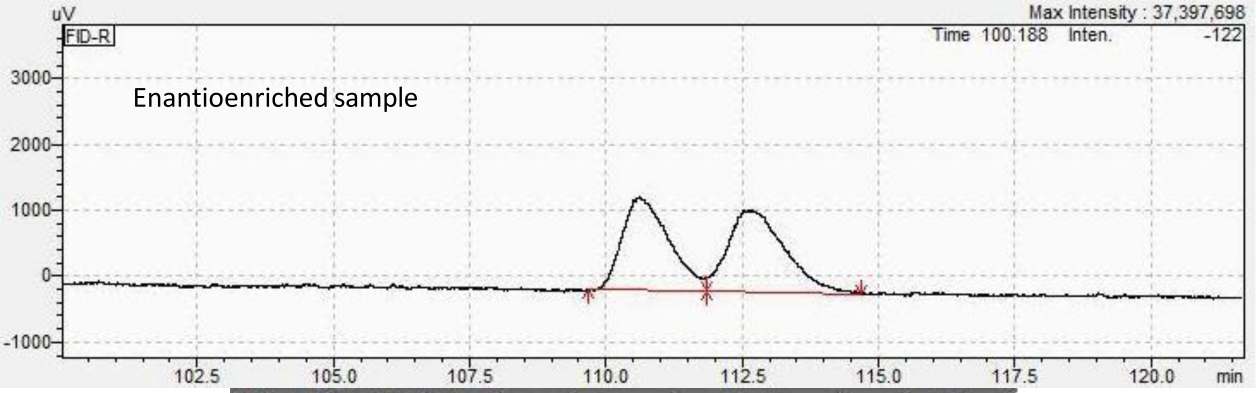

Peak#	Ret. Time	Area	Conc.	Area%
1	110.614	84449	48.267	48.267
2	112.627	90511	51.733	51.733
Total		174960	100.000	100.000

Chiral GC data for the same experiment but starting from(*Z*)-**4e** and ethanol with **L5** as ligand.

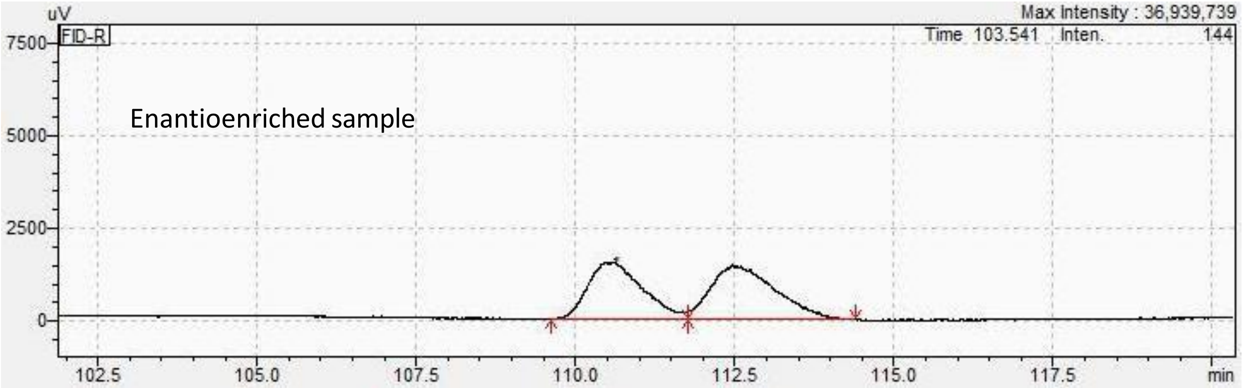

Peak#	Ret. Time	Area	Conc.	Area%
1	110.537	91572	47.004	47.004
2	112.464	103245	52.996	52.996
Total		194817	100.000	100.000

Chiral GC data for the same experiment but starting from(*E*)-**4e** and ethanol with **L6** as ligand.

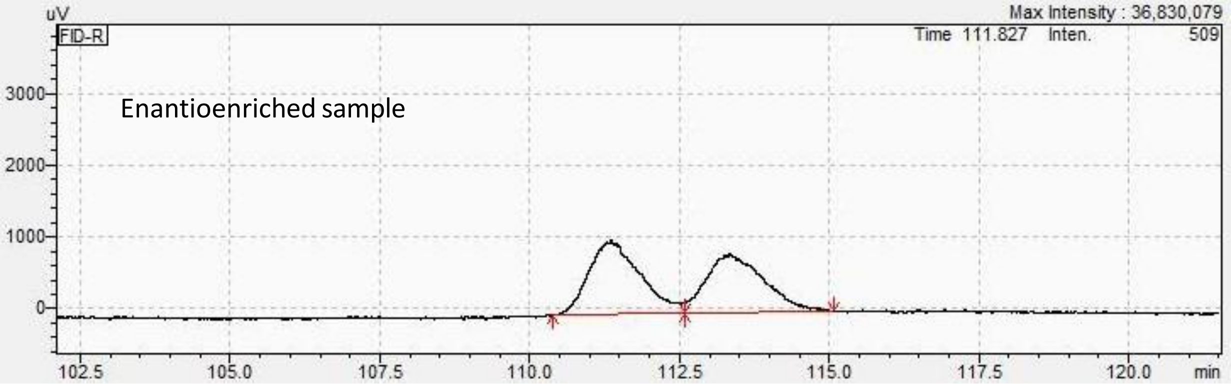

Peak#	Ret. Time	Area	Conc.	Area%
1	111.366	60992	53.041	53.041
2	113.340	53997	46.959	46.959
Total		114989	100.000	100.000

Chiral GC data for the same experiment but starting from (*E*)-**4e** and ethanol with **L5** as ligand.

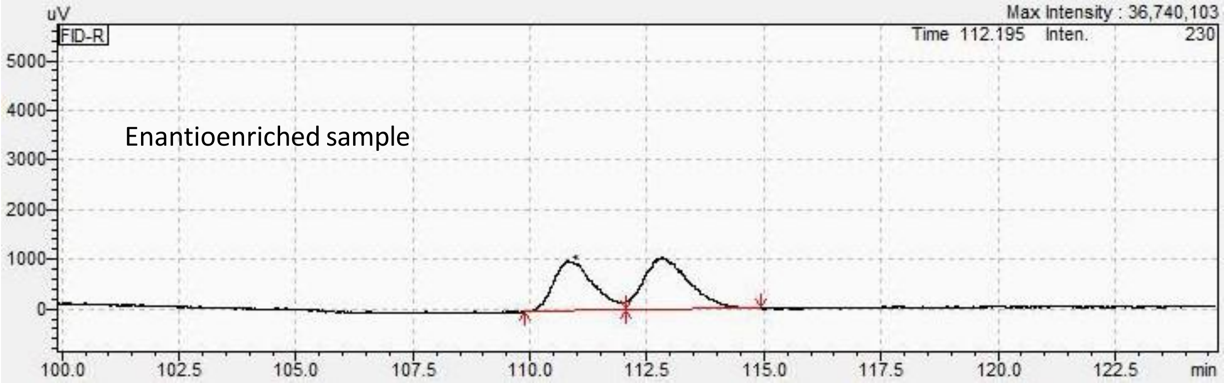

Peak#	Ret. Time	Area	Conc.	Area%
1	110.816	59729	45.706	45.706
2	112.815	70953	54.294	54.294
Total		130681	100.000	100.000

Ethyl bicyclo[2.2.1]heptane-2-carboxylate (5fa)

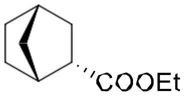

The compound was prepared according to the general procedure by reaction of norbornene (47 mg, 0.50 mmol) and ethanol (15 µL, 0.25 mmol), $PdBr_2(ACN)_2$ (1.8 mg, 0.005 mmol), **L5** (3.3 mg, 0.01 mmol), at 45 °C for 48 h, and was isolated by column chromatography (silica gel, petroleum ether/dichloromethane = 9/1), yielding the title product as a colourless oil (28 mg, 67%).

^{1}H NMR (500 MHz, $CDCl_3$) δ 4.15 – 4.04 (m, 2H), 2.51 – 2.45 (m, 1H), 2.33 – 2.24 (m, 2H), 1.87 – 1.76 (m, 1H), 1.59 – 1.40 (m, 4H), 1.26 – 1.22 (m, 4H), 1.19 – 1.13 (m, 2H).

^{13}C-{^{1}H} NMR (126 MHz, $CDCl_3$) δ 176.3, 60.3, 46.7, 41.1, 36.6, 36.2, 34.2, 29.6, 28.8, 14.4.

HRMS (ESI) m/z calcd. for $C_{10}H_{17}O_2$ ($[M+H]^+$): 169.1223; found: 169.1221.

82:18 er.

GC analysis (CP-Chirasil-Dex CB Column 25 m x 0.25 mm x 0.25 µm, flow: 2.16 mL/min, 70 °C): t_1 = 79.61 min (major), t_2 = 81.64 min (minor). The absolute configuration of **5fa** was not assigned.

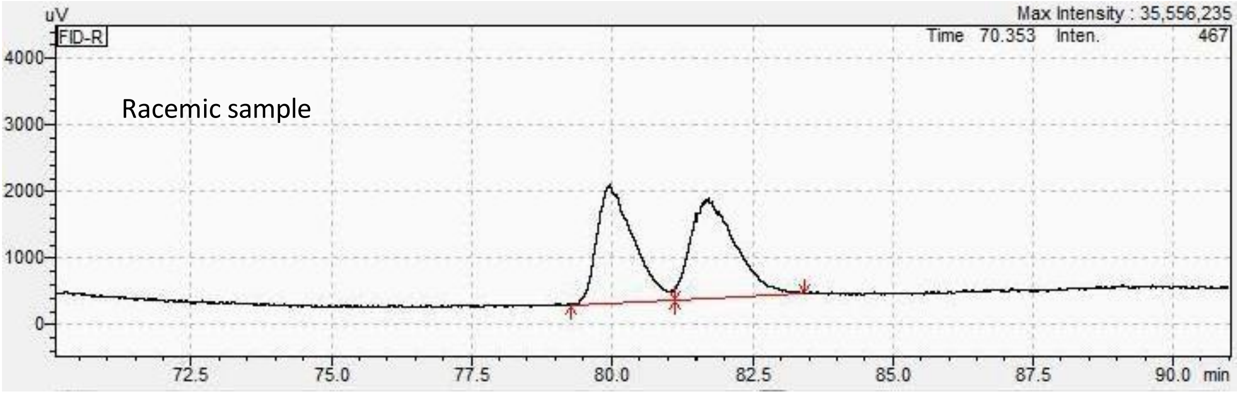

Peak#	Ret. Time	Area	Conc.	Area%
1	79.976	79457	49.878	49.878
2	81.702	79846	50.122	50.122
Total		159304	100.000	100.000

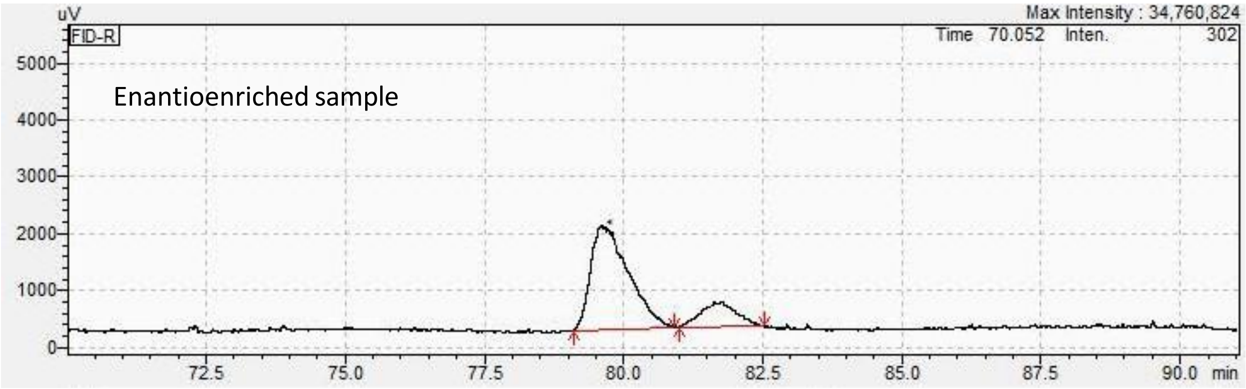

Peak#	Ret. Time	Area	Conc.	Area%
1	79.613	85645	81.842	81.842
2	81.635	19002	18.158	18.158
Total		104647	100.000	100.000

Chiral GC data for the same experiment but starting from norbornene and ethanol with **L10** as ligand.

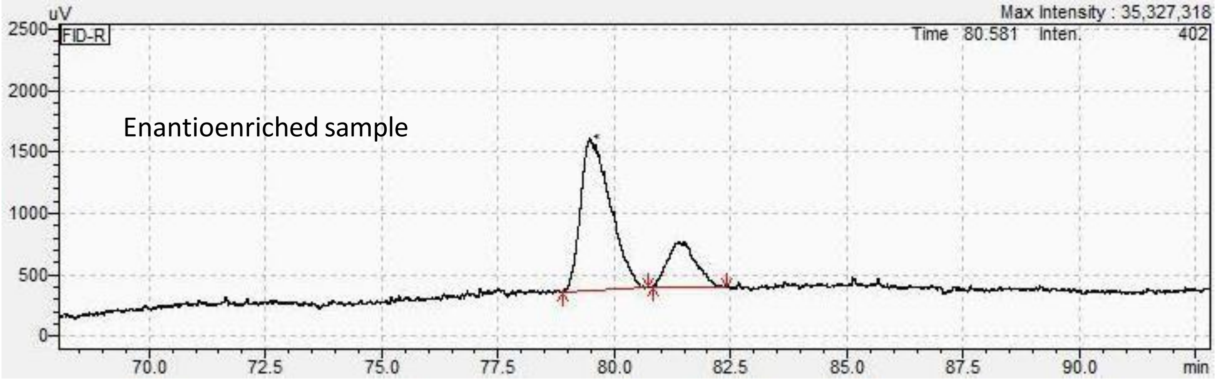

Peak#	Ret. Time	Area	Conc.	Area%
1	79.480	54738	78.168	78.168
2	81.392	15288	21.832	21.832
Total		70026	100.000	100.000

Chiral GC data for the same experiment but starting from norbornene and ethanol with **L6** as ligand.

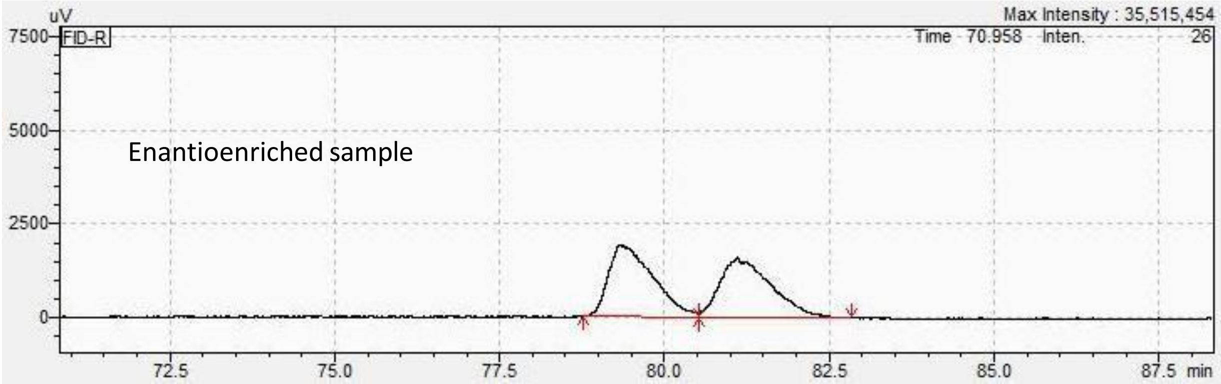

Peak#	Ret. Time	Area	Conc.	Area%
1	79.335	89915	51.441	51.441
2	81.103	84877	48.559	48.559
Total		174792	100.000	100.000

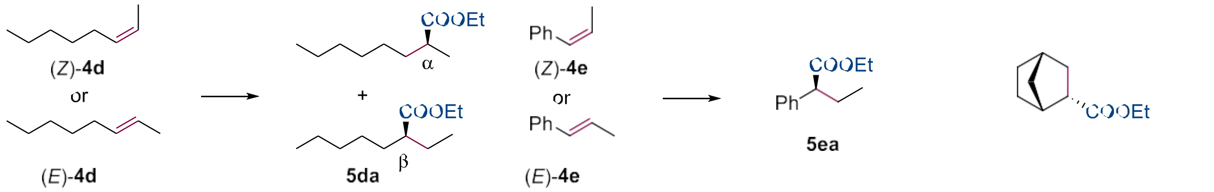

for (*Z*)-**4d**:
74:26 er(α), 81:19 er(β), 82:18 rr, 67%
(w/ **L6**): 60:40 er(α), 51:49 er(β), 81:19 rr, 89%[a,b]
(w/ **L5**): 45:55 er(α), 68:32 er(β), 65:35 rr, 98%[a,c]
for (*E*)-**4d**:
88:12 er(α), 68:32 er(β), 87:13 rr, 15%[a]
(w/ **L6**): 56:44 er(α), 59:41 er(β), 74:26 rr, 98%[a,b]
(w/ **L5**): 34:66 er(α), 76:24 er(β), 65:35 rr, 98%[a,c]

for (*Z*)-**4e**:
48:52 er, 88:12 rr, 20%[a]
(w/ **L6**): 40:60 er, 94:6 rr, 80%[b]
(w/ **L5**): 47:53 er, >98:2 rr, 99%[a,c]
for (*E*)-**4e**:
95:5 rr, 9%[a]
(w/ **L6**): 53:47 er, 95:5 rr, 67%[a,b]
(w/ **L5**): 46:54 er, >98:2 rr, 60%[a,c]

5fa,
78:22 er, >95:5 dr, 48%[a]
(w/ **L6**): 51:49 er, >95:5 dr, 85%[a,b]
(w/ **L5**): 18:82 er, >95:5 dr, 67%[c]

Fig. S2. Enantio- and regioselective alkoxycarbonylation of 1,2-bissubstituted alkenes – effect of ligand structure. Unless noted otherwise, er's and yields of isolated materials; dr's and rr's for the reaction mixture; [a] NMR yield. [b] **L6**.[c] **L5**.

Ethyl 2,2-dimethylundecanoate (5ga):

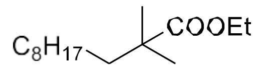

The compound was prepared according to the general procedure by reaction of 2-methyl-1-undecene (112 µL, 0.50 mmol) and ethanol (15 µL, 0.25 mmol), $PdBr_2(ACN)_2$ (3.6 mg, 0.01 mmol), rac-BIDIME (6.6 mg, 0.02 mmol), at 45 ^oC for 48 h, and was isolated by column chromatography (silica gel, petroleum ether/dichloromethane = 9/1), yielding the title product as a colourless oil (40 mg, 33%).

^{1}H NMR (500 MHz, $CDCl_3$) δ = 4.10 (q, *J* = 7.1 Hz, 2H), 1.54 – 1.42 (m, 2H), 1.30 – 1.20 (m, 17H), 1.14 (s, 6H), 0.87 (t, *J* = 7.0 Hz, 3H).

^{13}C NMR (126 MHz, $CDCl_3$) δ = 178.3, 60.3, 42.3, 40.9, 32.0, 30.2, 29.7, 29.7, 29.5, 25.3, 25.0, 22.8, 14.4, 14.2.

Ethyl 2-methyl-2-phenylpropanoate (5ha):

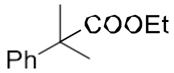

The compound was prepared according to the general procedure by reaction of α-methylstyrene (65 µL, 0.50 mmol) and ethanol (15 µL, 0.25 mmol), $PdBr_2(ACN)_2$ (3.6 mg, 0.01 mmol), rac-BIDIME (6.6 mg, 0.02 mmol), at 45 ^oC for 48 h, and was isolated by column chromatography (silica gel, petroleum ether/dichloromethane = 8/2), yielding the title product as a colourless oil (11 mg, 22%). The NMR data match those reported in the literature.[6]

^{1}H NMR (500 MHz, $CDCl_3$) δ = 7.36 – 7.30 (m, 4H), 7.26 – 7.21 (m, 1H), 4.12 (q, *J* = 7.1 Hz, 2H), 1.58 (s, 6H), 1.18 (t, *J* = 7.1 Hz, 3H).

^{13}C NMR (126 MHz, $CDCl_3$) δ = 176.9, 145.0, 128.5, 126.7, 125.8, 60.9, 46.6, 26.7, 14.2.

HRMS (ESI) m/z calcd. For $C_{14}H_{16}O_4N$ ([M+H]+): 262.1074; found:262.1068.

7. Synthesis and characterisation of starting materials

11-Chloroundec-1-ene (1j):

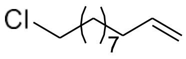

The title compound was prepared according to a literature procedure.[7] A solution of undec-10-en-1-ol (2.0 mL, 10.0 mmol) and triphenylphosphine (3.2 g, 12 mmol) in CCl_4 (5 mL) was refluxed for 3 h. After cooling to room temperature, petroleum ether (15 mL) was added, resulting in the formation of a white precipitate. The precipitate was removed by filtration, and the filtrate was concentrated under reduced pressure. The crude product was purified by column chromatography (silica gel, 0-1% EtOAc in petroleum ether), affording the product as a colourless oil (1.6 g, 85%). The NMR data match previously reported data for the title compound.[7]

^{1}H NMR (500 MHz, $CDCl_3$) δ = 5.81 (ddt, *J* = 16.9, 10.2, 6.7 Hz, 1H), 5.02 – 4.92 (m, 2H), 3.53 (t, *J* = 6.8 Hz, 2H), 2.04 (q, *J* = 6.8 Hz, 2H), 1.77 (p, *J* = 6.9 Hz, 2H), 1.45 – 1.26 (m, 12H).

11-Bromoundec-1-ene (1k):

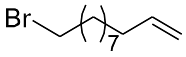

The title compound was prepared according to a literature procedure.[8] To a mixture of undec-10-en-1-ol (2.6 g, 15.3 mmol) and CBr_4 (6.5 g, 19.5 mmol) in dry CH_2Cl_2 (25 mL) was added portionwise triphenylphosphine (5.1 g, 19.5 mmol), and the resulting mixture was stirred at room temperature for 3 h. The reaction mixture was filtered, and the filtrate was concentrated under reduced pressure. The crude product was purified by column chromatography (silica gel, 0-1% EtOAc in petroleum ether), affording the product as a pale yellow oil (3.2 g, 91%). The NMR data match previously reported data for the title compound.[8]

^{1}H NMR (500 MHz, $CDCl_3$) δ = 5.81 (ddt, *J* = 16.9, 10.2, 6.7 Hz, 1H), 5.02 – 4.92 (m, 2H), 3.41 (t, *J* = 6.9 Hz, 2H), 2.04 (q, *J* = 6.8 Hz, 2H), 1.85 (p, *J* = 7.1 Hz, 2H), 1.44 – 1.26 (m, 12H).

***tert*-Butyldiphenyl(undec-10-en-1-yloxy)silane (1l):**

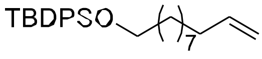

The title compound was prepared according to a literature procedure.[9] To a mixture of undec-10-en-1-ol (2.55 g, 15 mmol), DMAP (92.0 mg, 0.75 mmol), and Et_3N (3.0 mL, 22.0 mmol) in dry CH_2Cl_2 (50 mL) was added *tert*-butyl(chloro)diphenylsilane (5.0 g, 18.0 mmol). The resulting mixture was stirred at room temperature for 18 h. The reaction mixture was quenched with saturated aqueous $NaHCO_3$, and the organic layer was extracted with EtOAc (3 × 50 mL). The combined organic layers were dried over Na_2SO_4, filtered, and concentrated under reduced pressure. The crude product was purified by column chromatography (silica gel, 0-3% EtOAc in petroleum ether), affording the product as a colourless oil (5.3 g, 87%). The NMR data match previously reported data for the title compound.[9]

^{1}H NMR (400 MHz, $CDCl_3$) δ = 7.70 – 7.66 (m, 4H), 7.44 – 7.36 (m, 6H), 5.82 (ddt, *J* = 16.8, 10.2, 6.7 Hz, 1H), 5.03 – 4.92 (m, 2H), 3.66 (t, *J* = 6.5 Hz, 2H), 2.05 (q, *J* = 6.9 Hz, 2H), 1.59 – 1.53 (m, 2H), 1.41 – 1.23 (m, 12H), 1.06 (s, 9H).

Triisopropyl(undec-10-en-1-yloxy)silane (1l'):

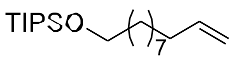

The title compound was prepared according to a modified literature procedure.[10] Undec-10-en-1-ol (1.2 mL, 6.2 mmol) was added to a solution of imidazole (0.8 g, 12.4 mmol) and DMAP (0.15 g, 1.2 mmol) in dry DMF (15 mL) under a nitrogen atmosphere. The reaction mixture was cooled to 0 °C, and TIPSCl (1.6 mL, 7.4 mmol) was added dropwise. The resulting mixture was allowed to warm to room temperature and stirred for 18 h. The reaction mixture was diluted with H_2O (20 mL) and extracted with petroleum ether (3 × 20 mL). The combined organic layers were washed with

saturated aqueous $NaHCO_3$ (30 mL) and brine (30 mL), dried over Na_2SO_4, filtered, and concentrated under reduced pressure. The crude product was purified by column chromatography (silica gel, 0-2% EtOAc in petroleum ether), affording the product as a colourless oil (1.8 g, 89%). The NMR data match previously reported data for the title compound.[11]

^{1}H NMR (500 MHz, $CDCl_3$) δ = 5.81 (ddt, *J* = 16.9, 10.2, 6.7 Hz, 1H), 5.01 – 4.91 (m, 2H), 3.67 (t, *J* = 6.7 Hz, 2H), 2.04 (q, *J* = 6.9 Hz, 2H), 1.56 – 1.50 (m, 2H), 1.40 – 1.26 (m, 12H), 1.11 – 1.03 (m, 21H).

((Undec-10-en-1-yloxy)methyl)benzene (1m):

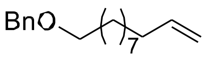

The title compound was prepared according to a procedure adapted from the literature.[12] Undec-10-en-1-ol (1.5 g, 8.7 mmol) was added dropwise to a suspension of NaH (0.70 g of a 60% dispersion in mineral oil, 0.42 g, 17.4 mmol) and tetrabutylammonium iodide (100 mg, 0.27 mmol) in dry THF (10 mL) at 0 °C under a nitrogen atmosphere. The resulting mixture was stirred for 15 min, after which benzyl bromide (1.2 mL, 10.4 mmol) was added. The reaction mixture was stirred at room temperature for 18 h. The reaction was quenched with saturated aqueous NH_4Cl (~7.5 mL), and the organic layer was extracted with Et_2O (3 × 20 mL). The combined organic layers were dried over Na_2SO_4, filtered, and concentrated under reduced pressure. The crude product was purified by column chromatography (silica gel, 0-5% EtOAc in petroleum ether), affording the product as a colourless oil (2.1 g, 93%). The NMR data match previously reported data for the title compound.[13]

^{1}H NMR (500 MHz, $CDCl_3$) δ = 7.37 – 7.33 (m, 4H), 7.30 – 7.26 (m, 1H), 5.82 (ddt, *J* = 16.9, 10.2, 6.7 Hz, 1H), 5.02 – 4.91 (m, 2H), 4.51 (s, 2H), 3.47 (t, *J* = 6.7 Hz, 2H), 2.04 (tdd, *J* = 6.5, 5.3, 1.4 Hz, 2H), 1.61 (p, *J* = 6.7 Hz, 2H), 1.41 – 1.25 (m, 12H).

Phenyl(undec-10-en-1-yl)sulfane (1n):

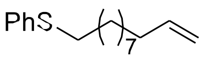

The title compound was prepared according to a modified literature procedure.[14] To a solution of thiophenol (0.8 mL, 7.8 mmol) in DMF (15 mL) was added K_2CO_3 (1.4 g, 10.0 mmol), followed by 11-bromoundec-1-ene (1.8 g, 7.8 mmol). The reaction mixture was stirred at room temperature for 18 h and then diluted with H_2O (25 mL). The organic layer was extracted with petroleum ether (3 × 30 mL), and the combined organic layers were dried over Na_2SO_4, filtered, and concentrated under reduced pressure. The crude product was purified by column chromatography (silica gel, 100% petroleum ether), affording the product as a colourless oil (1.8 g, 90%). The NMR data match previously reported data for the title compound.[14]

^{1}H NMR (500 MHz, $CDCl_3$) δ = 7.34 – 7.31 (m, 2H), 7.29 – 7.25 (m, 2H), 7.18 – 7.14 (m, 1H), 5.81 (ddt, *J* = 16.9, 10.2, 6.7 Hz, 1H), 5.02 – 4.92 (m, 2H), 2.92 (t, *J* = 7.2 Hz, 2H), 2.04 (q, *J* = 7.2 Hz, 2H), 1.65 (p, *J* = 7.2 Hz, 2H), 1.43 – 1.25 (m, 12H).

1,1-Diphenylundec-10-en-1-ol (1o):

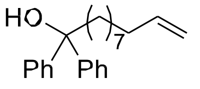

The title compound was prepared according to a modified literature procedure.[15] A solution of methyl undec-10-enoate (0.9 g, 4.6 mmol) in Et_2O (15 mL) was added dropwise over 1 h to a stirred solution of PhMgBr (3.0 M in Et_2O, 3.8 mL, 11.4 mmol) in Et_2O (15 mL) at 0 °C under a nitrogen atmosphere. The resulting mixture was allowed to warm to room temperature and stirred for an additional 3 h. The reaction was quenched by the addition of ice-cold saturated aqueous NH_4Cl. The reaction mixture was extracted with Et_2O (3 × 20 mL), and the combined organic layers were washed with saturated aqueous $NaHCO_3$ (20 mL) and brine (20 mL), dried over $MgSO_4$, filtered, and concentrated under reduced pressure. The crude product was purified by column chromatography (silica gel, 0-10% EtOAc in petroleum ether), affording the product as a colourless oil (1.1 g, 77%).

^{1}H NMR (400 MHz, $CDCl_3$) δ = 7.44 – 7.39 (m, 4H), 7.33 – 7.28 (m, 4H), 7.24 – 7.19 (m, 2H), 5.80 (ddt, *J* = 16.9, 10.2, 6.7 Hz, 1H), 5.01 – 4.90 (m, 2H), 2.30 – 2.24 (m, 2H), 2.12 – 2.00 (m, 3H), 1.39 – 1.20 (m, 12H).
^{13}C-{^{1}H} NMR (126 MHz, $CDCl_3$) δ = 147.3, 139.4, 128.3, 126.9, 126.2, 114.3, 78.4, 42.2, 33.9, 30.1, 29.6, 29.6, 29.2, 29.0, 23.9.
HRMS (ESI) m/z calcd. for $C_{23}H_{31}O$ ($[M+H]^+$): 323.2369; found: 323.2361.

Undec-10-en-1-yl 4-methylbenzenesulfonate (1p):

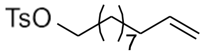

The title compound was prepared according to a procedure adapted from the literature.[16] TsCl (1.8 g, 9.6 mmol) and Et_3N (1.5 mL, 10.4 mmol) were added sequentially to a stirred solution of undec-10-en-1-ol (1.6 mL, 8.0 mmol) in CH_2Cl_2 (24 mL). The reaction mixture was stirred for 18 h and poured into H_2O (24 mL). The aqueous phase was extracted with EtOAc (3 × 30 mL). The combined organic layers were washed with brine (30 mL) and dried over Na_2SO_4. The mixture was filtered, concentrated and purified by column chromatography (silica gel, 0-5% EtOAc in petroleum ether), affording the pure product as a colourless oil (1.3 g, 50%). The NMR data match previously reported data for the title compound.[16]
^{1}H NMR (500 MHz, $CDCl_3$) δ = 7.82 – 7.75 (m, 2H), 7.38 – 7.31 (m, 2H), 5.80 (ddt, *J* = 16.9, 10.1, 6.7 Hz, 1H), 5.03 – 4.89 (m, 2H), 4.01 (t, *J* = 6.5 Hz, 2H), 2.45 (s, 3H), 2.08 – 1.96 (m, 2H), 1.62 (dq, *J* = 7.9, 6.5 Hz, 2H), 1.41 – 1.15 (m, 12H).

Dodec-11-enenitrile (1q):

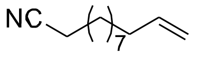

The title compound was prepared according to a procedure adapted from the literature.[17] NaCN (0.42 g, 8.6 mmol) was added to a solution of 11-bromoundec-1-ene (1.0 g, 4.3 mmol) in DMF (30 mL) at room temperature. The reaction mixture was stirred for 18 h, then diluted with H_2O (20 mL), and extracted with Et_2O (3 × 30 mL). The combined organic layers were washed with H_2O (3 × 20 mL) and brine (20 mL), dried over $MgSO_4$, filtered, and concentrated under reduced pressure, affording the product as a colourless oil (0.7 g, 89%). The NMR data match previously reported data for the title compound.[18]
^{1}H NMR (500 MHz, $CDCl_3$) δ = 5.81 (ddt, *J* = 16.9, 10.2, 6.7 Hz, 1H), 5.01 – 4.92 (m, 2H), 2.33 (t, *J* = 7.1 Hz, 2H), 2.04 (q, *J* = 6.7 Hz, 2H), 1.65 (p, *J* = 7.2 Hz, 2H), 1.46 – 1.25 (m, 12H).

Furan-2-ylmethyl undec-10-enoate (1s):

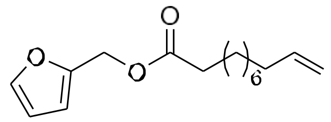

The title compound was prepared according to a modified literature procedure.[19] A mixture of 10-undecenoic acid (1.8 g, 10.0 mmol), DMAP (1.2 g, 10.0 mmol), DCC (2.1 g, 10.0 mmol), and furfuryl alcohol (0.9 mL, 10.0 mmol) in dry CH_2Cl_2 (50 mL) was stirred for 18 h at room temperature under a nitrogen atmosphere. The resulting precipitate was removed by filtration, and the filtrate was washed with H_2O (2 × 30 mL), followed by 5% aqueous AcOH (30 mL) and H_2O (2 × 30 mL). The organic layer was dried over Na_2SO_4, filtered, and concentrated under reduced pressure. The crude product was purified by column chromatography (silica gel, 0-5% EtOAc in petroleum ether), affording the product as a colourless oil (2.2 g, 85%).
^{1}H NMR (500 MHz, $CDCl_3$) δ = 7.42 (dd, *J* = 1.9, 0.8 Hz, 1H), 6.40 (dd, *J* = 3.3, 0.7 Hz, 1H), 6.36 (dd, *J* = 3.3, 1.8 Hz, 1H), 5.80 (ddt, *J* = 16.9, 10.2, 6.7 Hz, 1H), 5.06 (s, 2H), 5.01 – 4.91 (m, 2H), 2.32 (t, *J* = 7.6 Hz, 2H), 2.03 (q, *J* = 6.8 Hz, 2H), 1.65 – 1.59 (m, 2H), 1.39 – 1.25 (m, 10H).
^{13}C-{^{1}H} NMR (126 MHz, $CDCl_3$) δ = 173.6, 149.8, 143.3, 139.3, 114.3, 110.7, 110.6, 58.0, 34.3, 33.9, 29.4, 29.3, 29.2, 29.2, 29.0, 25.0.
HRMS (ESI) m/z calcd. for $C_{16}H_{26}O_3$ ($[M+2H]^{2+}$): 133.0935; found: 133.0926.

***S*-phenyl undec-10-enethioate (1t):**

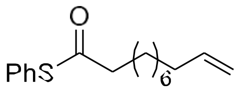

The title compound was prepared according to a modified literature procedure. **Step I.**[20] A solution of 10-undecenoic acid (1.5 g, 8.1 mmol) in dry CH_2Cl_2 (15 mL) was cooled to 0 °C under a nitrogen atmosphere, followed by the addition of a drop of DMF. Oxalyl chloride (1.0 mL, 12.1 mmol) was then added dropwise, and the resulting mixture was allowed to warm to room temperature and stirred for 2 h. The solvent was removed under reduced pressure, and the crude acid chloride was used directly in the next step.

Step II.[21] The crude acid chloride was dissolved in toluene (25 mL) and added dropwise over 30 min to a stirred solution of thiophenol (0.8 mL, 8.1 mmol) and Et_3N (1.1 mL, 8.1 mmol) in toluene (15 mL). The reaction mixture was stirred at room temperature for an additional 1 h, then washed with saturated aqueous $NaHCO_3$ (2 × 30 mL) and brine (30 mL). The organic layer was dried over $MgSO_4$, filtered, and concentrated under reduced pressure. The crude product was purified by column chromatography (silica gel, 0-5% EtOAc in petroleum ether), affording the product as a pale yellow oil (2.0 g, 90%). The NMR data match previously reported data for the title compound.[21]

^{1}H NMR (500 MHz, $CDCl_3$) δ = 7.42 – 7.39 (m, 5H), 5.81 (ddt, *J* = 16.9, 10.2, 6.7 Hz, 1H), 5.02 – 4.92 (m, 2H), 2.66 (t, *J* = 7.4 Hz, 2H), 2.04 (q, *J* = 6.8 Hz, 2H), 1.71 (p, *J* = 7.5 Hz, 2H), 1.40 – 1.26 (m, 10H).

***N*-methoxy-*N*-methylundec-10-enamide (1u):**

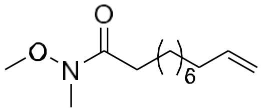

The title compound was prepared according to a modified literature procedure.[22] CDI (6.3 g, 39.0 mmol) was added to a stirred solution of 10-undecenoic acid (5.5 g, 30.0 mmol) in dry CH_2Cl_2 (100 mL) at 0 °C. The reaction mixture was stirred for 30 minutes. *N*-*O*-Dimethyl hydroxylamine·HCl (7.3 g, 75.0 mmol) was added to the reaction mixture and stirred at room temperature for 24 h. The resulting mixture was filtered and washed with CH_2Cl_2 (3 × 30 mL). The combined organic layers were washed with 1 N HCl (30 mL) and brine (30 mL), dried over Na_2SO_4 and concentrated under reduced pressure. The crude product was purified by column chromatography (silica gel, 0-10% EtOAc in petroleum ether), affording the product as a colourless oil (5.7 g, 83%). The NMR data match previously reported data for the title compound.[22]

^{1}H NMR (500 MHz, $CDCl_3$) δ = 5.80 (ddt, *J* = 16.9, 10.2, 6.7 Hz, 1H), 5.02 – 4.88 (m, 2H), 3.67 (s, 3H), 3.17 (s, 3H), 2.40 (t, *J* = 7.7 Hz, 2H), 2.07 – 1.97 (m, 2H), 1.67 – 1.56 (m, 2H), 1.40 – 1.22 (m, 10H).

1-Phenylundec-10-en-1-one (1w):

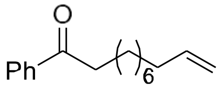

The title compound was prepared according to a procedure adapted from the literature.[23] *N*-methoxy-*N*-methylundec-10-enamide (3.4 g, 15.0 mmol) and dry THF (30 mL) were added to an oven-dried round-bottom flask. The solution was cooled to 0 °C and a solution of PhMgBr (3.0 M in Et_2O, 9 mL, 27.0 mmol) was added dropwise. The resulting mixture was stirred at 0 °C for 2 h. After completion of the reaction, as monitored by TLC, the reaction mixture was quenched by adding saturated aqueous NH_4Cl solution (5 mL/mmol). The organic phase was separated. The aqueous layer was extracted with EtOAc (3 × 20 mL). The combined organic layers were washed with brine (20 mL) and dried over Na_2SO_4. The crude product was purified by column chromatography (silica gel, 0-5% EtOAc in petroleum ether), affording the product as a colourless oil (3.3 g, 90%).

^{1}H NMR (500 MHz, $CDCl_3$) δ = 7.99 – 7.91 (m, 2H), 7.59 – 7.51 (m, 1H), 7.50 – 7.41 (m, 2H), 5.81 (ddt, *J* = 16.9, 10.1, 6.7 Hz, 1H), 5.04 – 4.87 (m, 2H), 3.03 – 2.89 (m, 2H), 2.10 – 1.94 (m, 2H), 1.73 (td, *J* = 7.7, 6.6 Hz, 2H), 1.44 – 1.22 (m, 10H).

^{13}C-{^{1}H} NMR (126 MHz, $CDCl_3$) δ = 200.7, 139.3, 137.2, 133.0, 128.7, 128.2, 114.3, 38.8, 33.9, 29.6, 29.5, 29.5, 29.2, 29.1, 24.5.

HRMS (ESI) m/z calcd. for $C_{17}H_{25}O$ ($[M+H]^+$): 245.1900; found: 245.1896.

2-(Dec-9-en-1-yl)-1,3-dioxolane (1y):

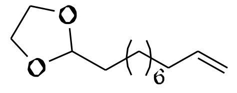

The title compound was prepared according to a modified literature procedure.[24] A round-bottom flask, equipped with a Dean–Stark apparatus, was charged with 10-undecenal (4.0 mL, 20.0 mmol), ethylene glycol (1.1 mL, 20.0 mmol), *p*-toluenesulfonic acid (138.0 mg, 0.8 mmol) and toluene (40 mL). The reaction mixture was heated at reflux for 8 h. The mixture was cooled, diluted with Et_2O and washed with H_2O (3 × 50 mL). The organic layer was dried over Na_2SO_4 and concentrated under reduced pressure. The crude product was purified by column chromatography (silica gel, 0-4% EtOAc in petroleum ether), affording the product as a colourless oil (4.1 g, 90%). The NMR data match previously reported data for the title compound.[24]

^{1}H NMR (500 MHz, CDCl$_3$) δ = 5.80 (ddt, *J* = 16.9, 10.2, 6.7 Hz, 1H), 5.02 – 4.88 (m, 2H), 4.83 (t, *J* = 4.9 Hz, 1H), 4.00 – 3.90 (m, 2H), 3.88 – 3.79 (m, 2H), 2.07 – 1.98 (m, 2H), 1.68 – 1.60 (m, 2H), 1.46 – 1.21 (m, 12H).

2-(Undec-10-en-1-yl)isoindoline-1,3-dione (1z):

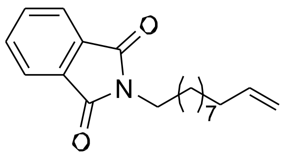

The title compound was prepared according to a modified literature procedure.[25] Undec-10-en-1-ol (2.0 mL, 10.0 mmol) was added to a solution of phthalimide (3.1 g, 21.0 mmol) and triphenylphosphine (5.3 g, 20.0 mmol) in Et_2O (27 mL) under an argon atmosphere. The reaction was cooled to 0 °C and diisopropylazodicarboxylate (3.4 mL, 20.0 mmol) was added dropwise. The reaction mixture was gradually warmed to room temperature and stirred for 18 h. The solid precipitate was filtered and washed with Et_2O. The filtrate was concentrated under reduced pressure. The crude product was purified by column chromatography (silica gel, 0-10% EtOAc in petroleum ether), affording the product as a white solid (1.8 g, 60%).

^{1}H NMR (500 MHz, CDCl$_3$) δ = 7.87 – 7.79 (m, 2H), 7.73 – 7.66 (m, 2H), 5.79 (ddt, *J* = 16.9, 10.1, 6.7 Hz, 1H), 5.03 – 4.86 (m, 2H), 3.71 – 3.61 (m, 2H), 2.06 – 1.97 (m, 2H), 1.71 – 1.59 (m, 2H), 1.41 – 1.20 (m, 12H).

^{13}C-{^{1}H} NMR (126 MHz, CDCl$_3$) δ = 168.6, 139.4, 134.0, 132.3, 123.3, 114.2, 38.2, 33.9, 29.54, 29.51, 29.3, 29.2, 29.0, 28.7, 27.0.

HRMS (ESI) m/z calcd. for $C_{19}H_{26}NO_2$ ($[M+H]^+$): 300.1958; found: 300.1949.

8. Mechanistic studies

a. Synthesis of deuterium-containing alkenes

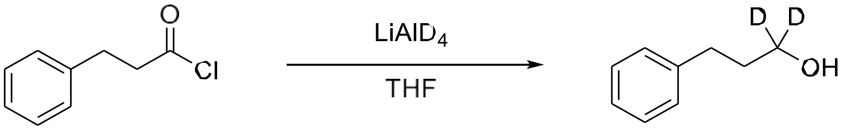

In a 100 mL round-bottom flask, equipped with a stirring bar, $LiAlD_4$ (1.49 g, 35.5 mmol) was added to dry tetrahydrofuran (47 mL). Subsequently, a solution of benzenepropanoyl chloride (877 mg in 10 mL of tetrahydrofuran) was slowly added into the suspension. The mixture was allowed to stir for 16 hours at room temperature. The mixture was then treated carefully with water (20 mL) and allowed to stir for 5 minutes. Next, the organic layer was extracted, dried over $MgSO_4$, and filtered. The volatiles from the filtrate were removed under reduced pressure, yielding the target alcohol (725 mg, 97%). The NMR data match those reported in the literature.[26]

^{1}H NMR (500 MHz, $CDCl_3$) δ 7.41 – 7.36 (m, 2H), 7.32 – 7.28 (m, 3H), 2.86 (s, 1H), 2.79 (t, *J* = 7.8 Hz, 2H), 1.97 (t, *J* = 7.9 Hz, 2H).

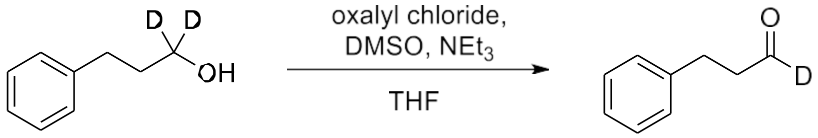

To a solution of DMSO (0.77 mL, 10.86 mmol) in tetrahydrofuran (24 mL) at -78 °C under nitrogen, oxalyl chloride (0.57 mL, 7.24 mmol) was added dropwise. The mixture was allowed to stir for 15 minutes. Next, 3-phenylpropan-1,1-d_2-1-ol (500 mg, 3.62 mmol) in tetrahydrofuran (24 mL) was added dropwise into the mixture. The mixture was stirred at -78 °C for 30 minutes. Triethylamine (21.72 mmol, 2.74 mL) was then added dropwise into the mixture. The mixture was stirred for 15 minutes at -78 °C, before being warmed to room temperature. The mixture was filtered to remove the precipitate, and the filtrate was concentrated under reduced pressure. The content was then diluted with dichloromethane (20 mL). The organic phase was washed with water (3 x 20 mL), dried over $MgSO_4$, and filtered. The volatiles from the filtrate were removed under reduced pressure. The residue was next subjected to column chromatography on silica gel (petroleum ether/ethyl acetate = 85/15) to afford the target product as a colourless oil (353 mg, 72%). The NMR data match those reported in the literature.[27]

^{1}H NMR (500 MHz, $CDCl_3$) δ 7.36 – 7.31 (m, 2H), 7.27 – 7.21 (m, 3H), 2.99 (t, *J* = 7.5 Hz, 2H), 2.81 (t, *J* = 7.5 Hz, 2H).

$$CD_3I \quad + \quad PPh_3 \xrightarrow[\text{THF, reflux}]{} [D_3CPPh_3]^+I^-$$

The compound was prepared following a literature procedure:[28] To a solution of triphenylphosphine (3.93 g, 15 mmol) in tetrahydrofuran (30 mL) under a nitrogen atmosphere, iodomethane-d_3 (1.18 mL, 18 mmol) was added dropwise. The mixture was refluxed for 1 hour. The reaction mixture was then allowed to cool to room temperature. The suspension was filtered through a sintered glass to collect the precipitate, which was then washed with xylene (3 x 20 mL) and dried under reduced pressure, affording the ylide salt as a white powder (4.76 g, 78%).

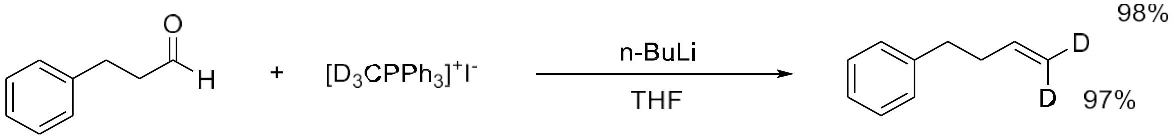

Under a nitrogen atmosphere, to a solution of CD_3PPh_3I (1.2 g, 2.5 mmol) in tetrahydrofuran (10 mL) at -78 °C, n-BuLi (1.6 mL from 1.6 M in hexanes, 2.5 mmol) was added dropwise over 5 minutes. The resulting mixture was allowed to stir for 15 minutes at -78 °C. Then, the mixture was allowed to warm to room temperature and stirred for an additional 30 minutes. Next, the reaction was cooled to -78 °C. Then, the aldehyde (0.33 mL in 2 mL tetrahydrofuran, 2.5 mmol) was added dropwise over 5 minutes. The resulting mixture was allowed to stir for 20 minutes at -78 °C. Then, the mixture was warmed to room temperature and stirred at this temperature for 16 hours. The mixture was concentrated under reduced pressure (100 mbar with the bath set at 25 °C to avoid evaporation of the target alkene product). The residue was subjected to column chromatography on silica gel, using n-pentane as an eluant, affording the target product as a colourless oil (230 mg, 68%). The NMR data match those reported in the literature.[29]

^{1}H NMR (400 MHz, $CDCl_3$) δ 7.32 – 7.26 (m, 2H), 7.24 – 7.16 (m, 3H), 5.86 (s, 1H), 2.75 – 2.68 (m, 2H), 2.41 – 2.34 (m, 2H).

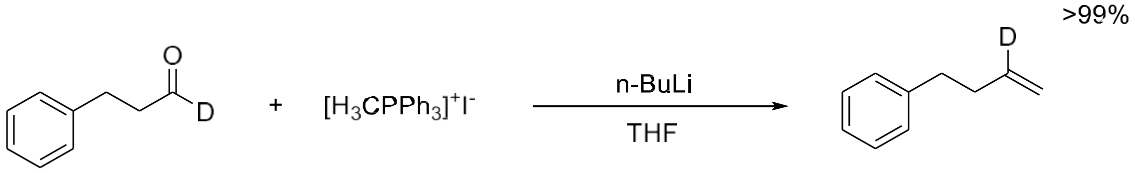

Under a nitrogen atmosphere, to a solution of CH_3PPh_3I (600 mg, 1.25 mmol) in dry tetrahydrofuran (5 mL) at -78 °C, n-BuLi (0.8 mL from 1.6 M in hexanes, 1.25 mmol) was added dropwise over 5 minutes. The resulting mixture was allowed to stir for 15 minutes at -78 °C. The mixture was allowed to warm to room temperature and stirred for an additional 30 minutes. Next, the reaction was cooled down to -78 °C. The aldehyde (175 mg in 2 mL tetrahydrofuran, 1.25 mmol) was added dropwise over 5 minutes. The resulting mixture was allowed to stir for 20 minutes at -78 °C. The mixture was warmed up to room temperature and stirred at this temperature for 16 hours. The reaction mixture was concentrated under reduced pressure (100 mbar with the bath set at 25 °C to avoid evaporation of the desired alkene product). The residue was subjected to column chromatography on silica gel, using n-pentane as an eluent, affording the target product as a colourless oil (91 mg, 54%). The NMR data match those reported in the literature. [30]

^{1}H NMR (400 MHz, $CDCl_3$) δ 7.37 – 7.27 (m, 2H), 7.22 – 7.14 (m, 3H), 5.08 – 4.93 (m, 2H), 2.76 – 2.67 (m, 2H), 2.38 (t, *J* = 7.8 Hz, 2H).

b. Deuterium-labelled experiments

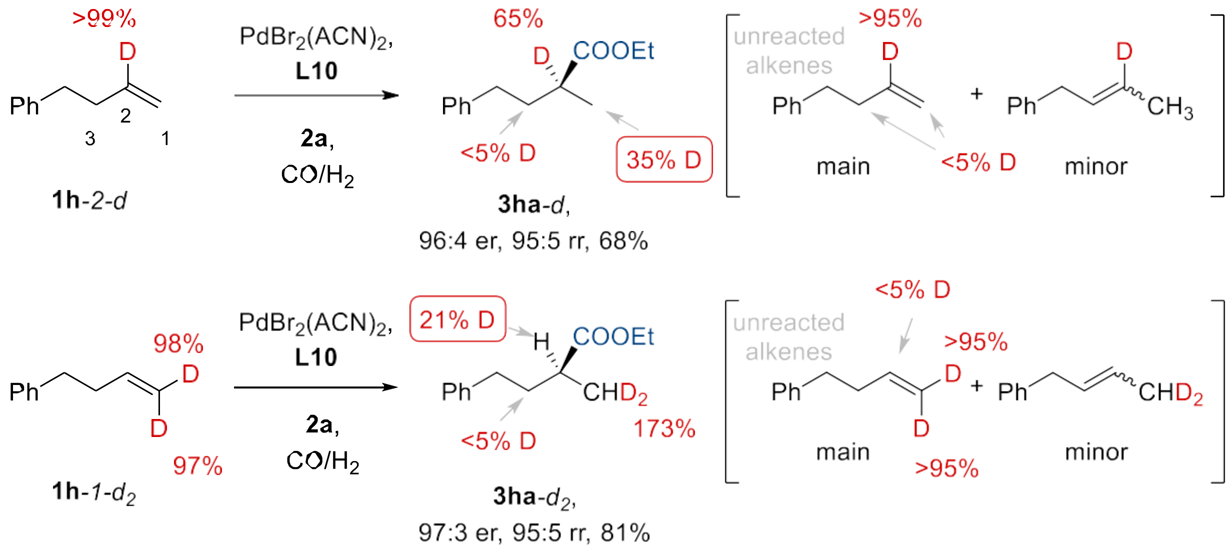

The experiments were performed following the general procedure described in Section 5, using **1h**-*2-d* (75 µL, 0.50 mmol) or **1h**-*1-d_2* (75 µL, 0.50 mmol) and ethanol **2a** (15 µL, 0.50 mmol). At the end of the reaction, the autoclave was allowed to cool to room temperature and the pressure was carefully released. The ^{1}H and ^{2}H NMR spectra and the GC-MS and GC-FID chromatograms were measured for the resulting reaction mixture to analyze the unreacted alkenes. The NMR sample was combined with the remaining reaction mixture (stored in the freezer during that time). The volatiles from the reaction mixture were removed under reduced pressure. The residue was subjected to column chromatography on silica gel (petroleum ether/dichloromethane = 8/2) affording the ester product as a colourless oil. The ^{1}H and ^{2}H NMR analysis of the isolated product was used to determine the deuterium incorporation. Specifically, the deuterium incorporation was calculated based on the quantitative ^{1}H NMR spectrum. The ^{2}H NMR spectrum confirmed the relative distribution of deuterium in the product.

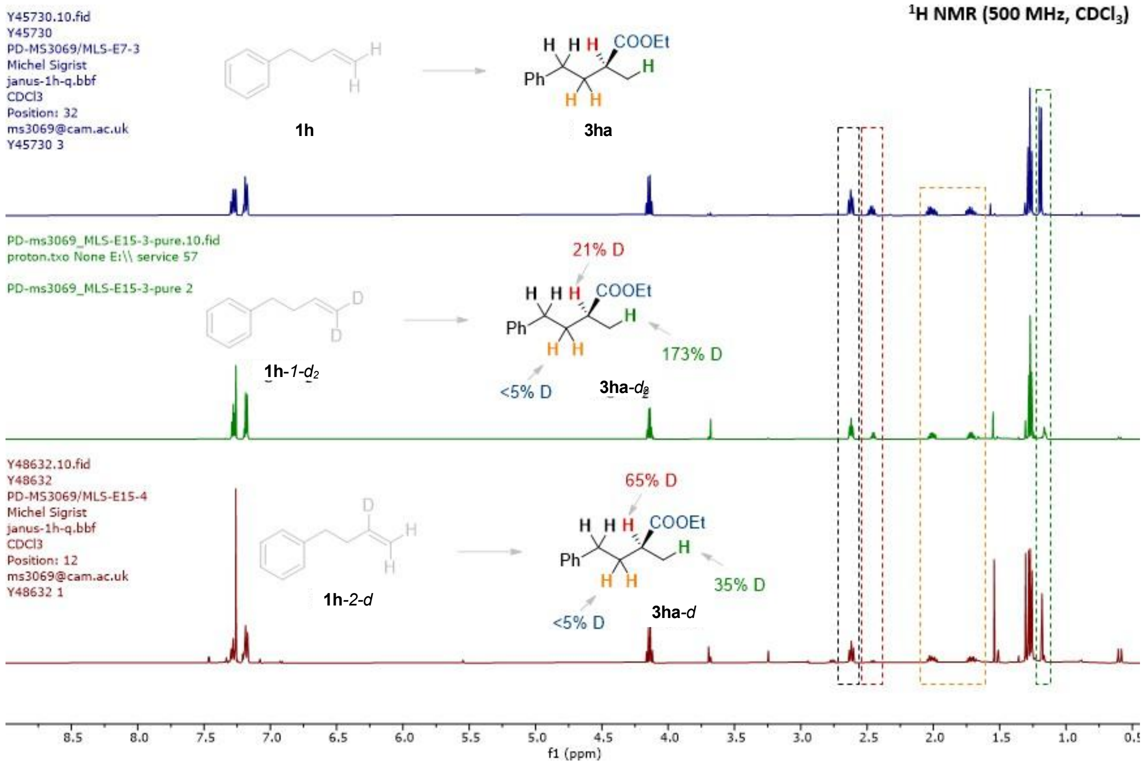

Fig. S3. *Comparison of the ^{1}H NMR spectra, measured in CDCl$_3$, of isolated esters* ***3ha***, ***3ha***-*d$_2$*, *or* ***3ha***-*d from the palladium-catalysed carbonylation of* ***1h***, ***1h***-*1-d$_2$*, *or* ***1h***-*2-d and ethanol* ***2a***.

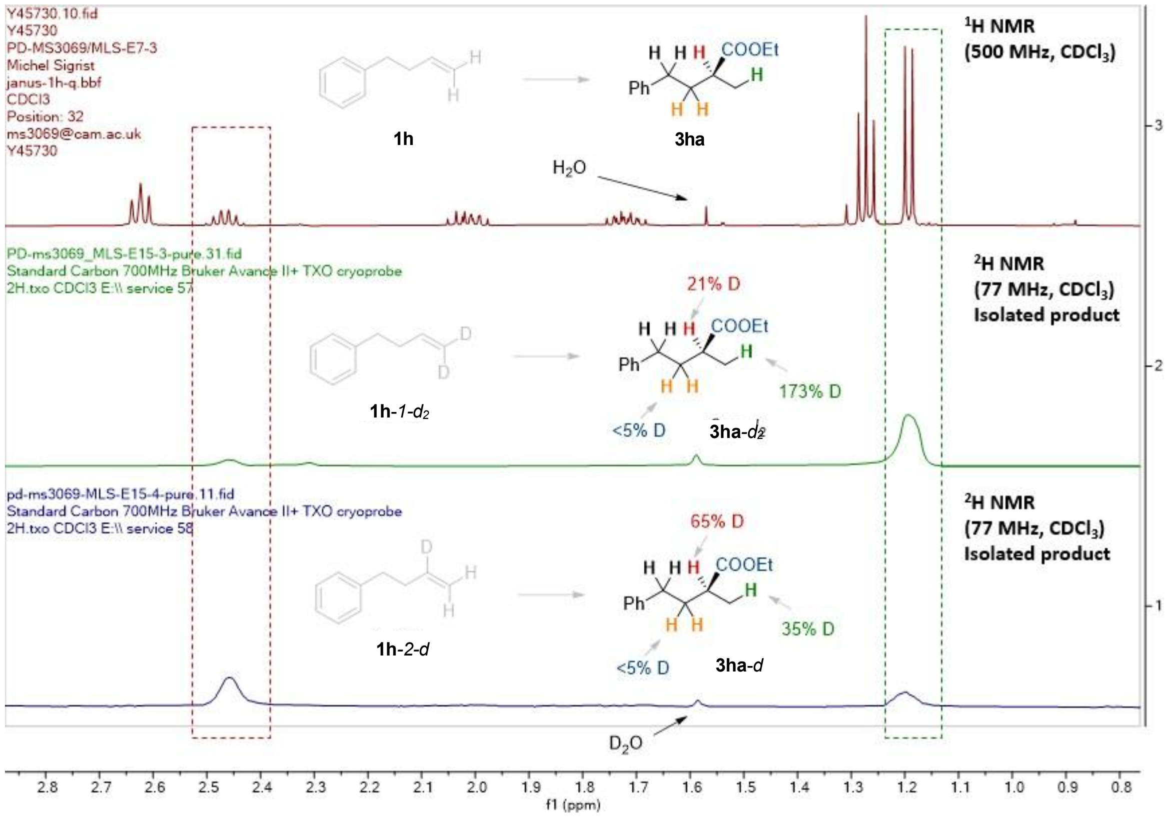

Fig. S4. *Comparison of the ^{1}H NMR spectrum, measured in CDCl$_3$, of isolated ester* ***3ha*** *from the palladium-catalysed carbonylation of* ***1h*** *and ethanol* ***2a***, *and the ^{2}H NMR spectrum of isolated esters* ***3ha***-*d$_2$ and* ***3ha***-*d from the palladium-catalysed carbonylation of* ***1h***-*1-d$_2$ or* ***1h***-*2-d and ethanol* ***2a***.

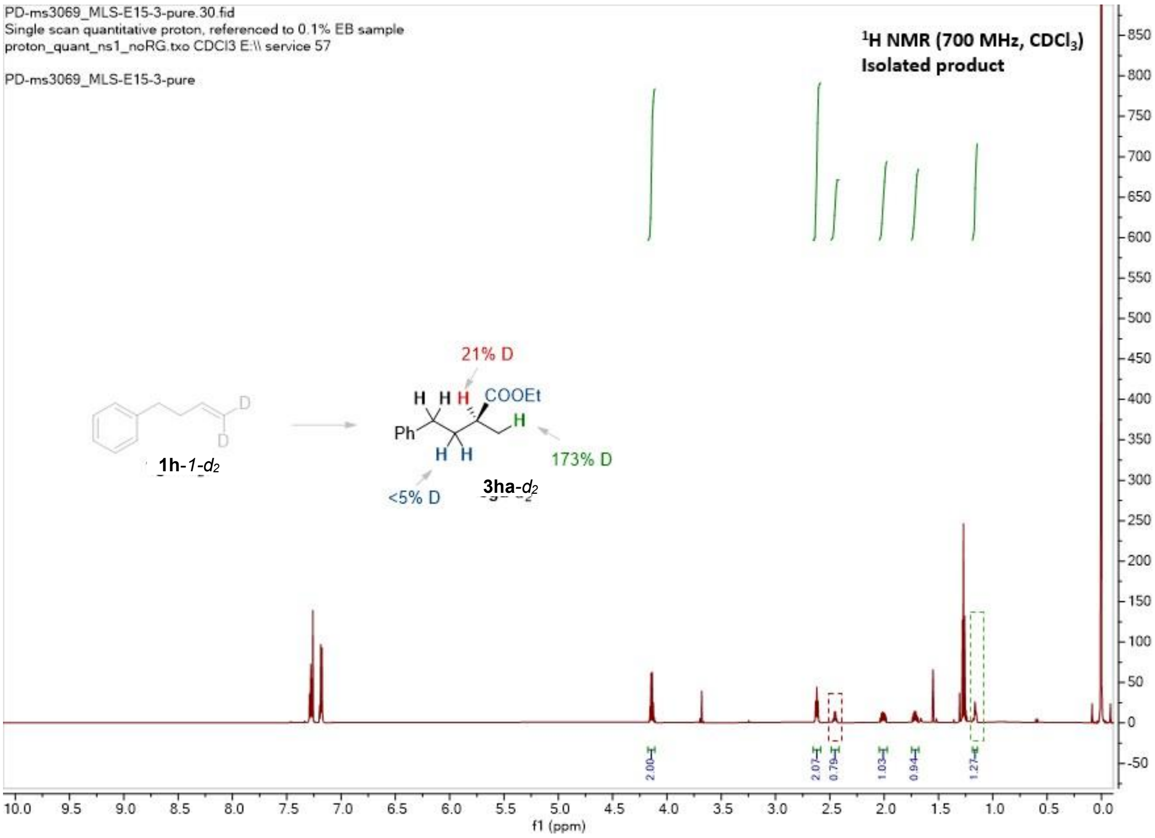

Fig. S5. *Quantitative* [1]*H NMR spectrum of isolated ester* ***3ha***-d_2*, from the palladium-catalysed carbonylation of* ***1h***-1-d_2 *and ethanol* ***2a***.

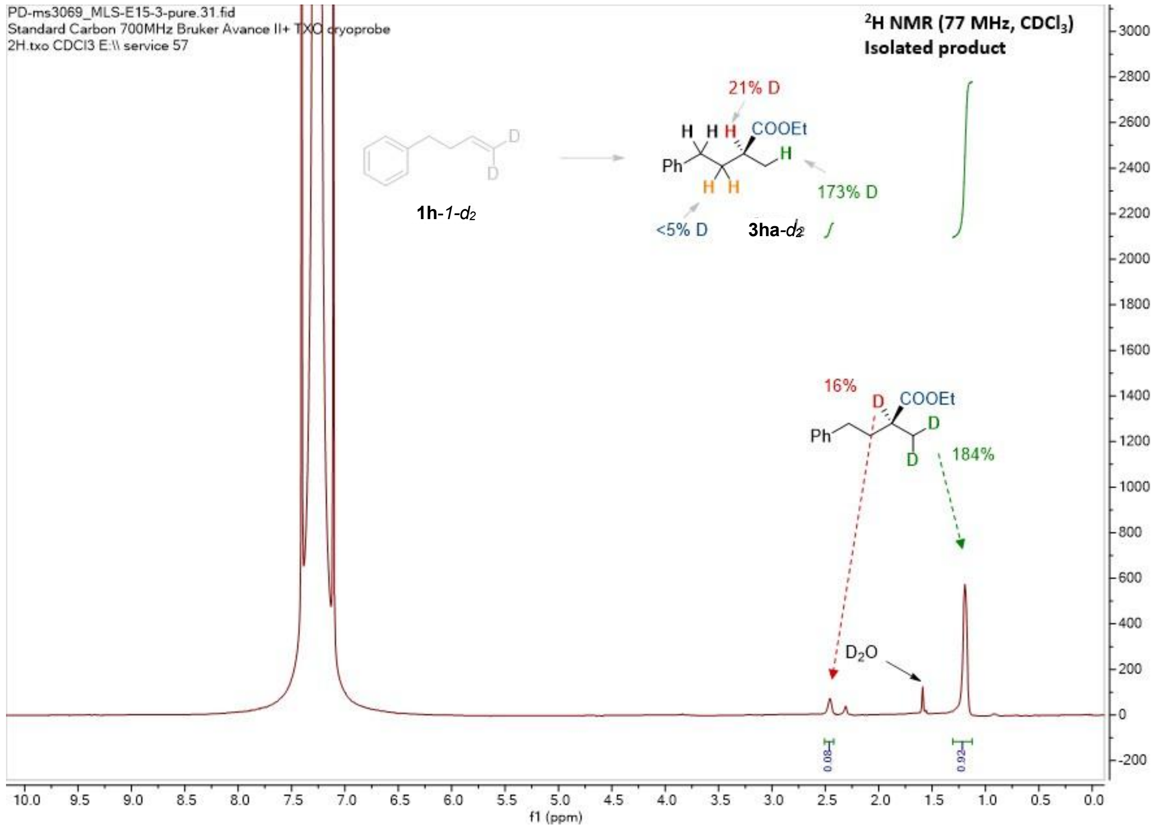

Fig. S6. ^{2}H NMR spectrum of isolated ester **3ha**-d_2, from the palladium-catalysed carbonylation of **1h**-1-d_2 *and ethanol* ***2a***.

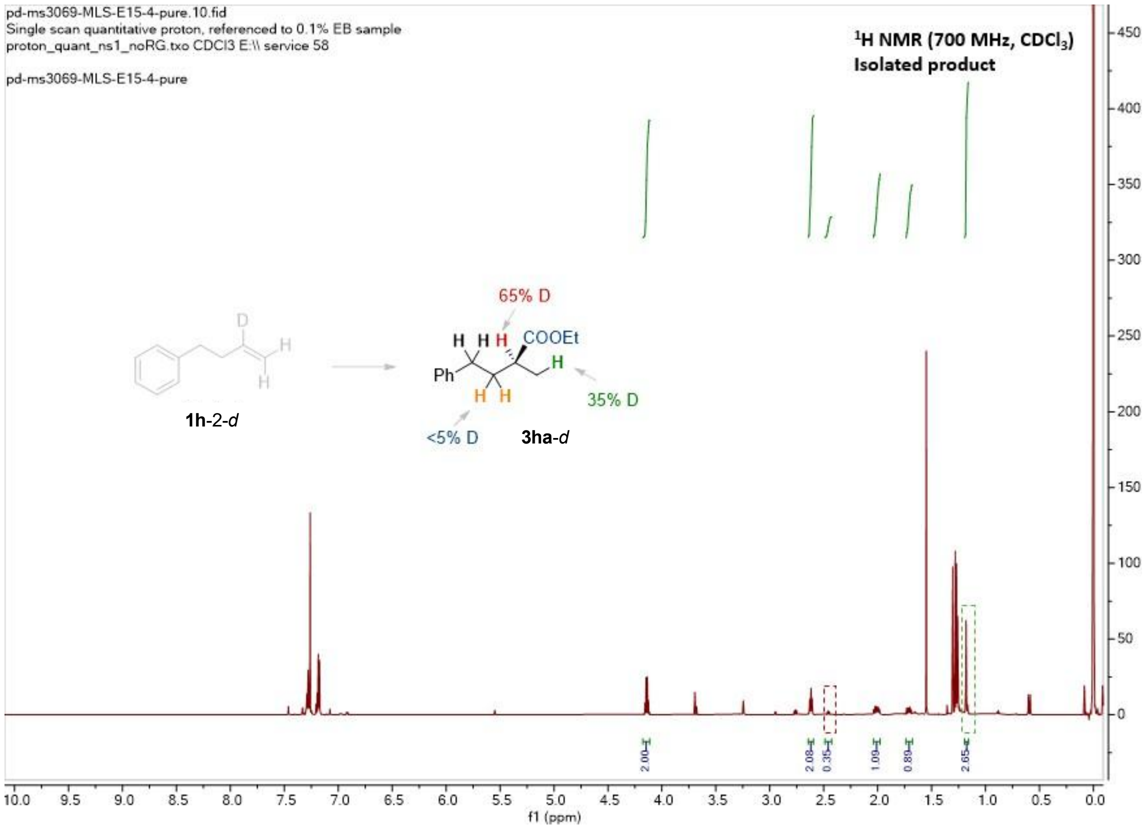

Fig. S7. *Quantitative ^{1}H NMR spectrum of isolated ester* ***3ha****-d from the palladium-catalysed carbonylation of* ***1h****-2-d and ethanol* ***2a****.*

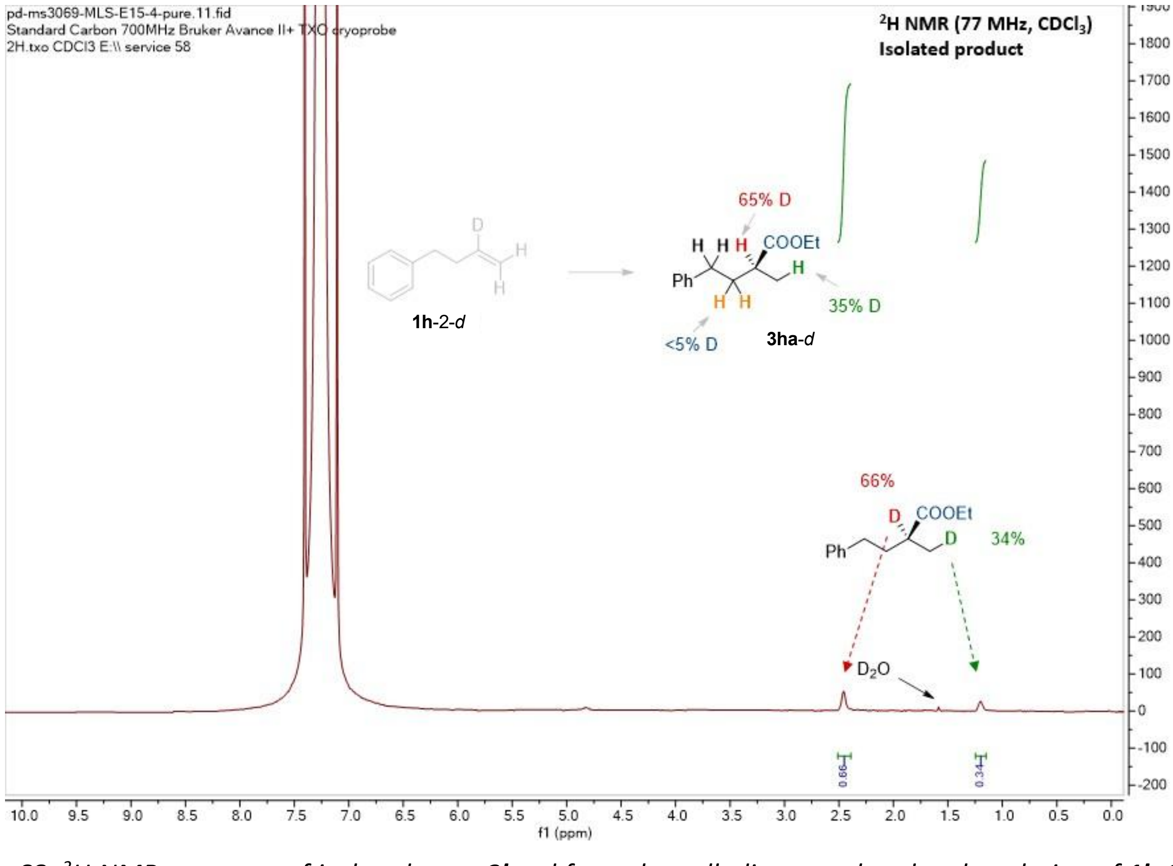

Fig. S8. *^{2}H NMR spectrum of isolated ester* ***3ha****-d from the palladium-catalysed carbonylation of* ***1h****-2-d and ethanol* ***2a****.*

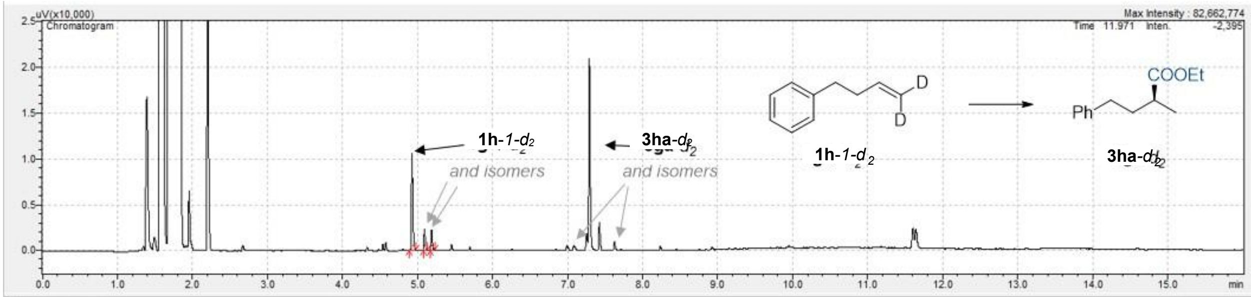

Results - Peak Table

Peak Table | Compound | Group | Calibration Curve

Peak#	Ret.Time	Area	Area%
1	4.927	13510.9	70.5363
2	5.092	2758.0	14.3986
3	5.188	2885.7	15.0651

Fig. S9. GC-FID chromatogram of the reaction mixture from the palladium-catalysed carbonylation of **1h**-1-d_2 and ethanol **2a**.

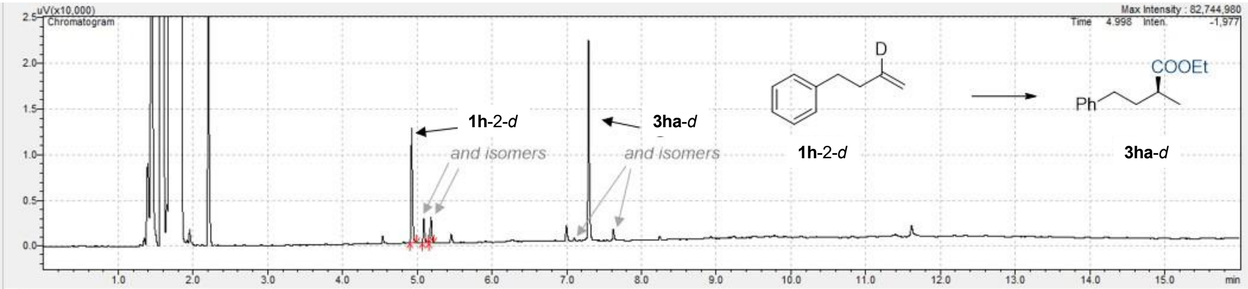

Results - Peak Table

Peak Table | Compound | Group | Calibration Curve

Peak#	Ret.Time	Area	Area%
1	4.925	16069.8	69.5511
2	5.087	3427.7	14.8350
3	5.184	3607.6	15.6139

Fig. S10. GC-FID chromatogram of the reaction mixture from the palladium-catalysed carbonylation of **1h**-2-d and ethanol **2a**.

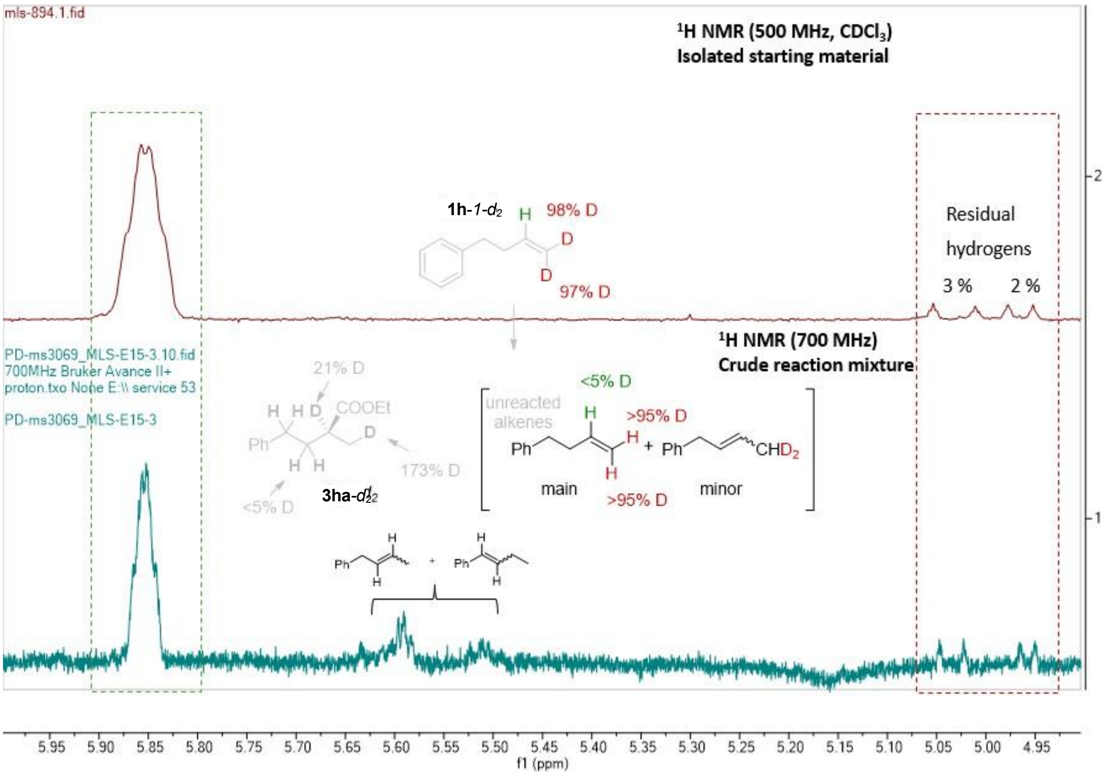

Fig. S11. *Comparison of the R-CH=CH$_2$ areas of the ^{1}H NMR spectrum of the isolated alkene* **1h***-1-d$_2$ and the ^{1}H NMR spectrum of the reaction mixture from the palladium-catalysed carbonylation of* **1h***-1-d$_2$ and ethanol* **2a***.*

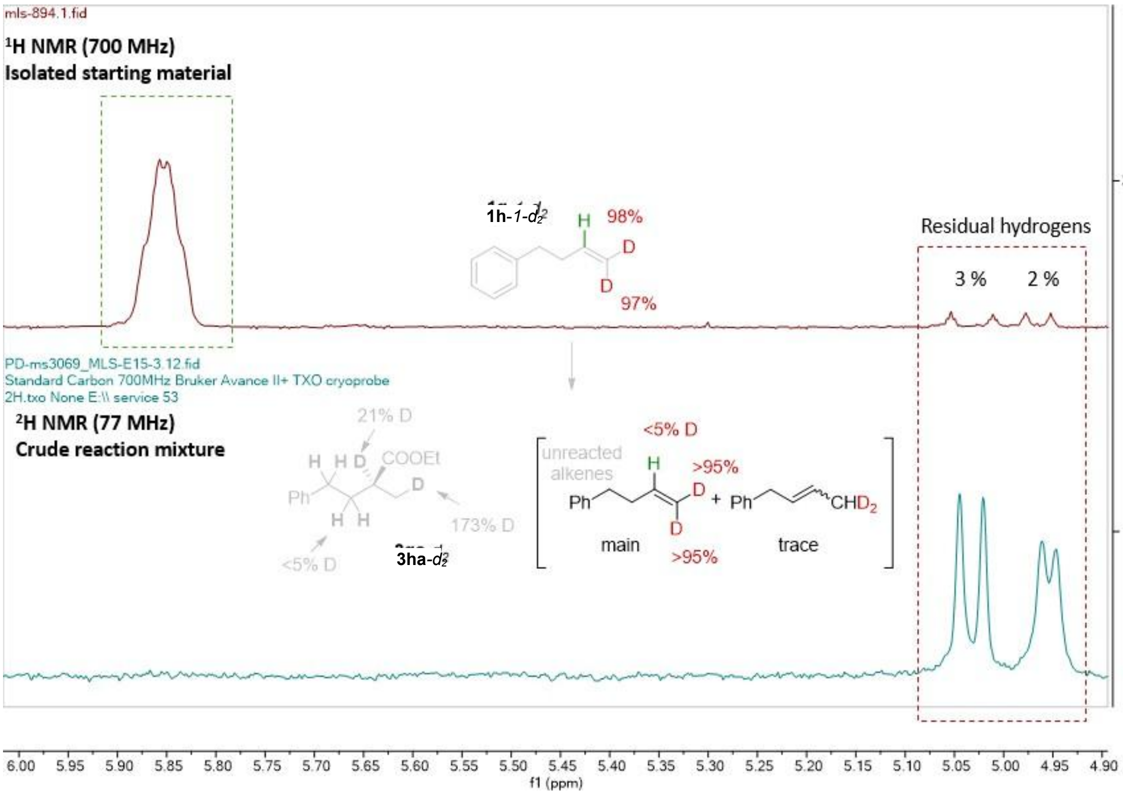

Fig. S12. *Comparison of the R-CH=CH$_2$ areas of the ^{1}H NMR spectrum of the isolated alkene* **1h***-1-d$_2$ and the ^{2}H NMR spectrum of the reaction mixture from the palladium-catalysed carbonylation of* **1h***-1-d$_2$ and ethanol* **2a***.*

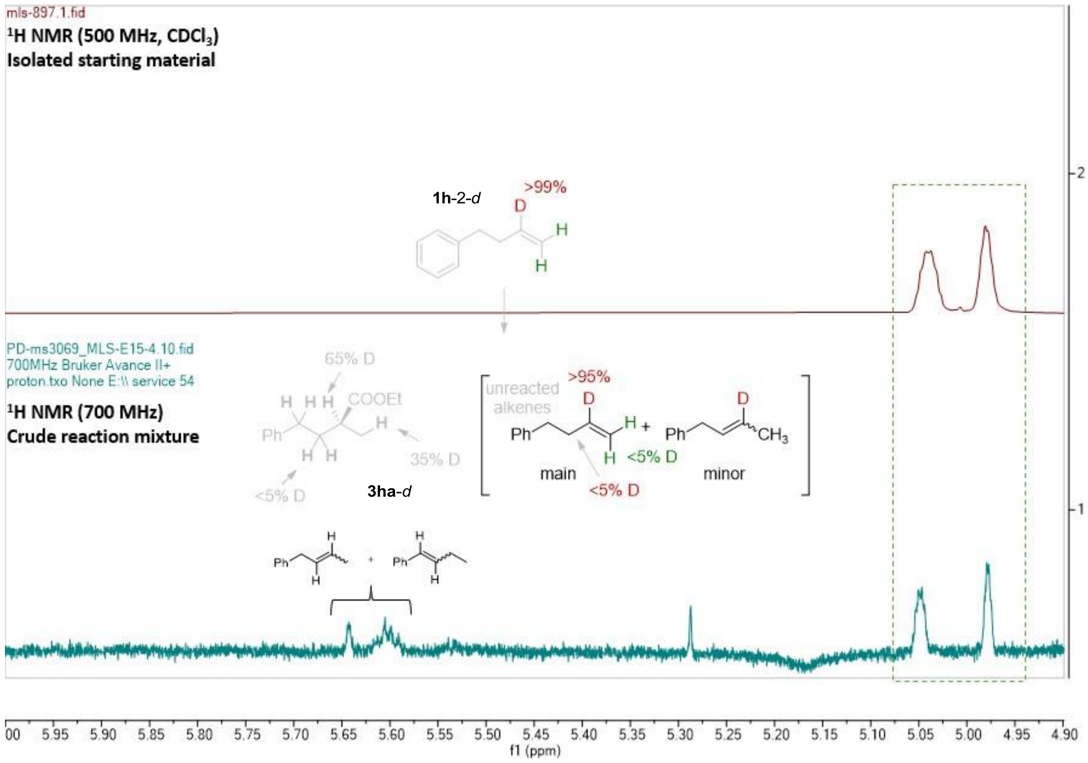

Fig. S13. *Comparison of the R-CH=CH$_2$ areas of the ^{1}H NMR spectrum of the isolated alkene **1h**-2-d and the ^{1}H NMR spectrum of the reaction mixture from the palladium-catalysed carbonylation of **1h**-2-d and ethanol **2a**.*

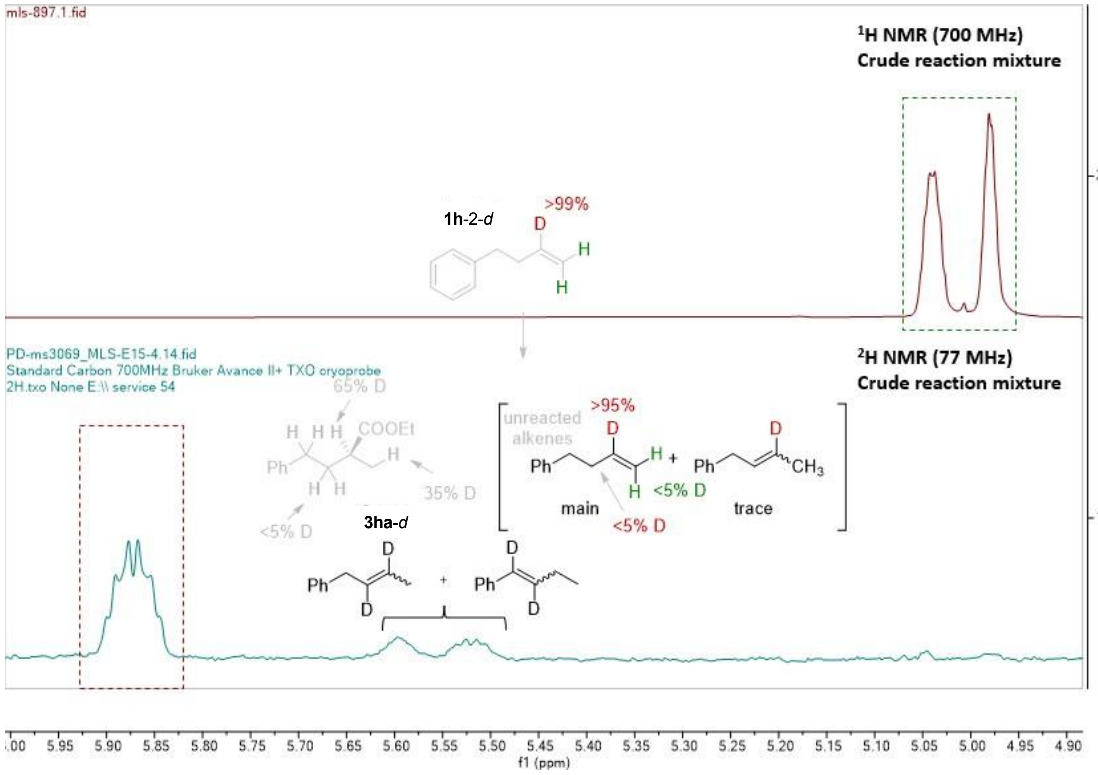

Fig. S14. *Comparison of the R-CH=CH$_2$ areas of the ^{1}H NMR spectrum of the isolated alkene **1h**-2-d and the ^{2}H NMR spectrum of the reaction mixture from the palladium-catalysed carbonylation of **1h**-2-d and ethanol **2a**.*

9. Copies of NMR spectra

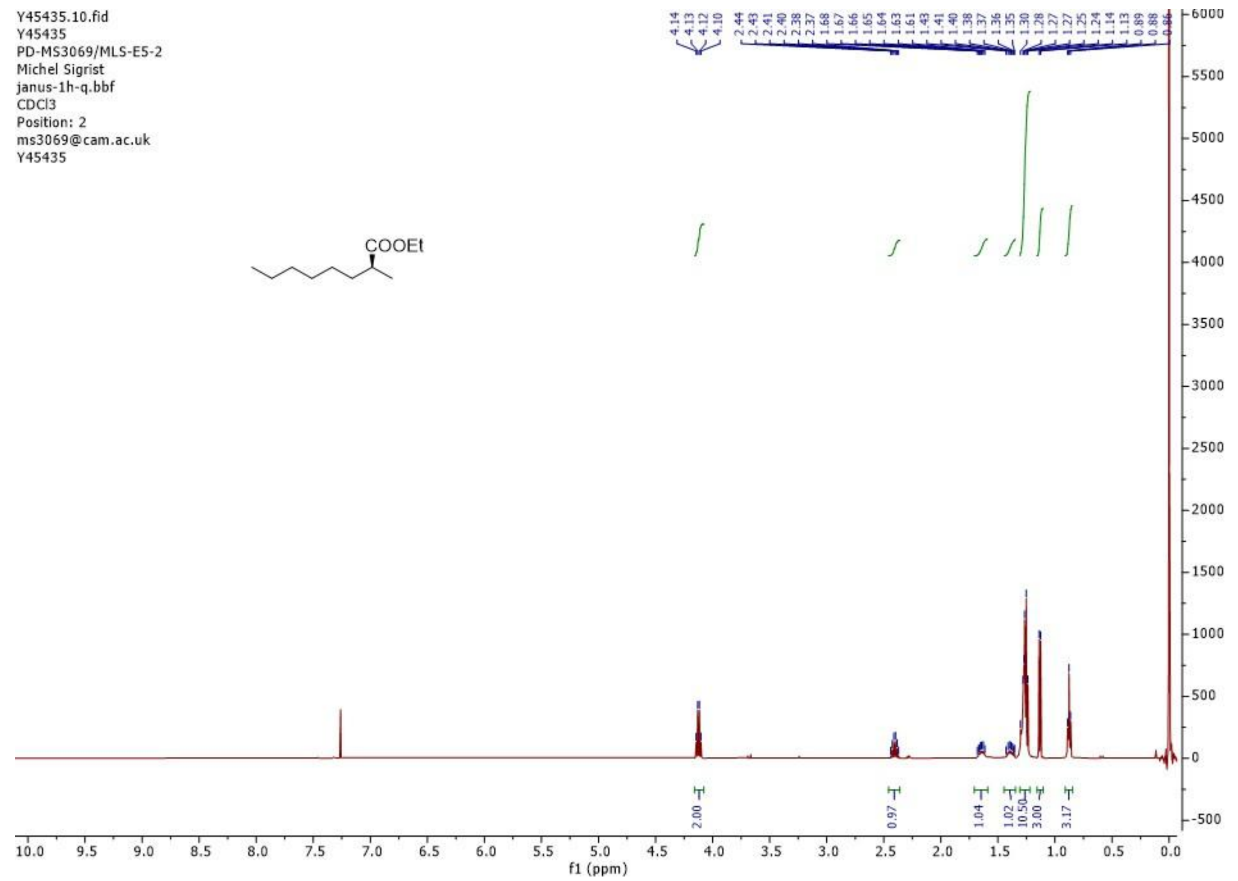

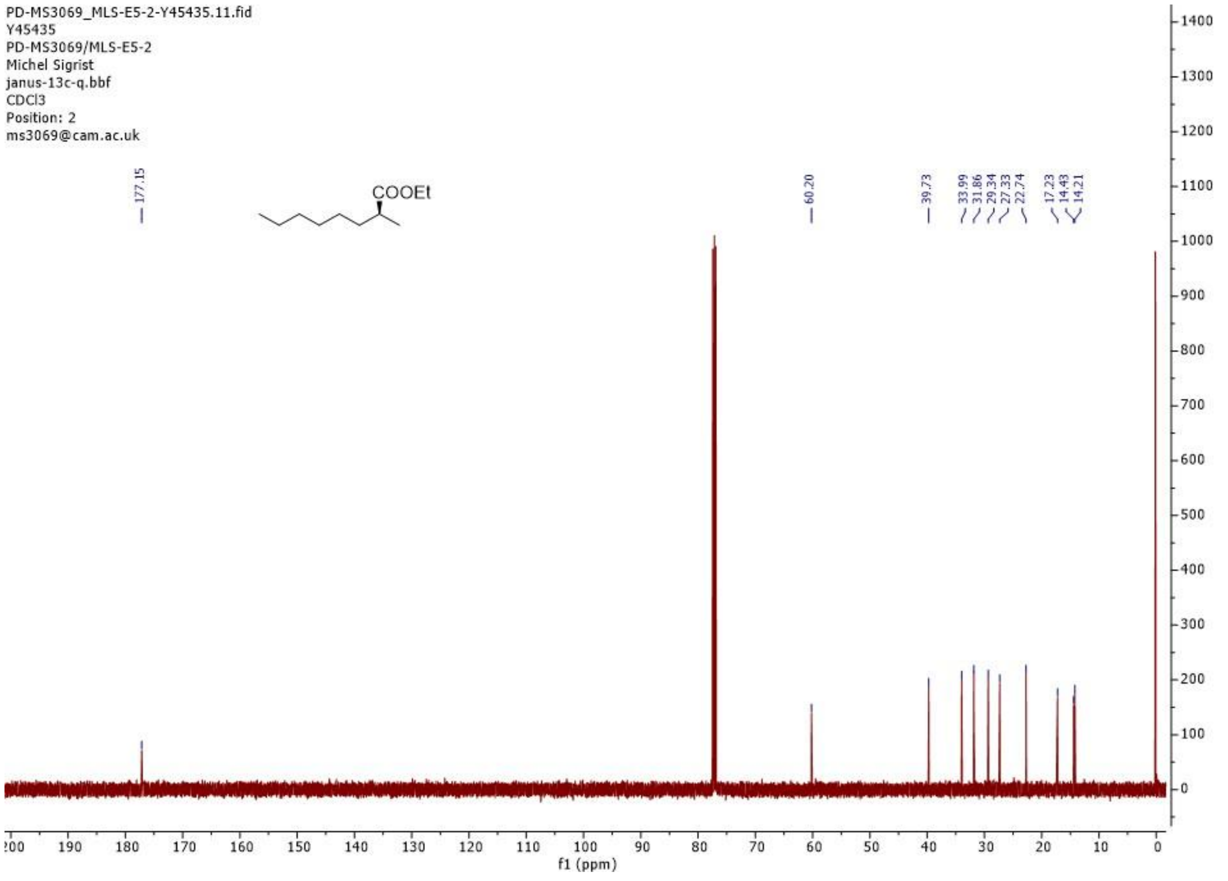

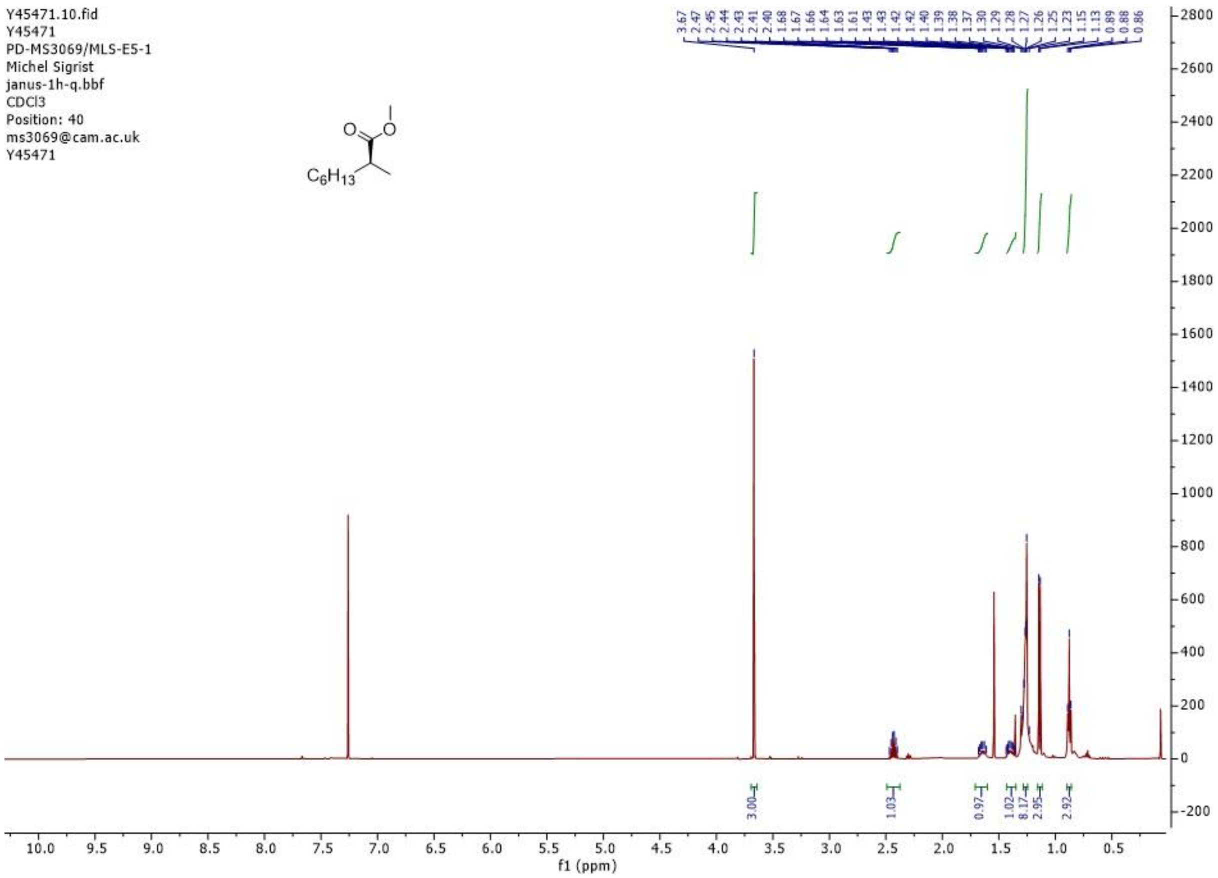
Y45471.10.fid
Y45471
PD-MS3069/MLS-E5-1
Michel Sigrist
janus-1h-q.bbf
CDCl3
Position: 40
ms3069@cam.ac.uk
Y45471
f1 (ppm)

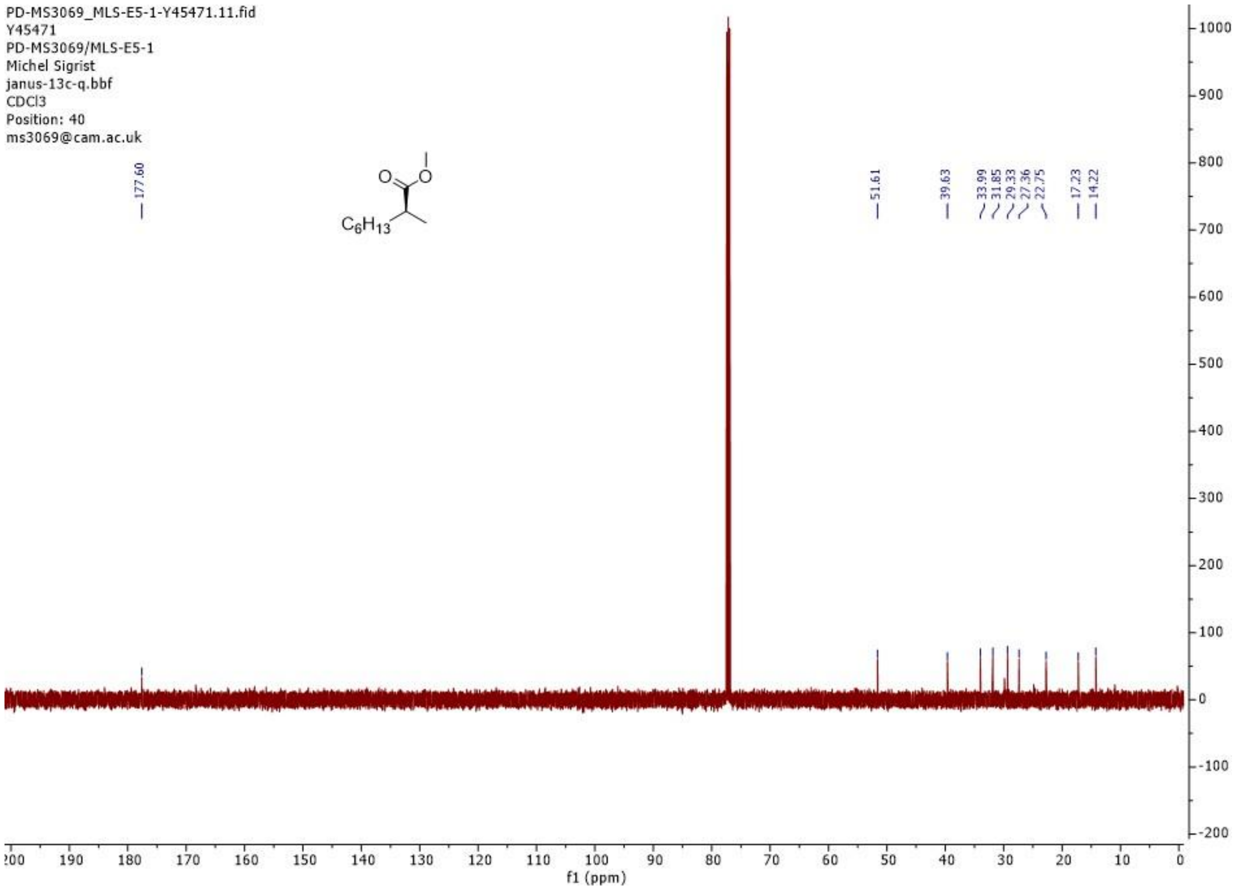
PD-MS3069_MLS-E5-1-Y45471.11.fid
Y45471
PD-MS3069/MLS-E5-1
Michel Sigrist
janus-13c-q.bbf
CDCl3
Position: 40
ms3069@cam.ac.uk
177.60
51.61
39.63
33.99
31.85
29.33
27.36
22.75
17.23
14.22
f1 (ppm)

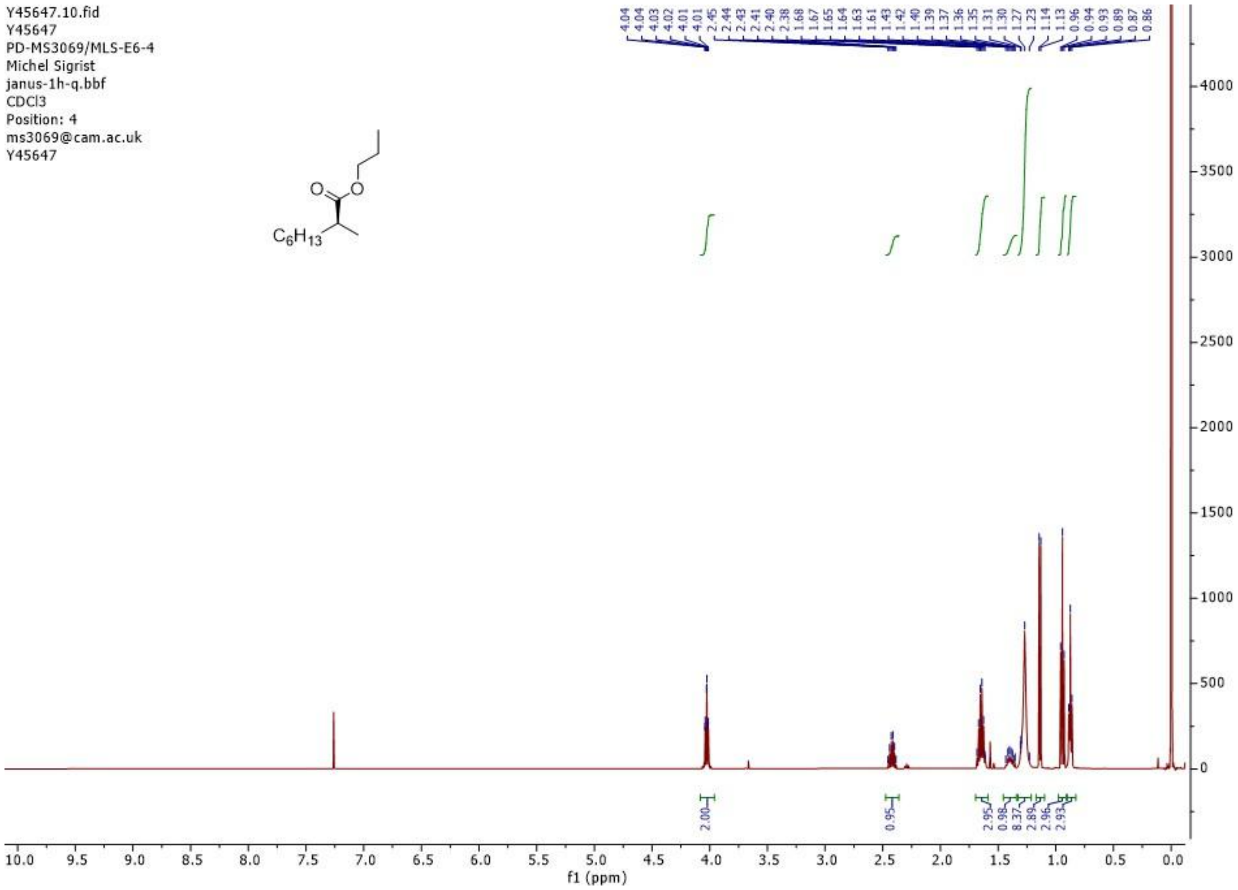
Y45647.10.fid
Y45647
PD-MS3069/MLS-E6-4
Michel Sigrist
janus-1h-q.bbf
CDCl3
Position: 4
ms3069@cam.ac.uk
Y45647
4.04 4.04 4.03 4.02 4.01 4.01 2.45 2.44 2.43 2.41 2.40 2.38 1.68 1.67 1.65 1.64 1.63 1.61 1.43 1.42 1.40 1.39 1.37 1.36 1.35 1.31 1.30 1.27 1.23 1.14 1.13 0.96 0.94 0.93 0.89 0.87 0.86
2.00 0.95 2.95 0.98 8.37 2.89 2.96 2.93
f1 (ppm)

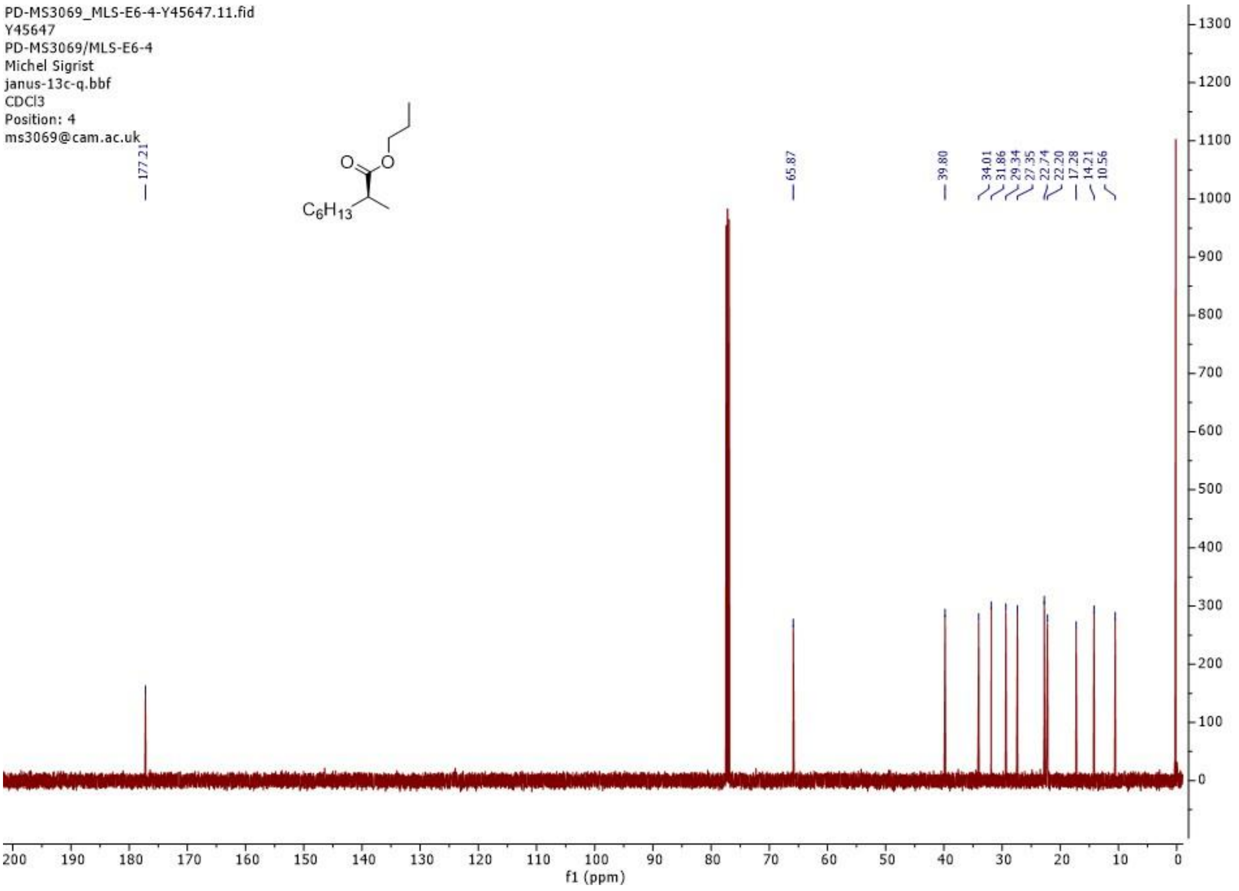
PD-MS3069_MLS-E6-4-Y45647.11.fid
Y45647
PD-MS3069/MLS-E6-4
Michel Sigrist
janus-13c-q.bbf
CDCl3
Position: 4
ms3069@cam.ac.uk
177.21
65.87
39.80
34.01 31.86 29.34 27.35 22.74 22.20 17.28 14.21 10.56
f1 (ppm)

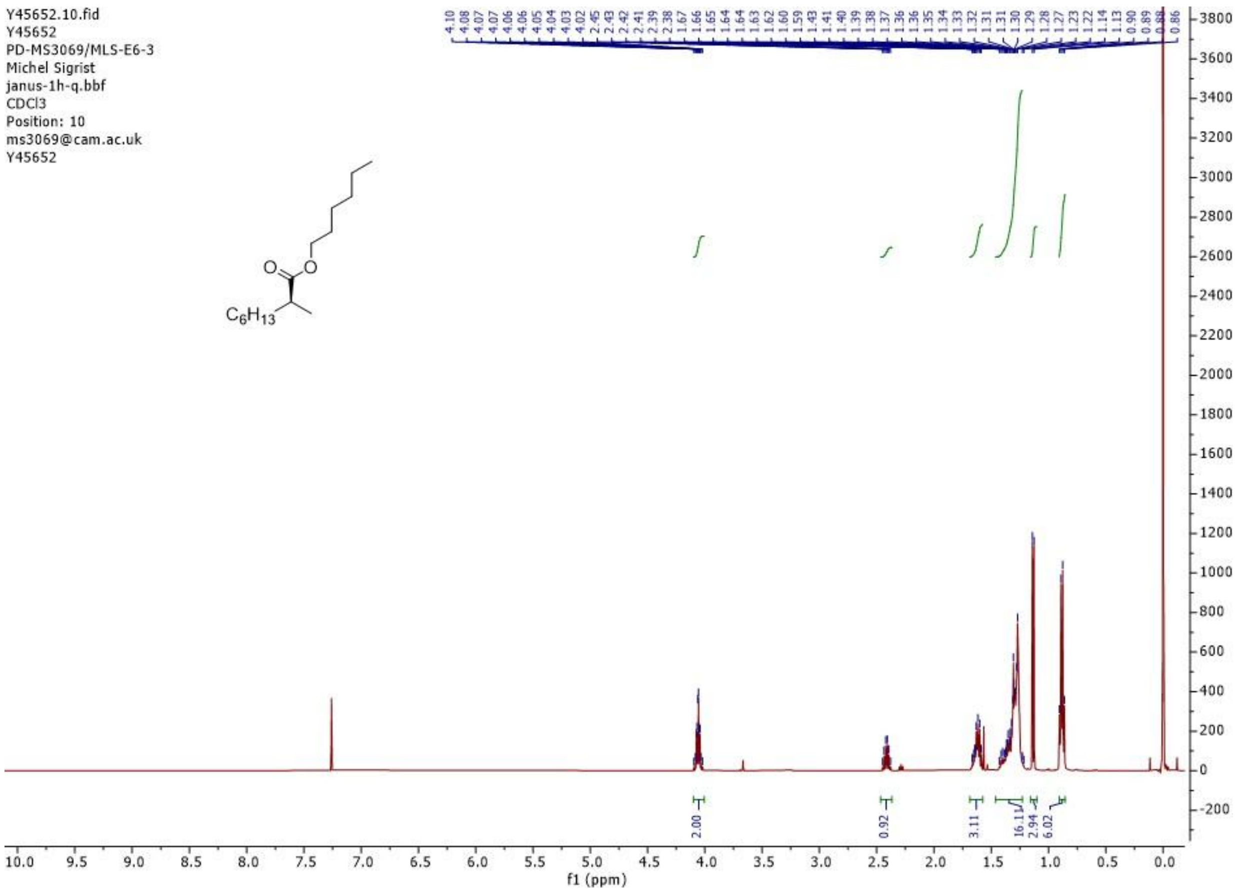
Y45652.10.fid
Y45652
PD-MS3069/MLS-E6-3
Michel Sigrist
janus-1h-q.bbf
CDCl3
Position: 10
ms3069@cam.ac.uk
Y45652
4.10 4.08 4.07 4.06 4.06 4.05 4.04 4.03 4.02 2.45 2.43 2.42 2.41 2.39 2.38 1.67 1.66 1.65 1.64 1.63 1.62 1.60 1.59 1.43 1.41 1.40 1.39 1.38 1.37 1.36 1.36 1.35 1.34 1.33 1.32 1.31 1.31 1.30 1.29 1.28 1.27 1.23 1.22 1.14 1.13 0.90 0.89 0.88 0.86
O
O
C6H13
2.00
0.92
3.11
16.11
2.94
6.02
f1 (ppm)

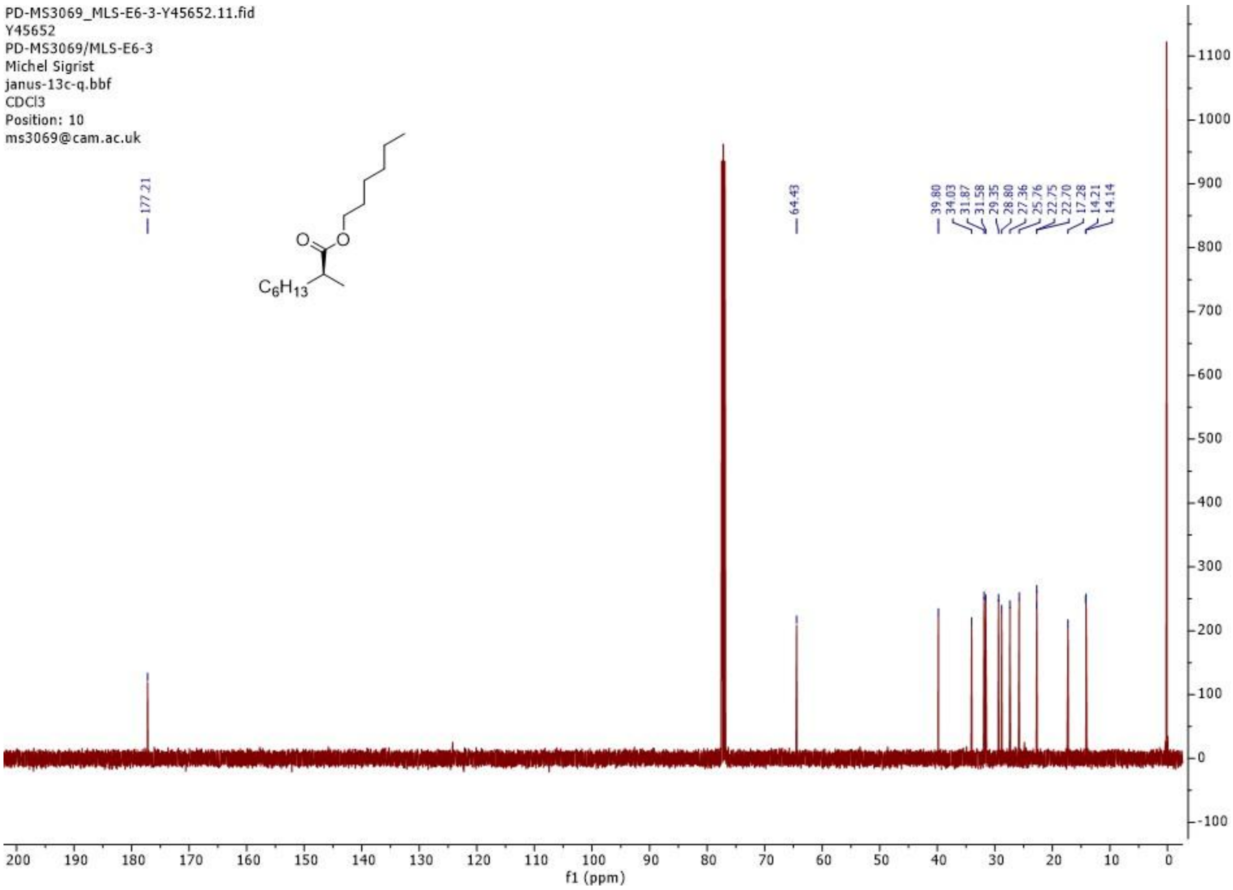
PD-MS3069_MLS-E6-3-Y45652.11.fid
Y45652
PD-MS3069/MLS-E6-3
Michel Sigrist
janus-13c-q.bbf
CDCl3
Position: 10
ms3069@cam.ac.uk
177.21
64.43
39.80 34.03 31.87 31.58 29.35 28.80 27.36 25.76 22.75 22.70 17.28 14.21 14.14
O
O
C6H13
f1 (ppm)

Y45610.10.fid
Y45610
PD-MS3069/MLS-E6-1
Michel Sigrist
janus-1h-q.bbf
CDCl3
Position: 25
ms3069@cam.ac.uk
Y45610

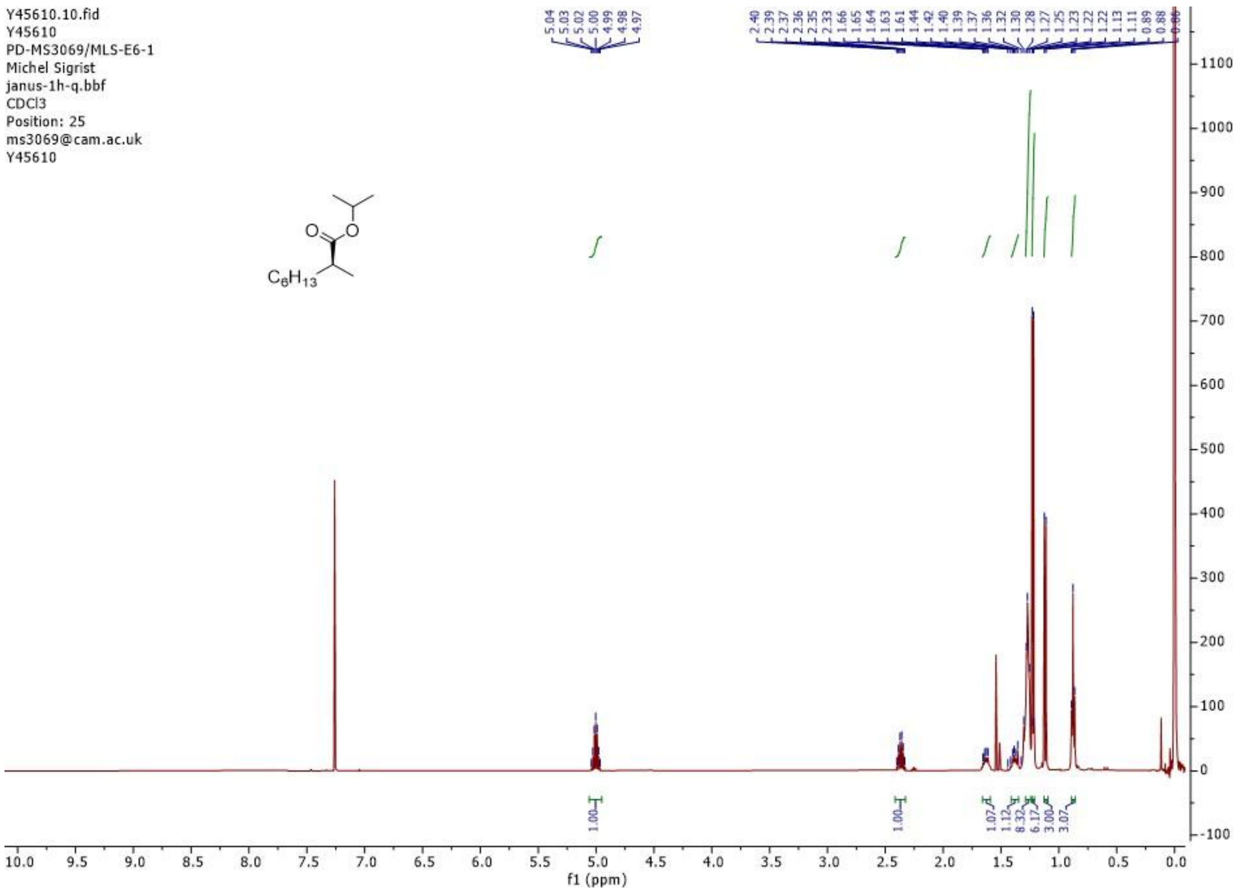

PD-MS3069_MLS-E6-1-Y45610.11.fid
Y45610
PD-MS3069/MLS-E6-1
Michel Sigrist
janus-13c-q.bbf
CDCl3
Position: 25
ms3069@cam.ac.uk

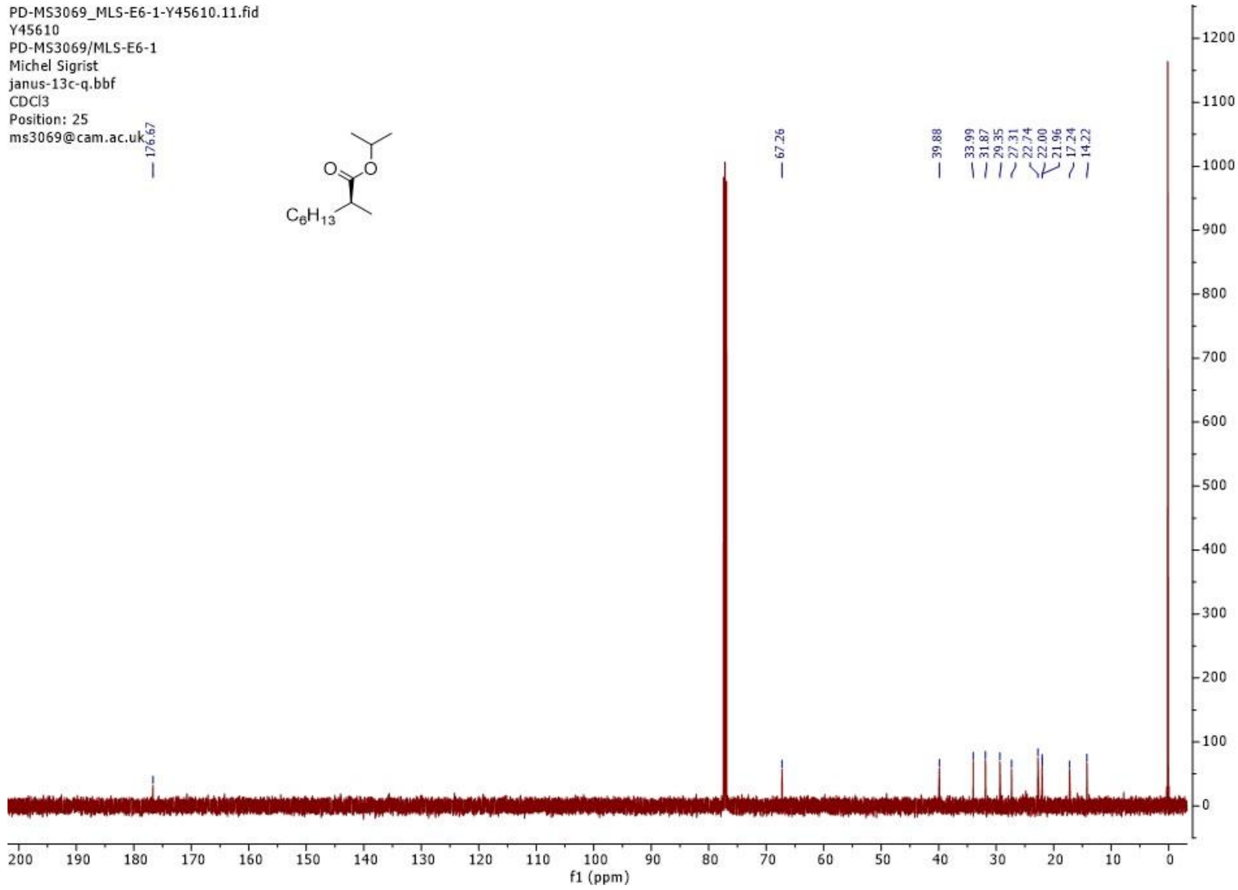

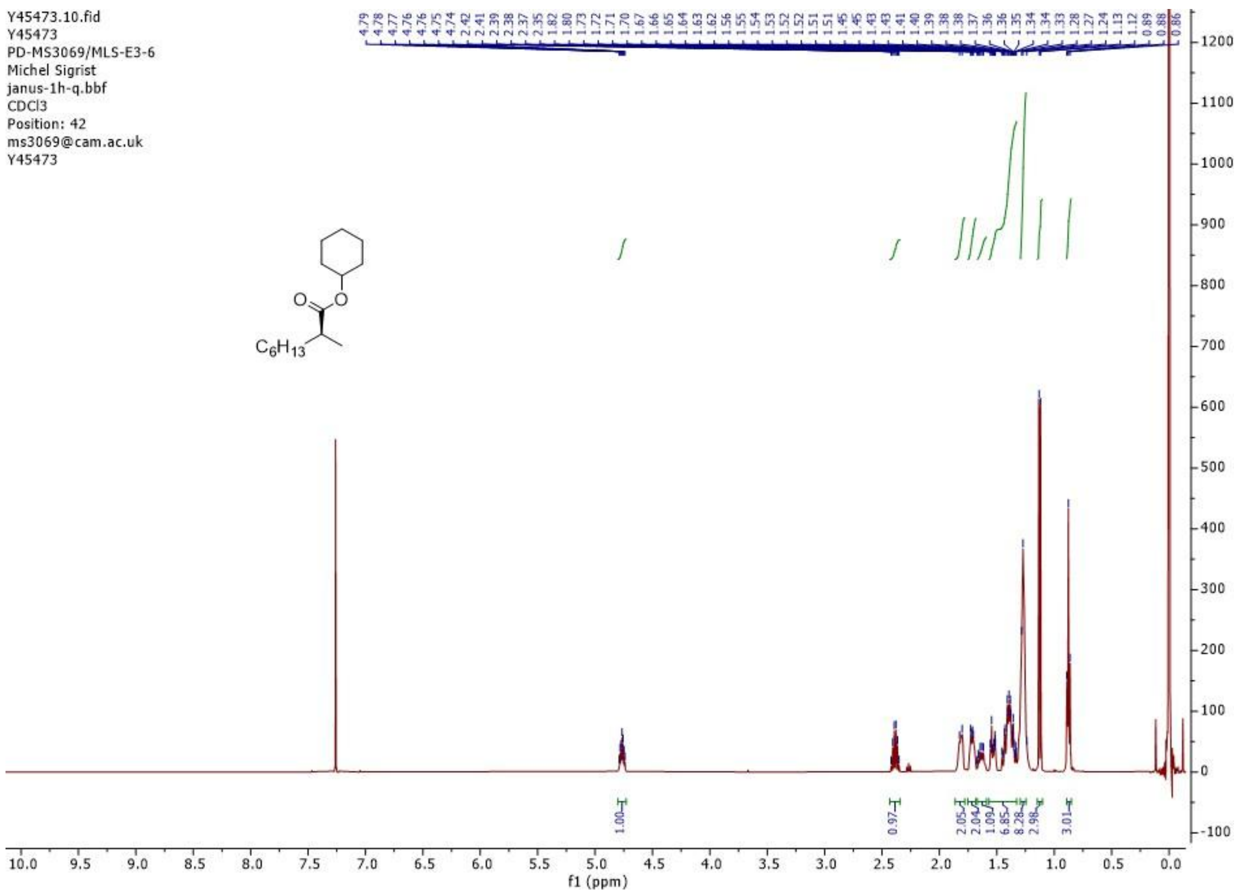
Y45473.10.fid
Y45473
PD-MS3069/MLS-E3-6
Michel Sigrist
janus-1h-q.bbf
CDCl3
Position: 42
ms3069@cam.ac.uk
Y45473
4.79 4.78 4.77 4.76 4.76 4.75 4.74 2.42 2.41 2.39 2.38 2.37 2.35 1.82 1.80 1.73 1.72 1.71 1.70 1.67 1.66 1.65 1.64 1.63 1.62 1.56 1.55 1.54 1.53 1.52 1.52 1.51 1.51 1.45 1.45 1.43 1.43 1.41 1.40 1.39 1.38 1.38 1.37 1.36 1.36 1.35 1.34 1.34 1.33 1.28 1.27 1.24 1.13 1.12 0.89 0.88 0.86
O
O
C_6H_{13}
1.00
0.97
2.05
2.04
1.09
6.85
8.28
2.98
3.01
f1 (ppm)

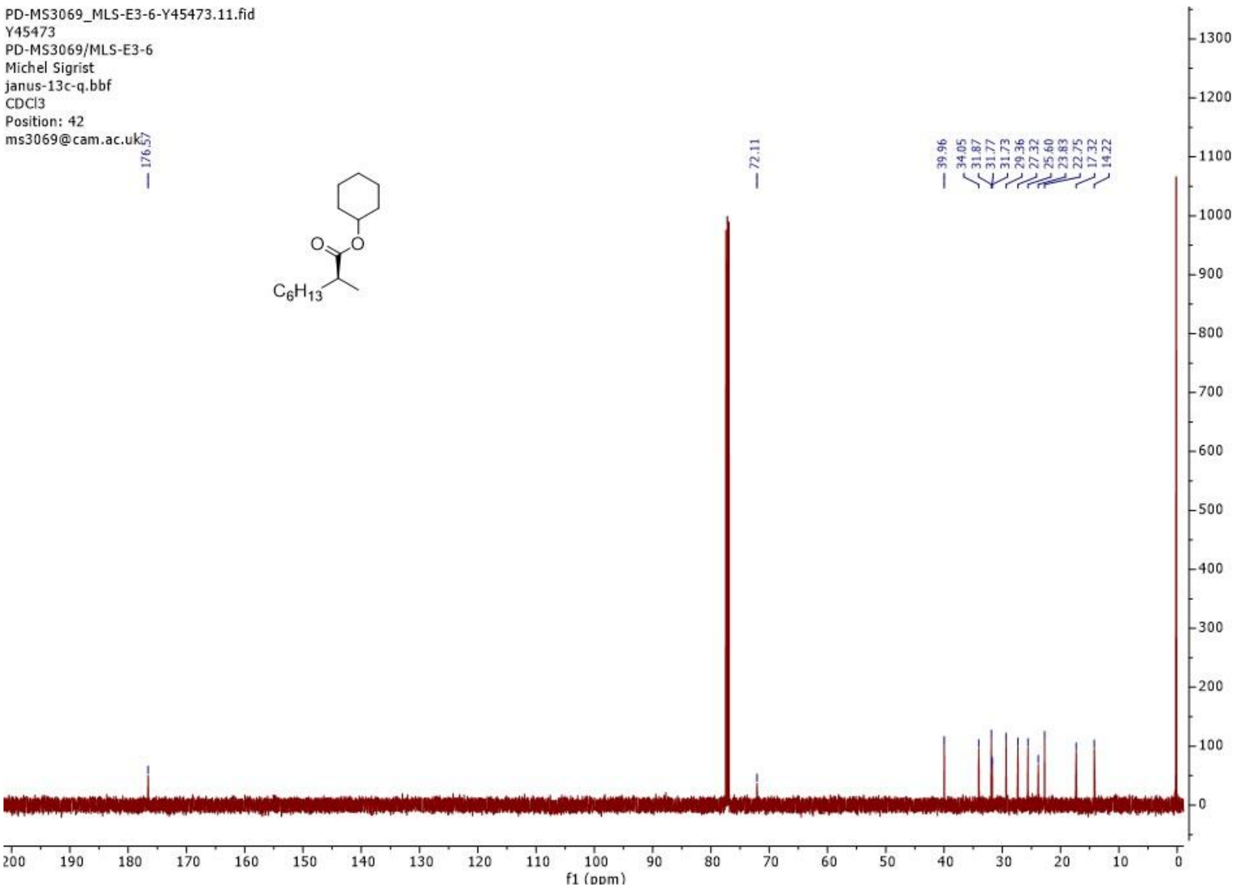
PD-MS3069_MLS-E3-6-Y45473.11.fid
Y45473
PD-MS3069/MLS-E3-6
Michel Sigrist
janus-13c-q.bbf
CDCl3
Position: 42
ms3069@cam.ac.uk
176.57
72.11
39.96 34.05 31.87 31.77 31.73 29.36 27.32 25.60 23.83 22.75 17.32 14.22
O
O
C_6H_{13}
f1 (ppm)

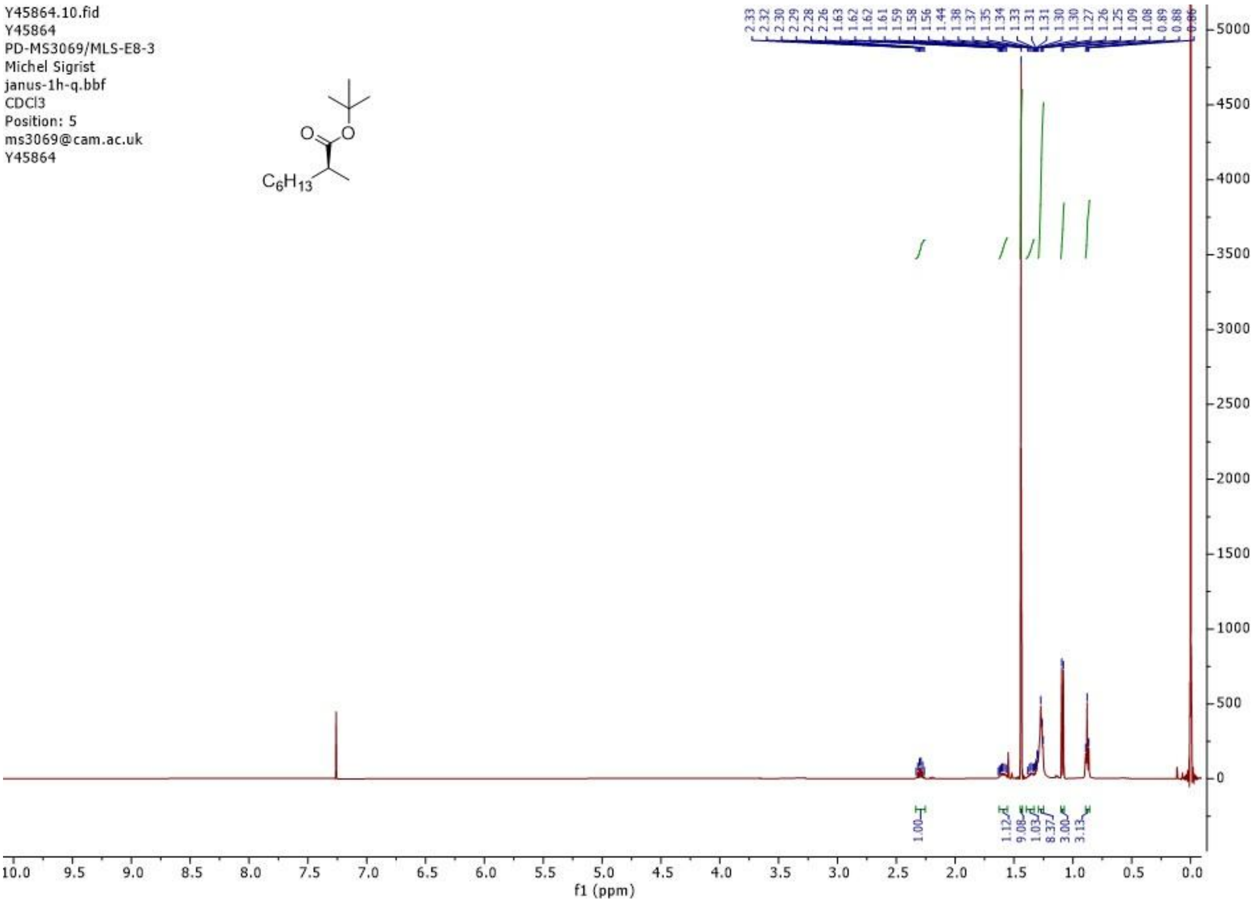
Y45864.10.fid
Y45864
PD-MS3069/MLS-E8-3
Michel Sigrist
janus-1h-q.bbf
CDCl3
Position: 5
ms3069@cam.ac.uk
Y45864
C6H13
2.33 2.32 2.30 2.29 2.28 2.26 1.63 1.62 1.62 1.61 1.59 1.58 1.56 1.44 1.38 1.37 1.35 1.34 1.33 1.31 1.31 1.30 1.30 1.27 1.26 1.25 1.09 1.08 0.89 0.88 0.86
1.00 1.12 9.08 1.03 8.37 3.00 3.13
f1 (ppm)

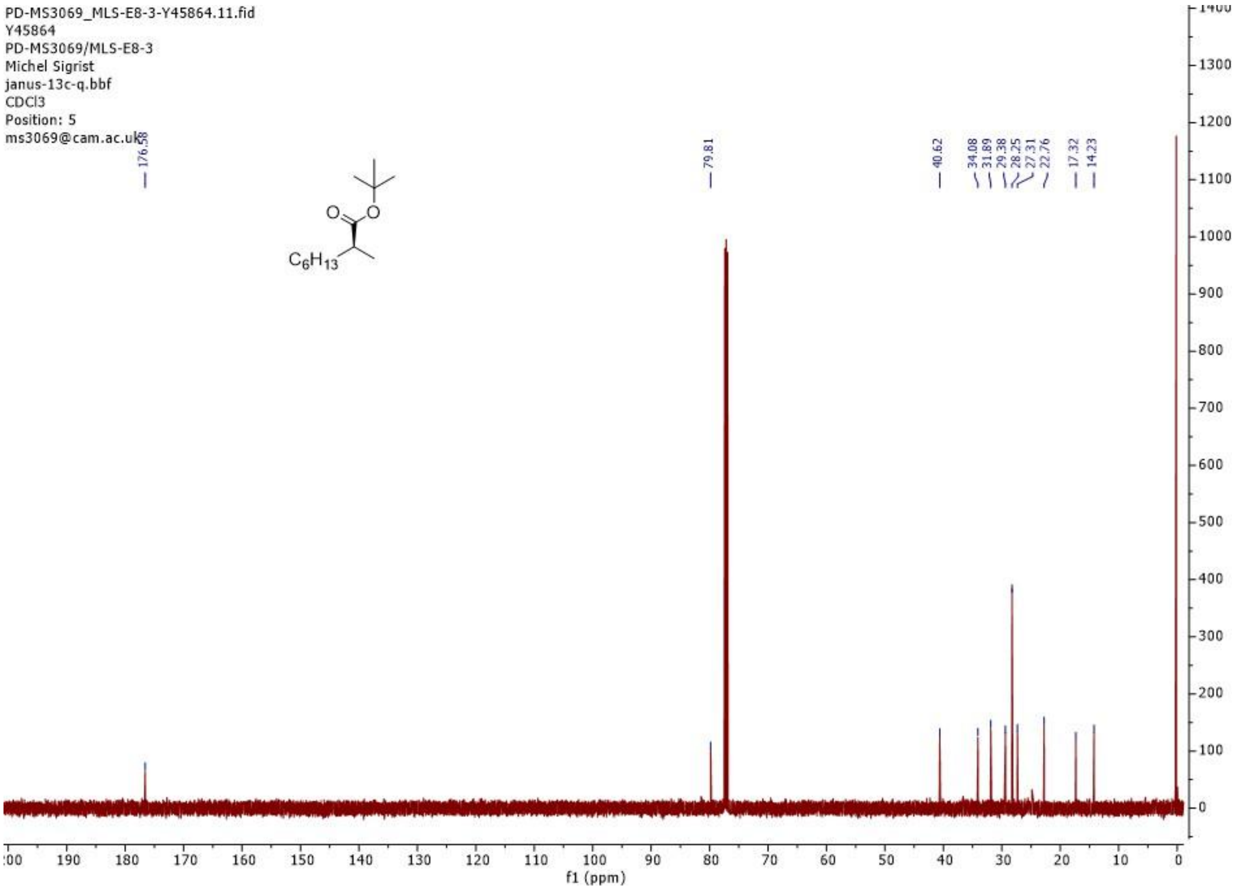
PD-MS3069_MLS-E8-3-Y45864.11.fid
Y45864
PD-MS3069/MLS-E8-3
Michel Sigrist
janus-13c-q.bbf
CDCl3
Position: 5
ms3069@cam.ac.uk
176.58
79.81
40.62 34.08 31.89 29.38 28.25 27.31 22.76 17.32 14.23
C6H13
f1 (ppm)

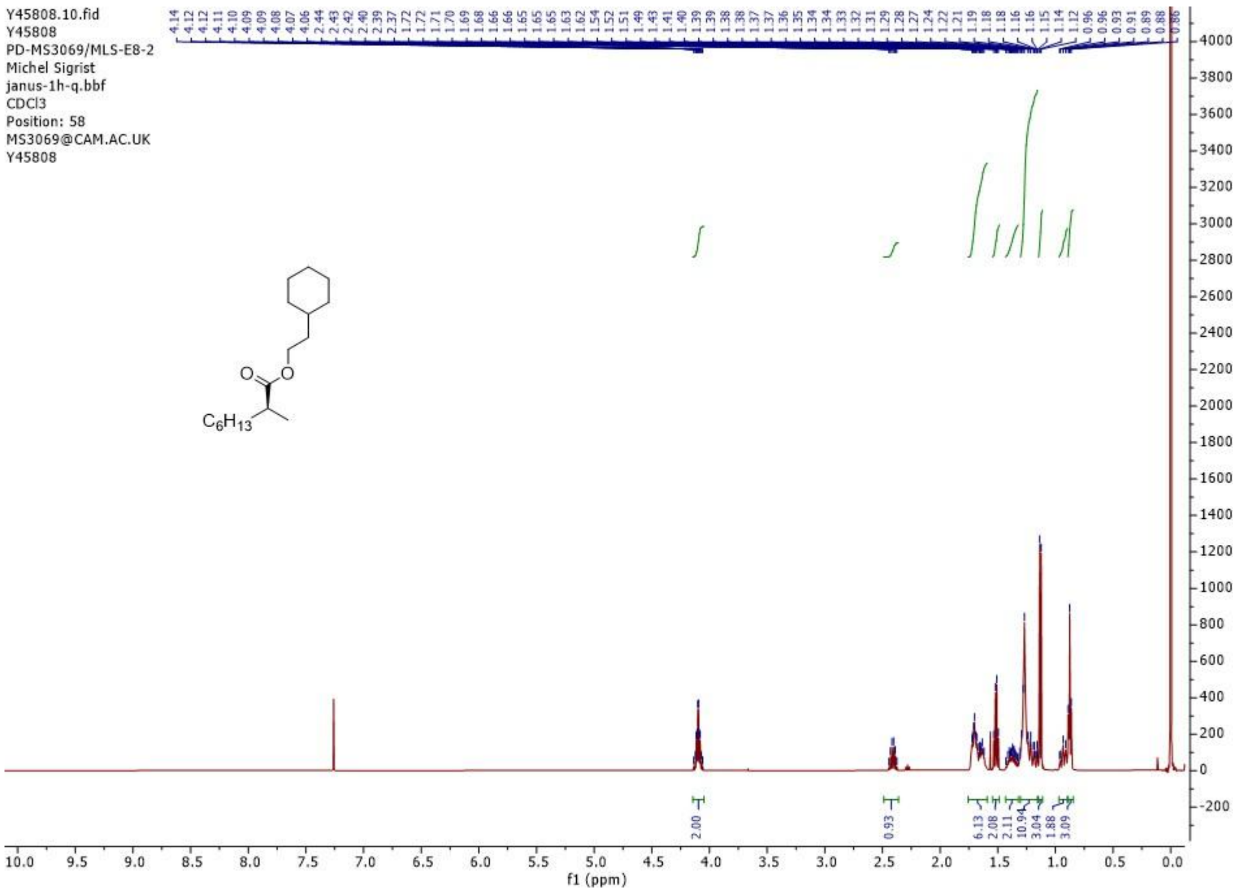
Y45808.10.fid
Y45808
PD-MS3069/MLS-E8-2
Michel Sigrist
janus-1h-q.bbf
CDCl3
Position: 58
MS3069@CAM.AC.UK
Y45808
4.14 4.12 4.12 4.11 4.10 4.09 4.09 4.08 4.07 4.06 2.44 2.43 2.42 2.40 2.39 2.37 1.72 1.72 1.71 1.70 1.69 1.68 1.66 1.66 1.65 1.65 1.63 1.62 1.54 1.52 1.51 1.49 1.43 1.41 1.40 1.39 1.39 1.38 1.38 1.37 1.37 1.36 1.35 1.34 1.34 1.33 1.32 1.31 1.29 1.28 1.27 1.24 1.22 1.21 1.19 1.18 1.18 1.16 1.16 1.15 1.14 1.12 0.96 0.96 0.93 0.91 0.89 0.88 0.86
C6H13
2.00
0.93
6.13
2.08
2.11
10.94
3.04
1.88
3.09
f1 (ppm)

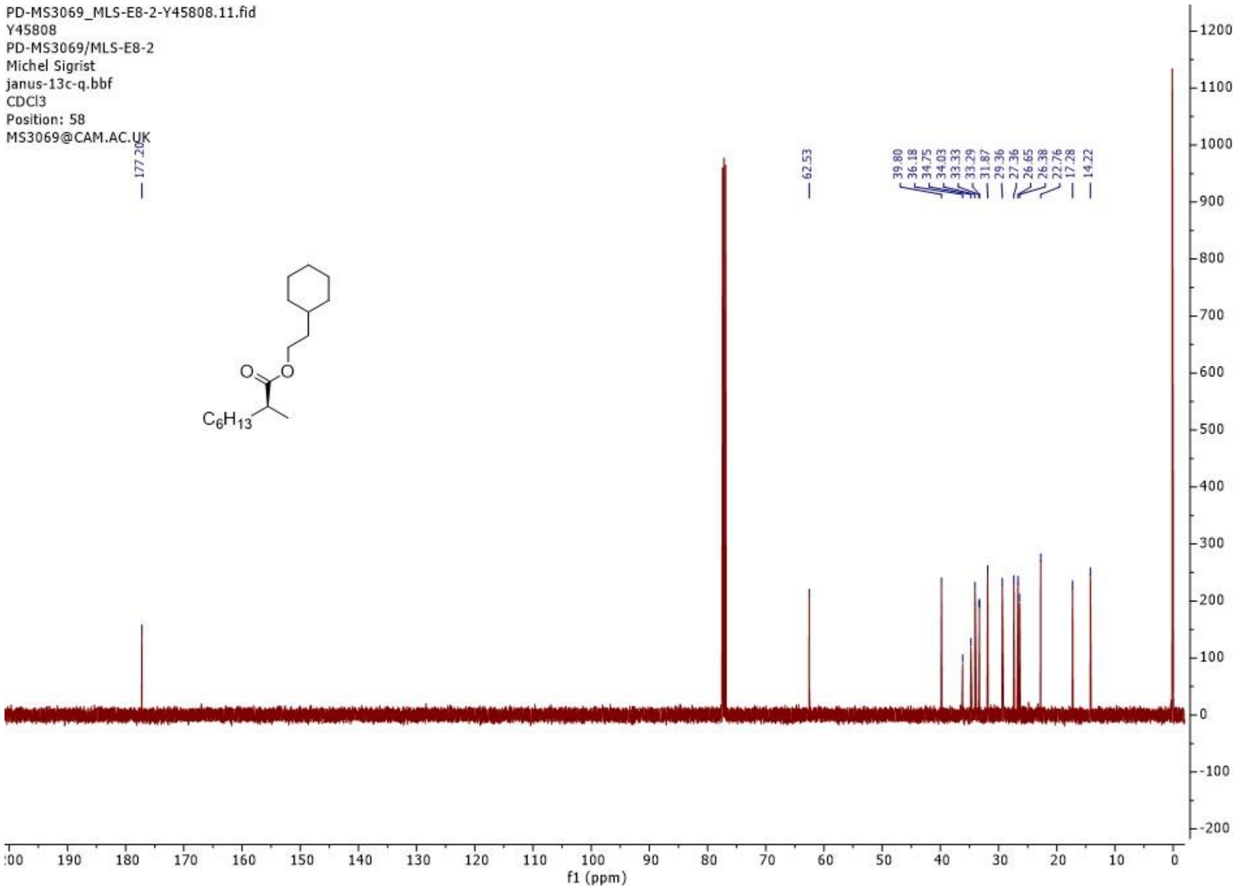
PD-MS3069_MLS-E8-2-Y45808.11.fid
Y45808
PD-MS3069/MLS-E8-2
Michel Sigrist
janus-13c-q.bbf
CDCl3
Position: 58
MS3069@CAM.AC.UK
177.20
62.53
39.80 36.18 34.75 34.03 33.33 33.29 31.87 29.36 27.36 26.65 26.38 22.76 17.28 14.22
C6H13
f1 (ppm)

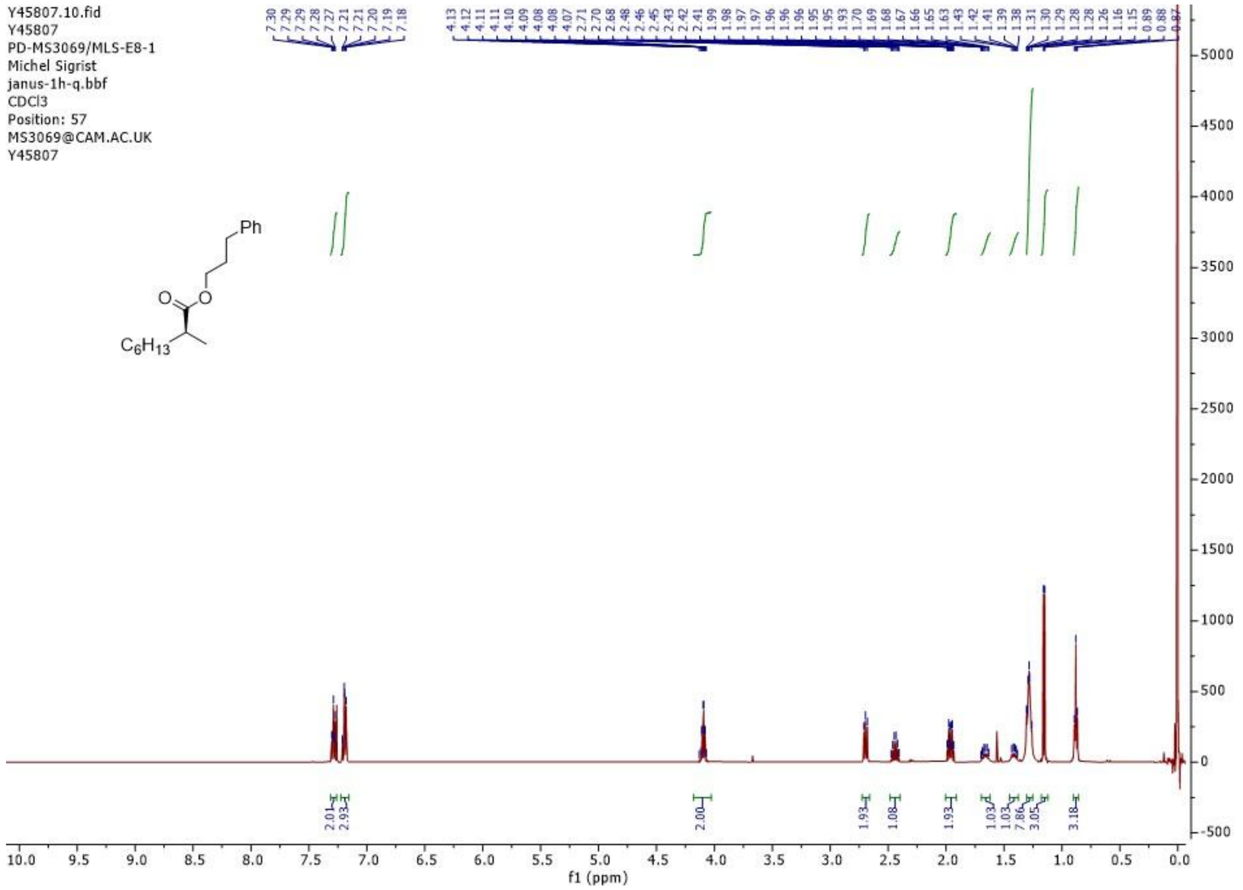
Y45807.10.fid
Y45807
PD-MS3069/MLS-E8-1
Michel Sigrist
janus-1h-q.bbf
CDCl3
Position: 57
MS3069@CAM.AC.UK
Y45807
Ph
O
O
C_6H_{13}
2.01
2.93
2.00
1.93
1.08
1.93
1.03
1.03
7.86
3.05
3.18
f1 (ppm)

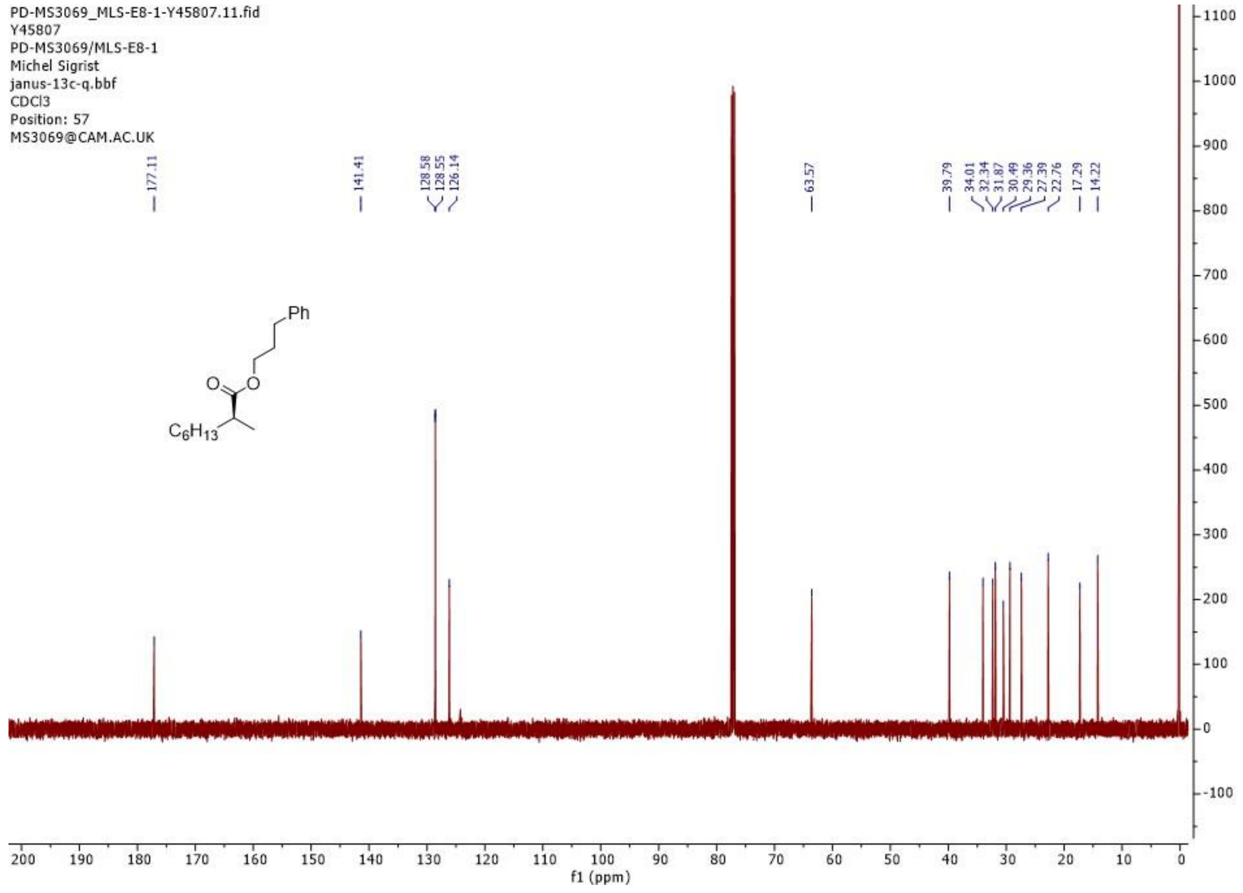
PD-MS3069_MLS-E8-1-Y45807.11.fid
Y45807
PD-MS3069/MLS-E8-1
Michel Sigrist
janus-13c-q.bbf
CDCl3
Position: 57
MS3069@CAM.AC.UK
177.11
141.41
128.58
128.55
126.14
63.57
39.79
34.01
32.34
31.87
30.49
29.36
27.39
22.76
17.29
14.22
Ph
O
O
C_6H_{13}
f1 (ppm)

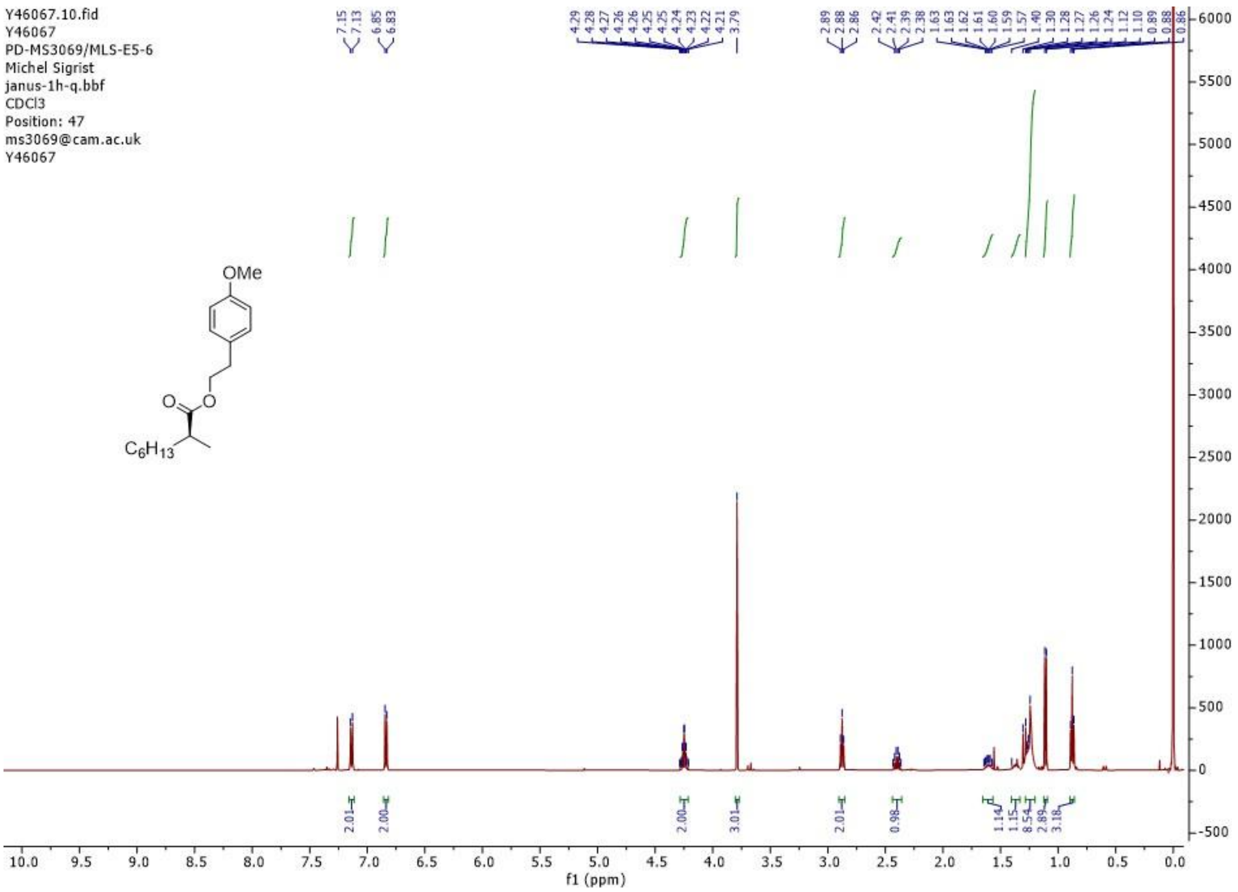
Y46067.10.fid
Y46067
PD-MS3069/MLS-E5-6
Michel Sigrist
janus-1h-q.bbf
CDCl3
Position: 47
ms3069@cam.ac.uk
Y46067
OMe
C6H13
f1 (ppm)

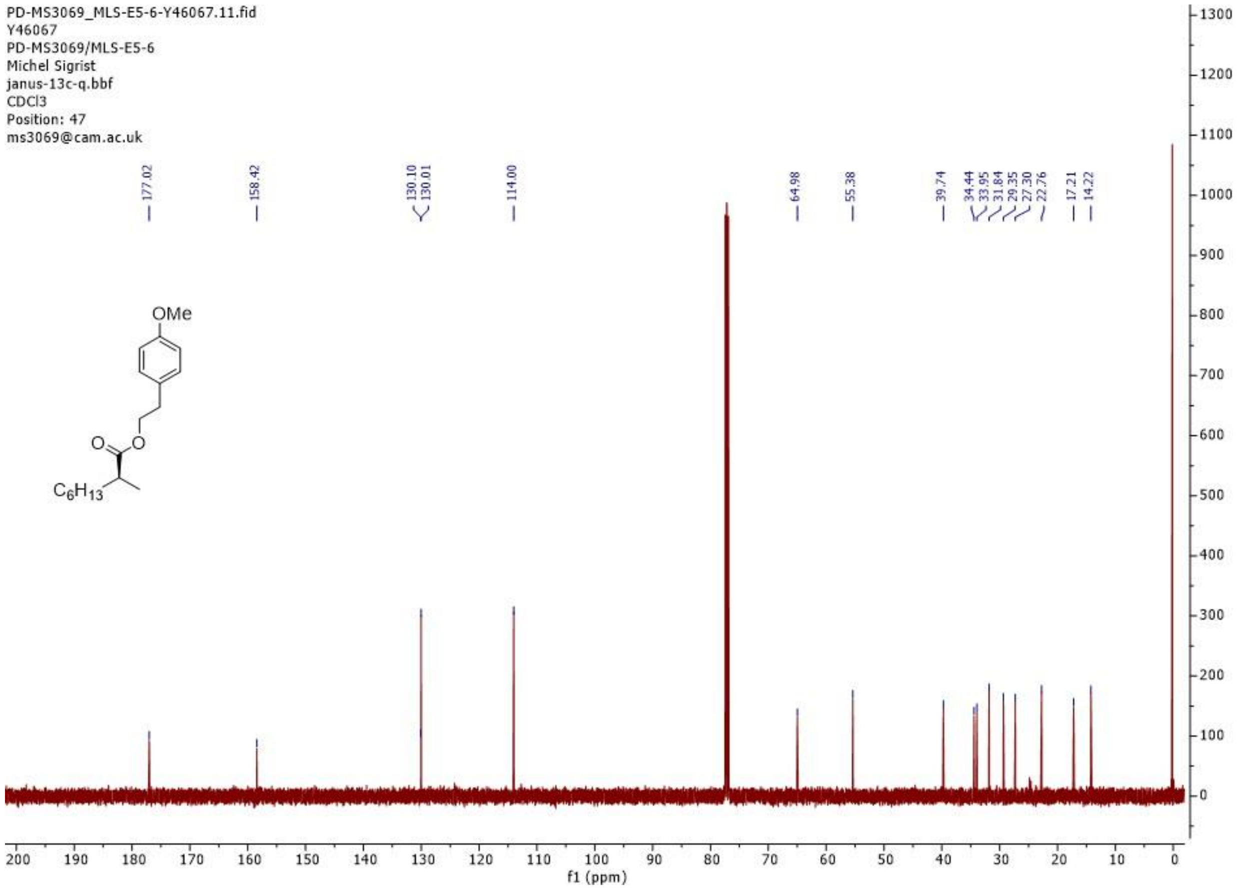
PD-MS3069_MLS-E5-6-Y46067.11.fid
Y46067
PD-MS3069/MLS-E5-6
Michel Sigrist
janus-13c-q.bbf
CDCl3
Position: 47
ms3069@cam.ac.uk
177.02
158.42
130.10
130.01
114.00
64.98
55.38
39.74
34.44
33.95
31.84
29.35
27.30
22.76
17.21
14.22
OMe
C6H13
f1 (ppm)

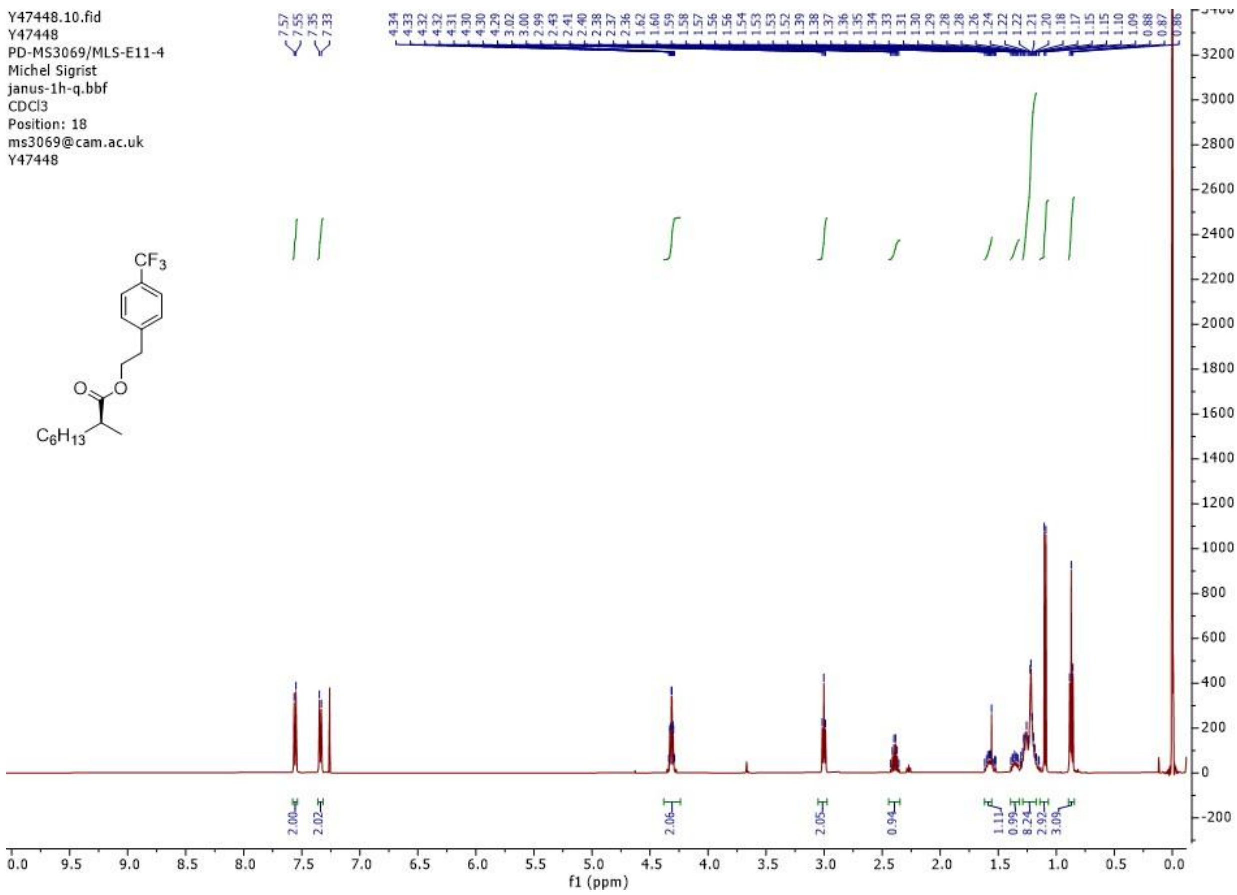
Y47448.10.fid
Y47448
PD-MS3069/MLS-E11-4
Michel Sigrist
janus-1h-q.bbf
CDCl3
Position: 18
ms3069@cam.ac.uk
Y47448
7.57
7.55
7.35
7.33
4.34
4.33
4.32
4.31
4.30
4.30
4.29
3.02
3.00
2.99
2.43
2.41
2.40
2.38
2.37
2.36
1.62
1.60
1.59
1.58
1.57
1.56
1.56
1.54
1.53
1.53
1.52
1.39
1.38
1.37
1.36
1.35
1.34
1.33
1.31
1.30
1.29
1.28
1.26
1.24
1.22
1.22
1.21
1.20
1.18
1.17
1.15
1.15
1.10
1.09
0.88
0.87
0.86
CF3
O
O
C6H13
2.00
2.02
2.06
2.05
0.94
1.11
0.99
8.24
2.92
3.09
f1 (ppm)

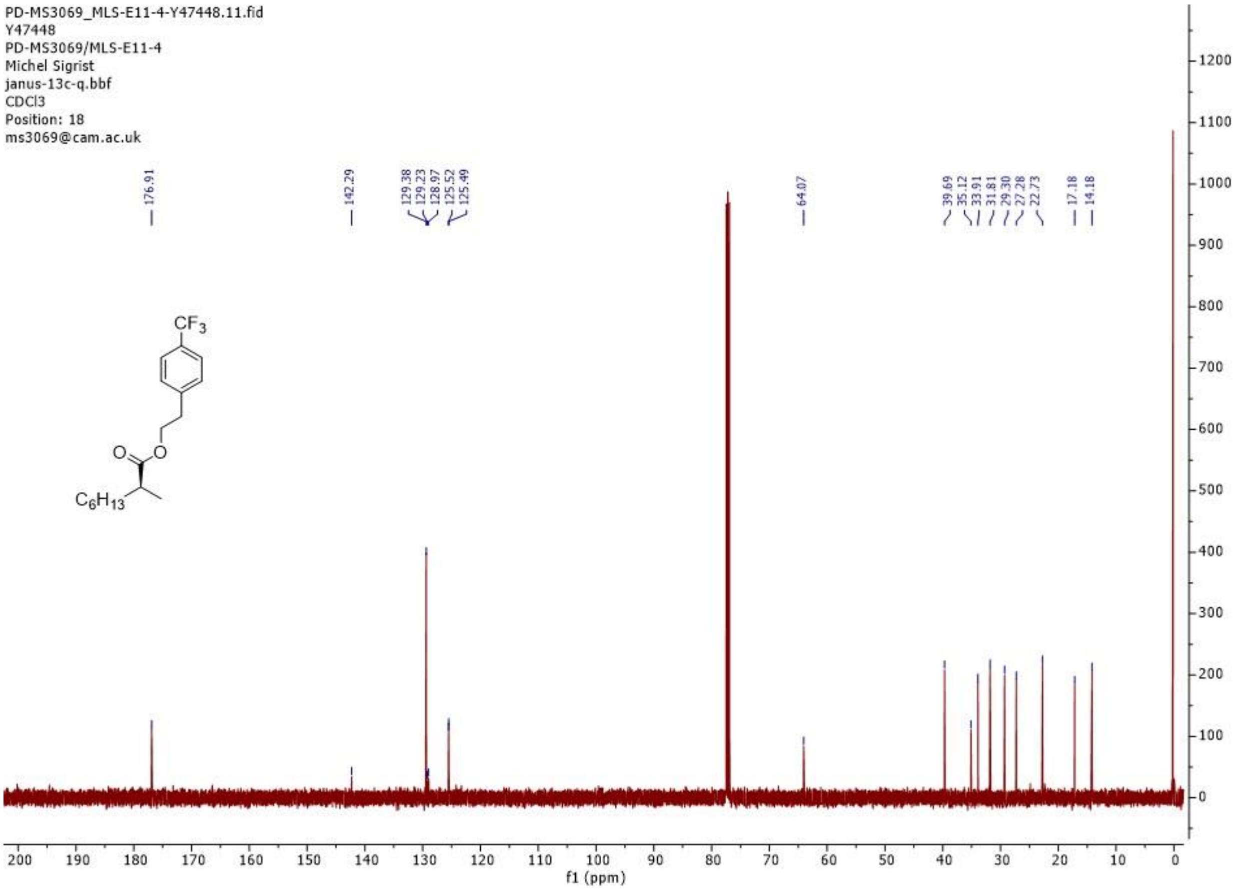
PD-MS3069_MLS-E11-4-Y47448.11.fid
Y47448
PD-MS3069/MLS-E11-4
Michel Sigrist
janus-13c-q.bbf
CDCl3
Position: 18
ms3069@cam.ac.uk
176.91
142.29
129.38
129.23
128.97
125.52
125.49
64.07
39.69
35.12
33.91
31.81
29.30
27.28
22.73
17.18
14.18
CF3
O
O
C6H13
f1 (ppm)

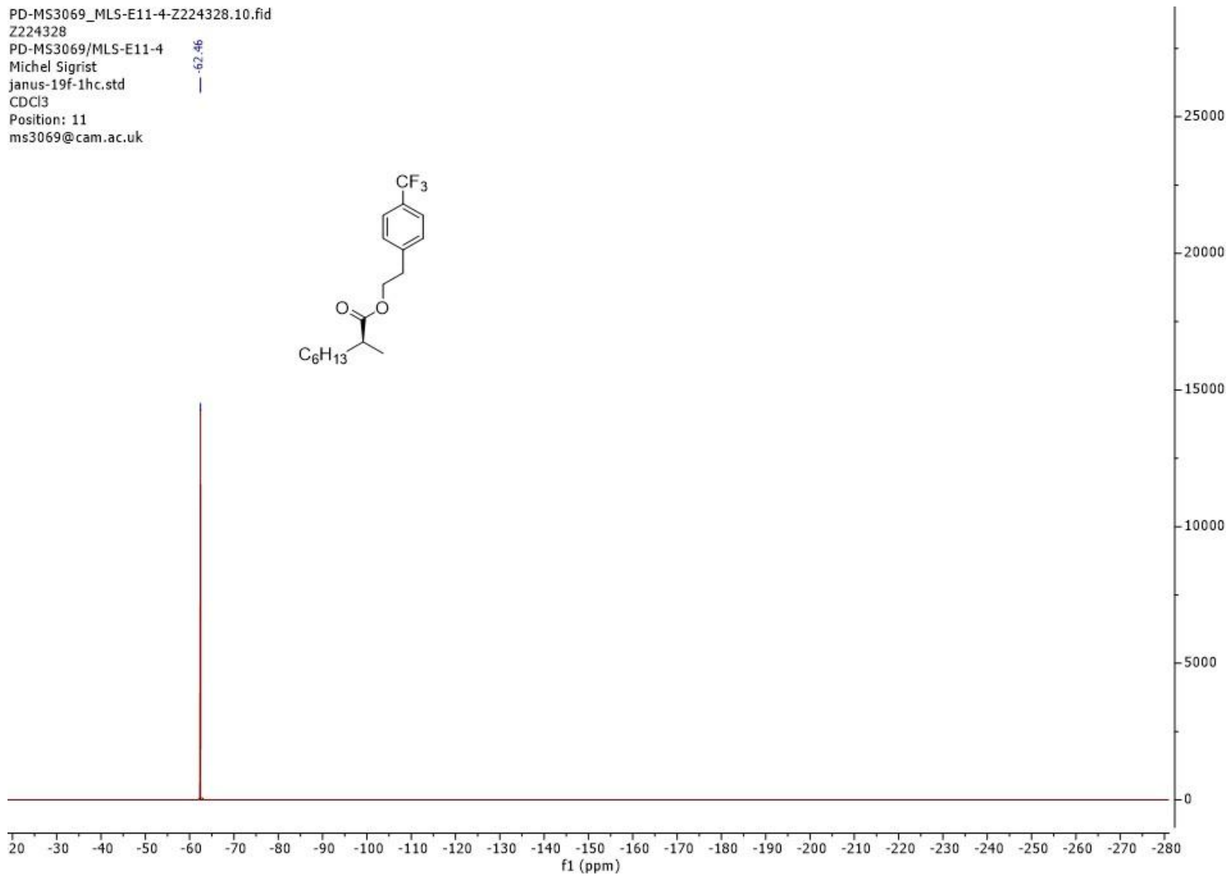
PD-MS3069_MLS-E11-4-Z224328.10.fid
Z224328
PD-MS3069/MLS-E11-4
Michel Sigrist
janus-19f-1hc.std
CDCl3
Position: 11
ms3069@cam.ac.uk
-62.46
CF3
O
O
C6H13
f1 (ppm)

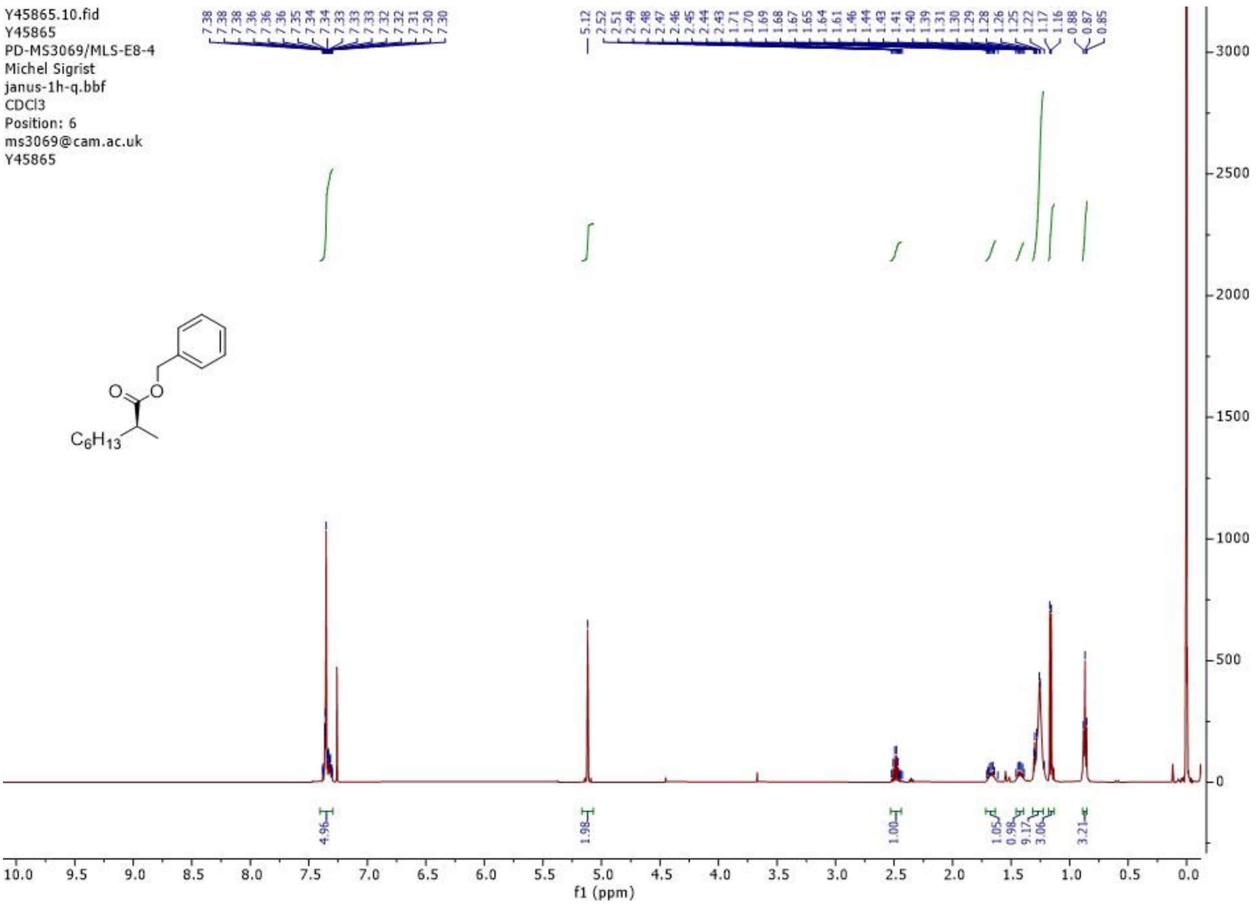

Y45865.10.fid
Y45865
PD-MS3069/MLS-E8-4
Michel Sigrist
janus-1h-q.bbf
CDCl3
Position: 6
ms3069@cam.ac.uk
Y45865
C6H13
f1 (ppm)

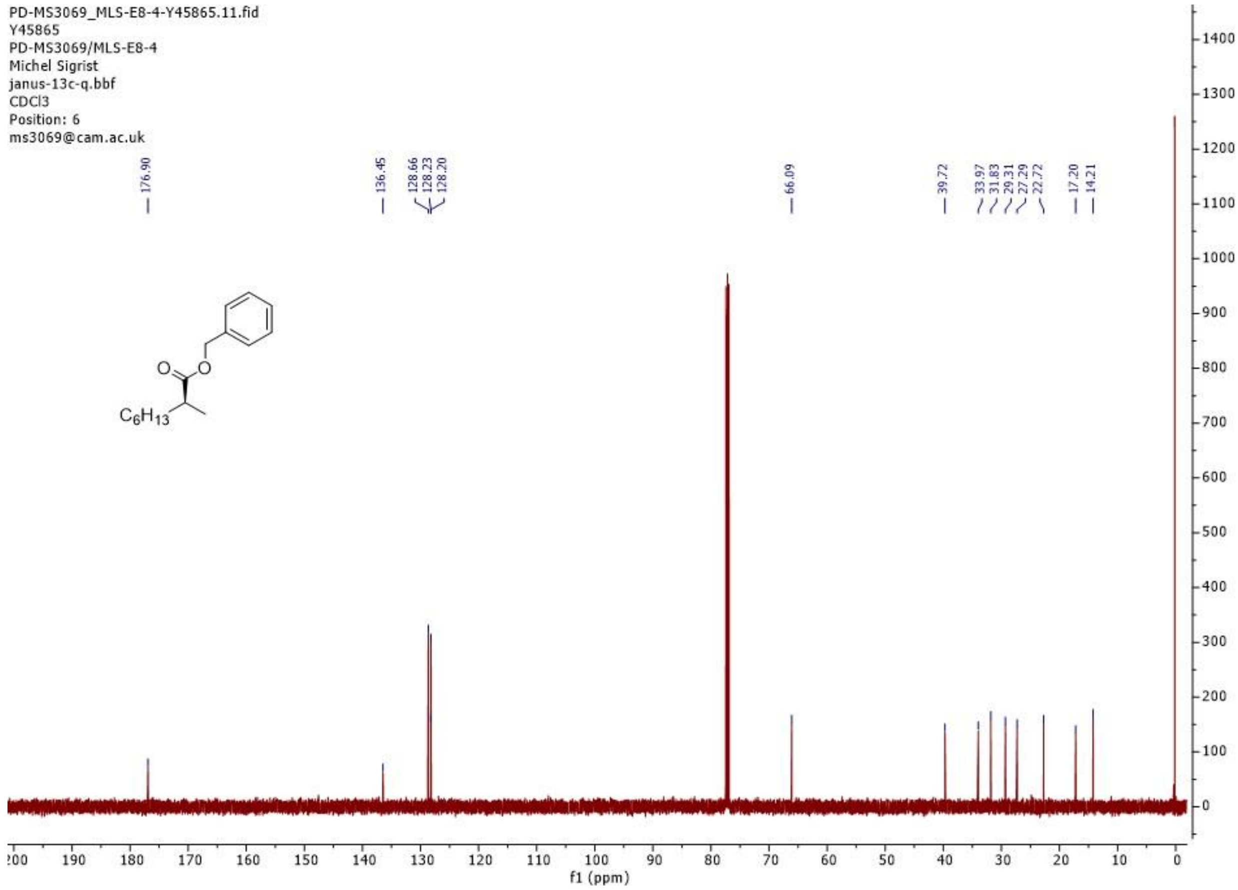

PD-MS3069_MLS-E8-4-Y45865.11.fid
Y45865
PD-MS3069/MLS-E8-4
Michel Sigrist
janus-13c-q.bbf
CDCl3
Position: 6
ms3069@cam.ac.uk
176.90
136.45
128.66
128.23
128.20
66.09
39.72
33.97
31.83
29.31
27.29
22.72
17.20
14.21
C6H13
f1 (ppm)

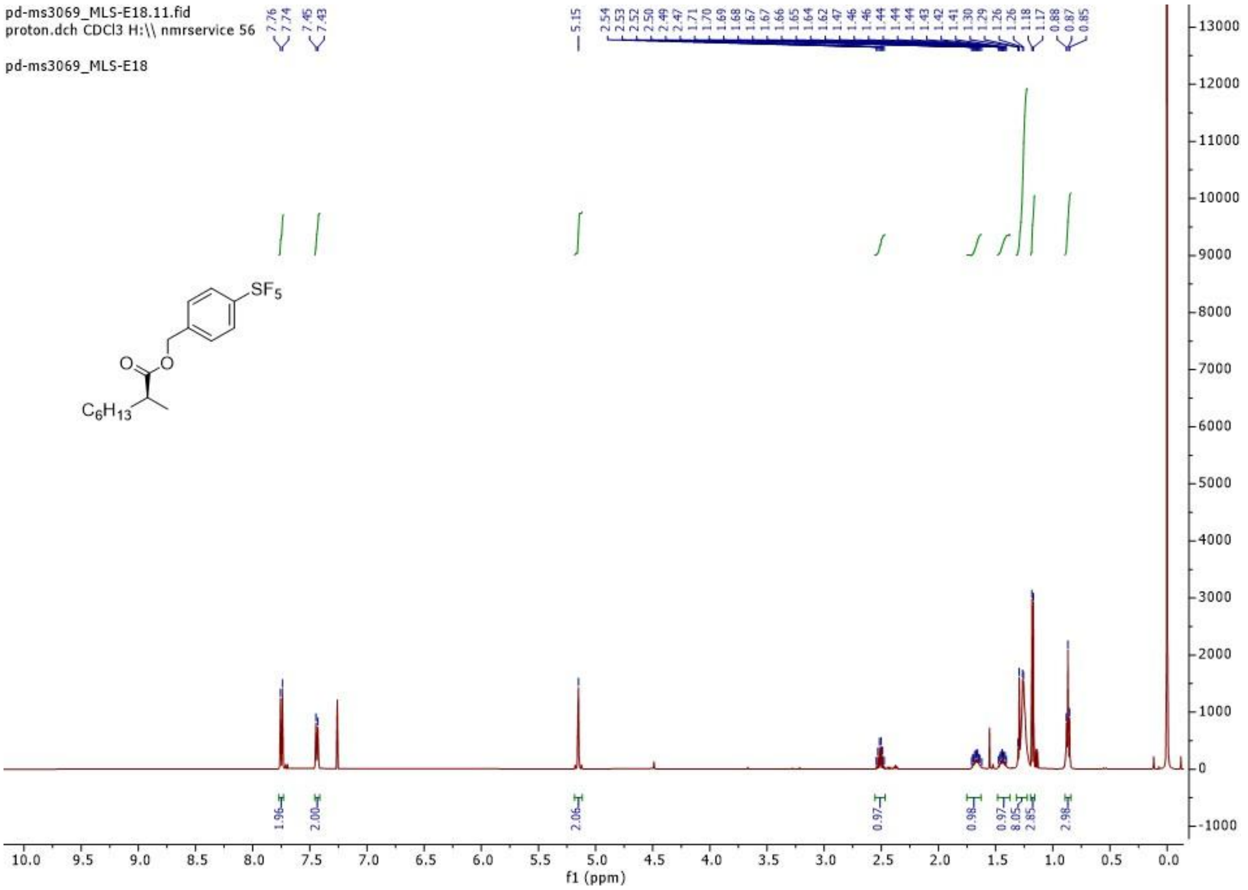
pd-ms3069_MLS-E18.11.fid
proton.dch CDCl3 H:\\ nmrservice 56
pd-ms3069_MLS-E18
7.76 7.74 7.45 7.43
5.15
2.54 2.53 2.52 2.50 2.49 2.47 1.71 1.70 1.69 1.68 1.67 1.67 1.66 1.65 1.64 1.62 1.47 1.46 1.46 1.44 1.44 1.44 1.43 1.42 1.41 1.30 1.29 1.26 1.26 1.18 1.17 0.88 0.87 0.85
SF_5
O
O
C_6H_{13}
1.96 2.00 2.06 0.97 0.98 0.97 8.05 2.85 2.98
f1 (ppm)

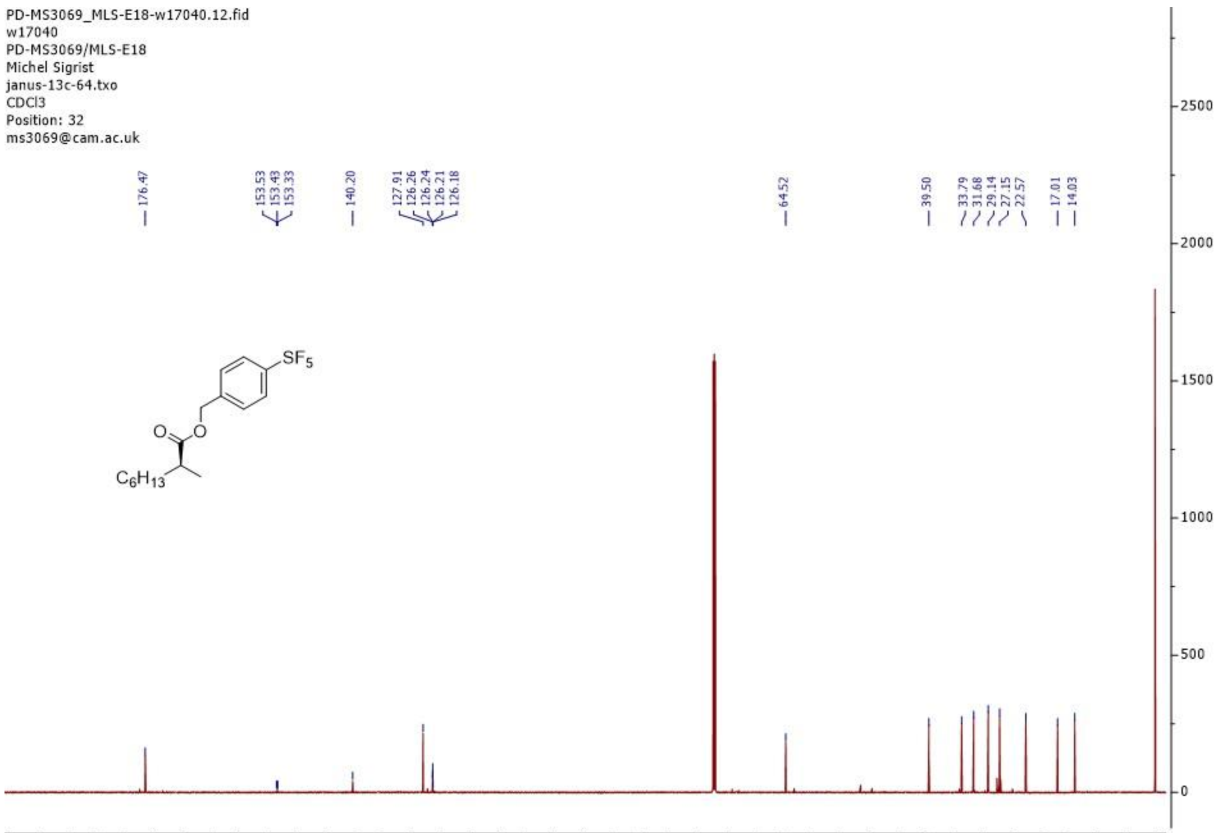
PD-MS3069_MLS-E18-w17040.12.fid
w17040
PD-MS3069/MLS-E18
Michel Sigrist
janus-13c-64.txo
CDCl3
Position: 32
ms3069@cam.ac.uk
176.47
153.53 153.43 153.33
140.20
127.91 126.26 126.24 126.21 126.18
64.52
39.50
33.79 31.68 29.14 27.15 22.57
17.01 14.03
SF_5
O
O
C_6H_{13}
f1 (ppm)

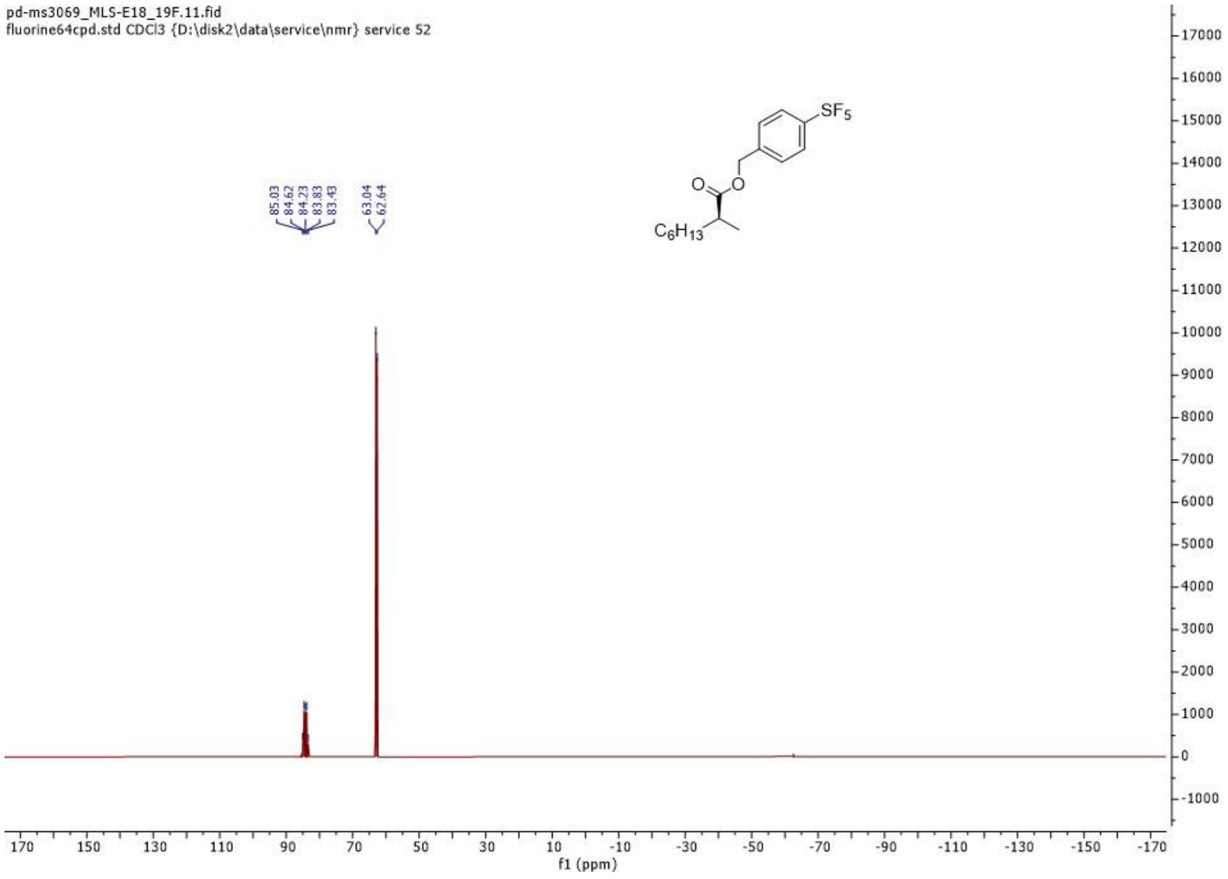

pd-ms3069_MLS-E18_19F.11.fid
fluorine64cpd.std CDCl3 {D:\disk2\data\service\nmr} service 52
85.03
84.62
84.23
83.83
83.43
63.04
62.64
SF5
O
O
C6H13
f1 (ppm)

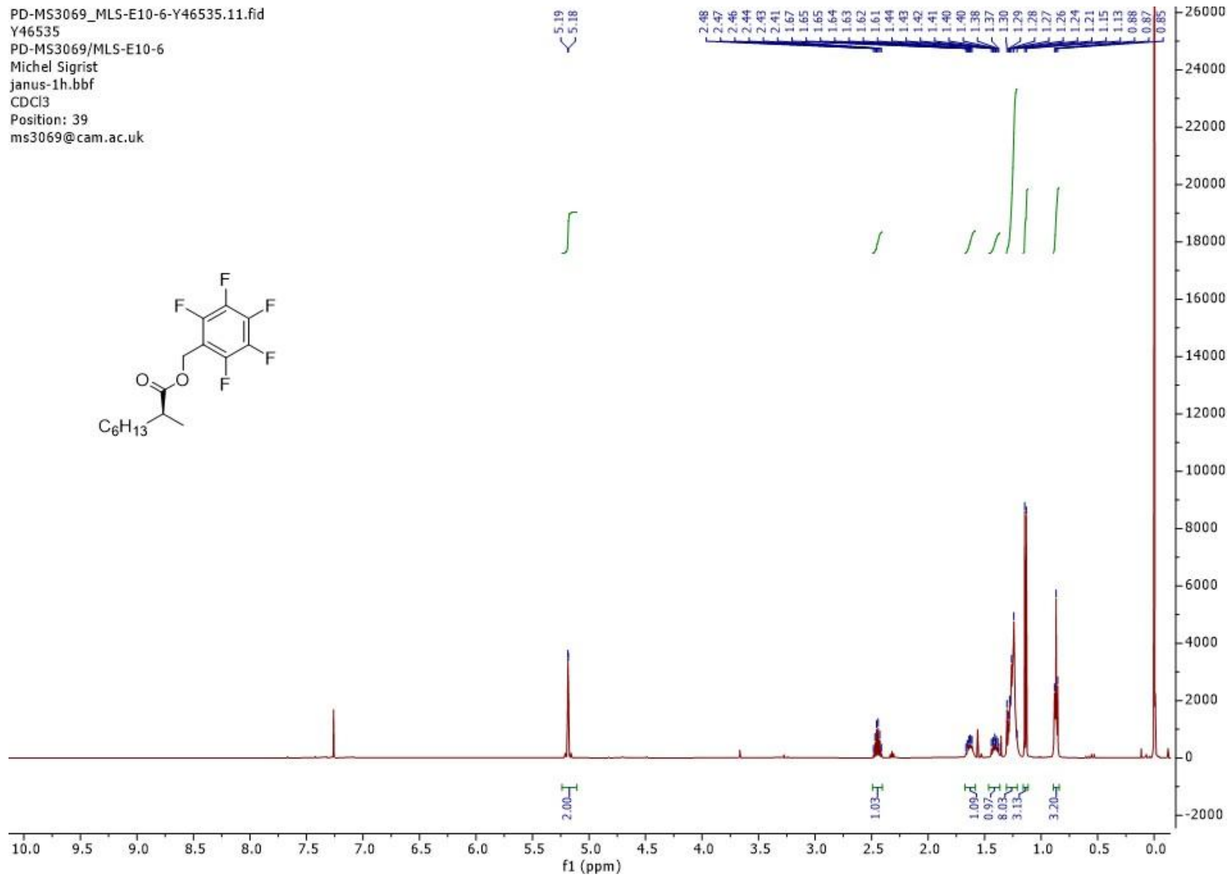
PD-MS3069_MLS-E10-6-Y46535.11.fid
Y46535
PD-MS3069/MLS-E10-6
Michel Sigrist
janus-1h.bbf
CDCl3
Position: 39
ms3069@cam.ac.uk
5.19
5.18
2.48
2.47
2.46
2.44
2.43
2.41
1.67
1.65
1.65
1.64
1.63
1.62
1.61
1.44
1.43
1.42
1.41
1.40
1.40
1.38
1.37
1.30
1.29
1.28
1.27
1.26
1.24
1.21
1.15
1.13
0.88
0.87
0.85
2.00
1.03
1.09
0.97
8.03
3.13
3.20
F
F
F
F
F
O
O
C_6H_{13}
f1 (ppm)

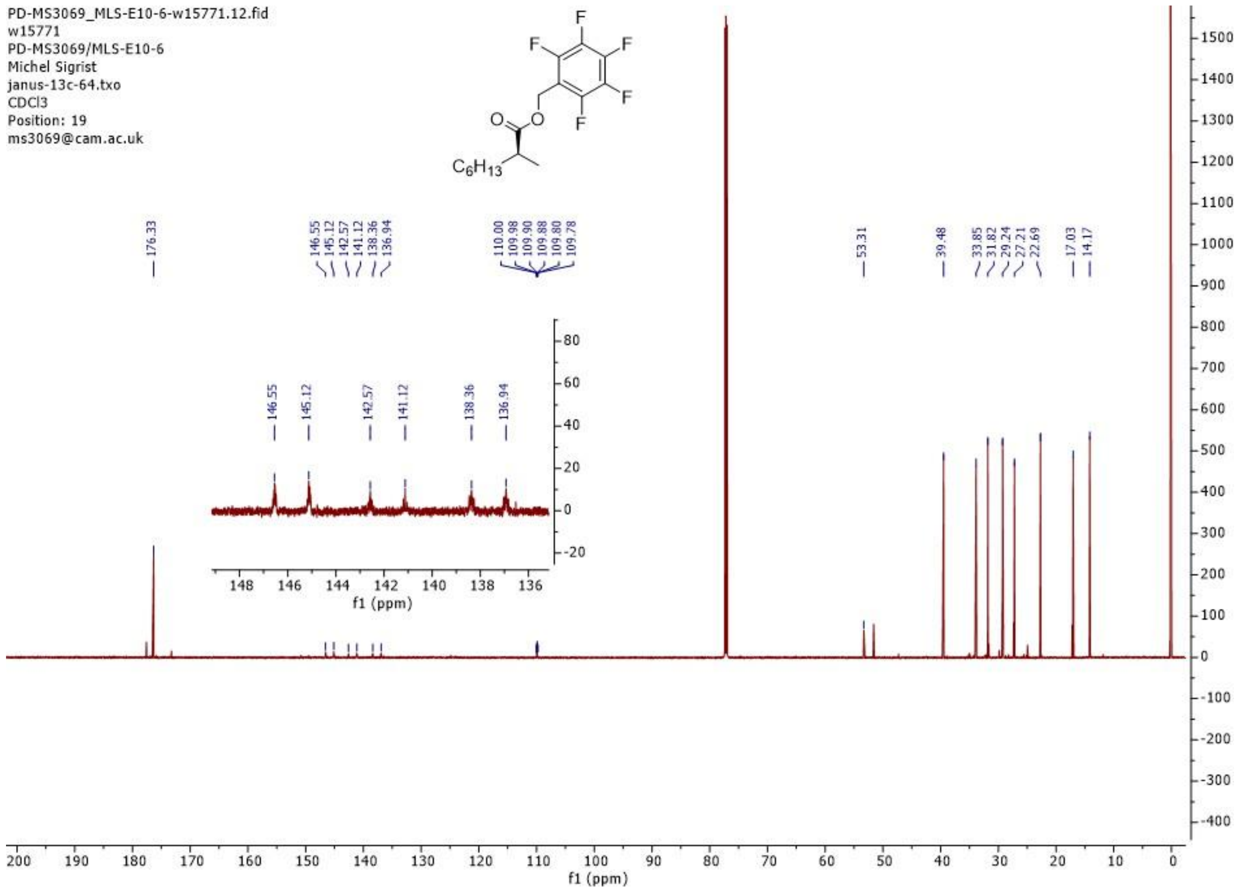
PD-MS3069_MLS-E10-6-w15771.12.fid
w15771
PD-MS3069/MLS-E10-6
Michel Sigrist
janus-13c-64.txo
CDCl3
Position: 19
ms3069@cam.ac.uk
176.33
146.55
145.12
142.57
141.12
138.36
136.94
110.00
109.98
109.90
109.88
109.80
109.78
53.31
39.48
33.85
31.82
29.24
27.21
22.69
17.03
14.17
F
F
F
F
F
O
O
C_6H_{13}
f1 (ppm)

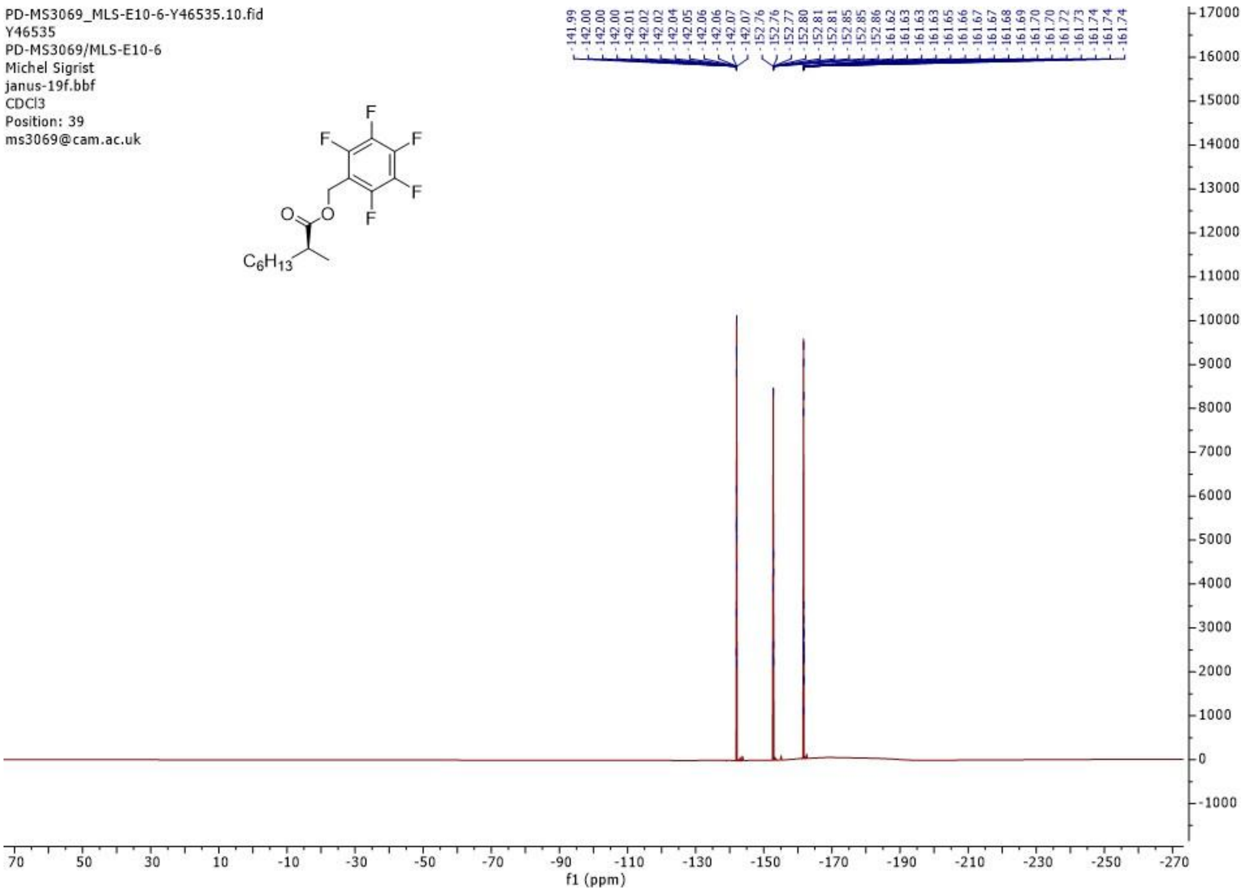
PD-MS3069_MLS-E10-6-Y46535.10.fid
Y46535
PD-MS3069/MLS-E10-6
Michel Sigrist
janus-19f.bbf
CDCl3
Position: 39
ms3069@cam.ac.uk
f1 (ppm)

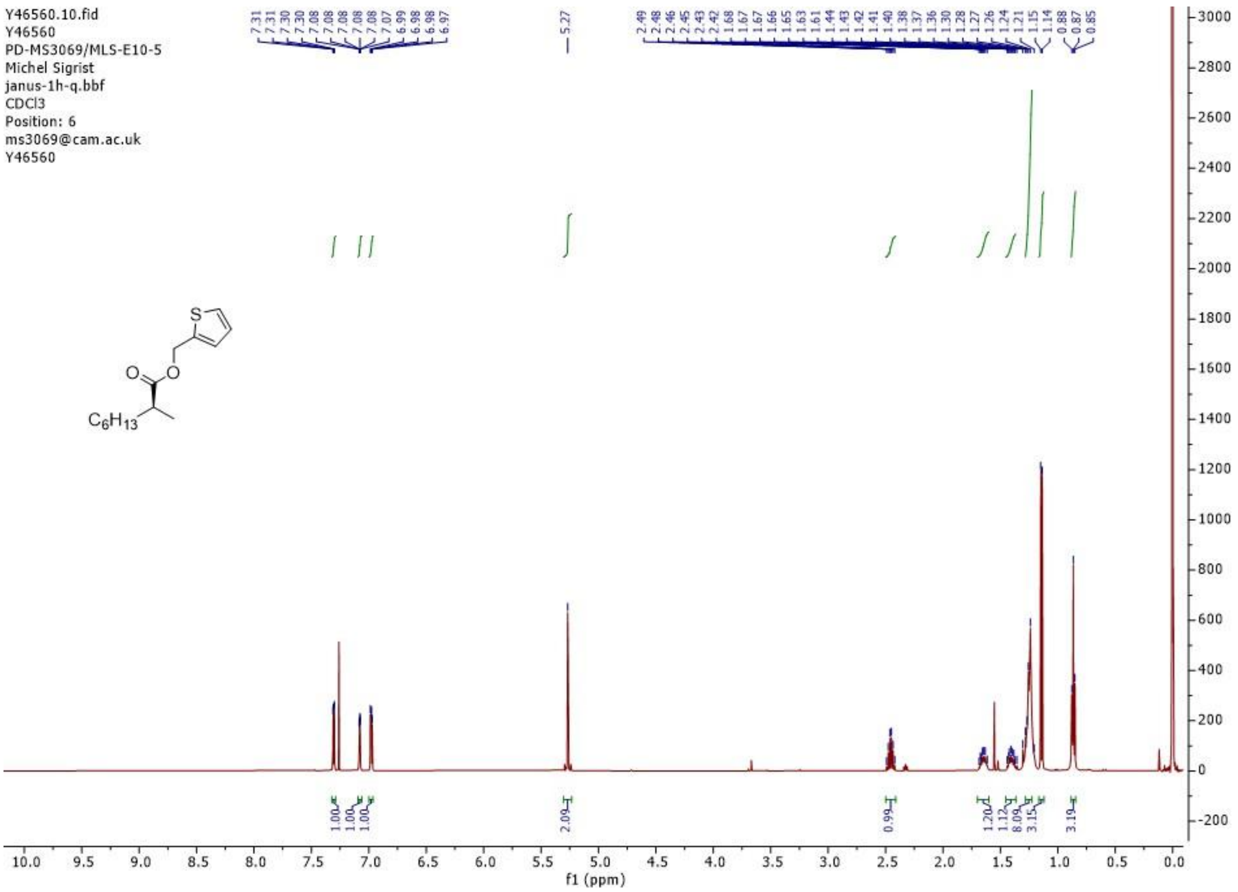
Y46560.10.fid
Y46560
PD-MS3069/MLS-E10-5
Michel Sigrist
janus-1h-q.bbf
CDCl3
Position: 6
ms3069@cam.ac.uk
Y46560
7.31 7.31 7.30 7.30 7.08 7.08 7.08 7.08 7.07 6.99 6.98 6.98 6.97
5.27
2.49 2.48 2.46 2.45 2.43 2.42 1.68 1.67 1.67 1.66 1.65 1.63 1.61 1.44 1.43 1.42 1.41 1.40 1.38 1.37 1.36 1.30 1.28 1.27 1.26 1.24 1.21 1.15 1.14 0.88 0.87 0.85
S
O
O
C_6H_{13}
1.00 1.00 1.00
2.09
0.99
1.20 1.12 8.09 3.15
3.19
f1 (ppm)

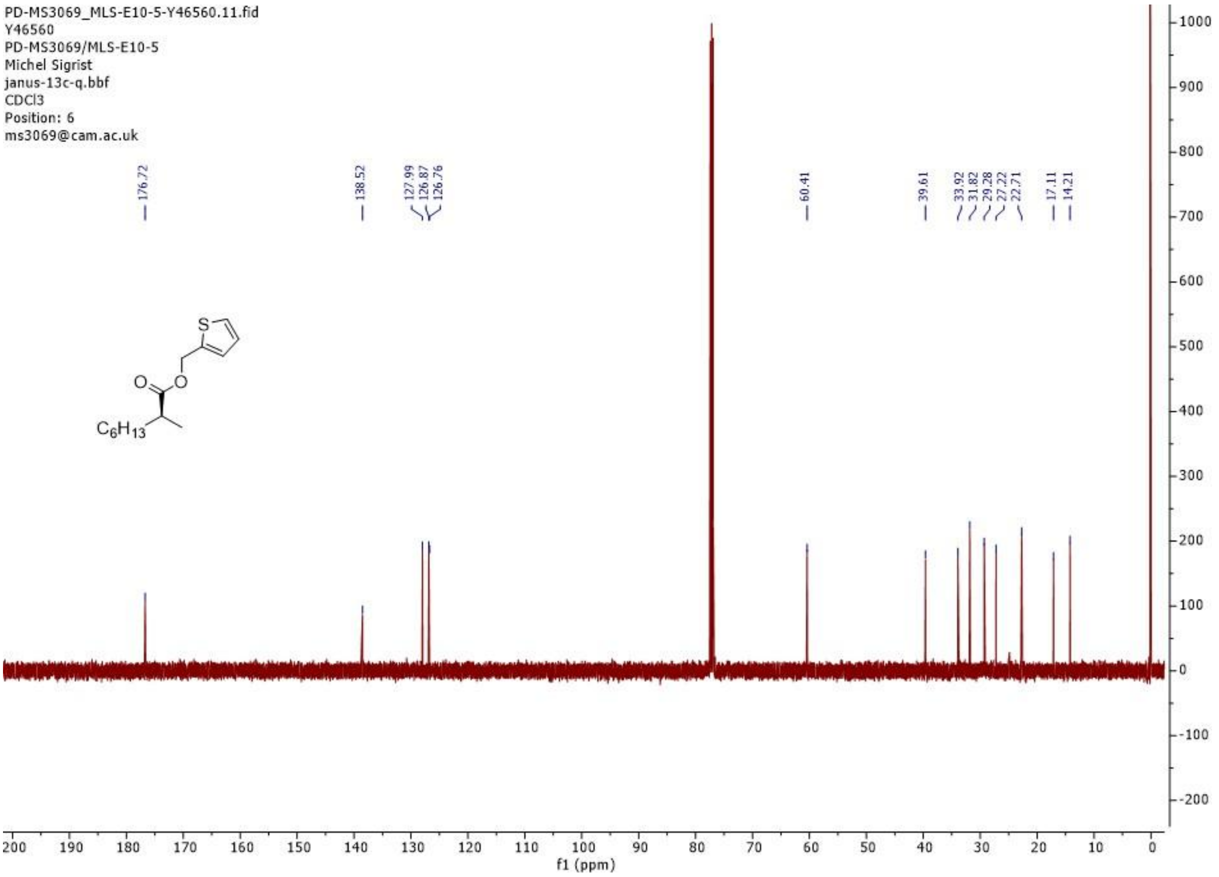
PD-MS3069_MLS-E10-5-Y46560.11.fid
Y46560
PD-MS3069/MLS-E10-5
Michel Sigrist
janus-13c-q.bbf
CDCl3
Position: 6
ms3069@cam.ac.uk
176.72
138.52
127.99 126.87 126.76
60.41
39.61
33.92 31.82 29.28 27.22 22.71
17.11 14.21
S
O
O
C_6H_{13}
f1 (ppm)

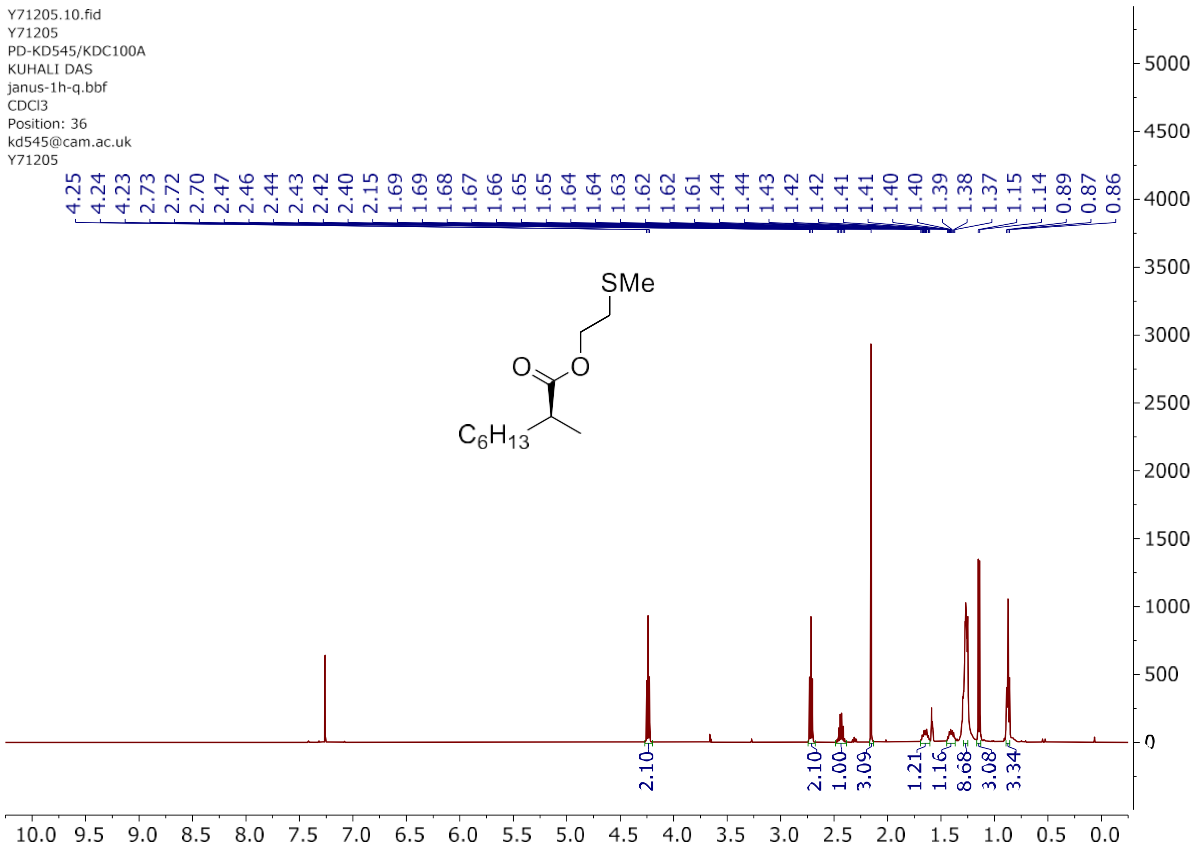
Y71205.10.fid
Y71205
PD-KD545/KDC100A
KUHALI DAS
janus-1h-q.bbf
CDCl3
Position: 36
kd545@cam.ac.uk
Y71205
4.25 4.24 4.23 2.73 2.72 2.70 2.47 2.46 2.44 2.43 2.42 2.40 2.15 1.69 1.69 1.68 1.67 1.66 1.65 1.65 1.64 1.64 1.63 1.62 1.62 1.61 1.44 1.44 1.43 1.42 1.42 1.41 1.41 1.40 1.40 1.39 1.38 1.37 1.15 1.14 0.89 0.87 0.86
SMe
O
O
C6H13
2.10
2.10
1.00
3.09
1.21
1.16
8.68
3.08
3.34
10.0 9.5 9.0 8.5 8.0 7.5 7.0 6.5 6.0 5.5 5.0 4.5 4.0 3.5 3.0 2.5 2.0 1.5 1.0 0.5 0.0
5000 4500 4000 3500 3000 2500 2000 1500 1000 500 0

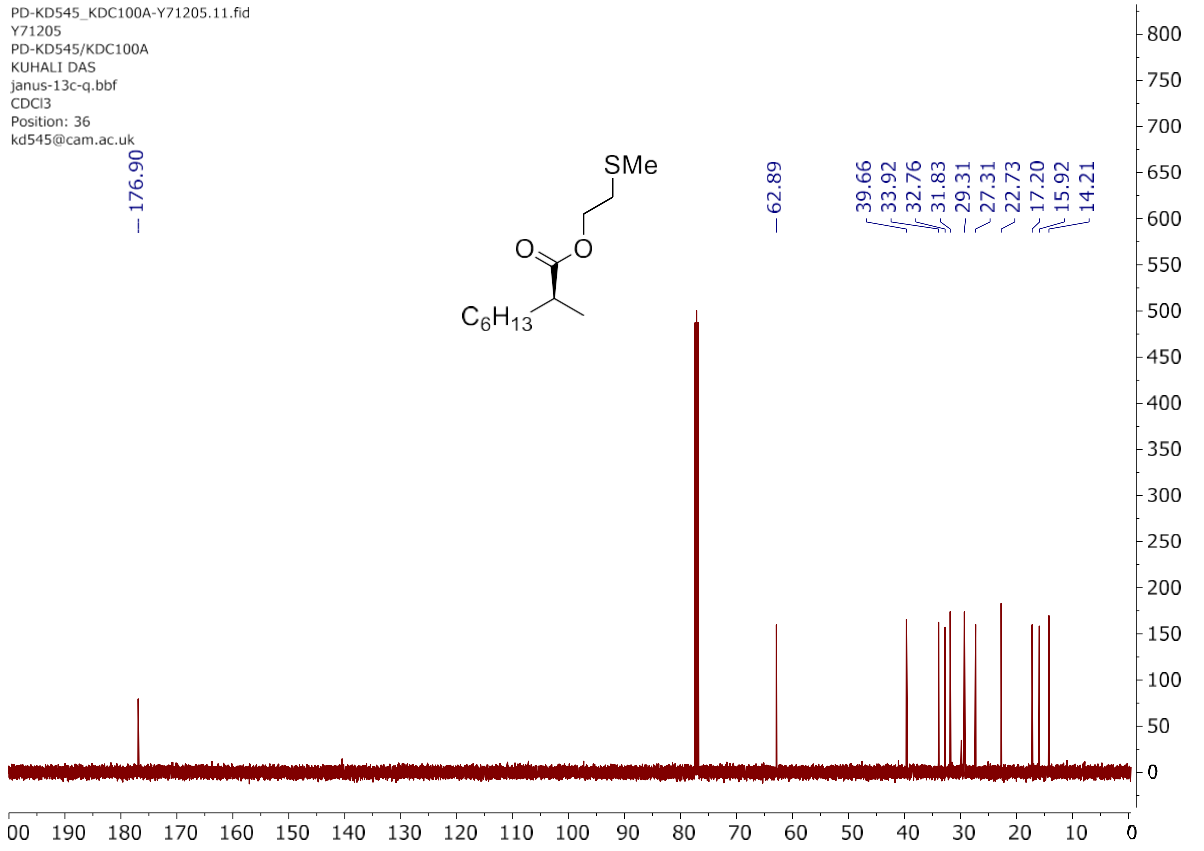
PD-KD545_KDC100A-Y71205.11.fid
Y71205
PD-KD545/KDC100A
KUHALI DAS
janus-13c-q.bbf
CDCl3
Position: 36
kd545@cam.ac.uk
176.90
SMe
O
O
C6H13
62.89
39.66 33.92 32.76 31.83 29.31 27.31 22.73 17.20 15.92 14.21
00 190 180 170 160 150 140 130 120 110 100 90 80 70 60 50 40 30 20 10 0
800 750 700 650 600 550 500 450 400 350 300 250 200 150 100 50 0

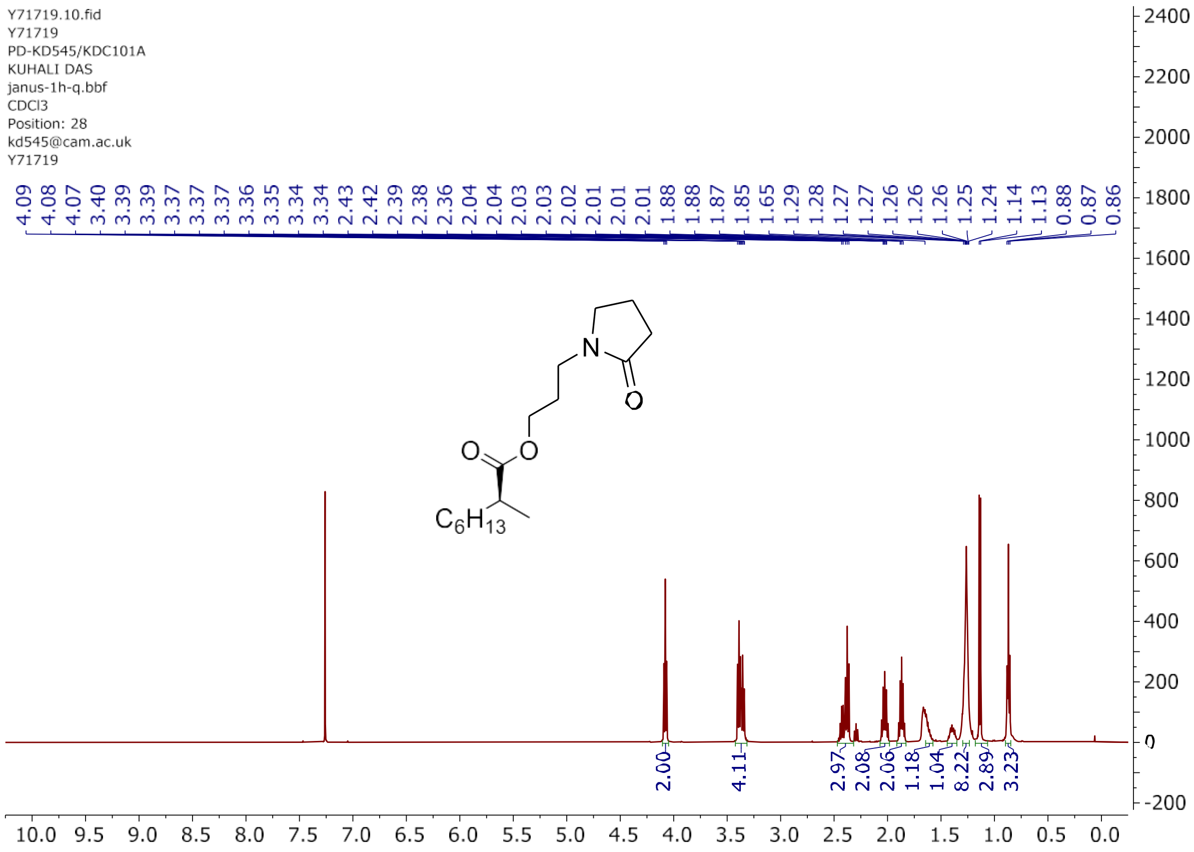
Y71719.10.fid
Y71719
PD-KD545/KDC101A
KUHALI DAS
janus-1h-q.bbf
CDCl3
Position: 28
kd545@cam.ac.uk
Y71719
4.09 4.08 4.07 3.40 3.39 3.39 3.37 3.37 3.37 3.36 3.35 3.34 3.34 2.43 2.42 2.39 2.38 2.36 2.04 2.04 2.03 2.03 2.02 2.01 2.01 2.01 1.88 1.88 1.87 1.85 1.65 1.29 1.28 1.27 1.27 1.26 1.26 1.26 1.25 1.24 1.14 1.13 0.88 0.87 0.86
N
O
O
O
C6H13
2.00 4.11 2.97 2.08 2.06 1.18 1.04 8.22 2.89 3.23

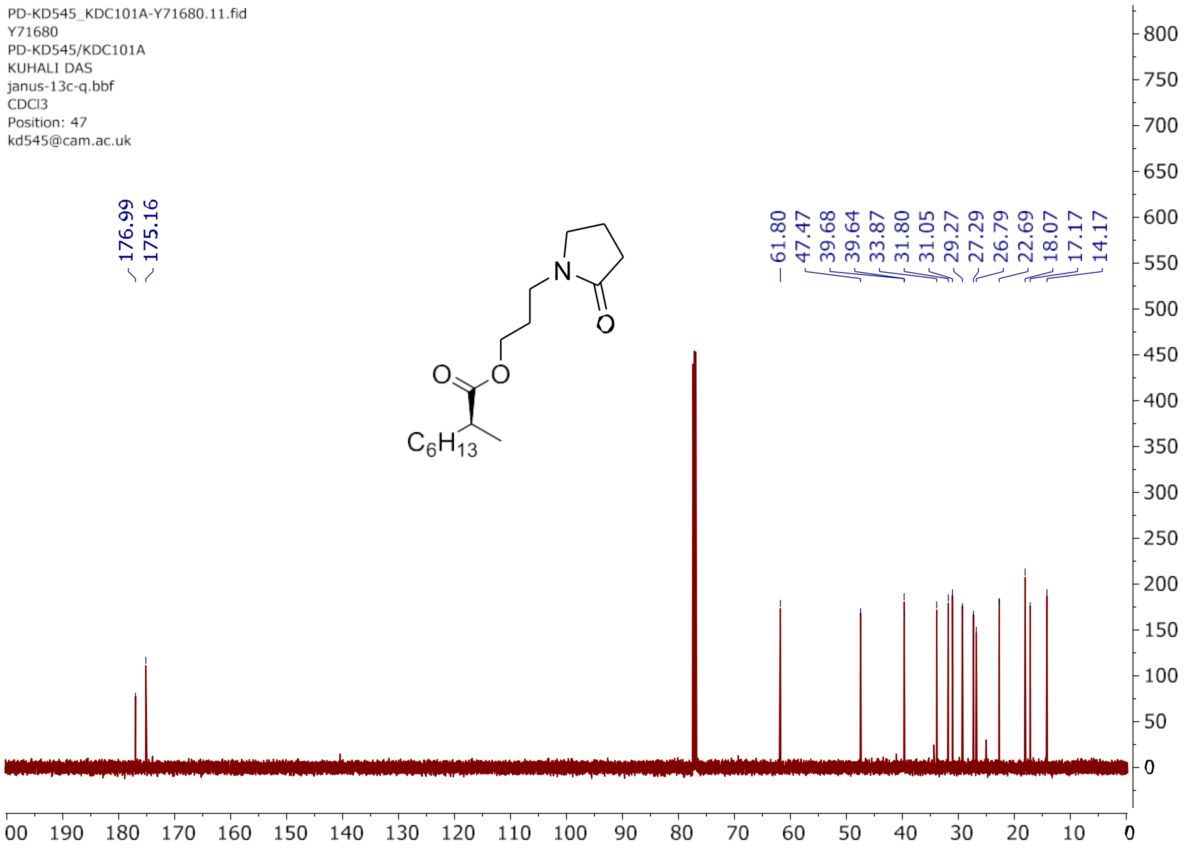
PD-KD545_KDC101A-Y71680.11.fid
Y71680
PD-KD545/KDC101A
KUHALI DAS
janus-13c-q.bbf
CDCl3
Position: 47
kd545@cam.ac.uk
176.99 175.16
61.80 47.47 39.68 39.64 33.87 31.80 31.05 29.27 27.29 26.79 22.69 18.07 17.17 14.17
N
O
O
O
C6H13

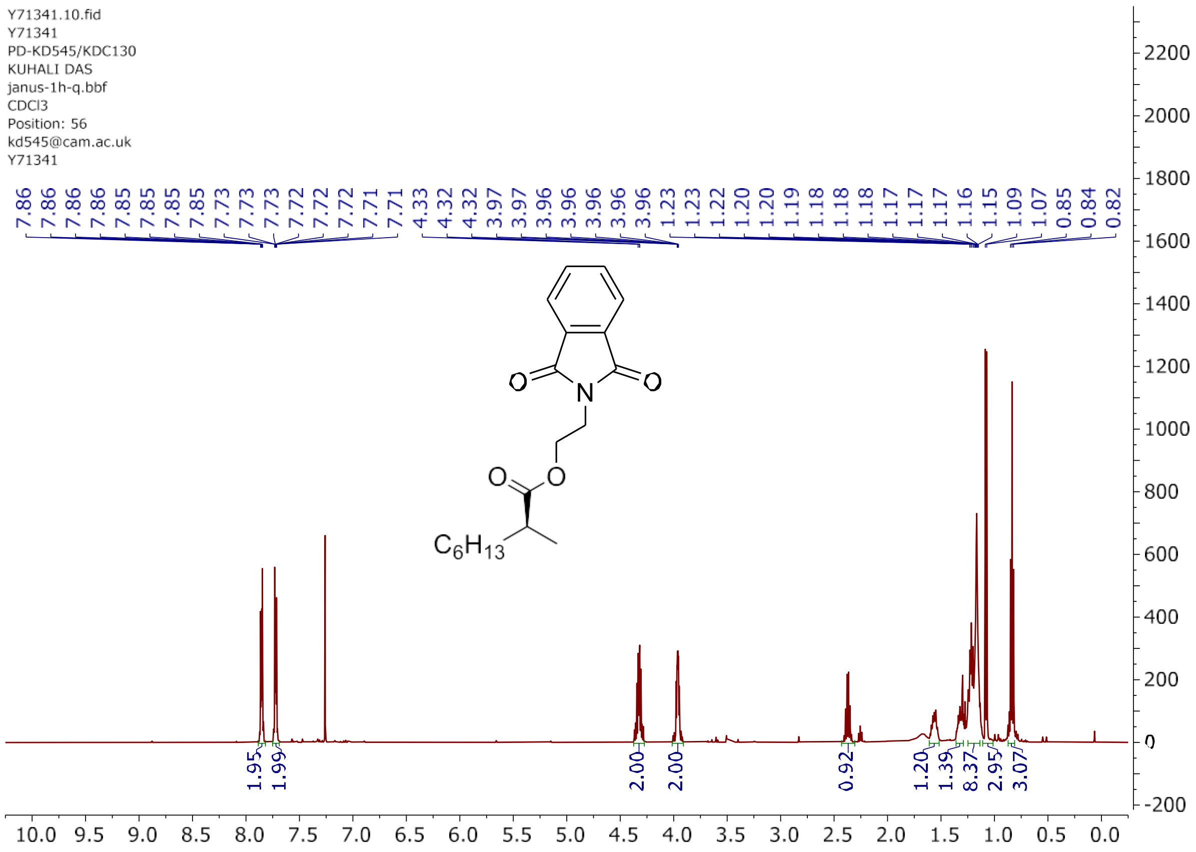
Y71341.10.fid
Y71341
PD-KD545/KDC130
KUHALI DAS
janus-1h-q.bbf
CDCl3
Position: 56
kd545@cam.ac.uk
Y71341
7.86 7.86 7.86 7.86 7.85 7.85 7.85 7.85 7.73 7.73 7.73 7.72 7.72 7.72 7.71 7.71 4.33 4.32 4.32 3.97 3.97 3.96 3.96 3.96 3.96 3.96 1.23 1.23 1.22 1.20 1.20 1.19 1.18 1.18 1.18 1.17 1.17 1.17 1.16 1.15 1.09 1.07 0.85 0.84 0.82
1.95 1.99 2.00 2.00 0.92 1.20 1.39 8.37 2.95 3.07
C_6H_{13}

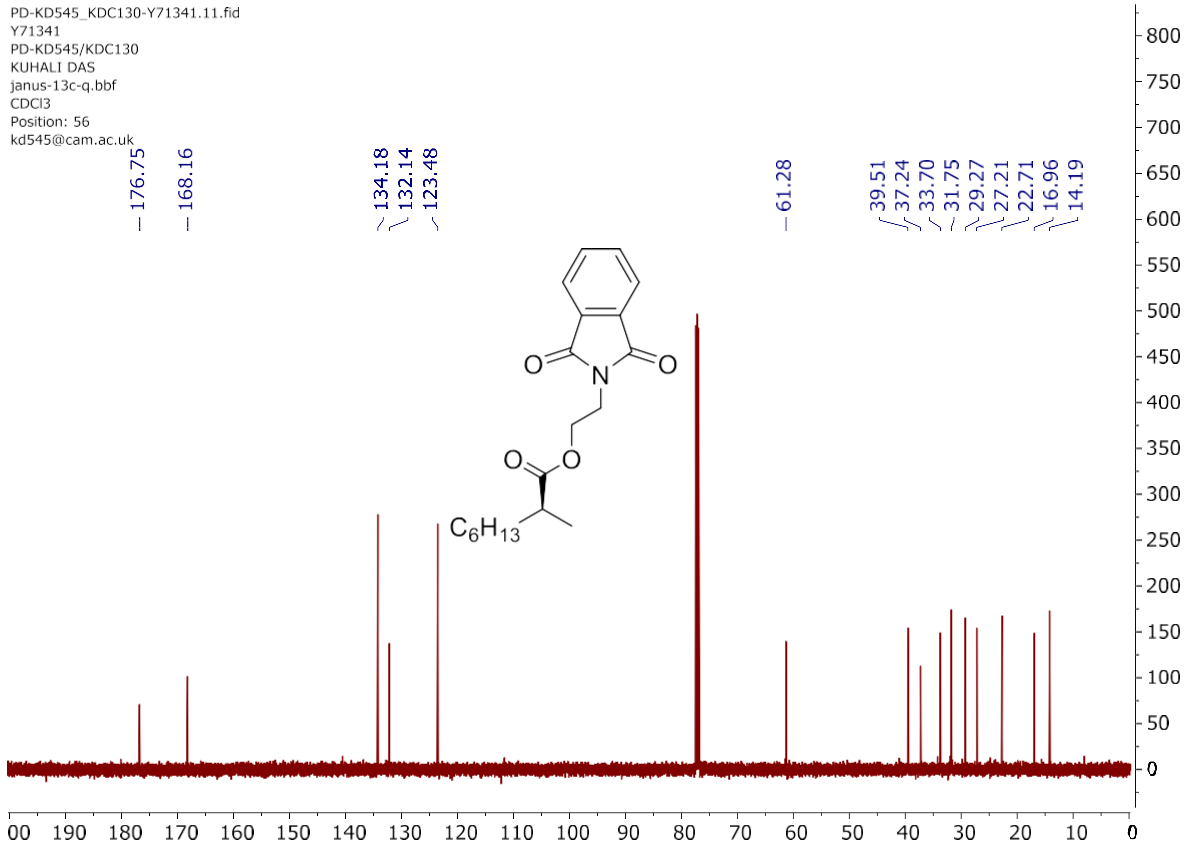
PD-KD545_KDC130-Y71341.11.fid
Y71341
PD-KD545/KDC130
KUHALI DAS
janus-13c-q.bbf
CDCl3
Position: 56
kd545@cam.ac.uk
176.75 168.16 134.18 132.14 123.48 61.28 39.51 37.24 33.70 31.75 29.27 27.21 22.71 16.96 14.19
C_6H_{13}

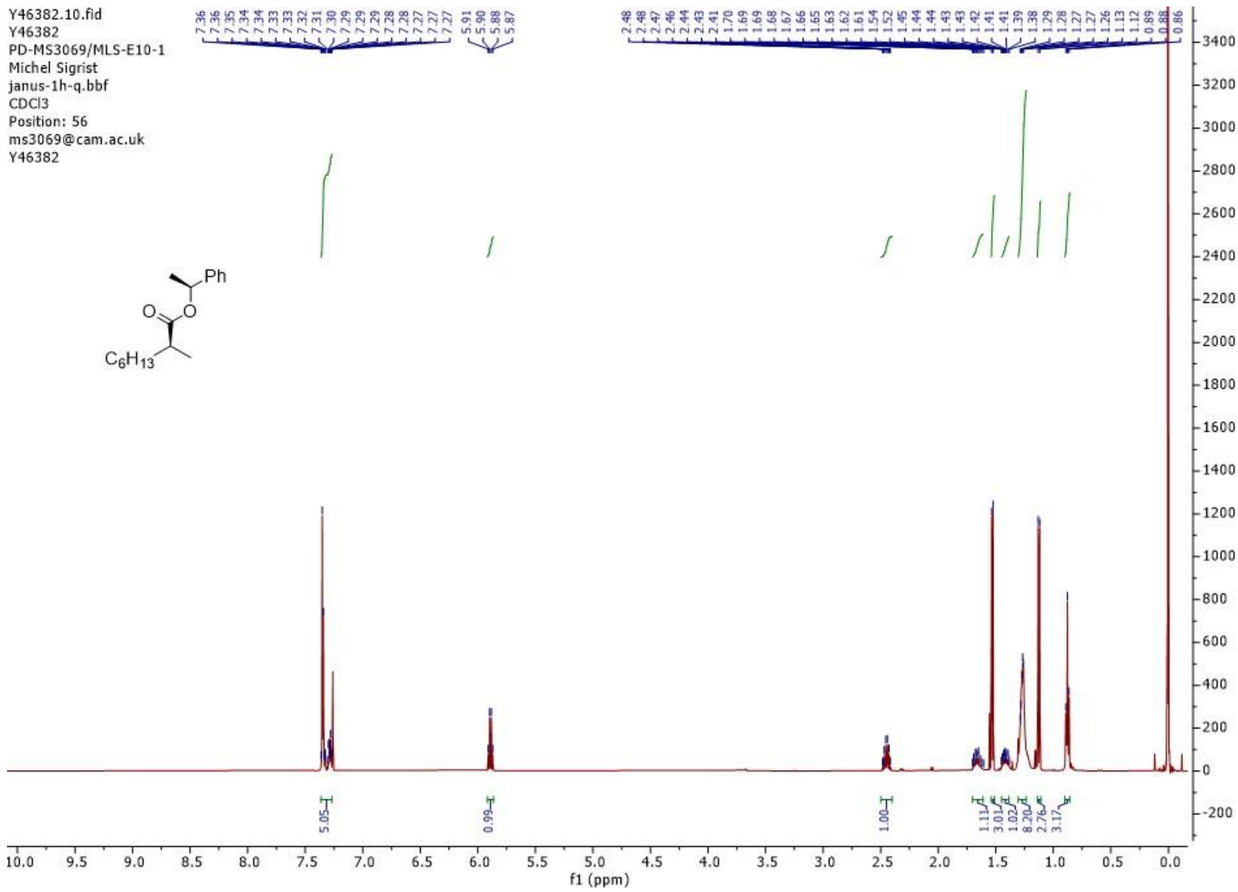
Y46382.10.fid
Y46382
PD-MS3069/MLS-E10-1
Michel Sigrist
janus-1h-q.bbf
CDCl3
Position: 56
ms3069@cam.ac.uk
Y46382
f1 (ppm)

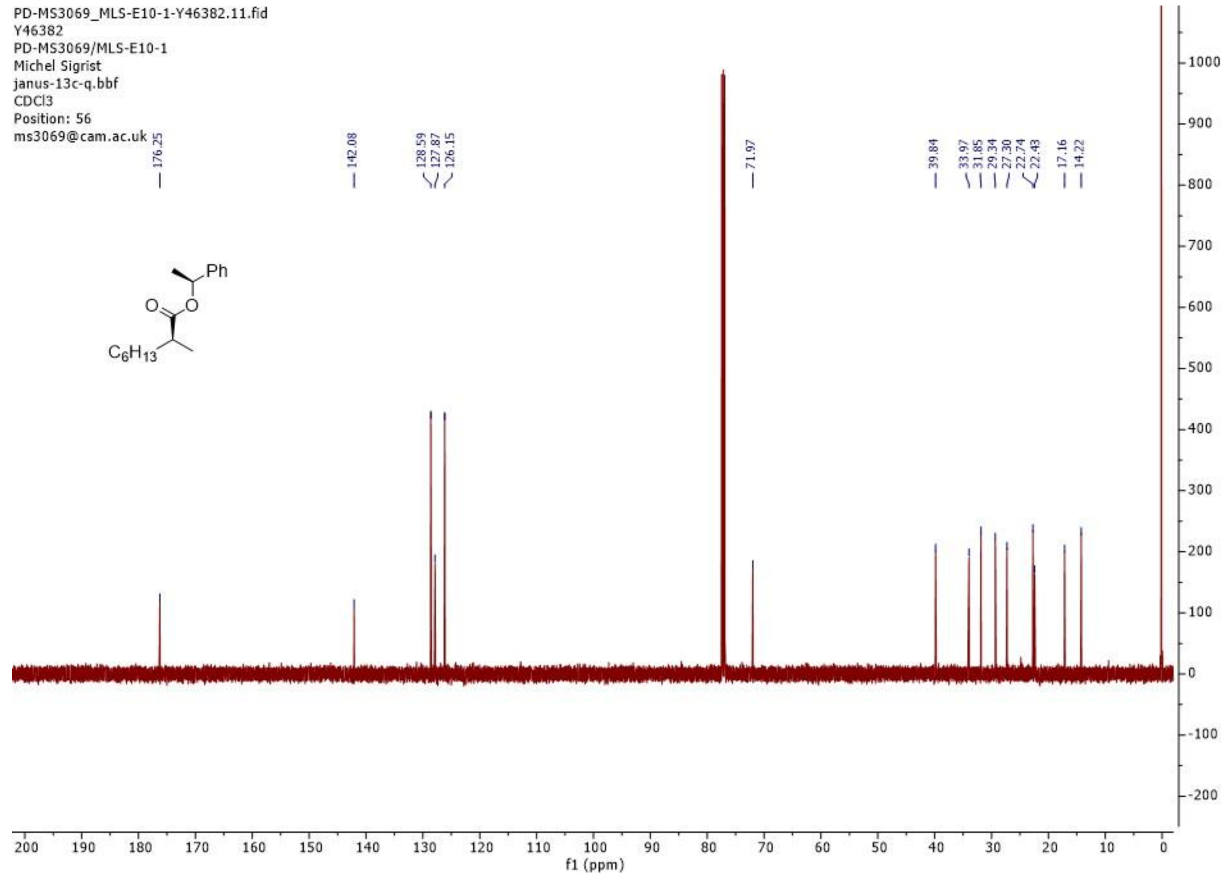
PD-MS3069_MLS-E10-1-Y46382.11.fid
Y46382
PD-MS3069/MLS-E10-1
Michel Sigrist
janus-13c-q.bbf
CDCl3
Position: 56
ms3069@cam.ac.uk
176.25
142.08
128.59
127.87
126.15
71.97
39.84
33.97
31.85
29.34
27.30
22.74
22.43
17.16
14.22
f1 (ppm)

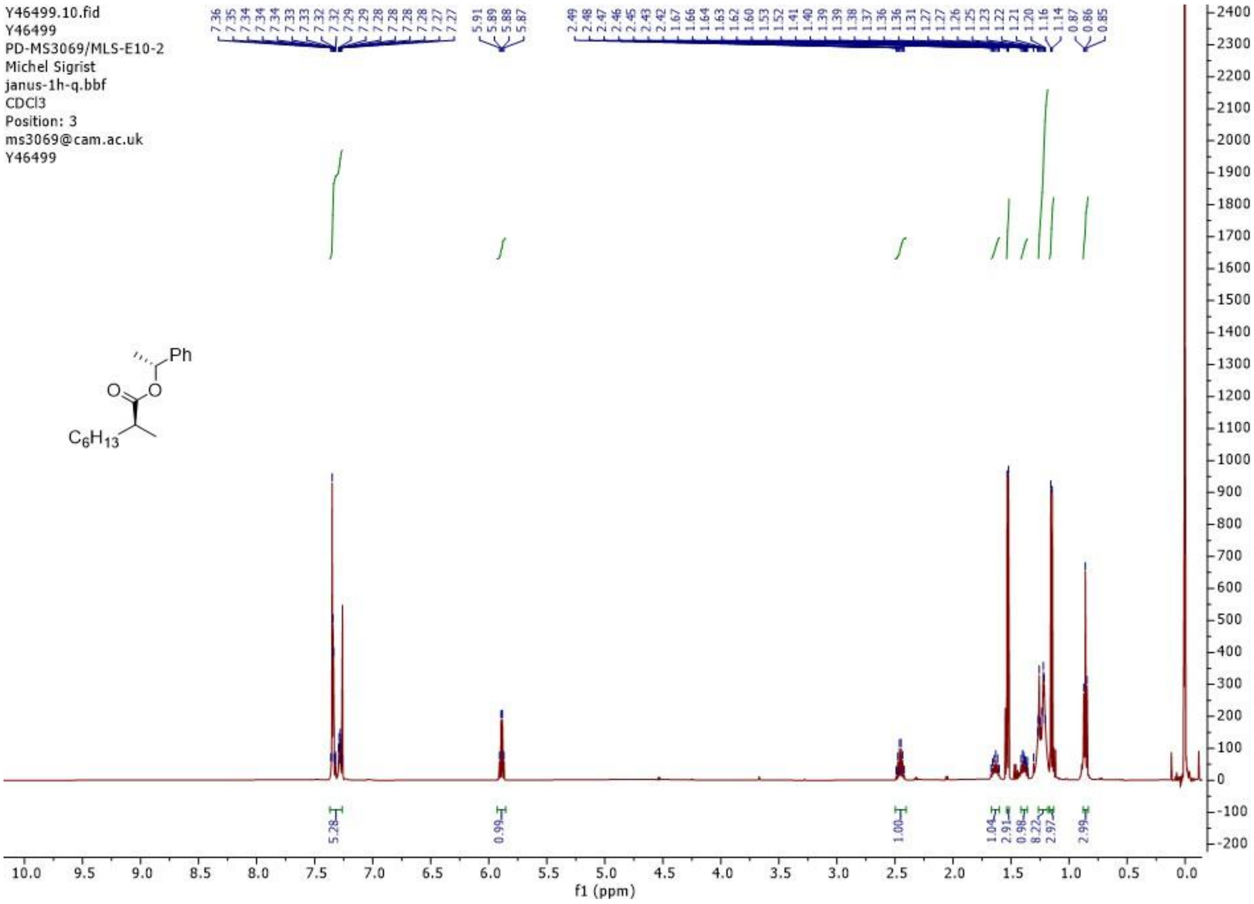
Y46499.10.fid
Y46499
PD-MS3069/MLS-E10-2
Michel Sigrist
janus-1h-q.bbf
CDCl3
Position: 3
ms3069@cam.ac.uk
Y46499
7.36 7.35 7.34 7.34 7.34 7.33 7.33 7.32 7.32 7.29 7.29 7.28 7.28 7.28 7.27 7.27
5.91 5.89 5.88 5.87
2.49 2.48 2.47 2.46 2.45 2.43 2.42 1.67 1.66 1.64 1.63 1.62 1.60 1.53 1.52 1.41 1.40 1.39 1.39 1.38 1.37 1.36 1.36 1.31 1.27 1.27 1.26 1.25 1.23 1.22 1.21 1.20 1.16 1.14 0.87 0.86 0.85
Ph
O
O
C_6H_{13}
5.28
0.99
1.00
1.04
2.91
0.98
8.22
2.97
2.99
f1 (ppm)

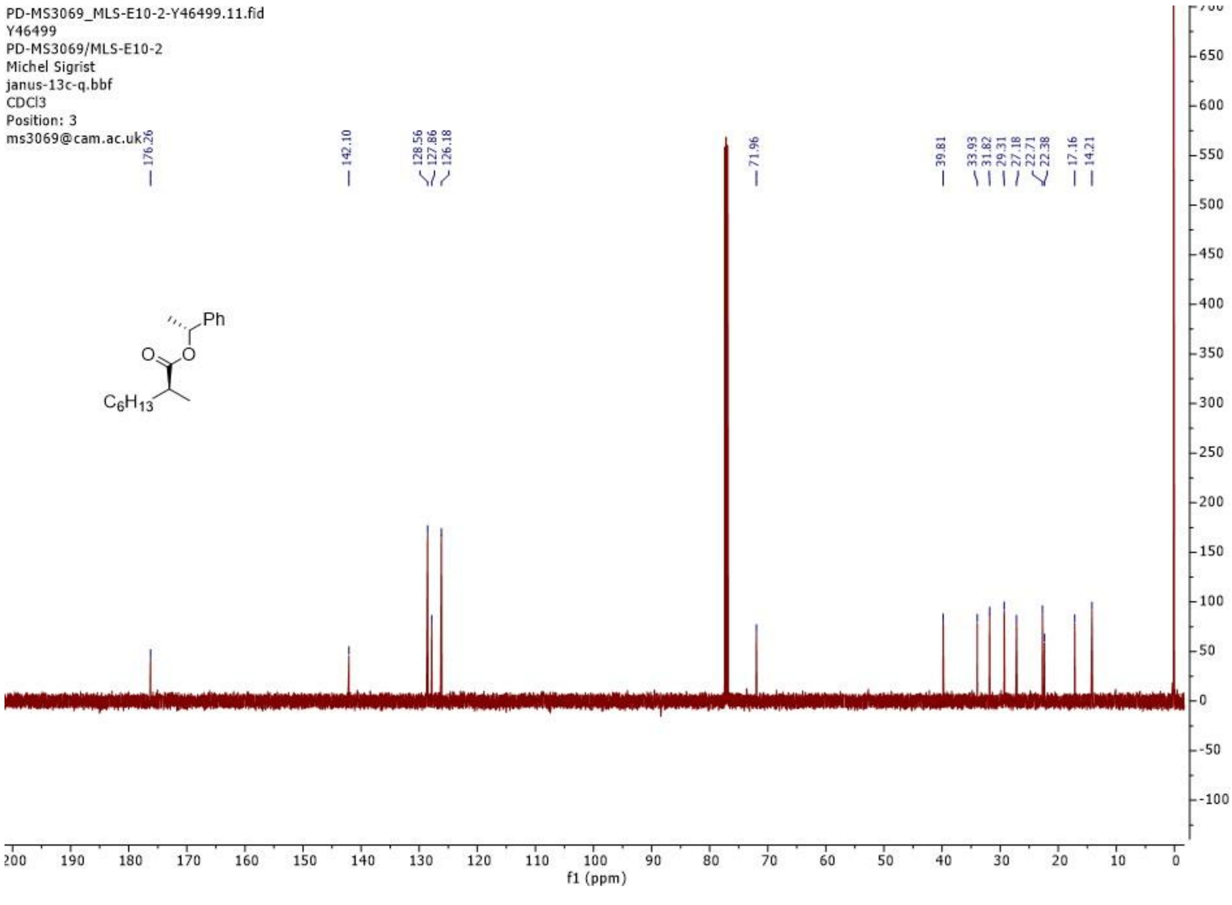
PD-MS3069_MLS-E10-2-Y46499.11.fid
Y46499
PD-MS3069/MLS-E10-2
Michel Sigrist
janus-13c-q.bbf
CDCl3
Position: 3
ms3069@cam.ac.uk
176.26
142.10
128.56 127.86 126.18
71.96
39.81
33.93 31.82 29.31 27.18 22.71 22.38
17.16 14.21
Ph
O
O
C_6H_{13}
f1 (ppm)

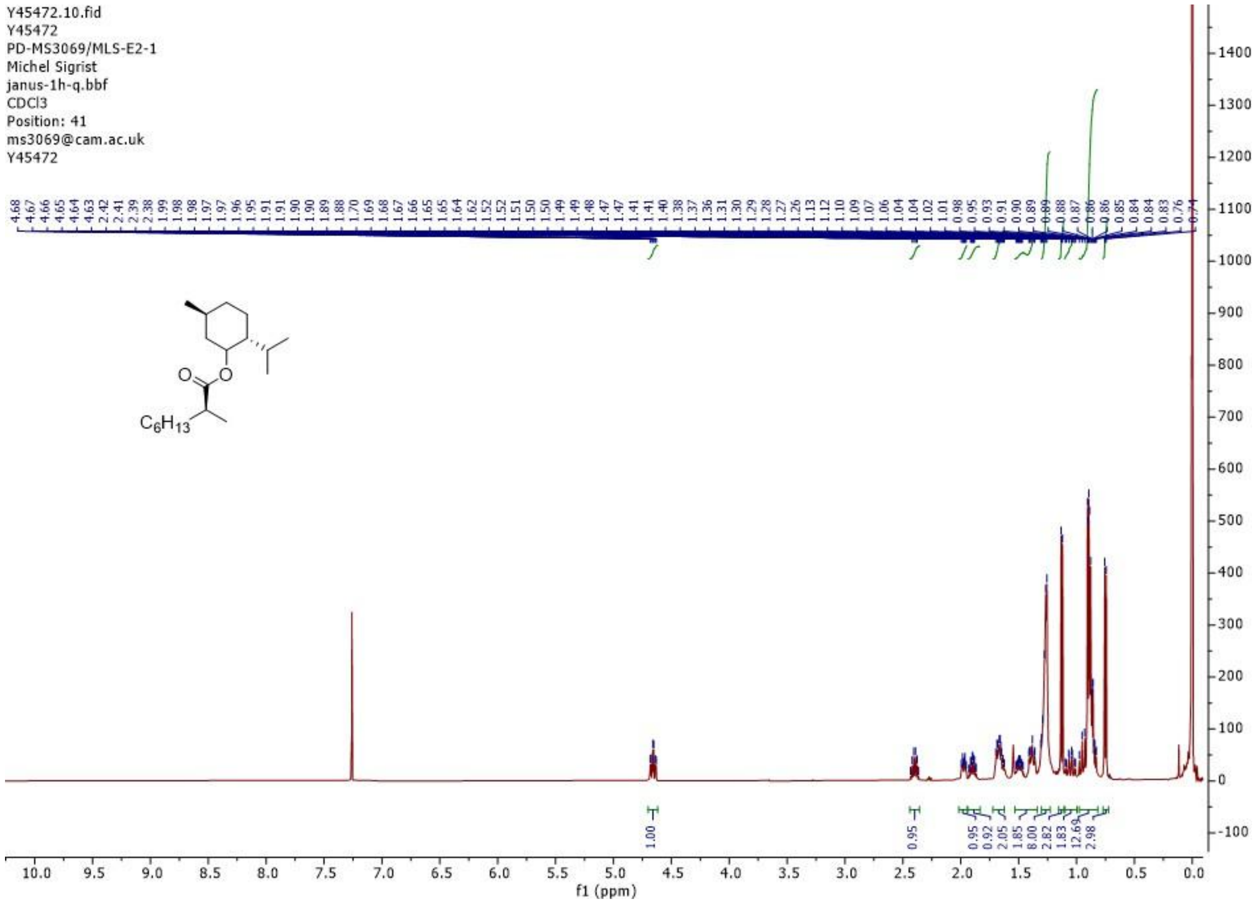
Y45472.10.fid
Y45472
PD-MS3069/MLS-E2-1
Michel Sigrist
janus-1h-q.bbf
CDCl3
Position: 41
ms3069@cam.ac.uk
Y45472
C_6H_{13}
f1 (ppm)

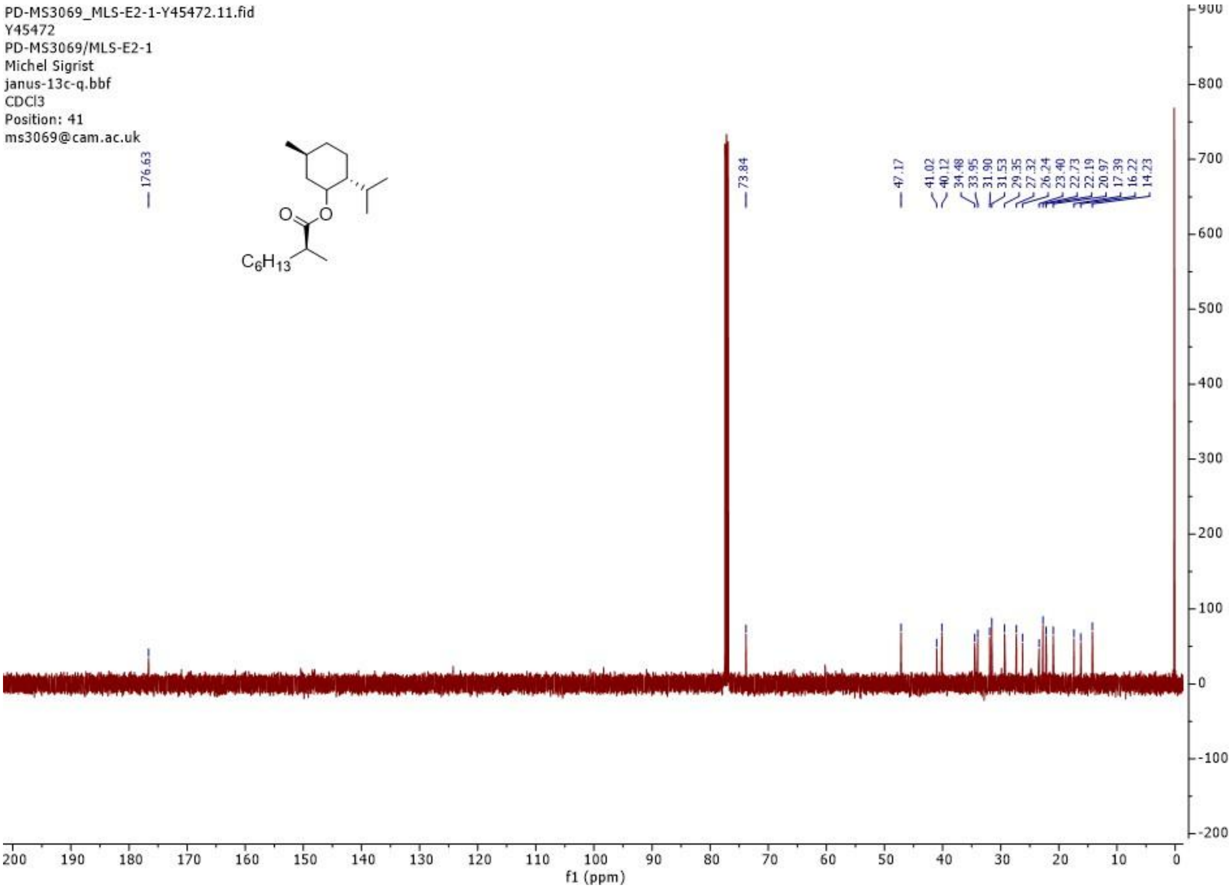
PD-MS3069_MLS-E2-1-Y45472.11.fid
Y45472
PD-MS3069/MLS-E2-1
Michel Sigrist
janus-13c-q.bbf
CDCl3
Position: 41
ms3069@cam.ac.uk
176.63
73.84
47.17
41.02
40.12
34.48
33.95
31.90
31.53
29.35
27.32
26.24
23.40
22.73
22.19
20.97
17.39
16.22
14.23
C_6H_{13}
f1 (ppm)

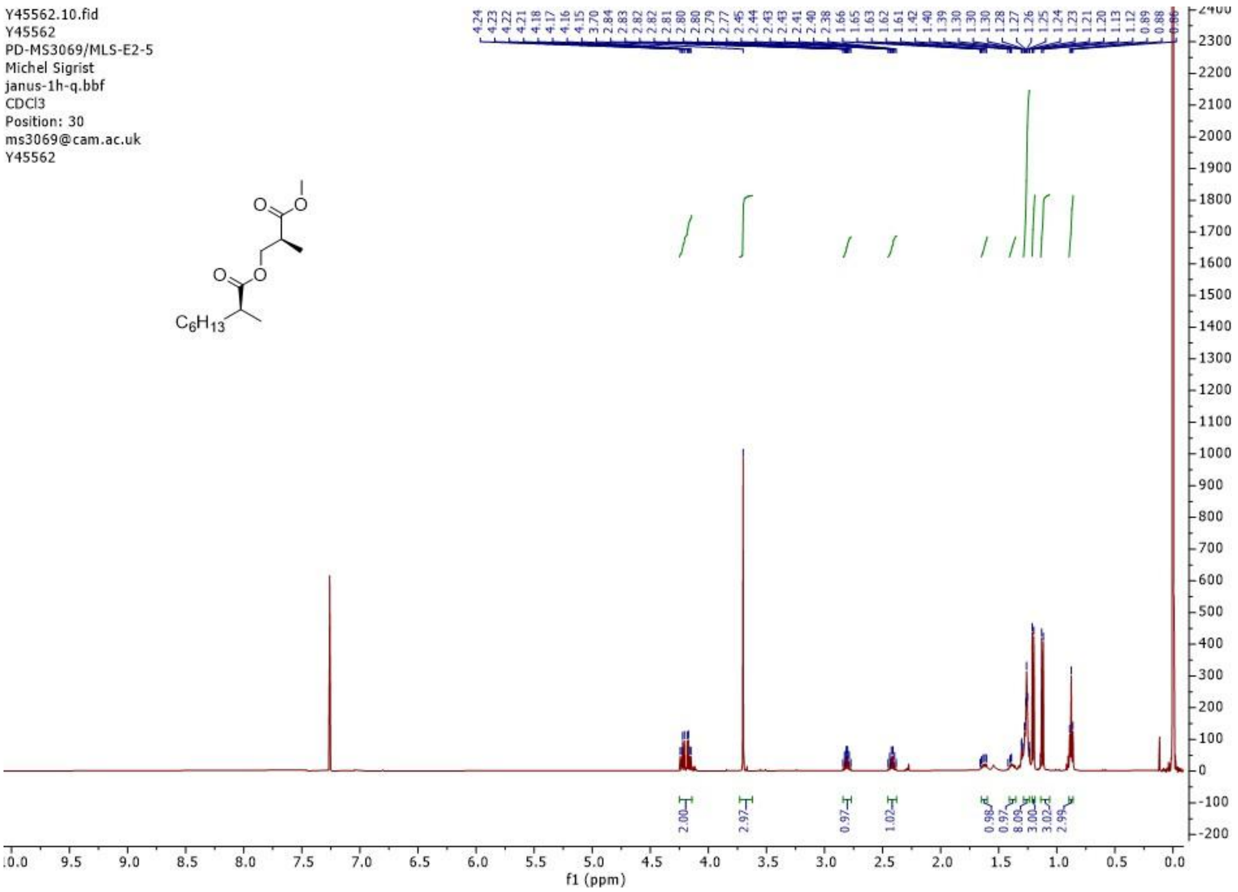
Y45562.10.fid
Y45562
PD-MS3069/MLS-E2-5
Michel Sigrist
janus-1h-q.bbf
CDCl3
Position: 30
ms3069@cam.ac.uk
Y45562
C_6H_{13}
4.24 4.23 4.22 4.21 4.18 4.17 4.16 4.15 3.70 2.84 2.83 2.82 2.82 2.81 2.80 2.80 2.79 2.77 2.45 2.44 2.43 2.43 2.41 2.40 2.38 1.66 1.65 1.63 1.62 1.61 1.42 1.40 1.39 1.30 1.30 1.30 1.28 1.27 1.26 1.25 1.24 1.23 1.21 1.20 1.13 1.12 0.89 0.88 0.86
2.00 2.97 0.97 1.02 0.98 0.97 8.09 3.00 3.02 2.99
f1 (ppm)

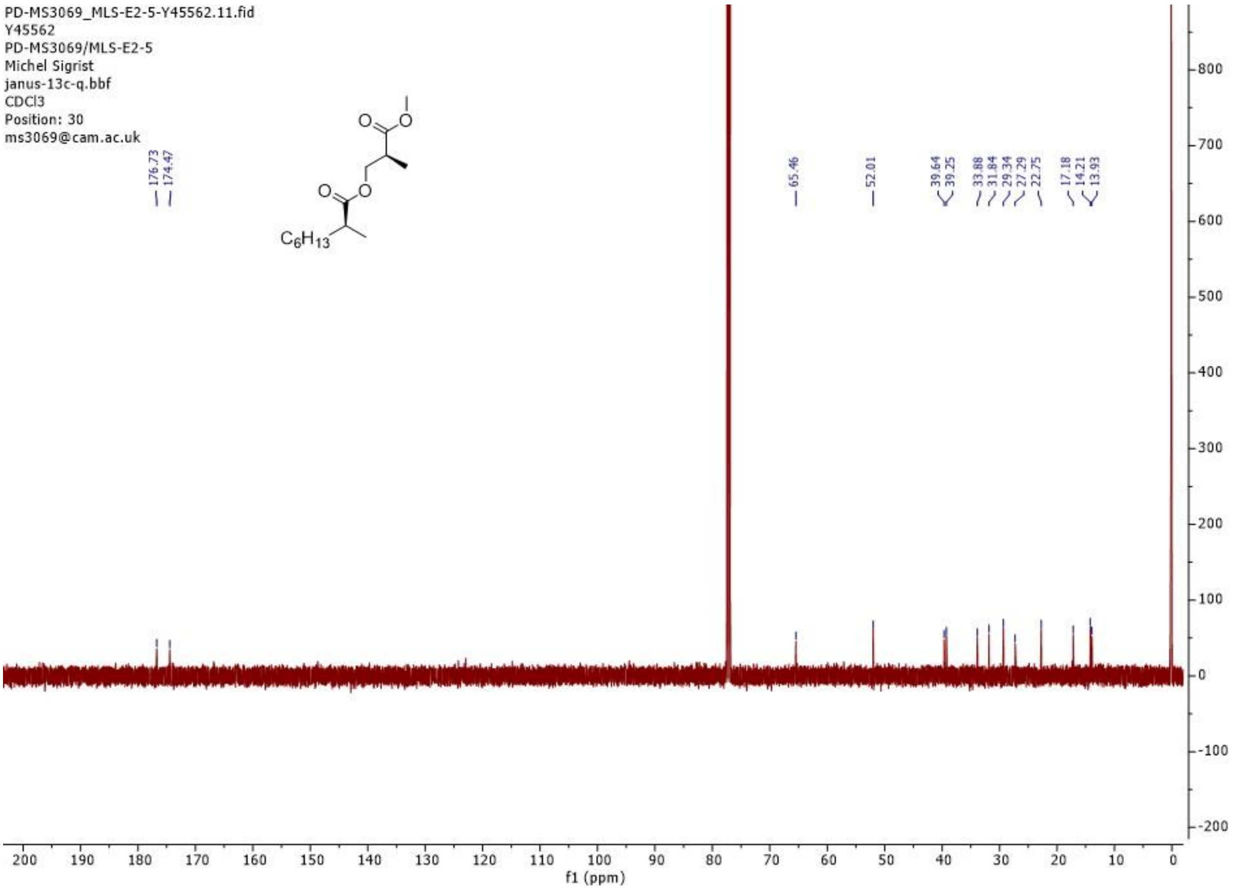
PD-MS3069_MLS-E2-5-Y45562.11.fid
Y45562
PD-MS3069/MLS-E2-5
Michel Sigrist
janus-13c-q.bbf
CDCl3
Position: 30
ms3069@cam.ac.uk
C_6H_{13}
176.73 174.47 65.46 52.01 39.64 39.25 33.88 31.84 29.34 27.29 22.75 17.18 14.21 13.93
f1 (ppm)

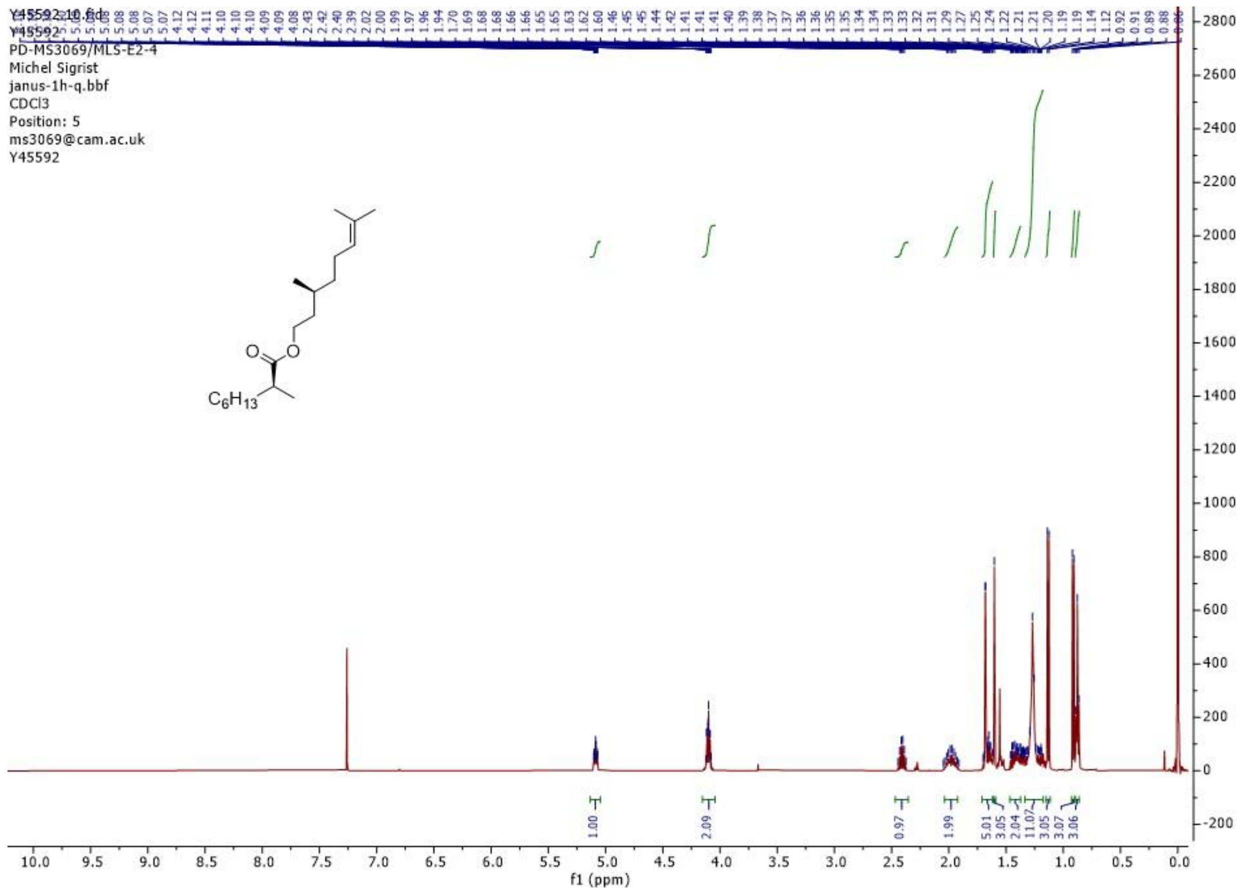
Y45592.10.fid
Y45592
PD-MS3069/MLS-E2-4
Michel Sigrist
janus-1h-q.bbf
CDCl3
Position: 5
ms3069@cam.ac.uk
Y45592
O
O
C_6H_{13}
1.00
2.09
0.97
1.99
5.01
3.05
2.04
11.07
3.05
3.07
3.06
f1 (ppm)

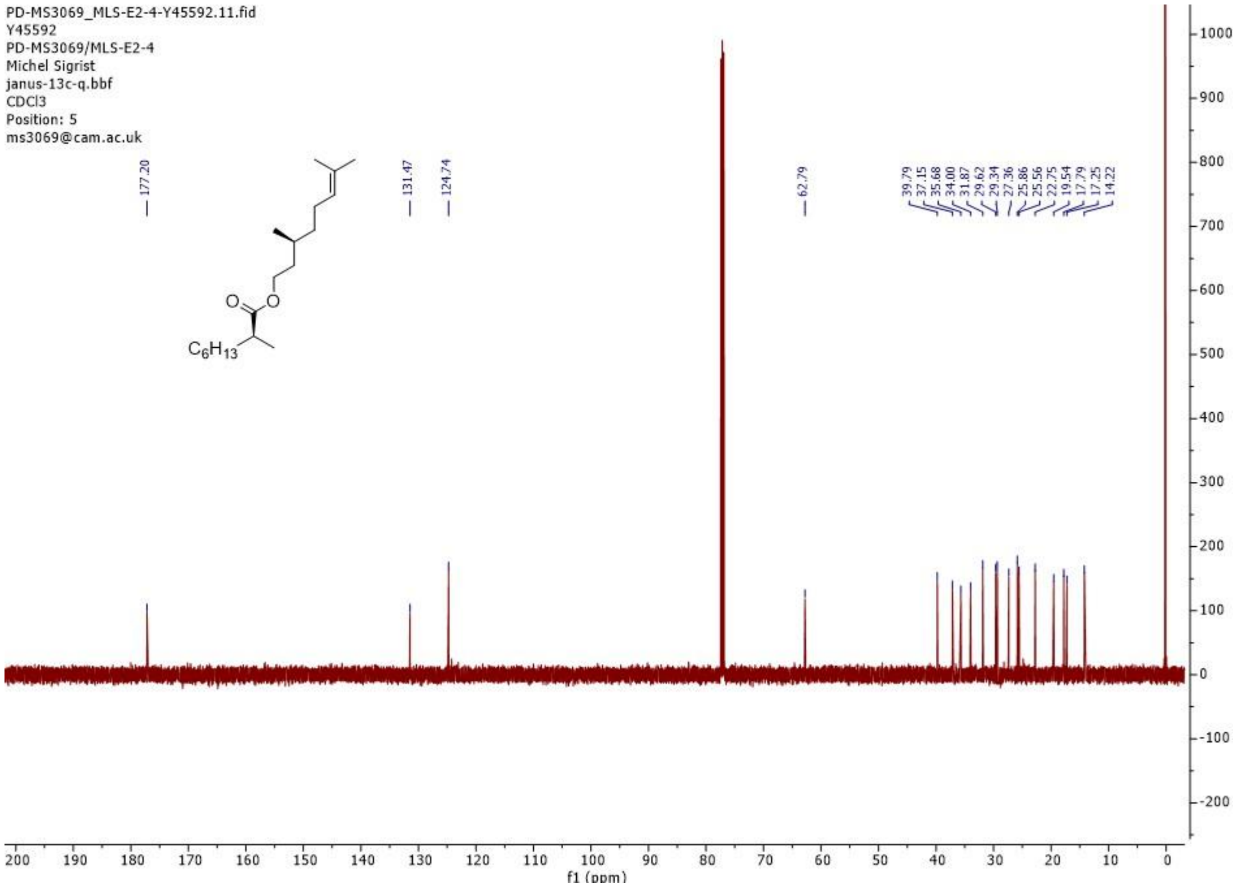
PD-MS3069_MLS-E2-4-Y45592.11.fid
Y45592
PD-MS3069/MLS-E2-4
Michel Sigrist
janus-13c-q.bbf
CDCl3
Position: 5
ms3069@cam.ac.uk
177.20
131.47
124.74
62.79
39.79
37.15
35.68
34.00
31.87
29.62
29.34
27.36
25.86
25.56
22.75
19.54
17.79
17.25
14.22
O
O
C_6H_{13}
f1 (ppm)

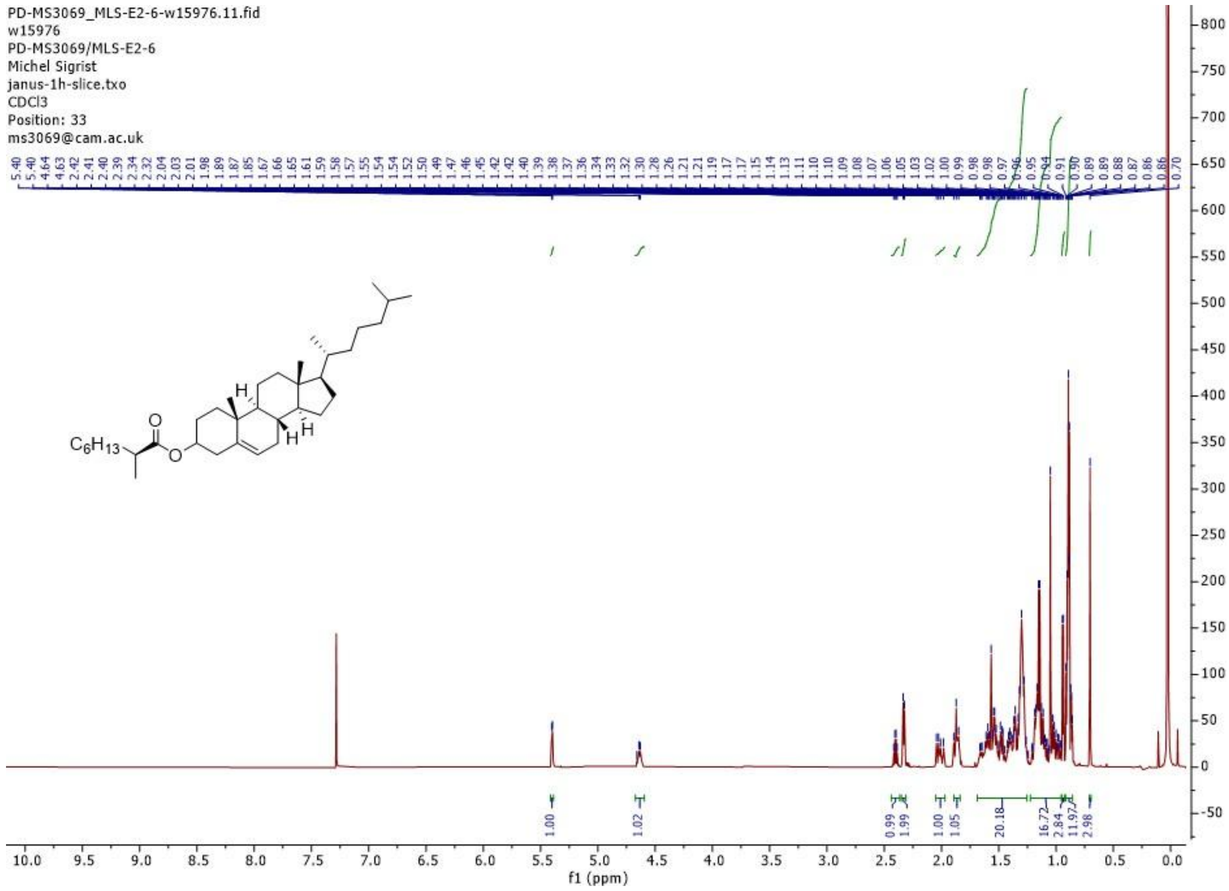
PD-MS3069_MLS-E2-6-w15976.11.fid
w15976
PD-MS3069/MLS-E2-6
Michel Sigrist
janus-1h-slice.txo
CDCl3
Position: 33
ms3069@cam.ac.uk
C_6H_{13}
f1 (ppm)

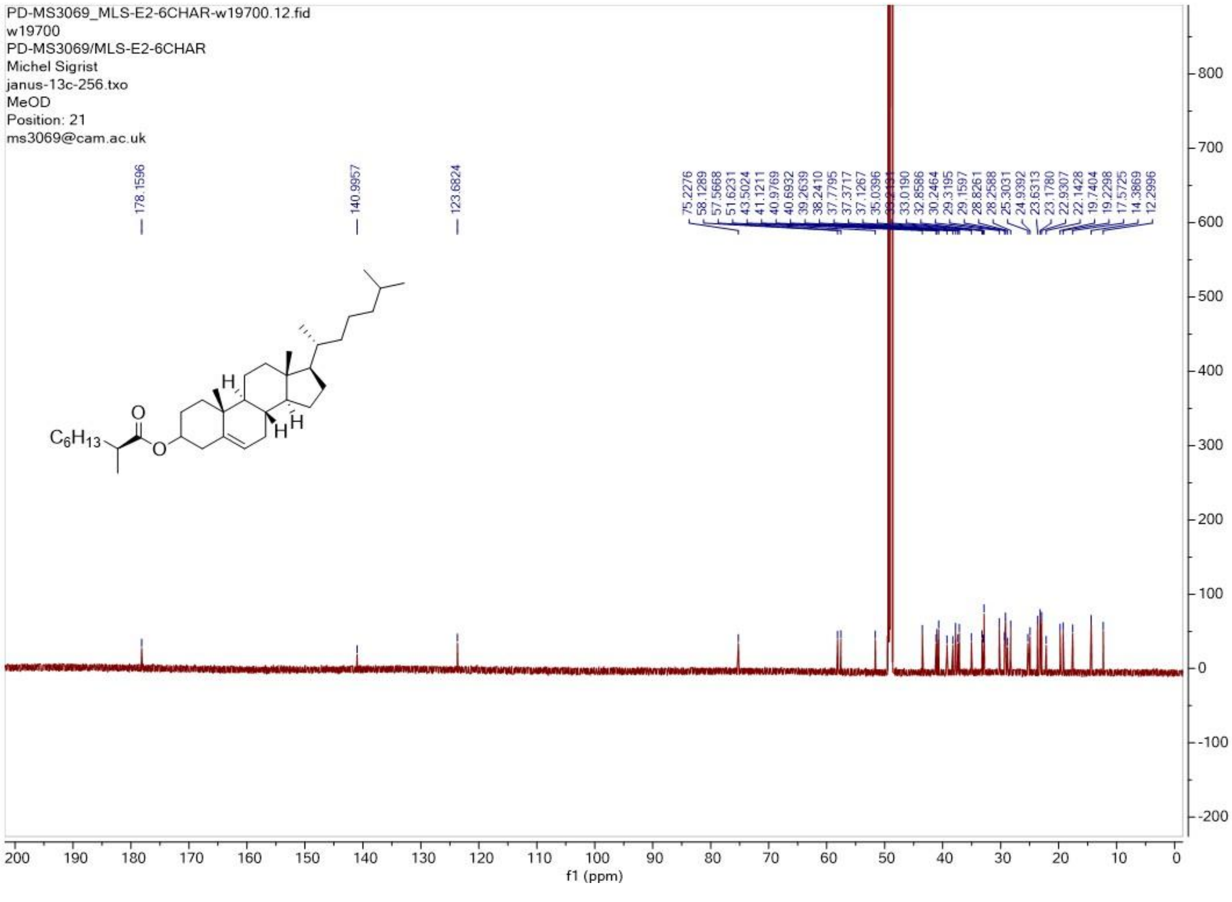
PD-MS3069_MLS-E2-6CHAR-w19700.12.fid
w19700
PD-MS3069/MLS-E2-6CHAR
Michel Sigrist
janus-13c-256.txo
MeOD
Position: 21
ms3069@cam.ac.uk
178.1596
140.9957
123.6824
C_6H_{13}
f1 (ppm)

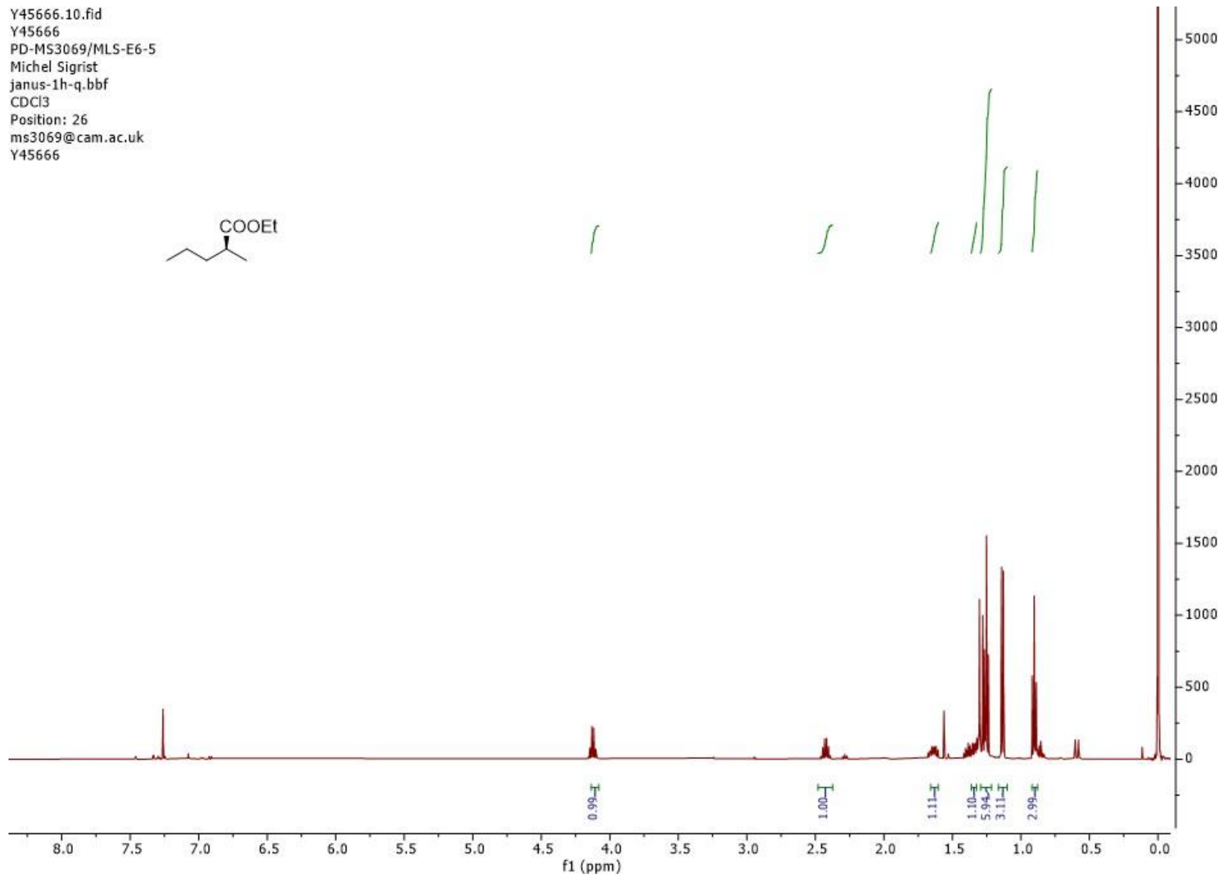
Y45666.10.fid
Y45666
PD-MS3069/MLS-E6-5
Michel Sigrist
janus-1h-q.bbf
CDCl3
Position: 26
ms3069@cam.ac.uk
Y45666
COOEt
0.99
1.00
1.11
1.10
5.94
3.11
2.99
f1 (ppm)

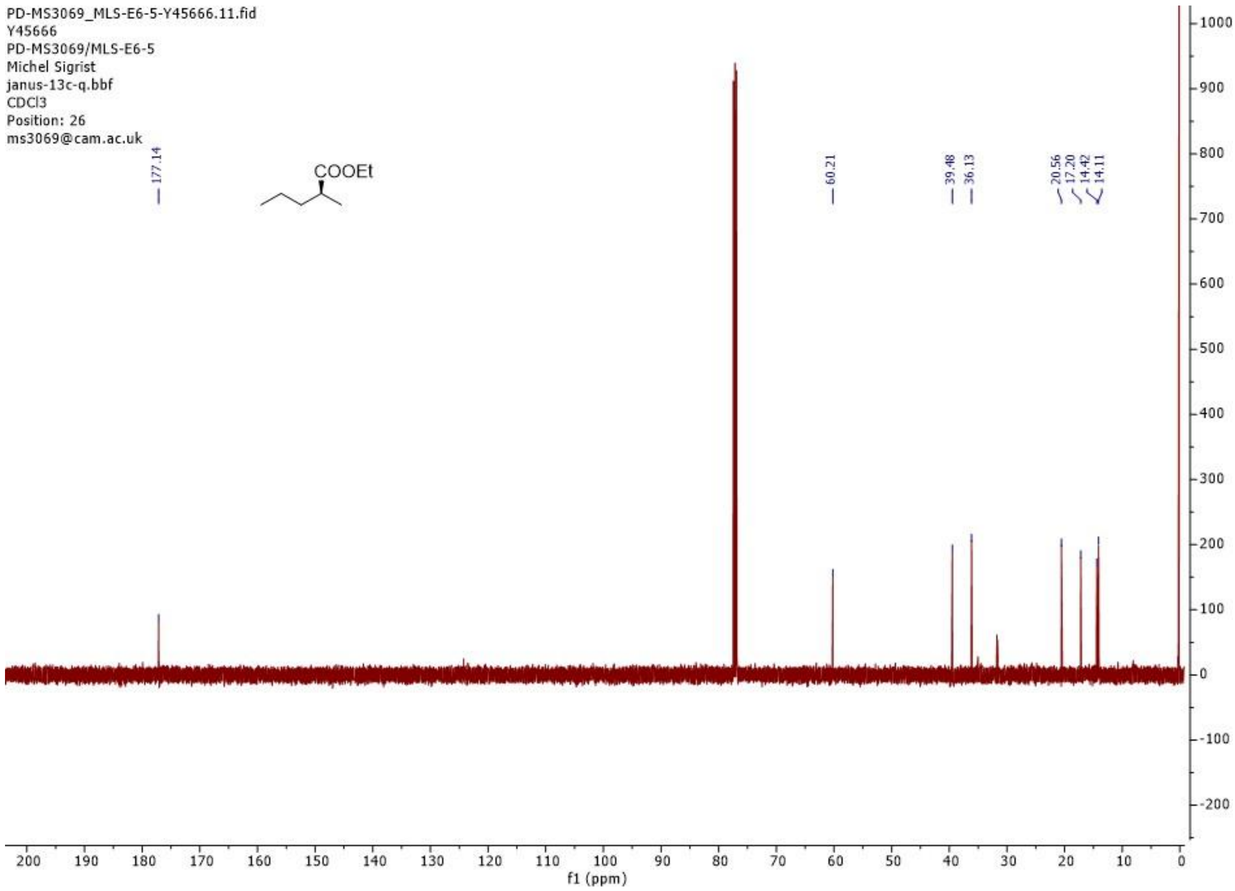
PD-MS3069_MLS-E6-5-Y45666.11.fid
Y45666
PD-MS3069/MLS-E6-5
Michel Sigrist
janus-13c-q.bbf
CDCl3
Position: 26
ms3069@cam.ac.uk
177.14
COOEt
60.21
39.48
36.13
20.56
17.20
14.42
14.11
f1 (ppm)

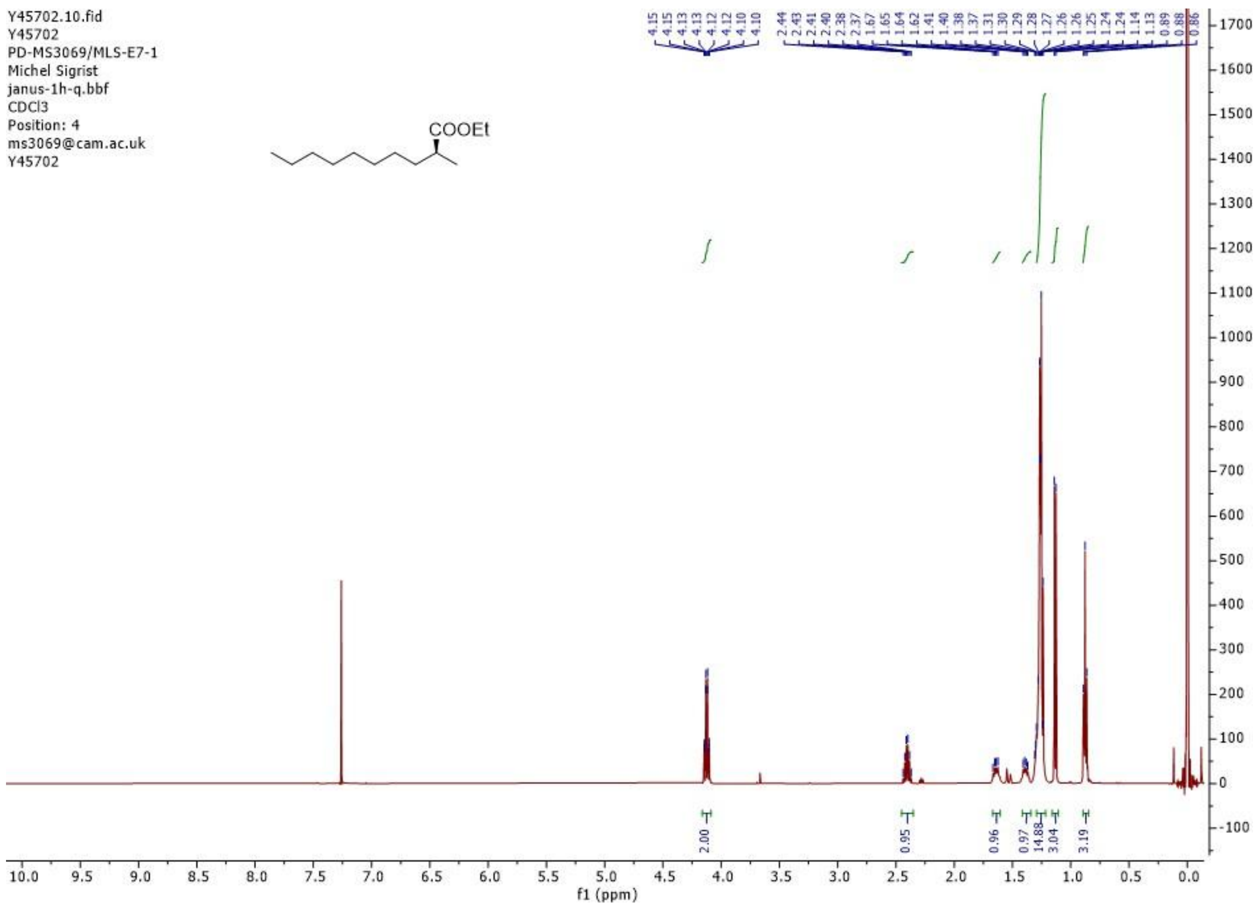
Y45702.10.fid
Y45702
PD-MS3069/MLS-E7-1
Michel Sigrist
janus-1h-q.bbf
CDCl3
Position: 4
ms3069@cam.ac.uk
Y45702
COOEt
4.15 4.15 4.13 4.13 4.12 4.12 4.10 4.10
2.44 2.43 2.41 2.40 2.38 2.37 1.67 1.65 1.64 1.62 1.41 1.40 1.38 1.37 1.31 1.30 1.29 1.28 1.27 1.26 1.26 1.25 1.24 1.24 1.14 1.13 0.89 0.88 0.86
2.00 0.95 0.96 0.97 14.88 3.04 3.19
f1 (ppm)

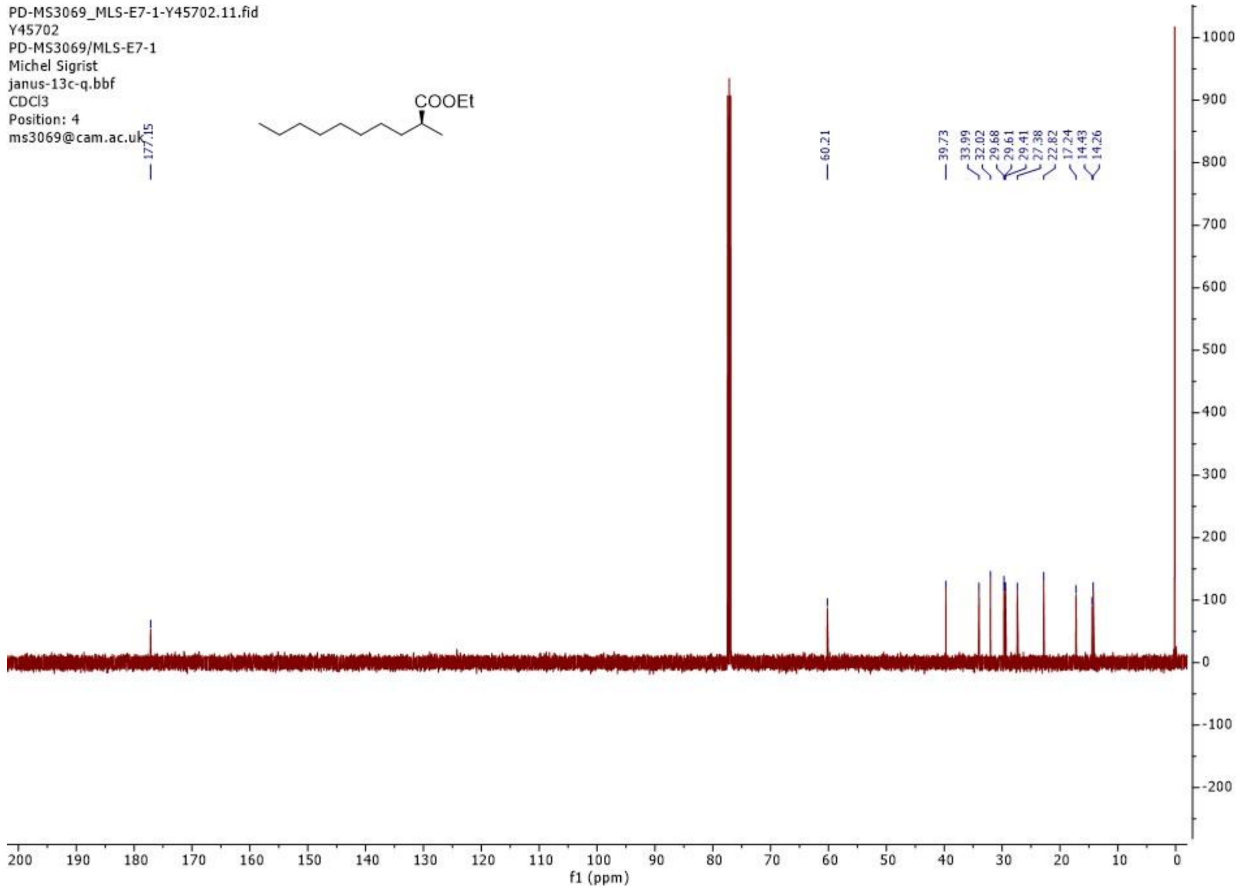
PD-MS3069_MLS-E7-1-Y45702.11.fid
Y45702
PD-MS3069/MLS-E7-1
Michel Sigrist
janus-13c-q.bbf
CDCl3
Position: 4
ms3069@cam.ac.uk
COOEt
177.15
60.21
39.73 33.99 32.02 29.68 29.61 29.41 27.38 22.82 17.24 14.43 14.26
f1 (ppm)

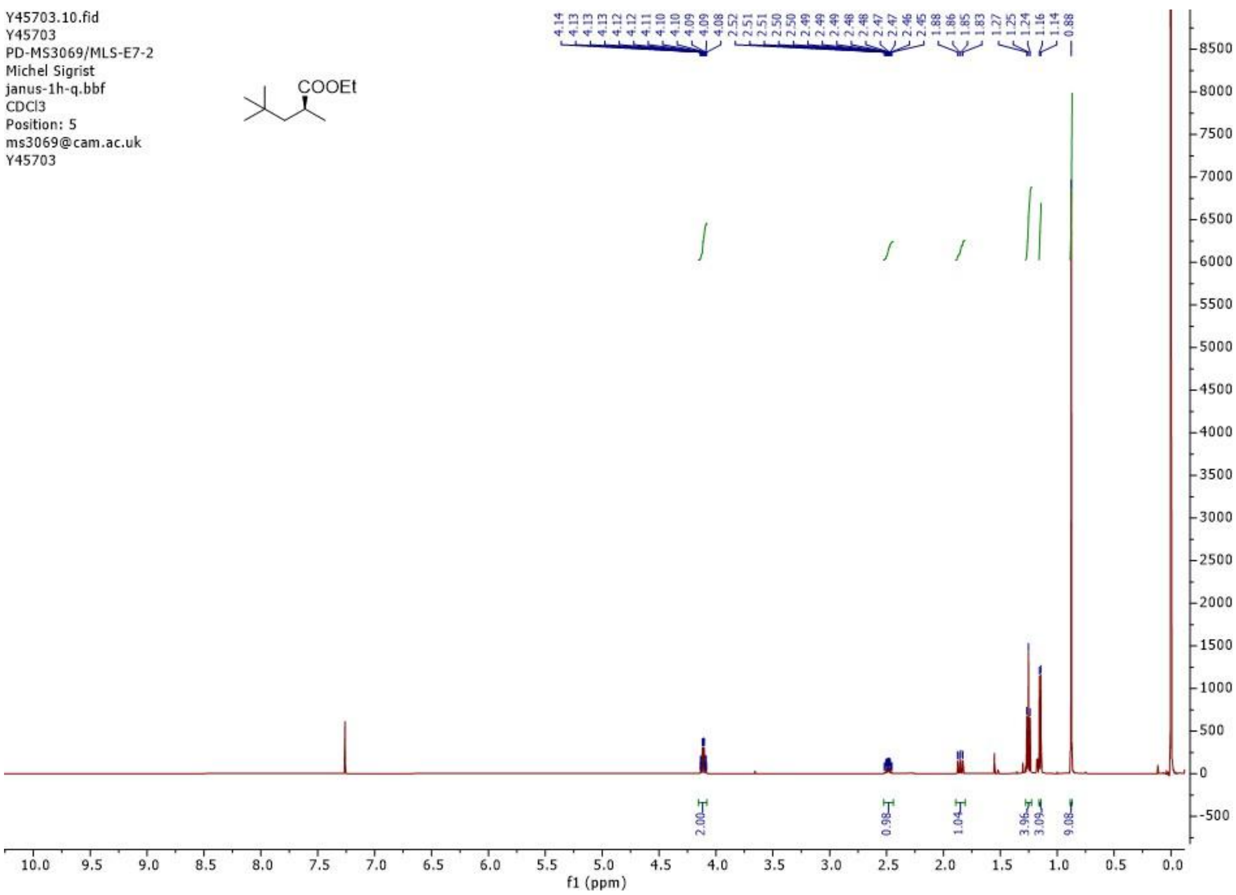
Y45703.10.fid
Y45703
PD-MS3069/MLS-E7-2
Michel Sigrist
janus-1h-q.bbf
CDCl3
Position: 5
ms3069@cam.ac.uk
Y45703
COOEt
4.14 4.13 4.13 4.12 4.12 4.11 4.10 4.10 4.09 4.09 4.08 2.52 2.51 2.51 2.50 2.50 2.49 2.49 2.49 2.48 2.48 2.47 2.47 2.46 2.45 1.88 1.86 1.85 1.83 1.27 1.25 1.24 1.16 1.14 0.88
2.00 0.98 1.04 3.96 3.09 9.08
f1 (ppm)

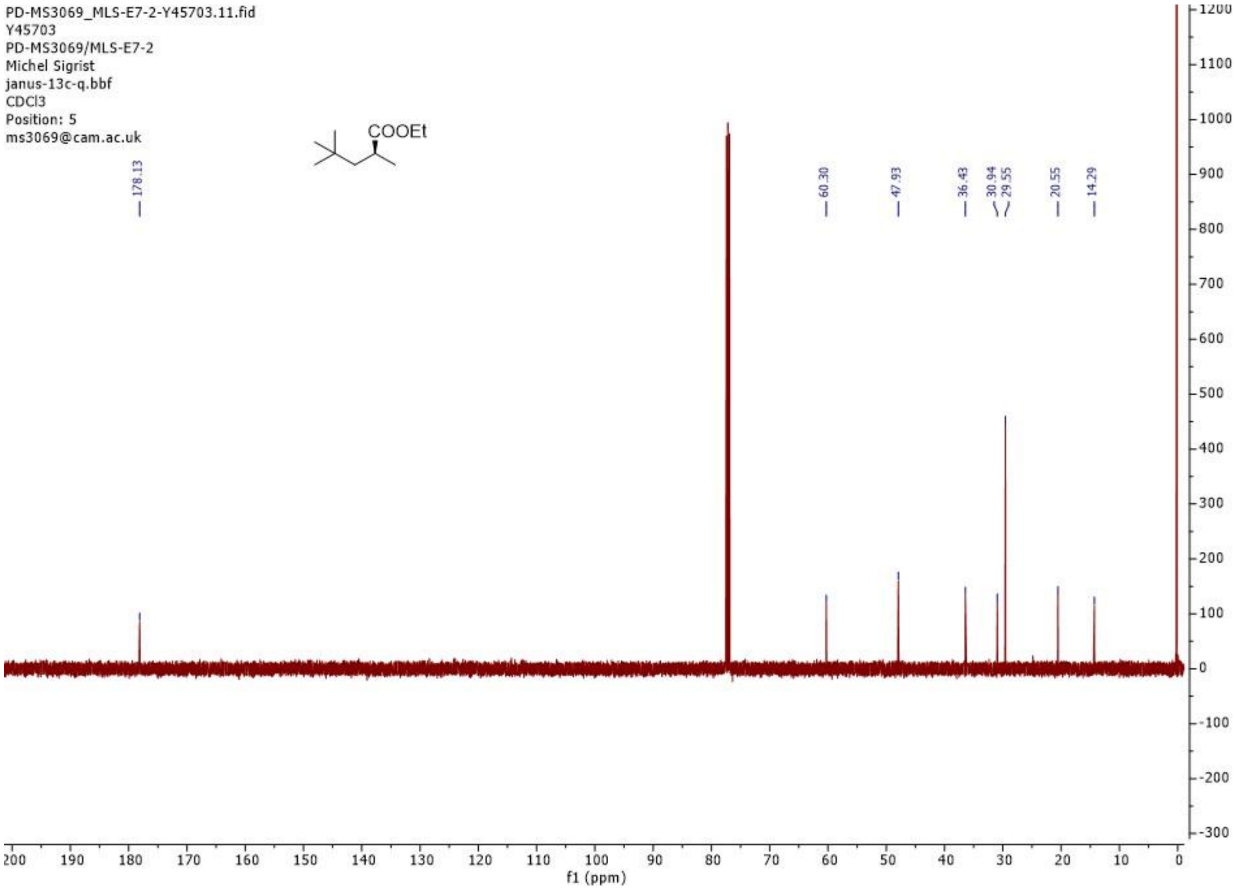
PD-MS3069_MLS-E7-2-Y45703.11.fid
Y45703
PD-MS3069/MLS-E7-2
Michel Sigrist
janus-13c-q.bbf
CDCl3
Position: 5
ms3069@cam.ac.uk
COOEt
178.13
60.30
47.93
36.43
30.94
29.55
20.55
14.29
f1 (ppm)

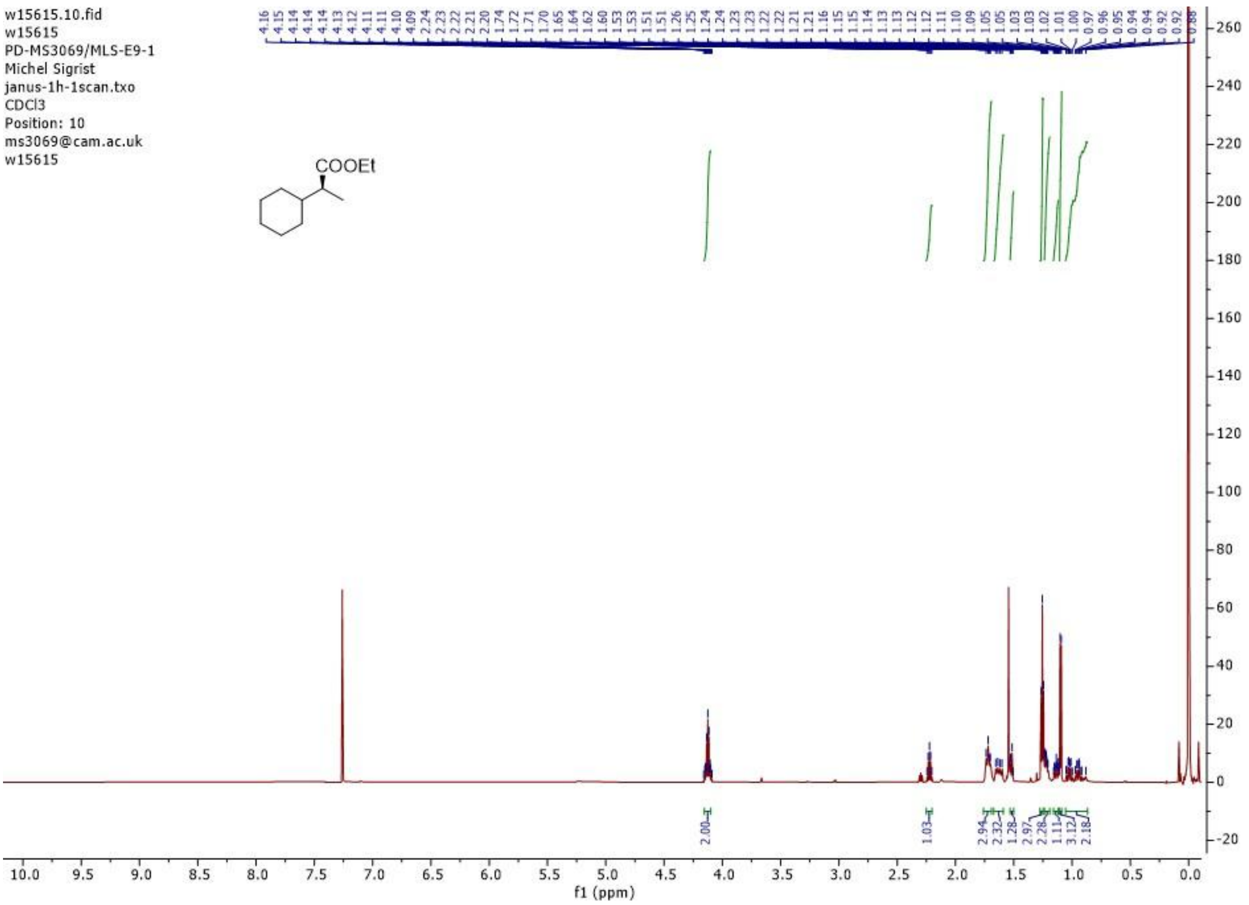
w15615.10.fid
w15615
PD-MS3069/MLS-E9-1
Michel Sigrist
janus-1h-1scan.txo
CDCl3
Position: 10
ms3069@cam.ac.uk
w15615
COOEt
4.16 4.15 4.14 4.14 4.14 4.13 4.12 4.11 4.11 4.10 4.09 2.24 2.23 2.22 2.21 2.20 1.74 1.72 1.71 1.70 1.65 1.64 1.62 1.60 1.53 1.53 1.51 1.51 1.26 1.25 1.24 1.24 1.23 1.23 1.22 1.22 1.21 1.21 1.16 1.15 1.15 1.14 1.13 1.13 1.13 1.12 1.12 1.11 1.10 1.09 1.05 1.05 1.03 1.03 1.02 1.01 1.00 0.97 0.96 0.95 0.94 0.94 0.92 0.92 0.88
2.00
1.03
2.94
2.32
1.28
2.97
2.28
1.11
3.12
2.18
f1 (ppm)

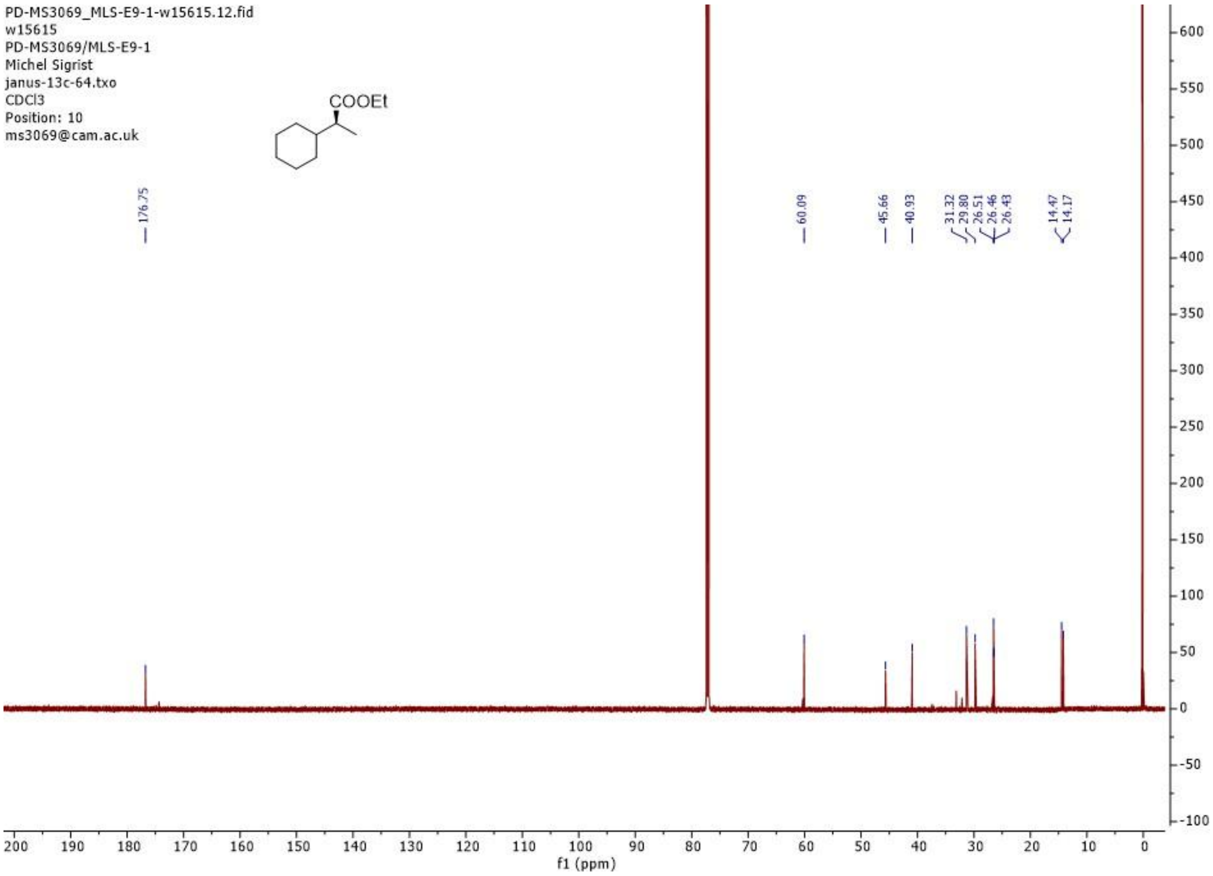
PD-MS3069_MLS-E9-1-w15615.12.fid
w15615
PD-MS3069/MLS-E9-1
Michel Sigrist
janus-13c-64.txo
CDCl3
Position: 10
ms3069@cam.ac.uk
COOEt
176.75
60.09
45.66
40.93
31.32
29.80
26.51
26.46
26.43
14.47
14.17
f1 (ppm)

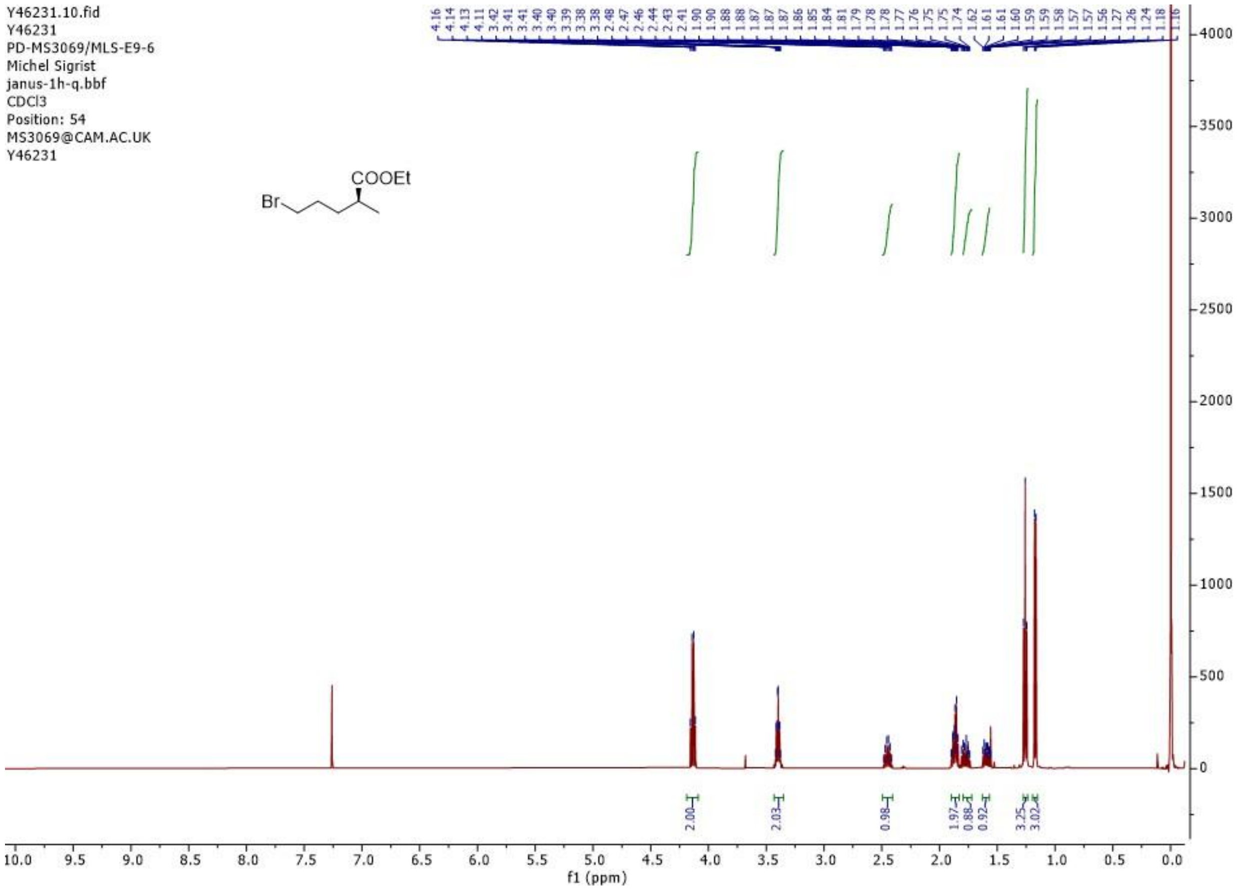
Y46231.10.fid
Y46231
PD-MS3069/MLS-E9-6
Michel Sigrist
janus-1h-q.bbf
CDCl3
Position: 54
MS3069@CAM.AC.UK
Y46231
COOEt
Br
4.16 4.14 4.13 4.11 3.42 3.41 3.41 3.40 3.40 3.39 3.38 3.38 2.48 2.47 2.46 2.44 2.43 2.41 1.90 1.88 1.88 1.87 1.87 1.86 1.85 1.84 1.81 1.79 1.78 1.78 1.77 1.76 1.75 1.75 1.74 1.62 1.61 1.61 1.60 1.59 1.59 1.58 1.57 1.57 1.56 1.27 1.26 1.24 1.18 1.16
2.00 2.03 0.98 1.97 0.88 0.92 3.25 3.02
f1 (ppm)

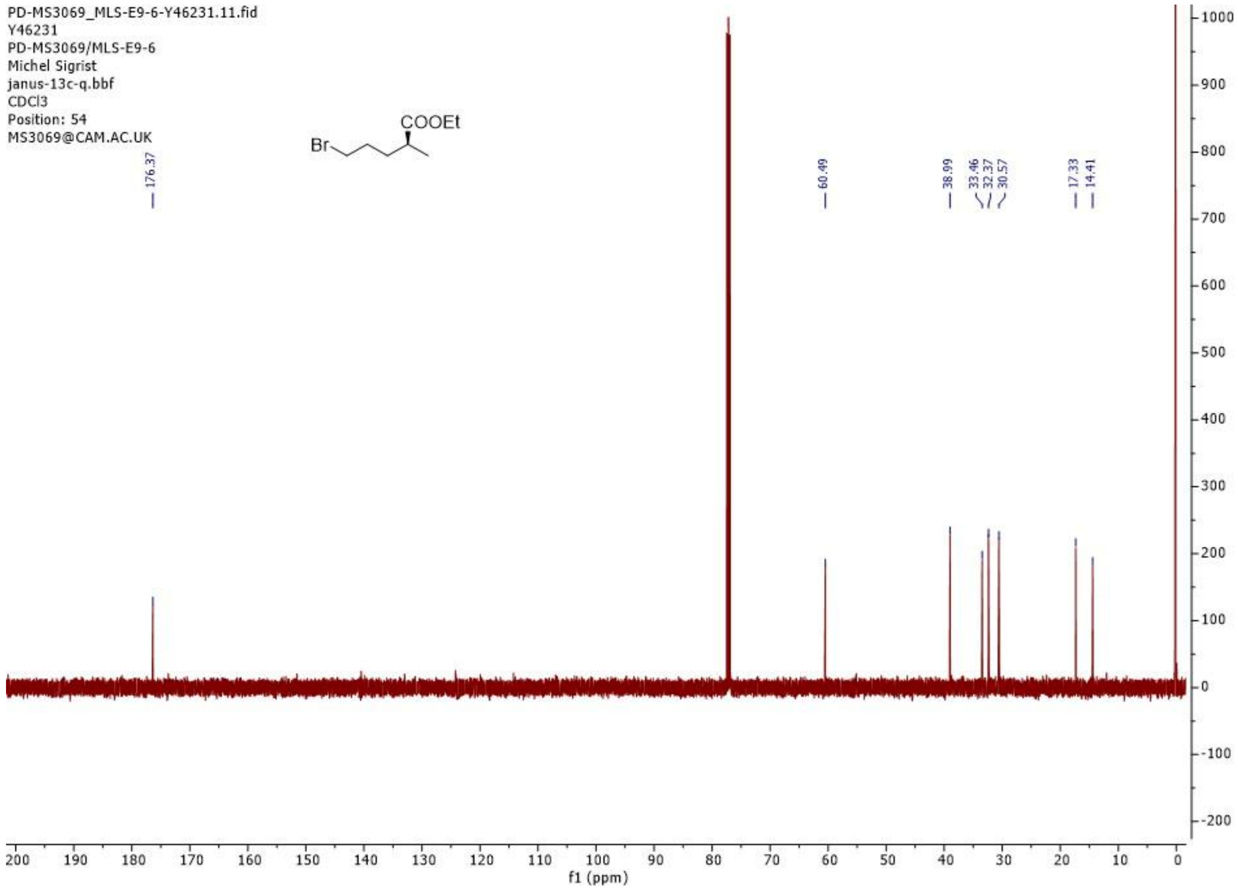
PD-MS3069_MLS-E9-6-Y46231.11.fid
Y46231
PD-MS3069/MLS-E9-6
Michel Sigrist
janus-13c-q.bbf
CDCl3
Position: 54
MS3069@CAM.AC.UK
COOEt
Br
176.37
60.49
38.99
33.46
32.37
30.57
17.33
14.41
f1 (ppm)

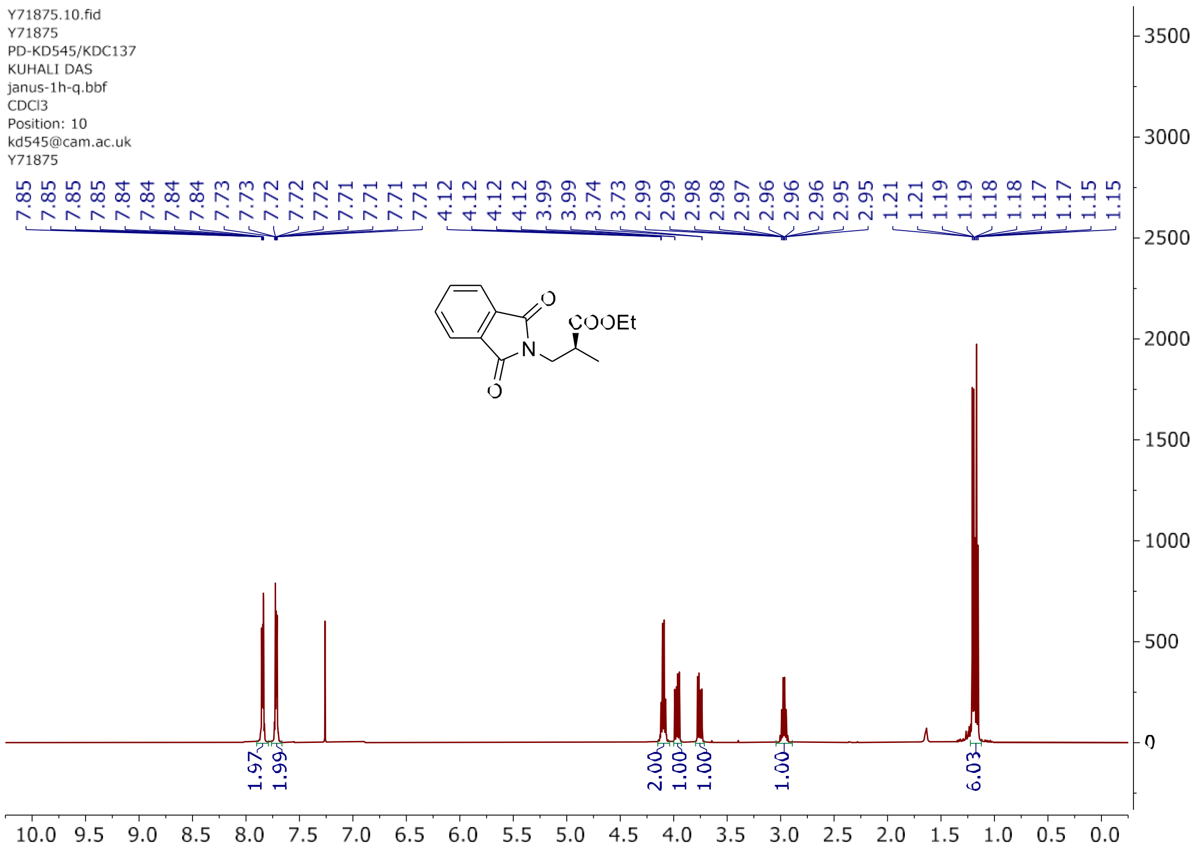
Y71875.10.fid
Y71875
PD-KD545/KDC137
KUHALI DAS
janus-1h-q.bbf
CDCl3
Position: 10
kd545@cam.ac.uk
Y71875
7.85 7.85 7.85 7.85 7.84 7.84 7.84 7.84 7.73 7.73 7.72 7.72 7.72 7.71 7.71 7.71 7.71 4.12 4.12 4.12 4.12 3.99 3.99 3.74 3.73 2.99 2.99 2.98 2.98 2.97 2.96 2.96 2.96 2.95 2.95 1.21 1.21 1.19 1.19 1.18 1.18 1.17 1.17 1.15 1.15
COOEt
N
O
1.97 1.99 2.00 1.00 1.00 1.00 6.03

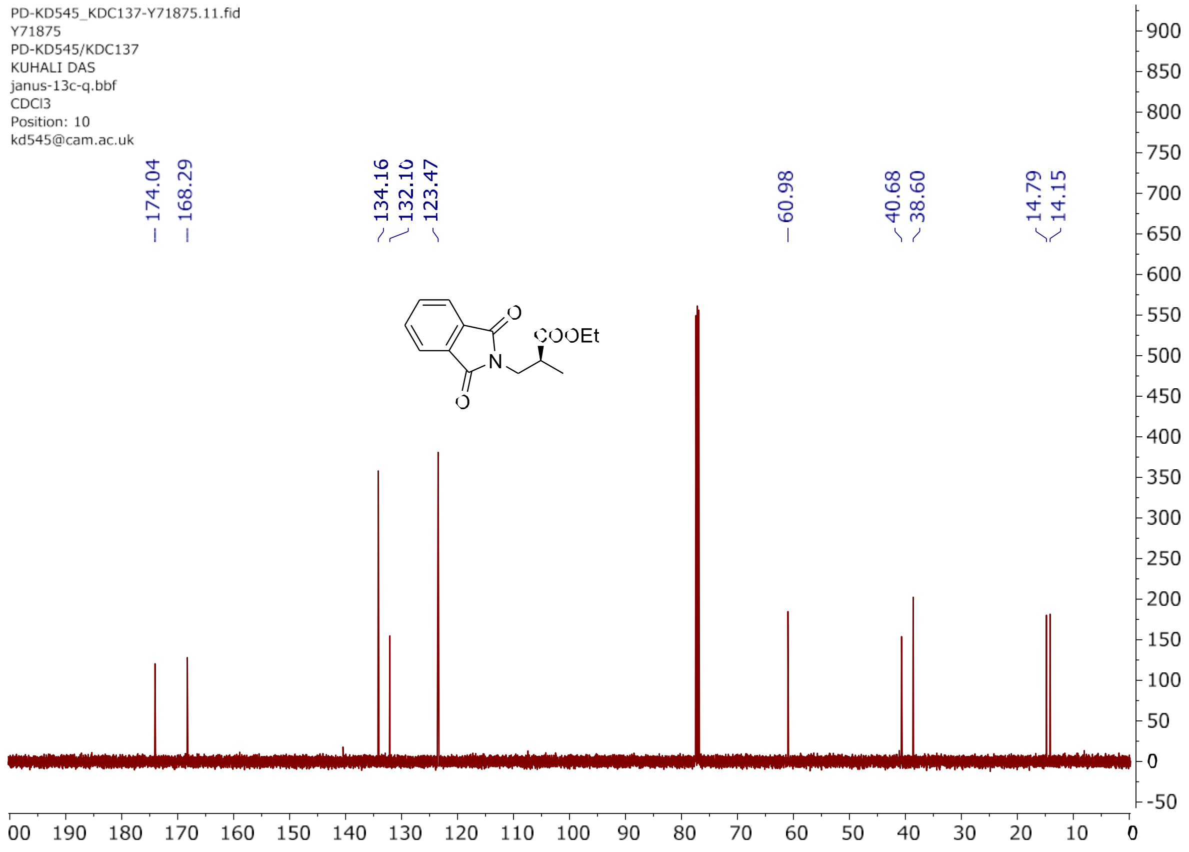
PD-KD545_KDC137-Y71875.11.fid
Y71875
PD-KD545/KDC137
KUHALI DAS
janus-13c-q.bbf
CDCl3
Position: 10
kd545@cam.ac.uk
174.04
168.29
134.16
132.10
123.47
60.98
40.68
38.60
14.79
14.15
COOEt
N
O

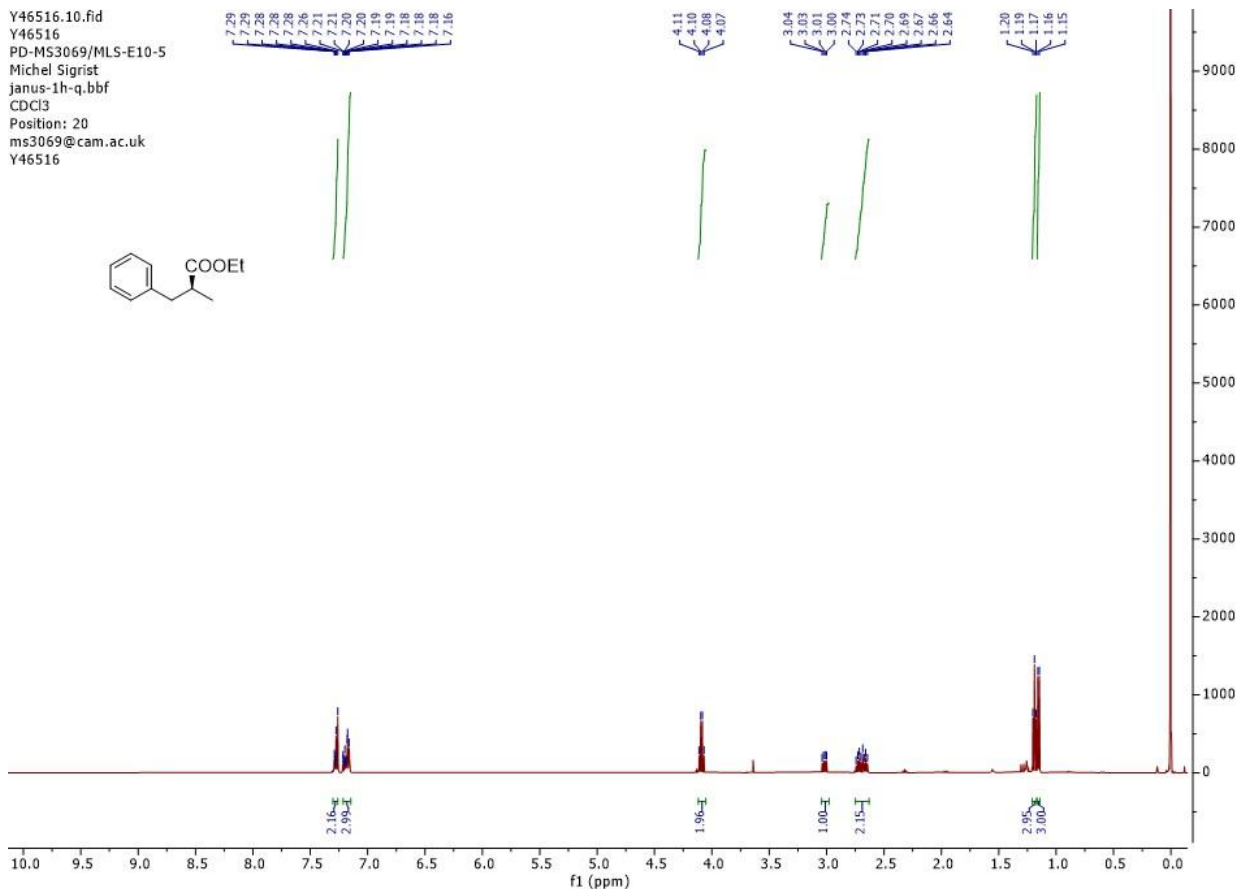
Y46516.10.fid
Y46516
PD-MS3069/MLS-E10-5
Michel Sigrist
janus-1h-q.bbf
CDCl3
Position: 20
ms3069@cam.ac.uk
Y46516
COOEt
f1 (ppm)

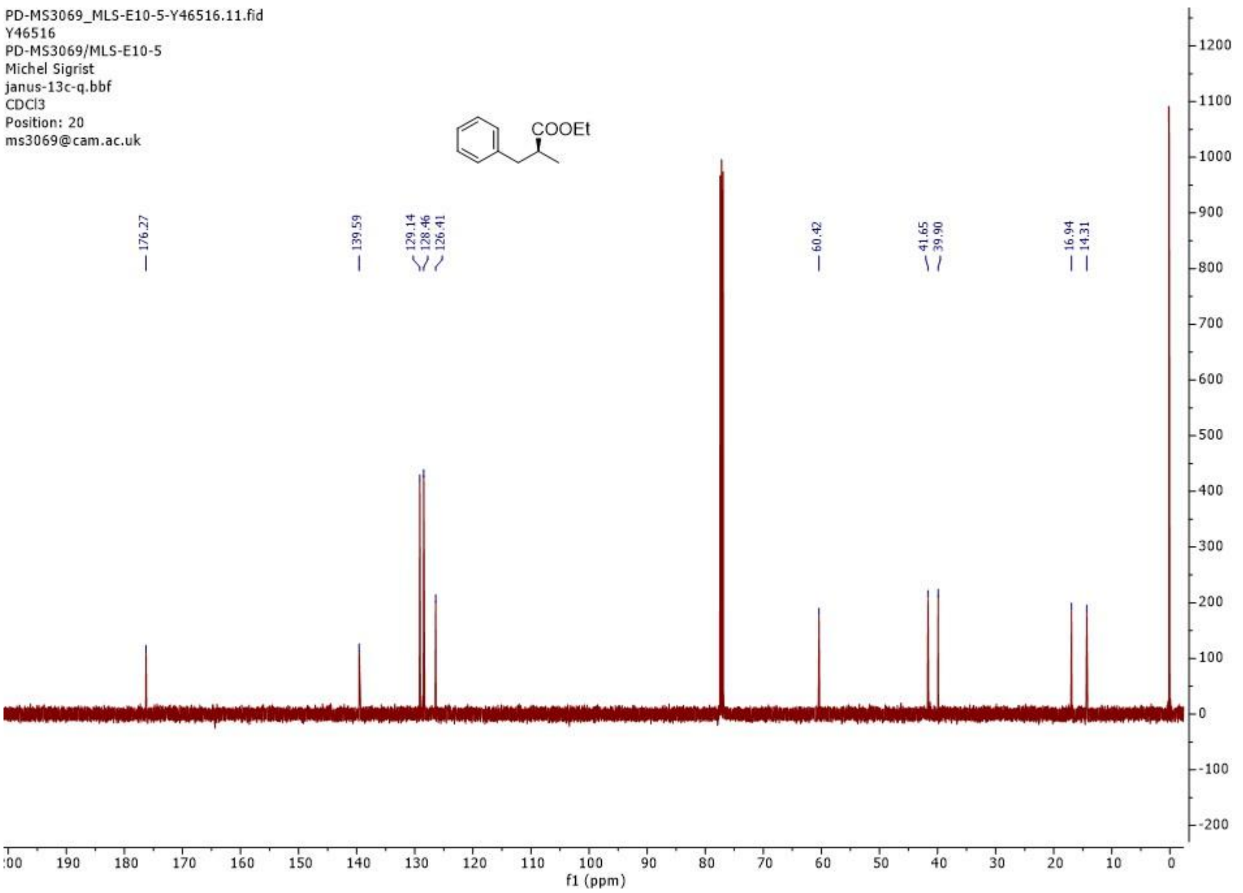
PD-MS3069_MLS-E10-5-Y46516.11.fid
Y46516
PD-MS3069/MLS-E10-5
Michel Sigrist
janus-13c-q.bbf
CDCl3
Position: 20
ms3069@cam.ac.uk
COOEt
176.27
139.59
129.14
128.46
126.41
60.42
41.65
39.90
16.94
14.31
f1 (ppm)

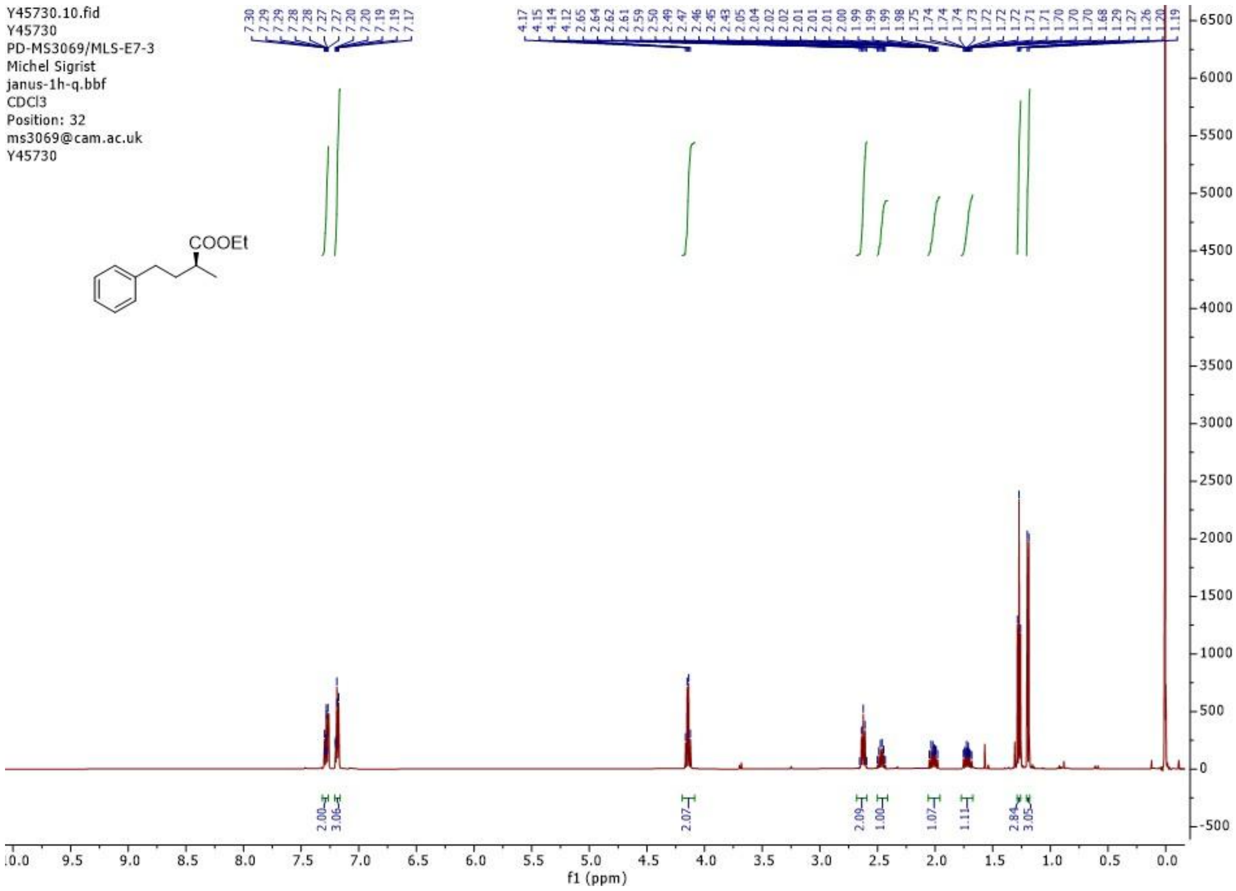
Y45730.10.fid
Y45730
PD-MS3069/MLS-E7-3
Michel Sigrist
janus-1h-q.bbf
CDCl3
Position: 32
ms3069@cam.ac.uk
Y45730
7.30 7.29 7.29 7.28 7.27 7.27 7.20 7.20 7.19 7.19 7.17
4.17 4.15 4.14 4.12 2.65 2.64 2.62 2.61 2.59 2.50 2.49 2.47 2.46 2.45 2.43 2.05 2.04 2.02 2.02 2.01 2.01 2.00 1.99 1.99 1.99 1.98 1.75 1.74 1.74 1.74 1.73 1.72 1.72 1.72 1.71 1.71 1.70 1.70 1.68 1.27 1.26 1.20 1.19
COOEt
2.00 3.06 2.07 2.09 1.00 1.07 1.11 2.84 3.05
f1 (ppm)

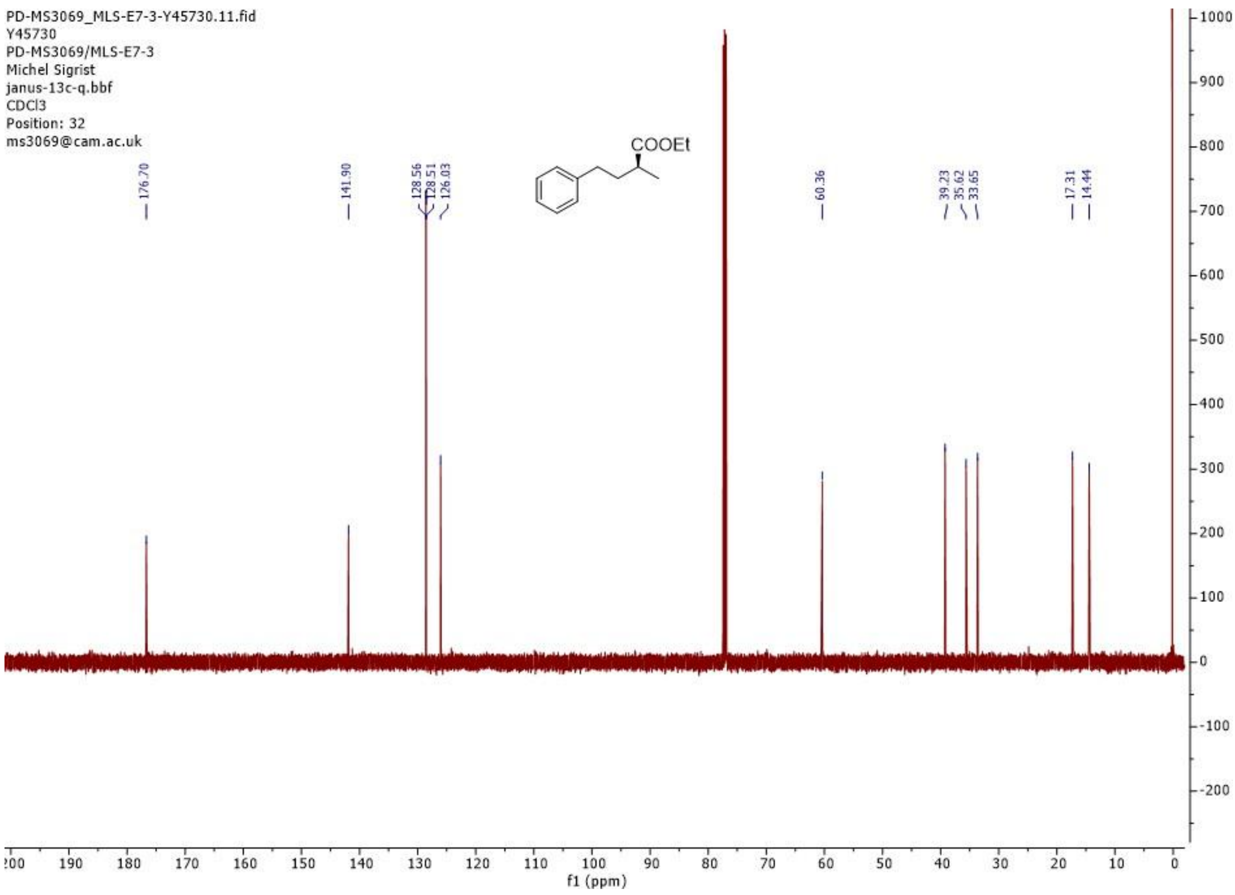
PD-MS3069_MLS-E7-3-Y45730.11.fid
Y45730
PD-MS3069/MLS-E7-3
Michel Sigrist
janus-13c-q.bbf
CDCl3
Position: 32
ms3069@cam.ac.uk
176.70
141.90
128.56 128.51 126.03
COOEt
60.36
39.23 35.62 33.65
17.31 14.44
f1 (ppm)

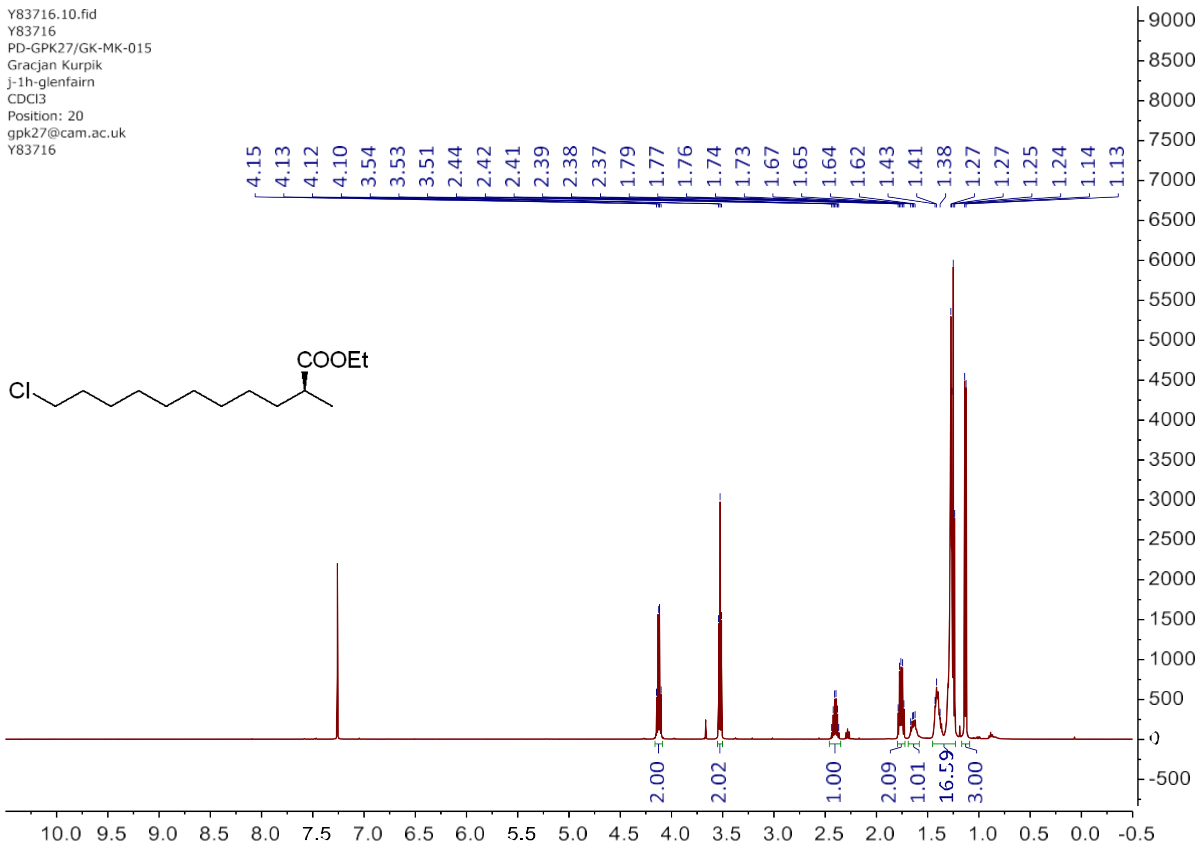

Y83716.10.fid
Y83716
PD-GPK27/GK-MK-015
Gracjan Kurpik
j-1h-glenfairn
CDCl3
Position: 20
gpk27@cam.ac.uk
Y83716
4.15 4.13 4.12 4.10 3.54 3.53 3.51 2.44 2.42 2.41 2.39 2.38 2.37 1.79 1.77 1.76 1.74 1.73 1.67 1.65 1.64 1.62 1.43 1.41 1.38 1.27 1.27 1.25 1.24 1.14 1.13
COOEt
Cl
2.00 2.02 1.00 2.09 1.01 16.59 3.00

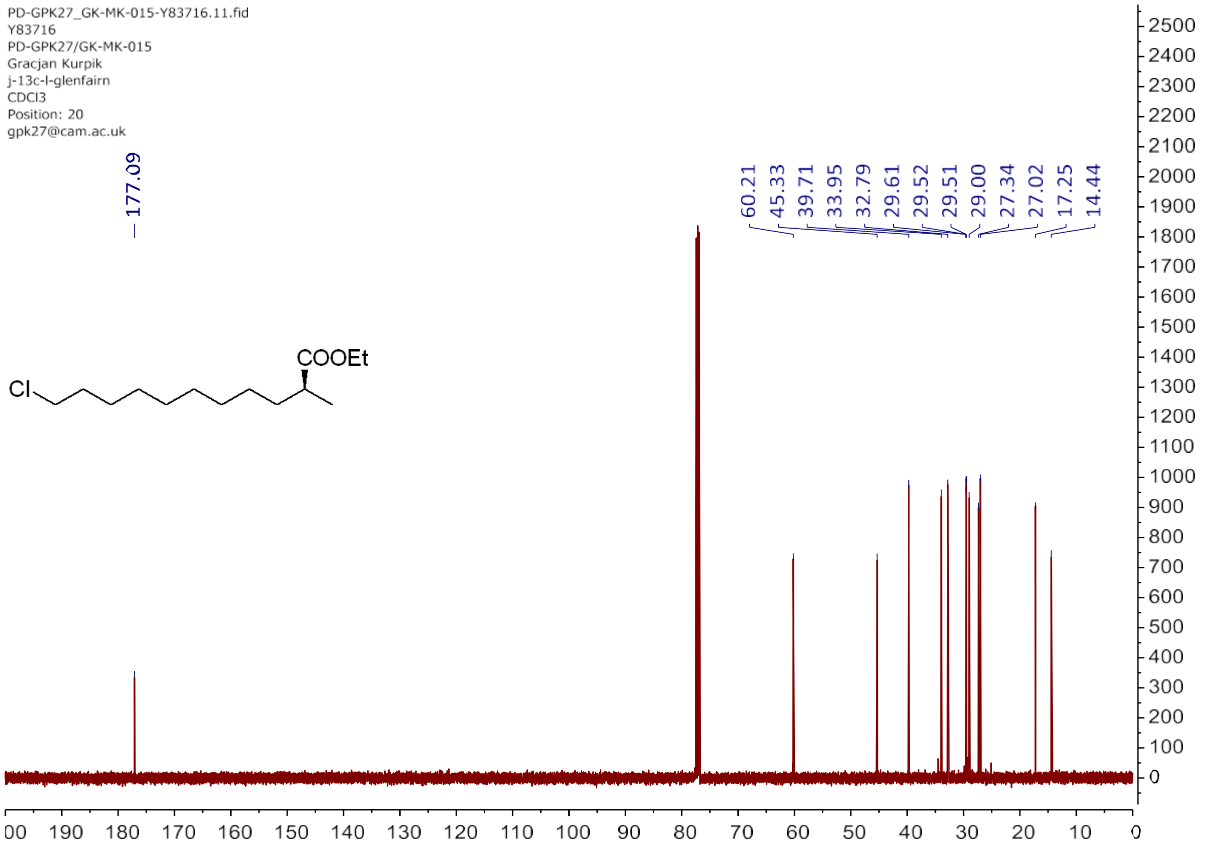

PD-GPK27_GK-MK-015-Y83716.11.fid
Y83716
PD-GPK27/GK-MK-015
Gracjan Kurpik
j-13c-l-glenfairn
CDCl3
Position: 20
gpk27@cam.ac.uk
177.09
60.21 45.33 39.71 33.95 32.79 29.61 29.52 29.51 29.00 27.34 27.02 17.25 14.44
COOEt
Cl

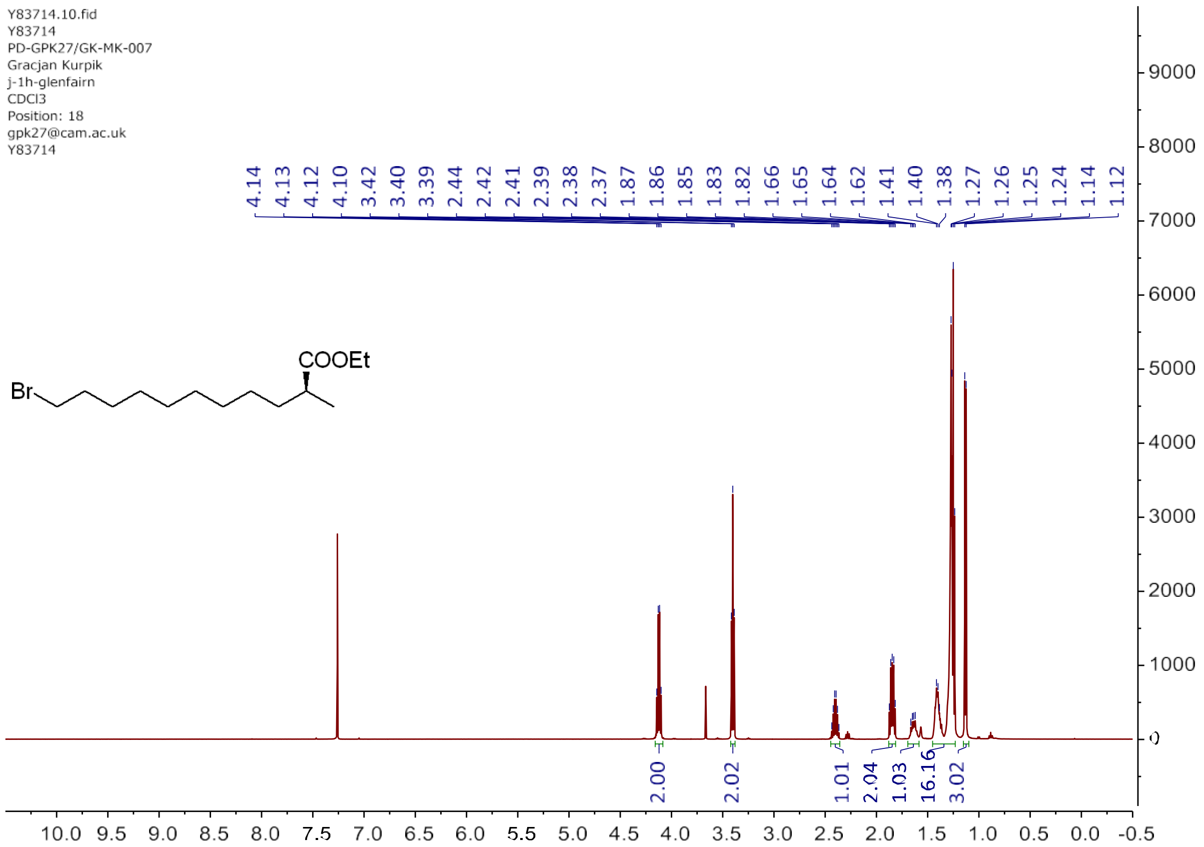
Y83714.10.fid
Y83714
PD-GPK27/GK-MK-007
Gracjan Kurpik
j-1h-glenfairn
CDCl3
Position: 18
gpk27@cam.ac.uk
Y83714
4.14 4.13 4.12 4.10 3.42 3.40 3.39 2.44 2.42 2.41 2.39 2.38 2.37 1.87 1.86 1.85 1.83 1.82 1.66 1.65 1.64 1.62 1.41 1.40 1.38 1.27 1.26 1.25 1.24 1.14 1.12
COOEt
Br
2.00 2.02 1.01 2.04 1.03 16.16 3.02

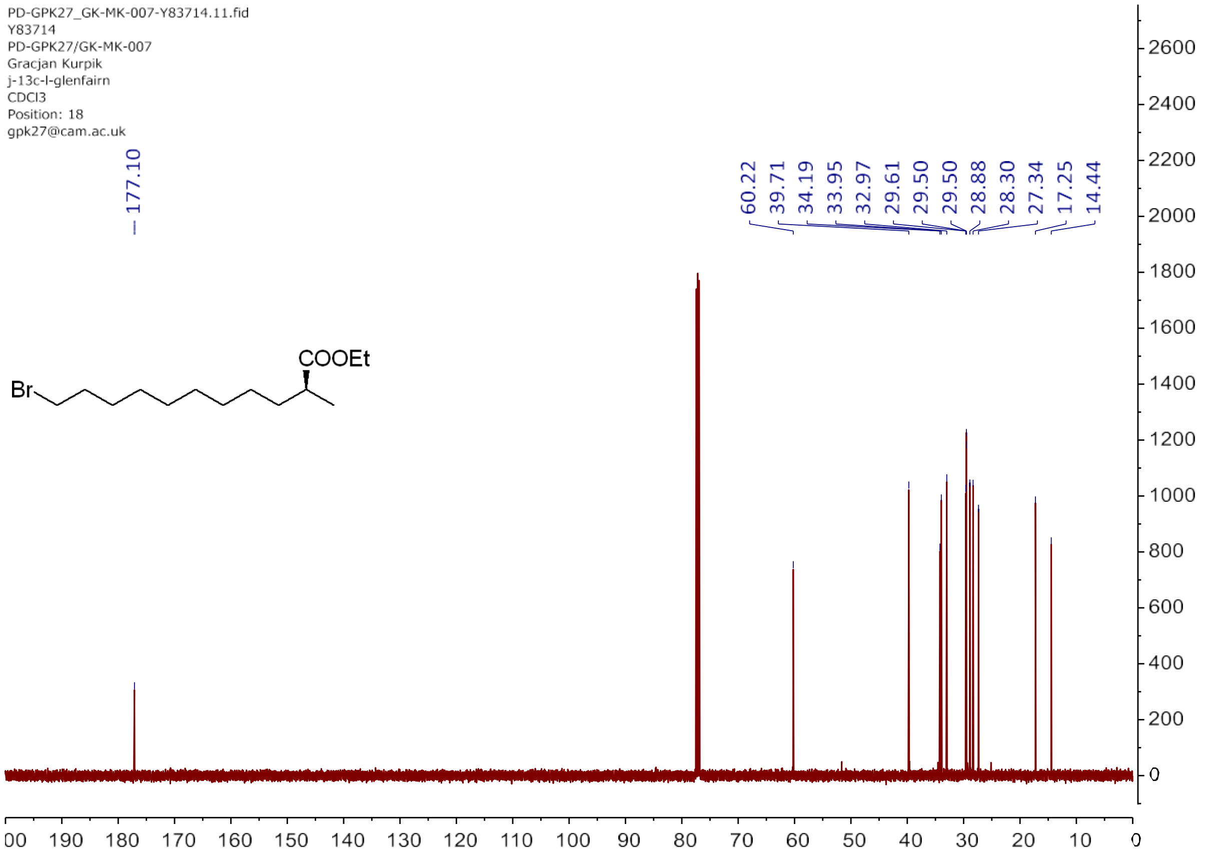
PD-GPK27_GK-MK-007-Y83714.11.fid
Y83714
PD-GPK27/GK-MK-007
Gracjan Kurpik
j-13c-l-glenfairn
CDCl3
Position: 18
gpk27@cam.ac.uk
177.10
60.22 39.71 34.19 33.95 32.97 29.61 29.50 29.50 28.88 28.30 27.34 17.25 14.44
COOEt
Br

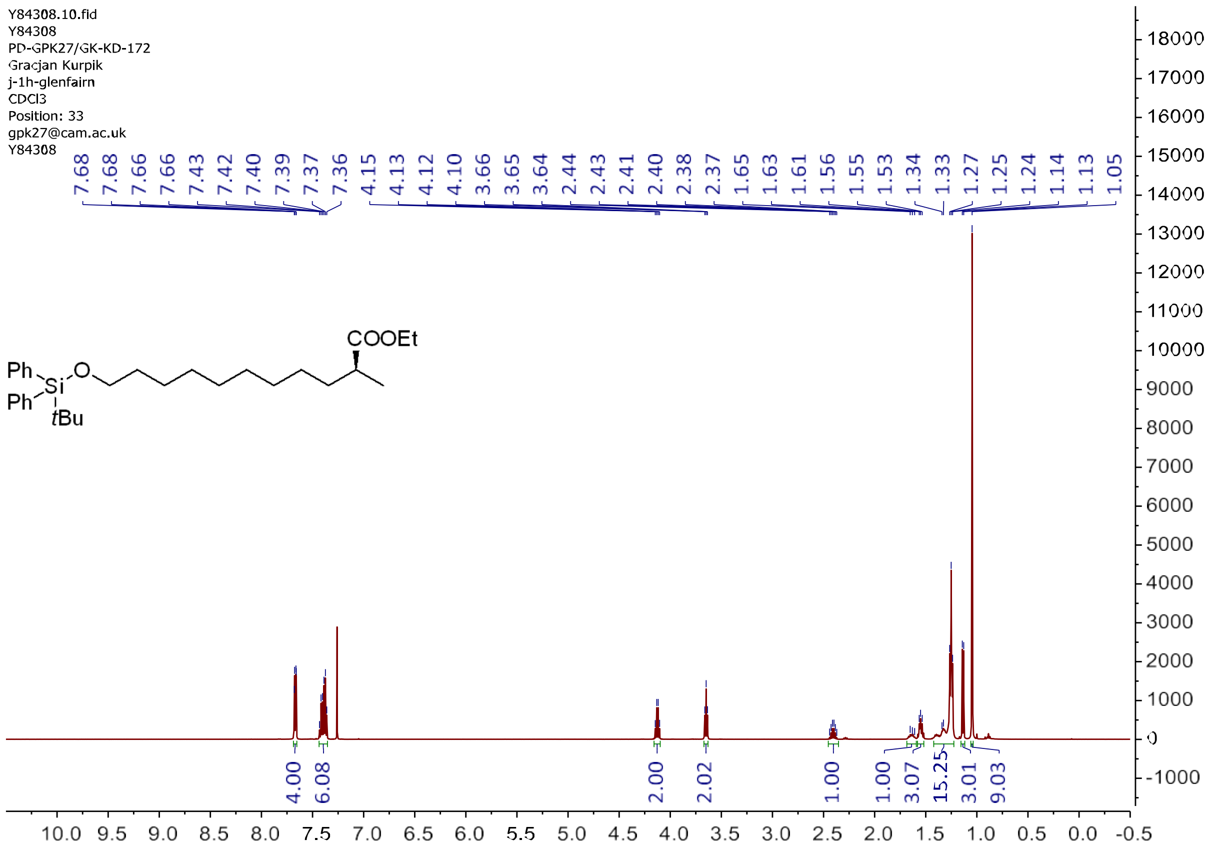
Y84308.10.fid
Y84308
PD-GPK27/GK-KD-172
Gracjan Kurpik
j-1h-glenfairn
CDCl3
Position: 33
gpk27@cam.ac.uk
Y84308
7.68 7.68 7.66 7.66 7.43 7.42 7.40 7.39 7.37 7.36 4.15 4.13 4.12 4.10 3.66 3.65 3.64 2.44 2.43 2.41 2.40 2.38 2.37 1.65 1.63 1.61 1.56 1.55 1.53 1.34 1.33 1.27 1.25 1.24 1.14 1.13 1.05
COOEt
Ph
Si
O
Ph
tBu
4.00 6.08 2.00 2.02 1.00 1.00 3.07 15.25 3.01 9.03

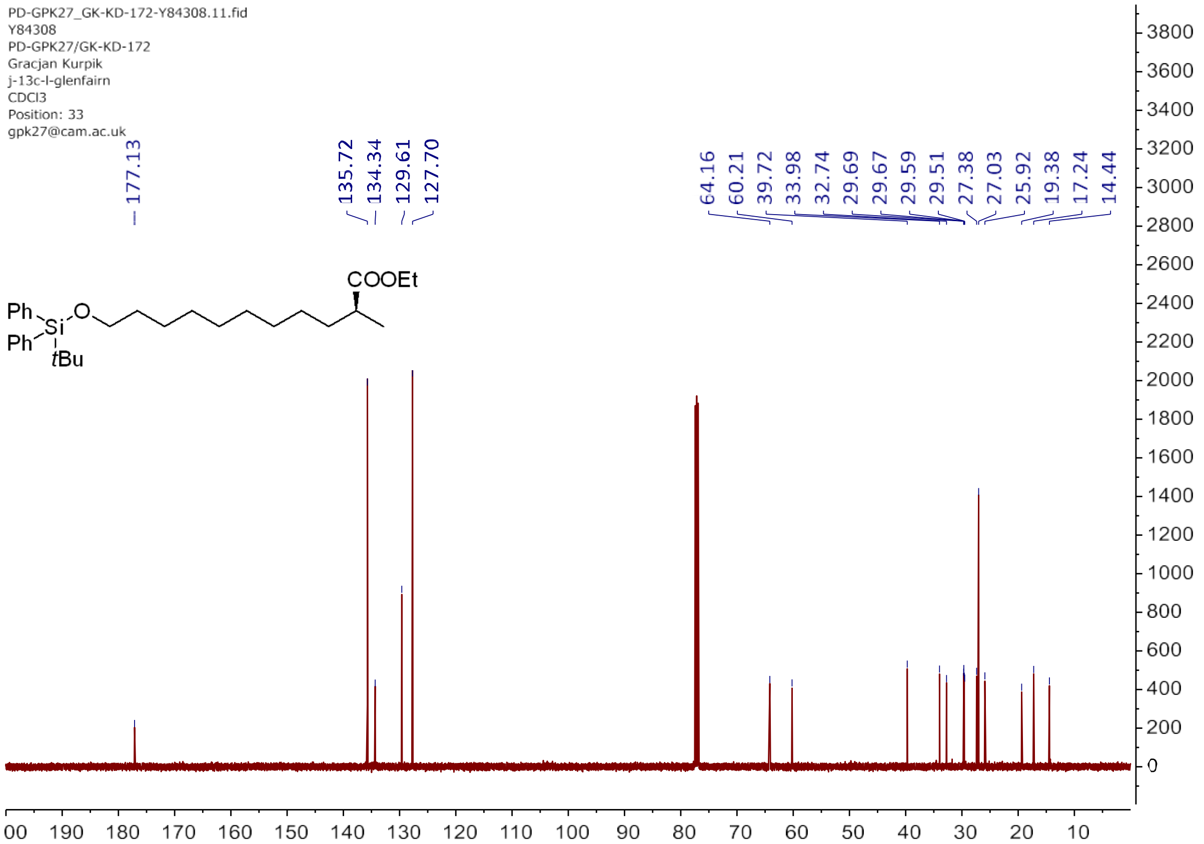
PD-GPK27_GK-KD-172-Y84308.11.fid
Y84308
PD-GPK27/GK-KD-172
Gracjan Kurpik
j-13c-l-glenfairn
CDCl3
Position: 33
gpk27@cam.ac.uk
177.13
135.72 134.34 129.61 127.70
64.16 60.21 39.72 33.98 32.74 29.69 29.67 29.59 29.51 27.38 27.03 25.92 19.38 17.24 14.44
COOEt
Ph
Si
O
Ph
tBu

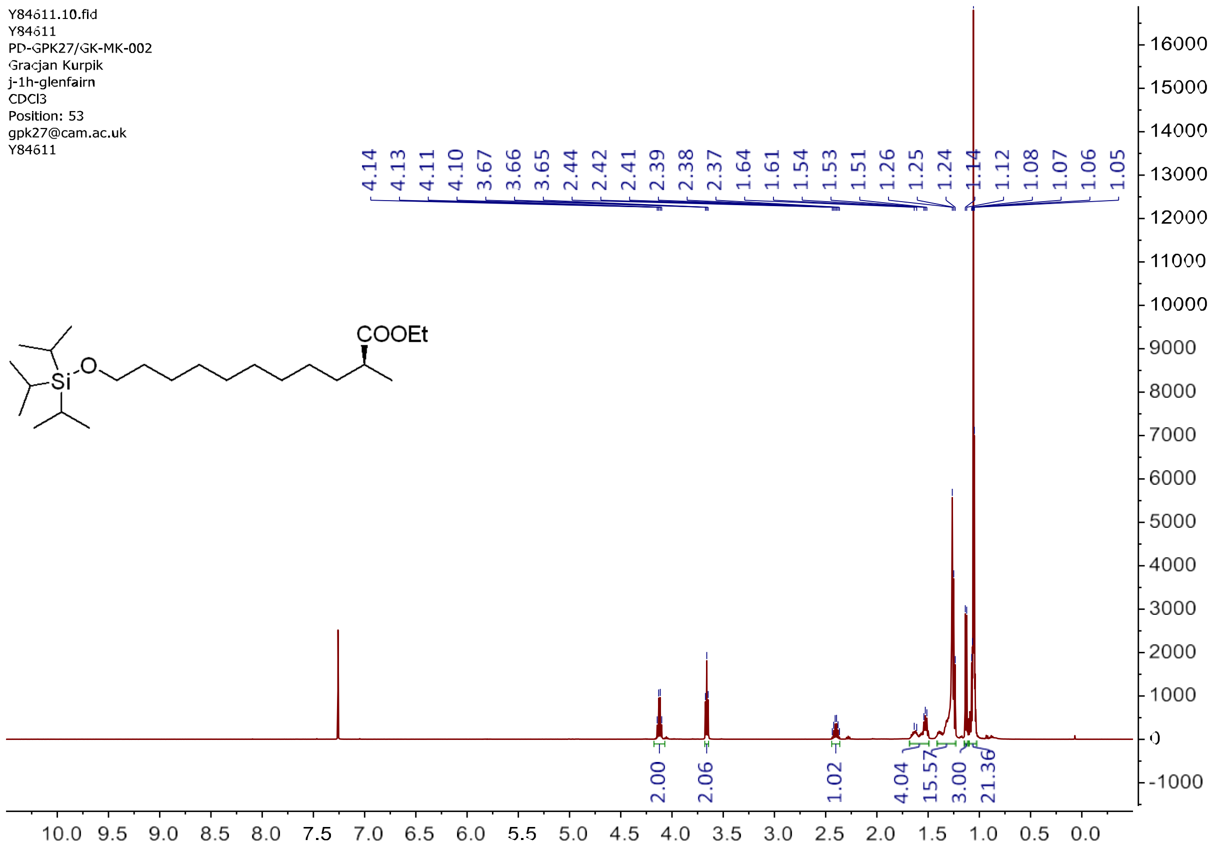
Y84611.10.fid
Y84611
PD-GPK27/GK-MK-002
Gracjan Kurpik
j-1h-glenfairn
CDCl3
Position: 53
gpk27@cam.ac.uk
Y84611
4.14
4.13
4.11
4.10
3.67
3.66
3.65
2.44
2.42
2.41
2.39
2.38
2.37
1.64
1.61
1.54
1.53
1.51
1.26
1.25
1.24
1.14
1.12
1.08
1.07
1.06
1.05
COOEt
Si
O
2.00
2.06
1.02
4.04
15.57
3.00
21.36

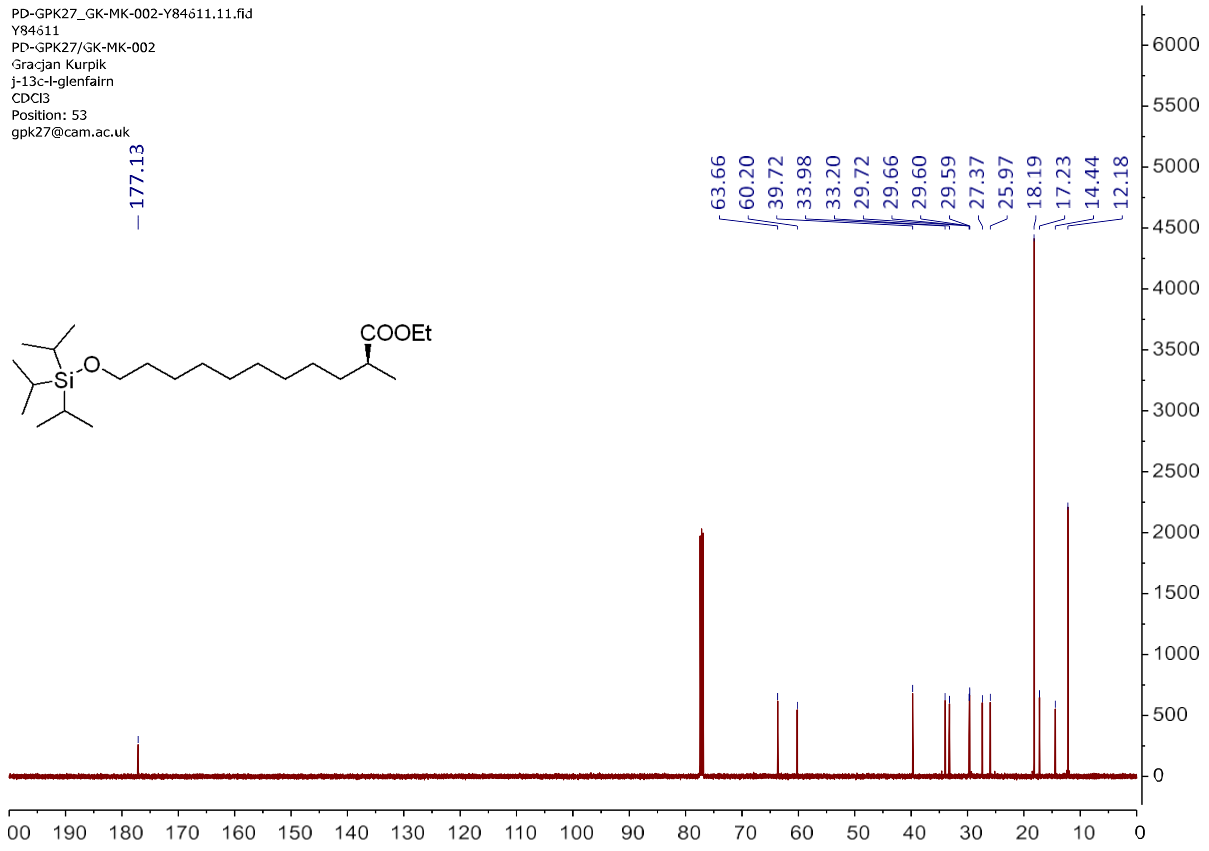
PD-GPK27_GK-MK-002-Y84611.11.fid
Y84611
PD-GPK27/GK-MK-002
Gracjan Kurpik
j-13c-l-glenfairn
CDCl3
Position: 53
gpk27@cam.ac.uk
177.13
63.66
60.20
39.72
33.98
33.20
29.72
29.66
29.60
29.59
27.37
25.97
18.19
17.23
14.44
12.18
COOEt
Si
O

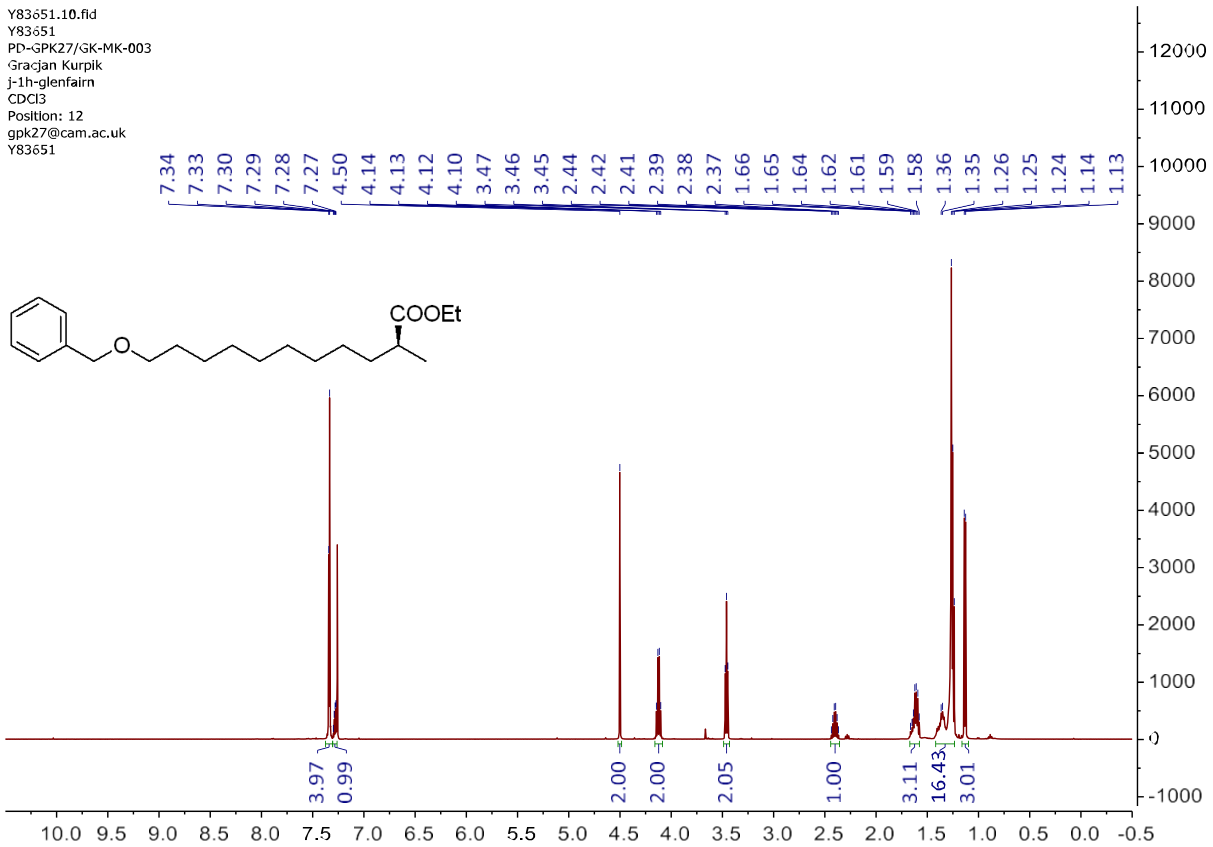
Y83651.10.fid
Y83651
PD-GPK27/GK-MK-003
Gracjan Kurpik
j-1h-glenfairn
CDCl3
Position: 12
gpk27@cam.ac.uk
Y83651
7.34 7.33 7.30 7.29 7.28 7.27 4.50 4.14 4.13 4.12 4.10 3.47 3.46 3.45 2.44 2.42 2.41 2.39 2.38 2.37 1.66 1.65 1.64 1.62 1.61 1.59 1.58 1.36 1.35 1.26 1.25 1.24 1.14 1.13
COOEt
O
3.97 0.99 2.00 2.00 2.05 1.00 3.11 16.43 3.01

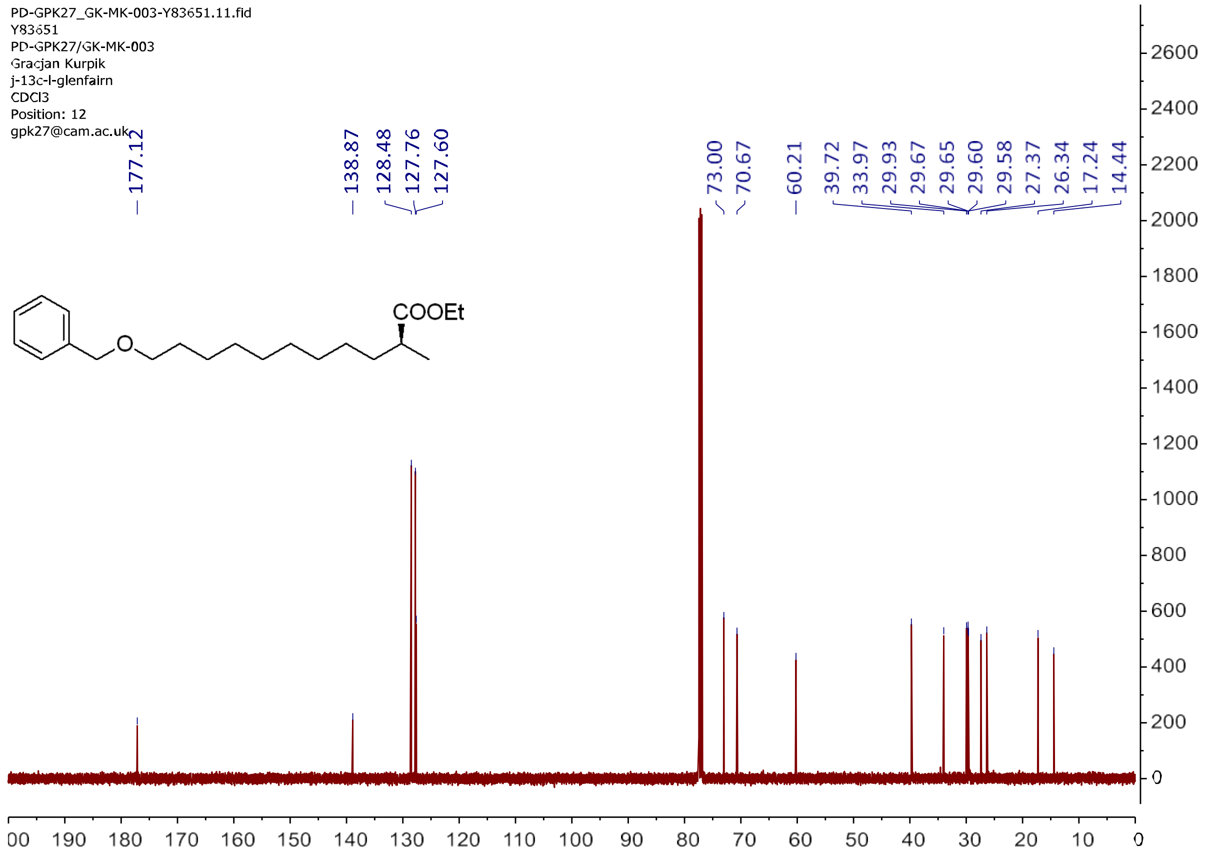
PD-GPK27_GK-MK-003-Y83651.11.fid
Y83651
PD-GPK27/GK-MK-003
Gracjan Kurpik
j-13c-l-glenfairn
CDCl3
Position: 12
gpk27@cam.ac.uk
177.12 138.87 128.48 127.76 127.60 73.00 70.67 60.21 39.72 33.97 29.93 29.67 29.65 29.60 29.58 27.37 26.34 17.24 14.44
COOEt
O

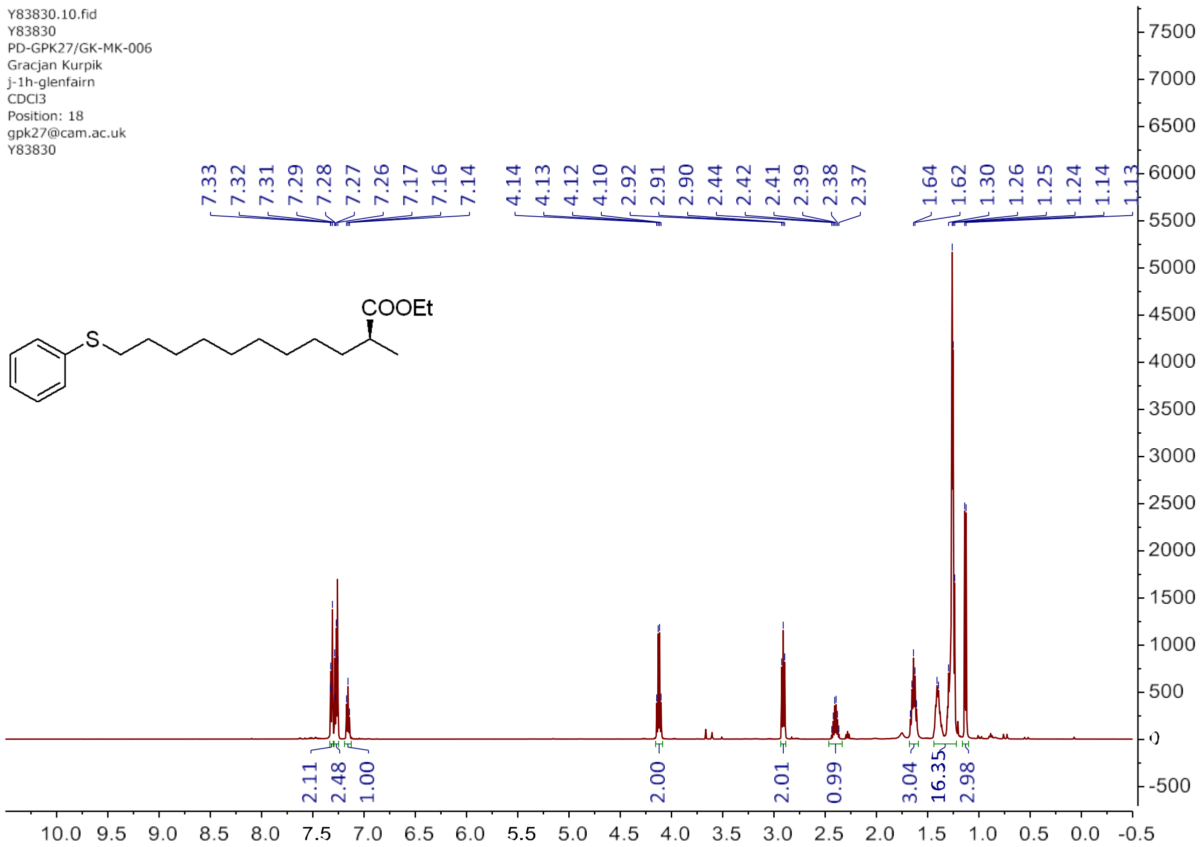
Y83830.10.fid
Y83830
PD-GPK27/GK-MK-006
Gracjan Kurpik
j-1h-glenfairn
CDCl3
Position: 18
gpk27@cam.ac.uk
Y83830
7.33 7.32 7.31 7.29 7.28 7.27 7.26 7.17 7.16 7.14 4.14 4.13 4.12 4.10 2.92 2.91 2.90 2.44 2.42 2.41 2.39 2.38 2.37 1.64 1.62 1.30 1.26 1.25 1.24 1.14 1.13
COOEt
S
2.11 2.48 1.00 2.00 2.01 0.99 3.04 16.35 2.98

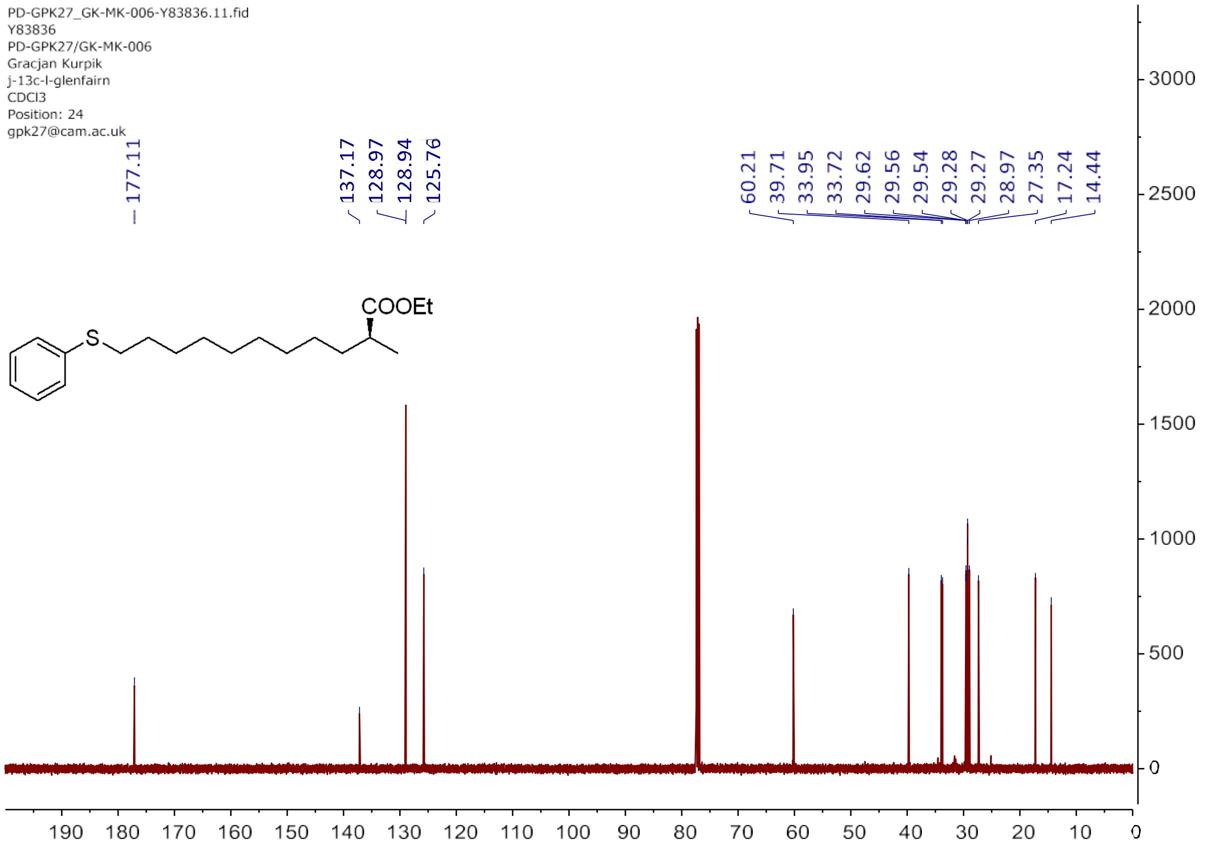
PD-GPK27_GK-MK-006-Y83836.11.fid
Y83836
PD-GPK27/GK-MK-006
Gracjan Kurpik
j-13c-l-glenfairn
CDCl3
Position: 24
gpk27@cam.ac.uk
177.11
137.17 128.97 128.94 125.76
60.21 39.71 33.95 33.72 29.62 29.56 29.54 29.28 29.27 28.97 27.35 17.24 14.44
COOEt
S

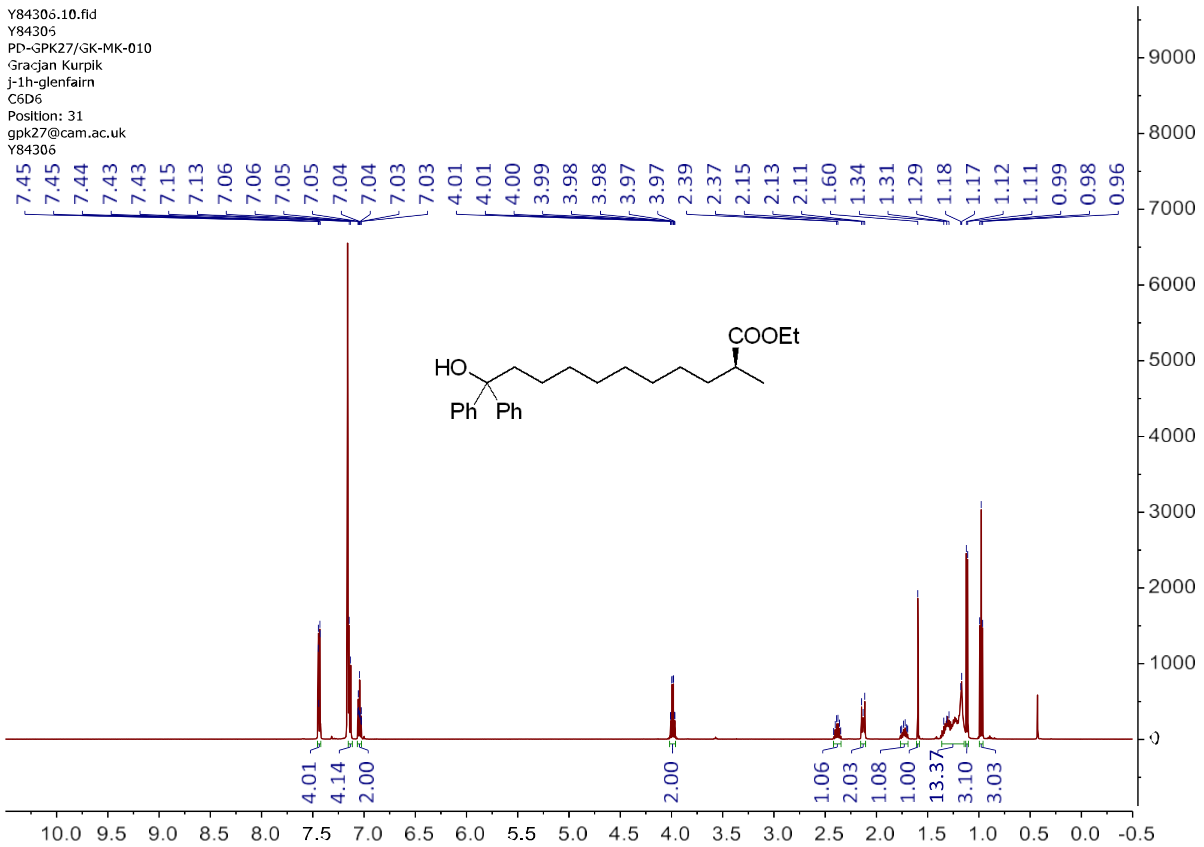

Y84306.10.fid
Y84306
PD-GPK27/GK-MK-010
Gracjan Kurpik
j-1h-glenfairn
C6D6
Position: 31
gpk27@cam.ac.uk
Y84306
7.45 7.45 7.44 7.43 7.43 7.15 7.13 7.06 7.06 7.05 7.05 7.04 7.04 7.03 7.03 4.01 4.01 4.00 3.99 3.98 3.98 3.97 3.97 2.39 2.37 2.15 2.13 2.11 1.60 1.34 1.31 1.29 1.18 1.17 1.12 1.11 0.99 0.98 0.96
COOEt
HO
Ph Ph
4.01 4.14 2.00 2.00 1.06 2.03 1.08 1.00 13.37 3.10 3.03

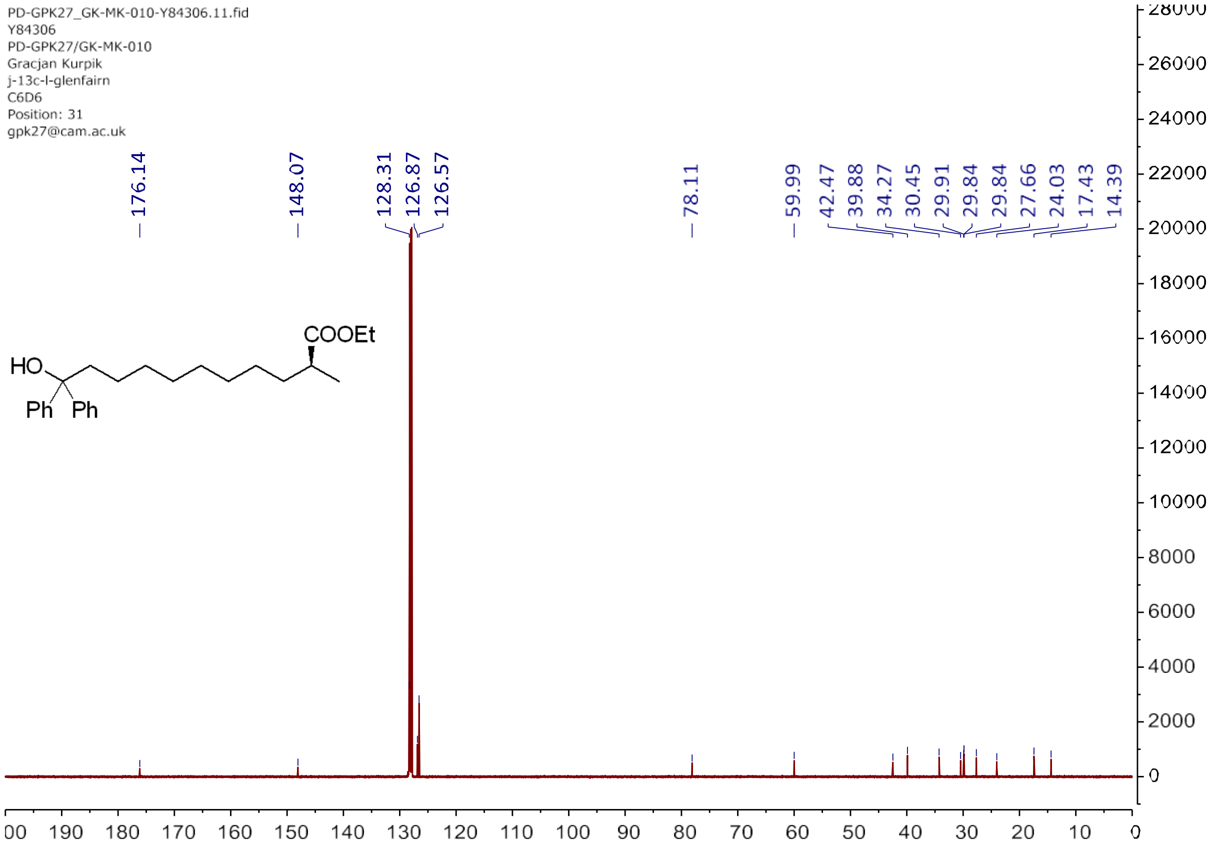

PD-GPK27_GK-MK-010-Y84306.11.fid
Y84306
PD-GPK27/GK-MK-010
Gracjan Kurpik
j-13c-l-glenfairn
C6D6
Position: 31
gpk27@cam.ac.uk
176.14 148.07 128.31 126.87 126.57 78.11 59.99 42.47 39.88 34.27 30.45 29.91 29.84 29.84 27.66 24.03 17.43 14.39
COOEt
HO
Ph Ph

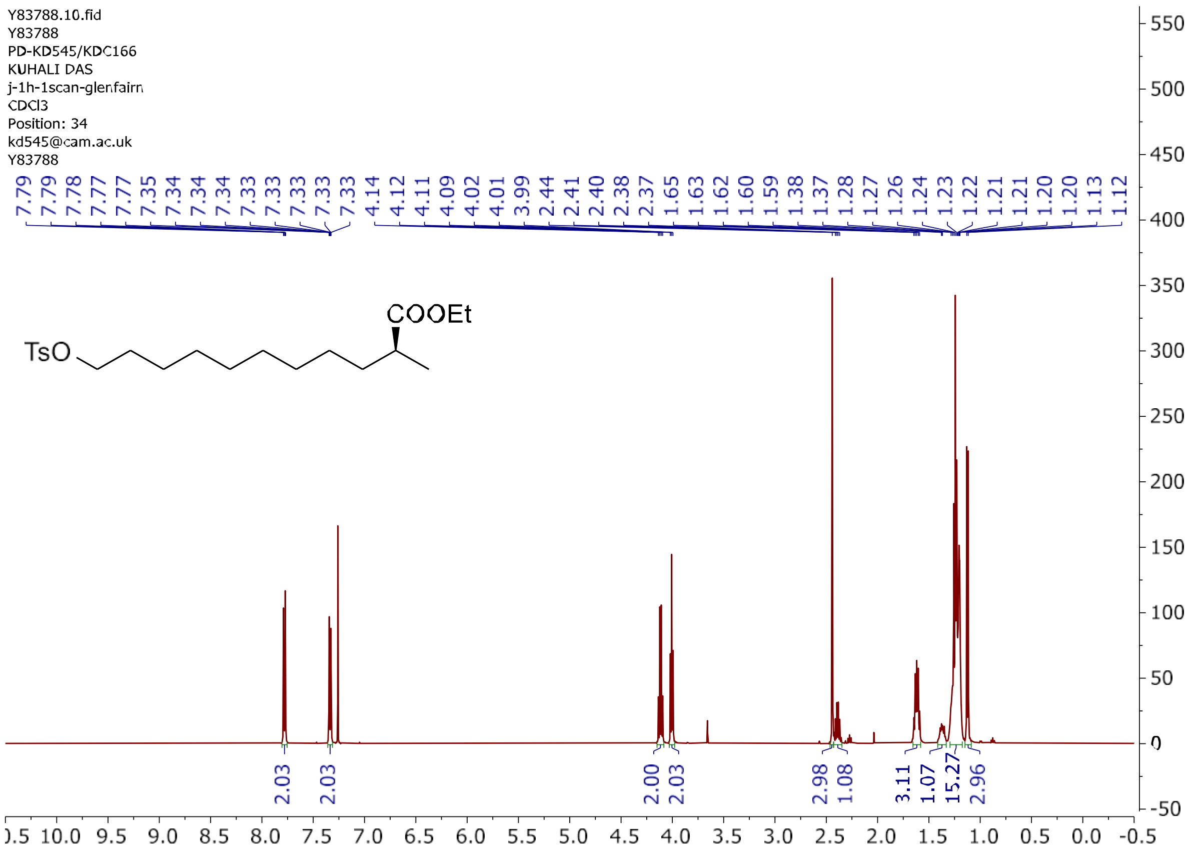
Y83788.10.fid
Y83788
PD-KD545/KDC166
KUHALI DAS
j-1h-1scan-glenfairn
CDCl3
Position: 34
kd545@cam.ac.uk
Y83788
7.79 7.79 7.78 7.77 7.77 7.35 7.34 7.34 7.34 7.33 7.33 7.33 7.33 7.33 4.14 4.12 4.11 4.09 4.02 4.01 3.99 2.44 2.41 2.40 2.38 2.37 1.65 1.63 1.62 1.60 1.59 1.38 1.37 1.28 1.27 1.26 1.24 1.23 1.22 1.21 1.21 1.20 1.20 1.13 1.12
TsO
COOEt
2.03 2.03 2.00 2.03 2.98 1.08 3.11 1.07 15.27 2.96

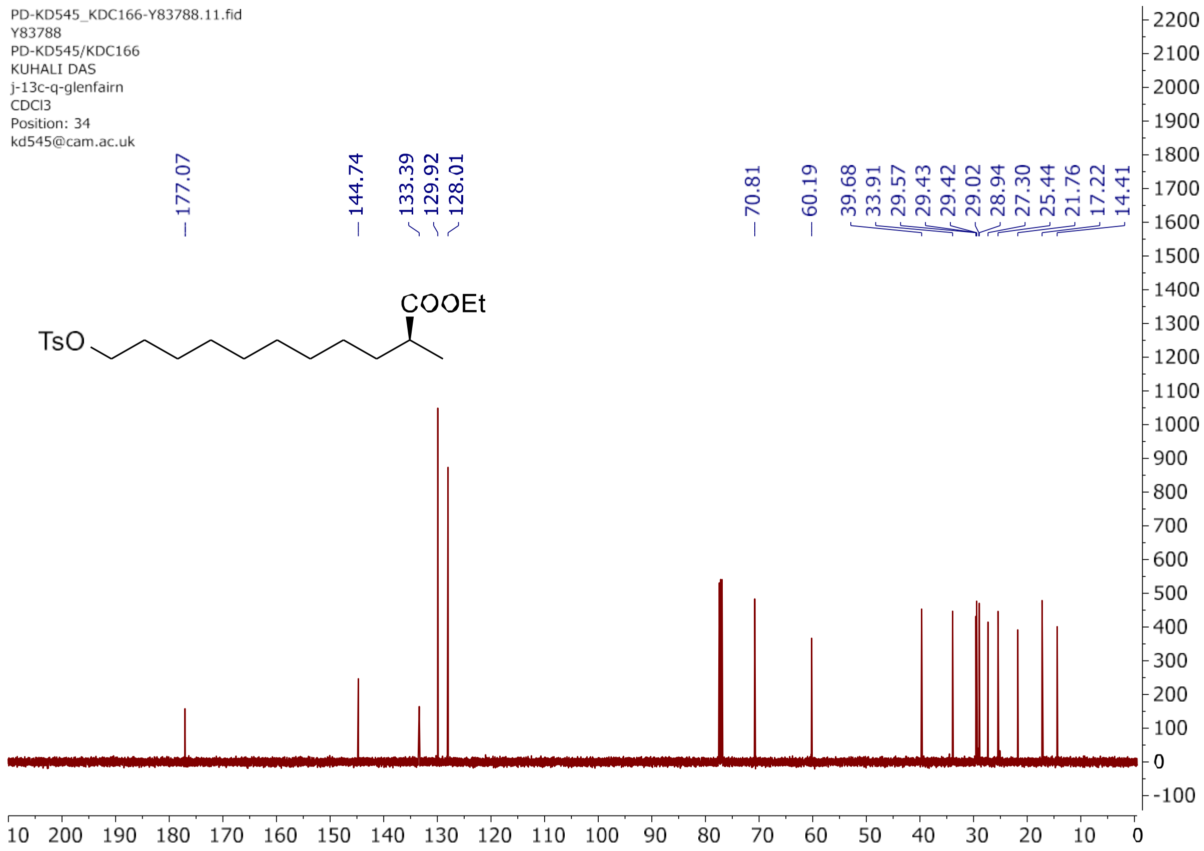
PD-KD545_KDC166-Y83788.11.fid
Y83788
PD-KD545/KDC166
KUHALI DAS
j-13c-q-glenfairn
CDCl3
Position: 34
kd545@cam.ac.uk
177.07 144.74 133.39 129.92 128.01 70.81 60.19 39.68 33.91 29.57 29.43 29.42 29.02 28.94 27.30 25.44 21.76 17.22 14.41
TsO
COOEt

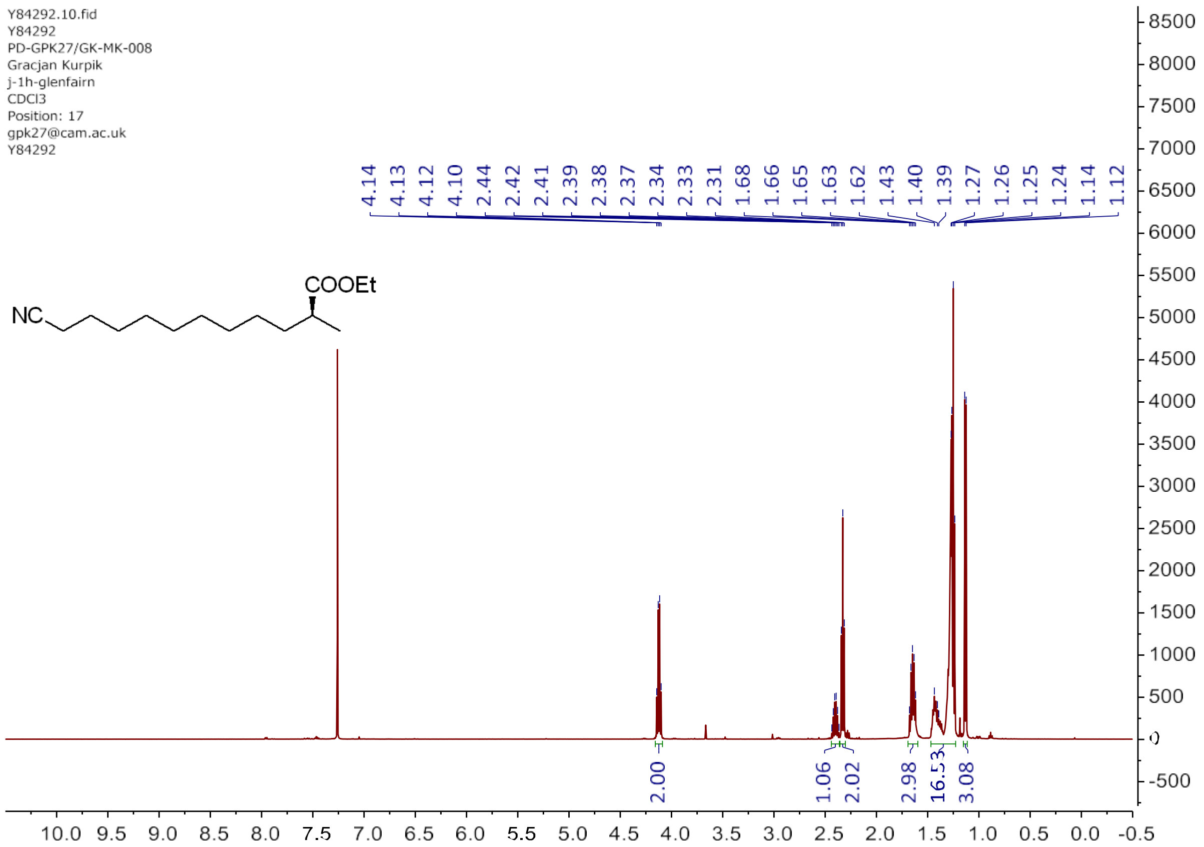
Y84292.10.fid
Y84292
PD-GPK27/GK-MK-008
Gracjan Kurpik
j-1h-glenfairn
CDCl3
Position: 17
gpk27@cam.ac.uk
Y84292
4.14
4.13
4.12
4.10
2.44
2.42
2.41
2.39
2.38
2.37
2.34
2.33
2.31
1.68
1.66
1.65
1.63
1.62
1.43
1.40
1.39
1.27
1.26
1.25
1.24
1.14
1.12
COOEt
NC
2.00
1.06
2.02
2.98
16.53
3.08

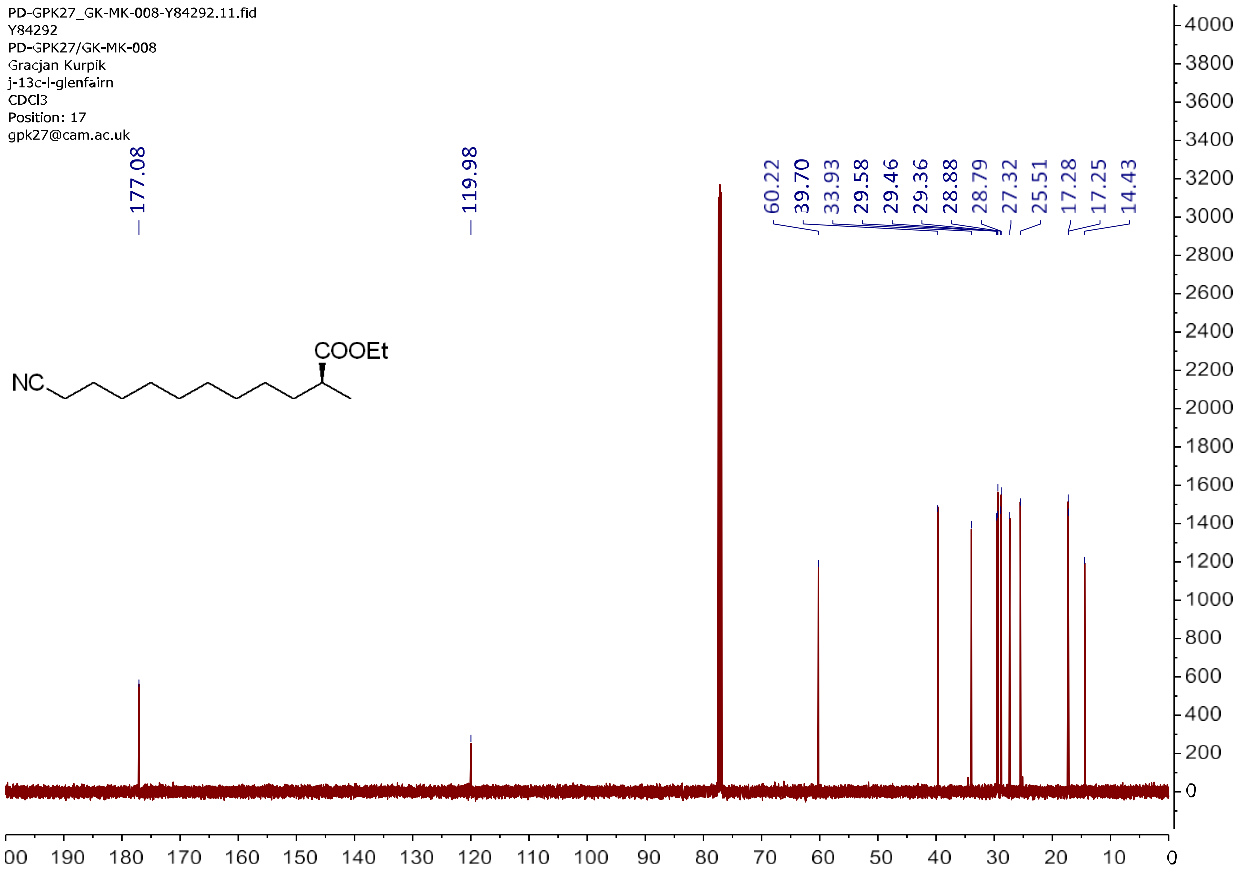
PD-GPK27_GK-MK-008-Y84292.11.fid
Y84292
PD-GPK27/GK-MK-008
Gracjan Kurpik
j-13c-l-glenfairn
CDCl3
Position: 17
gpk27@cam.ac.uk
177.08
119.98
60.22
39.70
33.93
29.58
29.46
29.36
28.88
28.79
27.32
25.51
17.28
17.25
14.43
COOEt
NC

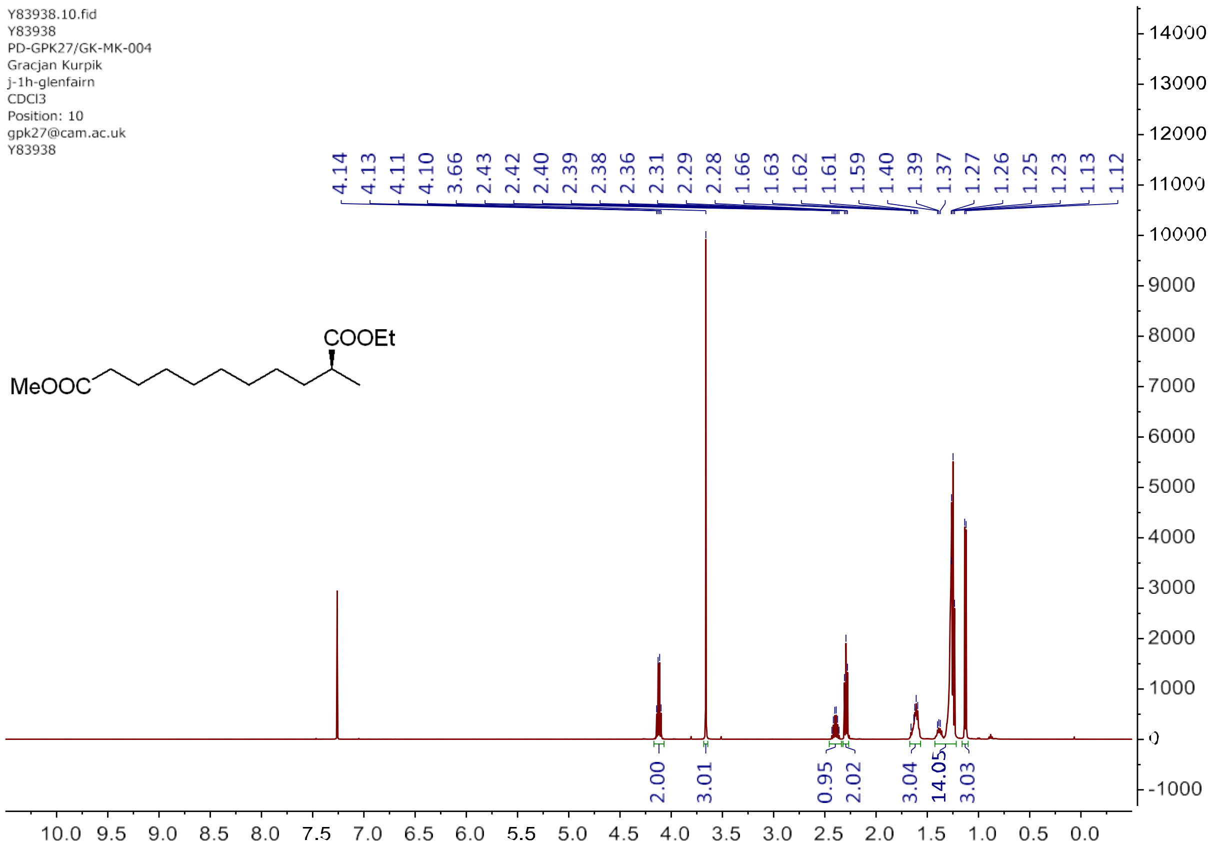
Y83938.10.fid
Y83938
PD-GPK27/GK-MK-004
Gracjan Kurpik
j-1h-glenfairn
CDCl3
Position: 10
gpk27@cam.ac.uk
Y83938
4.14
4.13
4.11
4.10
3.66
2.43
2.42
2.40
2.39
2.38
2.36
2.31
2.29
2.28
1.66
1.63
1.62
1.61
1.59
1.40
1.39
1.37
1.27
1.26
1.25
1.23
1.13
1.12
COOEt
MeOOC
2.00
3.01
0.95
2.02
3.04
14.05
3.03

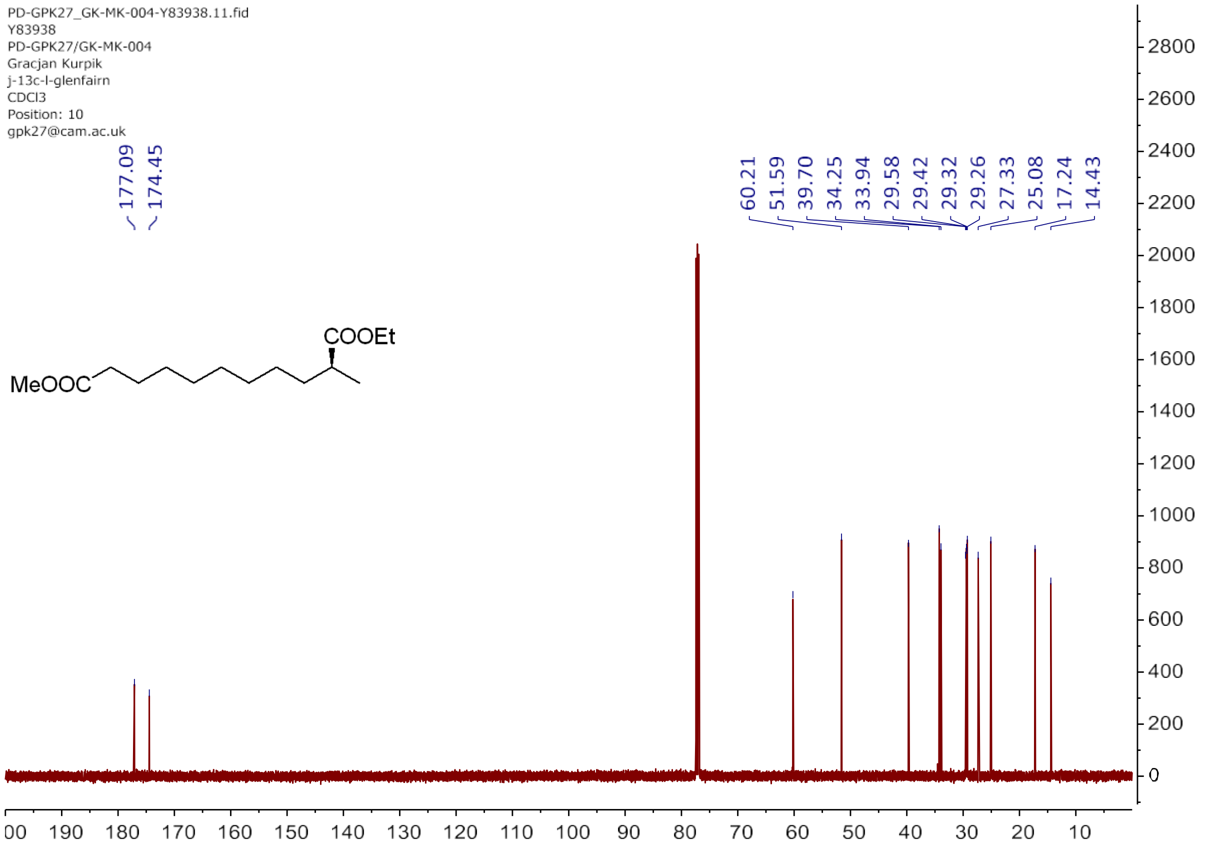
PD-GPK27_GK-MK-004-Y83938.11.fid
Y83938
PD-GPK27/GK-MK-004
Gracjan Kurpik
j-13c-l-glenfairn
CDCl3
Position: 10
gpk27@cam.ac.uk
177.09
174.45
60.21
51.59
39.70
34.25
33.94
29.58
29.42
29.32
29.26
27.33
25.08
17.24
14.43
COOEt
MeOOC

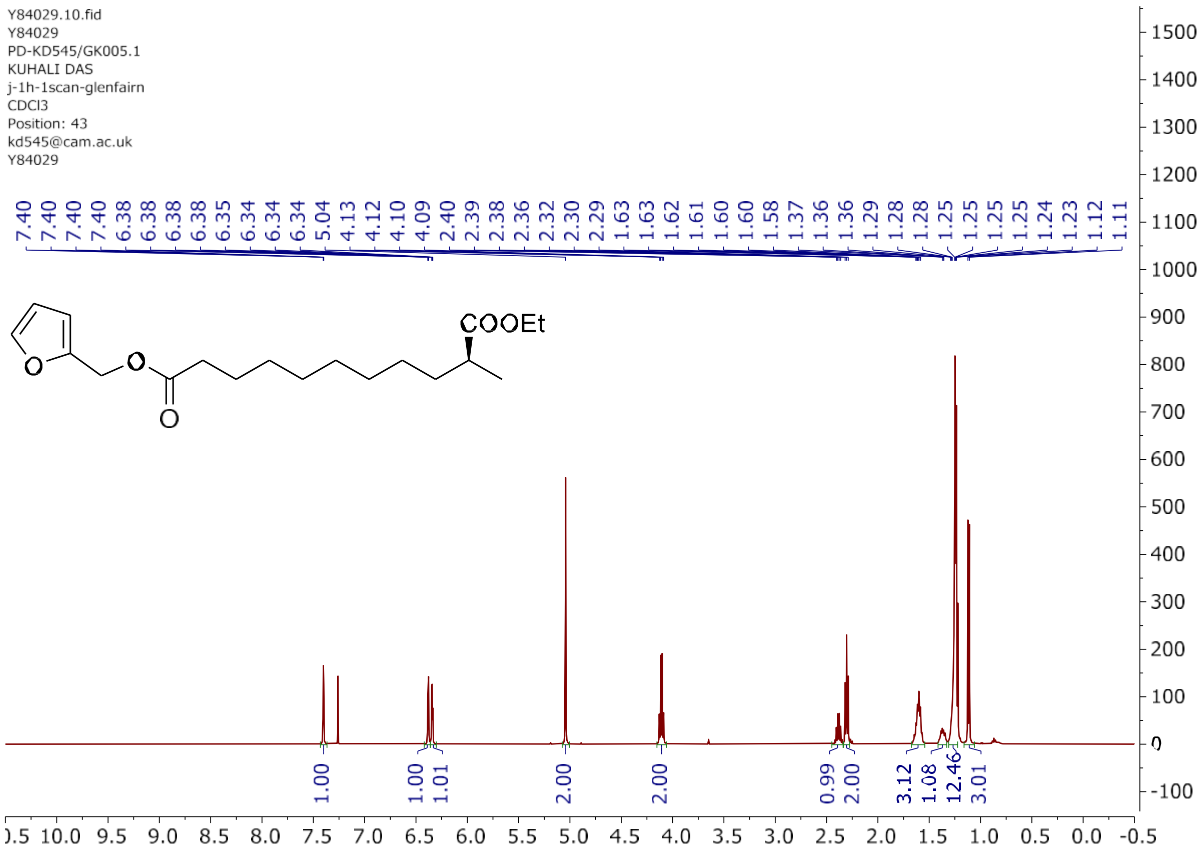
Y84029.10.fid
Y84029
PD-KD545/GK005.1
KUHALI DAS
j-1h-1scan-glenfairn
CDCl3
Position: 43
kd545@cam.ac.uk
Y84029
7.40 7.40 7.40 7.40 6.38 6.38 6.38 6.38 6.35 6.34 6.34 6.34 5.04 4.13 4.12 4.10 4.09 2.40 2.39 2.38 2.36 2.32 2.30 2.29 1.63 1.63 1.62 1.61 1.60 1.60 1.58 1.37 1.36 1.36 1.29 1.28 1.28 1.25 1.25 1.25 1.25 1.24 1.23 1.12 1.11
COOEt
O
O
O
1.00 1.00 1.01 2.00 2.00 0.99 2.00 3.12 1.08 12.46 3.01

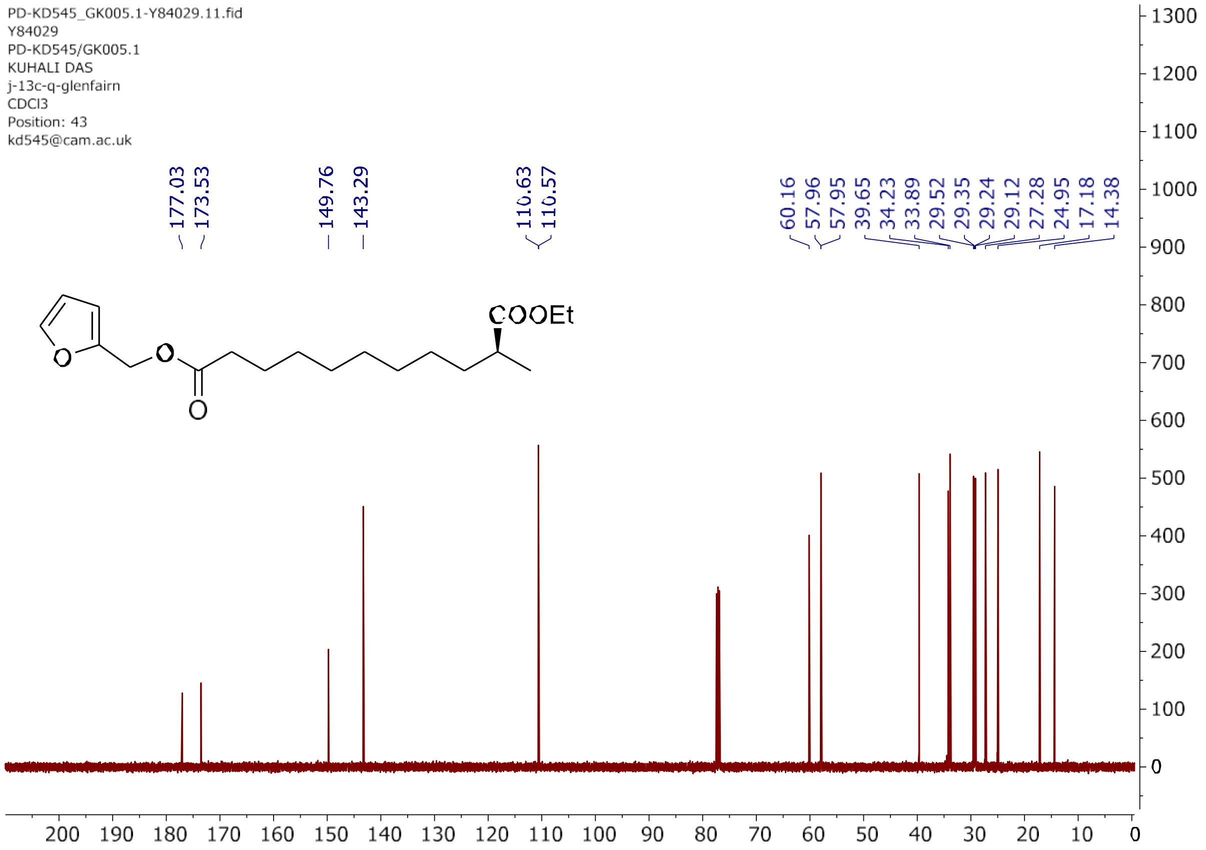
PD-KD545_GK005.1-Y84029.11.fid
Y84029
PD-KD545/GK005.1
KUHALI DAS
j-13c-q-glenfairn
CDCl3
Position: 43
kd545@cam.ac.uk
177.03 173.53 149.76 143.29 110.63 110.57 60.16 57.96 57.95 39.65 34.23 33.89 29.52 29.35 29.24 29.12 27.28 24.95 17.18 14.38
COOEt
O
O
O

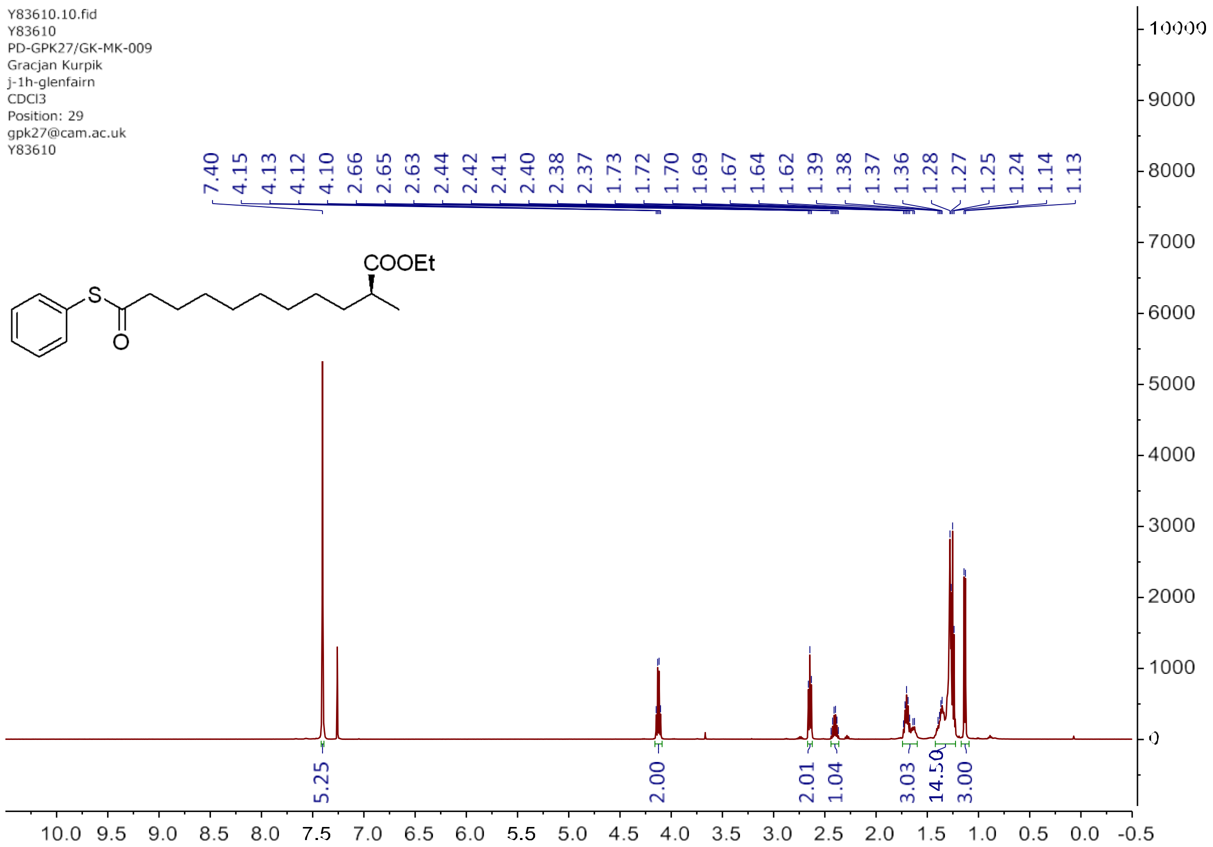
Y83610.10.fid
Y83610
PD-GPK27/GK-MK-009
Gracjan Kurpik
j-1h-glenfairn
CDCl3
Position: 29
gpk27@cam.ac.uk
Y83610
7.40 4.15 4.13 4.12 4.10 2.66 2.65 2.63 2.44 2.42 2.41 2.40 2.38 2.37 1.73 1.72 1.70 1.69 1.67 1.64 1.62 1.39 1.38 1.37 1.36 1.28 1.27 1.25 1.24 1.14 1.13
COOEt
S
O
5.25 2.00 2.01 1.04 3.03 14.50 3.00
10.0 9.5 9.0 8.5 8.0 7.5 7.0 6.5 6.0 5.5 5.0 4.5 4.0 3.5 3.0 2.5 2.0 1.5 1.0 0.5 0.0 -0.5
10000 9000 8000 7000 6000 5000 4000 3000 2000 1000 0

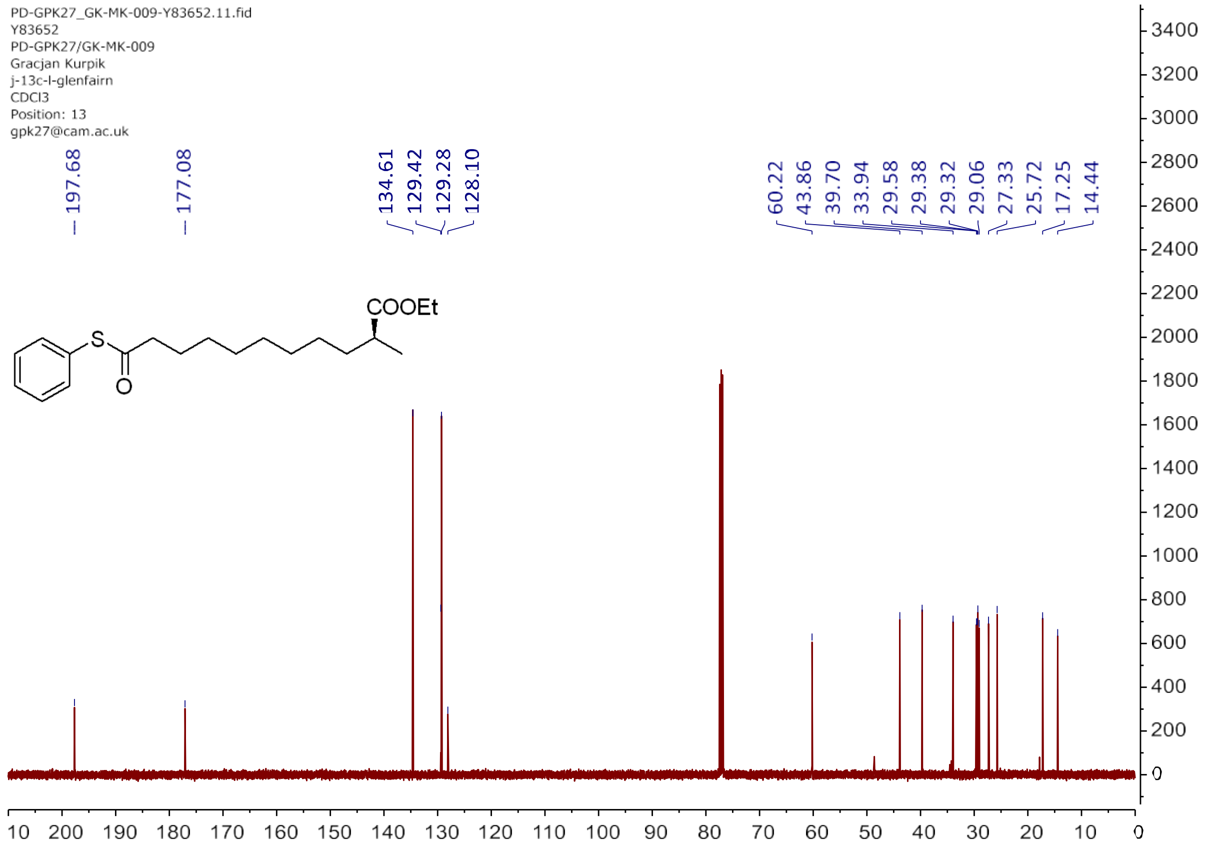
PD-GPK27_GK-MK-009-Y83652.11.fid
Y83652
PD-GPK27/GK-MK-009
Gracjan Kurpik
j-13c-l-glenfairn
CDCl3
Position: 13
gpk27@cam.ac.uk
197.68 177.08 134.61 129.42 129.28 128.10 60.22 43.86 39.70 33.94 29.58 29.38 29.32 29.06 27.33 25.72 17.25 14.44
COOEt
S
O
210 200 190 180 170 160 150 140 130 120 110 100 90 80 70 60 50 40 30 20 10 0
3400 3200 3000 2800 2600 2400 2200 2000 1800 1600 1400 1200 1000 800 600 400 200 0

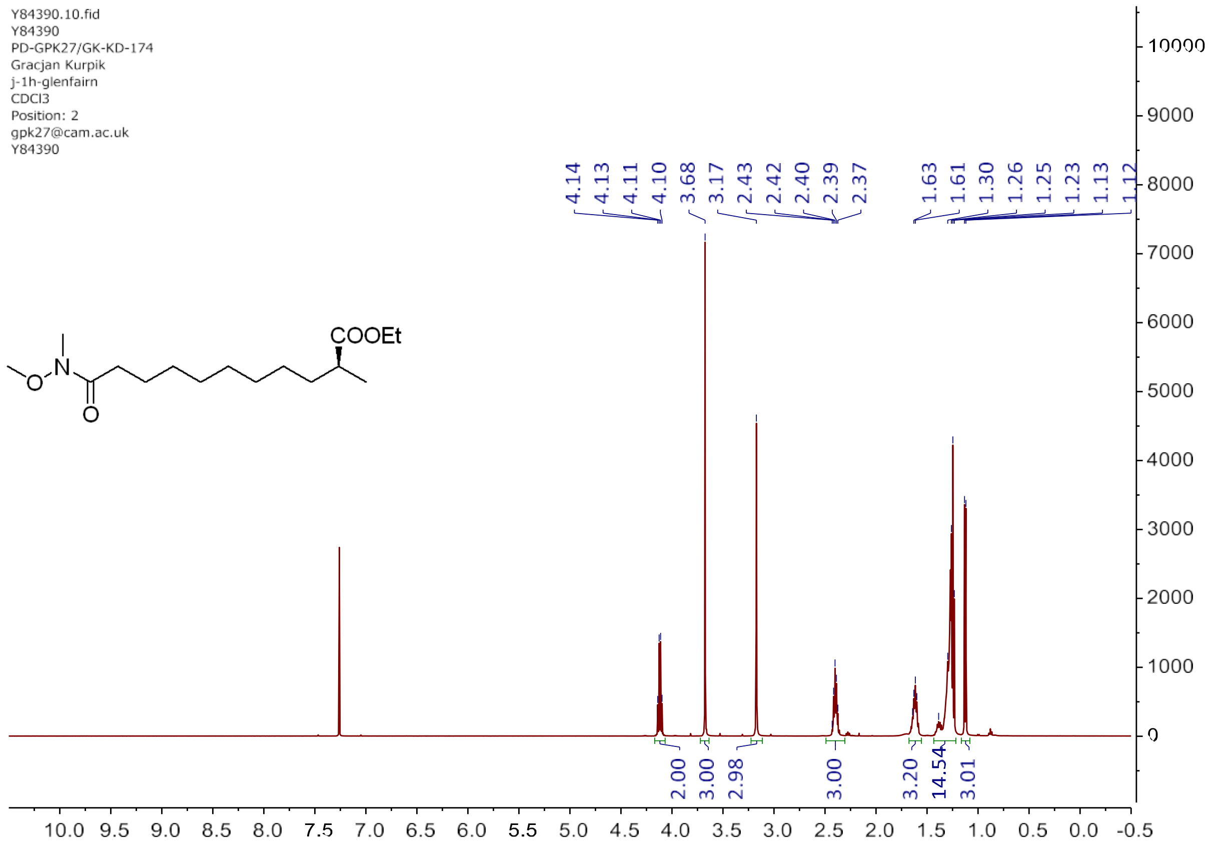
Y84390.10.fid
Y84390
PD-GPK27/GK-KD-174
Gracjan Kurpik
j-1h-glenfairn
CDCl3
Position: 2
gpk27@cam.ac.uk
Y84390
COOEt
N
O
O

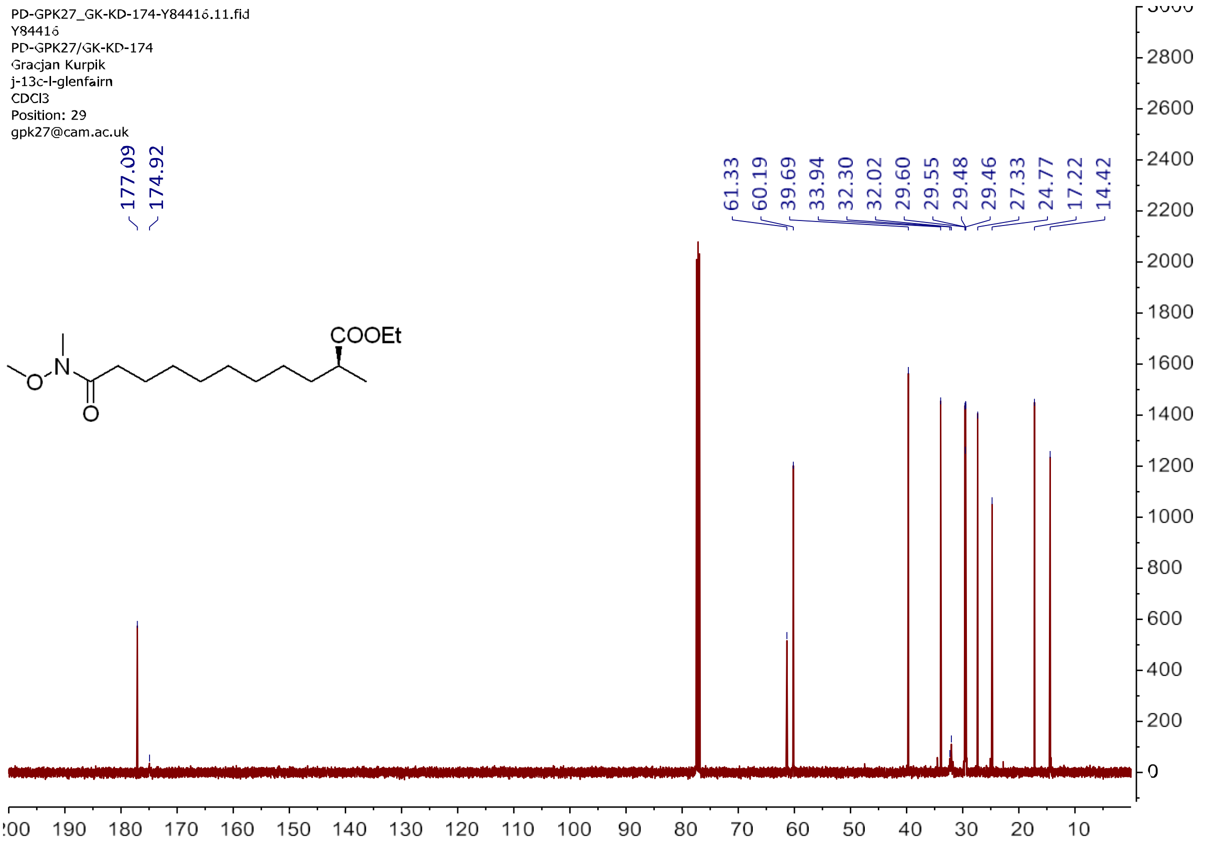
PD-GPK27_GK-KD-174-Y84416.11.fid
Y84416
PD-GPK27/GK-KD-174
Gracjan Kurpik
j-13c-l-glenfairn
CDCl3
Position: 29
gpk27@cam.ac.uk
COOEt
N
O
O

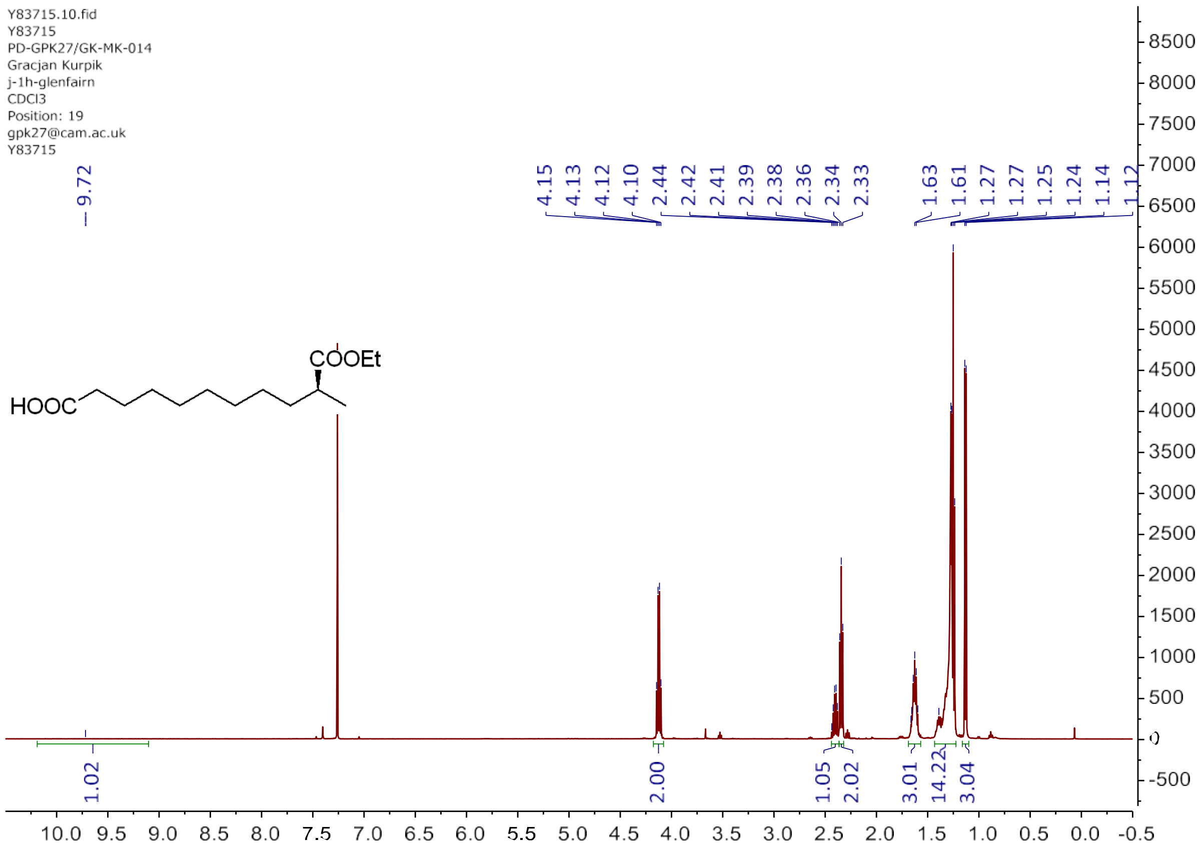
Y83715.10.fid
Y83715
PD-GPK27/GK-MK-014
Gracjan Kurpik
j-1h-glenfairn
CDCl3
Position: 19
gpk27@cam.ac.uk
Y83715
HOOC
COOEt

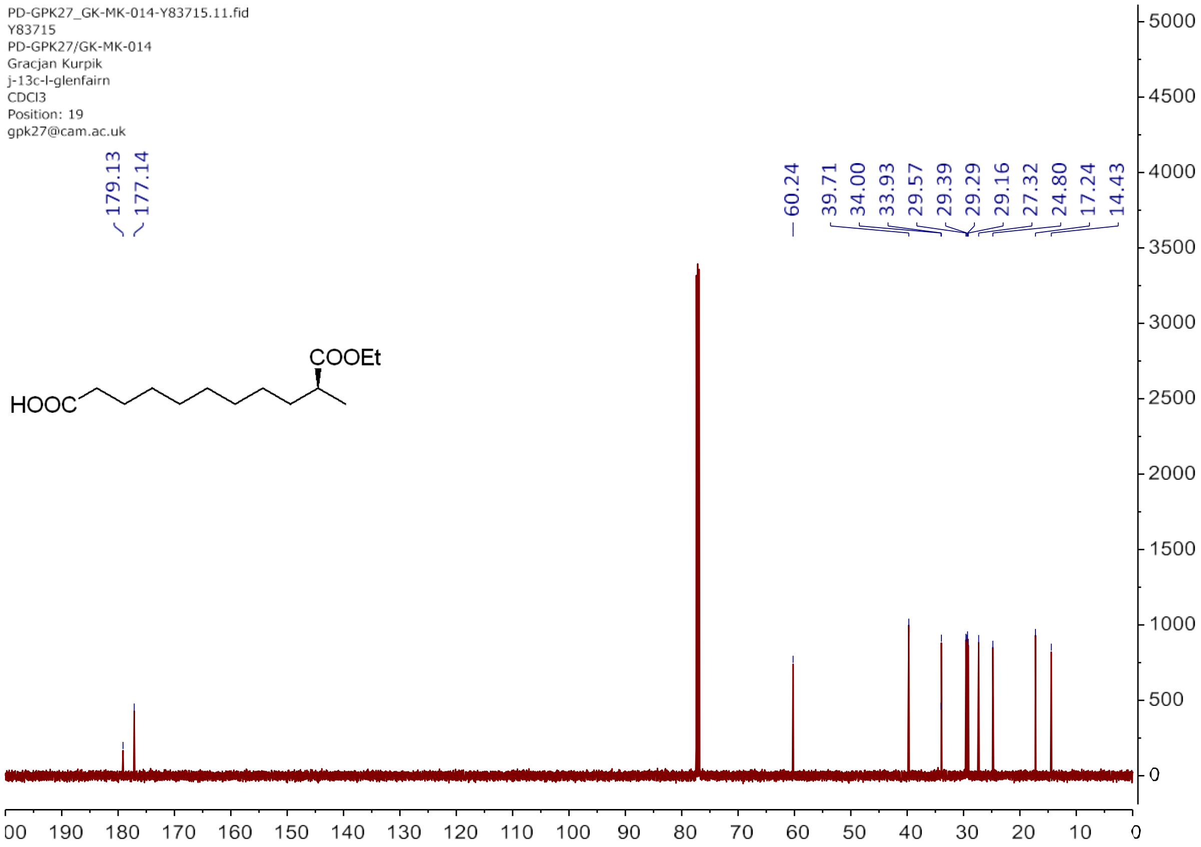
PD-GPK27_GK-MK-014-Y83715.11.fid
Y83715
PD-GPK27/GK-MK-014
Gracjan Kurpik
j-13c-l-glenfairn
CDCl3
Position: 19
gpk27@cam.ac.uk
HOOC
COOEt

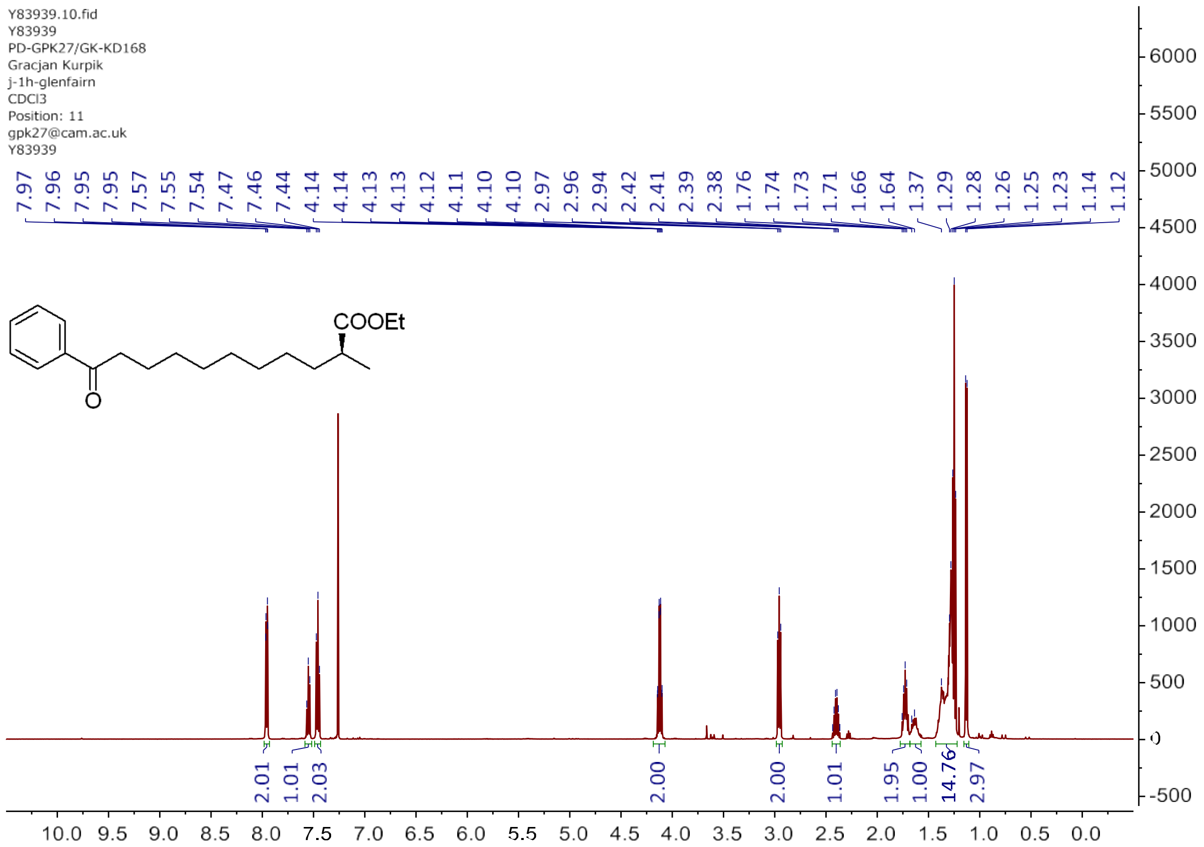
Y83939.10.fid
Y83939
PD-GPK27/GK-KD168
Gracjan Kurpik
j-1h-glenfairn
CDCl3
Position: 11
gpk27@cam.ac.uk
Y83939
7.97 7.96 7.95 7.95 7.57 7.55 7.54 7.47 7.46 7.44 4.14 4.14 4.13 4.13 4.12 4.11 4.10 4.10 2.97 2.96 2.94 2.42 2.41 2.39 2.38 1.76 1.74 1.73 1.71 1.66 1.64 1.37 1.29 1.28 1.26 1.25 1.23 1.14 1.12
COOEt
O
2.01 1.01 2.03 2.00 2.00 1.01 1.95 1.00 14.76 2.97

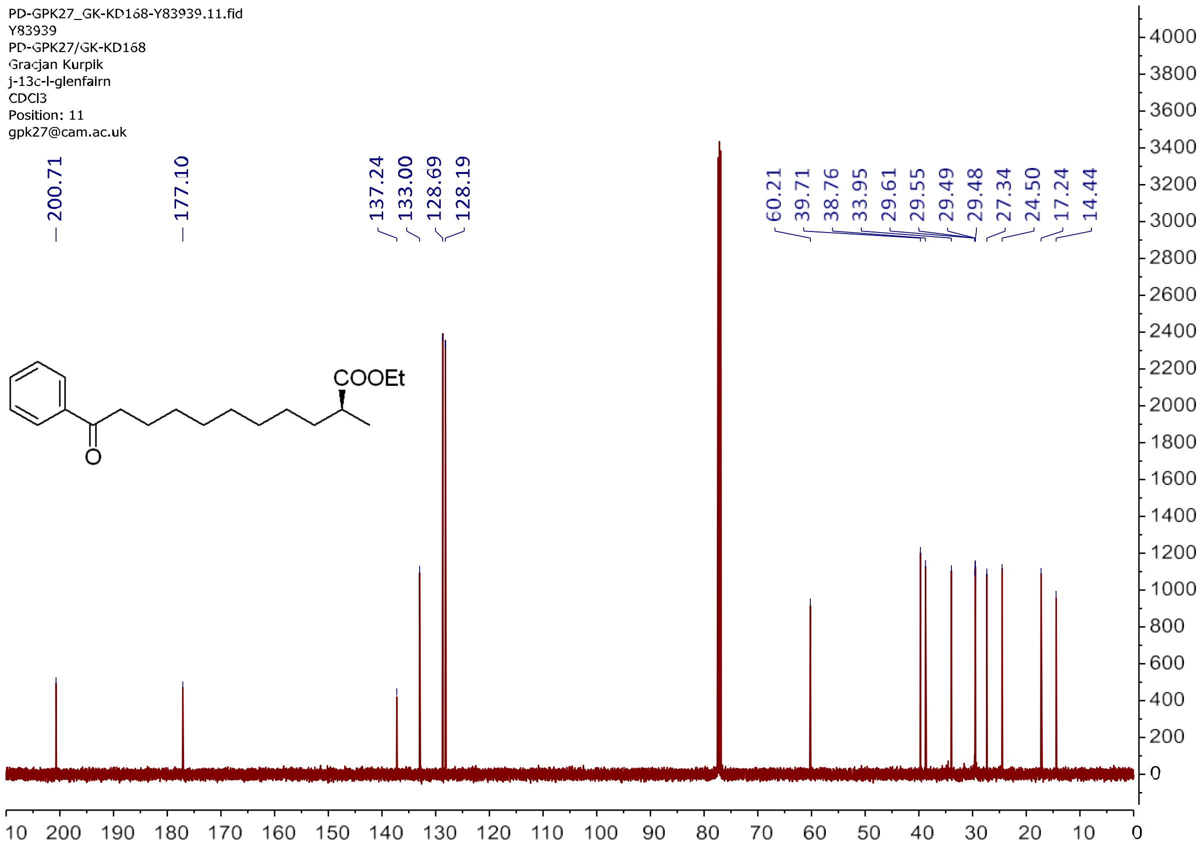
PD-GPK27_GK-KD168-Y83939.11.fid
Y83939
PD-GPK27/GK-KD168
Gracjan Kurpik
j-13c-l-glenfairn
CDCl3
Position: 11
gpk27@cam.ac.uk
200.71
177.10
137.24 133.00 128.69 128.19
60.21 39.71 38.76 33.95 29.61 29.55 29.49 29.48 27.34 24.50 17.24 14.44
COOEt
O

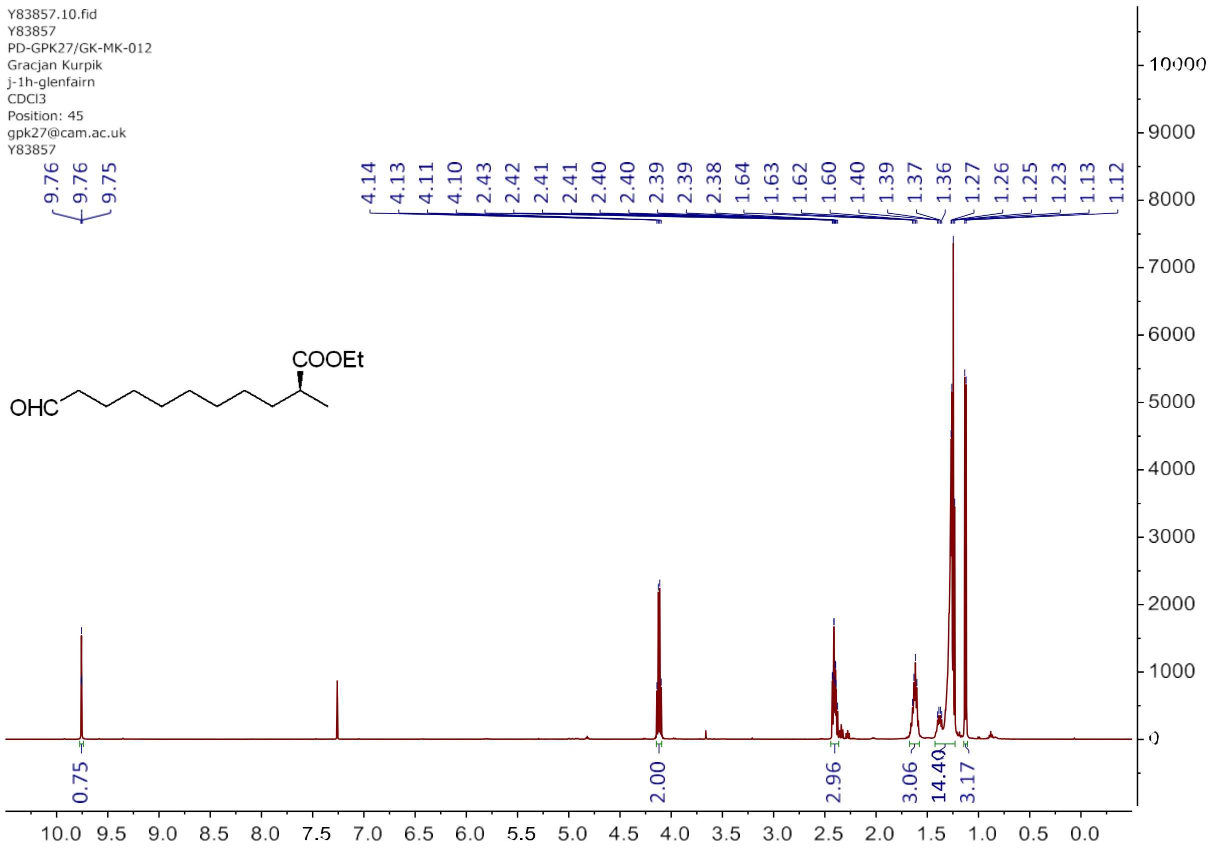
Y83857.10.fid
Y83857
PD-GPK27/GK-MK-012
Gracjan Kurpik
j-1h-glenfairn
CDCl3
Position: 45
gpk27@cam.ac.uk
Y83857
9.76
9.76
9.75
4.14
4.13
4.11
4.10
2.43
2.42
2.41
2.41
2.40
2.40
2.39
2.39
2.38
1.64
1.63
1.62
1.60
1.40
1.39
1.37
1.36
1.27
1.26
1.25
1.23
1.13
1.12
COOEt
OHC
0.75
2.00
2.96
3.06
14.40
3.17

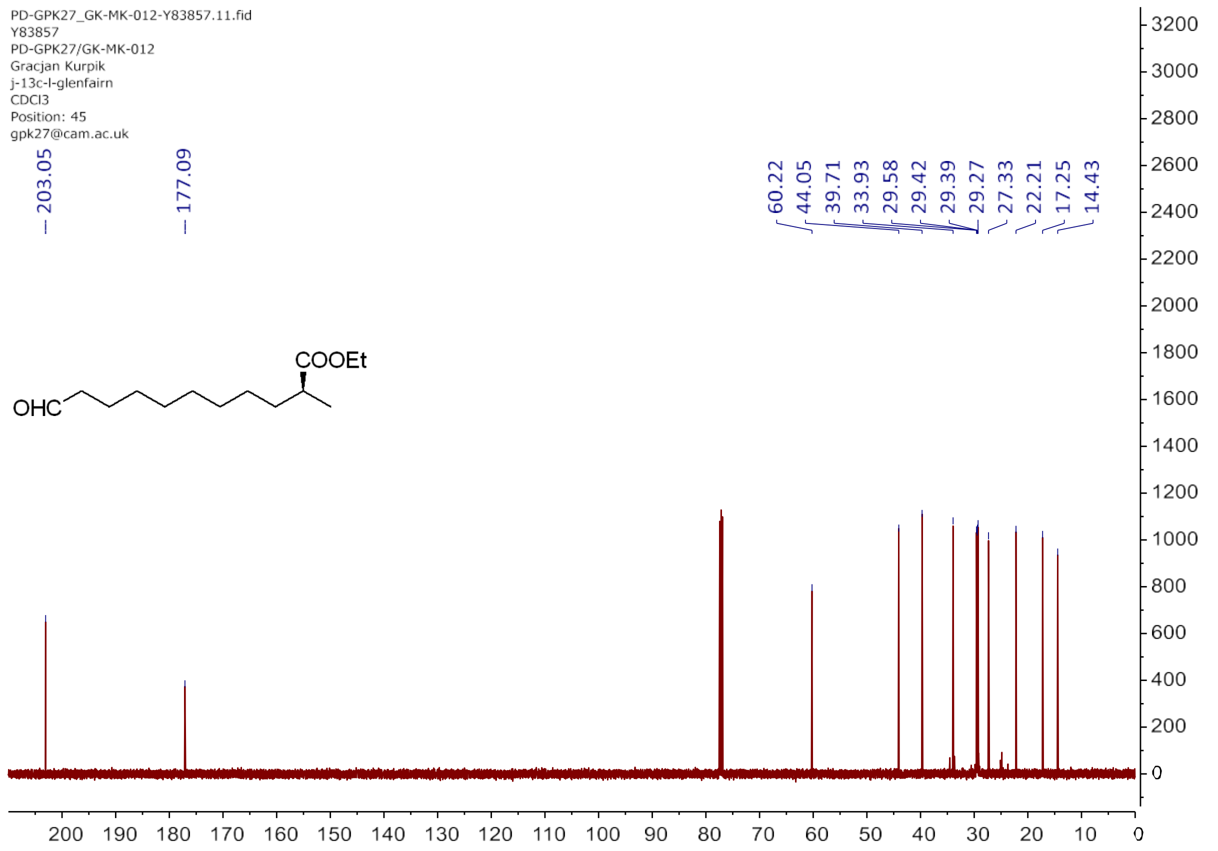
PD-GPK27_GK-MK-012-Y83857.11.fid
Y83857
PD-GPK27/GK-MK-012
Gracjan Kurpik
j-13c-l-glenfairn
CDCl3
Position: 45
gpk27@cam.ac.uk
203.05
177.09
60.22
44.05
39.71
33.93
29.58
29.42
29.39
29.27
27.33
22.21
17.25
14.43
COOEt
OHC

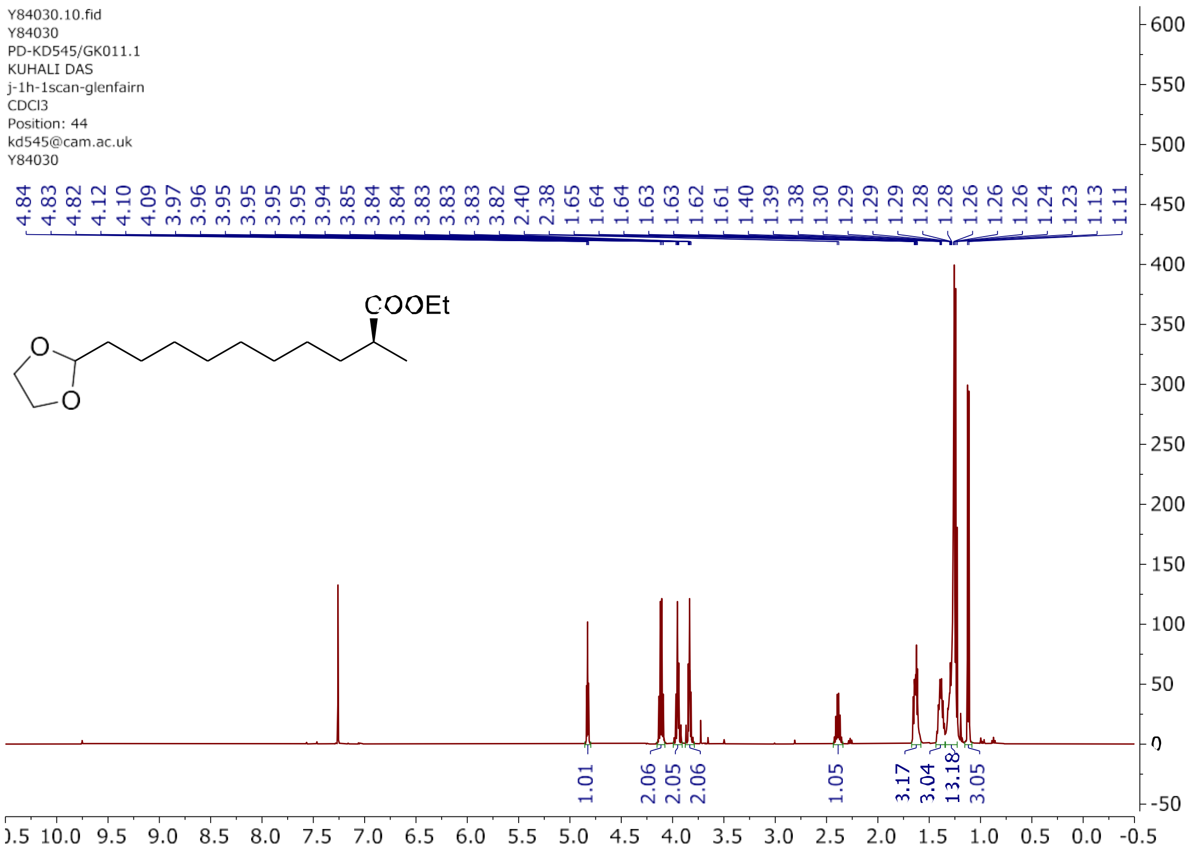
Y84030.10.fid
Y84030
PD-KD545/GK011.1
KUHALI DAS
j-1h-1scan-glenfairn
CDCl3
Position: 44
kd545@cam.ac.uk
Y84030
4.84 4.83 4.82 4.12 4.10 4.09 3.97 3.96 3.95 3.95 3.95 3.94 3.85 3.84 3.84 3.83 3.83 3.83 3.82 2.40 2.38 1.65 1.64 1.64 1.63 1.63 1.62 1.61 1.40 1.39 1.38 1.30 1.29 1.29 1.29 1.28 1.28 1.26 1.26 1.26 1.24 1.23 1.13 1.11
COOEt
O
O
1.01 2.06 2.05 2.06 1.05 3.17 3.04 13.18 3.05

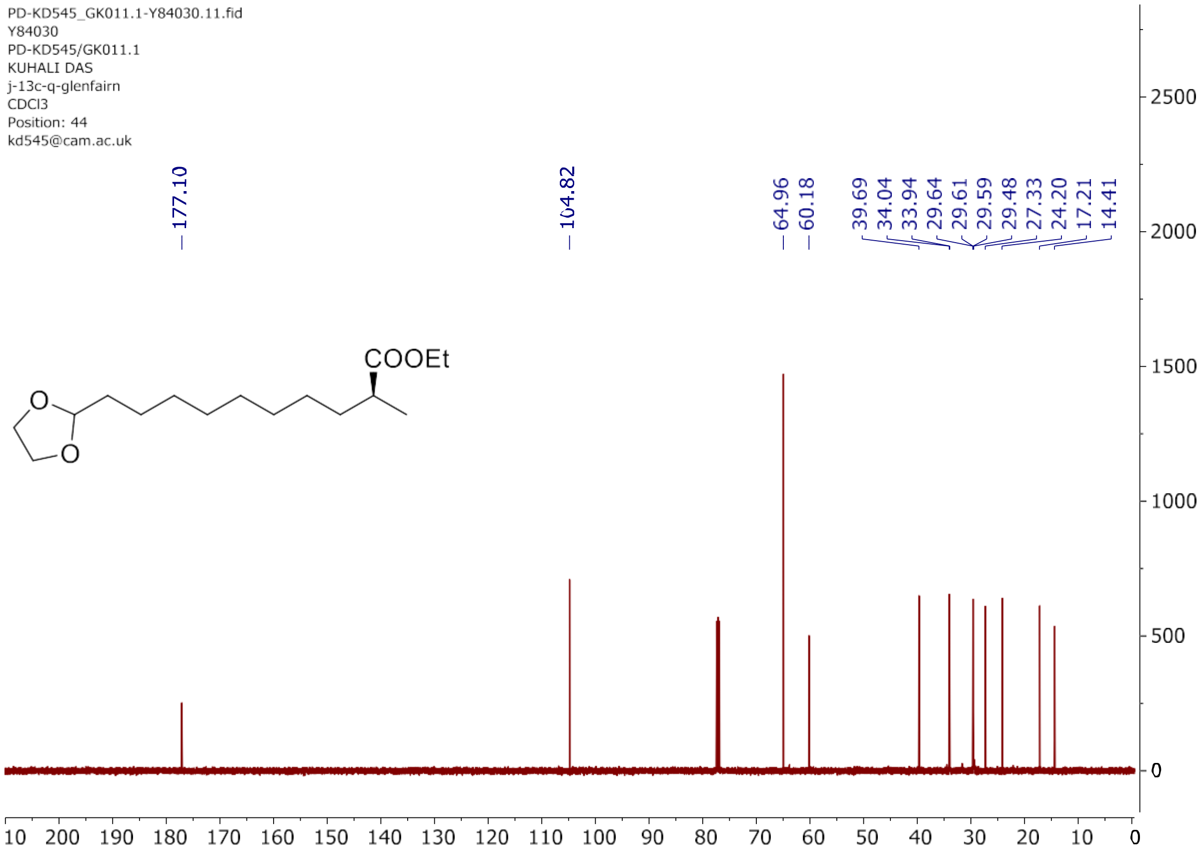
PD-KD545_GK011.1-Y84030.11.fid
Y84030
PD-KD545/GK011.1
KUHALI DAS
j-13c-q-glenfairn
CDCl3
Position: 44
kd545@cam.ac.uk
177.10
104.82
64.96
60.18
39.69 34.04 33.94 29.64 29.61 29.59 29.48 27.33 24.20 17.21 14.41
COOEt
O
O

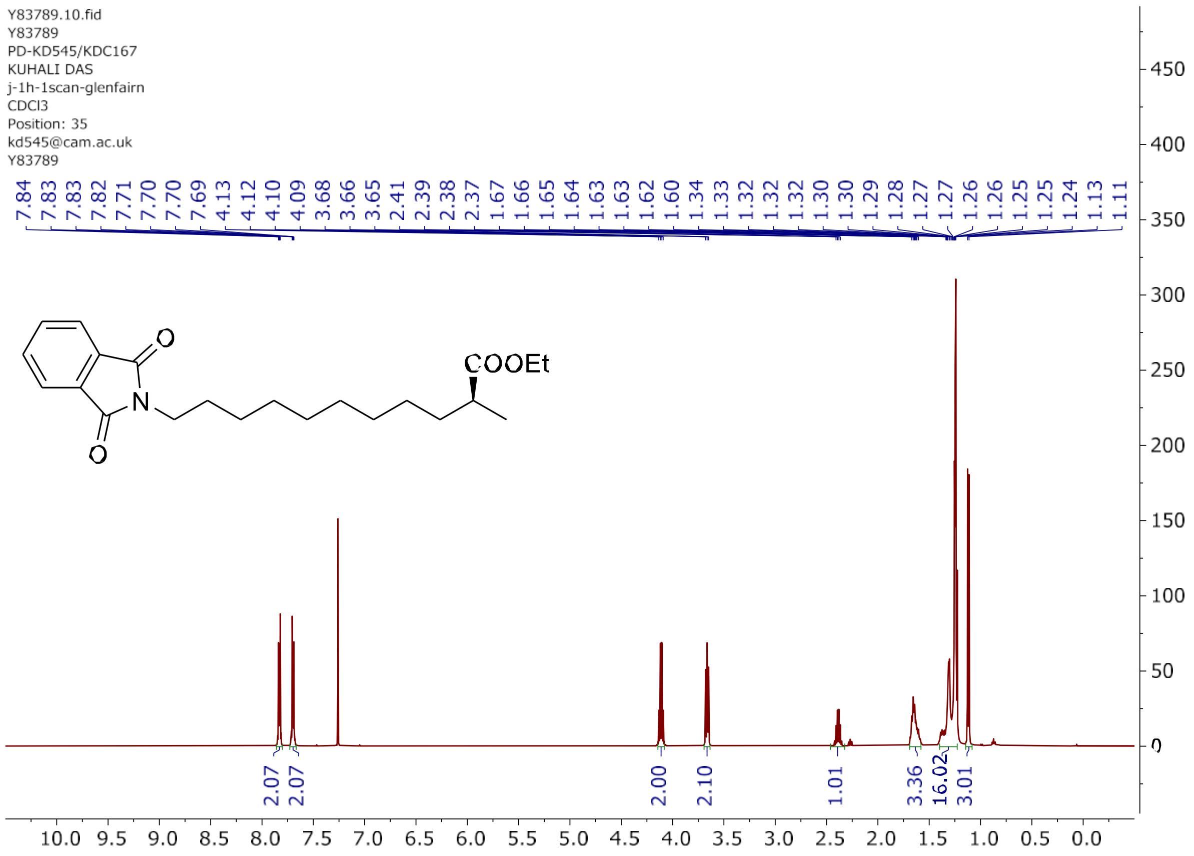
Y83789.10.fid
Y83789
PD-KD545/KDC167
KUHALI DAS
j-1h-1scan-glenfairn
CDCl3
Position: 35
kd545@cam.ac.uk
Y83789
7.84 7.83 7.83 7.82 7.71 7.70 7.70 7.69 4.13 4.12 4.10 4.09 3.68 3.66 3.65 2.41 2.39 2.38 2.37 1.67 1.66 1.65 1.64 1.63 1.63 1.62 1.60 1.34 1.33 1.32 1.32 1.32 1.30 1.30 1.29 1.28 1.27 1.27 1.26 1.26 1.25 1.25 1.24 1.13 1.11
COOEt
N
O
O
2.07 2.07 2.00 2.10 1.01 3.36 16.02 3.01

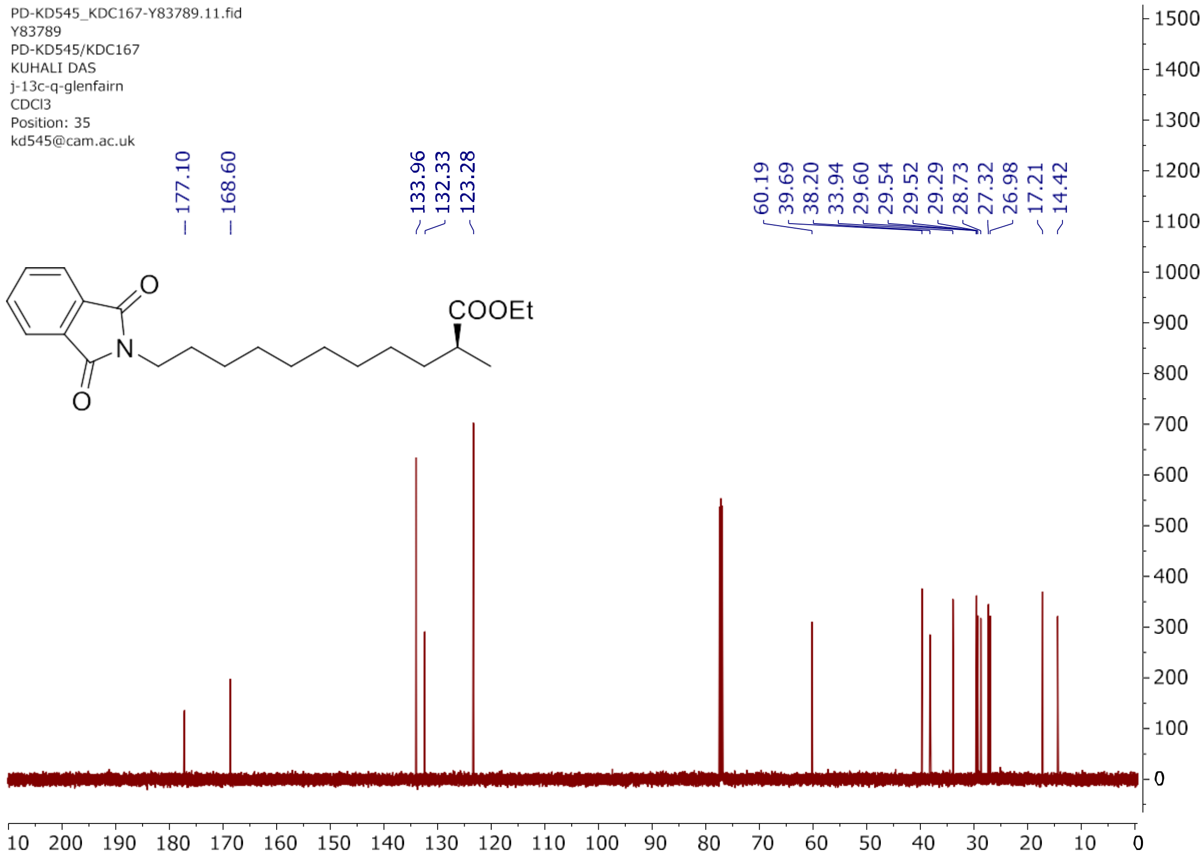
PD-KD545_KDC167-Y83789.11.fid
Y83789
PD-KD545/KDC167
KUHALI DAS
j-13c-q-glenfairn
CDCl3
Position: 35
kd545@cam.ac.uk
177.10 168.60 133.96 132.33 123.28 60.19 39.69 38.20 33.94 29.60 29.54 29.52 29.29 28.73 27.32 26.98 17.21 14.42
COOEt
N
O
O

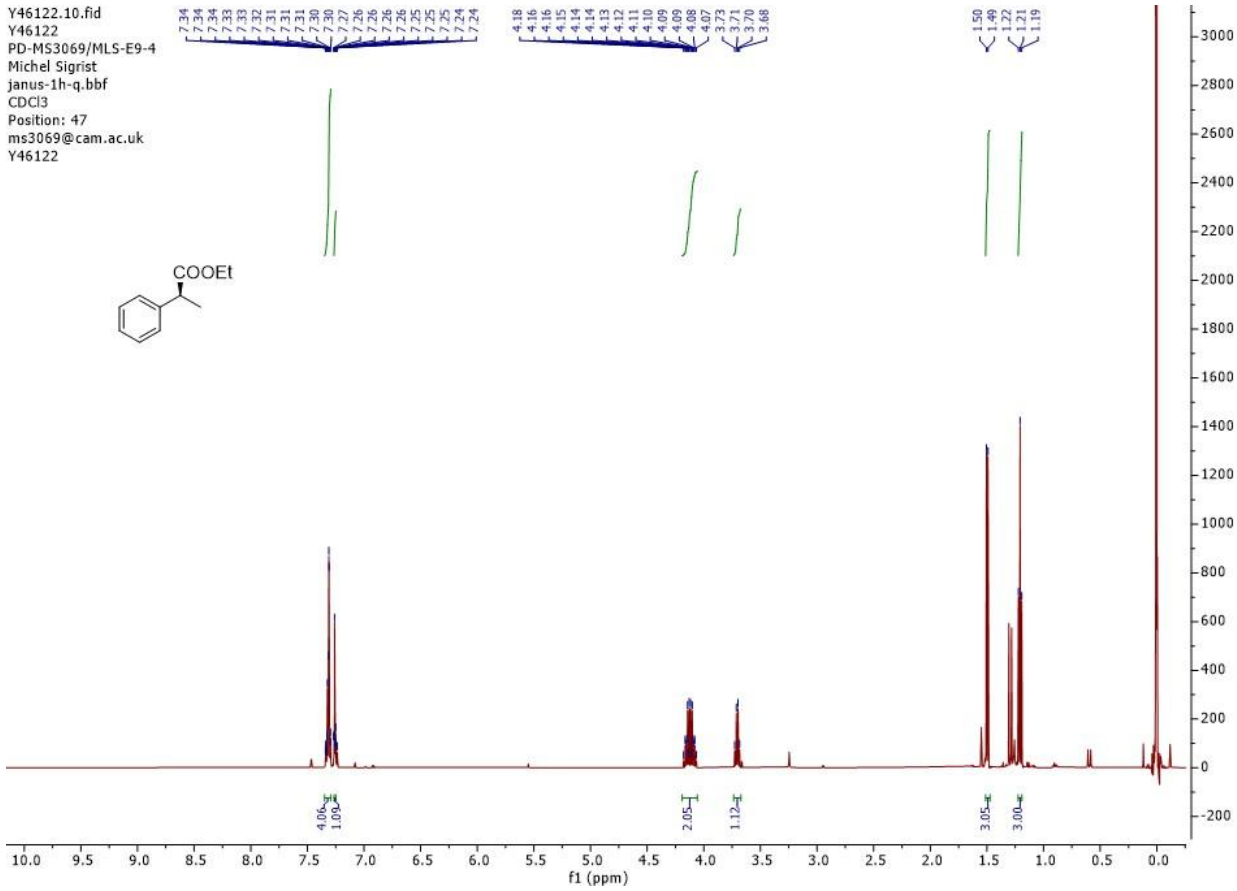
Y46122.10.fid
Y46122
PD-MS3069/MLS-E9-4
Michel Sigrist
janus-1h-q.bbf
CDCl3
Position: 47
ms3069@cam.ac.uk
Y46122
COOEt
f1 (ppm)

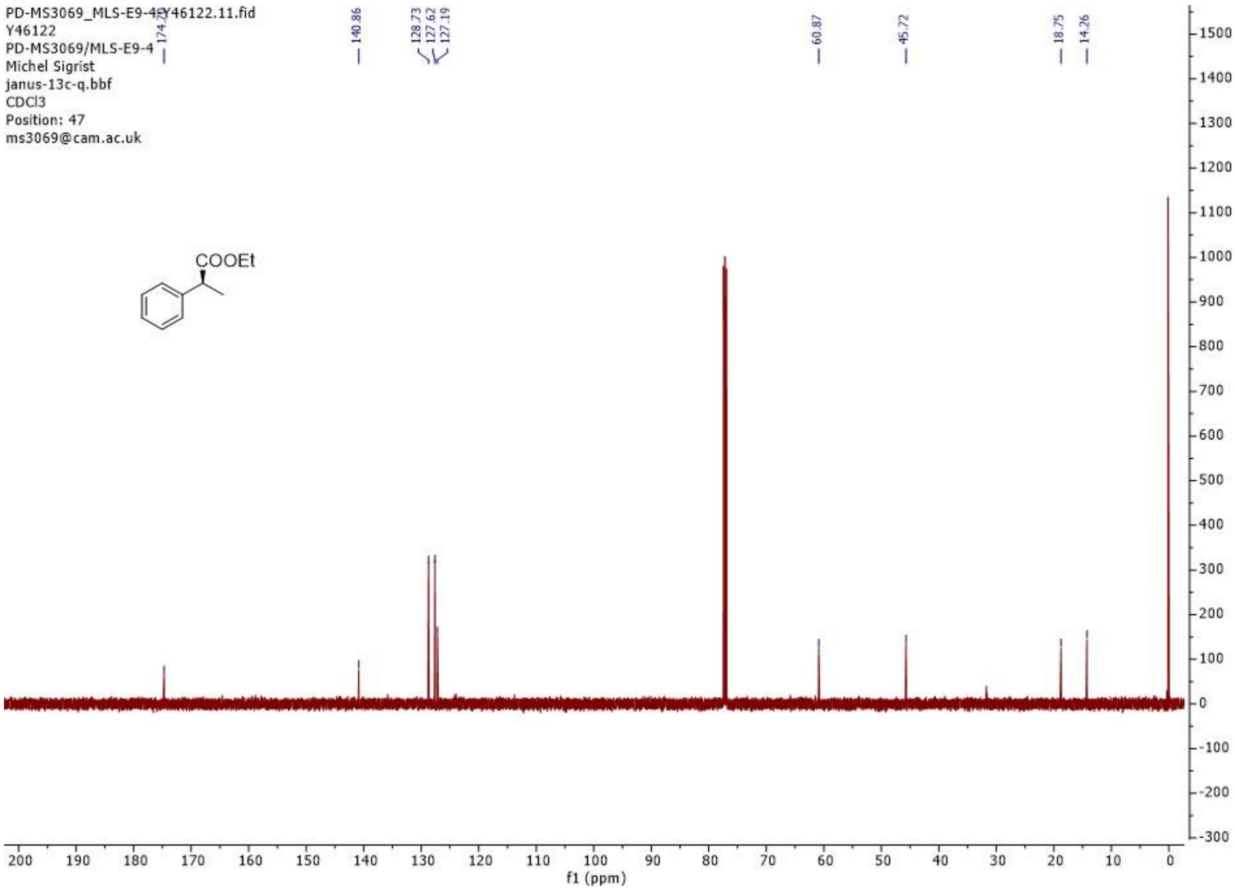
PD-MS3069_MLS-E9-4 Y46122.11.fid
Y46122
PD-MS3069/MLS-E9-4
Michel Sigrist
janus-13c-q.bbf
CDCl3
Position: 47
ms3069@cam.ac.uk
140.86
128.73
127.62
127.19
60.87
45.72
18.75
14.26
COOEt
f1 (ppm)

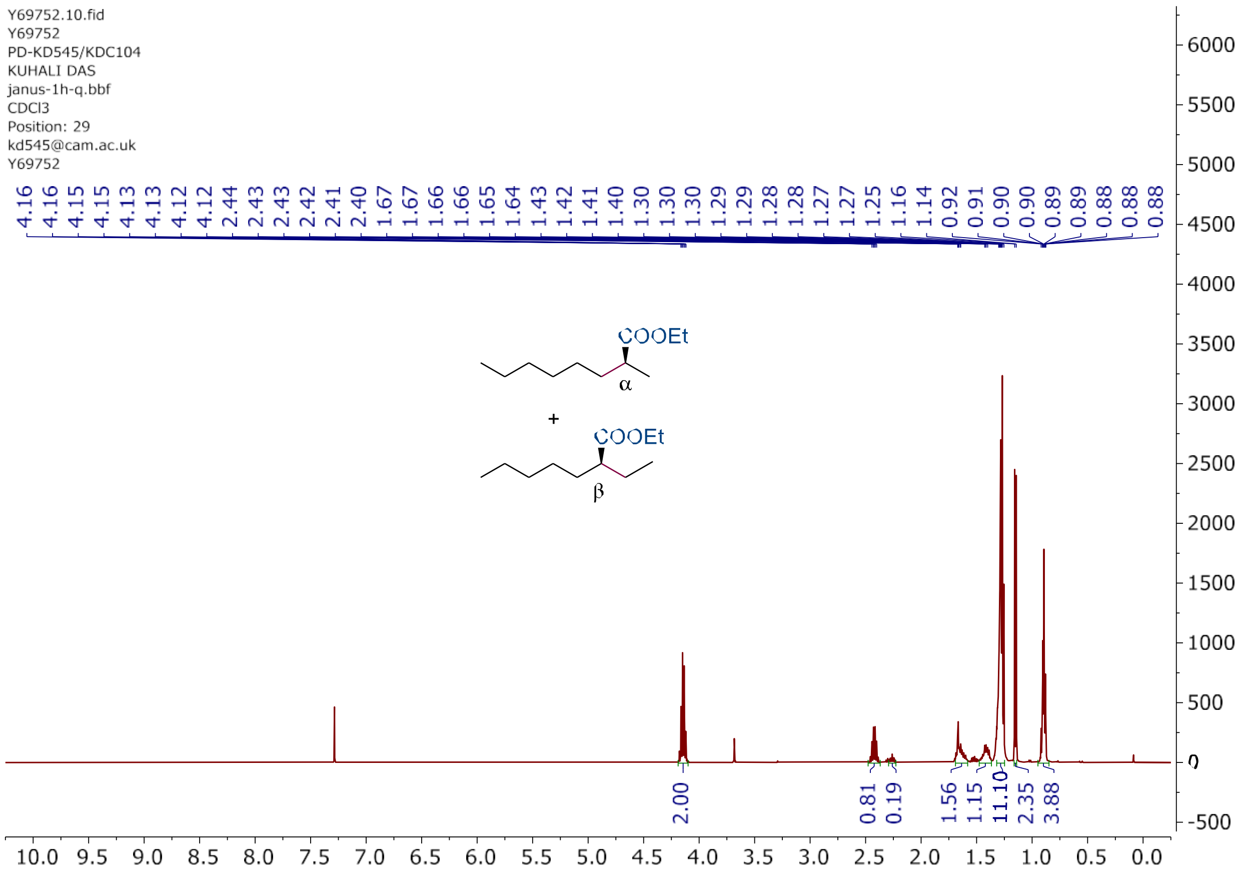
Y69752.10.fid
Y69752
PD-KD545/KDC104
KUHALI DAS
janus-1h-q.bbf
CDCl3
Position: 29
kd545@cam.ac.uk
Y69752
4.16 4.16 4.15 4.15 4.13 4.13 4.12 4.12 2.44 2.43 2.43 2.42 2.41 2.40 1.67 1.67 1.66 1.66 1.65 1.64 1.43 1.42 1.41 1.40 1.30 1.30 1.30 1.29 1.29 1.28 1.28 1.27 1.27 1.25 1.16 1.14 0.92 0.91 0.90 0.90 0.89 0.89 0.88 0.88 0.88
COOEt
α
+
COOEt
β
2.00
0.81
0.19
1.56
1.15
11.10
2.35
3.88

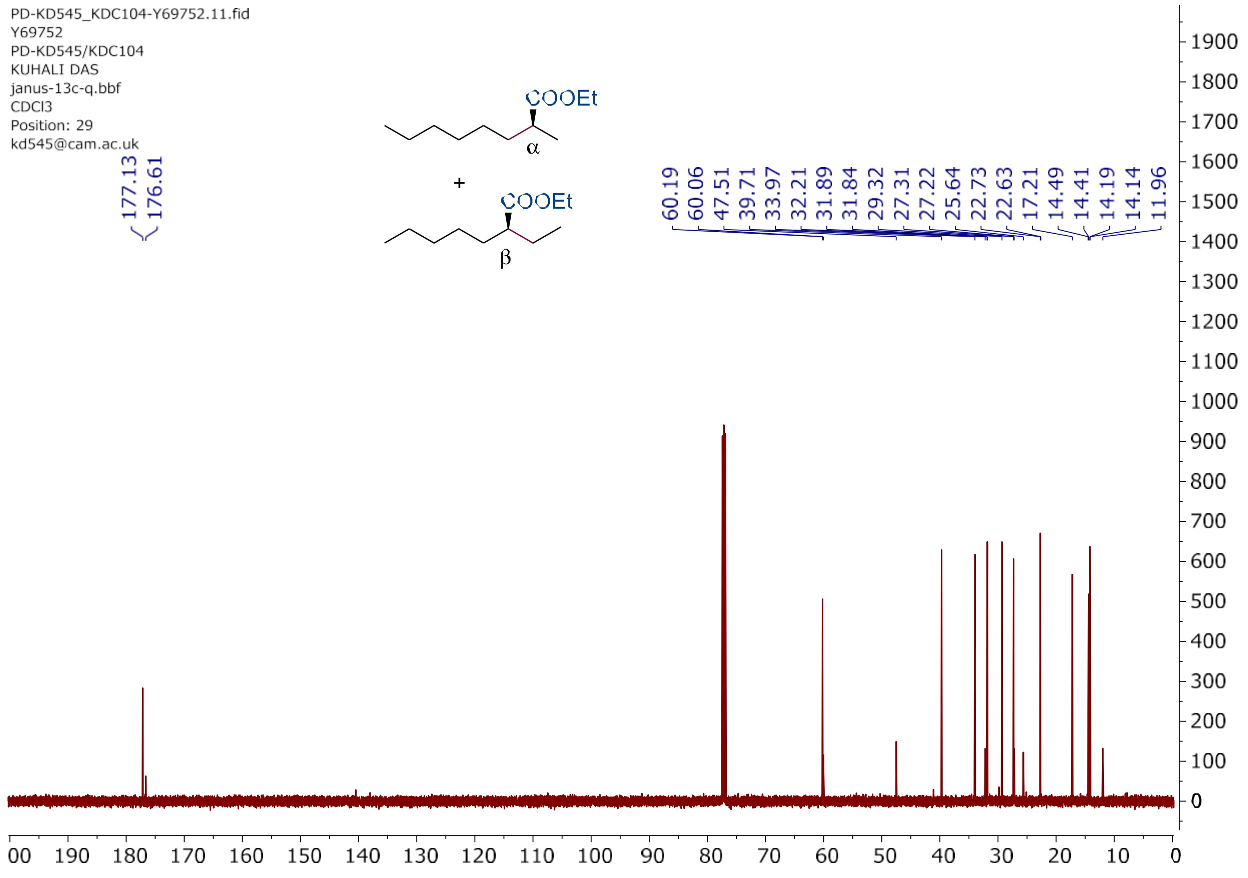
PD-KD545_KDC104-Y69752.11.fid
Y69752
PD-KD545/KDC104
KUHALI DAS
janus-13c-q.bbf
CDCl3
Position: 29
kd545@cam.ac.uk
177.13
176.61
COOEt
α
+
COOEt
β
60.19 60.06 47.51 39.71 33.97 32.21 31.89 31.84 29.32 27.31 27.22 25.64 22.73 22.63 17.21 14.49 14.41 14.19 14.14 11.96

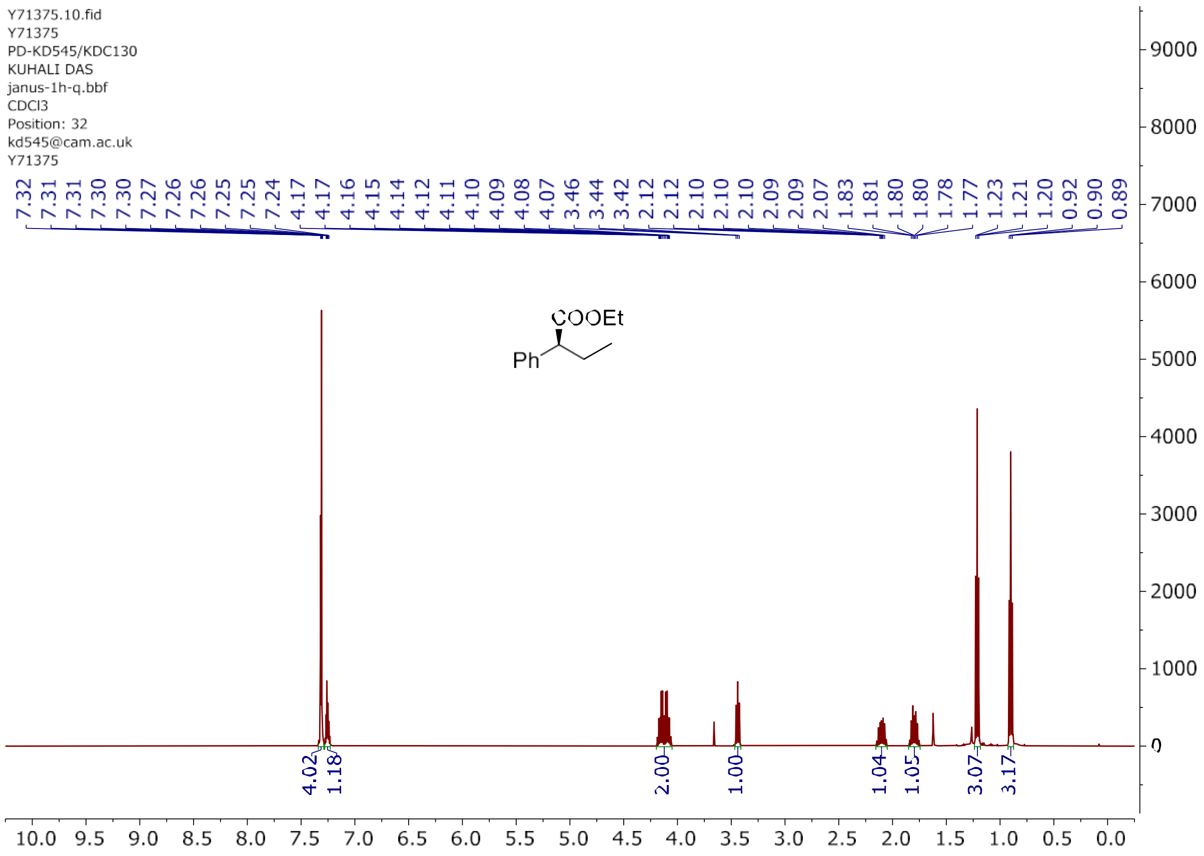
Y71375.10.fid
Y71375
PD-KD545/KDC130
KUHALI DAS
janus-1h-q.bbf
CDCl3
Position: 32
kd545@cam.ac.uk
Y71375
7.32 7.31 7.31 7.30 7.30 7.27 7.26 7.26 7.25 7.25 7.24 4.17 4.17 4.16 4.15 4.14 4.12 4.11 4.10 4.09 4.08 4.07 3.46 3.44 3.42 2.12 2.12 2.10 2.10 2.10 2.09 2.09 2.07 1.83 1.81 1.80 1.80 1.78 1.77 1.23 1.21 1.20 0.92 0.90 0.89
COOEt
Ph
4.02 1.18 2.00 1.00 1.04 1.05 3.07 3.17

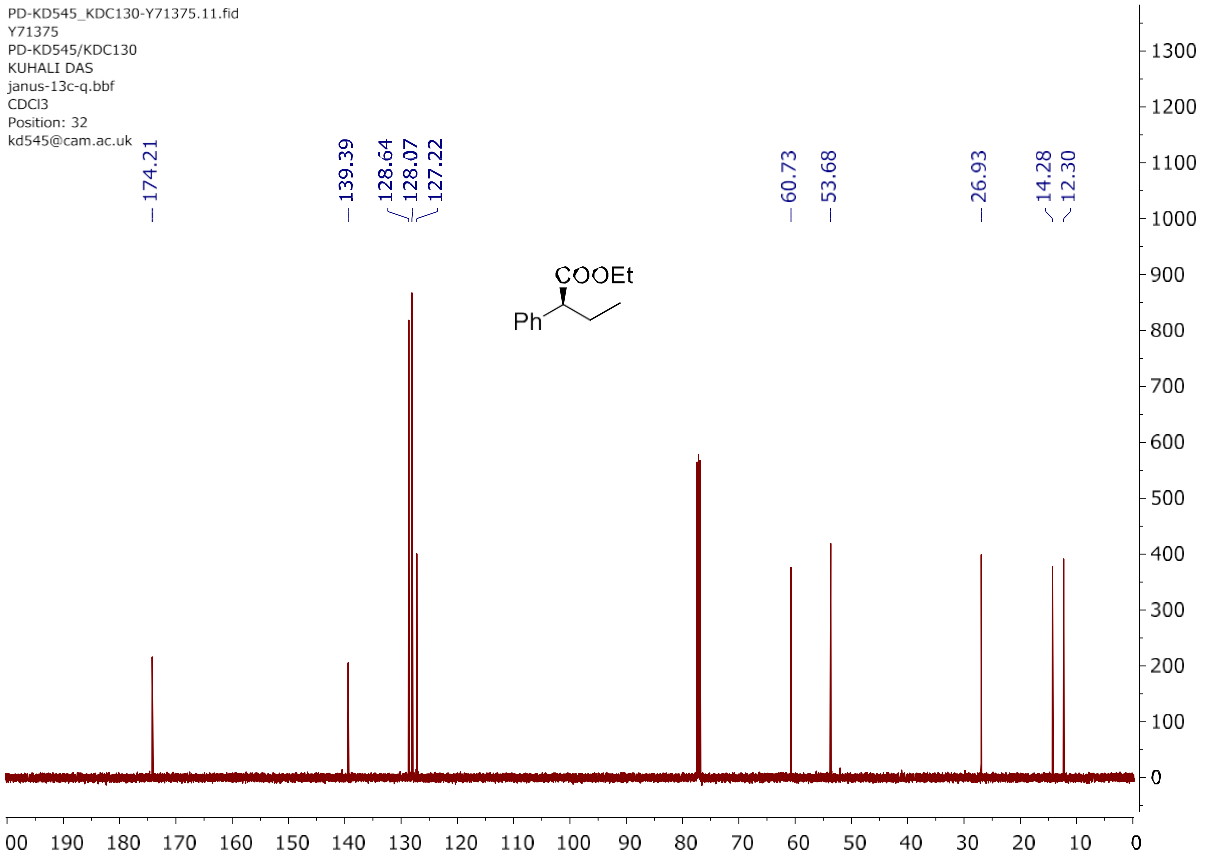
PD-KD545_KDC130-Y71375.11.fid
Y71375
PD-KD545/KDC130
KUHALI DAS
janus-13c-q.bbf
CDCl3
Position: 32
kd545@cam.ac.uk
174.21 139.39 128.64 128.07 127.22 60.73 53.68 26.93 14.28 12.30
COOEt
Ph

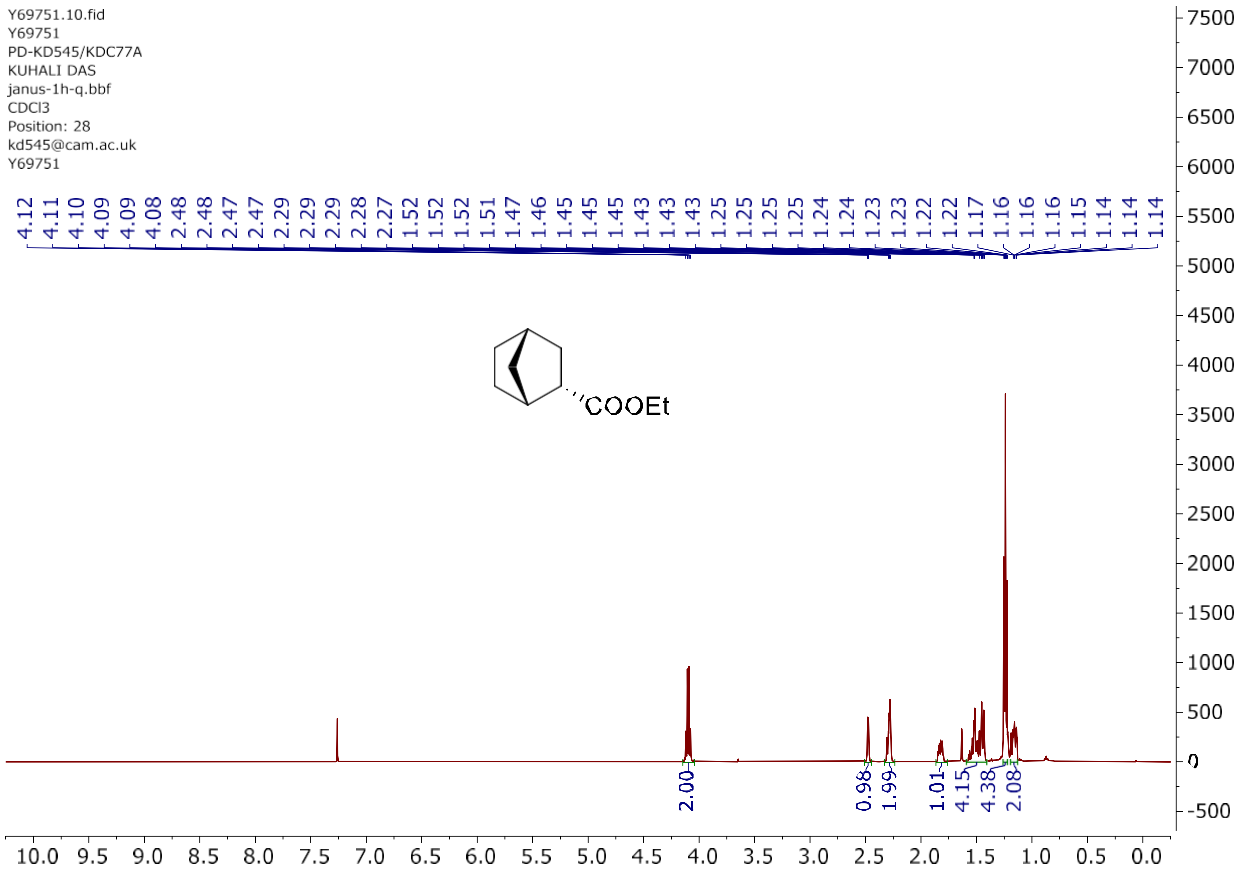
Y69751.10.fid
Y69751
PD-KD545/KDC77A
KUHALI DAS
janus-1h-q.bbf
CDCl3
Position: 28
kd545@cam.ac.uk
Y69751
4.12 4.11 4.10 4.09 4.09 4.08 2.48 2.48 2.47 2.47 2.29 2.29 2.29 2.28 2.27 1.52 1.52 1.52 1.51 1.47 1.46 1.45 1.45 1.45 1.43 1.43 1.43 1.25 1.25 1.25 1.25 1.24 1.24 1.23 1.23 1.22 1.22 1.17 1.16 1.16 1.16 1.15 1.14 1.14 1.14
COOEt
2.00 0.98 1.99 1.01 4.15 4.38 2.08
10.0 9.5 9.0 8.5 8.0 7.5 7.0 6.5 6.0 5.5 5.0 4.5 4.0 3.5 3.0 2.5 2.0 1.5 1.0 0.5 0.0

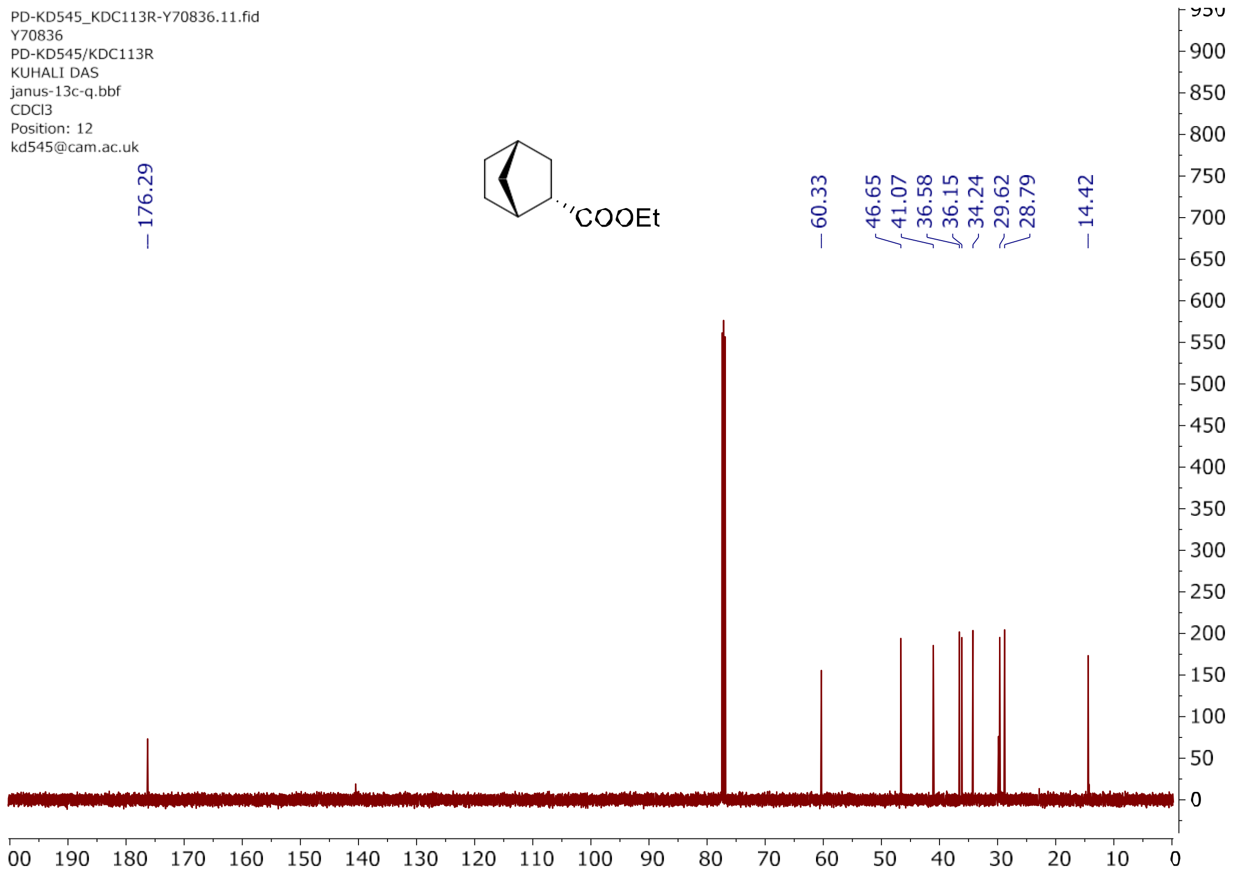
PD-KD545_KDC113R-Y70836.11.fid
Y70836
PD-KD545/KDC113R
KUHALI DAS
janus-13c-q.bbf
CDCl3
Position: 12
kd545@cam.ac.uk
176.29
COOEt
60.33
46.65 41.07 36.58 36.15 34.24 29.62 28.79
14.42
200 190 180 170 160 150 140 130 120 110 100 90 80 70 60 50 40 30 20 10 0

Y72508.10.fid
Y72508
PD-KD545/KDC155
KUHALI DAS
janus-1h-q.bbf
CDCl3
Position: 5
kd545@cam.ac.uk
Y72508

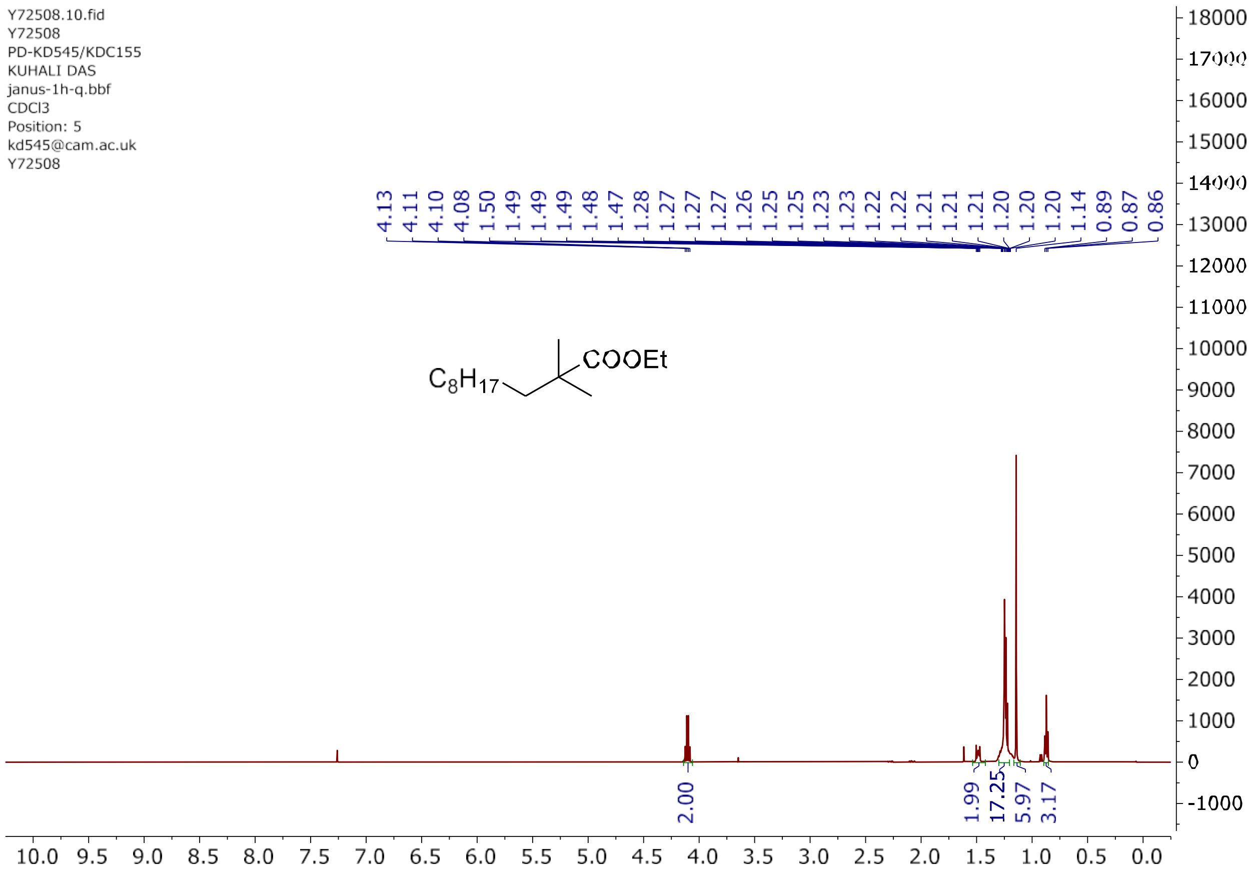

PD-KD545_KDC155-Y72508.11.fid
Y72508
PD-KD545/KDC155
KUHALI DAS
janus-13c-q.bbf
CDCl3
Position: 5
kd545@cam.ac.uk

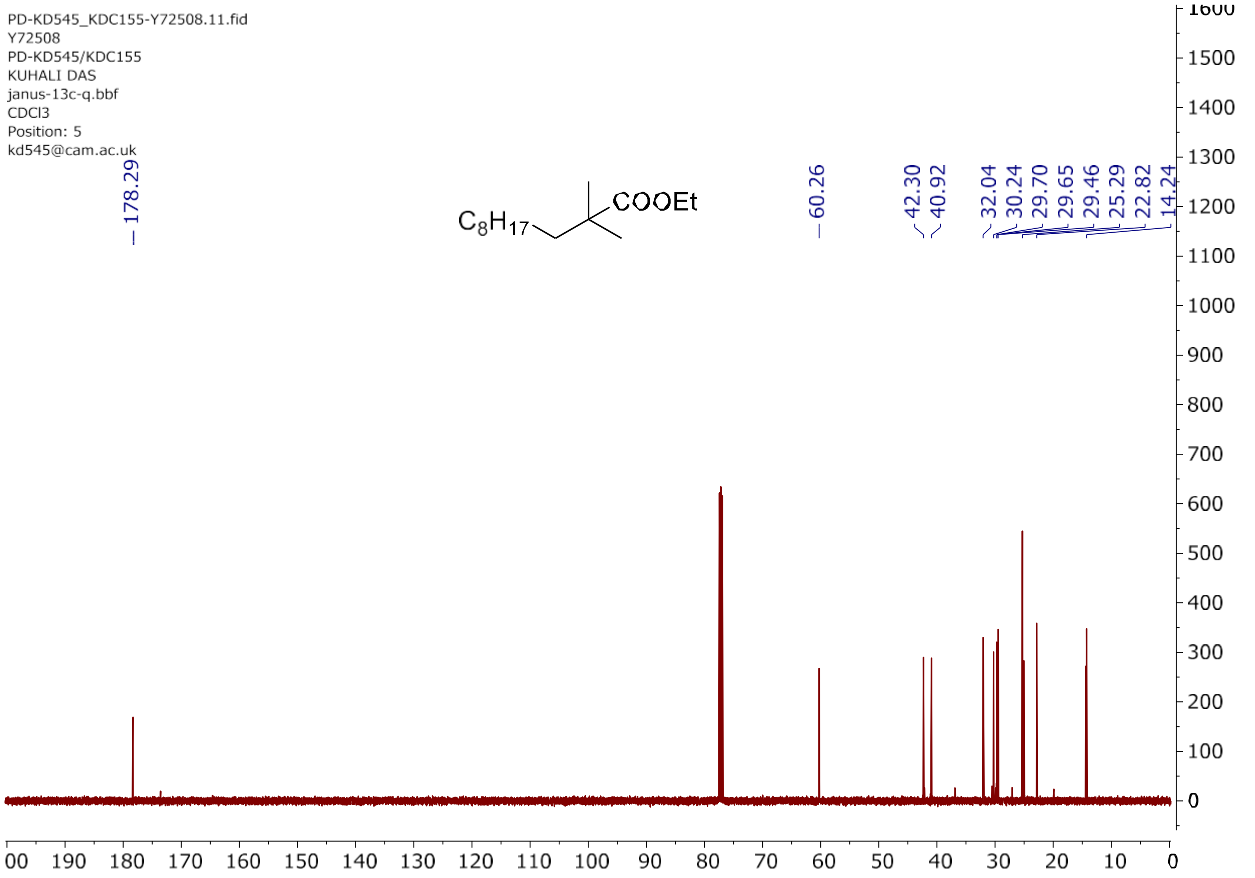

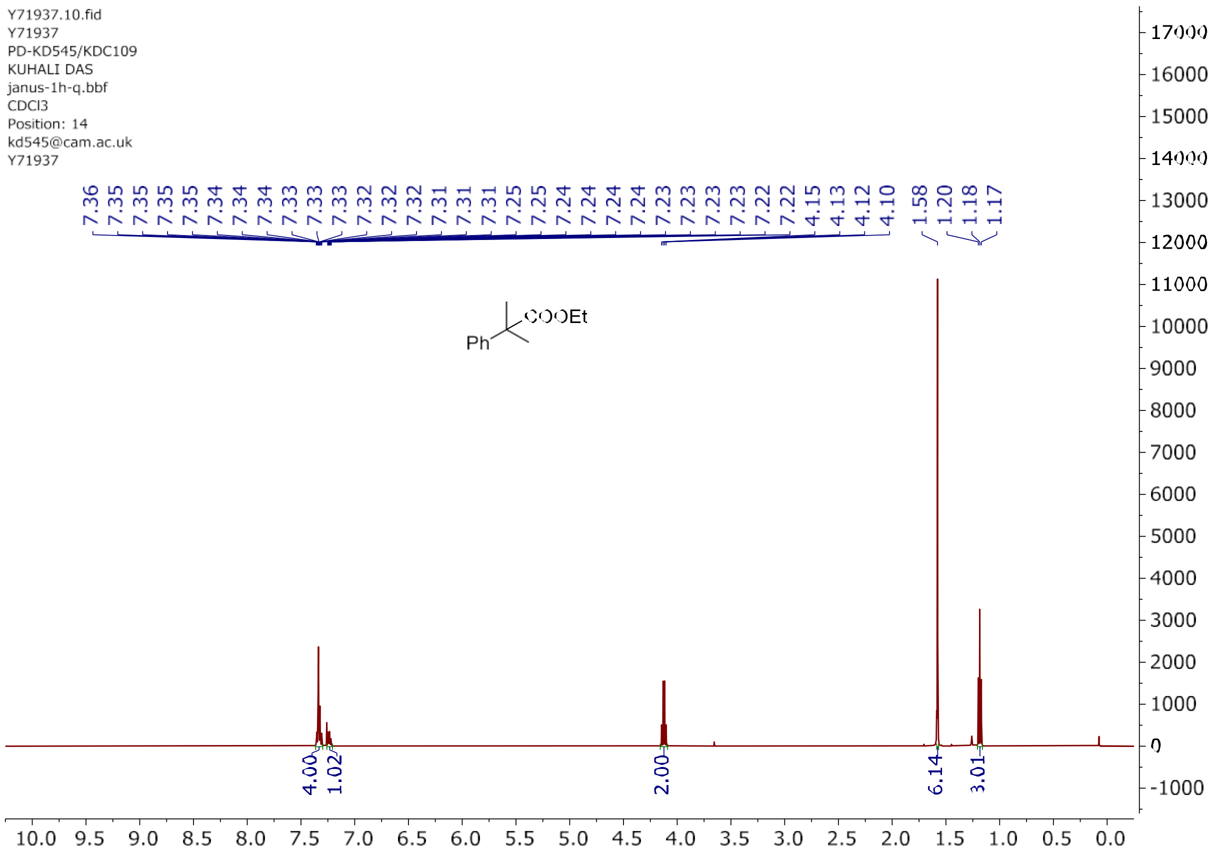

Y71937.10.fid
Y71937
PD-KD545/KDC109
KUHALI DAS
janus-1h-q.bbf
CDCl3
Position: 14
kd545@cam.ac.uk
Y71937
7.36 7.35 7.35 7.35 7.35 7.34 7.34 7.34 7.33 7.33 7.33 7.32 7.32 7.32 7.31 7.31 7.31 7.25 7.25 7.24 7.24 7.24 7.24 7.23 7.23 7.23 7.23 7.22 7.22 4.15 4.13 4.12 4.10 1.58 1.20 1.18 1.17
COOEt
Ph
4.00 1.02 2.00 6.14 3.01

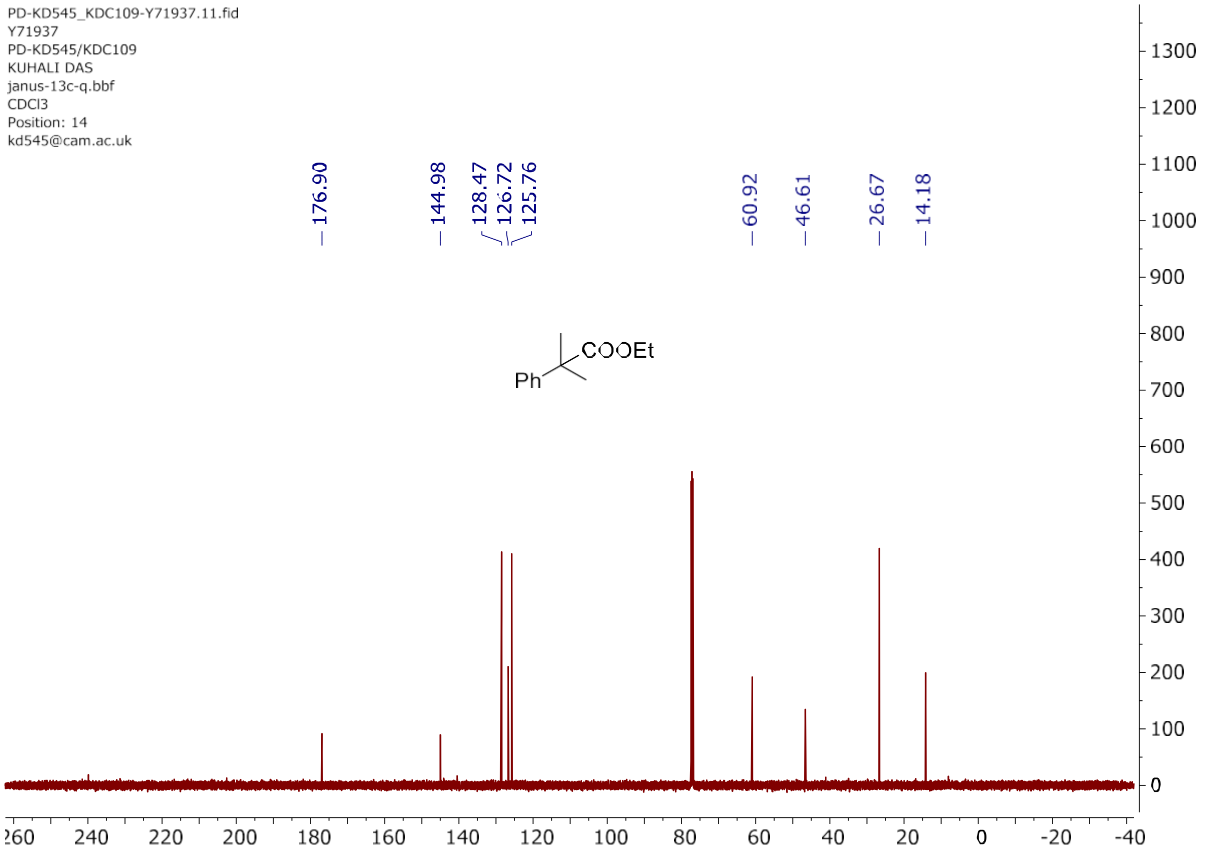

PD-KD545_KDC109-Y71937.11.fid
Y71937
PD-KD545/KDC109
KUHALI DAS
janus-13c-q.bbf
CDCl3
Position: 14
kd545@cam.ac.uk
176.90
144.98
128.47 126.72 125.76
60.92
46.61
26.67
14.18
COOEt
Ph

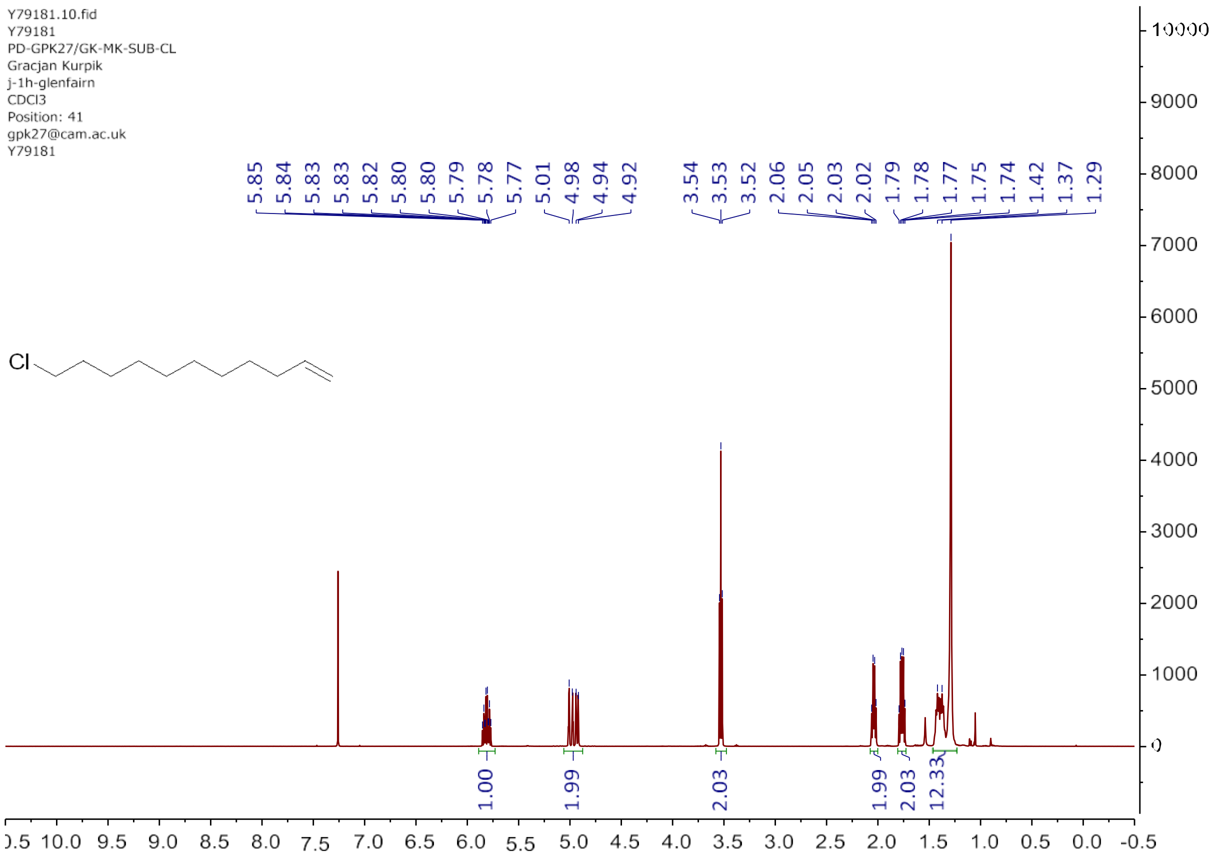
Y79181.10.fid
Y79181
PD-GPK27/GK-MK-SUB-CL
Gracjan Kurpik
j-1h-glenfairn
CDCl3
Position: 41
gpk27@cam.ac.uk
Y79181
5.85 5.84 5.83 5.83 5.82 5.80 5.80 5.79 5.78 5.77 5.01 4.98 4.94 4.92 3.54 3.53 3.52 2.06 2.05 2.03 2.02 1.79 1.78 1.77 1.75 1.74 1.42 1.37 1.29
Cl
1.00 1.99 2.03 1.99 2.03 12.33

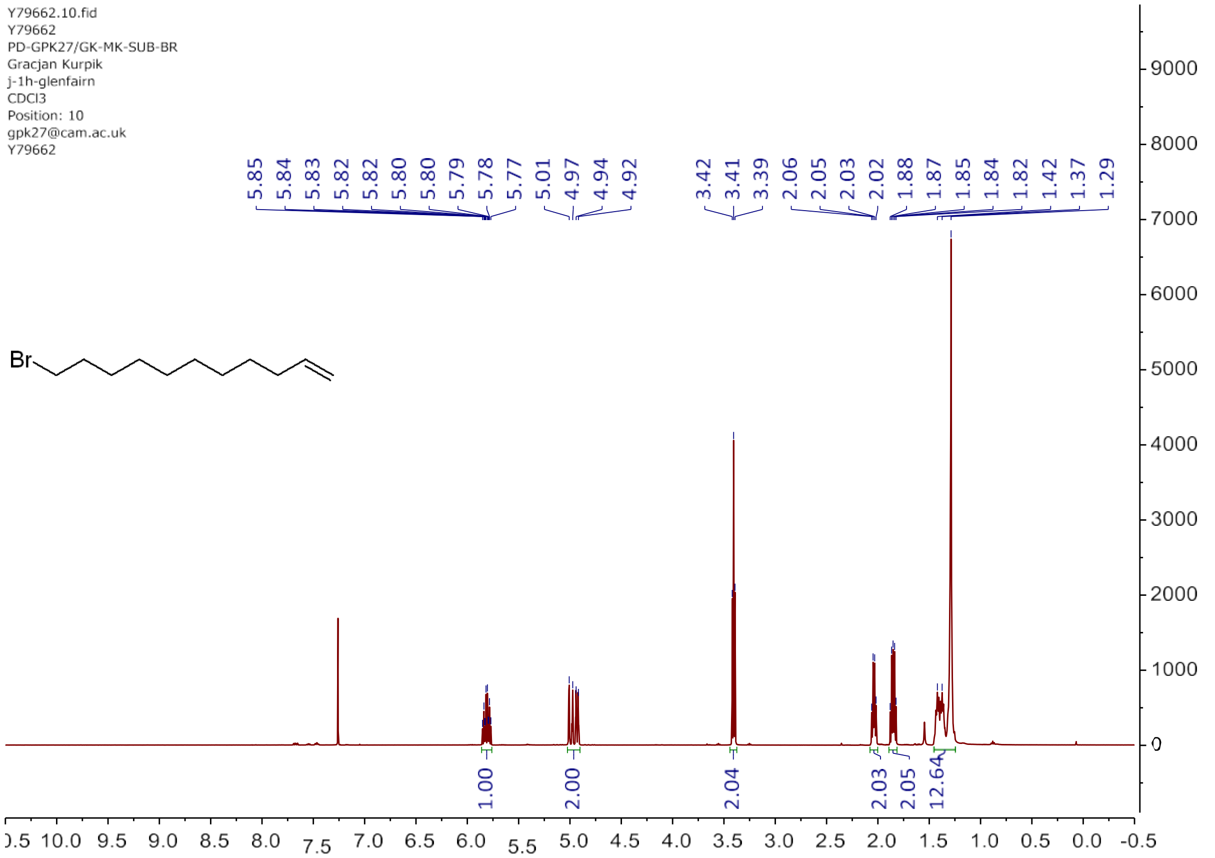
Y79662.10.fid
Y79662
PD-GPK27/GK-MK-SUB-BR
Gracjan Kurpik
j-1h-glenfairn
CDCl3
Position: 10
gpk27@cam.ac.uk
Y79662
5.85 5.84 5.83 5.82 5.82 5.80 5.80 5.79 5.78 5.77 5.01 4.97 4.94 4.92 3.42 3.41 3.39 2.06 2.05 2.03 2.02 1.88 1.87 1.85 1.84 1.82 1.42 1.37 1.29
Br
1.00 2.00 2.04 2.03 2.05 12.64

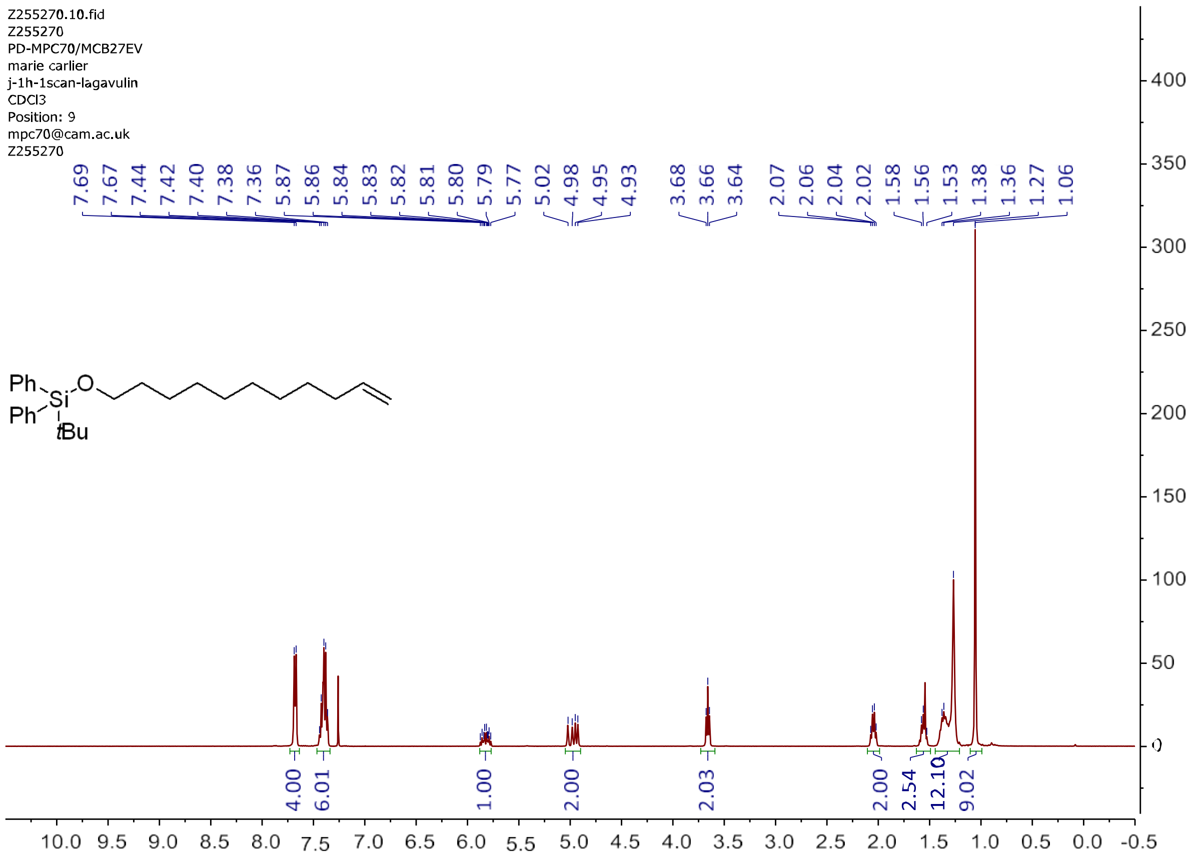
Z255270.10.fid
Z255270
PD-MPC70/MCB27EV
marie carlier
j-1h-1scan-lagavulin
CDCl3
Position: 9
mpc70@cam.ac.uk
Z255270
7.69 7.67 7.44 7.42 7.40 7.38 7.36 5.87 5.86 5.84 5.83 5.82 5.81 5.80 5.79 5.77 5.02 4.98 4.95 4.93 3.68 3.66 3.64 2.07 2.06 2.04 2.02 1.58 1.56 1.53 1.38 1.36 1.27 1.06
Ph
Ph
Si
O
tBu
4.00 6.01 1.00 2.00 2.03 2.00 2.54 12.10 9.02

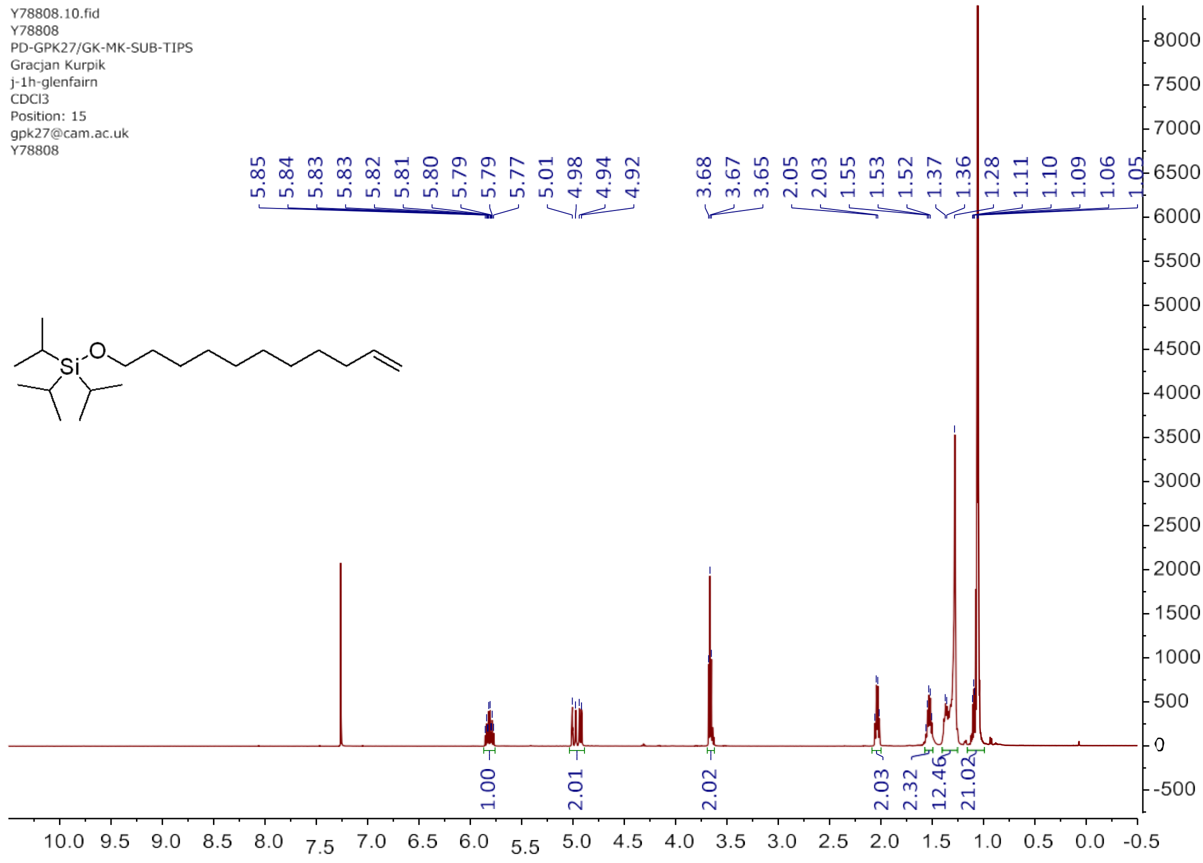
Y78808.10.fid
Y78808
PD-GPK27/GK-MK-SUB-TIPS
Gracjan Kurpik
j-1h-glenfairn
CDCl3
Position: 15
gpk27@cam.ac.uk
Y78808
5.85 5.84 5.83 5.83 5.82 5.81 5.80 5.79 5.79 5.77 5.01 4.98 4.94 4.92 3.68 3.67 3.65 2.05 2.03 1.55 1.53 1.52 1.37 1.36 1.28 1.11 1.10 1.09 1.06 1.05
Si
O
1.00 2.01 2.02 2.03 2.32 12.46 21.02

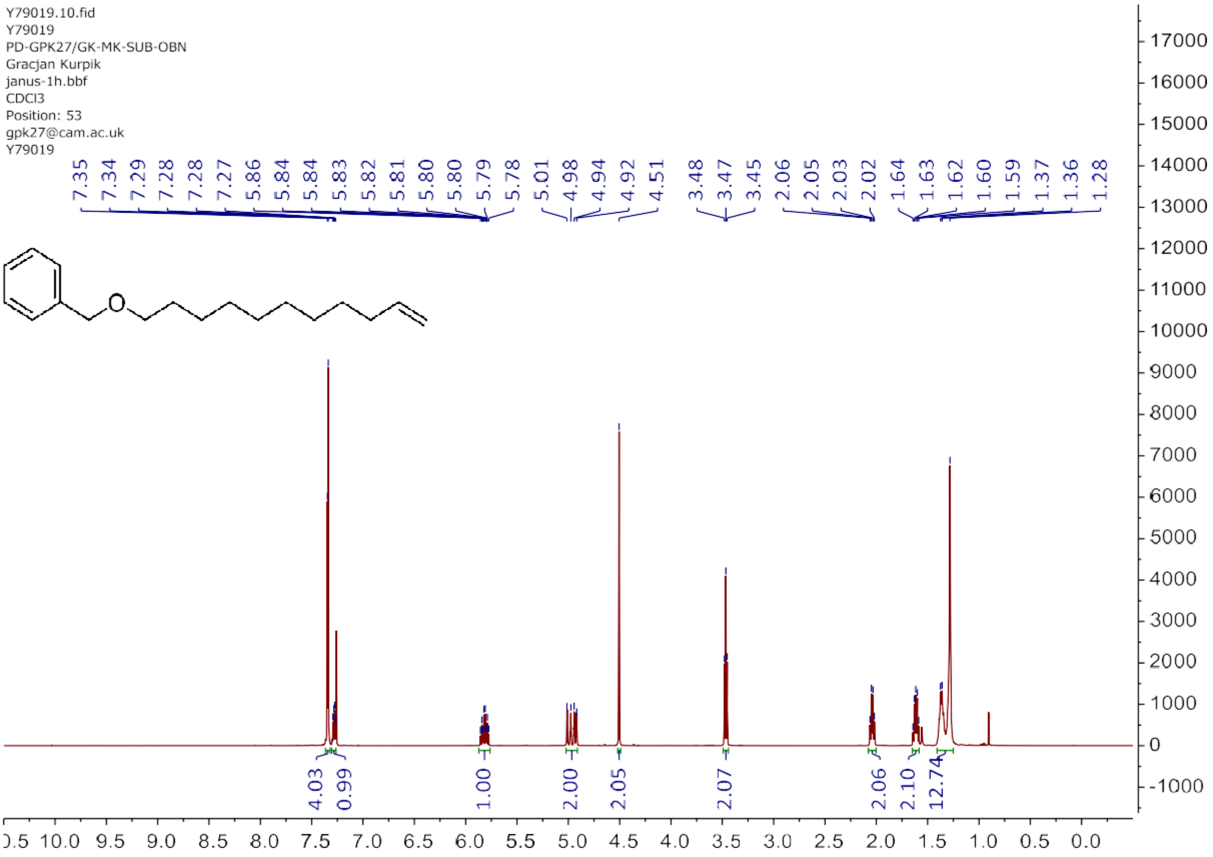
Y79019.10.fid
Y79019
PD-GPK27/GK-MK-SUB-OBN
Gracjan Kurpik
janus-1h.bbf
CDCl3
Position: 53
gpk27@cam.ac.uk
Y79019
7.35 7.34 7.29 7.28 7.28 7.27 5.86 5.84 5.84 5.83 5.82 5.81 5.80 5.80 5.79 5.78 5.01 4.98 4.94 4.92 4.51 3.48 3.47 3.45 2.06 2.05 2.03 2.02 1.64 1.63 1.62 1.60 1.59 1.37 1.36 1.28
O
4.03 0.99 1.00 2.00 2.05 2.07 2.06 2.10 12.74

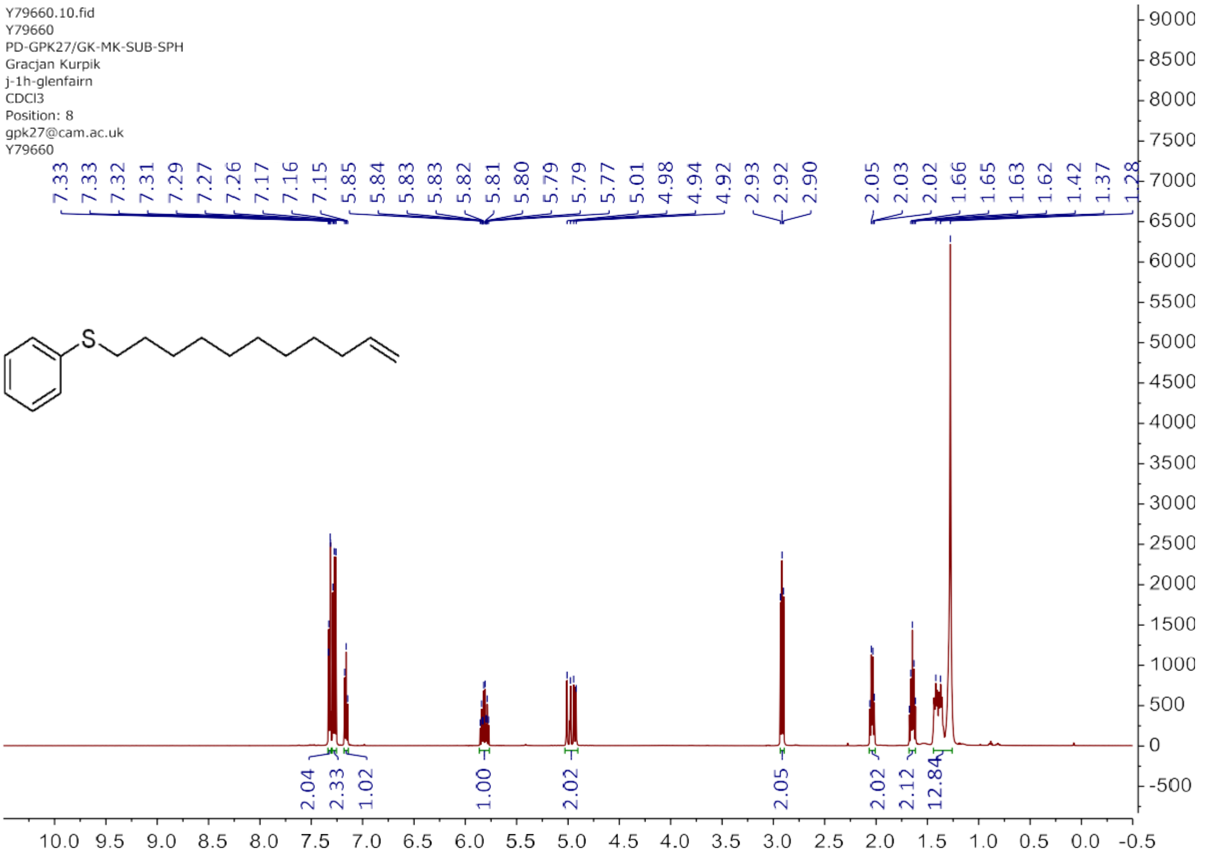
Y79660.10.fid
Y79660
PD-GPK27/GK-MK-SUB-SPH
Gracjan Kurpik
j-1h-glenfairn
CDCl3
Position: 8
gpk27@cam.ac.uk
Y79660
7.33 7.33 7.32 7.31 7.29 7.27 7.26 7.17 7.16 7.15 5.85 5.84 5.83 5.83 5.82 5.81 5.80 5.79 5.79 5.77 5.01 4.98 4.94 4.92 2.93 2.92 2.90 2.05 2.03 2.02 1.66 1.65 1.63 1.62 1.42 1.37 1.28
S
2.04 2.33 1.02 1.00 2.02 2.05 2.02 2.12 12.84

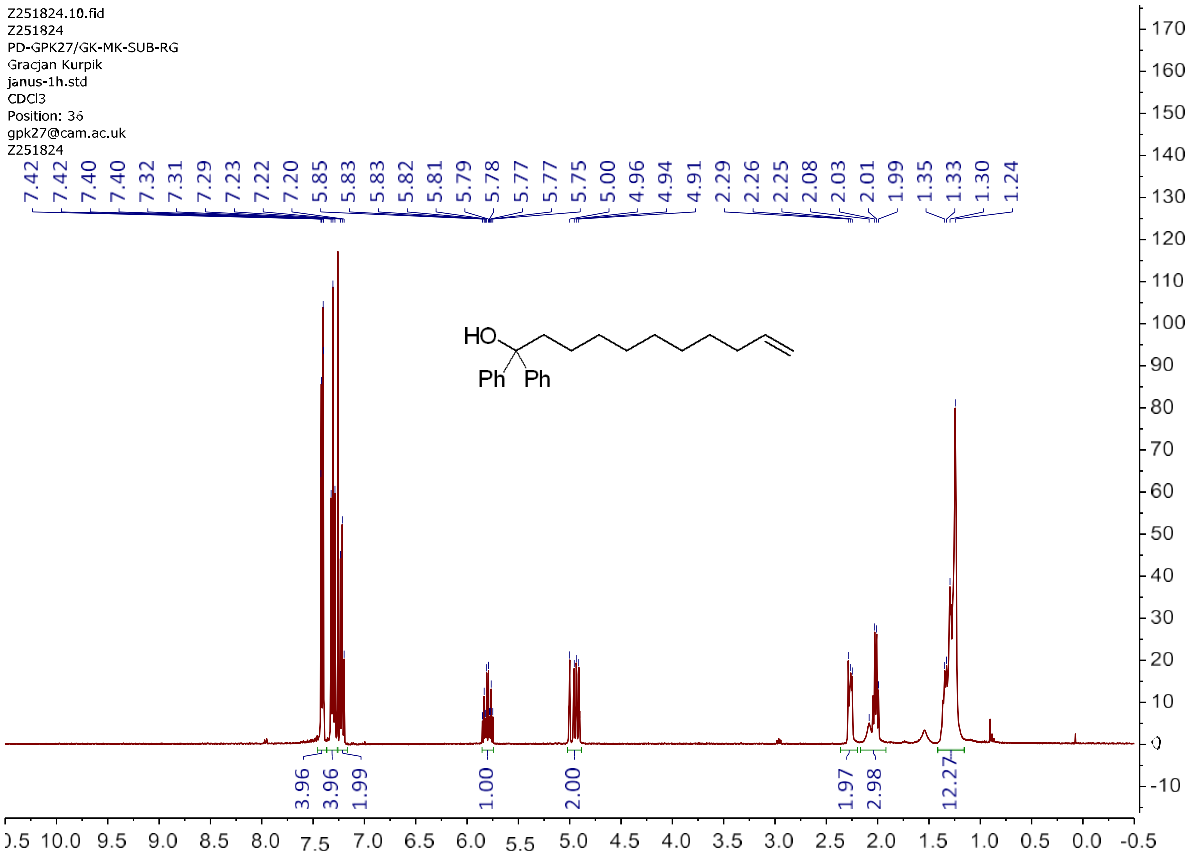
Z251824.10.fid
Z251824
PD-GPK27/GK-MK-SUB-RG
Gracjan Kurpik
janus-1h.std
CDCl3
Position: 36
gpk27@cam.ac.uk
Z251824
7.42 7.42 7.40 7.40 7.32 7.31 7.29 7.23 7.22 7.20 5.85 5.83 5.83 5.82 5.81 5.79 5.78 5.77 5.77 5.75 5.00 4.96 4.94 4.91 2.29 2.26 2.25 2.08 2.03 2.01 1.99 1.35 1.33 1.30 1.24
HO
Ph Ph
3.96 3.96 1.99 1.00 2.00 1.97 2.98 12.27

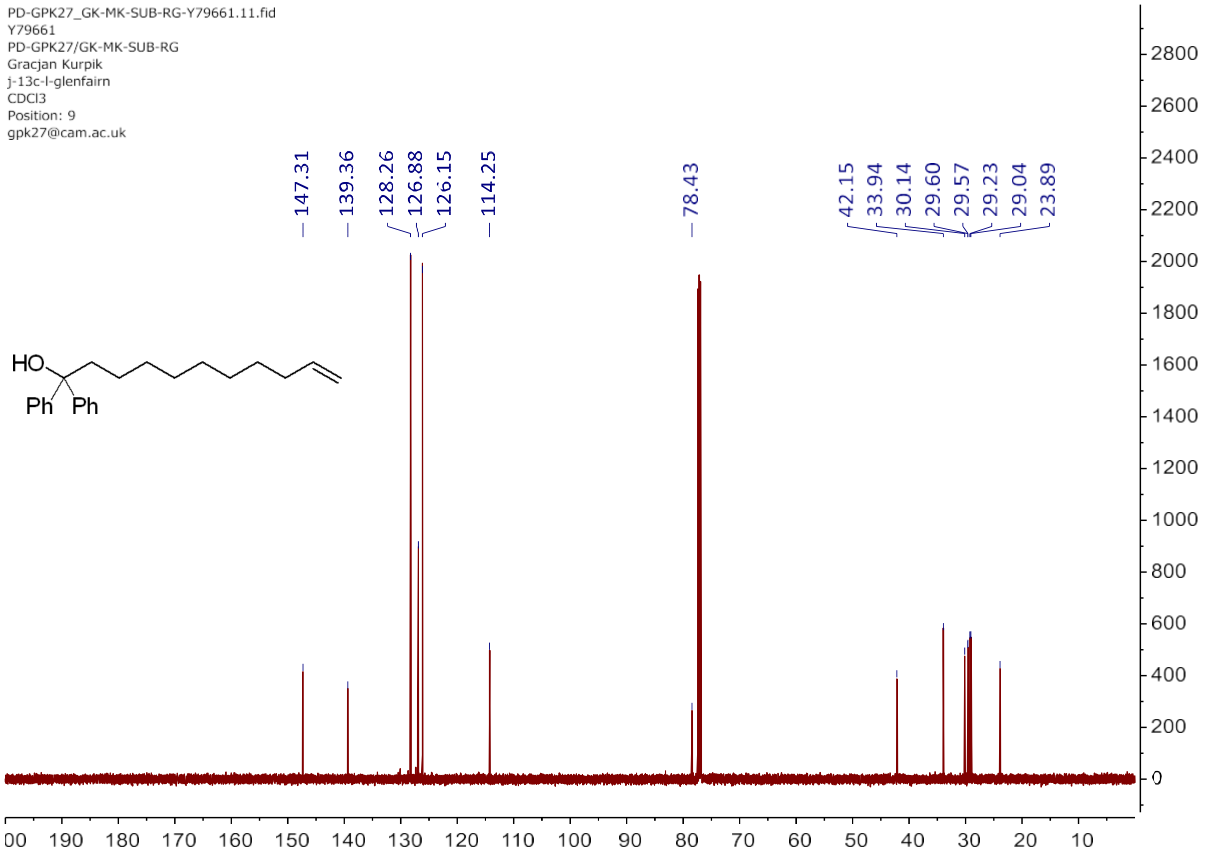
PD-GPK27_GK-MK-SUB-RG-Y79661.11.fid
Y79661
PD-GPK27/GK-MK-SUB-RG
Gracjan Kurpik
j-13c-l-glenfairn
CDCl3
Position: 9
gpk27@cam.ac.uk
147.31 139.36 128.26 126.88 126.15 114.25 78.43 42.15 33.94 30.14 29.60 29.57 29.23 29.04 23.89
HO
Ph Ph

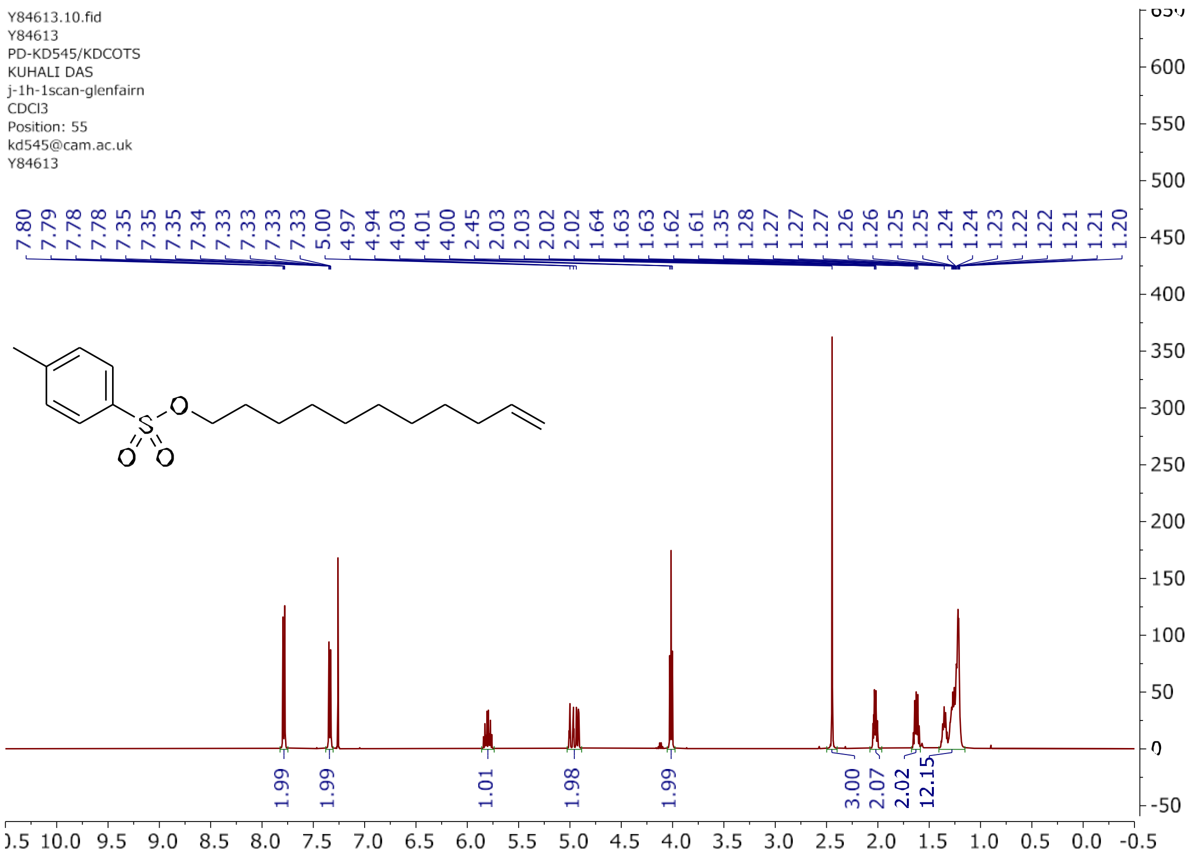
Y84613.10.fid
Y84613
PD-KD545/KDCOTS
KUHALI DAS
j-1h-1scan-glenfairn
CDCl3
Position: 55
kd545@cam.ac.uk
Y84613

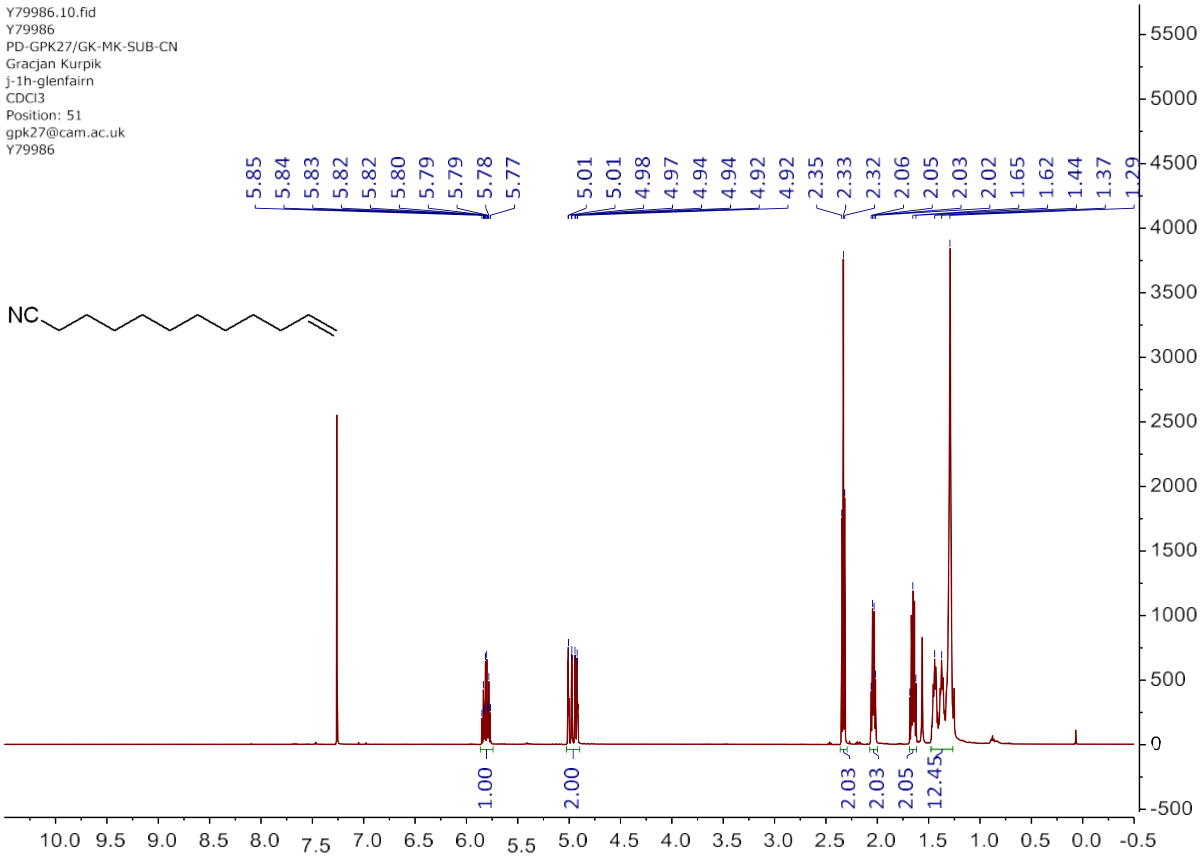
Y79986.10.fid
Y79986
PD-GPK27/GK-MK-SUB-CN
Gracjan Kurpik
j-1h-glenfairn
CDCl3
Position: 51
gpk27@cam.ac.uk
Y79986
NC

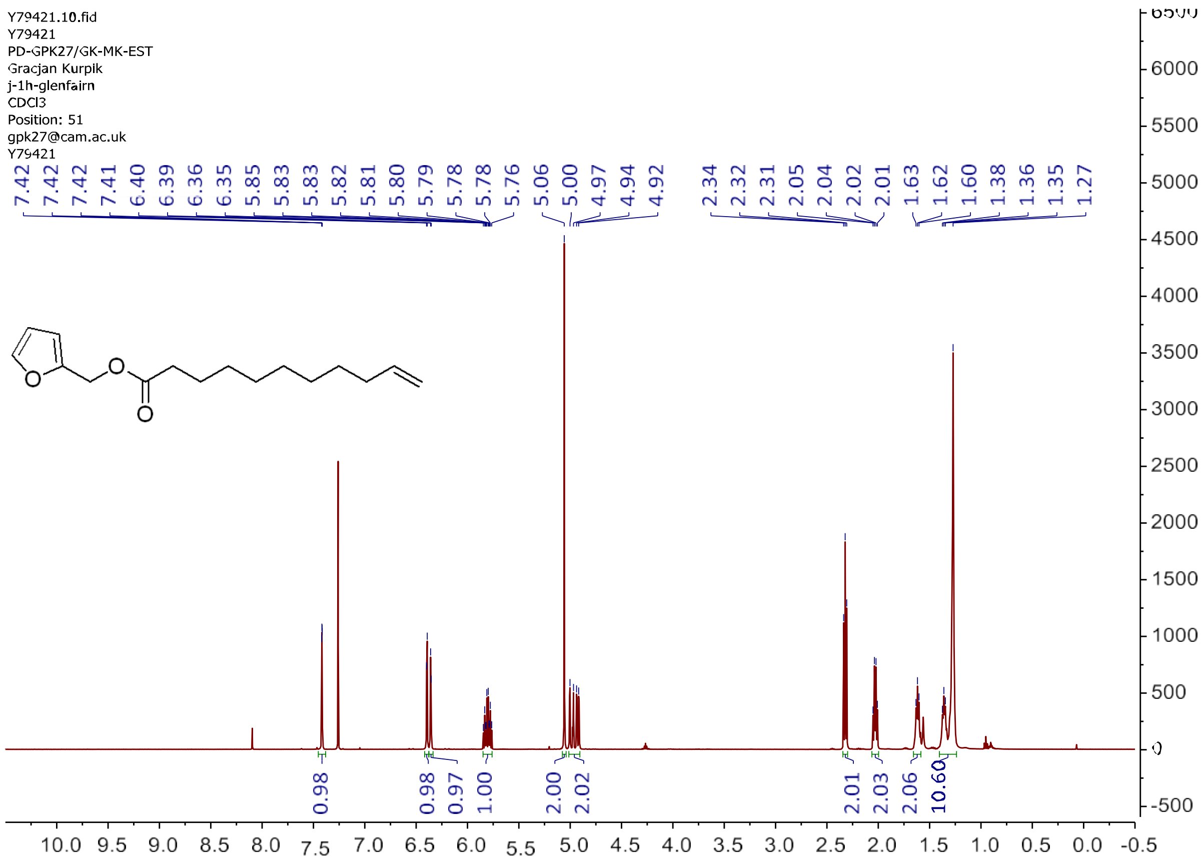
Y79421.10.fid
Y79421
PD-GPK27/GK-MK-EST
Gracjan Kurpik
j-1h-glenfairn
CDCl3
Position: 51
gpk27@cam.ac.uk
Y79421
7.42
7.42
7.42
7.41
6.40
6.39
6.36
6.35
5.85
5.83
5.83
5.82
5.81
5.80
5.79
5.78
5.78
5.76
5.06
5.00
4.97
4.94
4.92
2.34
2.32
2.31
2.05
2.04
2.02
2.01
1.63
1.62
1.60
1.38
1.36
1.35
1.27
0.98
0.98
0.97
1.00
2.00
2.02
2.01
2.03
2.06
10.60

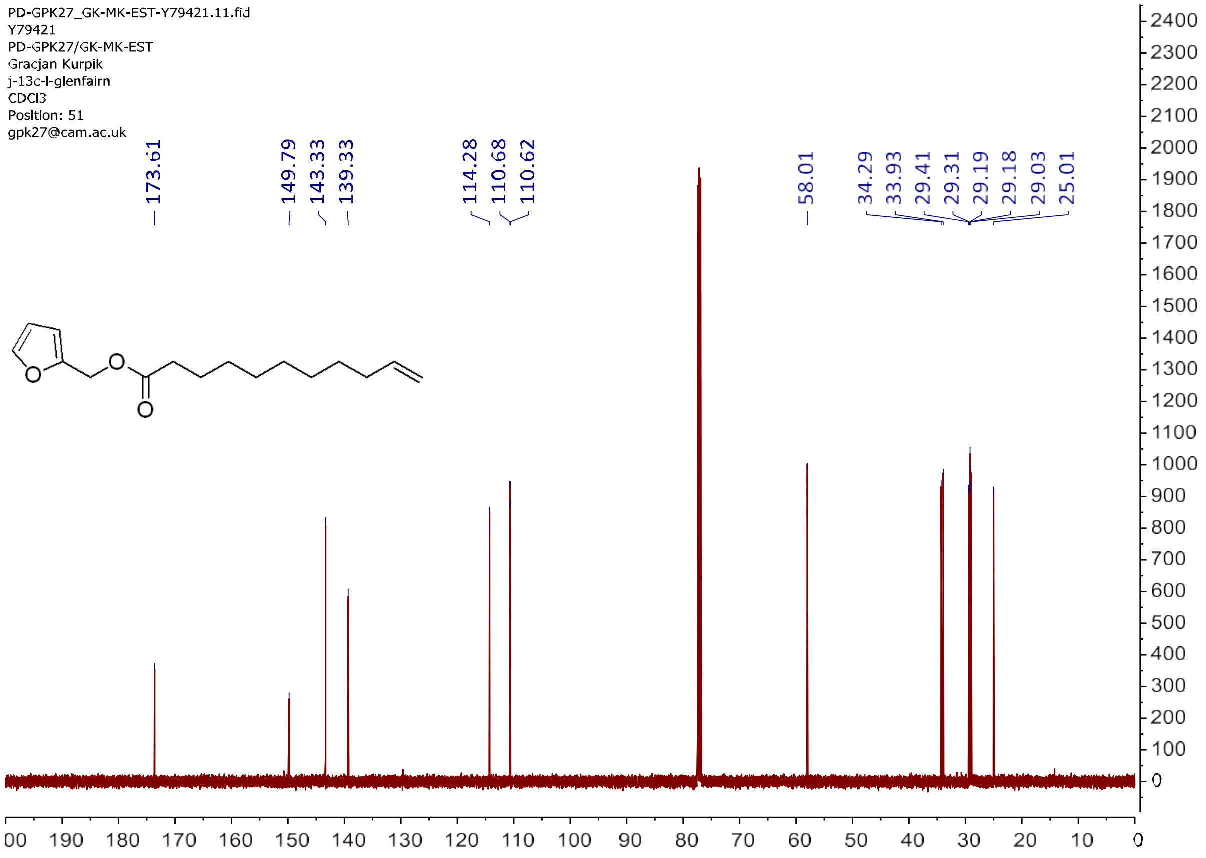
PD-GPK27_GK-MK-EST-Y79421.11.fid
Y79421
PD-GPK27/GK-MK-EST
Gracjan Kurpik
j-13c-l-glenfairn
CDCl3
Position: 51
gpk27@cam.ac.uk
173.61
149.79
143.33
139.33
114.28
110.68
110.62
58.01
34.29
33.93
29.41
29.31
29.19
29.18
29.03
25.01

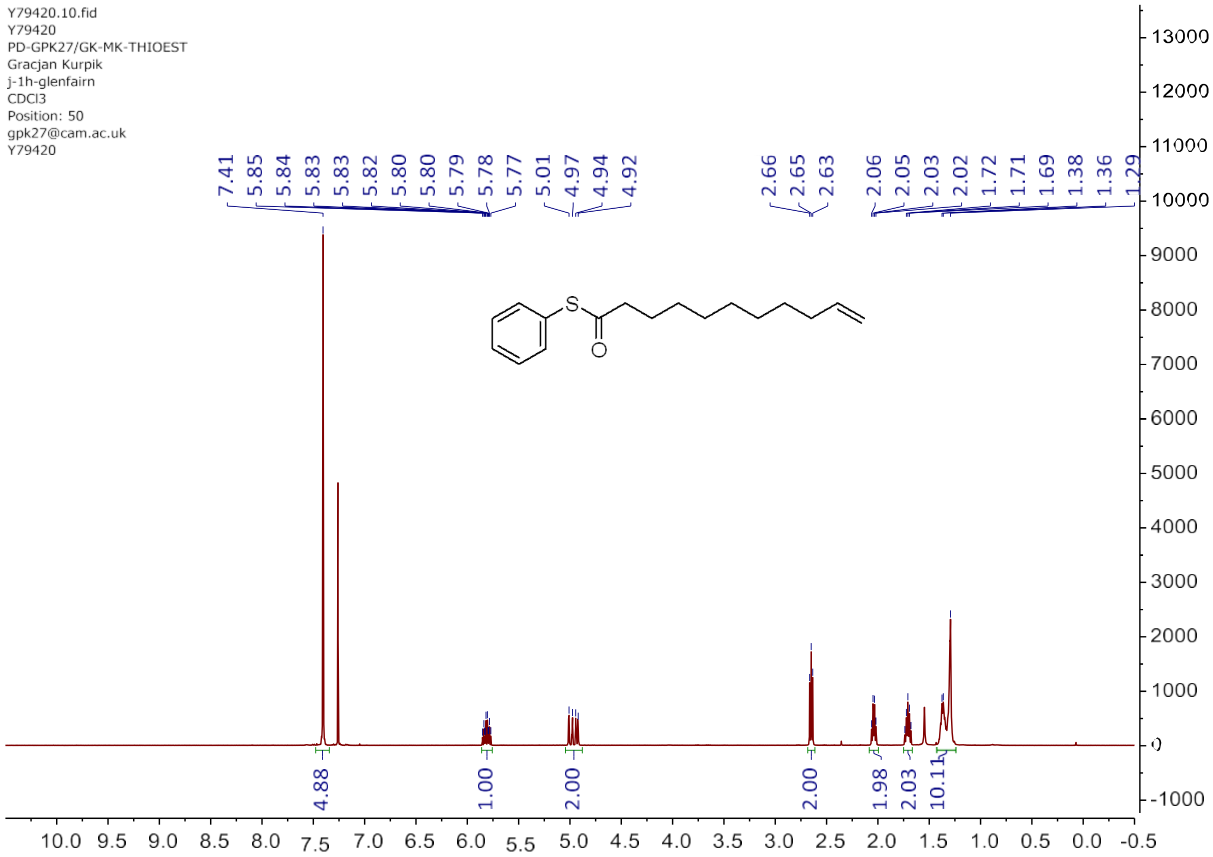
Y79420.10.fid
Y79420
PD-GPK27/GK-MK-THIOEST
Gracjan Kurpik
j-1h-glenfairn
CDCl3
Position: 50
gpk27@cam.ac.uk
Y79420
7.41 5.85 5.84 5.83 5.83 5.82 5.80 5.80 5.79 5.78 5.77 5.01 4.97 4.94 4.92 2.66 2.65 2.63 2.06 2.05 2.03 2.02 1.72 1.71 1.69 1.38 1.36 1.29
S
O
4.88 1.00 2.00 2.00 1.98 2.03 10.11
10.0 9.5 9.0 8.5 8.0 7.5 7.0 6.5 6.0 5.5 5.0 4.5 4.0 3.5 3.0 2.5 2.0 1.5 1.0 0.5 0.0 -0.5

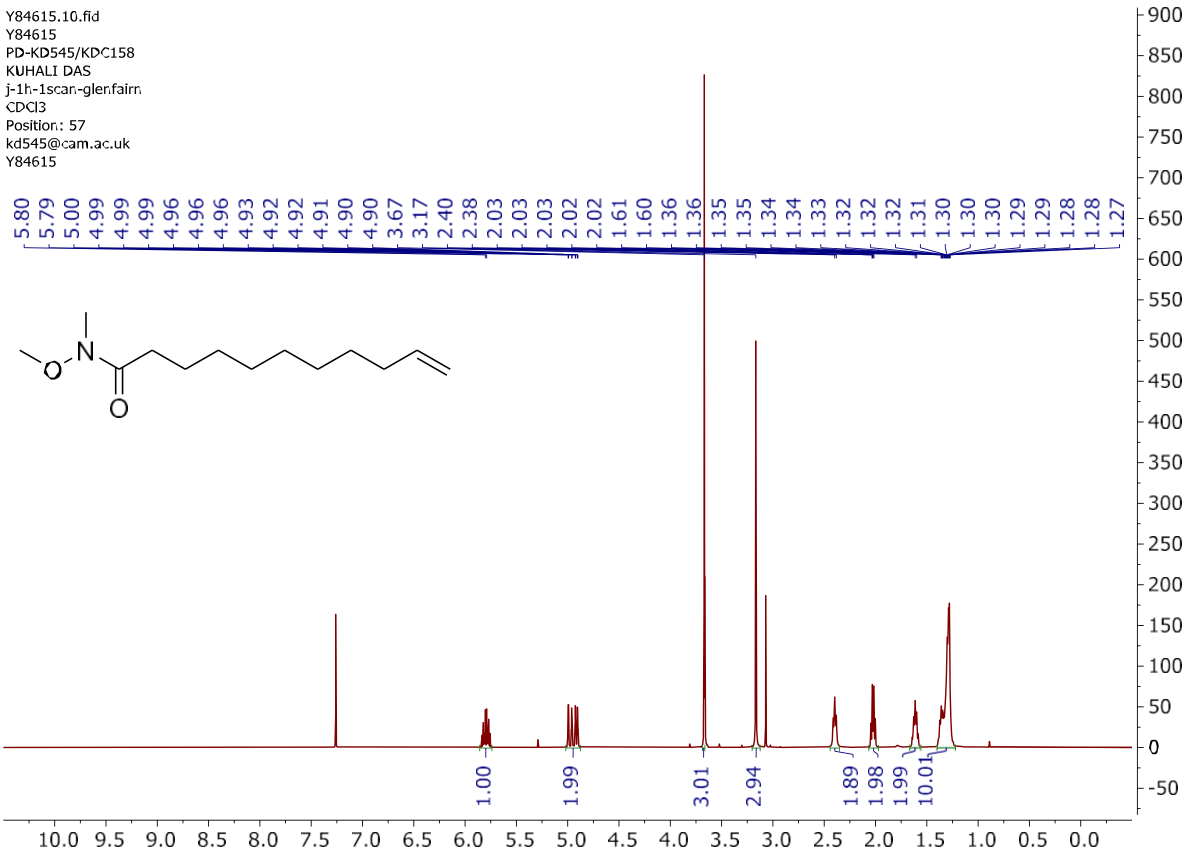
Y84615.10.fid
Y84615
PD-KD545/KDC158
KUHALI DAS
j-1h-1scan-glenfairn
CDCl3
Position: 57
kd545@cam.ac.uk
Y84615
5.80 5.79 5.00 4.99 4.99 4.99 4.96 4.96 4.93 4.92 4.92 4.91 4.90 4.90 3.67 3.17 2.40 2.38 2.03 2.03 2.03 2.02 2.02 1.61 1.60 1.36 1.36 1.35 1.35 1.34 1.34 1.33 1.32 1.32 1.32 1.31 1.30 1.30 1.30 1.29 1.29 1.28 1.28 1.27
N
O
O
1.00 1.99 3.01 2.94 1.89 1.98 1.99 10.01
10.0 9.5 9.0 8.5 8.0 7.5 7.0 6.5 6.0 5.5 5.0 4.5 4.0 3.5 3.0 2.5 2.0 1.5 1.0 0.5 0.0

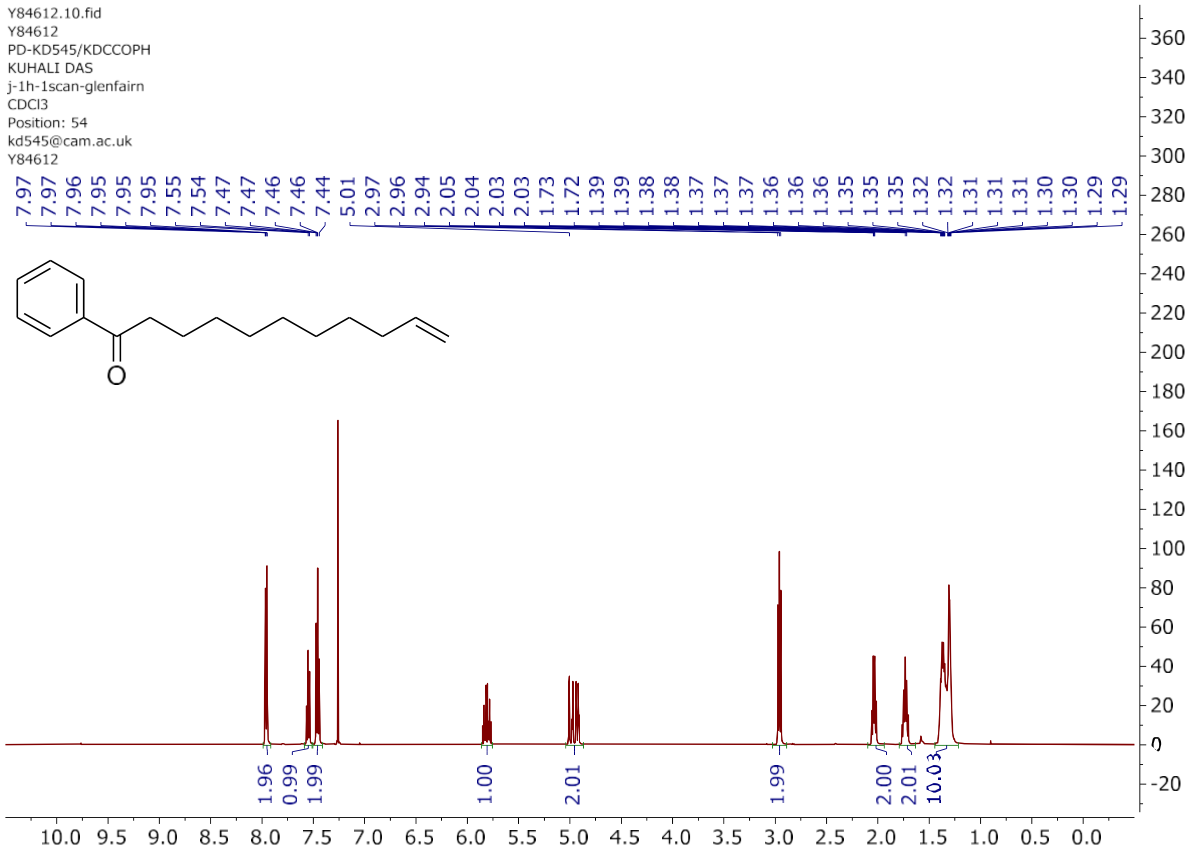
Y84612.10.fid
Y84612
PD-KD545/KDCCOPH
KUHALI DAS
j-1h-1scan-glenfairn
CDCl3
Position: 54
kd545@cam.ac.uk
Y84612
7.97 7.97 7.96 7.95 7.95 7.95 7.55 7.54 7.47 7.47 7.46 7.46 7.44 5.01 2.97 2.96 2.94 2.05 2.04 2.03 2.03 1.73 1.72 1.39 1.39 1.38 1.38 1.37 1.37 1.37 1.36 1.36 1.36 1.35 1.35 1.35 1.32 1.32 1.31 1.31 1.31 1.30 1.30 1.29 1.29
O
1.96 0.99 1.99 1.00 2.01 1.99 2.00 2.01 10.03

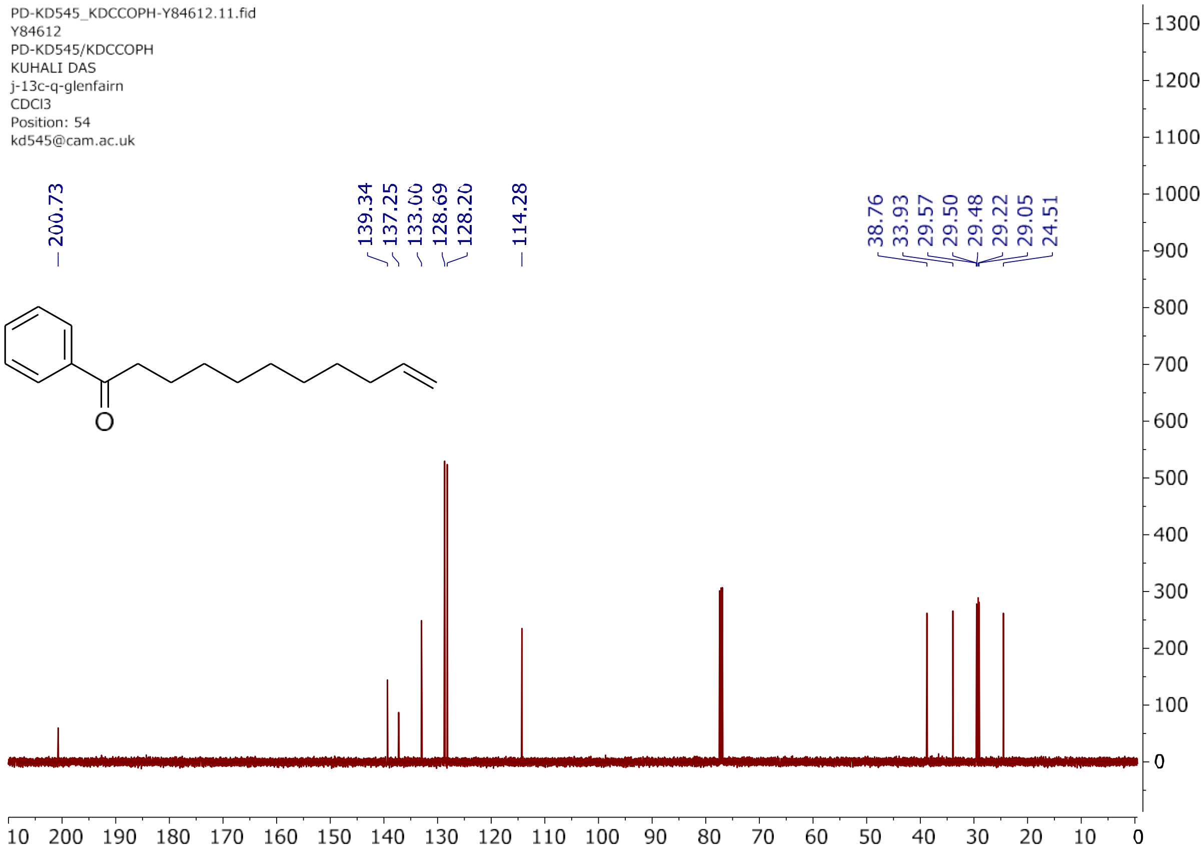
PD-KD545_KDCCOPH-Y84612.11.fid
Y84612
PD-KD545/KDCCOPH
KUHALI DAS
j-13c-q-glenfairn
CDCl3
Position: 54
kd545@cam.ac.uk
200.73
139.34 137.25 133.00 128.69 128.20
114.28
38.76 33.93 29.57 29.50 29.48 29.22 29.05 24.51
O

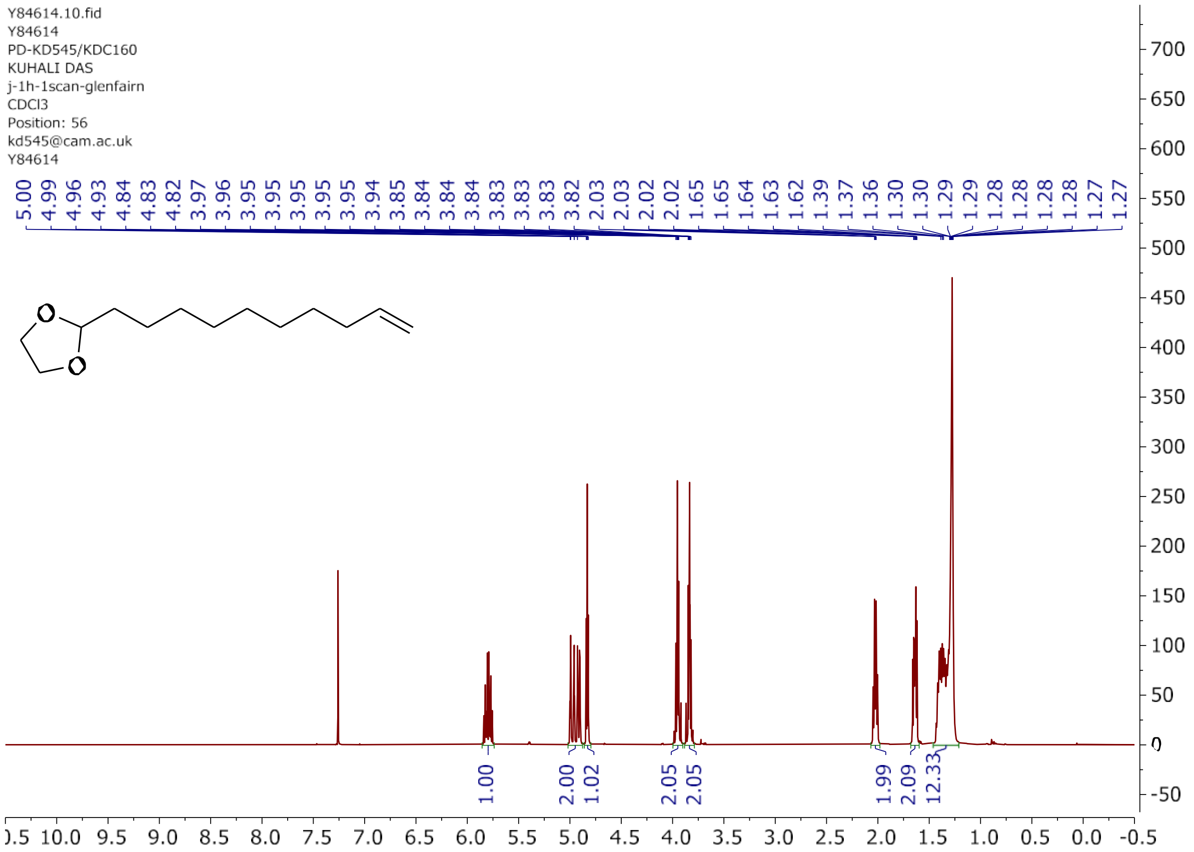
Y84614.10.fid
Y84614
PD-KD545/KDC160
KUHALI DAS
j-1h-1scan-glenfairn
CDCl3
Position: 56
kd545@cam.ac.uk
Y84614
5.00 4.99 4.96 4.93 4.84 4.83 4.82 3.97 3.96 3.95 3.95 3.95 3.95 3.95 3.94 3.85 3.84 3.84 3.84 3.83 3.83 3.83 3.82 2.03 2.03 2.02 2.02 1.65 1.65 1.64 1.63 1.62 1.39 1.37 1.36 1.30 1.30 1.29 1.29 1.28 1.28 1.28 1.28 1.27 1.27
1.00 2.00 1.02 2.05 2.05 1.99 2.09 12.33

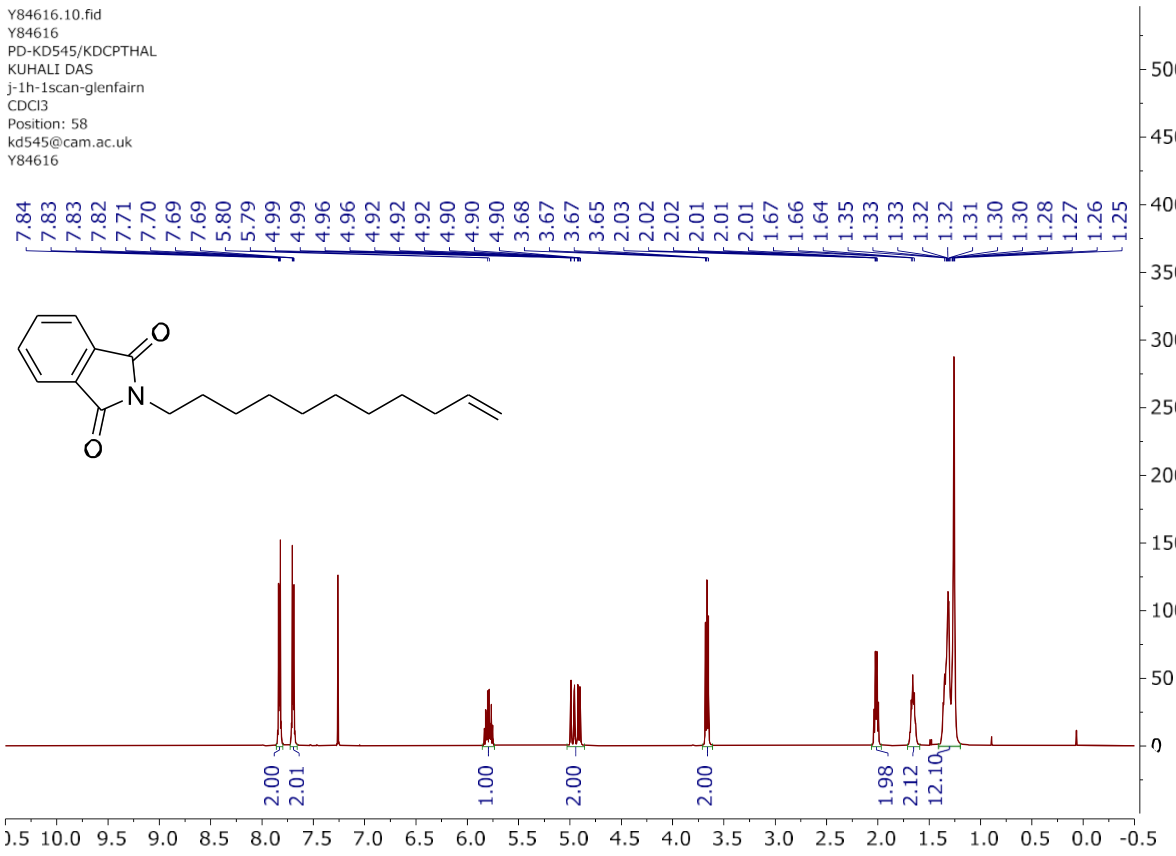
Y84616.10.fid
Y84616
PD-KD545/KDCPTHAL
KUHALI DAS
j-1h-1scan-glenfairn
CDCl3
Position: 58
kd545@cam.ac.uk
Y84616
7.84 7.83 7.83 7.82 7.71 7.70 7.69 7.69 5.80 5.79 4.99 4.99 4.96 4.96 4.92 4.92 4.92 4.90 4.90 4.90 3.68 3.67 3.67 3.65 2.03 2.02 2.02 2.01 2.01 2.01 1.67 1.66 1.64 1.35 1.33 1.33 1.32 1.32 1.31 1.30 1.30 1.28 1.27 1.26 1.25
O
N
O
2.00
2.01
1.00
2.00
2.00
1.98
2.12
12.10

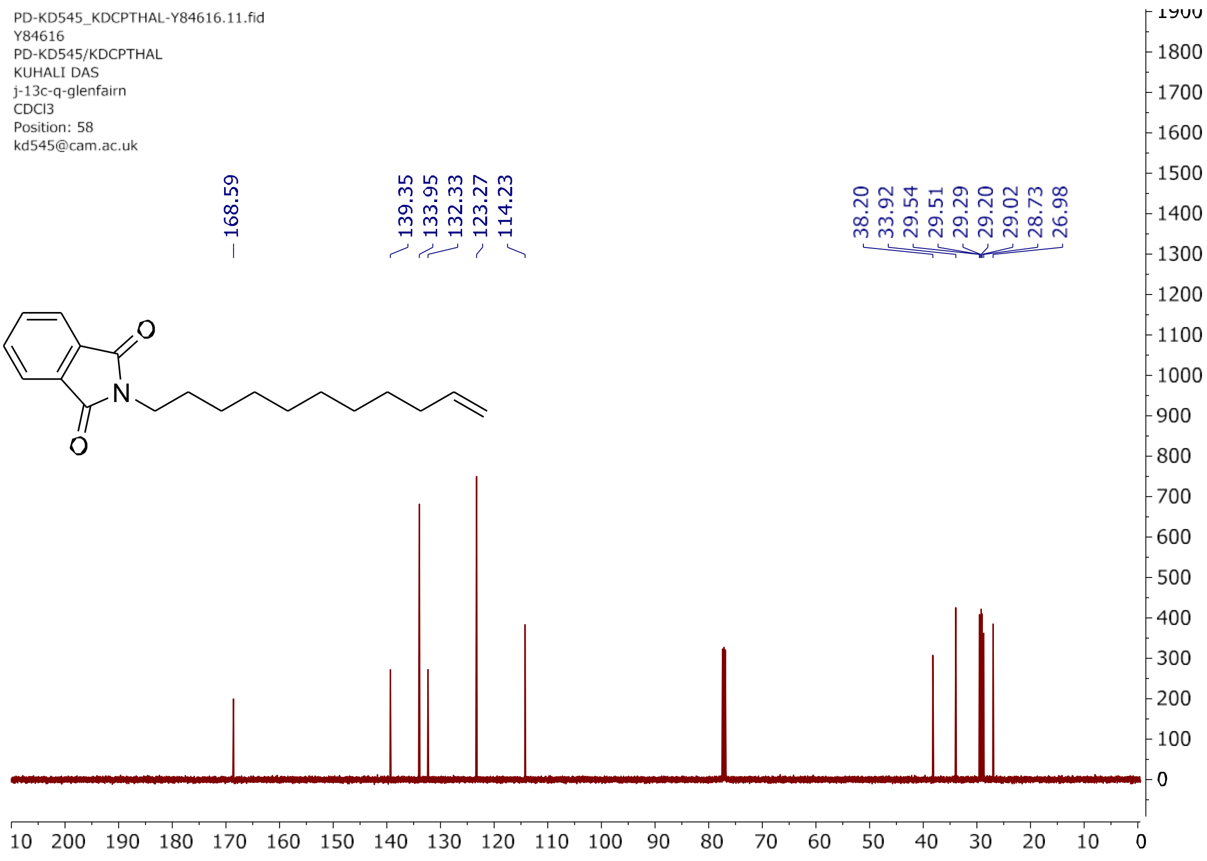
PD-KD545_KDCPTHAL-Y84616.11.fid
Y84616
PD-KD545/KDCPTHAL
KUHALI DAS
j-13c-q-glenfairn
CDCl3
Position: 58
kd545@cam.ac.uk
168.59
139.35 133.95 132.33 123.27 114.23
38.20 33.92 29.54 29.51 29.29 29.20 29.02 28.73 26.98
O
N
O

10. Single-crystal X-ray diffraction

X-ray data were collected on a Bruker D8-QUEST diffractometer, equipped with an Incoatec IμS microsource (either CuKα or MoKα) and a PHOTON-III detector operating in shutterless mode. The crystal was mounted on a MiTeGen crystal mount using inert polyfluoroether oil and the analysis was carried out under an Oxford Cryosystems open-flow N_2 Cryostream operating at 180(2) or 120(2) K The control and processing software was Bruker *APEX5* [Bruker, 2023]. The diffraction images were integrated using *SAINT* in *APEX5*, and a multi-scan correction was applied using *SADABS*. Unit-cell parameters were refined against all reflections. Structures were solved using *SHELXT*[31] and refined using *SHELXL*.[32]

Table S8.1. Summary of crystallographic data.

	**3au**	**$(L5)_2PdBr_2$**	**$(L10)PdBr_2$**
CCDC deposition number	2375113	2375114	2483759
Local data identifier	PD_B1_0011	pdms201008	PD_B1_0018
Chemical formula	$C_{37}H_{63}Cl_3O_2$	$C_{46}H_{62}Br_2O_8P_2Pd$	$C_{83}H_{141}Br_2O_9PPd$
Formula weight	646.22	1071.11	1580.14
Temperature / K	180(2)	120(2)	180(2)
Crystal system	monoclinic	monoclinic	orthorhombic
Space group	$P\ 2_1$	P c	$P\ 2_1\ 2_1\ 2_1$
a / Å	12.4294(5)	10.5199(4)	18.9341(5)
b / Å	9.1425(4)	8.4676(4)	21.2306(5)
c / Å	17.4417(7)	25.9204(12)	21.9621(6)
α / °	90	90	90
β / °	109.329(2)	96.088(2)	90
γ / °	90	90	90
Unit-cell volume / $Å^3$	1870.28(14)	2295.92(18)	8828.4(4)
Z	2	2	4
Calc. density / g cm^{-3}	1.148	1.549	1.189
F(000)	704	1096	3368
Radiation type	Cu Kα	Mo Kα	Cu Kα
Absorption coefficient / mm^{-1}	2.427	2.266	3.306
Crystal size / mm^3	0.18 x 0.14 x 0.06	0.15 x 0.12 x 0.11	0.22 x 0.12 x 0.10
2-Theta range / degrees	5.37–133.58	3.89–55.88	6.16–133.19
Completeness to max 2-theta	0.996	0.997	0.999
No. of reflections measured	32303	67405	88988
No. of independent reflections	6578	10680	15567
R(int)	0.0854	0.0902	0.0486
No. parameters / restraints	388 / 28	543 / 2	873 / 558
Final R1 values (I > 2σ(I))	0.0816	0.0444	0.0358
Final wR(F^2) values (all data)	0.2406	0.0936	0.0979
Goodness-of-fit on F^2	1.023	1.076	1.034
Largest difference peak & hole / e $Å^{-3}$	0.619, –0.491	1.178, –0.839	0.904, –0.615
Flack parameter	0.012(13)	0.434(12)*	–0.014(3)

* Refined as an inversion twin.

Refinement details

Refinements of **3au** and **$(L5)_2PdBr_2$** were straightforward. Refinement of **$(L10)PdBr_2$** was more challenging due to the inclusion of several methyl t-butyl ether solvent molecules, which are resolved to varying degrees. One molecule (containing O1S) is disordered over two orientations but refines well, consistent with having a regular position due to accepting a hydrogen bond from the hydroxyl group on **L10**. The solvent molecule containing O2S refines adequately with two orientations and anisotropic ADPs. Three other solvent molecules are harder to model as discrete disorder components, and anisotropic ADPs become quite large. These molecules are therefore modelled with a common isotropic displacement parameters for all non-H atoms. For comparison: omitting the five solvent molecules entirely and applying the *SQUEEZE* algorithm (A. L. Spek, Acta Cryst. (2015), C71, 9–18) yields R1 = 0.022, wR2 = 0.055.

For **3au** and **$(L10)PdBr_2$**, the absolute structure is determined satisfactorily. For **$(L5)_2PdBr_2$**, the refined Flack parameter indicates an inversion twin. The **$(L5)_2PdBr_2$** complex displays inversion symmetry (point group C_i), but the inversion centres are not retained in the space-group symmetry of the crystal.

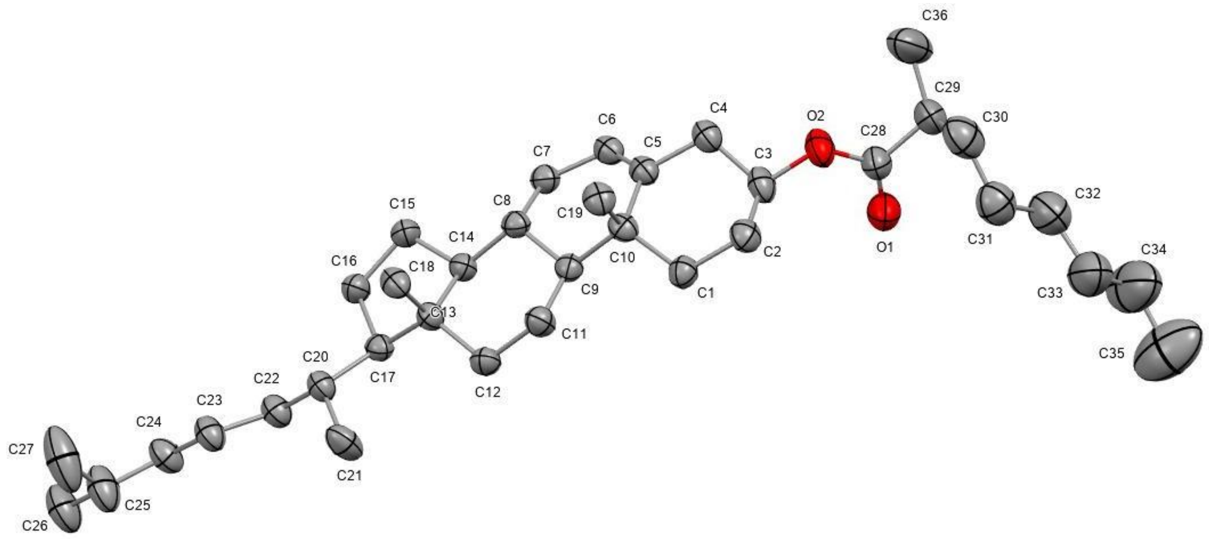

Fig. S15. *Molecular structure of **3au** with displacement ellipsoids at 50% probability (H atoms and chloroform solvent molecules are omitted).*

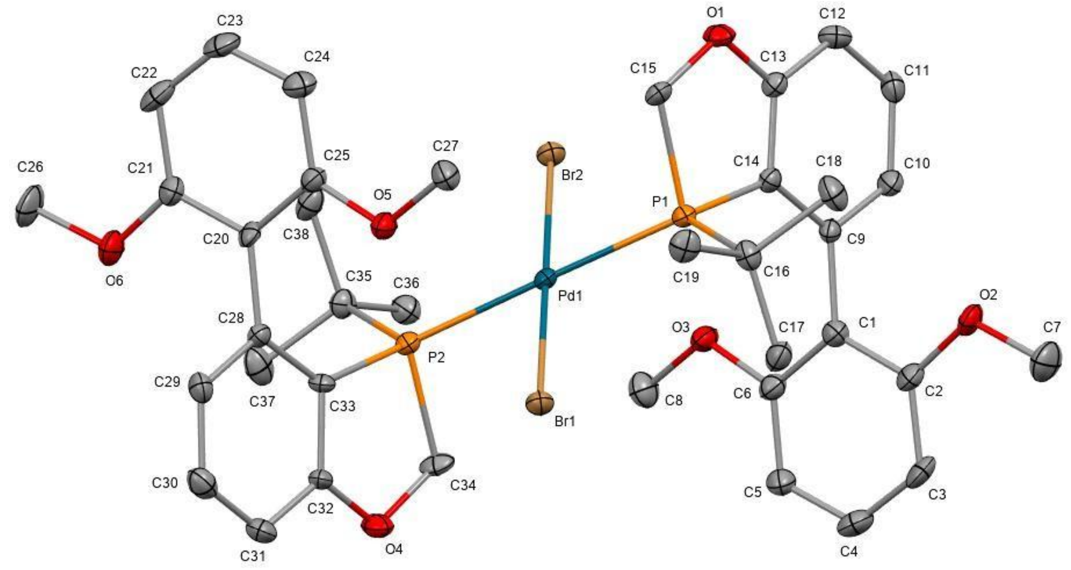

Fig. S16. *Molecular structure of* ***(L5)₂PdBr₂*** *with displacement ellipsoids at 50% probability (H atoms and THF solvent molecules omitted). The complex displays (non-crystallographic) inversion symmetry (point group* C_i*).*

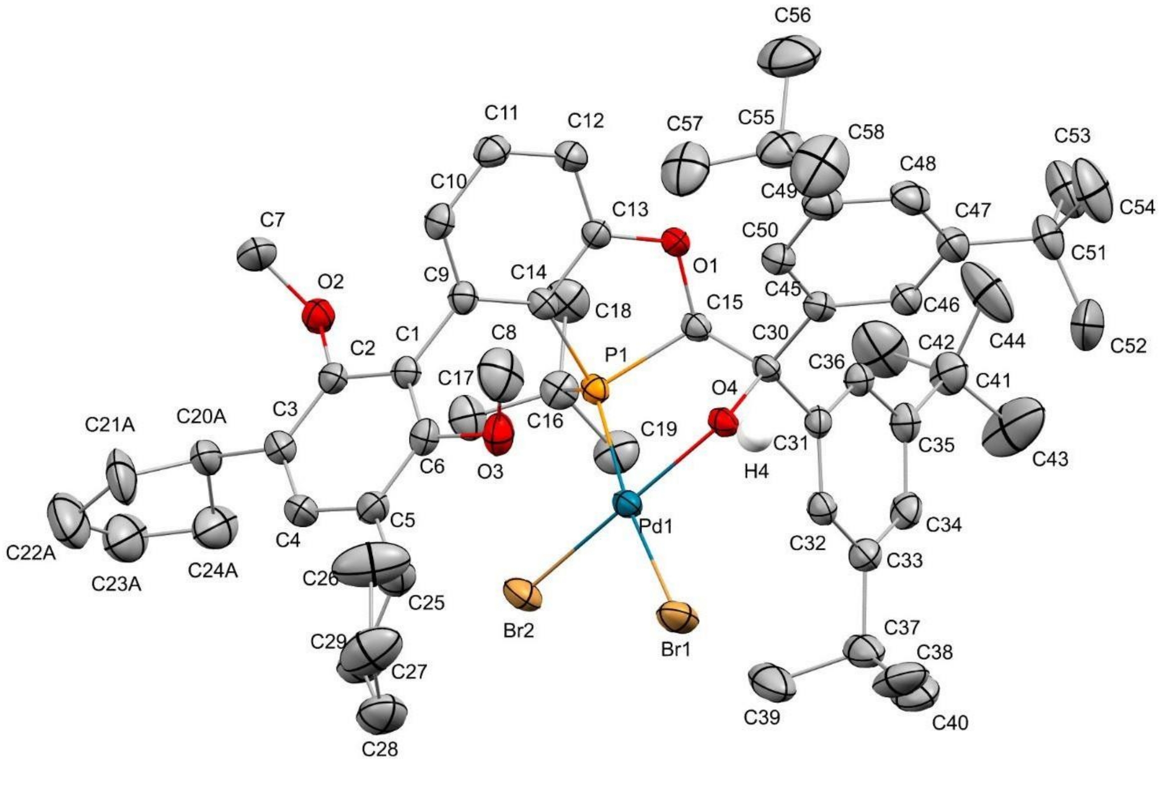

Fig. S17. *Molecule structure of* ***(L10)PdBr₂*** *with displacement ellipsoids at 50% probability (H atoms omitted). Five methyl t-butyl ether solvent molecules are omitted from the diagram, plus a second disorder component for the cyclopentyl ring C20–C24.*

11. References

(1) Ebner, D. C.; Trend, R. M.; Genet, C.; McGrath, M. J.; O'Brien, P.; Stoltz, B. M. Palladium-Catalyzed Enantioselective Oxidation of Chiral Secondary Alcohols: Access to Both Enantiomeric Series. *Angew. Chem. Int. Ed.* **2008**, *47* (34), 6367–6370. https://doi.org/10.1002/anie.200801865.

(2) Yang, H.; Sun, J.; Gu, W.; Tang, W. Enantioselective Cross-Coupling for Axially Chiral Tetra-Ortho-Substituted Biaryls and Asymmetric Synthesis of Gossypol. *J. Am. Chem. Soc.* **2020**, *142* (17), 8036–8043. https://doi.org/10.1021/jacs.0c02686.

(3) Li, H.; Dong, K.; Jiao, H.; Neumann, H.; Jackstell, R.; Beller, M. The Scope and Mechanism of Palladium-Catalysed Markovnikov Alkoxycarbonylation of Alkenes. *Nat. Chem.* **2016**, *8* (12), 1159–1166. https://doi.org/10.1038/nchem.2586.

(4) Yao, Y.-H.; Zou, X.-J.; Wang, Y.; Yang, H.-Y.; Ren, Z.-H.; Guan, Z.-H. Palladium-Catalyzed Asymmetric Markovnikov Hydroxycarbonylation and Hydroalkoxycarbonylation of Vinyl Arenes: Synthesis of 2-Arylpropanoic Acids. *Angew. Chem. Int. Ed.* **2021**, *60* (43), 23117–23122. https://doi.org/10.1002/anie.202107856.

(5) Mao, J.; Liu, F.; Wang, M.; Wu, L.; Zheng, B.; Liu, S.; Zhong, J.; Bian, Q.; Walsh, P. J. Cobalt–Bisoxazoline-Catalyzed Asymmetric Kumada Cross-Coupling of Racemic α-Bromo Esters with Aryl Grignard Reagents. *J. Am. Chem. Soc.* **2014**, *136* (50), 17662–17668. https://doi.org/10.1021/ja5109084.

(6) Loesche, A.-C.; Brückner, R. Dienolates of Cycloalkenones and α,β-Unsaturated Esters Form Diels–Alder Adducts by a Michael/Michael-Tandem Reaction Rather Than in One Step. *Eur. J. Org. Chem.* **2019**, *2019* (2–3), 562–573. https://doi.org/10.1002/ejoc.201801193.

(7) Wang, H.; Cheng, F.; Li, M.; Peng, W.; Qu, J. Reactivity and Kinetics of Vinyl Sulfone-Functionalized Self-Assembled Monolayers for Bioactive Ligand Immobilization. *Langmuir* **2015**, *31* (11), 3413–3421. https://doi.org/10.1021/la504087a.

(8) Deng, Q.; Shen, Y.; Zhu, H.; Tu, T. A Magnetic Nanoparticle-Supported N-Heterocyclic Carbene-Palladacycle: An Efficient and Recyclable Solid Molecular Catalyst for Suzuki–Miyaura Cross-Coupling of 9-Chloroacridine. *Chem. Commun.* **2017**, *53* (97), 13063–13066. https://doi.org/10.1039/C7CC06958H.

(9) Cheng, C.; Shi, J. X.; Kang, E.-H.; Nelson, T. F.; Sander, M.; McNeill, K.; Hartwig, J. F. Polymers from Plant Oils Linked by Siloxane Bonds for Programmed Depolymerization. *J. Am. Chem. Soc.* **2024**, *146* (18), 12645–12655. https://doi.org/10.1021/jacs.4c01982.

(10) Wu, J.; Krische, M. J. β-Hydroxy Esters as Malonic Semialdehyde Proelectrophiles in Enantioselective Butadiene-Mediated Crotylation: Total Synthesis of Octalactins A and B. *Org. Lett.* **2024**, *26* (22), 4830–4834. https://doi.org/10.1021/acs.orglett.4c01644.

(11) Kumar, R.; Kawasaki, H.; Harada, T. Enantioselective Alkylation of Aldehydes Using Functionalized Alkylboron Reagents Catalyzed by a Chiral Titanium Complex. *Org. Lett.* **2013**, *15* (16), 4198–4201. https://doi.org/10.1021/ol4019248.

(12) Blass, B. E.; Gao, R.; Blattner, K. M.; Gordon, J. C.; Pippin, D. A.; Canney, D. J. Synthesis and Evaluation of Novel, Selective, Functionalized γ-Butyrolactones as Sigma-2 Ligands. *Med. Chem. Res.* **2022**, *31* (2), 337–349. https://doi.org/10.1007/s00044-021-02831-5.

(13) Tappin, N. D. C.; Michalska, W.; Rohrbach, S.; Renaud, P. Cyclopropanation of Terminal Alkenes through Sequential Atom-Transfer Radical Addition/1,3-Elimination. *Angew. Chem. Int. Ed.* **2019**, *58* (40), 14240–14244. https://doi.org/10.1002/anie.201907962.

(14) Nakagawa, M.; Matsuki, Y.; Nagao, K.; Ohmiya, H. A Triple Photoredox/Cobalt/Brønsted Acid Catalysis Enabling Markovnikov Hydroalkoxylation of Unactivated Alkenes. *J. Am. Chem. Soc.* **2022**, *144* (18), 7953–7959. https://doi.org/10.1021/jacs.2c00527.

(15) Ranganathan, D.; Ranganathan, S.; Mehrotra, M. M. The Synthesis of PGF1α by Re-Structuring of Castor Oil. *Tetrahedron* **1980**, *36* (12), 1869–1875. https://doi.org/10.1016/0040-4020(80)80089-5.

(16) Wang, M.-M.; Ning, X.-S.; Qu, J.-P.; Kang, Y.-B. Dehydrogenative Synthesis of Linear α,β-Unsaturated Aldehydes with Oxygen at Room Temperature Enabled by tBuONO. *ACS Catal.* **2017**, *7* (6), 4000–4003. https://doi.org/10.1021/acscatal.7b01008.

(17) Rivera-Chao, E.; Olivier, W. J.; Tilby, M. J.; Leonori, D. Photocatalytic Hydrogenation of Alkenes Using Ammonia-Borane. *Chem* **2026**, *12* (1). https://doi.org/10.1016/j.chempr.2025.102711.

(18) Imai, M.; Tanaka, M.; Nagumo, S.; Kawahara, N.; Suemune, H. Nitrile-Promoted Rh-Catalyzed Intermolecular Hydroacylation of Olefins with Salicylaldehyde. *J. Org. Chem.* **2007**, *72* (7), 2543–2546. https://doi.org/10.1021/jo062501u.

(19) Vilela, C.; Silvestre, A. J. D.; Gandini, A. Thermoreversible Nonlinear Diels-Alder Polymerization of Furan/Plant Oil Monomers. *J. Polym. Sci. Part Polym. Chem.* **2013**, *51* (10), 2260–2270. https://doi.org/10.1002/pola.26610.

(20) Scarborough, R. M. Jr.; Toder, B. H.; Smith, A. B. I. A Stereospecific Total Synthesis of (.+-.)-Methylenomycin A and Its Epimer, (.+-.)-Epimethylenomycin A. *J. Am. Chem. Soc.* **1980**, *102* (11), 3904–3913. https://doi.org/10.1021/ja00531a037.

(21) Böttcher, T.; Sieber, S. A. β-Lactones as Privileged Structures for the Active-Site Labeling of Versatile Bacterial Enzyme Classes. *Angew. Chem. Int. Ed.* **2008**, *47* (24), 4600–4603. https://doi.org/10.1002/anie.200705768.

(22) Sui, G.; Lv, Q.; Song, X.; Guo, H.; Dai, J.; Ren, L.; Lee, C.-S.; Zhou, W.; Hao, H.-D. Chemoselective Reduction of Aldehydes via a Combination of NaBH4 and Acetylacetone. *New J. Chem.* **2019**, *43* (39), 15793–15796. https://doi.org/10.1039/C9NJ03210J.

(23) Suresh, R.; Simlandy, A. K.; Mukherjee, S. A Catalytic Enantioselective Iodocyclization Route to Dihydrooxazines. *Org. Lett.* **2018**, *20* (5), 1300–1303. https://doi.org/10.1021/acs.orglett.8b00002.

(24) Lipshutz, B. H.; Ghorai, S.; Leong, W. W. Y.; Taft, B. R.; Krogstad, D. V. Manipulating Micellar Environments for Enhancing Transition Metal-Catalyzed Cross-Couplings in Water at Room Temperature. *J. Org. Chem.* **2011**, *76* (12), 5061–5073. https://doi.org/10.1021/jo200746y.

(25) Wang, Z.-X.; Xu, Y.; Gilmour, R. Regioselective Fluorination of Allenes Enabled by I(I)/I(III) Catalysis. *Nat. Commun.* **2024**, *15* (1), 5770. https://doi.org/10.1038/s41467-024-50227-x.

(26) Jean, M.; Renault, J.; Uriac, P.; Capet, M.; van de Weghe, P. Unexpected Formation of Aryl Ketones by Palladium-Catalyzed Coupling of Aryl Bromides with Vinylic Acetates. *Org. Lett.* **2007**, *9* (18), 3623–3625. https://doi.org/10.1021/ol7015065.

(27) Li, H.; Hou, Y.; Liu, C.; Lai, Z.; Ning, L.; Szostak, R.; Szostak, M.; An, J. Pentafluorophenyl Esters: Highly Chemoselective Ketyl Precursors for the Synthesis of α,α-Dideuterio Alcohols Using SmI2 and D2O as a Deuterium Source. *Org. Lett.* **2020**, *22* (4), 1249–1253. https://doi.org/10.1021/acs.orglett.9b04383.

(28) Loup, J.; Larin, E. M.; Lautens, M. Iron-Catalyzed Reductive Cyclization by Hydromagnesiation: A Modular Strategy Towards N-Heterocycles. *Angew. Chem. Int. Ed.* **2021**, *60* (41), 22345–22351. https://doi.org/10.1002/anie.202106996.

(29) Wang, Q.; Jung, H.; Kim, D.; Chang, S. Iridium-Catalyzed Migratory Terminal C(Sp3)–H Amidation of Heteroatom-Substituted Internal Alkenes via Olefin Chain Walking. *J. Am. Chem. Soc.* **2023**, *145* (45), 24940–24951. https://doi.org/10.1021/jacs.3c09679.

(30) Negishi, E.; Nguyen, T.; Maye, J. P.; Choueiri, D.; Suzuki, N.; Takahashi, T. Factors Affecting the Unusual Reactivity Order in the β-Hydrogen Abstraction of Dialkylzirconocenes. *Chem. Lett.* **1992**, *21* (12), 2367–2370. https://doi.org/10.1246/cl.1992.2367.

(31) Sheldrick, G. M. SHELXT – Integrated Space-Group and Crystal-Structure Determination. *Acta Crystallogr. Sect. Found. Adv.* **2015**, *71* (1), 3–8. https://doi.org/10.1107/S2053273314026370.

(32) Sheldrick, G. M. Crystal Structure Refinement with SHELXL. *Acta Crystallogr. Sect. C Struct. Chem.* **2015**, *71* (1), 3–8. https://doi.org/10.1107/S2053229614024218.
